# Review of lattice results concerning low-energy particle physics

**DOI:** 10.1140/epjc/s10052-014-2890-7

**Published:** 2014-09-17

**Authors:** FLAG Working Group, S. Aoki, Y. Aoki, C. Bernard, T. Blum, G. Colangelo, M. Della Morte, S. Dürr, A. X. El-Khadra, H. Fukaya, R. Horsley, A. Jüttner, T. Kaneko, J. Laiho, L. Lellouch, H. Leutwyler, V. Lubicz, E. Lunghi, S. Necco, T. Onogi, C. Pena, C. T. Sachrajda, S. R. Sharpe, S. Simula, R. Sommer, R. S. Van de Water, A. Vladikas, U. Wenger, H. Wittig

**Affiliations:** 1 Yukawa Institute for Theoretical Physics, Kyoto University, Kitashirakawa Oiwakecho, Sakyo-ku, Kyoto, 606-8502 Japan; 2 Kobayashi-Maskawa Institute for the Origin of Particles and the Universe (KMI), Nagoya University, Nagoya, 464-8602 Japan; 3 RIKEN BNL Research Center, Brookhaven National Laboratory, Upton, NY 11973 USA; 4 Department of Physics, Washington University, Saint Louis, MO 63130 USA; 5 Physics Department, University of Connecticut, Storrs, CT 06269-3046 USA; 6 Albert Einstein Center for Fundamental Physics, Institut für theoretische Physik, Universität Bern, Sidlerstr. 5, 3012 Bern, Switzerland; 7 CP3-Origins & Danish IAS, University of Southern Denmark, Campusvej 55, 5230 Odense M, Denmark; 8 IFIC (CSIC), c/ Catedrático José Beltrán, 2, 46980 Paterna, Spain; 9 Bergische Universität Wuppertal, Gaußstraße 20, 42119 Wuppertal, Germany; 10 Jülich Supercomputing Center, Forschungszentrum Jülich, 52425 Jülich, Germany; 11 Department of Physics, University of Illinois, Urbana, IL 61801 USA; 12 Department of Physics, Osaka University, Toyonaka, Osaka, 560-0043 Japan; 13 School of Physics, University of Edinburgh, Edinburgh, EH9 3JZ UK; 14 School of Physics and Astronomy, University of Southampton, Southampton, SO17 1BJ UK; 15 High Energy Accelerator Research Organization (KEK), Ibaraki, 305-0801 Japan; 16 SUPA, Department of Physics and Astronomy, University of Glasgow, Glasgow, G12 8QQ UK; 17 Aix-Marseille Université, CNRS, CPT, UMR 7332, 13288 Marseille, France; 18 Université de Toulon, CNRS, CPT, UMR 7332, 83957 La Garde, France; 19 Dipartimento di Matematica e Fisica, Università Roma Tre, Via della Vasca Navale 84, 00146 Rome, Italy; 20 INFN, Sezione di Roma Tre, Via della Vasca Navale 84, 00146 Rome, Italy; 21 Physics Department, Indiana University, Bloomington, IN 47405 USA; 22 Instituto de Física Teórica UAM/CSIC and Departamento de Física Teórica, Universidad Autónoma de Madrid, Cantoblanco, 28049 Madrid, Spain; 23 Physics Department, University of Washington, Seattle, WA 98195-1560 USA; 24 NIC @ DESY, Platanenallee 6, 15738 Zeuthen, Germany; 25 Fermi National Accelerator Laboratory, Batavia, IL 60510 USA; 26 INFN, Sezione di Tor Vergata, c/o Dipartimento di Fisica, Università di Roma Tor Vergata, Via della Ricerca Scientifica 1, 00133 Rome, Italy; 27 PRISMA Cluster of Excellence, Institut für Kernphysik and Helmholtz Institute Mainz, University of Mainz, 55099 Mainz, Germany; 28 Present Address: Department of Physics, Syracuse University, Syracuse, New York USA

## Abstract

We review lattice results related to pion, kaon, $D$- and $B$-meson physics with the aim of making them easily accessible to the particle-physics community. More specifically, we report on the determination of the light-quark masses, the form factor $f_+(0)$, arising in semileptonic $K \rightarrow \pi$ transition at zero momentum transfer, as well as the decay-constant ratio $f_K/f_\pi$ of decay constants and its consequences for the CKM matrix elements $V_{us}$ and $V_{ud}$. Furthermore, we describe the results obtained on the lattice for some of the low-energy constants of $\hbox {SU}(2)_L\times \hbox {SU}(2)_R$ and $\hbox {SU}(3)_L\times \hbox {SU}(3)_R$ Chiral Perturbation Theory and review the determination of the $B_K$ parameter of neutral kaon mixing. The inclusion of heavy-quark quantities significantly expands the FLAG scope with respect to the previous review. Therefore, we focus here on $D$- and $B$-meson decay constants, form factors, and mixing parameters, since these are most relevant for the determination of CKM matrix elements and the global CKM unitarity-triangle fit. In addition we review the status of lattice determinations of the strong coupling constant $\alpha _\mathrm{s}$.

## Introduction

Flavour physics provides an important opportunity for exploring the limits of the Standard Model of particle physics and for constraining possible extensions of theories that go beyond it. As the LHC explores a new energy frontier and as experiments continue to extend the precision frontier, the importance of flavour physics will grow, both in terms of searches for signatures of new physics through precision measurements and in terms of attempts to unravel the theoretical framework behind direct discoveries of new particles. A major theoretical limitation consists in the precision with which strong interaction effects can be quantified. Large-scale numerical simulations of lattice QCD allow for the computation of these effects from first principles. The scope of the Flavour Lattice Averaging Group (FLAG) is to review the current status of lattice results for a variety of physical quantities in low-energy physics. Set up in November 2007,^1^ it comprises experts in Lattice Field Theory and Chiral Perturbation Theory. Our aim is to provide an answer to the frequently posed question “What is currently the best lattice value for a particular quantity?”, in a way which is readily accessible to non-lattice-experts. This is generally not an easy question to answer; different collaborations use different lattice actions (discretisations of QCD) with a variety of lattice spacings and volumes, and with a range of masses for the $u$- and $d$-quarks. Not only are the systematic errors different, but also the methodology used to estimate these uncertainties varies between collaborations. In the present work we summarise the main features of each of the calculations and provide a framework for judging and combining the different results. Sometimes it is a single result which provides the “best” value; more often it is a combination of results from different collaborations. Indeed, the consistency of values obtained using different formulations adds significantly to our confidence in the results.

The first edition of the FLAG review was published in 2011 [1]. It was limited to lattice results related to pion and kaon physics: light-quark masses ($u$-, $d$- and $s$-flavours), the form factor $f_+(0)$ arising in semileptonic $K \rightarrow \pi$ transitions at zero momentum transfer and the decay constant ratio $f_K/f_\pi$, as well as their implications for the CKM matrix elements $V_{us}$ and $V_{ud}$. Furthermore, results were reported for some of the low-energy constants of $\hbox {SU}(2)_L \otimes \hbox {SU}(2)_R$ and $\hbox {SU}(3)_L \otimes \hbox {SU}(3)_R$ Chiral Perturbation Theory and the $B_K$ parameter of neutral kaon mixing. Results for all of these quantities have been updated in the present paper. Moreover, the scope of the present review has been extended by including lattice results related to $D$- and $B$-meson physics. We focus on $B$- and $D$-meson decay constants, form factors, and mixing parameters, which are most relevant for the determination of CKM matrix elements and the global CKM unitarity-triangle fit. Last but not least, the current status of lattice results on the QCD coupling $\alpha _\mathrm{s}$ is also reviewed. Bottom- and charm-quark masses, though important parametric inputs to Standard Model calculations, have not been covered in the present edition. They will be included in a future FLAG report.

Our plan is to continue providing FLAG updates, in the form of a peer-reviewed paper, roughly on a biannual basis. This effort is supplemented by our more frequently updated website http://itpwiki.unibe.ch/flag, where figures as well as pdf-files for the individual sections can be downloaded. The papers reviewed in the present edition have appeared before the closing date 30 November 2013.

Finally, we draw attention to a particularly important point. As stated above, our aim is to make lattice QCD results easily accessible to non-lattice-experts and we are well aware that it is likely that some readers will only consult the present paper and not the original lattice literature. We consider it very important that this paper is not the only one which gets cited when the lattice results which are discussed and analysed here are quoted. Readers who find the review and compilations offered in this paper useful are therefore kindly requested to also cite the original sources. The bibliography at the end of this paper should make this task easier. Indeed we hope that the bibliography will be one of the most widely used elements of the whole paper.

This review is organised as follows. In the remainder of Sect. 1 we summarise the composition and rules of FLAG, describe the goals of the FLAG effort and general issues that arise in modern lattice calculations. For the reader’s convenience, Table 1 summarises the main results (averages and estimates) of the present review. In Sect. 2 we explain our general methodology for evaluating the robustness of lattice results which have appeared in the literature. We also describe the procedures followed for combining results from different collaborations in a single average or estimate (see Sect. 2.2 for our use of these terms). The rest of the paper consists of sections, each of which is dedicated to a single (or groups of closely connected) physical quantity(ies). Each of these sections is accompanied by an Appendix with explicatory notes.

**Table 1:** Summary of the main results of this review, grouped in terms of $N_\mathrm{f}$, the number of dynamical quark flavours in lattice simulations. Quark masses and the quark condensate are given in the ${\overline{\mathrm{MS}}}$ scheme at running scale $\mu =2\,\mathrm{GeV}$; the other quantities listed are specified in the quoted sections. The columns marked  indicate the number of results that enter our averages for each quantity. We emphasise that these numbers only give a very rough indication of how thoroughly the quantity in question has been explored on the lattice and recommend to consult the detailed tables and figures in the relevant section for more significant information For explanations on the source of the quoted errors for each quantity, the reader is advised to consult the corresponding section, as indicated in the second column

Quantity	Sect.		$N_\mathrm{f} = 2 + 1 + 1$		$N_\mathrm{f}\mathrm{}=2+1$		$N_\mathrm{f}=2$
$m_{s}$ (MeV)	3.3			3	93.8 (1.5) (1.9)	2	101 (3)
$m_{ud}$ (MeV)	3.3			3	3.42 (6) (7)	1	3.6 (2)
$m_{s}/m_{ud}$	3.3			3	27.46 (15) (41)	1	28.1 (1.2)
$m_{d}$ (MeV)	3.4				4.68 (14) (7)		4.80 (23)
$m_{u}$ (MeV)	3.4				2.16 (9) (7)		2.40 (23)
$m_{u}/m_{d}$	3.4				0.46 (2) (2)		0.50 (4)
$f_+^{K\pi } (0)$	4.3			1	0.9661 (32)	1	0.9560 (57) (62)
$f_{K^+}/f_{\pi ^+}$	4.3	2	1.194 (5)	4	1.192 (5)	1	1.205 (6) (17)
$f_K$ (MeV)	4.6			3	156.3 (0.9)	1	158.1 (2.5)
$f_\pi$ (MeV)	4.6			3	130.2 (1.4)		
$\Sigma$ (MeV)	5.1			2	271 (15)	1	269 (8)
$F_\pi /F$	5.1	1	1.0760 (28)	2	1.0620 (21)	1	1.0744 (67)
$\bar{\ell }_3$	5.1	1	3.70 (27)	3	3.05 (99)	1	3.41 (41)
$\bar{\ell }_4$	5.1	1	4.67 (10)	3	4.02(28)	1	4.62 (22)
$\hat{B}_K$	6.2			4	0.766 (10)	1	0.729 (25) (17)
$B_K^{\bar{\mathrm {MS}}}$ (2 GeV)	6.2			4	0.560 (7)	1	0.533 (18) (12)
$f_D$ (MeV)	7.1			2	209.2 (3.3)	1	208 (8)
$f_{D_{s}}$ (MeV)	7.1			2	248.6 (2.7)	1	250 (7)
$f_{D_{s}}/f_D$	7.1			2	1.187 (12)	1	1.20 (2)
$f_+^{D\pi } (0)$	7.2			1	0.666 (29)		
$f_+^{DK} (0)$	7.2			1	0.747 (19)		
$f_B$ (MeV)	8.1	1	186 (4)	3	190.5 (4.2)	1	189 (8)
$f_{B_{s}}$ (MeV)	8.1	1	224 (5)	3	227.7 (4.5)	1	228 (8)
$f_{B_{s}}/f_B$	8.1	1	1.205 (7)	2	1.202 (22)	1	1.206 (24)
$f_{B_{d}}\sqrt{\hat{B}_{B_{d}}}$ (MeV)	8.2			1	216 (15)		
$f_{B_{s}}\sqrt{\hat{B}_{B_{s}}}$ (MeV)	8.2			1	266 (18)		
$\hat{B}_{B_{d}}$	8.2			1	1.27 (10)		
$\hat{B}_{B_{s}}$	8.2			1	1.33 (6)		
$\xi$	8.2			1	1.268 (63)		
$\hat{B}_{B_{s}}/\hat{B}_{B_{d}}$	8.2			1	1.06 (11)		
$\Delta \zeta ^{B\pi }$ (ps$^{-1}$)	8.3			2	2.16 (50)		
$f_+^{B\pi } (q^2): a_0^\mathrm{BCL}$	8.3			2	0.453 (33)		
$a_1^\mathrm{BCL}$				2	$-$0.43 (33)		
$a_2^\mathrm{BCL}$				2	0.9 (3.9)		
${\mathcal {F}}^{B \rightarrow D^*} (1)$	8.4			1	0.906 (4) (12)		
$R (D)$	8.4			1	0.316 (12) (7)		
$\alpha _{\overline{\mathrm{MS}}}^{ (5)} (M_Z)$	9.9			4	0.1184 (12)		

### FLAG enlargement

Upon completion of the first review, it was decided to extend the project by adding new physical quantities and co-authors. FLAG became more representative of the lattice community, both in terms of the geographical location of its members and the lattice collaborations to which they belong. At the time a parallel effort had been made [2, 3]; the two efforts have now merged in order to provide a single source of information on lattice results to the particle-physics community.

The experience gained in managing the activities of a medium-sized group of co-authors taught us that it was necessary to have a more formal structure and a set of rules by which all concerned had to abide, in order to make the inner workings of FLAG function smoothly. The collaboration presently consists of an Advisory Board (AB), an Editorial Board (EB), and seven Working Groups (WG). The rôle of the Advisory Board is that of general supervision and consultation. Its members may interfere at any point in the process of drafting the paper, expressing their opinion and offering advice. They also give their approval of the final version of the preprint before it is rendered public. The Editorial Board coordinates the activities of FLAG, sets priorities and intermediate deadlines, and takes care of the editorial work needed to amalgamate the sections written by the individual working groups into a uniform and coherent review. The working groups concentrate on writing up the review of the physical quantities for which they are responsible, which is subsequently circulated to the whole collaboration for criticisms and suggestions.

The most important internal FLAG rules are the following:

- members of the AB have a 4-year mandate (to avoid a simultaneous change of all members, some of the current members of the AB will have a shorter mandate);
- the composition of the AB reflects the main geographical areas in which lattice collaborations are active: one member comes from America, one from Asia/Oceania and one from Europe;
- the mandate of regular members is not limited in time, but we expect that a certain turnover will occur naturally;
- whenever a replacement becomes necessary this has to keep, and possibly improve, the balance in FLAG;
- in all working groups the three members must belong to three different lattice collaborations;^2^
- a paper is in general not reviewed (nor colour-coded, as described in the next section) by one of its authors;
- lattice collaborations not represented in FLAG will be asked to check whether the colour coding of their calculation is correct.

The current list of FLAG members and their Working Group assignments is:

$\bullet$ Advisory Board (AB):    S. Aoki, C. Bernard, C. Sachrajda

$\bullet$ Editorial Board (EB):    G. Colangelo, H. Leutwyler,

A. Vladikas, U. Wenger

$\bullet$ Working Groups (WG)

(each WG coordinator is listed first):

- Quark masses:    L. Lellouch, T. Blum, V. Lubicz
- $V_{us},V_{ud}$:    A. Jüttner, T. Kaneko, S. Simula
- LEC:    S. Dürr, H. Fukaya, S. Necco
- $B_K$:    H. Wittig, J. Laiho, S. Sharpe
- $f_{B_{(s)}}$, $f_{D_{(s)}}$, $B_B$:    A. El-Khadra,Y. Aoki, M. Della Morte
- $B_{(s)}$, $D$ semileptonic and radiative decays:    R. Van de Water, E. Lunghi, C. Pena, J. Shigemitsu^3^
- $\alpha _\mathrm{s}$:    R. Sommer, R. Horsley, T. Onogi

### General issues and summary of the main results

The present review aims at two distinct goals:

- offer a **description** of the work done on the lattice concerning low-energy particle physics;
- draw **conclusions** on the basis of that work, which summarise the results obtained for the various quantities of physical interest.

The core of the information about the work done on the lattice is presented in the form of tables, which not only list the various results, but also describe the quality of the data that underlie them. We consider it important that this part of the review represents a generally accepted description of the work done. For this reason, we explicitly specify the quality requirements used and provide sufficient details in the appendices so that the reader can verify the information given in the tables.

The conclusions drawn on the basis of the available lattice results, on the other hand, are the responsibility of FLAG alone. We aim at staying on the conservative side and in several cases reach conclusions which are more cautious than what a plain average of the available lattice results would give, in particular when this is dominated by a single lattice result. An additional issue occurs when only one lattice result is available for a given quantity. In such cases one does not have the same degree of confidence in results and errors as one has when there is agreement among many different calculations using different approaches. Since this degree of confidence cannot be quantified, it is not reflected in the quoted errors, but it should be kept in mind by the reader. At present, the issue of having only a single result occurs much more often in heavy-quark physics than in light-quark physics. We are confident that the heavy-quark calculations will soon reach the state that pertains in light-quark physics.

Several general issues concerning the present review are thoroughly discussed in Sect. 1.1 of our initial paper [1] and we encourage the reader to consult the relevant pages. In the remainder of the present section, we focus on a few important points.

Each discretisation has its merits but also its shortcomings. For the topics covered already in the first edition of the FLAG review, we have by now a remarkably broad data base, and for most quantities lattice calculations based on totally different discretisations are now available. This is illustrated by the dense population of the tables and figures shown in the first part of this review. Those calculations which do satisfy our quality criteria indeed lead to consistent results, confirming universality within the accuracy reached. In our opinion, the consistency between independent lattice results, obtained with different discretisations, methods and simulation parameters, is an important test of lattice QCD, and observing such consistency then also provides further evidence that systematic errors are fully under control.

In the sections dealing with heavy quarks and with $\alpha _\mathrm{s}$, the situation is not the same. Since the $b$-quark mass cannot be resolved with current lattice spacings, all lattice methods for treating $b$ quarks use effective field theory at some level. This introduces additional complications not present in the light-quark sector. An overview of the issues specific to heavy-quark quantities is given in the introduction of Sect. 8. For $B$- and $D$-meson leptonic decay constants, there already exist a good number of different independent calculations that use different heavy-quark methods, but there are only one or two independent calculations of semileptonic $B$- and $D$-meson form factors and $B$ meson mixing parameters. For $\alpha _\mathrm{s}$, most lattice methods involve a range of scales that need to be resolved and controlling the systematic error over a large range of scales is more demanding. The issues specific to determinations of the strong coupling are summarised in Sect. 9.

The lattice spacings reached in recent simulations go down to 0.05 fm or even smaller. In that region, growing autocorrelation times slow down the sampling of the configurations [4–8]. Many groups check for autocorrelations in a number of observables, including the topological charge, for which a rapid growth of the autocorrelation time is observed if the lattice spacing becomes small. In the following, we assume that the continuum limit can be reached by extrapolating the existing simulations.

Lattice simulations of QCD currently involve at most four dynamical quark flavours. Moreover, most of the data concern simulations for which the masses of the two lightest quarks are set equal. This is indicated by the notation $N_\mathrm{f}=2+1+1$ which, in this case, denotes a lattice calculation with four dynamical quark flavours and $m_{u} = m_{d} \ne m_{s} \ne m_{c}$. Note that calculations with $N_\mathrm{f}=2$ dynamical flavours often include strange valence quarks interacting with gluons, so that bound states with the quantum numbers of the kaons can be studied, albeit neglecting strange sea quark fluctuations. The quenched approximation ($N_\mathrm{f}=0$), in which the sea quarks are treated as a mean field, is no longer used in modern lattice simulations. Accordingly, we will review results obtained with $N_\mathrm{f}=2$, $N_\mathrm{f}=2+1$ and $N_\mathrm{f} = 2+1+1$, but we omit earlier results with $N_\mathrm{f}=0$. On the other hand, the dependence of the QCD coupling constant $\alpha _\mathrm{s}$ on the number of flavours is a theoretical issue of considerable interest, and we therefore include results obtained for gluodynamics in the $\alpha _\mathrm{s}$ section. We stress, however, that only results with $N_\mathrm{f} \ge 3$ are used to determine the physical value of $\alpha _\mathrm{s}$ at a high scale.

The remarkable recent progress in the precision of lattice calculations is due to improved algorithms, better computing resources and, last but not least, conceptual developments, such as improved actions which reduce lattice artefacts, actions which preserve (remnants of) chiral symmetry, understanding finite-size effects, non-perturbative renormalisation, etc. A concise characterisation of the various discretisations that underlie the results reported in the present review is given in Appendix A.1.

Lattice simulations are performed at fixed values of the bare QCD parameters (gauge coupling and quark masses) and physical quantities with mass dimensions (e.g. quark masses, decay constants...) are computed in units of the lattice spacing; i.e. they are dimensionless. Their conversion to physical units requires knowledge of the lattice spacing at the fixed values of the bare QCD parameters of the simulations. This is achieved by requiring agreement between the lattice calculation and experimental measurement of a known quantity, which “sets the scale” of a given simulation. A few details on this procedure are provided in Appendix A.2.

Several of the results covered by this review, such as quark masses, the gauge coupling, and $B$-parameters, are quantities defined in a given renormalisation scheme and scale. The schemes employed are often chosen because of their specific merits when combined with the lattice regularisation. For a brief discussion of their properties, see Appendix A.3. The conversion of the results, obtained in these so-called intermediate schemes, to more familiar regularisation schemes, such as the ${\overline{\mathrm{MS}}}$-scheme, is done with the aid of perturbation theory. It must be stressed that the renormalisation scales accessible by the simulations are subject to limitations, naturally arising in Field-Theory computations at finite UV and small non-zero IR cutoff. Typically, such scales are of the order of the UV cutoff, or $\Lambda _\mathrm{QCD}$, depending on the chosen scheme. To safely match to ${\overline{\mathrm{MS}}}$, a scheme defined in perturbation theory, Renormalisation Group (RG) running to higher scales is performed, either perturbatively, or non-perturbatively (the latter using finite-size scaling techniques).

Because of limited computing resources, lattice simulations are often performed at unphysically heavy pion masses, although results at the physical point have recently become available. Further, numerical simulations must be done at finite lattice spacing. In order to obtain physical results, lattice data are generated at a sequence of pion masses and a sequence of lattice spacings, and then extrapolated to $M_\pi \approx 135$ MeV and $a \rightarrow 0$. To control the associated systematic uncertainties, these extrapolations are guided by effective theory. For light-quark actions, the lattice-spacing dependence is described by Symanzik’s effective theory [9, 10]; for heavy quarks, this can be extended and/or supplemented by other effective theories such as Heavy-Quark Effective Theory (HQET). The pion-mass dependence can be parameterised with Chiral Perturbation Theory ($\chi$PT), which takes into account the Nambu–Goldstone nature of the lowest excitations that occur in the presence of light quarks; similarly one can use Heavy-Light Meson Chiral Perturbation Theory (HM$\chi$PT) to extrapolate quantities involving mesons composed of one heavy ($b$ or $c$) and one light quark. One can combine Symanzik’s effective theory with $\chi$PT to simultaneously extrapolate to the physical pion mass and continuum; in this case, the form of the effective theory depends on the discretisation. See Appendix A.4 for a brief description of the different variants in use and some useful references.

## Quality criteria

The essential characteristics of our approach to the problem of rating and averaging lattice quantities reported by different collaborations have been outlined in our first publication [1]. Our aim is to help the reader assess the reliability of a particular lattice result without necessarily studying the original article in depth. This is a delicate issue, which may make things appear simpler than they are. However, it safeguards against the common practice of using lattice results and drawing physics conclusions from them, without a critical assessment of the quality of the various calculations. We believe that despite the risks, it is important to provide some compact information about the quality of a calculation. However, the importance of the accompanying detailed discussion of the results presented in the bulk of the present review cannot be underestimated.

### Systematic errors and colour-coding

In Ref. [1], we identified a number of sources of systematic errors, for which a systematic improvement is possible, and assigned one of three coloured symbols to each calculation: green star, amber disc or red square. The appearance of a red tag, even in a single source of systematic error of a given lattice result, disqualified it from the global averaging. Since results with green and amber tags entered the averages, and since this policy has been retained in the present edition, we have decided to substitute the amber disc by a green unfilled circle. Thus the new colour coding is as follows:

the systematic error has been estimated in a satisfactory manner and convincingly shown to be under control;
a reasonable attempt at estimating the systematic error has been made, although this could be improved;
no or a clearly unsatisfactory attempt at estimating the systematic error has been made. We stress once more that only results without a red tag in the systematic errors are averaged in order to provide a given FLAG estimate.

The precise criteria used in determining the colour coding is unavoidably time-dependent; as lattice calculations become more accurate the standards against which they are measured become tighter. For quantities related to the light-quark sector, which have been dealt with in the first edition of the FLAG review [1], some of the quality criteria have remained the same, while others have been tightened up. We will compare them to those of Ref. [1], case-by-case, below. For the newly introduced physical quantities, related to heavy quark physics, the adoption of new criteria was necessary. This is due to the fact that, in most cases, the discretisation of the heavy quark action follows a very different approach to that of light flavours. Moreover, the two Working Groups dedicated to heavy flavours have opted for a somewhat different rating of the extrapolation of lattice results to the continuum limit. Finally, the strong coupling being in a class of its own, as far as methods for its computation are concerned, led to the introduction of dedicated rating criteria for it.

Of course any colour coding has to be treated with caution; we repeat that the criteria are subjective and evolving. Sometimes a single source of systematic error dominates the systematic uncertainty and it is more important to reduce this uncertainty than to aim for green stars for other sources of error. In spite of these caveats we hope that our attempt to introduce quality measures for lattice results will prove to be a useful guide. In addition we would like to stress that the agreement of lattice results obtained using different actions and procedures evident in many of the tables presented below provides further validation.

For a coherent assessment of the present situation, the quality of the data plays a key role, but the colour coding cannot be carried over to the figures. On the other hand, simply showing all data on equal footing would give the misleading impression that the overall consistency of the information available on the lattice is questionable. As a way out, the figures do indicate the quality in a rudimentary way:

results included in the average;

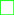
 results that are not included in the average but pass all quality criteria;

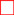
 all other results.

The reason for not including a given result in the average is not always the same: the paper may fail one of the quality criteria, may not be published, be superseded by other results or not offer a complete error budget. Symbols other than squares are used to distinguish results with specific properties and are always explained in the caption.

There are separate criteria for light-flavour, heavy-flavour, and $\alpha _\mathrm{s}$ results. In the following the criteria for the former two are discussed in detail, while the criteria for the $\alpha _\mathrm{s}$ results will be exposed separately in Sect. 9.2.

#### Light-quark physics

The colour code used in the tables is specified as follows:

$$\bullet $$ Chiral extrapolation:

$$M_{\pi ,{\mathrm {min}}}< 200$$ MeV
200 MeV $$\le M_{\pi ,{\mathrm {min}}} \le $$ 400 MeV
400 MeV $< M_{\pi ,{\mathrm {min}}}$ It is assumed that the chiral extrapolation is done with at least a three-point analysis; otherwise this will be explicitly mentioned. Note that, compared to Ref. [1], chiral extrapolations are now treated in a somewhat more stringent manner and the cutoff between green star and green open circle (formerly amber disc), previously set at 250 MeV, is now lowered to 200 MeV.

$$\bullet $$ Continuum extrapolation:

three or more lattice spacings, at least two points below 0.1 fm
two or more lattice spacings, at least one point below 0.1 fm
otherwise
  It is assumed that the action is $O(a)$-improved (i.e. the discretisation errors vanish quadratically with the lattice spacing); otherwise this will be explicitly mentioned. Moreover, for non-improved actions an additional lattice spacing is required. This criterion is the same as the one adopted in Ref. [1].

$\bullet$ Finite-volume effects:

$M_{\pi ,{\mathrm {min}}} L > 4$ or at least three volumes
$M_{\pi ,{\mathrm {min}}} L > 3$ and at least two volumes
otherwise
  These ratings apply to calculations in the $p$-regime and it is assumed that $L_\mathrm{min}\ge 2$ fm; otherwise this will be explicitly mentioned and a red square will be assigned.

$\bullet$ Renormalisation (where applicable):

non-perturbative
one-loop perturbation theory or higher with a reasonable estimate of truncation errors
otherwise
  In Ref. [1], we assigned a red square to all results which were renormalised at one loop in perturbation theory. We now feel that this is too restrictive, since the error arising from renormalisation constants, calculated in perturbation theory at one loop, is often estimated conservatively and reliably.

$\bullet$ Running (where applicable):

- For scale-dependent quantities, such as quark masses or $B_K$, it is essential that contact with continuum perturbation theory can be established. Various different methods are used for this purpose (cf. Appendix A.3): Regularisation-independent Momentum Subtraction (RI/MOM), Schrödinger functional, direct comparison with (resummed) perturbation theory. Irrespective of the particular method used, the uncertainty associated with the choice of intermediate renormalisation scales in the construction of physical observables must be brought under control. This is best achieved by performing comparisons between non-perturbative and perturbative running over a reasonably broad range of scales. These comparisons were initially only made in the Schrödinger functional (SF) approach, but they are now also being performed in RI/MOM schemes. We mark the data for which information about non-perturbative running checks is available and give some details, but we do not attempt to translate this into a colour-code.

The pion mass plays an important rôle in the criteria relevant for chiral extrapolation and finite volume. For some of the regularisations used, however, it is not a trivial matter to identify this mass. In the case of twisted-mass fermions, discretisation effects give rise to a mass difference between charged and neutral pions even when the up- and down-quark masses are equal, with the charged pion being the heavier of the two. The discussion of the twisted-mass results presented in the following sections assumes that the artificial isospin-breaking effects which occur in this regularisation are under control. In addition, we assume that the mass of the charged pion may be used when evaluating the chiral-extrapolation and finite-volume criteria. In the case of staggered fermions, discretisation effects give rise to several light states with the quantum numbers of the pion.^4^ The mass splitting among these “taste” partners represents a discretisation effect of ${\mathcal {O}}(a^2)$, which can be significant at big lattice spacings but shrinks as the spacing is reduced. In the discussion of the results obtained with staggered quarks given in the following sections, we assume that these artefacts are under control. When evaluating the chiral-extrapolation criteria, we conservatively identify $M_{\pi ,\mathrm{min}}$ with the root-mean square (RMS) of the mass of all taste partners. These masses are also used in Sects. 4 and 6 when evaluating the finite-volume criteria, while in Sects. 3, 5, 7 and 8, a more stringent finite-volume criterion is applied: $M_{\pi ,\mathrm{min}}$ is identified with the mass of the lightest state.

#### Heavy-quark physics

This subsection discusses the criteria adopted for the heavy-quark quantities included in this review, characterised by non-zero charm and bottom quantum numbers. There are several different approaches to treating heavy quarks on the lattice, each with their own issues and considerations. In general all $b$-quark methods rely on the use of Effective Field Theory (EFT) at some point in the computation, either via direct simulation of the EFT, use of the EFT to estimate the size of cutoff errors, or use of the EFT to extrapolate from the simulated lattice quark mass up to the physical $b$-quark mass. Some simulations of charm-quark quantities use the same heavy-quark methods as for bottom quarks, but there are also computations that use improved light-quark actions to simulate charm quarks. Hence, with some methods and for some quantities, truncation effects must be considered together with discretisation errors. With other methods, discretisation errors are more severe for heavy-quark quantities than for the corresponding light-quark quantities.

In order to address these complications, we add a new heavy-quark treatment category to the ratings system. The purpose of this criterion is to provide a guideline for the level of action and operator improvement needed in each approach to make reliable calculations possible, in principle. In addition, we replace the rating criteria for the continuum extrapolations of Sect. 2.1.1 with a new empirical approach based on the size of observed discretisation errors in the lattice simulation data. This accounts for the fact that whether discretisation and truncation effects in a given calculation are sufficiently small as to be controllable depends not only on the range of lattice spacings used in the simulations, but also on the simulated heavy-quark masses and on the level of action and operator improvement. For the other categories, we adopt the same strict criteria as in Sect. 2.1.1, with one minor modification, as explained below.

$\bullet$ Heavy-quark treatment

- A description of the different approaches to treating heavy quarks on the lattice is given in Appendix A.1.3 including a discussion of the associated discretisation, truncation, and matching errors. For truncation errors we use HQET power counting throughout, since this review is focussed on heavy quark quantities involving $B$ and $D$ mesons. Here we describe the criteria for how each approach must be implemented in order to receive an acceptable () rating for both the heavy quark actions and the weak operators. Heavy-quark implementations without the level of improvement described below are rated not acceptable (). The matching is evaluated together with renormalisation, using the renormalisation criteria described in Sect. 2.1.1. We emphasise that the heavy-quark implementations rated as acceptable and described below have been validated in a variety of ways, such as via phenomenological agreement with experimental measurements, consistency between independent lattice calculations, and numerical studies of truncation errors. These tests are summarised in Sect. 8.

*Relativistic heavy quark actions:*

at least tree-level $O(a)$ improved action and weak operators
  This is similar to the requirements for light quark actions. All current implementations of relativistic heavy quark actions satisfy these criteria.
*NRQCD:*
tree-level matched through $O(1/m_{h})$ and improved through $O(a^2)$
  The current implementations of NRQCD satisfy these criteria, and also include tree-level corrections of $O(1/m_{h}^2)$ in the action.
*HQET:*
tree-level matched through $O(1/m_{h})$ with discretisation errors starting at $O(a^2)$
  The current implementation of HQET by the ALPHA collaboration satisfies these criteria with an action and weak operators that are non-perturbatively matched through $O(1/m_{h})$. Calculations that exclusively use a static-limit action do not satisfy theses criteria, since the static-limit action, by definition, does not include $1/m_{h}$ terms. However, for SU(3)-breaking ratios such as $\xi$ and $f_{B_{s}}/f_B$ truncation errors start at $O((m_{s} - m_{d})/m_{h})$. We therefore consider lattice calculations of such ratios that use a static-limit action to still have controllable truncation errors.
*Light-quark actions for heavy quarks:*
discretisation errors starting at $O(a^2)$ or higher This applies to calculations that use the tmWilson action, a non-perturbatively improved Wilson action, or the HISQ action for charm quark quantities. It also applies to calculations that use these light quark actions in the charm region and above together with either the static limit or with an HQET-inspired extrapolation to obtain results at the physical $b$ quark mass. In these cases, the continuum-extrapolation criteria must be applied to the entire range of heavy quark masses used in the calculation.

$$\bullet $$ Continuum extrapolation:

- First we introduce the following definitions:
  1 $$\begin{aligned} D(a) = \frac{Q(a) - Q(0)}{Q(a)}, \end{aligned}$$
  where $Q(a)$ denotes the central value of quantity $Q$ obtained at lattice spacing $a$ and $Q(0)$ denotes the continuum extrapolated value. $D(a)$ is a measure of how far the continuum extrapolated result is from the lattice data. We evaluate this quantity on the smallest lattice spacing used in the calculation, $a_\mathrm{min}$.
  2 $$\begin{aligned} \delta (a) = \frac{Q(a) - Q(0)}{\sigma _Q}, \end{aligned}$$
  where $\sigma _Q$ is the combined statistical and systematic (due to the continuum extrapolation) error. $\delta (a)$ is a measure of how well the continuum-extrapolated result agrees with the lattice data within the statistical and systematic errors of the calculation. Again, we evaluate this quantity on the smallest lattice spacing used in the calculation, $a_\mathrm{min}$.

(i) Three or more lattice spacings,
  (ii) $$a^2_\mathrm{max} / a^2_\mathrm{min} \ge 2$$,
  (iii) $$D(a_\mathrm{min}) \le 2\,\%$$, and
  (iv) $$\delta (a_\mathrm{min}) \le 1$$
(i) Two or more lattice spacings,
  (ii) $$a^2_\mathrm{max} / a^2_\mathrm{min} \ge 1.4$$,
  (iii) $$D(a_\mathrm{min}) \le 10\,\%$$,
  (iv) $$\delta (a_\mathrm{min}) \le 2$$,
otherwise.
  For the time being, these new criteria for the quality of the continuum extrapolation have only been adopted for the heavy-quark quantities, but their use may be extended to all FLAG quantities in future reviews.

$$\bullet $$ Finite-volume:

$M_{\pi ,\mathrm{min}} L \gtrsim 3.7$ or two volumes at fixed parameters
$$M_{\pi ,\mathrm{min}} L \gtrsim 3$$
otherwise

Here the boundary between green star and open circle is slightly relaxed compared to that in Sect. 2.1.1 to account for the fact that heavy-quark quantities are less sensitive to this systematic error than light-quark quantities. A  rating requires an estimate of the finite-volume error either by analysing data on two or more physical volumes (with all other parameters fixed) or by using finite-volume chiral perturbation theory. In the case of staggered sea quarks, $M_{\pi ,\mathrm{min}}$ refers to the lightest (taste Goldstone) pion mass.

### Averages and estimates

For many observables there are enough independent lattice calculations of good quality that it makes sense to average them and propose such an *average* as the best current lattice number. In order to decide whether this is true for a certain observable, we rely on the colour coding. We restrict the averages to data for which the colour code does not contain any red tags. In some cases, the averaging procedure nevertheless leads to a result which in our opinion does not cover all uncertainties. This is related to the fact that procedures for estimating errors and the resulting conclusions necessarily have an element of subjectivity, and would vary between groups even with the same data set. In order to stay on the conservative side, we may replace the average by an *estimate* (or a *range*), which we consider as a fair assessment of the knowledge acquired on the lattice at present. This estimate is not obtained with a prescribed mathematical procedure, but it is based on a critical analysis of the available information.

There are two other important criteria which also play a role in this respect, but which cannot be colour coded, because a systematic improvement is not possible. These are: (i) the publication status, and (ii) the number of flavours $N_\mathrm{f}$. As far as the former criterion is concerned, we adopt the following policy: we average only results which have been published in peer-reviewed journals, i.e. they have been endorsed by referee(s). The only exception to this rule consists in obvious updates of previously published results, typically presented in conference proceedings. Such updates, which supersede the corresponding results in the published papers, are included in the averages. Nevertheless, all results are listed and their publication status is identified by the following symbols:

$$\bullet $$ Publication status:

- A published or plain update of published results
- P preprint
- C conference contribution

Note that updates of earlier results rely, at least partially, on the same gauge-field configuration ensembles. For this reason, we do not average updates with earlier results. In the present edition, the publication status on November 30, 2013 is relevant. If the paper appeared in print after that date this is accounted for in the bibliography, but it does not affect the averages.

In this review we present results from simulations with $N_\mathrm{f}=2$, $N_\mathrm{f}=2+1$ and $N_\mathrm{f}=2+1+1$ (for $r_0 \Lambda _{\overline{\mathrm{MS}}}$ also with $N_\mathrm{f}=0$). We are not aware of an *a priori* way to quantitatively estimate the difference between results produced in simulations with a different number of dynamical quarks. We therefore average results at fixed $N_\mathrm{f}$ separately; averages of calculations with different $N_\mathrm{f}$ will not be provided.

To date, no significant differences between results with different values of $N_\mathrm{f}$ have been observed. In the future, as the accuracy and the control over systematic effects in lattice calculations will increase, it will hopefully be possible to see a difference between $N_\mathrm{f}= 2$ and $N_\mathrm{f}= 2 + 1$ calculations and so determine the size of the Zweig-rule violations related to strange quark loops. This is a very interesting issue per se, and one which can be quantitatively addressed only with lattice calculations.

### Averaging procedure and error analysis

In [1], the FLAG averages and their errors were estimated through the following procedure: Having added in quadrature statistical and systematic errors for each individual result, we obtained their weighted $\chi ^2$ average. This was our central value. If the fit was of good quality ($\chi _\mathrm{min}^2/\hbox {dof} \le 1$), we calculated the net uncertainty $\delta$ from $\chi ^2 = \chi _\mathrm{min}^2 + 1$; otherwise, we inflated the result obtained in this way by the factor $S = \sqrt{(}\chi ^2/\hbox {dof})$. Whenever this $\chi ^2$ minimisation procedure resulted in a total error which was smaller than the smallest systematic error of any individual lattice result, we assigned the smallest systematic error of that result to the total systematic error in the average.

One of the problems arising when forming such averages is that not all of the data sets are independent; in fact, some rely on the same ensembles. In particular, the same gauge-field configurations, produced with a given fermion discretisation, are often used by different research teams with different valence quark lattice actions, obtaining results which are not really independent. In the present paper we have modified our averaging procedure, in order to account for such correlations. To start with, we examine error budgets for individual calculations and look for potentially correlated uncertainties. Specific problems encountered in connection with correlations between different data sets are commented in the text. If there is any reason to believe that a source of error is correlated between two calculations, a 100 % correlation is assumed. We then obtain the central value from a $\chi ^2$ weighted average, evaluated by adding statistical and systematic errors in quadrature (just as in Ref. [1]): for a set of individual measurements $x_i$ with error $\sigma _i$ and correlation matrix $C_{ij}$, central value and error of the average are given by

3 $$\begin{aligned} x_\mathrm{average}&= \sum _i x_i\, \omega _i, \quad \omega _i = \dfrac{\sigma _i^{-2}}{\sum _j\sigma _j^{-2}},\end{aligned}$$

4 $$\begin{aligned} \sigma ^2_\mathrm{average}&= \sum _{i,j} \omega _i \,\omega _j \,C_{ij}. \end{aligned}$$

The correlation matrix for the set of correlated lattice results is estimated with Schmelling’s prescription [16]. When necessary, the statistical and systematic error bars are stretched by a factor $S$, as specified in the previous paragraph.

## Masses of the light quarks

Quark masses are fundamental parameters of the Standard Model. An accurate determination of these parameters is important for both phenomenological and theoretical applications. The charm and bottom masses, for instance, enter the theoretical expressions of several cross sections and decay rates in heavy-quark expansions. The up-, down- and strange-quark masses govern the amount of explicit chiral symmetry breaking in QCD. From a theoretical point of view, the values of quark masses provide information about the flavour structure of physics beyond the Standard Model. The Review of Particle Physics of the Particle Data Group contains a review of quark masses [17], which covers light as well as heavy flavours. The present summary only deals with the light-quark masses (those of the up, down and strange quarks), but it discusses the lattice results for these in more detail.

Quark masses cannot be measured directly with experiment because quarks cannot be isolated, as they are confined inside hadrons. On the other hand, quark masses are free parameters of the theory and, as such, cannot be obtained on the basis of purely theoretical considerations. Their values can only be determined by comparing the theoretical prediction for an observable, which depends on the quark mass of interest, with the corresponding experimental value. What makes light-quark masses particularly difficult to determine is the fact that they are very small (for the up and down) or small (for the strange) compared to typical hadronic scales. Thus, their impact on typical hadronic observables is minute and it is difficult to isolate their contribution accurately.

Fortunately, the spontaneous breaking of SU(3)$_L\otimes$SU(3)$_R$ chiral symmetry provides observables which are particularly sensitive to the light-quark masses: the masses of the resulting Nambu–Goldstone bosons (NGB), i.e. pions, kaons and etas. Indeed, the Gell-Mann–Oakes–Renner relation [18] predicts that the squared mass of a NGB is directly proportional to the sum of the masses of the quark and antiquark which compose it, up to higher-order mass corrections. Moreover, because these NGBs are light and are composed of only two valence particles, their masses have a particularly clean statistical signal in lattice-QCD calculations. In addition, the experimental uncertainties on these meson masses are negligible.

Three flavour QCD has four free parameters: the strong coupling, $\alpha _\mathrm{s}$ (alternatively $\Lambda _\mathrm{QCD}$) and the up, down and strange quark masses, $m_{u}$, $m_{d}$ and $m_{s}$. However, present day lattice calculations are often performed in the isospin limit, and the up and down quark masses (especially those in the sea) usually get replaced by a single parameter: the isospin-averaged up- and down-quark mass, $m_{ud}=\frac{1}{2}(m_{u}+m_{d})$. A lattice determination of these parameters requires two steps:

1. Calculations of three experimentally measurable quantities are used to fix the three bare parameters. As already discussed, NGB masses are particularly appropriate for fixing the light-quark masses. Another observable, such as the mass of a member of the baryon octet, can be used to fix the overall scale. It is important to note that until recently, most calculations were performed at values of $m_{ud}$ which were still substantially larger than its physical value, typically four times as large. Reaching the physical up- and down-quark mass point required a significant extrapolation. This situation is changing fast. The PACS-CS [19–21] and BMW [22, 23] calculations were performed with masses all the way down to their physical value (and even below in the case of BMW), albeit in very small volumes for PACS-CS. More recently, MILC [24] and RBC/UKQCD [25] have also extended their simulations almost down to the physical point, by considering pions with $M_\pi \gtrsim 170\,\mathrm{MeV}$.^5^ Regarding the strange quark, modern simulations can easily include them with masses that bracket its physical value, and only interpolations are needed.
2. Renormalisations of these bare parameters must be performed to relate them to the corresponding cutoff-independent, renormalised parameters.^6^ These are short-distance calculations, which may be performed perturbatively. Experience shows that one-loop calculations are unreliable for the renormalisation of quark masses: usually at least two loops are required to have trustworthy results. Therefore, it is best to perform the renormalisations non-perturbatively to avoid potentially large perturbative uncertainties due to neglected higher-order terms. However, we will include in our averages one-loop results which carry a solid estimate of the systematic uncertainty due to the truncation of the series.

Of course, in quark mass ratios the renormalisation factor cancels, so that this second step is no longer relevant.

### Contributions from the electromagnetic interaction

As mentioned in Sect. 2.1, the present review relies on the hypothesis that, at low energies, the Lagrangian $\mathcal{L}_{\mathrm{QCD}}+\mathcal{L}_{\mathrm{QED}}$ describes nature to a high degree of precision. Moreover, we assume that, at the accuracy reached by now and for the quantities discussed here, the difference between the results obtained from simulations with three dynamical flavours and full QCD is small in comparison with the quoted systematic uncertainties. This will soon no longer be the case. The electromagnetic (e.m.) interaction, on the other hand, cannot be ignored. Quite generally, when comparing QCD calculations with experiment, radiative corrections need to be applied. In lattice simulations, where the QCD parameters are fixed in terms of the masses of some of the hadrons, the electromagnetic contributions to these masses must be accounted for.^7^

The electromagnetic interaction plays a crucial role in determinations of the ratio $m_{u}/m_{d}$, because the isospin-breaking effects generated by this interaction are comparable to those from $m_{u}\ne m_{d}$ (see Sect. 3.4). In determinations of the ratio $m_{s}/m_{ud}$, the electromagnetic interaction is less important, but at the accuracy reached, it cannot be neglected. The reason is that, in the determination of this ratio, the pion mass enters as an input parameter. Because $M_\pi$ represents a small symmetry-breaking effect, it is rather sensitive to the perturbations generated by QED.

We distinguish the physical mass $M_P$, $P\in \{\pi ^+, \pi ^0$, $K^+$, $K^0\}$, from the mass $\hat{M}_P$ within QCD alone. The e.m. self-energy is the difference between the two, $M_P^\gamma \equiv M_P-\hat{M}_P$. Because the self-energy of the Nambu–Goldstone bosons diverges in the chiral limit, it is convenient to replace it by the contribution of the e.m. interaction to the *square* of the mass,

5 $$\begin{aligned} \Delta _{P}^\gamma \equiv M_P^2-\hat{M}_P^2= 2\,M_P M_P^\gamma +O(e^4). \end{aligned}$$

The main effect of the e.m. interaction is an increase in the mass of the charged particles, generated by the photon cloud that surrounds them. The self-energies of the neutral ones are comparatively small, particularly for the Nambu–Goldstone bosons, which do not have a magnetic moment. Dashen’s theorem [31] confirms this picture, as it states that, to leading order (LO) of the chiral expansion, the self-energies of the neutral NGBs vanish, while the charged ones obey $\Delta _{K^+}^\gamma = \Delta _{\pi ^+}^\gamma$. It is convenient to express the self-energies of the neutral particles as well as the mass difference between the charged and neutral pions within QCD in units of the observed mass difference, $\Delta _\pi \equiv M_{\pi ^+}^2-M_{\pi ^0}^2$:

6 $$\begin{aligned} \Delta _{\pi ^0}^\gamma \equiv \epsilon _{\pi ^0}\,\Delta _\pi ,\Delta _{K^0}^\gamma \!\equiv \! \epsilon _{K^0}\,\Delta _\pi ,\hat{M}_{\pi ^+}^2\!-\! \hat{M}_{\pi ^0}^2\equiv \epsilon _{m}\,\Delta _\pi .\end{aligned}$$

In this notation, the self-energies of the charged particles are given by

7 $$\begin{aligned}&\Delta _{\pi ^+}^\gamma =(1+\epsilon _{\pi ^0}-\epsilon _{m})\,\Delta _\pi ,\nonumber \\&\quad \Delta _{K^+}^\gamma =(1+\epsilon +\epsilon _{K^0}-\epsilon _{m})\,\Delta _\pi ,\end{aligned}$$

where the dimensionless coefficient $\epsilon$ parameterises the violation of Dashen’s theorem,^8^

8 $$\begin{aligned} \Delta _{K^+}^\gamma -\Delta _{K^0}^\gamma - \Delta _{\pi ^+}^\gamma +\Delta _{\pi ^0}^\gamma \equiv \epsilon \,\Delta _\pi .\end{aligned}$$

Any determination of the light-quark masses based on a calculation of the masses of $\pi ^+,K^+$ and $K^0$ within QCD requires an estimate for the coefficients $\epsilon$, $\epsilon _{\pi ^0}$, $\epsilon _{K^0}$ and $\epsilon _{m}$.

The first determination of the self-energies on the lattice was carried out by Duncan et al. [33]. Using the quenched approximation, they arrived at $M_{K^+}^\gamma -M_{K^0}^\gamma = 1.9\,\hbox {MeV}$. Actually, the parameterisation of the masses given in that paper yields an estimate for all but one of the coefficients introduced above (since the mass splitting between the charged and neutral pions in QCD is neglected, the parameterisation amounts to setting $\epsilon _{m}=0$ ab initio). Evaluating the differences between the masses obtained at the physical value of the electromagnetic coupling constant and at $e=0$, we obtain $\epsilon = 0.50(8)$, $\epsilon _{\pi ^0} = 0.034(5)$ and $\epsilon _{K^0} = 0.23(3)$. The errors quoted are statistical only: an estimate of lattice systematic errors is not possible from the limited results of Duncan et al. [33]. The result for $\epsilon$ indicates that the violation of Dashen’s theorem is sizeable: according to this calculation, the non-leading contributions to the self-energy difference of the kaons amount to 50 % of the leading term. The result for the self-energy of the neutral pion cannot be taken at face value, because it is small, comparable to the neglected mass difference $\hat{M}_{\pi ^+}-\hat{M}_{\pi ^0}$. To illustrate this, we note that the numbers quoted above are obtained by matching the parameterisation with the physical masses for $\pi ^0$, $K^+$ and $K^0$. This gives a mass for the charged pion that is too high by 0.32 MeV. Tuning the parameters instead such that $M_{\pi ^+}$ comes out correctly, the result for the self-energy of the neutral pion becomes larger: $\epsilon _{\pi ^0}=0.10(7)$ where, again, the error is statistical only.

In an update of this calculation by the RBC collaboration [34] (RBC 07), the electromagnetic interaction is still treated in the quenched approximation, but the strong interaction is simulated with $N_\mathrm{f}=2$ dynamical quark flavours. The quark masses are fixed with the physical masses of $\pi ^0$, $K^+$ and $K^0$. The outcome for the difference in the electromagnetic self-energy of the kaons reads $M_{K^+}^\gamma -M_{K^0}^\gamma = 1.443(55)\,\hbox {MeV}$. This corresponds to a remarkably small violation of Dashen’s theorem. Indeed, a recent extension of this work to $N_\mathrm{f}=2+1$ dynamical flavours [32] leads to a significantly larger self-energy difference: $M_{K^+}^\gamma -M_{K^0}^\gamma = 1.87(10)\,\hbox {MeV}$, in good agreement with the estimate of Eichten et al. Expressed in terms of the coefficient $\epsilon$ that measures the size of the violation of Dashen’s theorem, it corresponds to $\epsilon =0.5(1)$.

The input for the electromagnetic corrections used by MILC is specified in [35]. In their analysis of the lattice data, $\epsilon _{\pi ^0}$, $\epsilon _{K^0}$ and $\epsilon _{m}$ are set equal to zero. For the remaining coefficient, which plays a crucial role in determinations of the ratio $m_{u}/m_{d}$, the very conservative range $\epsilon =1\pm 1$ was used in MILC 04 [36], while in more recent work, in particular in MILC 09 [15] and MILC 09A [37], this input is replaced by $\epsilon =1.2\pm 0.5$, as suggested by phenomenological estimates for the corrections to Dashen’s theorem [38, 39]. Results of an evaluation of the electromagnetic self-energies based on $N_\mathrm{f}=2+1$ dynamical quarks in the QCD sector and on the quenched approximation in the QED sector are also reported by MILC [40–42]. Their preliminary result is $\bar{\epsilon }=0.65(7)(14)(10)$, where the first error is statistical, the second systematic, and the third a separate systematic for the combined chiral and continuum extrapolation. The estimate of the systematic error does not yet include finite-volume effects. With the estimate for $\epsilon _{m}$ given in (9), this result corresponds to $\epsilon = 0.62(7)(14)(10)$. Similar preliminary results were previously reported by the BMW collaboration in conference proceedings [43, 44].

The RM123 collaboration employs a new technique to compute e.m. shifts in hadron masses in two-flavour QCD: the effects are included at leading order in the electromagnetic coupling $\alpha$ through simple insertions of the fundamental electromagnetic interaction in quark lines of relevant Feynman graphs [45]. They find $\epsilon =0.79(18)(18)$ where the first error is statistical and the second is the total systematic error resulting from chiral, finite-volume, discretisation, quenching and fitting errors all added in quadrature.

The effective Lagrangian that governs the self-energies to next-to-leading order (NLO) of the chiral expansion was set up in [46]. The estimates in [38, 39] are obtained by replacing QCD with a model, matching this model with the effective theory and assuming that the effective coupling constants obtained in this way represent a decent approximation to those of QCD. For alternative model estimates and a detailed discussion of the problems encountered in models based on saturation by resonances, see [47–49]. In the present review of the information obtained on the lattice, we avoid the use of models altogether.

There is an indirect phenomenological determination of $\epsilon$, which is based on the decay $\eta \rightarrow 3\pi$ and does not rely on models. The result for the quark mass ratio $Q$, defined in (24) and obtained from a dispersive analysis of this decay, implies $\epsilon = 0.70(28)$ (see Sect. 3.4). While the values found in older lattice calculations [32–34] are a little less than one standard deviation lower, the most recent determinations [40–45, 50], though still preliminary, are in excellent agreement with this result and have significantly smaller error bars. However, even in the more recent calculations, e.m. effects are treated in the quenched approximation. Thus, we choose to quote $\epsilon = 0.7(3)$, which is essentially the $\eta \rightarrow 3\pi$ result and covers generously the range of post 2010 lattice results. Note that this value has an uncertainty which is reduced by about 40 % compared to the result quoted in the first edition of the FLAG review [1].

We add a few comments concerning the physics of the self-energies and then specify the estimates used as an input in our analysis of the data. The Cottingham formula [51] represents the self-energy of a particle as an integral over electron scattering cross sections; elastic as well as inelastic reactions contribute. For the charged pion, the term due to elastic scattering, which involves the square of the e.m. form factor, makes a substantial contribution. In the case of the $\pi ^0$, this term is absent, because the form factor vanishes on account of charge conjugation invariance. Indeed, the contribution from the form factor to the self-energy of the $\pi ^+$ roughly reproduces the observed mass difference between the two particles. Furthermore, the numbers given in [52–54] indicate that the inelastic contributions are significantly smaller than the elastic contributions to the self-energy of the $\pi ^+$. The low energy theorem of Das et al. [55] ensures that, in the limit $m_{u},m_{d}\rightarrow 0$, the e.m. self-energy of the $\pi ^0$ vanishes, while the one of the $\pi ^+$ is given by an integral over the difference between the vector and axial-vector spectral functions. The estimates for $\epsilon _{\pi ^0}$ obtained in [33] are consistent with the suppression of the self-energy of the $\pi ^0$ implied by chiral SU(2) $\times$ SU(2). In our opinion, $\epsilon _{\pi ^0}=0.07(7)$ is a conservative estimate for this coefficient. The self-energy of the $K^0$ is suppressed less strongly, because it remains different from zero if $m_{u}$ and $m_{d}$ are taken massless and only disappears if $m_{s}$ is turned off as well. Note also that, since the e.m. form factor of the $K^0$ is different from zero, the self-energy of the $K^0$ does pick up an elastic contribution. The lattice result for $\epsilon _{K^0}$ indicates that the violation of Dashen’s theorem is smaller than in the case of $\epsilon$. In the following, we use $\epsilon _{K^0}=0.3(3)$.

Finally, we consider the mass splitting between the charged and neutral pions in QCD. This effect is known to be very small, because it is of second order in $m_{u}-m_{d}$. There is a parameter-free prediction, which expresses the difference $\hat{M}_{\pi ^+}^2-\hat{M}_{\pi ^0}^2$ in terms of the physical masses of the pseudoscalar octet and is valid to NLO of the chiral perturbation series. Numerically, the relation yields $\epsilon _{m}=0.04$ [56], indicating that this contribution does not play a significant role at the present level of accuracy. We attach a conservative error also to this coefficient: $\epsilon _{m}=0.04(2)$. The lattice result for the self-energy difference of the pions, reported in [32], $M_{\pi ^+}^\gamma -M_{\pi ^0}^\gamma = 4.50(23)\,\hbox {MeV}$, agrees with this estimate: expressed in terms of the coefficient $\epsilon _{m}$ that measures the pion mass splitting in QCD, the result corresponds to $\epsilon _{m}=0.04(5)$. The corrections of next-to-next-to-leading order (NNLO) have been worked out [57], but the numerical evaluation of the formulae again meets with the problem that the relevant effective coupling constants are not reliably known.

In summary, we use the following estimates for the e.m. corrections:

9 $$\begin{aligned} \epsilon ={ 0.7(3)} ,\ \epsilon _{\pi ^0}=0.07(7),\ \epsilon _{K^0}=0.3(3),\ \epsilon _{m}=0.04(2).\nonumber \\ \end{aligned}$$

While the range used for the coefficient $\epsilon$ affects our analysis in a significant way, the numerical values of the other coefficients only serve to set the scale of these contributions. The range given for $\epsilon _{\pi ^0}$ and $\epsilon _{K^0}$ may be overly generous, but because of the exploratory nature of the lattice determinations, we consider it advisable to use a conservative estimate.

Treating the uncertainties in the four coefficients as statistically independent and adding errors in quadrature, the numbers in equation (9) yield the following estimates for the e.m. self-energies,

10 $$\begin{aligned}&M_{\pi ^+}^\gamma = 4.7(3)\, \hbox {MeV},\ M_{\pi ^0}^\gamma = 0.3(3)\,\hbox {MeV} ,\nonumber \\&\quad M_{\pi ^+}^\gamma -M_{\pi ^0}^\gamma =4.4(1)\, \hbox {MeV},\nonumber \\&M_{K^+}^\gamma = 2.5(5)\,\hbox {MeV},\ M_{K^0}^\gamma =0.4(4)\,\hbox {MeV},\nonumber \\&\quad M_{K^+}^\gamma -M_{K^0}^\gamma = 2.1(4)\, \hbox {MeV}, \end{aligned}$$

and for the pion and kaon masses occurring in the QCD sector of the Standard Model,

11 $$\begin{aligned}&\hat{M}_{\pi ^+}= 134.8(3)\, \hbox {MeV},\ \hat{M}_{\pi ^0} = 134.6(3)\,\hbox {MeV} ,\nonumber \\&\quad \hat{M}_{\pi ^+} -\hat{M}_{\pi ^0}=0.2(1)\, \hbox {MeV},\nonumber \\&\hat{M}_{K^+}= 491.2(5)\,\hbox {MeV},\ \hat{M}_{K^0} =497.2(4)\,\hbox {MeV},\nonumber \\&\quad \hat{M}_{K^+}-\hat{M}_{K^0}=-6.1(4)\, \hbox {MeV}.\end{aligned}$$

The self-energy difference between the charged and neutral pion involves the same coefficient $\epsilon _{m}$ that describes the mass difference in QCD—this is why the estimate for $M_{\pi ^+}^\gamma -M_{\pi ^0}^\gamma$ is so sharp.

### Pion and kaon masses in the isospin limit

As mentioned above, most of the lattice calculations concerning the properties of the light mesons are performed in the isospin limit of QCD ($m_{u}-m_{d}\rightarrow 0$ at fixed $m_{u}+m_{d}$). We denote the pion and kaon masses in that limit by $\overline{M}_{\pi }$ and $\overline{M}_{K}$, respectively. Their numerical values can be estimated as follows. Since the operation $u\leftrightarrow d$ interchanges $\pi ^+$ with $\pi ^-$ and $K^+$ with $K^0$, the expansion of the quantities $\hat{M}_{\pi ^+}^2$ and $\frac{1}{2}(\hat{M}_{K^+}^2+\hat{M}_{K^0}^2)$ in powers of $m_{u}-m_{d}$ only contains even powers. As shown in [58], the effects generated by $m_{u}-m_{d}$ in the mass of the charged pion are strongly suppressed: the difference $\hat{M}_{\pi ^+}^2-\overline{M}_{\pi }^{\,2}$ represents a quantity of $O[(m_{u}-m_{d})^2(m_{u}+m_{d})]$ and is therefore small compared to the difference $\hat{M}_{\pi ^+}^2-\hat{M}_{\pi ^0}^2$, for which an estimate was given above. In the case of $\frac{1}{2}(\hat{M}_{K^+}^2+\hat{M}_{K^0}^2)-\overline{M}_{K}^{\,2}$, the expansion does contain a contribution at NLO, determined by the combination $2L_8-L_5$ of low-energy constants, but the lattice results for that combination show that this contribution is very small, too. Numerically, the effects generated by $m_{u}-m_{d}$ in $\hat{M}_{\pi ^+}^2$ and in $\frac{1}{2}(\hat{M}_{K^+}^2+\hat{M}_{K^0}^2)$ are negligible compared to the uncertainties in the electromagnetic self-energies. The estimates for these given in Eq. (11) thus imply

12 $$\begin{aligned}&\overline{M}_{\pi }= \hat{M}_{\pi ^+}=134.8(3)\,\mathrm{MeV},\nonumber \\&\overline{M}_{K}= \sqrt{\frac{1}{2}\left( \hat{M}_{K^+}^2+\hat{M}_{K^0}^2\right) }= 494.2(4)\,\mathrm{MeV}. \end{aligned}$$

This shows that, for the convention used above to specify the QCD sector of the Standard Model, and within the accuracy to which this convention can currently be implemented, the mass of the pion in the isospin limit agrees with the physical mass of the neutral pion: $\overline{M}_{\pi }-M_{\pi 0}=-0.2(3)$ MeV.

### Lattice determination of $m_{s}$ and $m_{ud}$

We now turn to a review of the lattice calculations of the light-quark masses and begin with $m_{s}$, the isospin-averaged up- and down-quark mass, $m_{ud}$, and their ratio. Most groups quote only $m_{ud}$, not the individual up- and down-quark masses. We then discuss the ratio $m_{u}/m_{d}$ and the individual determination of $m_{u}$ and $m_{d}$.

Quark masses have been calculated on the lattice since the mid-1990s. However, early calculations were performed in the quenched approximation, leading to unquantifiable systematics. Thus in the following, we only review modern, unquenched calculations, which include the effects of light sea-quarks.

Tables 2 and 3 list the results of $N_\mathrm{f}=2$ and $N_\mathrm{f}=2+1$ lattice calculations of $m_{s}$ and $m_{ud}$. These results are given in the ${\overline{\mathrm{MS}}}$ scheme at $2\,\mathrm{GeV}$, which is standard nowadays, though some groups are starting to quote results at higher scales (e.g. [25]). The tables also show the colour-coding of the calculations leading to these results. The corresponding results for $m_{s}/m_{ud}$ are given in Table 4. As indicated earlier in this review, we treat $N_\mathrm{f}=2$ and $N_\mathrm{f}=2+1$ calculations separately. The latter include the effects of a strange sea-quark, but the former do not.

**Table 2:** $N_\mathrm{f}=2$ lattice results for the masses $m_{ud}$ and $m_{s}$ (MeV, running masses in the ${\overline{\mathrm{MS}}}$ scheme at scale 2 GeV). The significance of the colours is explained in Sect. 2. If information about non-perturbative running is available, this is indicated in the column “running”, with details given at the bottom of the table

Collaboration	Ref.	Publication status	Chiral extrapolation	Continuum extrapolation	Finite volume	Renormalisation	Running	$m_{ud}$	$m_{s}$
ALPHA 12	[59]	A					$^\mathrm{a,b}$		102 (3) (1)
Dürr 11$^\ddagger$	[61]	A				–	–	3.52 (10) (9)	97.0 (2.6) (2.5)
ETM 10B	[60]	A					$^\mathrm{c}$	3.6 (1) (2)	95 (2) (6)
JLQCD/ TWQCD 08A	[67]	A					–	4.452 (81) (38) $(^{+0}_{227})$	–
RBC 07$^\dagger$	[34]	A					–	4.25 (23) (26)	119.5 (5.6) (7.4)
ETM 07	[62]	A					–	3.85 (12) (40)	105 (3) (9)
QCDSF/ UKQCD 06	[68]	A					–	4.08 (23) (19) (23)	111 (6) (4) (6)
SPQcdR 05	[69]	A					–	4.3 (4) ($$^{+1.1}_{-0.0})$$	101 (8) $$(^{+25}_{-0})$$
ALPHA 05	[64]	A					$^\mathrm{a}$		97 (4) (18)^§^
QCDSF/ UKQCD 04	[66]	A					–	4.7 (2) (3)	119 (5) (8)
JLQCD 02	[70]	A					–	3.223 $(^{+46}_{-69})$	84.5 $(^{+12.0}_{-1.7})$
CP-PACS 01	[63]	A					–	3.45 (10) $(^{+11}_{-18})$	89 (2) $(^{+2}_{-6})^\star$

$^\ddagger$ What is calculated is $m_{c}/m_{s}=11.27(30)(26)$. $m_{s}$ is then obtained using lattice and phenomenological determinations of $m_{c}$ which rely on perturbation theory. Finally, $m_{ud}$ is determined from $m_{s}$ using BMW 10A, 10B’s $N_\mathrm{f}=2+1$ result for $m_{s}/m_{ud}$ [22, 23]. Since $m_{c}/m_{s}$ is renormalisation group invariant in QCD, the renormalisation and running of the quark masses enter indirectly through that of $m_{c}$, a mass that we do not review here

$^\dagger$ The calculation includes quenched e.m. effects

^§^ The data used to obtain the bare value of $m_{s}$ are from UKQCD/QCDSF 04 [66]

$^\star$ This value of $m_{s}$ was obtained using the kaon mass as input. If the $\phi$-meson mass is used instead, the authors find $m_{s} =90^{+5}_{-11}$

$^\mathrm{a}$ The masses are renormalised and run non-perturbatively up to a scale of $100\,\mathrm{GeV}$ in the $N_\mathrm{f}=2$ SF scheme. In this scheme, non-perturbative and NLO running for the quark masses are shown to agree well from 100 GeV all the way down to 2 GeV [64]

$^\mathrm{b}$ The running and renormalisation results of [64] are improved in [59] with higher statistical and systematic accuracy

$^\mathrm{c}$ The masses are renormalised non-perturbatively at scales $1/a\sim 2\div 3\,\mathrm{GeV}$ in the $N_\mathrm{f}=2$ RI/MOM scheme. In this scheme, non-perturbative and N$^3$LO running for the quark masses are shown to agree from 4 GeV down 2 GeV to better than 3 % [71]

**Table 3:** $N_\mathrm{f}=2+1$ lattice results for the masses $m_{ud}$ and $m_{s}$ (see Table 2 for notation)

Collaboration	Ref.	Publication status	Chiral extrapolation	Continuum extrapolation	Finite volume	Renormalisation	Running	$$m_{ud} $$	$$m_{s} $$
RBC/UKQCD 12$^\ominus$	[25]	A					$^\mathrm{a}$	3.37 (9) (7) (1) (2)	92.3 (1.9) (0.9) (0.4) (0.8)
PACS-CS 12$^\star$	[76]	A					$^\mathrm{b}$	3.12 (24) (8)	83.60 (0.58) (2.23)
Laiho 11	[77]	C					–	3.31 (7) (20) (17)	94.2 (1.4) (3.2) (4.7)
BMW 10A, 10B$^+$	[22, 23]	A					$^\mathrm{c}$	3.469 (47) (48)	95.5 (1.1) (1.5)
PACS-CS 10	[21]	A					$^\mathrm{b}$	2.78 (27)	86.7 (2.3)
MILC 10A	[75]	C					–	3.19 (4) (5) (16)	–
HPQCD 10$^*$	[73]	A				–	–	3.39 (6)	92.2 (1.3)
RBC/UKQCD 10A	[78]	A					$^\mathrm{a}$	3.59 (13) (14) (8)	96.2 (1.6) (0.2) (2.1)
Blum 10$^\dagger$	[32]	A					–	3.44 (12) (22)	97.6 (2.9) (5.5)
PACS-CS 09	[20]	A					$^\mathrm{b}$	2.97 (28) (3)	92.75 (58) (95)
HPQCD 09A$^\oplus$	[72]	A				–	–	3.40 (7)	92.4 (1.5)
MILC 09A	[37]	C					–	3.25 (1) (7) (16) (0)	89.0 (0.2) (1.6) (4.5) (0.1)
MILC 09	[15]	A					–	3.2 (0) (1) (2) (0)	88 (0) (3) (4) (0)
PACS-CS 08	[19]	A					–	2.527 (47)	72.72 (78)
RBC/UKQCD 08	[79]	A					–	$3.72 (16) (33) (18)$	$107.3\, (4.4) (9.7) (4.9)$
CP-PACS/JLQCD 07	[80]	A					–	$3.55 (19) (^{+56}_{-20})$	$90.1\, (4.3) (^{+16.7}_{-4.3})$
HPQCD 05	[81]	A					–	$3.2\, (0)\, (2) (2) (0)^\ddagger$	$87\, (0) (4) (4) (0)^\ddagger$
MILC 04, HPQCD/MILC/UKQCD 04	[36, 82]	A					–	$2.8\, (0)\, (1) (3) (0)$	$76\, (0) (3) (7) (0)$
HPQCD 05	[81]	A					–	$3.2\, (0) (2) (2) (0)^\ddagger$	$87\, (0) (4) (4) (0)^\ddagger$
MILC 04, HPQCD/MILC/UKQCD 04	[36, 82]	A					–	$2.8\, (0) (1) (3) (0)$	$76 (0) (3) (7) (0)$

$^\ominus$ The results are given in the ${\overline{\mathrm{MS}}}$ scheme at 3 instead of 2 GeV: $m_{ud}^{\overline{\mathrm{MS}}}(3\,\mathrm{GeV})=3.05(8)(6)(1)(2)\,\mathrm{MeV}$, $m_{s}^{\overline{\mathrm{MS}}}(3\,\mathrm{GeV})=83.5(1.7)(0.8)(0.4)(0.7)\,\mathrm{MeV}$, where the errors are statistical, chiral, finite-volume and from the perturbative matching. We run them down to 2 GeV using numerically integrated four-loop running [83, 84] with $N_\mathrm{f}=3$ and with the values of $\alpha _\mathrm{s}(M_Z)$, $m_{b}$ and $m_{c}$ taken from [74]. The running factor is 1.106. At three loops it is only 0.2 % smaller. We therefore neglect the small uncertainty associated with this conversion

$^\star$ The calculation includes e.m. and $m_{u}\ne m_{d}$ effects through reweighting

$^+$ The fermion action used is tree-level improved

$^*$ What is calculated is $m_{c}(m_{c})=1.273(6)$ GeV, using lattice results and perturbation theory. $m_{s}$ is then obtained by combing this result with HPQCD 09A’s $m_{c}/m_{s}=11.85(16)$ [72]. Finally, $m_{ud}$ is determined from $m_{s}$ with the MILC 09 result for $m_{s}/m_{ud}$. Since $m_{c}/m_{s}$ is renormalisation group invariant in QCD, the renormalisation and running of the quark masses enter indirectly through that of $m_{c}$, a mass that we do not review here

$^\dagger$ The calculation includes quenched e.m. effects

$^\oplus$ What is calculated is $m_{c}/m_{s}=11.85(16)$. $m_{s}$ is then obtained by combing this result with the determination $m_{c}(m_{c}) = 1.268\,(9)$ GeV from [85]. Finally, $m_{ud}$ is determined from $m_{s}$ with the MILC 09 result for $m_{s}/m_{ud}$

$^\ddagger$ The bare numbers are those of MILC 04. The masses are simply rescaled, using the ratio of the two-loop to one-loop renormalisation factors

$^\mathrm{a}$ The masses are renormalised non-perturbatively at a scale of 2 GeV in a couple of $N_\mathrm{f}=3$ RI/SMOM schemes. A careful study of perturbative matching uncertainties has been performed by comparing results in the two schemes in the region of 2–3 GeV [78]

$^\mathrm{b}$ The masses are renormalised and run non-perturbatively up to a scale of $40\,\mathrm{GeV}$ in the $N_\mathrm{f}=3$ SF scheme. In this scheme, non-perturbative and NLO running for the quark masses are shown to agree well from 40 GeV all the way down to 3 GeV [21]

$^\mathrm{c}$ The masses are renormalised and run non-perturbatively up to a scale of 4 GeV in the $N_\mathrm{f}=3$ RI/MOM scheme. In this scheme, non-perturbative and N$^3$LO running for the quark masses are shown to agree from 6 GeV down to 3 GeV to better than 1 % [23]

**Table 4:** Lattice results for the ratio $m_{s}/m_{ud}$

Collaboration	Ref.	$N_\mathrm{f}$	Publication status	Chiral extrapolation	Continuum extrapolation	Finite volume	$m_{s}/m_{ud}$
RBC/UKQCD 12$^\ominus$	[25]	$2+1$	A				27.36 (39) (31) (22)
PACS-CS 12$^\star$	[76]	$2+1$	A				26.8 (2.0)
Laiho 11	[77]	$2+1$	C				28.4 (0.5) (1.3)
BMW 10A, 10B$^+$	[22, 23]	$2+1$	A				27.53 (20) (8)
RBC/UKQCD 10A	[78]	$2+1$	A				26.8 (0.8) (1.1)
Blum 10$^\dagger$	[32]	$2+1$	A				28.31 (0.29) (1.77)
PACS-CS 09	[20]	$2+1$	A				31.2 (2.7)
MILC 09A	[37]	$2+1$	C				27.41 (5) (22) (0) (4)
MILC 09	[15]	$2+1$	A				27.2 (1) (3) (0) (0)
PACS-CS 08	[19]	$2+1$	A				28.8 (4)
RBC/UKQCD 08	[79]	$2+1$	A				28.8 (0.4) (1.6)
MILC 04, HPQCD/ MILC/UKQCD 04	[36, 82]	$2+1$	A				27.4 (1) (4) (0) (1)
ETM 10B	[60]	2	A				27.3 (5) (7)
RBC 07$^\dagger$	[34]	2	A				28.10 (38)
ETM 07	[62]	2	A				27.3 (0.3) (1.2)
QCDSF/UKQCD 06	[68]	2	A				27.2 (3.2)

$^\ominus$ The errors are statistical, chiral and finite-volume

$^\star$ The calculation includes e.m. and $m_{u}\ne m_{d}$ effects through reweighting

$^+$ The fermion action used is tree-level improved

$^\dagger$ The calculation includes quenched e.m. effects

#### $N_\mathrm{f}=2$ lattice calculations

We begin with $N_\mathrm{f}=2$ calculations. A quick inspection of Table 2 indicates that only the most recent calculations, ALPHA 12 [59] and ETM 10B [60], control all systematic effects—the special case of Dürr 11 [61] is discussed below. Only ALPHA 12 [59], ETM 10B [60] and ETM 07 [62] really enter the chiral regime, with pion masses down to about 270 MeV for ALPHA and ETM. Because this pion mass is still quite far from the physical pion mass, ALPHA 12 refrain from determining $m_{ud}$ and give only $m_{s}$. All the other calculations have significantly more massive pions, the lightest being about 430 MeV, in the calculation by CP-PACS 01 [63]. Moreover, the latter calculation is performed on very coarse lattices, with lattice spacings $a\ge 0.11\,\,{\mathrm {fm}}$ and only one-loop perturbation theory is used to renormalise the results.

ETM 10B’s [60] calculation of $m_{ud}$ and $m_{s}$ is an update of the earlier twisted-mass determination of ETM 07 [62]. In particular, they have added ensembles with a larger volume and three new lattice spacings, $a = 0.054, 0.067$ and $0.098\,\,{\mathrm {fm}}$, allowing for a continuum extrapolation. In addition, it presents analyses performed in SU(2) and $\hbox {SU}(3) \chi$PT.

The new ALPHA 12 [59] calculation of $m_{s}$ is an update of ALPHA 05 [64], which pushes computations to finer lattices and much lighter pion masses. It also importantly includes a determination of the lattice spacing with the decay constant $F_K$, whereas ALPHA 05 converted results to physical units using the scale parameter $r_0$ [65], defined via the force between static quarks. In particular, the conversion relied on measurements of $r_0/a$ by QCDSF/UKQCD 04 [66] which differ significantly from the new determination by ALPHA 12. As in ALPHA 05, in ALPHA 12 both non-perturbative running and non-perturbative renormalisation are performed in a controlled fashion, using Schrödinger functional methods.

The conclusion of our analysis of $N_\mathrm{f}=2$ calculations is that the results of ALPHA 12 [59] and ETM 10B [60] (which update and extend ALPHA 05 [64] and ETM 07 [62], respectively), are the only ones to date which satisfy our selection criteria. Thus we average those two results for $m_{s}$, obtaining 101(3) MeV. Regarding $m_{ud}$, for which only ETM 10B [60] gives a value, we do not offer an average but simply quote ETM’s number. Because ALPHA’s result induces an increase by 7 % of our earlier average for $m_{s}$ [1], while $m_{ud}$ remains unchanged, our average for $m_{s}/m_{ud}$ also increases by 7 %. For the latter, however, we retain the percent error quoted by ETM, who directly estimates this ratio, and add it in quadrature to the percent error on ALPHA’s $m_{s}$. Thus, we quote as our estimates:

13 $$\begin{aligned}&N_\mathrm{f}=2 : m_{s}= 101(3) \,\hbox {MeV},\ m_{ud}= 3.6(2) \,\hbox {MeV} ,\nonumber \\&\quad \frac{m_{s}}{m_{ud}} = 28.1(1.2).\end{aligned}$$

The errors on these results are 3, 6 and 4 %, respectively. The error is smaller in the ratio than one would get from combining the errors on $m_{ud}$ and $m_{s}$, because statistical and systematic errors cancel in ETM’s result for this ratio, most notably those associated with renormalisation and the setting of the scale. It is worth noting that thanks to ALPHA 12 [59], the total error on $m_{s}$ has reduced significantly, from 7 % in the last edition of our report to 3 % now. It is also interesting to remark that ALPHA 12’s [59] central value for $m_{s}$ is about 1 $\sigma$ larger than that of ETM 10B [60] and nearly 2 $\sigma$ larger than our present $N_\mathrm{f}=2+1$ determination given in (14). Moreover, this larger value for $m_{s}$ increases our $N_\mathrm{f}=2$ determination of $m_{s}/m_{ud}$, making it larger than ETM 10B’s direct measurement, though compatible within errors.

We have not discussed yet the precise results of Dürr 11 [61] which satisfy our selection criteria. This is because Dürr 11 pursue an approach which is sufficiently different from the one of other calculations that we prefer not to include it in an average at this stage. Following HPQCD 09A, 10 [72, 73], the observable which they actually compute is $m_{c}/m_{s}=11.27(30)(26)$, with an accuracy of 3.5 %. This result is about 1.5 combined standard deviations below ETM 10B’s [60] result $m_{c}/m_{s}=12.0(3)$. $m_{s}$ is subsequently obtained using lattice and phenomenological determinations of $m_{c}$ which rely on perturbation theory. The value of the charm-quark mass which they use is an average of those determinations, which they estimate to be $m_{c}(2\,\mathrm{GeV})=1.093(13)\,\mathrm{GeV}$, with a 1.2 % total uncertainty. Note that this value is consistent with the PDG average $m_{c}(2\,\mathrm{GeV})=1.094(21)\,\mathrm{GeV}$ [74], though the latter has a larger 2.0 % uncertainty. Dürr 11’s value of $m_{c}$ leads to $m_{s}=97.0(2.6)(2.5)\,\mathrm{MeV}$ given in Table 2, which has a total error of 3.7 %. The use of the PDG value for $m_{c}$ [74] would lead to a very similar result. The result for $m_{s}$ is perfectly compatible with our estimate given in (13) and has a comparable error bar. To determine $m_{ud}$, Dürr 11 combine their result for $m_{s}$ with the $N_\mathrm{f}=2+1$ calculation of $m_{s}/m_{ud}$ of BMW 10A, 10B [22, 23] discussed below. They obtain $m_{ud}=3.52(10)(9)\,\mathrm{MeV}$ with a total uncertainty of less than 4 %, which is again fully consistent with our estimate of (13) and its uncertainty.

#### $N_\mathrm{f}=2+1$ lattice calculations

We turn now to $N_\mathrm{f}=2+1$ calculations. These and the corresponding results are summarised in Tables 3 and 4. Somewhat paradoxically, these calculations are more mature than those with $N_\mathrm{f}=2$. This is thanks, in large part, to the head start and sustained effort of MILC, who have been performing $N_\mathrm{f}=2+1$ rooted staggered fermion calculations for the past ten or so years. They have covered an impressive range of parameter space, with lattice spacings which, today, go down to 0.045 fm and valence pion masses down to approximately 180 MeV [37]. The most recent updates, MILC 10A [75] and MILC 09A [37], include significantly more data and use two-loop renormalisation. Since these data sets subsume those of their previous calculations, these latest results are the only ones that must be kept in any world average.

Since our last report [1] the situation for $N_\mathrm{f}=2+1$ determinations of light quarks has undergone some evolution. There are new computations by RBC/UKQCD 12 [25], PACS-CS 12 [76] and Laiho 11 [77]. Furthermore, the results of BMW 10A, 10B [22, 23] have been published and can now be included in our averages.

The RBC/UKQCD 12 [25] computation improves on the one of RBC/UKQCD 10A [78] in a number of ways. In particular it involves a new simulation performed at a rather coarse lattice spacing of 0.144 fm, but with unitary pion masses down to 171(1) MeV and valence pion masses down to 143(1) MeV in a volume of $(4.6\,\,{\mathrm {fm}})^3$, compared, respectively, to 290 MeV, 225 MeV and $(2.7\,\,{\mathrm {fm}})^3$ in RBC/UKQCD 10A. This provides them with a significantly better control over the extrapolation to physical $M_\pi$ and to the infinite-volume limit. As before, they perform non-perturbative renormalisation and running in RI/SMOM schemes. The only weaker point of the calculation comes from the fact that two of their three lattice spacings are larger than 0.1 fm and correspond to different discretisations, while the finest is only 0.085 fm, making it difficult to convincingly claim full control over the continuum limit. This is mitigated by the fact that the scaling violations which they observe on their coarsest lattice are for many quantities small, around 5 %.

The Laiho 11 results [77] are based on MILC staggered ensembles at the lattice spacings 0.15, 0.09 and 0.06 fm, on which they propagate domain-wall quarks. Moreover, they work in volumes of up to $(4.8\,\,{\mathrm {fm}})^3$. These features give them full control over the continuum and infinite-volume extrapolations. Their lightest RMS sea pion mass is 280 MeV and their valence pions have masses down to 210 MeV. The fact that their sea pions do not enter deeply into the chiral regime penalises somewhat their extrapolation to physical $M_\pi$. Moreover, to renormalise the quark masses, they use one-loop perturbation theory for $Z_A/Z_S-1$ which they combine with $Z_A$ determined non-perturbatively from the axial-vector Ward identity. Although they conservatively estimate the uncertainty associated with the procedure to be 5 %, which is the size of their largest one-loop correction, this represents a weaker point of this calculation.

The new PACS-CS 12 [76] calculation represents an important extension of the collaboration’s earlier 2010 computation [21], which already probed pion masses down to $M_\pi \simeq 135\,\mathrm{MeV}$, i.e. down to the physical-mass point. This was achieved by reweighting the simulations performed in PACS-CS 08 [19] at $M_\pi \simeq 160\,\mathrm{MeV}$. If adequately controlled, this procedure eliminates the need to extrapolate to the physical-mass point and, hence, the corresponding systematic error. The new calculation now applies similar reweighting techniques to include electromagnetic and $m_{u}\ne m_{d}$ isospin-breaking effects directly at the physical pion mass. It technically adds to Blum 10 [32] and BMW’s preliminary results of [43, 44] by including these effects not only for valence but also for sea-quarks, as is also done in [86]. Further, as in PACS-CS 10 [21], renormalisation of quark masses is implemented non-perturbatively, through the Schrödinger functional method [87]. As it stands, the main drawback of the calculation, which makes the inclusion of its results in a world average of lattice results inappropriate at this stage, is that for the lightest quark mass the volume is very small, corresponding to $LM_\pi \simeq 2.0$, a value for which finite-volume effects will be difficult to control. Another problem is that the calculation was performed at a single lattice spacing, forbidding a continuum extrapolation. Further, it is unclear at this point what might be the systematic errors associated with the reweighting procedure.

As shown by the colour-coding in Tables 3 and 4, the BMW 10A, 10B [22, 23] calculation is still the only one to have addressed all sources of systematic effects while reaching the physical up- and down-quark mass by *interpolation* instead of by extrapolation. Moreover, their calculation was performed at five lattice spacings ranging from 0.054 to 0.116 fm, with full non-perturbative renormalisation and running and in volumes of up to (6 fm)$^3$ guaranteeing that the continuum limit, renormalisation and infinite-volume extrapolation are controlled. It does neglect, however, isospin-breaking effects, which are small on the scale of their error bars.

Finally we come to another calculation which satisfies our selection criteria, HPQCD 10 [73] (which updates HPQCD 09A [72]). The strange-quark mass is computed using a precise determination of the charm-quark mass, $m_{c}(m_{c})=1.273(6)$ GeV [73, 85], whose accuracy is better than 0.5 %, and a calculation of the quark-mass ratio $m_{c}/m_{s}=11.85(16)$ [72], which achieves a precision slightly above 1 %. The determination of $m_{s}$ via the ratio $m_{c}/m_{s}$ displaces the problem of lattice renormalisation in the computation of $m_{s}$ to one of renormalisation in the continuum for the determination of $m_{c}$. To calculate $m_{ud}$ HPQCD 10 [73] use the MILC 09 determination of the quark-mass ratio $m_{s}/m_{ud}$ [15].

The high precision quoted by HPQCD 10 on the strange-quark mass relies in large part on the precision reached in the determination of the charm-quark mass [73, 85]. This calculation uses an approach based on the lattice determination of moments of charm-quark pseudoscalar, vector and axial-vector correlators. These moments are then combined with four-loop results from continuum perturbation theory to obtain a determination of the charm-quark mass in the ${\overline{\mathrm{MS}}}$ scheme. In the preferred case, in which pseudoscalar correlators are used for the analysis, there are no lattice renormalisation factors required, since the corresponding axial-vector current is partially conserved in the staggered lattice formalism.

Instead of combining the result for $m_{c}/m_{s}$ of [72] with $m_{c}$ from [73], one can use it with the PDG [74] average $m_{c}(m_{c})=1.275(25)\,\mathrm{GeV}$, whose error is four times as large as the one obtained by HPQCD 10. If one does so, one obtains $m_{s}=92.3(2.2)$ in lieu of the value $m_{s}=92.2(1.3)$ given in Table 3, thereby nearly doubling HPQCD 10’s error. Though we plan to do so in the future, we have not yet performed a review of lattice determinations of $m_{c}$. Thus, as for the results of Dürr 11 [61] in the $N_\mathrm{f}=2$ case, we postpone its inclusion in our final averages until we have performed an independent analysis of $m_{c}$, emphasizing that this novel strategy for computing the light-quark masses may very well turn out to be the best way to determine them.

This discussion leaves us with three results for our final average for $m_{s}$, those of MILC 09A [37], BMW 10A, 10B [22, 23] and RBC/UKQCD 12 [25], and the result of HPQCD 10 [73] as an important cross-check. Thus, we first check that the three other results which will enter our final average are consistent with HPQCD 10’s result. To do this we implement the averaging procedure described in Sect. 2.2 on all four results. This yields $m_{s}=93.0(1.0)\,\mathrm{MeV}$ with a $\chi ^2/\hbox {dof} = 3.0/3=1.0$, indicating overall consistency. Note that in making this average, we have accounted for correlations in the small statistical errors of HPQCD 10 and MILC 09A. Omitting HPQCD 10 in our final average results in an increase by 50 % of the average’s uncertainty and by 0.8 $\sigma$ of its central value. Thus, we obtain $m_{s}=93.8(1.5)\,\mathrm{MeV}$ with a $\chi ^2/\hbox {dof} = 2.26/2=1.13$. When repeating the exercise for $m_{ud}$, we replace MILC 09A by the more recent analysis reported in MILC 10A [75]. A fit of all four results yields $m_{ud}=3.41(5)\,\mathrm{MeV}$ with a $\chi ^2/\hbox {dof} = 2.6/3=0.9$ and including only the same three as above gives $m_{ud}=3.42(6)\,\mathrm{MeV}$ with a $\chi ^2/\hbox {dof} = 2.4/2=1.2$. Here the results are barely distinguishable, indicating full compatibility of all four results. Note that the outcome of the averaging procedure amounts to a determination of $m_{s}$ and $m_{ud}$ of 1.6 and 1.8 %, respectively.

The heavy sea-quarks affect the determination of the light-quark masses only through contributions of order $1/m_{c}^2$, which moreover are suppressed by the Okubo–Zweig–Iizuka-rule. We expect these contributions to be small. However, note that the effect of omitted sea quarks on a given quantity is not uniquely defined: the size of the effect depends on how the theories with and without these flavours are matched. One way to set conventions is to ensure that the bare parameters common to both theories are fixed by the same physical observables and that the renormalisations are performed in the same scheme and at the same scale, with the appropriate numbers of flavours.

An upper bound on the heavy-quark contributions can be obtained by looking at the presumably much larger effect associated with omitting the strange quark in the sea. Within errors, the average value $m_{ud} = 3.42(6)$ MeV obtained above from the data with $N_\mathrm{f} = 2+1$ agrees with the result $m_{ud} = 3.6(2)$ MeV for $N_\mathrm{f} = 2$ quoted in (13): assuming that the underlying calculations more or less follow the above matching prescription, the effects generated by the quenching of the strange quark in $m_{ud}$ are within the noise. Interpreting the two results as Gaussian distributions, the probability distribution of the difference $\Delta m_{ud} \equiv (m_{ud}|_{N_\mathrm{f}=2})- (m_{ud}|_{N_\mathrm{f}=3})$ is also Gaussian, with $\Delta m_{ud}=0.18(21)$ MeV. The corresponding root-mean-square $\langle \Delta m_{ud}^2\rangle ^\frac{1}{2}= 0.28$ MeV provides an upper bound for the size of the effects due to strange quark quenching; it amounts to 8 % of $m_{ud}$. In the case of $m_{s}$, the analogous calculation yields $\langle \Delta m_{s}^2\rangle ^\frac{1}{2}=7.9$ MeV and thus also amounts to an upper bound of about 8 %. Taking any of these numbers as an upper bound on the omission of charm effects in the $N_\mathrm{f}=2+1$ results is, we believe, a significant overestimate.

An underestimate of the upper bound on the sea-charm contributions to $m_{s}$ can be obtained by transposing, to the $s\bar{s}$ system, the perturbative, heavy quarkonium arguments put forward in [94] to determine the effect of sea charm on the $\eta _{c}$ and $J/\psi$ masses. An estimate using constituent quark masses [95] leads very roughly to a 0.05 % effect on $m_{s}$, from which [95] concludes that the error on $m_{s}$ and $m_{ud}$ due to the omission of charm is of order 0.1 %.

One could also try to estimate the effect by analysing the relation between the parameters of QCD$_3$ and those of full QCD in perturbation theory. The $\beta$- and $\gamma$-functions, which control the renormalisation of the coupling constants and quark masses, respectively, are known to four loops [83, 84, 96, 97]. The precision achieved in this framework for the decoupling of the $t$- and $b$-quarks is excellent, but the $c$-quark is not heavy enough: at the percent level, we believe that the corrections of order $1/m_{c}^2$ cannot be neglected and the decoupling formulae of perturbation theory do not provide a reliable evaluation, because the scale $m_{c}(m_{c})\simeq 1.28\,\mathrm{GeV}$ is too low for these formulae to be taken at face value. Consequently, the accuracy to which it is possible to identify the running masses of the light quarks of full QCD in terms of those occurring in QCD$_3$ is limited. For this reason, it is preferable to characterise the masses $m_{u}$, $m_{d}$, $m_{s}$ in terms of QCD$_4$, where the connection with full QCD is under good control.

The role of the $c$-quarks in the determination of the light-quark masses will soon be studied in detail—some simulations with $2+1+1$ dynamical quarks have already been carried out [24, 98]. For the moment, we choose to consider a crude, and hopefully reasonably conservative, upper bound on the size of the effects due to the neglected heavy quarks that can be established within the $N_\mathrm{f}=2+1$ simulations themselves, without invoking perturbation theory. In [99] it is found that when the scale is set by $M_\Xi$, the result for $M_\Lambda$ agrees well with experiment within the 2.3 % accuracy of the calculation. Because of the very strong correlations between the statistical and systematic errors of these two masses, we expect the uncertainty in the difference $M_\Xi -M_\Lambda$ to also be of order 2 %. To leading order in the chiral expansion this mass difference is proportional to $m_{s}-m_{ud}$. Barring accidental cancellations, we conclude that the agreement of $N_\mathrm{f}= 2+1$ calculations with experiment suggests an upper bound on the sensitivity of $m_{s}$ to heavy sea-quarks of order 2 %.

**Fig. 1:**
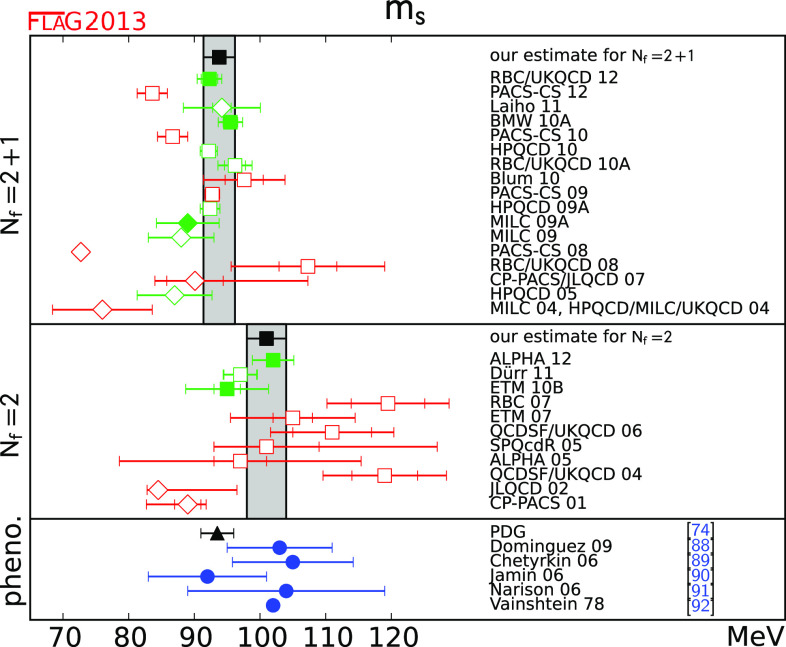
Mass of the strange quark (${\overline{\mathrm{MS}}}$ scheme, running scale 2 GeV). The *central* and *top panels* show the lattice results listed in Tables 2 and 3. For comparison, the *bottom panel* collects a few sum rule results and also indicates the current PDG estimate. *Diamonds* represent results based on perturbative renormalisation, while *squares* indicate that, in the relation between the lattice regularised and renormalised ${\overline{\mathrm{MS}}}$ masses, non-perturbative effects are accounted for. The *black squares* and the *grey bands* represent our estimates (13) and (14). The significance of the colours is explained in Sect. 2

**Fig. 2:**
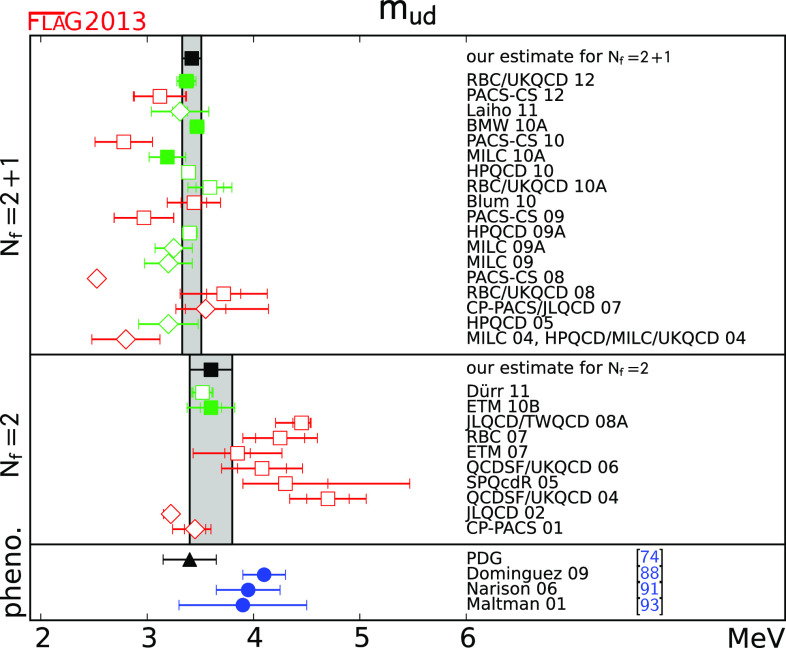
Mean mass of the two lightest quarks, $m_{ud}=\frac{1}{2}(m_{u}+m_{d})$ (for details see Fig. 1)

Taking this uncertainty into account yields the following averages:

14 $$\begin{aligned} N_\mathrm{f}\!=\!2\!+\!1 : m_{ud} \!=\! 3.42(6)(7) ;\mathrm{MeV},\ m_{s}\!=\! 93.8(1.5)(1.9);\mathrm{MeV},\nonumber \\ \end{aligned}$$

where the first error comes from the averaging of the lattice results, and the second is the one that we add to account for the neglect of sea effects from the charm and more massive quarks. This corresponds to determinations of $m_{ud}$ and $m_{s}$ with a precision of and 2.6 and 2.7 %, respectively. These estimates represent the conclusions we draw from the information gathered on the lattice until now. They are shown as vertical bands in Figs. 1 and 2, together with the $N_\mathrm{f}=2$ results (13).

In the ratio $m_{s}/m_{ud}$, one of the sources of systematic error—the uncertainties in the renormalisation factors—drops out. Also, we can compare the lattice results with the leading-order formula of $\chi$PT,

15 $$\begin{aligned} \frac{m_{s}}{m_{ud}}\mathop {=}\limits ^{\mathrm{LO}}\frac{\hat{M}_{K^+}^2+ \hat{M}_{K^0}^2-\hat{M}_{\pi ^+}^2}{\hat{M}_{\pi ^+}^2},\end{aligned}$$

which relates the quantity $m_{s}/m_{ud}$ to a ratio of meson masses in QCD. Expressing these in terms of the physical masses and the four coefficients introduced in (6)–(8), linearizing the result with respect to the corrections and inserting the observed mass values, we obtain

16 $$\begin{aligned} \frac{m_{s}}{m_{ud}} \mathop {=}\limits ^{\mathrm{LO}}25.9 - 0.1\, \epsilon + 1.9\, \epsilon _{\pi ^0} - 0.1\, \epsilon _{K^0} -1.8 \,\epsilon _{m}.\end{aligned}$$

If the coefficients $\epsilon$, $\epsilon _{\pi ^0}$, $\epsilon _{K^0}$ and $\epsilon _{m}$ are set equal to zero, the right hand side reduces to the value $m_{s}/m_{ud}=25.9$ that follows from Weinberg’s leading-order formulae for $m_{u}/m_{d}$ and $m_{s}/m_{d}$ [100], in accordance with the fact that these do account for the e.m. interaction at leading chiral order, and neglect the mass difference between the charged and neutral pions in QCD. Inserting the estimates (9) gives the effect of chiral corrections to the e.m. self-energies and of the mass difference between the charged and neutral pions in QCD. With these, the LO prediction in QCD becomes

17 $$\begin{aligned} \frac{m_{s}}{m_{ud}}\mathop {=}\limits ^{\mathrm{LO}}25.9(1).\end{aligned}$$

**Fig. 3:**
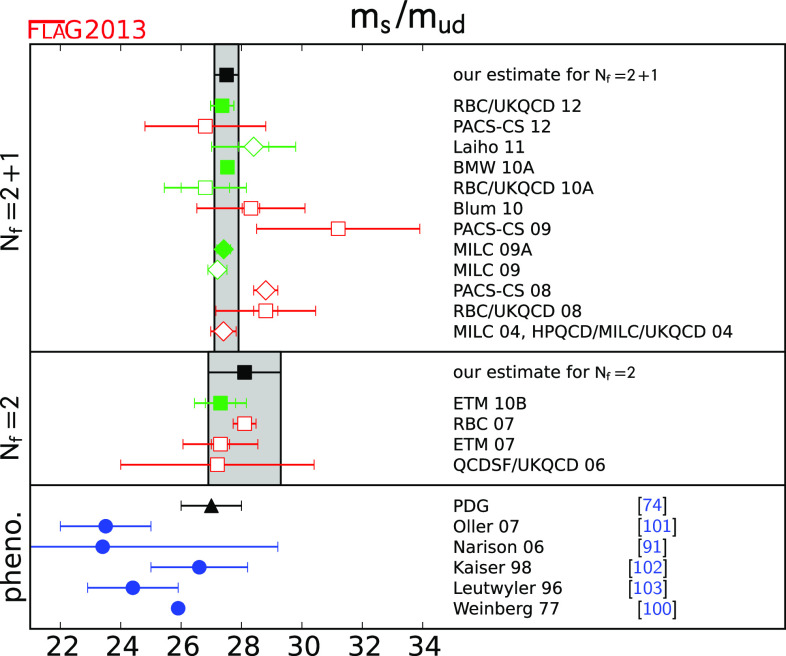
Results for the ratio $m_{s}/m_{ud}$. The *upper* part indicates the lattice results listed in Table 4. The *lower* part shows results obtained from $\chi$PT and sum rules, together with the current PDG estimate

The quoted uncertainty does not include an estimate for the higher-order contributions, but it only accounts for the error bars in the coefficients, which is dominated by the one in the estimate given for $\epsilon _{\pi ^0}$. The fact that the central value remains unchanged indicates that chiral corrections to the e.m. self-energies and mass-difference corrections are small in this particular quantity. However, given the high accuracy reached in lattice determinations of the ratio $m_{s}/m_{ud}$, the uncertainties associated with e.m. corrections are no longer completely irrelevant. This is seen by comparing the 0.1 in (17) with the 0.15 in (18). Nevertheless, this uncertainty is still smaller than our $\sim 1.\div 1.5\,\%$ upper bound on possible $1/m_{c}^2$ corrections (Fig. 3).

The lattice results in Table 4, which satisfy our selection criteria, indicate that the corrections generated by the nonleading terms of the chiral perturbation series are remarkably small, in the range 3–10 %. Despite the fact that the SU(3)-flavour-symmetry-breaking effects in the Nambu–Goldstone boson masses are very large ($M_K^2\simeq 13\, M_\pi ^2$), the mass spectrum of the pseudoscalar octet obeys the SU(3) $\times$ SU(3) formula (15) very well.

Our average for $m_{s}/m_{ud}$ is based on the results of MILC 09A, BMW 10A, 10B and RBC/UKQCD 12—the value quoted by HPQCD 10 does not represent independent information as it relies on the result for $m_{s}/m_{ud}$ obtained by the MILC collaboration. Averaging these results according to the precription of Sect. 2.3 gives $m_{s}/m_{ud}=27.46(15)$ with $\chi ^2/\hbox {dof}=0.2/2$. The fit is dominated by MILC 09A and BMW 10A, 10B. Since the errors associated with renormalisation drop out in the ratio, the uncertainties are even smaller than in the case of the quark masses themselves: the above number for $m_{s}/m_{ud}$ amounts to an accuracy of 0.5 %.

At this level of precision, the uncertainties in the electromagnetic and strong isospin-breaking corrections are not completely negligible. The error estimate in the LO result (17) indicates the expected order of magnitude. The uncertainties in $m_{s}$ and $m_{ud}$ associated with the heavy sea-quarks cancel at least partly. In view of this, we ascribe a total 1.5 % uncertainty to these two sources of error. Thus, we are convinced that our final estimate,

18 $$\begin{aligned} N_\mathrm{f}=2+1 :\quad \frac{m_{s}}{m_{ud}}=27.46(15)(41),\end{aligned}$$

is on the conservative side, with a total 1.5 % uncertainty. It is also fully consistent with the ratio computed from our individual quark masses in (14), $m_{s}/m_{ud}=27.6(6)$, which has a larger 2.2 % uncertainty. In (18) the first error comes from the averaging of the lattice results, and the second is the one that we add to account for the neglect of isospin-breaking and heavy sea-quark effects.

The lattice results show that the LO prediction of $\chi$PT in (17) receives only small corrections from higher orders of the chiral expansion: according to (18), these generate a shift of $5.7\pm 1.5\, \%$. Our estimate does therefore not represent a very sharp determination of the higher-order contributions.

The ratio $m_{s}/m_{ud}$ can also be extracted from the masses of the neutral Nambu–Goldstone bosons: neglecting effects of order $(m_{u}-m_{d})^2$ also here, the leading-order formula reads $m_{s}/m_{ud}\mathop {=}\limits ^{\mathrm{LO}}\frac{3}{2}\hat{M}_\eta ^2/\hat{M}_\pi ^2-\frac{1}{2}$. Numerically, this gives $m_{s}/m_{ud}\mathop {=}\limits ^{\mathrm{LO}}24.2$. The relation has the advantage that the e.m. corrections are expected to be much smaller here, but it is more difficult to calculate the $\eta$-mass on the lattice. The comparison with (18) shows that, in this case, the contributions of NLO are somewhat larger: $14\pm 2$ %.

### Lattice determination of $m_{u}$ and $m_{d}$

The determination of $m_{u}$ and $m_{d}$ separately requires additional input. MILC 09A [37] uses the mass difference between $K^0$ and $K^+$, from which they subtract electromagnetic effects using Dashen’s theorem with corrections, as discussed in Sect. 3.1. The up- and down- sea-quarks remain degenerate in their calculation, fixed to the value of $m_{ud}$ obtained from $M_{\pi ^0}$.

To determine $m_{u}/m_{d}$, BMW 10A, 10B [22, 23] follow a slightly different strategy. They obtain this ratio from their result for $m_{s}/m_{ud}$ combined with a phenomenological determination of the isospin-breaking quark-mass ratio $Q=22.3(8)$, defined below in (24), from $\eta \rightarrow 3\pi$ decays [30] (the decay $\eta \rightarrow 3\pi$ is very sensitive to QCD isospin-breaking but fairly insensitive to QED isospin-breaking). As discussed in Sect. 3.5, the central value of the e.m. parameter $\epsilon$ in (9) is taken from the same source.

RM123 11 [105] actually uses the e.m. parameter $\epsilon =0.7(5)$ from the first edition of the FLAG review [1]. However, they estimate the effects of strong isospin-breaking at first non-trivial order, by inserting the operator $\frac{1}{2}(m_{u}-m_{d})\int (\bar{u}u-\bar{d}d)$ into correlation functions, while performing the gauge averages in the isospin limit. Applying these techniques, they obtain $(\hat{M}_{K^0}^2-\hat{M}_{K^+}^2)/(m_{d}-m_{u})=2.57(8)\,\mathrm{MeV}$. Combining this result with the phenomenological $(\hat{M}_{K^0}^2-\hat{M}_{K^+}^2)=6.05(63)\times 10^3$ determined with the above value of $\epsilon$, they get $(m_{d}-m_{u})=2.35(8)(24)\,\mathrm{MeV}$, where the first error corresponds to the lattice statistical and systematic uncertainties combined in quadrature, while the second arises from the uncertainty on $\epsilon$. Note that below we quote results from RM123 11 for $m_{u}$, $m_{d}$ and $m_{u}/m_{d}$. As described in Table 5, we obtain them by combining RM123 11’s result for $(m_{d}-m_{u})$ with ETM 10B’s result for $m_{ud}$.

**Table 5:** Lattice results for $m_{u}$, $m_{d}$ (MeV) and for the ratio $m_{u}/m_{d}$. The values refer to the ${\overline{\mathrm{MS}}}$ scheme at scale 2 GeV. The upper part of the table lists results obtained with $N_\mathrm{f}=2+1$, while the lower part presents calculations with $N_\mathrm{f} = 2$

Collaboration	Ref.	Publication status	Chiral extrapolation	Continuum extrapolation	Finite volume	Renormalisation	Running	$m_{u}$	$m_{d}$	$m_{u}/m_{d}$
PACS-CS 12$^\star$	[76]	A					$\,a$	2.57 (26) (7)	3.68 (29) (10)	0.698 (51)
Laiho 11	[77]	C					–	1.90 (8) (21) (10)	4.73 (9) (27) (24)	0.401 (13) (45)
HPQCD 10$^\ddagger$	[73]	A					–	2.01 (14)	4.77 (15)	
BMW 10A, 10B$^+$	[22, 23]	A					$\,b$	2.15 (03) (10)	4.79 (07) (12)	0.448 (06) (29)
Blum 10$^\dagger$	[32]	A					–	2.24 (10) (34)	4.65 (15) (32)	0.4818 (96) (860)
MILC 09A	[37]	C					–	1.96 (0) (6) (10) (12)	4.53 (1) (8) (23) (12)	0.432 (1) (9) (0) (39)
MILC 09	[15]	A					–	1.9 (0) (1) (1) (1)	4.6 (0) (2) (2) (1)	0.42 (0) (1) (0) (4)
MILC 04, HPQCD/ MILC/UKQCD 04	[36, 82]	A					–	1.7 (0) (1) (2) (2)	3.9 (0) (1) (4) (2)	0.43 (0) (1) (0) (8)
RM123 13	[45]	A					$\,c$	2.40 (15) (17)	4.80 (15) (17)	0.50 (2) (3)
RM123 11$^\oplus$	[105]	A					$\,c$	*2.43 (11) (23)*	*4.78 (11) (23)*	*0.51 (2) (4)*
Dürr 11$^*$	[61]	A				–	–	2.18 (6) (11)	4.87 (14) (16)	
RBC 07$^\dagger$	[34]	A					–	3.02 (27) (19)	5.49 (20) (34)	0.550 (31)

$^\star$ The calculation includes e.m. and $m_{u}\ne m_{d}$ effects through reweighting

$^\ddagger$ Values obtained by combining the HPQCD 10 result for $m_{s}$ with the MILC 09 results for $m_{s}/m_{ud}$ and $m_{u}/m_{d}$

$^+$ The fermion action used is tree-level improved

$^*$ Values obtained by combining the Dürr 11 result for $m_{s}$ with the BMW 10A, 10B results for $m_{s}/m_{ud}$ and $m_{u}/m_{d}$. $^\oplus m_{u}$, $m_{d}$ and $m_{u}/m_{d}$ are obtained by combining the result of RM123 11 for $(m_{d}-m_{u})$ [105] with $m_{ud}=3.6(2)\,\mathrm{MeV}$ from ETM 10B. $(m_{d}-m_{u})=2.35(8)(24)\,\mathrm{MeV}$ in [105] was obtained assuming $\epsilon = 0.7(5)$ [1] and $\epsilon _{m}=\epsilon _{\pi ^0}=\epsilon _{K^0}=0$. In the quoted results, the first error corresponds to the lattice statistical and systematic errors combined in quadrature, while the second arises from the uncertainties associated with $\epsilon$

$^\dagger$ The calculation includes quenched e.m. effects

$a$ The masses are renormalised and run non-perturbatively up to a scale of $100\,\mathrm{GeV}$ in the $N_\mathrm{f}=2$ SF scheme. In this scheme, non-perturbative and NLO running for the quark masses are shown to agree well from 100 GeV all the way down to 2 GeV [64]

$b$ The masses are renormalised and run non-perturbatively up to a scale of 4 GeV in the $N_\mathrm{f}=3$ RI/MOM scheme. In this scheme, non-perturbative and N$^3$LO running for the quark masses are shown to agree from 6 GeV down to 3 GeV to better than 1 % [23]

$c$ The masses are renormalised non-perturbatively at scales $1/a\sim 2\div 3\,\mathrm{GeV}$ in the $N_\mathrm{f}=2$ RI/MOM scheme. In this scheme, non-perturbative and N$^3$LO running for the quark masses are shown to agree from 4 GeV down 2 GeV to better than 3 % [71]

Instead of subtracting electromagnetic effects using phenomenology, RBC 07 [34] and Blum 10 [32] actually include a quenched electromagnetic field in their calculation. This means that their results include corrections to Dashen’s theorem, albeit only in the presence of quenched electromagnetism. Since the up- and down-quarks in the sea are treated as degenerate, very small isospin corrections are neglected, as in MILC’s calculation.

PACS-CS 12 [76] takes the inclusion of isospin-breaking effects one step further. Using reweighting techniques, it also includes electromagnetic and $m_{u}-m_{d}$ effects in the sea.

Lattice results for $m_{u}$, $m_{d}$ and $m_{u}/m_{d}$ are summarised in Table 5. In order to discuss them, we consider the LO formula

19 $$\begin{aligned} \frac{m_{u}}{m_{d}}\mathop {=}\limits ^{\mathrm{LO}}\frac{\hat{M}_{K^+}^2-\hat{M}_{K^0}^2+\hat{M}_{\pi ^+}^2}{\hat{M}_{K^0}^2-\hat{M}_{K^+}^2+\hat{M}_{\pi ^+}^2} .\end{aligned}$$

Using Eqs. (6)–(8) to express the meson masses in QCD in terms of the physical ones and linearizing in the corrections, this relation takes the form

20 $$\begin{aligned} \frac{m_{u}}{m_{d}}\mathop {=}\limits ^{\mathrm{LO}}0.558 - 0.084\, \epsilon - 0.02\, \epsilon _{\pi ^0} + 0.11\, \epsilon _{m} .\end{aligned}$$

Inserting the estimates (9) and adding errors in quadrature, the LO prediction becomes

21 $$\begin{aligned} \frac{m_{u}}{m_{d}}\mathop {=}\limits ^{\mathrm{LO}}0.50(3).\end{aligned}$$

Again, the quoted error exclusively accounts for the errors attached to the estimates (9) for the epsilons—contributions of non-leading order are ignored. The uncertainty in the leading-order prediction is dominated by the one in the coefficient $\epsilon$, which specifies the difference between the meson squared-mass splittings generated by the e.m. interaction in the kaon and pion multiplets. The reduction in the error on this coefficient since the previous review [1] results in a reduction of a factor of a little less than 2 in the uncertainty on the LO value of $m_{u}/m_{d}$ given in (21).

It is interesting to compare the assumptions made or results obtained by the different collaborations for the violation of Dashen’s theorem. The input used in MILC 09A is $\epsilon =1.2(5)$ [37], while the $N_\mathrm{f}=2$ computation of RM123 13 finds $\epsilon =0.79(18)(18)$ [45]. As discussed in Sect. 3.5, the value of $Q$ used by BMW 10A, 10B [22, 23] gives $\epsilon =0.70(28)$ at NLO (see (31)). On the other hand, RBC 07 [34] and Blum 10 [32] obtain the results $\epsilon =0.13(4)$ and $\epsilon =0.5(1)$. Note that PACS-CS 12 [76] do not provide results which allow us to determine $\epsilon$ directly. However, using their result for $m_{u}/m_{d}$, together with (20), and neglecting NLO terms, one finds $\epsilon =-1.6(6)$, which is difficult to reconcile with what is known from phenomenology (see Sects. 3.1 and 3.5). Since the values assumed or obtained for $\epsilon$ differ, it does not come as a surprise that the determinations of $m_{u}/m_{d}$ are different.

These values of $\epsilon$ are also interesting because they allow us to estimate the chiral corrections to the LO prediction (21) for $m_{u}/m_{d}$. Indeed, evaluating the relation (20) for the values of $\epsilon$ given above, and neglecting all other corrections in this equation, yields the LO values $(m_{u}/m_{d})^{\mathrm {LO}}=0.46(4)$, 0.547(3), 0.52(1), 0.50(2), 0.49(2) for MILC 09A, RBC 07, Blum 10, BMW 10A, 10B and RM123 13, respectively. However, in comparing these numbers to the non-perturbative results of Table 5 one must be careful not to double count the uncertainty arising from $\epsilon$. One way to obtain a sharp comparison is to consider the ratio of the results of Table 5 to the LO values $(m_{u}/m_{d})^\mathrm{LO}$, in which the uncertainty from $\epsilon$ cancels to good accuracy. Here we will assume for simplicity that they cancel completely and will drop all uncertainties related to $\epsilon$. For $N_\mathrm{f} = 2$ we consider RM123 13 [45], which updates RM123 11 and has no red dots. Since the uncertainties common to $\epsilon$ and $m_{u}/m_{d}$ are not explicitly given in [45], we have to estimate them. For that we use the leading-order result for $m_{u}/m_{d}$, computed with RM123 13’s value for $\epsilon$. Its error bar is the contribution of the uncertainty on $\epsilon$ to $(m_{u}/m_{d})^\mathrm{LO}$. To good approximation this contribution will be the same for the value of $m_{u}/m_{d}$ computed in [45]. Thus, we subtract it in quadrature from RM123 13’s result in Table 5 and compute $(m_{u}/m_{d})/(m_{u}/m_{d})^\mathrm{LO}$, dropping uncertainties related to $\epsilon$. We find $(m_{u}/m_{d})/(m_{u}/m_{d})^\mathrm{LO} = 1.02(6)$. This result suggests that chiral corrections in the case of $N_\mathrm{f}=2$ are negligible. For the two most accurate $N_\mathrm{f}=2+1$ calculations, those of MILC 09A and BMW 10A, 10B, this ratio of ratios is 0.94(2) and 0.90(1), respectively. Though these two numbers are not fully consistent within our rough estimate of the errors, they indicate that higher-order corrections to (21) are negative and about 8 % when $N_\mathrm{f}=2+1$. In the following, we will take them to be $-$8(4) %. The fact that these corrections are seemingly larger and of opposite sign than in the $N_\mathrm{f}=2$ case is not understood at this point. It could be an effect associated with the quenching of the strange quark. It could also be due to the fact that the RM123 13 calculation does not probe deeply enough into the chiral regime—it has $M_\pi \gtrsim 270\,\mathrm{MeV}$—to pick up on important chiral corrections. Of course, being less than a two standard deviation effect, it may be that there is no problem at all and that differences from the LO result are actually small.

Given the exploratory nature of the RBC 07 calculation, its results do not allow us to draw solid conclusions about the e.m. contributions to $m_{u}/m_{d}$ for $N_\mathrm{f}=2$. As discussed in Sect. 3.3.2, the $N_\mathrm{f}=2+1$ results of Blum 10 and PACS-CS 12 do not pass our selection criteria either. We therefore resort to the phenomenological estimates of the electromagnetic self-energies discussed in Sect. 3.1, which are validated by recent, preliminary lattice results.

Since RM123 13 [45] includes a lattice estimate of e.m. corrections, for the $N_\mathrm{f}=2$ final results we simply quote the values of $m_{u}$, $m_{d}$ and $m_{u}/m_{d}$ from RM123 13 given in Table 5:

22 $$\begin{aligned} N_\mathrm{f}&= 2: m_{u} =2.40(23)\,\mathrm{MeV},\quad m_{d} = 4.80(23) \,\mathrm{MeV},\nonumber \\&\frac{m_{u}}{m_{d}} = 0.50(4), \end{aligned}$$

with errors of roughly 10, 5 and 8 %, respectively. In these results, the errors are obtained by combining the lattice statistical and systematic errors in quadrature.

For $N_\mathrm{f}=2+1$ there is to date no final, published computation of e.m. corrections. Thus, we take the LO estimate for $m_{u}/m_{d}$ of (21) and use the $-$8(4) % obtained above as an estimate of the size of the corrections from higher orders in the chiral expansion. This gives $m_{u}/m_{d}=0.46(3)$. The two individual masses can then be worked out from the estimate (14) for their mean. Therefore, for $N_\mathrm{f}=2+1$ we obtain

23 $$\begin{aligned}&N_\mathrm{f}= 2+1: m_{u} =2.16(9)(7)\,\mathrm{MeV},\nonumber \\&\quad m_{d} = 4.68(14)(7) \,\mathrm{MeV},\frac{m_{u}}{m_{d}} = 0.46(2)(2).\end{aligned}$$

In these results, the first error represents the lattice statistical and systematic errors, combined in quadrature, while the second arises from the uncertainties associated with e.m. corrections of (9). The estimates in (23) have uncertainties of order 5, 3 and 7 %, respectively.

Naively propagating errors to the end, we obtain $(m_{u}/m_{d})_{N_\mathrm{f}=2}/(m_{u}/m_{d})_{N_\mathrm{f}=2+1}=1.09(10)$. If instead of (22) we use the results from RM123 11, modified by the e.m. corrections in (9), as was done in our previous review, we obtain $(m_{u}/m_{d})_{N_\mathrm{f}=2}/(m_{u}/m_{d})_{N_\mathrm{f}=2+1}=1.11(7)(1)$, confirming again the strong cancellation of e.m. uncertainties in the ratio. The $N_\mathrm{f}=2$ and $2+1$ results are compatible at the 1 to 1.5 $\sigma$ level.

It is interesting to note that in the results above, the errors are no longer dominated by the uncertainties in the input used for the electromagnetic corrections, though these are still significant at the level of precision reached in the $N_\mathrm{f}=2+1$ results. This is due to the reduction in the error on $\epsilon$ discussed in Sect. 3.1. Nevertheless, the comparison of Eqs. (21) and (23) indicates that more than half of the difference between the prediction $m_{u}/m_{d}=0.558$ obtained from Weinberg’s mass formulae [100] and the result for $m_{u}/m_{d}$ obtained on the lattice stems from electromagnetism, the higher orders in the chiral perturbation generating a comparable correction.

In view of the fact that a *massless up-quark* would solve the strong CP-problem, many authors have considered this an attractive possibility, but the results presented above exclude this possibility: the value of $m_{u}$ in (23) differs from zero by 20 standard deviations. We conclude that nature solves the strong CP-problem differently. This conclusion relies on lattice calculations of kaon masses and on the phenomenological estimates of the e.m. self-energies discussed in Sect. 3.1. The uncertainties therein currently represent the limiting factor in determinations of $m_{u}$ and $m_{d}$. As demonstrated in [32–34, 40–44, 50], lattice methods can be used to calculate the e.m. self-energies. Further progress on the determination of the light-quark masses hinges on an improved understanding of the e.m. effects.

### Estimates for $R$ and $Q$

The quark-mass ratios

24 $$\begin{aligned} R\equiv \frac{m_{s}-m_{ud}}{m_{d}-m_{u}} \quad \hbox {and}\quad Q^2\equiv \frac{m_{s}^2-m_{ud}^2}{m_{d}^2-m_{u}^2} \end{aligned}$$

compare SU(3)-breaking with isospin-breaking. The quantity $Q$ is of particular interest because of a low-energy theorem [106], which relates it to a ratio of meson masses,

25 $$\begin{aligned}&Q^2_M\equiv \frac{\hat{M}_K^2}{\hat{M}_\pi ^2}\cdot \frac{\hat{M}_K^2-\hat{M}_\pi ^2}{\hat{M}_{K^0}^2-\hat{M}_{K^+}^2} ,\quad \hat{M}^2_\pi \equiv \frac{1}{2}\left( \hat{M}^2_{\pi ^+}+ \hat{M}^2_{\pi ^0}\right) ,\nonumber \\&\quad \hat{M}^2_K\equiv \frac{1}{2}\left( \hat{M}^2_{K^+}+\hat{M}^2_{K^0}\right) .\end{aligned}$$

Chiral symmetry implies that the expansion of $Q_M^2$ in powers of the quark masses (i) starts with $Q^2$ and (ii) does not receive any contributions at NLO:

26 $$\begin{aligned} Q_M\mathop {=}\limits ^{\mathrm{NLO}}Q .\end{aligned}$$

Inserting the estimates for the mass ratios $m_{s}/m_{ud}$ and $m_{u}/m_{d}$ given for $N_\mathrm{f}=2$ in Eqs. (13) and (22), respectively, we obtain

27 $$\begin{aligned} R=40.7(3.7)(2.2),\quad Q=24.3(1.4)(0.6) , \end{aligned}$$

where the errors have been propagated naively and the e.m. uncertainty has been separated out, as discussed in the third paragraph after (21). Thus, the meaning of the errors is the same as in (23). These numbers agree within errors with those reported in [45] where values for $m_{s}$ and $m_{ud}$ are taken from ETM 10B [60].

For $N_\mathrm{f}=2+1$, we use Eqs. (18) and (23) and obtain

28 $$\begin{aligned} R=35.8(1.9)(1.8),\quad Q=22.6(7)(6), \end{aligned}$$

where the meaning of the errors is the same as above. The $N_\mathrm{f}=2$ and $N_\mathrm{f}=2+1$ results are compatible within 2$\sigma$, even taking the correlations between e.m. effects into account.

It is interesting to use these results to study the size of chiral corrections in the relations of $R$ and $Q$ to their expressions in terms of meson masses. To investigate this issue, we use $\chi$PT to express the quark-mass ratios in terms of the pion and kaon masses in QCD and then again use Eqs. (6)–(8) to relate the QCD masses to the physical ones. Linearizing in the corrections, this leads to

29 $$\begin{aligned}&R \mathop {=}\limits ^{\mathrm{LO}}R_M \!=\! 43.9 - 10.8\, \epsilon \!+\! 0.2\, \epsilon _{\pi ^0} \!-\! 0.2\, \epsilon _{K^0}\!-\! 10.7\, \epsilon _{m},\nonumber \\\end{aligned}$$

30 $$\begin{aligned}&Q \mathop {=}\limits ^{\mathrm{NLO}}Q_M \!=\! 24.3 \!-\! 3.0\, \epsilon \!+\! 0.9\, \epsilon _{\pi ^0} \!-\! 0.1\, \epsilon _{K^0} \!+\! 2.6 \,\epsilon _{m} .\qquad \end{aligned}$$

While the first relation only holds to LO of the chiral perturbation series, the second remains valid at NLO, on account of the low energy theorem mentioned above. The first terms on the right hand side represent the values of $R$ and $Q$ obtained with the Weinberg leading-order formulae for the quark-mass ratios [100]. Inserting the estimates (9), we find that the e.m. corrections lower the Weinberg values to $R_M= 36.7(3.3)$ and $Q_M= 22.3(9)$, respectively.

Comparison of $R_M$ and $Q_M$ with the full results quoted above gives a handle on higher-order terms in the chiral expansion. Indeed, the ratios $R_M/R$ and $Q_M/Q$ give NLO and NNLO (and higher) corrections to the relations $R \mathop {=}\limits ^{\mathrm{LO}}R_M$ and $Q\mathop {=}\limits ^{\mathrm{NLO}}Q_M$, respectively. The uncertainties due to the use of the e.m. corrections of (9) are highly correlated in the numerators and denominators of these ratios, and we make the simplifying assumption that they cancel in the ratio. Thus, for $N_\mathrm{f}=2$ we evaluate (29) and (30) using $\epsilon =0.79(18)(18)$ from RM123 13 [45] and the other corrections from (9), dropping all uncertainties. We divide them by the results for $R$ and $Q$ in (27), omitting the uncertainties due to e.m. We obtain $R_M/R\simeq 0.88(8)$ and $Q_M/Q\simeq 0.91(5)$. We proceed analogously for $N_\mathrm{f}=2+1$, using $\epsilon =0.70(3)$ from (9) and $R$ and $Q$ from (28), and find $R_M/R\simeq 1.02(5)$ and $Q_M/Q\simeq 0.99(3)$. The chiral corrections appear to be small for $N_\mathrm{f}=2+1$, especially those in the relation of $Q$ to $Q_M$. This is less true for $N_\mathrm{f}=2$, where the NNLO and higher corrections to $Q=Q_M$ could be significant. However, as for other quantities which depend on $m_{u}/m_{d}$, this difference is not significant.

As mentioned in Sect. 3.1, there is a phenomenological determination of $Q$ based on the decay $\eta \rightarrow 3\pi$ [107, 108]. The key point is that the transition $\eta \rightarrow 3\pi$ violates isospin-conservation. The dominating contribution to the transition amplitude stems from the mass difference $m_{u}-m_{d}$. At NLO of $\chi$PT, the QCD part of the amplitude can be expressed in a parameter-free manner in terms of $Q$. It is well-known that the electromagnetic contributions to the transition amplitude are suppressed (a thorough recent analysis is given in [109]). This implies that the result for $Q$ is less sensitive to the electromagnetic uncertainties than the value obtained from the masses of the Nambu–Goldstone bosons. For a recent update of this determination and for further references to the literature, we refer to [110]. Using dispersion theory to pin down the momentum-dependence of the amplitude, the observed decay rate implies $Q=22.3(8)$ (since the uncertainty quoted in [110] does not include an estimate for all sources of error, we have retained the error estimate given in [104], which is twice as large). The formulae for the corrections of NNLO are available also in this case [111]—the poor knowledge of the effective coupling constants, particularly of those that are relevant for the dependence on the quark masses, is currently the limiting factor encountered in the application of these formulae.

As was to be expected, the central value of $Q$ obtained from $\eta$-decay agrees exactly with the central value obtained from the low-energy theorem: we have used that theorem to estimate the coefficient $\epsilon$, which dominates the e.m. corrections. Using the numbers for $\epsilon _{m}$, $\epsilon _{\pi ^0}$ and $\epsilon _{K^0}$ in (9) and adding the corresponding uncertainties in quadrature to those in the phenomenological result for $Q$, we obtain

31 $$\begin{aligned} \epsilon \mathop {=}\limits ^{\mathrm{NLO}}0.70(28).\end{aligned}$$

The estimate (9) for the size of the coefficient $\epsilon$ is taken from here, as it is confirmed by the most recent, preliminary lattice determinations [40–45].

Our final results for the masses $m_{u}$, $m_{d}$, $m_{ud}$, $m_{s}$ and the mass ratios $m_{u}/m_{d}$, $m_{s}/m_{ud}$, $R$, $Q$ are collected in Tables 6 and 7. We separate $m_{u}$, $m_{d}$, $m_{u}/m_{d}$, $R$ and $Q$ from $m_{ud}$, $m_{s}$ and $m_{s}/m_{ud}$, because the latter are completely dominated by lattice results while the former still include some phenomenological input.

**Table 6:** Our estimates for the strange and the average up-down quark masses in the ${\overline{\mathrm{MS}}}$ scheme at running scale $\mu =2\,\mathrm{GeV}$ for $N_\mathrm{f}=3$. Numerical values are given in MeV. In the results presented here, the first error is the one which we obtain by applying the averaging procedure of Sect. 2.2 to the relevant lattice results. We have added an uncertainty to the $N_\mathrm{f}=2+1$ results, which is associated with the neglect of heavy sea-quark and isospin-breaking effects, as discussed around (14) and (18). This uncertainty is not included in the $N_\mathrm{f}=2$ results, as it should be smaller than the uncontrolled systematic associated with the neglect of strange sea-quark effects which we choose not to estimate, as it cannot be done so reliably

$N_\mathrm{f}$	$m_{ud}$	$m_{s}$	$m_{s}/m_{ud}$
$2+1$	3.42 (6) (7)	93.8 (1.5) (1.9)	27.46 (15) (41)
2	3.6 (2)	101 (3)	28.1 (1.2)

**Table 7:** Our estimates for the masses of the two lightest quarks and related, strong isospin-breaking ratios. Again, the masses refer to the ${\overline{\mathrm{MS}}}$ scheme at running scale $\mu =2\,\mathrm{GeV}$ for $N_\mathrm{f}=3$ and the numerical values are given in MeV. In the results presented here, the first error is the one that comes from lattice computations while the second for $N_\mathrm{f}=2+1$ is associated with the phenomenological estimate of e.m. contributions, as discussed after (23). The second error on the $N_\mathrm{f}=2$ results for $R$ and $Q$ is also an estimate of the e.m. uncertainty, this time associated with the lattice computation of [45], as explained after (27). We present these results in a separate table, because they are less firmly established than those in Table 6. For $N_\mathrm{f}=2+1$ they still include information coming from phenomenology, in particular on e.m. corrections, and for $N_\mathrm{f}=2$ the e.m. contributions are computed neglecting the feedback of sea-quarks on the photon field

$$N_\mathrm{f}$$	$$m_{u} $$	$$m_{d} $$	$$m_{u}/m_{d}$$	$$R$$	$$Q$$
$2+1$	2.16 (9) (7)	4.68 (14) (7)	0.46 (2) (2)	35.8 (1.9) (1.8)	22.6 (7) (6)
2	2.40 (23)	4.80 (23)	0.50 (4)	40.7 (3.7) (2.2)	24.3 (1.4) (0.6)

## Leptonic and semileptonic kaon and pion decay and $|V_{ud}|$ and $|V_{us}|$

This section summarises state of the art lattice calculations of the leptonic kaon and pion decay constants and the kaon semileptonic decay form factor and provides an analysis in view of the Standard Model. With respect to the previous edition of the FLAG review [1] the data in this section have been updated, correlations of lattice data are now taken into account in all the analysis and a subsection on the individual decay constants $f_K$ and $f_\pi$ (rather than only the ratio) has been included. Furthermore, when combining lattice data with experimental results we now take into account the strong SU(2) isospin correction in chiral perturbation theory for the ratio of leptonic decay constants $f_K/f_\pi$.

### Experimental information concerning $|V_{ud}|$, $|V_{us}|$, $f_+(0)$ and ${f_{K^\pm }}/{f_{\pi ^\pm }}$

The following review relies on the fact that precision experimental data on kaon decays very accurately determine the product $|V_{us}|f_+(0)$ and the ratio $|V_{us}/V_{ud}|f_{K^\pm }/f_{\pi ^\pm }$ [112]:

32 $$\begin{aligned} |V_{us}| f_+(0) = 0.2163(5),\quad \left| \frac{V_{us}}{V_{ud}}\right| \frac{ f_{K^\pm }}{ f_{\pi ^\pm }} =0.2758(5).\end{aligned}$$

Here and in the following $f_{K^\pm }$ and $f_{\pi ^\pm }$ are the isospin-broken decay constants, respectively, in QCD (the electromagnetic effects have already been subtracted in the experimental analysis using chiral perturbation theory). We will refer to the decay constants in the SU(2) isospin-symmetric limit as $f_{K}$ and $f_{\pi }$. $|V_{ud}|$ and $|V_{us}|$ are elements of the Cabibbo–Kobayashi–Maskawa matrix and $f_+(t)$ represents one of the form factors relevant for the semileptonic decay $K^0\rightarrow \pi ^-\ell \,\nu$, which depends on the momentum transfer $t$ between the two mesons. What matters here is the value at $t=0$: $f_+(0)\equiv f_+^{K^0\pi ^-}(t)\,{}_{\;t\rightarrow 0}$. The pion and kaon decay constants are defined by^9^

$$\begin{aligned} {\langle }0|\,\bar{d}\gamma _\mu \gamma _5 u|\pi ^+(p)\rangle =i p_\mu f_{\pi ^+},\quad {\langle }0|\,\bar{s}\gamma _\mu \gamma _5 u|K^+(p)\rangle =i p_\mu f_{K^+}.\end{aligned}$$

In this normalisation, $f_{\pi ^\pm } \simeq 130$ MeV, $f_{K^\pm }\simeq 155$ MeV.

The measurement of $|V_{ud}|$ based on superallowed nuclear $\beta$ transitions has now become remarkably precise. The result of the update of Hardy and Towner [115], which is based on 20 different superallowed transitions, reads^10^

33 $$\begin{aligned} |V_{ud}| = 0.97425(22).\end{aligned}$$

The matrix element $|V_{us}|$ can be determined from semiinclusive $\tau$ decays [122–125]. Separating the inclusive decay $\tau \rightarrow \hbox {hadrons}+\nu$ into non-strange and strange final states, e.g. HFAG 12 [126] obtain

34 $$\begin{aligned} |V_{us}|=0.2173(22) .\end{aligned}$$

Maltman et al. [124, 127, 128] and Gamiz et al. [129, 130] arrive at very similar values.

In principle, $\tau$ decay offers a clean measurement of $|V_{us}|$, but a number of open issues yet remain to be clarified. In particular, the value of $|V_{us}|$ as determined from inclusive $\tau$ decays differs from the result one obtains from assuming three-flavour SM-unitarity by more than three standard deviations [126]. It is important to understand this apparent tension better. The most interesting possibility is that $\tau$ decay involves new physics, but more work both on the theoretical (see e.g. [131–134]) and experimental side is required.

The experimental results in Eq. (32) are for the semileptonic decay of a neutral kaon into a negatively charged pion and the charged pion and kaon leptonic decays, respectively, in QCD. In the case of the semileptonic decays the corrections for strong and electromagnetic isospin breaking in chiral perturbation theory at NLO have allowed for averaging the different experimentally measured isospin channels [112]. This is quite a convenient procedure as long as lattice QCD does not include strong or QED isospin-breaking effects. Lattice results for $f_K/f_\pi$ are typically quoted for QCD with (squared) pion and kaon masses of $M_\pi ^2=M_{\pi ^0}^2$ and $M_K^2=\frac{1}{2} (M_{K^\pm }^2+M_{K^0}^2-M_{\pi ^\pm }^2+M_{\pi ^0}^2)$ for which the leading strong and electromagnetic isospin violations cancel. While progress is being made for including strong and electromagnetic isospin breaking in the simulations (e.g. [19, 86, 105, 135–137]), for now contact to experimental results is made by correcting leading SU(2) isospin breaking guided by chiral perturbation theory.

In the following we will start by presenting the lattice results for isospin-symmetric QCD. For any Standard Model analysis based on these results we then utilise chiral perturbation theory to correct for the leading isospin-breaking effects.

### Lattice results for $f_+(0)$ and $f_K/f_\pi$

The traditional way of determining $|V_{us}|$ relies on using theory for the value of $f_+(0)$, invoking the Ademollo–Gatto theorem [150]. Since this theorem only holds to leading order of the expansion in powers of $m_{u}$, $m_{d}$ and $m_{s}$, theoretical models are used to estimate the corrections. Lattice methods have now reached the stage where quantities like $f_+(0)$ or $f_K/f_\pi$ can be determined to good accuracy. As a consequence, the uncertainties inherent in the theoretical estimates for the higher-order effects in the value of $f_+(0)$ do not represent a limiting factor any more and we shall therefore not invoke those estimates. Also, we will use the experimental results based on nuclear $\beta$ decay and $\tau$ decay exclusively for comparison—the main aim of the present review is to assess the information gathered with lattice methods and to use it for testing the consistency of the SM and its potential to provide constraints for its extensions.

The data base underlying the present review of the semileptonic form factor and the ratio of decay constants is listed in Tables 8 and 9. The properties of the lattice data play a crucial role for the conclusions to be drawn from these results: range of $M_\pi$, size of $L M_\pi$, continuum extrapolation, extrapolation in the quark masses, finite-size effects, etc. The key features of the various data sets are characterised by means of the colour code specified in Sect. 2.1. More detailed information on individual computations are compiled in Appendix B.2.

**Table 8:** Colour code for the data on $f_+(0)$

Collaboration	Ref.	$N_\mathrm{f}$	Publication status	Chiral extrapolation	Continuum extrapolation	Finite volume errors	$f_+ (0)$
FNAL/MILC 13C	[138]	$2+1+1$	C				0.9704 (24) (32)
RBC/UKQCD 13	[139]	$2+1$	A				0.9670 (20) (${}^{+18}_{-46}$)
FNAL/MILC 12	[140]	$2+1$	A				0.9667 (23) (33)
JLQCD 12	[141]	$2+1$	C				0.959 (6) (5)
JLQCD 11	[142]	$2+1$	C				0.964 (6)
RBC/UKQCD 10	[143]	$2+1$	A				0.9599 (34) (${}^{+31}_{-47}$) (14)
RBC/UKQCD 07	[144]	$2+1$	A				0.9644 (33) (34) (14)
ETM 10D	[145]	2	C				0.9544 (68)${}_\mathrm{stat}$
ETM 09A	[146]	2	A				0.9560 (57) (62)
QCDSF 07	[147]	2	C				0.9647 (15)${}_\mathrm{stat}$
RBC 06	[148]	2	A				0.968 (9) (6)
JLQCD 05	[149]	2	C				0.967 (6), 0.952 (6)

**Table 9:** Colour code for the data on the ratio of decay constants: $f_K/f_\pi$ is the pure QCD SU(2)-symmetric ratio and $f_{K^\pm }/f_{\pi ^\pm }$ is in pure QCD with the SU(2) isospin breaking applied after simulation

Collaboration	Ref.	$N_\mathrm{f}$	Publication status	Chiral extrapolation	Continuum extrapolation	Finite-volume errors	$f_K/f_\pi$	$f_{K^\pm }/f_{\pi ^\pm }$
ETM 13F	[155]	$2+1+1$	C				1.193 (13) (10)	1.183 (14) (10)
HPQCD 13A	[156]	$2+1+1$	A					1.1916 (15) (16)
MILC 13A	[157]	$2+1+1$	A					1.1947 (26) (37)
MILC 11	[24]	$2+1+1$	C					1.1872 (42)$^\dagger _\mathrm{stat.}$
ETM 10E	[158]	$2+1+1$	C				1.224 (13)$_\mathrm{stat}$	
RBC/UKQCD 12	[25]	$2+1$	A				1.199 (12) (14)	
Laiho 11	[77]	$2+1$	C					$1.202\, (11) (9) (2) (5)^{\dagger \dagger }$
MILC 10	[159]	$2+1$	C					1.197 (2) ($^{+3}_{-7}$)
JLQCD/TWQCD 10	[160]	$2+1$	C				1.230 (19)	
RBC/UKQCD 10A	[78]	$2+1$	A				1.204 (7) (25)	
PACS-CS 09	[20]	$2+1$	A				1.333 (72)	
BMW 10	[161]	$2+1$	A				1.192 (7) (6)	
JLQCD/TWQCD 09A	[162]	$2+1$	C				$1.210\, (12)_\mathrm{stat}$	
MILC 09A	[37]	$2+1$	C					1.198 (2) ($^{+6}_{-8}$)
MILC 09	[15]	$2+1$	A					1.197 (3) ($^{\;+6}_{-13}$)
Aubin 08	[163]	$2+1$	C					1.191 (16) (17)
PACS-CS 08, 08A	[19, 164]	$2+1$	A				1.189 (20)	
RBC/UKQCD 08	[79]	$2+1$	A				1.205 (18) (62)	
HPQCD/UKQCD 07	[165]	$2+1$	A				1.189 (2) (7)	
NPLQCD 06	[166]	$2+1$	A				1.218 (2) ($^{+11}_{-24}$)	
MILC 04	[36]	$2+1$	A					1.210 (4) (13)
ALPHA 13	[167]	2	C				1.1874 (57) (30)	
BGR 11	[168]	2	A				1.215 (41)	
ETM 10D	[145]	2	C				1.190 (8)$_\mathrm{stat}$	
ETM 09	[169]	2	A				1.210 (6) (15) (9)	
QCDSF/UKQCD 07	[170]	2	C				1.21 (3)	

$^\dagger$ Result with statistical error only from polynomial interpolation to the physical point

$^{\dagger \dagger }$ This work is the continuation of Aubin 08

The quantity $f_+(0)$ represents a matrix element of a strangeness changing null plane charge, $f_+(0)\!=\!(K|Q^{us}|\pi )$. The vector charges obey the commutation relations of the Lie algebra of SU(3), in particular $[Q^{us},Q^{su}]=Q^{{uu}-\mathrm{ss}}$. This relation implies the sum rule $\sum _n |(K|Q^{us}|n)|^2-\sum _n |(K|Q^{su}|n)|^2=1$. Since the contribution from the one-pion intermediate state to the first sum is given by $f_+(0)^2$, the relation amounts to an exact representation for this quantity [151]:

35 $$\begin{aligned} f_+(0)^2=1-\sum _{n\ne \pi } |(K|Q^{us}|n)|^2+\sum _n |(K|Q^{su}|n)|^2.\end{aligned}$$

While the first sum on the right extends over non-strange intermediate states, the second runs over exotic states with strangeness $\pm 2$ and is expected to be small compared to the first.

The expansion of $f_+(0)$ in SU(3) chiral perturbation theory in powers of $m_{u}$, $m_{d}$ and $m_{s}$ starts with $f_+(0)=1+f_2+f_4+\cdots \,$ [56]. Since all of the low energy constants occurring in $f_2$ can be expressed in terms of $M_\pi$, $M_K$, $M_\eta$ and $f_\pi$ [152], the NLO correction is known. In the language of the sum rule (35), $f_2$ stems from non-strange intermediate states with three mesons. Like all other non-exotic intermediate states, it lowers the value of $f_+(0)$: $f_2=-0.023$ when using the experimental value of $f_\pi$ as input. The corresponding expressions have also been derived in quenched or partially quenched (staggered) chiral perturbation theory [140, 153]. At the same order in the SU(2) expansion [154], $f_+(0)$ is parameterised in terms of $M_\pi$ and two a priori unknown parameters. The latter can be determined from the dependence of the lattice results on the masses of the quarks. Note that any calculation that relies on the $\chi$PT formula for $f_2$ is subject to the uncertainties inherent in NLO results: instead of using the physical value of the pion decay constant $f_\pi$, one may, for instance, work with the constant $f_0$ that occurs in the effective Lagrangian and represents the value of $f_\pi$ in the chiral limit. Although trading $f_\pi$ for $f_0$ in the expression for the NLO term affects the result only at NNLO, it may make a significant numerical difference in calculations where the latter are not explicitly accounted for (the lattice results concerning the value of the ratio $f_\pi /f_0$ are reviewed in Sect. 5.2).

**Fig. 4:**
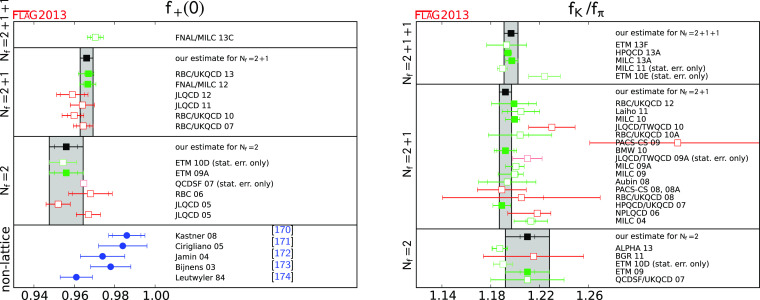
Comparison of lattice results (*squares*) for $f_+(0)$ and $f_K/ f_\pi$ with various model estimates based on $\chi$PT (*blue circles*). The *black squares* and *grey bands* indicate our estimates. The significance of the colours is explained in Sect. 2

The lattice results shown in the left panel of Fig. 4 indicate that the higher-order contributions $\Delta f\equiv f_+(0)-1-f_2$ are negative and thus amplify the effect generated by $f_2$. This confirms the expectation that the exotic contributions are small. The entries in the lower part of the left panel represent various model estimates for $f_4$. In [175] the symmetry-breaking effects are estimated in the framework of the quark model. The more recent calculations are more sophisticated, as they make use of the known explicit expression for the $K_{\ell 3}$ form factors to NNLO in $\chi$PT [174, 176]. The corresponding formula for $f_4$ accounts for the chiral logarithms occurring at NNLO and is not subject to the ambiguity mentioned above.^11^ The numerical result, however, depends on the model used to estimate the low-energy constants occurring in $f_4$ [171–174]. The figure indicates that the most recent numbers obtained in this way correspond to a positive rather than a negative value for $\Delta f$. We note that FNAL/MILC 12 [140] have made an attempt at determining some of the low-energy constants appearing in $f_4$ from lattice data.

### Direct determination of $f_+(0)$ and $f_{K^\pm }/f_{\pi ^\pm }$

All lattice results for the form factor and the ratio of decay constants that we summarise here (Tables 8, 9) have been computed in isospin-symmetric QCD. The reason for this unphysical parameter choice is that simulations of SU(2) isospin-breaking effects in lattice QCD, while ultimately the cleanest way for predicting these effects, are still rare and in their infancy [32, 33, 40, 43, 105, 136, 137]. In the meantime one relies either on chiral perturbation theory [36, 56] to estimate the correction to the isospin limit or one calculates the breaking at leading order in $(m_{u}-m_{d})$ in the valence quark sector by making a suitable choice of the physical point to which the lattice data are extrapolated. Aubin 08, MILC and Laiho 11 for example extrapolate their simulation results for the kaon decay constant to the physical value of the $up$-quark mass (the results for the pion decay constant are extrapolated to the value of the average light-quark mass $\hat{m}$). This then defines their prediction for $f_{K^\pm }/f_{\pi ^\pm }$.

As long as the majority of collaborations present their final results in the isospin-symmetric limit (as we will see this comprises the majority of results which qualify for inclusion into a FLAG average) we prefer to provide the overview of world data in Fig. 4 in this limit.

To this end we compute the isospin-symmetric ratio $f_{K}/f_{\pi }$ for Aubin 08, MILC and Laiho 11 using NLO chiral perturbation theory [56, 177] where

36 $$\begin{aligned} \frac{f_K}{f_\pi }=\frac{1}{\sqrt{\delta _\mathrm{SU}(2)+1}} \frac{f_{K^\pm }}{f_{\pi ^\pm }}, \end{aligned}$$

and where [177],

37 $$\begin{aligned} \delta _\mathrm{SU(2)}&\approx \sqrt{3}\,\epsilon _\mathrm{SU(2)} \left[ -\frac{4}{3} \left( f_{K^\pm }/f_{\pi ^\pm }-1\right) \right. \nonumber \\&\left. +\,\frac{2}{3 (4\pi )^2 f_0^2} \left( M_K^2-M_\pi ^2-M_\pi ^2\ln \frac{M_K^2}{M_\pi ^2}\right) \right] . \end{aligned}$$

We use as input $\epsilon _\mathrm{SU(2)}=\sqrt{3}/4/R$ with the FLAG result for $R$ of Eq. (28), $F_0=f_0/\sqrt{2}=80(20)$ MeV, $M_\pi =135$ MeV and $M_K=495$ MeV (we decided to choose a conservative uncertainty on $f_0$ in order to reflect the magnitude of potential higher-order corrections) and obtain for example

**Table:** 

	$f_{K^\pm }/f_{\pi ^\pm }$	$\delta _\mathrm{SU(2)}$	$f_K/f_\pi$
Aubin 08	1.202(11)(9)(2)(5)	$-$0.0044(8)	1.205(11)(2)(9)(2)(5)
MILC 10	1.197(2)($^{+3}_{-7}$)	$-$0.0043(7)	1.200(2)(2)($^{+3}_{-7}$)
Laiho 11	1.191(16)(17)	$-$0.0041(9)	1.193(16)(2)(17)

(and similarly also for all other $N_\mathrm{f}=2+1$ and $N_\mathrm{f}=2+1+1$ results where applicable). In the last column the first error is statistical and the second is the one from the isospin correction (the remaining errors are quoted in the same order as in the original data). For $N_\mathrm{f}=2$ a dedicated study of the strong-isospin correction in lattice QCD does exist. The result of the RM123 collaboration [105] amounts to $\delta _\mathrm{SU(2)}=-0.0078(7)$ and we will later use this result for the correction in the case of $N_\mathrm{f}=2$. We note that this value for the strong-isospin correction is incompatible with the above results based on SU(3) chiral perturbation theory. One would not expect the strange sea-quark contribution to be responsible for such a large effect. Whether higher-order effects in chiral perturbation theory or other sources are responsible still needs to be understood. To remain on the conservative side we attach the difference between the two- and three-flavour result as an additional uncertainty to the result based on chiral perturbation theory. For the further analysis we add both errors in quadrature.

The plots in Fig. 4 illustrate our compilation of data for $f_+(0)$ and $f_K/f_\pi$. In both cases the lattice data are largely consistent even when comparing simulations with different $N_\mathrm{f}$. We now proceed to form the corresponding averages, separately for the data with $N_\mathrm{f}=2+1+1$, $N_\mathrm{f}=2+1$ and $N_\mathrm{f}=2$ dynamical flavours and in the following will refer to these averages as the “direct” determinations.

For $f_+(0)$ there are currently two computational strategies: FNAL/MILC 12 and FNAL/MILC 13 use the Ward identity relating the $K\rightarrow \pi$ form factor at zero momentum transfer to the matrix element ${\langle }\pi |S|K\rangle$ of the flavour-changing scalar current. Peculiarities of the staggered fermion discretisation (see [140]) which FNAL/MILC is using makes this the favoured choice. The other collaborations are instead computing the vector-current matrix element ${\langle }\pi |V_\mu |K\rangle$. Apart from MILC 13C all simulations in Table 8 involve unphysically heavy quarks and therefore the lattice data need to be extrapolated to the physical pion and kaon masses corresponding to the $K^0\rightarrow \pi ^-$ channel. We note that all state of the art computations of $f_+(0)$ are using partially twisted boundary conditions which allow one to determine the form factor results directly at the relevant kinematical point $q^2=0$ [178, 179].

The colour code in Table 8 shows that for $f_+(0)$, presently only the result of ETM (we will be using ETM 09A [146]) with $N_\mathrm{f}=2$ and the results by the FNAL/MILC and RBC/UKQCD collaborations with $N_\mathrm{f}=2+1$ dynamical flavours of fermions, respectively, are without a red tag. The latter two results, $f_+(0) =0.9670(20)(^{+18}_{-46})$ (RBC/UKQCD 13) and $f_+(0) =0.9667(23)(33)$ (FNAL/MILC 12), agree very well. This is nice to observe given that the two collaborations are using different fermion discretisations (staggered fermions in the case of FNAL/MILC and domain-wall fermions in the case of RBC/UKQCD). Moreover, in the case of FNAL/MILC the form factor has been determined from the scalar-current matrix element while in the case of RBC/UKQCD it has been determined from the matrix element of the vector current. To a certain extent both simulations are expected to be affected by different systematic effects.

The result FNAL/MILC 12 is from simulations reaching down to a lightest RMS pion mass of about 380 MeV (the lightest valence pion mass for one of their ensembles is about 260 MeV). Their combined chiral and continuum extrapolation (results for two lattice spacings) is based on NLO staggered chiral perturbation theory supplemented by the continuum NNLO expression [174] and a phenomenological parameterisation of the breaking of the Ademollo–Gatto theorem at finite lattice spacing inherent in their approach. The $p^4$ low-energy constants entering the NNLO expression have been fixed in terms of external input [57].

RBC/UKQCD 13 has analysed results on ensembles with pion masses down to 170MeV, mapping out nearly the complete range from the SU(3)-symmetric limit to the physical point. Although no finite volume or cutoff effects were observed in the simulation results, the expected residual systematic effects for finite-volume effects in NLO chiral perturbation theory and an order of magnitude estimate for cutoff effects were included into the overall error budget. The dominant systematic uncertainty is the one due to the extrapolation in the light quark mass to the physical point which RBC/UKQCD did with the help of a model motivated and partly based on chiral perturbation theory. The model dependence is estimated by comparing different ansätze for the mass extrapolation.

The ETM collaboration which uses the twisted-mass discretisation provides a comprehensive study of the systematics by presenting results for three lattice spacings [180] and simulating at light pion masses (down to $M_\pi =260$ MeV). This allows one to constrain the chiral extrapolation, using both SU(3) [152] and SU(2) [154] chiral perturbation theory. Moreover, a rough estimate for the size of the effects due to quenching the strange quark is given, based on the comparison of the result for $N_\mathrm{f}=2$ dynamical quark flavours [169] with the one in the quenched approximation, obtained earlier by the SPQcdR collaboration [181]. We note for completeness that ETM extrapolate their lattice results to the point corresponding to $M_K^2$ and $M_\pi ^2$ as defined at the end of Sect. 4.1. At the current level of precision though this is expected to be a tiny effect.

We now compute the $N_\mathrm{f} =2+1$ FLAG-average for $f_+(0)$ based on FNAL/MILC 13 and RBC/UKQCD 12, which we consider uncorrelated, and for $N_\mathrm{f}=2$ the only result fulfilling the FLAG criteria is ETM 09A,

38 $$\begin{aligned} f_+(0)= 0.9661(32), \quad (\hbox {direct},\,N_\mathrm{f}=2+1), \nonumber \\ f_+(0)= 0.9560(57)(62), \quad (\hbox {direct},\,N_\mathrm{f}=2). \end{aligned}$$

The brackets in the second line indicate the statistical and systematic errors, respectively. The dominant source of systematic uncertainty in these simulations of $f_+(0)$, the chiral extrapolation, will soon be removed by simulations with physical light quark masses (see FNAL/MILC 13C [138] and RBC/UKQCD [182])

In the case of the ratio of decay constants the data sets that meet the criteria formulated in the introduction are MILC 13A [157] and HPQCD 13A [156] with $N_\mathrm{f}=2+1+1$, MILC 10 [159], BMW 10 [161], HPQCD/UKQCD 07 [165] and RBC/UKQCD 12 [25] (which is an update of RBC/UKQCD 10A [78]) with $N_\mathrm{f}=2+1$ and ETM 09 [169] with $N_\mathrm{f}=2$ dynamical flavours.

MILC 13A have determined the ratio of decay constants from a comprehensive set of ensembles of Highly Improved Staggered Quarks (HISQ) which have been taylored to reduce staggered taste-breaking effects. They have generated ensembles for four values of the lattice spacing (0.06–0.15 fm, scale set with $f_\pi$) and with the Goldstone pion masses approximately tuned to the physical point which at least on their finest lattice approximately agrees with the RMS pion mass (i.e. the difference in mass between different *pion species* which originates from staggered taste splitting). Supplementary simulations with slightly heavier Goldstone pion mass allow one to extract the ratio of decay constants for the physical value of the light-quark masses by means of polynomial interpolations. In a second step MILC extrapolates the data to the continuum limit where eventually the ratio ${f_{K^\pm }}/{f_{\pi ^\pm }}$ is extracted. The final result of their analysis is ${f_{K^\pm }}/{f_{\pi ^\pm }}=1.1947(26)(33)(17)(2)$ where the errors are statistical, due to the continuum extrapolation, due to finite volume effects and due to electromagnetic effects. MILC has found an increase in the central value of the ratio when going from the second finest to their finest ensemble and from this observation they derive the quoted 0.28 % uncertainty in the continuum extrapolation. They use NLO staggered chiral perturbation theory to correct for finite-volume effects and estimate the uncertainty in this approach by comparing to the alternative correction in NLO and NNLO continuum chiral perturbation theory. Although MILC and HPQCD are independent collaborations, MILC shares its gauge-field ensembles with HPQCD 13A, whose study of ${f_{K^\pm }}/{f_{\pi ^\pm }}$ is therefore based on the same set of ensembles bar the one for the finest lattice spacing ($a=$ 0.09–0.15 fm, scale set with $f_{\pi ^+}$ and relative scale set with the Wilson flow [183, 184]) supplemented by some simulation points with heavier quark masses. HPQCD employed a global fit based on continuum NLO SU(3) chiral perturbation theory for the decay constants supplemented by a model for higher-order terms including discretisation and finite-volume effects (61 parameters for 39 data points supplemented by Bayesian priors). Their final result is $f_{K^\pm }/f_{\pi ^\pm }=1.1916(15)(12)(1)(10)$, where the errors are statistical, due to the continuum extrapolation, due to finite-volume effects and the last error contains the combined uncertainties from the chiral extrapolation, the scale-setting uncertainty, the experimental input in terms of $f_{\pi ^+}$ and from the uncertainty in $m_{u}/m_{d}$.

Despite the large overlap in primary lattice data both collaborations arrive at surprisingly different error budgets. In the preparation of this report we interacted with both collaborations trying to understand the origin of the differences. HPQCD is using a rather new method to set the relative lattice scale for their ensembles which together with their more aggressive binning of the statistical samples, could explain the reduction in statistical error by a factor of 1.7 compared to MILC. Concerning the cutoff dependence, the finest lattice included into MILC’s analysis is $a=0.06$ fm while the finest lattice in HPQCD’s case is $a=0.09$ fm. MILC estimates the residual systematic after extrapolating to the continuum limit by taking the split between the result of an extrapolation with up to quartic and only up to quadratic terms in $a$ as their systematic. HPQCD on the other hand models cutoff effects within their global fit ansatz up to including terms in $a^8$. In this way HPQCD arrives at a systematic error due to the continuum limit which is smaller than MILC’s estimate by about a factor 2.8. HPQCD explains^12^ that in their setup, despite lacking the information from the fine ensemble ($a=0.06$ fm), the approach to the continuum limit is reliably described by the chosen fit formula leaving no room for the shift in the result on the finest lattice observed by MILC. They further explain that their different way of setting the relative lattice scale leads to reduced cutoff effects compared to MILC’s study. We now turn to finite-volume effects which in the MILC result is the second-largest source of systematic uncertainty. NLO staggered chiral perturbation theory (MILC) or continuum chiral perturbation theory (HPQCD) was used for correcting the lattice data towards the infinite-volume limit. MILC then compared the finite-volume correction to the one obtained by the NNLO expression and took the difference as their estimate for the residual finite-volume error. In addition they checked the compatibility of the effective theory predictions (NLO continuum, staggered and NNLO continuum chiral perturbation theory) against lattice data of different spatial extent. The final verdict on the related residual systematic uncertainty on ${f_{K^\pm }}/{f_{\pi ^\pm }}$ made by MILC is larger by an order of magnitude than the one made by HPQCD. We note that only HPQCD allows for taste-breaking terms in their fit model while MILC postpones such studies to future work.

The above comparison shows that MILC and HPQCD have studied similar sources of systematic uncertainties, e.g. by varying parts of the analysis procedure or by changing the functional form of a given fit ansatz. One observation worth mentioning in this context is the way in which the resulting variations in the fit result are treated. MILC tends to include the spread in central values from different ansätze into the systematic errors. HPQCD on the other hand determines the final result and attached errors from preferred fit-ansatz and then confirms that it agrees within errors with results from other ansätze without including the spreads into their error budget. In this way HPQCD is lifting the calculation of ${f_{K^\pm }}/{f_{\pi ^\pm }}$ to a new level of precision. FLAG is looking forward to independent confirmations of the result for ${f_{K^\pm }}/{f_{\pi ^\pm }}$ at the same level of precision. For now we will only provide a range for the result for $N_\mathrm{f}=2+1+1$ that covers the result of both HPQCD 13A and MILC 13A,

39 $$\begin{aligned} {f_{K^\pm }}/{f_{\pi ^\pm }}\!=\!1.194(5)\quad (\hbox {our estimate, direct, }N_\mathrm{f}\!=\!2\!+\!1\!+\!1)\nonumber \\ \end{aligned}$$

Concerning simulations with $N_\mathrm{f}\!=\!2+1$, MILC 10 and HPQCD/UKQCD 07 are based on staggered fermions, BMW 10 has used improved Wilson fermions and RBC/UKQCD 12’s result is based on the domain-wall formulation. For $N_\mathrm{f}=2$ ETM has simulated twisted-mass fermions. In contrast to MILC 13A all these latter simulations are for unphysically heavy quark masses (corresponding to smallest pion masses in the range 240–260 MeV in the case of MILC 10, HPQCD/UKQCD 07 and ETM 09 and around 170 MeV for RBC/UKQCD 12) and therefore slightly more sophisticated extrapolations needed to be controlled. Various ansätze for the mass and cutoff dependence comprising SU(2) and SU(3) chiral perturbation theory or simply polynomials were used and compared in order to estimate the model dependence.

We now provide the FLAG average for these data. While BMW 10 and RBC/UKQCD 12 are entirely independent computations, subsets of the MILC gauge ensembles used by MILC 10 and HPQCD/UKQCD 07 are the same. MILC 10 is certainly based on a larger and more advanced set of gauge configurations than HPQCD/UQKCD 07. This allows them for a more reliable estimation of systematic effects. In this situation we consider only their statistical but not their systematic uncertainties to be correlated. For $N_\mathrm{f}=2$ the FLAG average is just the result by ETM 09 and this is illustrated in terms of the vertical grey band in the r.h.s. panel of Fig. 4. For the purpose of this plot only, the isospin correction has been removed along the lines laid out earlier. For the average indicated in the case of $N_\mathrm{f}=2+1$ we take the original data of BMW 10, HPQCD/UKQCD 07 and RBC/UKQCD 12 and use the MILC 10 result as computed above. The resulting fit is of good quality, with $f_K/f_\pi =1.194(4)$ and $\chi ^2/\hbox {dof}=0.4$. The systematic errors of the individual data sets are larger for MILC 10, BMW 10, HPQCD/UKQCD 07 and RBC/UKQCD 12, respectively, and following again the prescription of Sect. 2.3 we replace the error by the smallest one of these leading to $f_K / f_\pi = 1.194(5)$ for $N_\mathrm{f}=2+1$.

Before determining the average for $f_{K^\pm }/f_{\pi ^\pm }$ which should be used for applications to Standard Model phenomenology we apply the isospin correction individually to all those results which have been published in the isospin-symmetric limit, i.e. BMW 10, HPQCD/UKQCD07 and RBC/UKQCD 12. To this end we invert Eq. (36) and use

40 $$\begin{aligned} \delta _\mathrm{SU(2)}&\approx \sqrt{3}\,\epsilon _\mathrm{SU(2)} \left[ -\frac{4}{3} (f_K/f_\pi -1)\right. \nonumber \\&\left. +\,\frac{2}{3(4\pi )^2 f_0^2}\left( M_K^2-M_\pi ^2- M_\pi ^2\ln \frac{M_K^2}{M_\pi ^2}\right) \right] . \end{aligned}$$

The results are:

**Table:** 

	$f_K/f_\pi$	$\delta _\mathrm{SU(2)}$	$f_{K^\pm }/f_{\pi ^\pm }$
HPQCD/UKQCD 07	1.189(2)(7)	$-$0.0040(7)	1.187(2) (2)(7)
BMW 10	1.192(7)(6)	$-$0.0041(7)	1.190(7) (2)(6)
RBC/UKQCD 12	1.199(12)(14)	$-$0.0043(9)	1.196(12)(2)(14)

As before, in the last column the first error is statistical and the second error is due to the isospin correction. Using these results we obtain

41 $$\begin{aligned} \begin{array}{rcll} f_{K^\pm } / f_{\pi ^\pm }&{}=&{} 1.192(5), \; &{}(\hbox {direct},\, N_\mathrm{f}=2+1),\\ f_{K^\pm } / f_{\pi ^\pm }&{}=&{} 1.205(6)(17), \; &{}(\hbox {direct},\, N_\mathrm{f}=2), \end{array} \end{aligned}$$

for QCD with broken isospin.

**Fig. 5:**
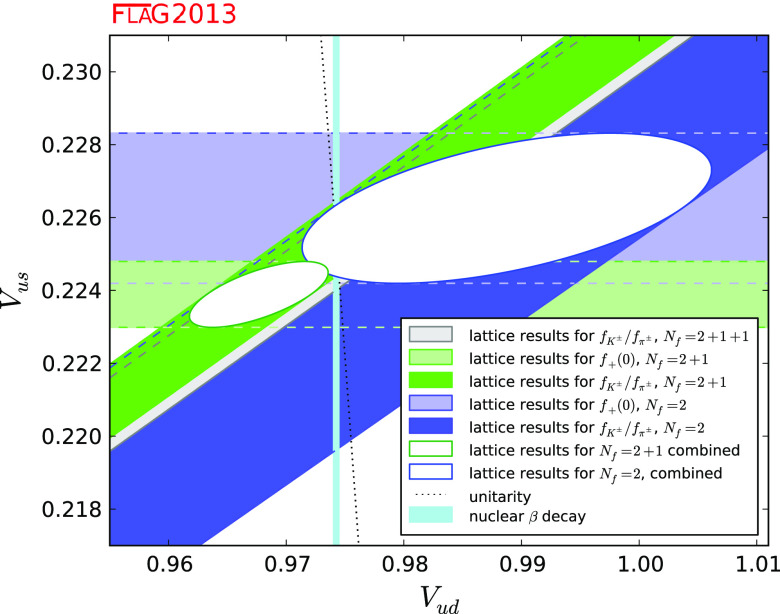
The plot compares the information for $|V_{ud}|$, $|V_{us}|$ obtained on the lattice with the experimental result extracted from nuclear $\beta$ transitions. The *dotted arc* indicates the correlation between $|V_{ud}|$ and $|V_{us}|$ that follows if the three-flavour CKM-matrix is unitary

It is instructive to convert the above results for $f_+(0)$ and ${f_{K^\pm }}/{f_{\pi ^\pm }}$ into a corresponding range for the CKM matrix elements $|V_{ud}|$ and $|V_{us}|$, using the relations (32). Consider first the results for $N_\mathrm{f}=2+1$. The range for $f_+(0)$ in (38) is mapped into the interval $|V_{us}|=0.2239(7)$, depicted as a horizontal green band in Fig. 5, while the one for ${f_{K^\pm }}/{f_{\pi ^\pm }}$ in (41) is converted into $|V_{us}|/|V_{ud}|= 0.2314(11)$, shown as a tilted green band. The smaller green ellipse is the intersection of these two bands.

More precisely, it represents the 68 % likelihood contour (note also that the ellipses shown in Fig. 5 of Ref. [1] have to be interpreted as 39 % likelihood contours), obtained by treating the above two results as independent measurements. Values of $|V_{us}|$, $|V_{ud}|$ in the region enclosed by this contour are consistent with the lattice data for $N_\mathrm{f}=2+1$, within one standard deviation. In particular, the plot shows that the nuclear $\beta$ decay result for $|V_{ud}|$ is in good agreement with these data. We note that with respect to the previous edition of the FLAG review the reanalysis including new results has moved the ellipse representing QCD with $N_\mathrm{f}=2+1$ slightly down and to the left.

Repeating the exercise for $N_\mathrm{f}=2$ leads to the larger blue ellipse. The figure indicates a slight tension between the $N_\mathrm{f}=2$ and $N_\mathrm{f}=2+1$ results, which, at the current level of precision is not visible if considering the $N_\mathrm{f}=2$ and $N_\mathrm{f}=2+1$ results for $f_+(0)$ and ${f_{K^\pm }}/{f_{\pi ^\pm }}$ in Fig. 4 on their own. It remains to be seen if this is a first indication of the effect of quenching the strange quark.

In the case of $N_\mathrm{f}=2+1+1$ only results for ${f_{K^\pm }}/{f_{\pi ^\pm }}$ are without red tags. In this case we have therefore only plotted the corresponding band for $|V_{us}|$ from $f_{K^\pm }/f_{\pi ^\pm }$ corresponding to $|V_{us}|/|V_{ud}|=0.2310(11)$.

### Testing the Standard Model

In the Standard Model, the CKM matrix is unitary. In particular, the elements of the first row obey

42 $$\begin{aligned} |V_{u}|^2\equiv |V_{ud}|^2 + |V_{us}|^2 + |V_{ub}|^2 = 1.\end{aligned}$$

The tiny contribution from $|V_{ub}|$ is known much better than needed in the present context: $|V_{ub}|= 4.15 (49) \cdot 10^{-3}$ [74]. In the following, we first discuss the evidence for the validity of the relation (42) and only then use it to analyse the lattice data within the Standard Model.

In Fig. 5, the correlation between $|V_{ud}|$ and $|V_{us}|$ imposed by the unitarity of the CKM matrix is indicated by a dotted arc (more precisely, in view of the uncertainty in $|V_{ub}|$, the correlation corresponds to a band of finite width, but the effect is too small to be seen here).

The plot shows that there is a slight tension with unitarity in the data for $N_\mathrm{f} = 2 + 1$: Numerically, the outcome for the sum of the squares of the first row of the CKM matrix reads $|V_{u}|^2 = 0.987(10)$. Still, it is fair to say that at this level the Standard Model passes a non-trivial test that exclusively involves lattice data and well-established kaon decay branching ratios. Combining the lattice results for $f_+(0)$ and ${f_{K^\pm }}/{f_{\pi ^\pm }}$ in (38) and (41) with the $\beta$ decay value of $|V_{ud}|$ quoted in (33), the test sharpens considerably: the lattice result for $f_+(0)$ leads to $|V_{u}|^2 = 0.9993(5)$, while the one for ${f_{K^\pm }}/{f_{\pi ^\pm }}$ implies $|V_{u}|^2 = 1.0000(6)$, thus confirming CKM unitarity at the permille level.

Repeating the analysis for $N_\mathrm{f} = 2$, we find $|V_{u}|^2 = 1.029(35)$ with the lattice data alone. This number is fully compatible with 1, in accordance with the fact that the dotted curve penetrates the blue contour. Taken by themselves, these results are perfectly consistent with the value of $|V_{ud}|$ found in nuclear $\beta$ decay: combining this value with the data on $f_+(0)$ yields $|V_{u}|^2=1.0004(10)$, combining it with the data on ${f_{K^\pm }}/{f_{\pi ^\pm }}$ gives $|V_{u}|^2= 0.9989(16)$. With respect to the first edition of the FLAG report the ellipse for $N_\mathrm{f}=2$ has moved slightly to the left because we have now taken into account isospin-breaking effects.

For $N_\mathrm{f}=2+1+1$ we can carry out the test of unitarity only with input from ${f_{K^\pm }}/{f_{\pi ^\pm }}$ which leads to $|V_{u}|^2=0.9998(7)$.

Note that the above tests also offer a check of the basic hypothesis that underlies our analysis: we are assuming that the weak interaction between the quarks and the leptons is governed by the same Fermi constant as the one that determines the strength of the weak interaction among the leptons and determines the lifetime of the muon. In certain modifications of the Standard Model, this is not the case. In those models it need not be true that the rates of the decays $\pi \rightarrow \ell \nu$, $K\rightarrow \ell \nu$ and $K\rightarrow \pi \ell \nu$ can be used to determine the matrix elements $|V_{ud}f_\pi |$, $|V_{us}f_K|$ and $|V_{us}f_+(0)|$, respectively and that $|V_{ud}|$ can be measured in nuclear $\beta$ decay. The fact that the lattice data are consistent with unitarity and with the value of $|V_{ud}|$ found in nuclear $\beta$ decay indirectly also checks the equality of the Fermi constants.

### Analysis within the Standard Model

The Standard Model implies that the CKM matrix is unitary. The precise experimental constraints quoted in (32) and the unitarity condition (42) then reduce the four quantities $|V_{ud}|,|V_{us}|,f_+(0), {f_{K^\pm }}/{f_{\pi ^\pm }}$ to a single unknown: any one of these determines the other three within narrow uncertainties.

**Fig. 6:**
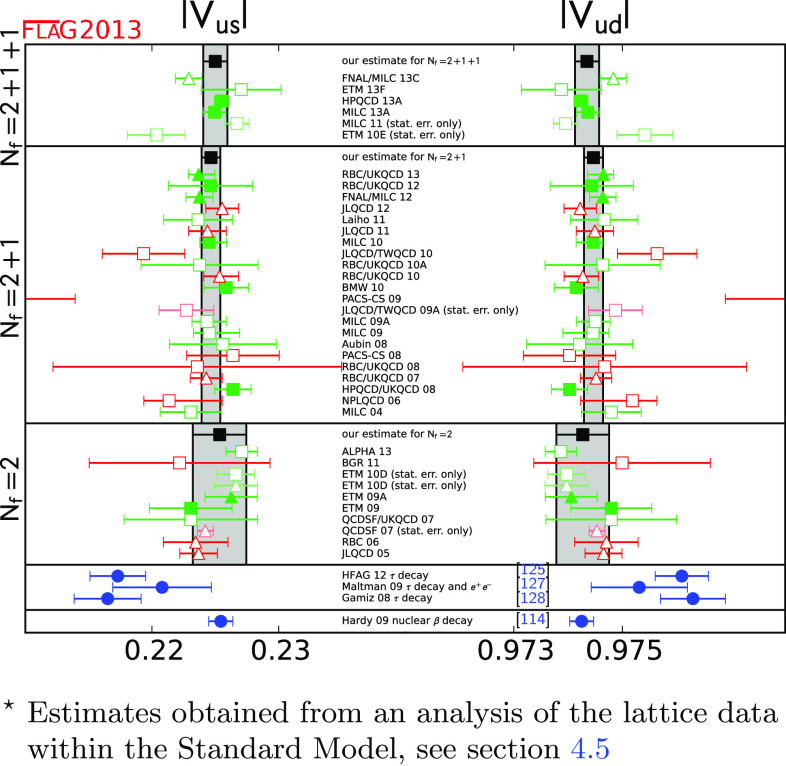
Results for $|V_{us}|$ and $|V_{ud}|$ that follow from the lattice data for $f_+(0)$ (*triangles*) and ${f_{K^\pm }}/{f_{\pi ^\pm }}$ (*squares*), on the basis of the assumption that the CKM matrix is unitary. The *black squares* and the *grey bands* represent our estimates, obtained by combining these two different ways of measuring $|V_{us}|$ and $|V_{ud}|$ on a lattice. For comparison, the figure also indicates the results obtained if the data on nuclear $\beta$ decay and $\tau$ decay are analysed within the Standard Model

Figure 6 shows that the results obtained for $|V_{us}|$ and $|V_{ud}|$ from the data on ${f_{K^\pm }}/{f_{\pi ^\pm }}$ (squares) are quite consistent with the determinations via $f_+(0)$ (triangles). In order to calculate the corresponding average values, we restrict ourselves to those determinations that we have considered best in Sect. 4.3. The corresponding results for $|V_{us}|$ are listed in Table 10 (the error in the experimental numbers used to convert the values of $f_+(0)$ and ${f_{K^\pm }}/{f_{\pi ^\pm }}$ into values for $|V_{us}|$ is included in the statistical error).

**Table 10:** Values of $|V_{us}|$ obtained from lattice determinations of $f_+(0)$ or ${f_{K^\pm }}/{f_{\pi ^\pm }}$ with CKM unitarity. The first (second) number in brackets represents the statistical (systematic) error

Collaboration	Ref.	$N_\mathrm{f}$	From	$|V_{us}|$
HPQCD 13A	[156]	$2+1+1$	${f_{K^\pm }}/{f_{\pi ^\pm }}$	0.2255 (5) (3)
MILC 13A	[157]	$2+1+1$	${f_{K^\pm }}/{f_{\pi ^\pm }}$	0.2249 (6) (7)
RBC/UKQCD 13	[139]	$2+1$	$f_+ (0)$	0.2237 (7) (7)
MILC 12	[140]	$2+1$	$f_+ (0)$	0.2238 (7) (8)
MILC 10	[159]	$2+1$	${f_{K^\pm }}/{f_{\pi ^\pm }}$	0.2249 (5) (9)
RBC/UKQCD 10A	[78]	$2+1$	${f_{K^\pm }}/{f_{\pi ^\pm }}$	0.2246 (22) (25)
BMW 10	[161]	$2+1$	${f_{K^\pm }}/{f_{\pi ^\pm }}$	0.2259 (13) (12)
HPQCD/UKQCD 07	[165]	$2+1$	${f_{K^\pm }}/{f_{\pi ^\pm }}$	0.2264 (5) (13)
ETM 09	[169]	2	${f_{K^\pm }}/{f_{\pi ^\pm }}$	0.2231 (11) (31)
ETM 09A	[146]	2	$f_+ (0)$	0.2263 (14) (15)

We consider the fact that the results from the five $N_\mathrm{f}=2+1$ data sets FNAL/MILC 12 [140], RBC/UKQCD 13 [139], RBC/UKQCD 12 [25], BMW 10 [161], MILC 10 [159] and HPQCD/UKQCD 07 [165] are consistent with each other to be an important reliability test of the lattice work. Applying the prescription of Sect. 2.3, where we consider MILC 10, FNAL/MILC 12 and HPQCD/UKQCD 07 on the one hand and RBC/UKQCD 12 and RBC/UKQCD 13 on the other hand, as mutually statistically correlated since the analysis in the two cases starts from partly the same set of gauge ensembles, we arrive at $|V_{us}| = 0.2247(7)$ with $\chi ^2/\hbox {dof}=0.8$. This result is indicated on the left hand side of Fig. 6 by the narrow vertical band. The value for $N_\mathrm{f}=2$, $|V_{us}|= 0.2253(21)$, with $\chi ^2/\hbox {dof}=0.9$, where we have considered ETM 09 and ETM 09A as statistically correlated is also indicated by a band. For $N_\mathrm{f}=2+1+1$ we only consider the data for ${f_{K^\pm }}/{f_{\pi ^\pm }}$ yielding $|V_{us}|=0.2251(10)$. The figure shows that the result obtained for the data with $N_\mathrm{f}=2$, $N_\mathrm{f}=2+1$ and $N_\mathrm{f}=2+1+1$ are perfectly consistent.

Alternatively, we can solve the relations for $|V_{ud}|$ instead of $|V_{us}|$. Again, the result $|V_{ud}|=0.97434(22)$ which follows from the lattice data with $N_\mathrm{f}=2+1+1$ is perfectly consistent with the values $|V_{ud}|=0.97447(18)$ and $|V_{ud}|=0.97427(49)$ obtained from those with $N_\mathrm{f}=2+1$ and $N_\mathrm{f}=2$, respectively. The reduction of the uncertainties in the result for $|V_{ud}|$ due to CKM unitarity is to be expected from Fig. 5: the unitarity condition reduces the region allowed by the lattice results to a nearly vertical interval.

Next, we determine the value of $f_+(0)$ that follows from the lattice data within the Standard Model. Using CKM unitarity to convert the lattice determinations of ${f_{K^\pm }}/{f_{\pi ^\pm }}$ into corresponding values for $f_+(0)$ and then combining these with the direct determinations of $f_+(0)$, we find $f_+(0)= 0.9634(32)$ from the data with $N_\mathrm{f}=2+1$ and $f_+(0)= 0.9595(90)$ for $N_\mathrm{f}=2$. In the case $N_\mathrm{f}=2+1+1$ we obtain $f_+(0)=0.9611(47)$.

Finally, we work out the analogous Standard Model fits for ${f_{K^\pm }}/{f_{\pi ^\pm }}$, converting the direct determinations of $f_+(0)$ into corresponding values for ${f_{K^\pm }}/{f_{\pi ^\pm }}$ and combining the outcome with the direct determinations of that quantity. The results read ${f_{K^\pm }}/{f_{\pi ^\pm }}=1.197(4)$ for $N_\mathrm{f}=2+1$ and ${f_{K^\pm }}/{f_{\pi ^\pm }}= 1.192(12)$ for $N_\mathrm{f}=2$, respectively.

The results obtained by analysing the lattice data in the framework of the Standard Model are collected in the upper half of Table 11. In the lower half of this table, we list the analogous results, found by working out the consequences of CKM unitarity for the experimental values of $|V_{ud}|$ and $|V_{us}|$ obtained from nuclear $\beta$ decay and $\tau$ decay, respectively. The comparison shows that the lattice result for $|V_{ud}|$ not only agrees very well with the totally independent determination based on nuclear $\beta$ transitions, but it is also remarkably precise. On the other hand, the values of $|V_{ud}|$, $f_+(0)$ and ${f_{K^\pm }}/{f_{\pi ^\pm }}$ which follow from the $\tau$ decay data if the Standard Model is assumed to be valid, are not in good agreement with the lattice results for these quantities. The disagreement is reduced considerably if the analysis of the $\tau$ data is supplemented with experimental results on electroproduction [128]: the discrepancy then amounts to little more than one standard deviation.

**Table 11:** The upper half of the table shows our final results for $|V_{us}|$, $|V_{ud}|$, $f_+(0)$ and ${f_{K^\pm }}/{f_{\pi ^\pm }}$, which are obtained by analysing the lattice data within the Standard Model. For comparison, the lower half lists the values that follow if the lattice results are replaced by the experimental results on nuclear $\beta$ decay and $\tau$ decay, respectively

	Ref.	$|V_{us}|$	$|V_{ud}|$	$f_+(0)$	${f_{K^\pm }}/{f_{\pi ^\pm }}$
$N_\mathrm{f}= 2+1+1$		0.2251 (10)	0.97434 (22)	0.9611 (47)	1.194 (5)
$N_\mathrm{f}= 2+1$		0.2247 (7)	0.97447 (18)	0.9634 (32)	1.197 (4)
$N_\mathrm{f}=2$		0.2253 (21)	0.97427 (49)	0.9595 (90)	1.192 (12)
$\beta$ decay	[115]	0.22544 (95)	0.97425 (22)	0.9595 (46)	1.1919 (57)
$\tau$ decay	[129]	0.2165 (26)	0.9763 (6)	0.999 (12)	1.244 (16)
$\tau$ decay	[128]	0.2208 (39)	0.9753 (9)	0.980 (18)	1.218 (23)

### Direct determination of $f_K$ and $f_\pi$

It is useful for flavour physics to provide not only the lattice average of $f_K / f_\pi$, but also the average of the decay constant $f_K$. Indeed, the $\Delta S = 2$ hadronic matrix element for neutral kaon mixing is generally parameterised by $M_K$, $f_K$ and the kaon bag parameter $B_K$. The knowledge of both $f_K$ and $B_K$ is therefore crucial for a precise theoretical determination of the CP-violation parameter $\epsilon _K$ and for the constraint on the apex of the CKM unitarity triangle.

The case of the decay constant $f_\pi$ is somehow different, since the experimental value of this quantity is often used for setting the scale in lattice QCD (see Appendix A.2). However, the physical scale can be set in different ways, namely by using as input the mass of the $\Omega$-baryon ($m_\Omega$) or the $\Upsilon$-meson spectrum ($\Delta M_\Upsilon$), which are less sensitive to the uncertainties of the chiral extrapolation in the light-quark mass with respect to $f_\pi$. In such cases the value of the decay constant $f_\pi$ becomes a direct prediction of the lattice QCD simulations. It is therefore interesting to provide also the average of the decay constant $f_\pi$, obtained when the physical scale is set through another hadron observable, in order to check the consistency of different scale-setting procedures.

Our compilation of the values of $f_\pi$ and $f_K$ with the corresponding colour code is presented in Table 12. With respect to the case of $f_K / f_\pi$ we have added two columns indicating which quantity is used to set the physical scale and the possible use of a renormalisation constant for the axial current. Indeed, for several lattice formulations the use of the non-singlet axial-vector Ward identity allows one to avoid the use of any renormalisation constant.

**Table 12:** Colour code for the lattice data on $f_\pi$ and $f_K$ together with information on the way the lattice spacing was converted to physical units and on whether or not an isospin-breaking correction has been applied (using chiral perturbation theory) to the quoted result. The numerical values are listed in MeV units

Collaboration	Ref.	$N_\mathrm{f}$	Publication status	Chiral extrapolation	Continuum extrapolation	Finite-volume errors	Renormalisation	Physical scale	SU(2) breaking	$f_\pi$	$f_K$
HPQCD 13A	[156]	$2+1+1$	A				na	$f_\pi$	$\checkmark$	–	155.37 (20) (28)
ETM 10E	[158]	$2+1+1$	C				na	$f_\pi$		–	160 (2)
RBC/UKQCD 12	[25]	$2+1$	A				NPR	$m_\Omega$		$127\, (3) (3)$	$152\, (3) (2)$
Laiho 11	[77]	$2+1$	C				na	$\dagger$	$\checkmark$	$130.53\, (87) (210)$	$156.8\, (1.0) (1.7)$
MILC 10	[159]	$2+1$	C				na	$\dagger$	$\checkmark$	129.2 (4) (14)	–
MILC 10	[159]	$2+1$	C				na	$f_\pi$	$\checkmark$	–	156.1 (4) ($^{+6}_{-9}$)
JLQCD/TWQCD 10	[160]	$2+1$	C				na	$m_\Omega$		118.5 (3.6)$_\mathrm{stat}$	145.8 (2.7)$_\mathrm{stat}$
RBC/UKQCD 10A	[78]	$2+1$	A				NPR	$m_\Omega$		124 (2) (5)	149 (2) (3)
PACS-CS 09	[20]	$2+1$	A				NPR	$m_\Omega$		124.6 (8.6) (0.9)	166.1 (3.4) (1.2)
JLQCD/TWQCD 09A	[162]	$2+1$	C				na	$f_\pi$		–	157.3 (5.5)$_\mathrm{stat}$
MILC 09A	[37]	$2+1$	C				na	$\Delta M_\Upsilon$	$\checkmark$	128.0 (0.3) (2.9)	153.8 (0.3) (3.9)
MILC 09A	[37]	$2+1$	C				na	$f_\pi$	$\checkmark$	–	156.2 (0.3) (1.1)
MILC 09	[15]	$2+1$	A				na	$\Delta M_\Upsilon$	$\checkmark$	128.3 (0.5) ($^{+2.4}_{-3.5}$)	154.3 (0.4) ($^{+2.1}_{-3.4}$)
MILC 09	[15]	$2+1$	A				na	$f_\pi$	$\checkmark$		156.5 (0.4) ($^{+1.0}_{-2.7}$)
Aubin 08	[163]	$2+1$	C				na	$\Delta M_\Upsilon$	$\checkmark$	129.1 (1.9) (4.0)	153.9 (1.7) (4.4)
PACS-CS 08, 08A	[19, 164]	$2+1$	A				1lp	$m_\Omega$		134.0 (4.2)$_\mathrm{stat}$	159.4 (3.1)$_\mathrm{stat}$
RBC/UKQCD 08	[79]	$2+1$	A				NPR	$m_\Omega$		124.1 (3.6) (6.9)	149.6 (3.6) (6.3)
HPQCD/UKQCD 07	[165]	$2+1$	A				na	$\Delta M_\Upsilon$		132 (2)	157 (2)
MILC 04	[36]	$2+1$	A				na	$\Delta M_\Upsilon$	$\checkmark$	129.5 (0.9) (3.5)	156.6 (1.0) (3.6)
TWQCD 11	[185]	2	P				na	$$r_0^*$$		127.3 (1.7) (2.0)$$^{**}$$	–
ETM 09	[169]	2	A				na	$f_\pi$		–	158.1 (0.8) (2.0) (1.1)$^{\dagger \dagger }$
JLQCD/TWQCD 08A	[67]	2	A				na	$$r_0$$		119.6 (3.0) ($$^{+6.5}_{-1.0}$$)$$^{**}$$	–

The label “na” indicates the lattice calculations which do not require the use of any renormalisation constant for the axial current, while the label “NPR” (“1lp”) signals the use of a renormalisation constant calculated non-perturbatively (at one-loop order in perturbation theory)

$^{\dagger }$ The ratios of lattice spacings within the ensembles were determined using the quantity $r_1$. The conversion to physical units was made basing on ref. [186] and we note that such a determination depends on the experimental value of the pion decay constant

$^{\dagger \dagger }$ Errors are (stat+chiral) ($a\ne 0$) (finite size)

$^*$ The ratio $f_\pi /M_\pi$ was used as experimental input to fix the light-quark mass

$$^{**}$$ $$L_\mathrm{min}<2$$ fm in these simulations

One can see that the determinations of $f_\pi$ and $f_K$ suffer from larger uncertainties with respect to the ones of the ratio $f_K / f_\pi$, which is less sensitive to various systematic effects (including the uncertainty of a possible renormalisation constant) and, moreover, is not so exposed to the uncertainties of the procedure used to set the physical scale.

According to the FLAG rules three data sets can form the average of $f_\pi$ and $f_K$ for $N_\mathrm{f} = 2 + 1$: RBC/UKQCD 12 [25] (update of RBC/UKQCD 10A), HPQCD/UKQCD 07 [165] and MILC 10 [159], which is the latest update of the MILC program.^13^ We consider HPQCD/UKQCD 07 and MILC 10 as statistically correlated and use the prescription of Sect. 2.3 to form an average. For $N_\mathrm{f} = 2$ the average cannot be formed for $f_\pi$, and only one data set (ETM 09) satisfies the FLAG rules in the case of $f_K$. Following the discussion around the $N_\mathrm{f}=2+1+1$ result for $f_{K^\pm }/f_{\pi ^\pm }$ we refrain from providing a FLAG-average for $f_K$ for this case.

Thus, our estimates (in the isospin-symmetric limit of QCD) read

43 $$\begin{aligned} f_\pi&= 130.2 ~ (1.4) ~ \hbox {MeV} \qquad \qquad (N_\mathrm{f} = 2 + 1),\end{aligned}$$

44 $$\begin{aligned} f_K&= 156.3 ~ (0.9) ~ \hbox {MeV} \qquad \qquad (N_\mathrm{f} = 2 + 1), \nonumber \\ f_K&= 158.1 ~ (2.5) ~ \hbox {MeV} \qquad \qquad (N_\mathrm{f} = 2). \end{aligned}$$

**Fig. 7:**
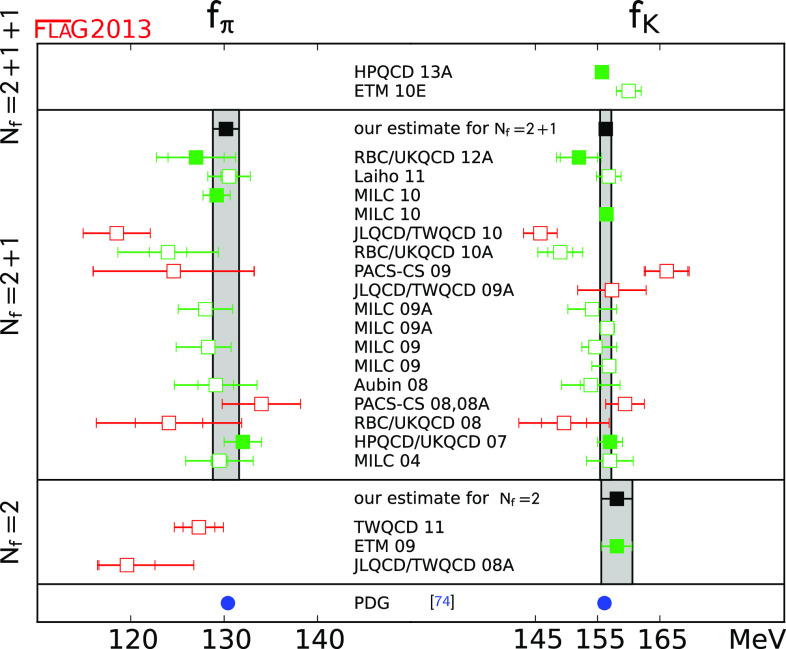
Values of $f_\pi$ and $f_K$. The *black squares* and *grey bands* indicate our estimates (43) and (44). The *blue dots* represent the experimental values quoted by the PDG (45)

The lattice results of Table 12 and our estimates (43–44) are reported in Fig. 7. The latter ones compare positively within the errors with the latest experimental determinations of $f_\pi$ and $f_K$ from the PDG:

45 $$\begin{aligned} f_\pi ^\mathrm{(PDG)}&= 130.41 ~ (0.20) ~ \hbox {MeV},\nonumber \\&f_K ^{(PDG)}= 156.1 ~ (0.8) ~ \hbox {MeV}, \end{aligned}$$

which, we recall, do not correspond, however, to pure QCD results in the isospin-symmetric limit. Moreover, the values of $f_\pi$ and $f_K$ quoted by the PDG are obtained assuming Eq. (32) for the value of $|V_{ud}|$ and adopting the RBC-UKQCD 07 result for $f_+(0)$.

## Low-energy constants

In the study of the quark-mass dependence of QCD observables calculated on the lattice it is common practice to invoke Chiral Perturbation Theory ($\chi$PT). For a given quantity this framework predicts the non-analytic quark-mass dependence and it provides symmetry relations among different observables. These relations are best expressed with the help of a set of linearly independent and universal (i.e. process-independent) low-energy constants (LECs), which appear as coefficients of the polynomial terms (in $m_{q}$ or $M_\pi ^2$) in different observables. If one expands around the SU(2) chiral limit, in the Chiral Effective Lagrangian there appear two LECs at order $p^2$

46 $$\begin{aligned}&F\equiv F_\pi \,\Big |_{m_{u},m_{d}\rightarrow 0}, \qquad B\equiv \frac{\Sigma }{F^2}\quad \hbox {where}\nonumber \\&\quad \Sigma \equiv -{\langle }\bar{u}u{\rangle }\,\Big |_{\;m_{u},m_{d}\rightarrow 0}, \end{aligned}$$

and seven at order $p^4$, indicated by $\bar{\ell }_i$ with $i=1,\ldots ,7$. In the analysis of the SU(3) chiral limit there are also just two LECs at order $p^2$

47 $$\begin{aligned}&F_0\equiv F_\pi \,\Big |_{m_{u},m_{d},m_{s}\rightarrow 0} , \qquad B_0\equiv \frac{\Sigma _0}{F_0^2}\quad \nonumber \\&\quad \hbox {where}\quad \Sigma _0\equiv -{\langle }\bar{u}u{\rangle }\,\Big |_{m_{u},m_{d},m_{s}\rightarrow 0}, \end{aligned}$$

but ten at order $p^4$, indicated by the capital letter $L_i(\mu )$ with $i=1,\ldots ,10$. These constants are independent of the quark masses^14^, but they become scale dependent after renormalisation (sometimes a superscript $r$ is added). The SU(2) constants $\bar{\ell }_i$ are scale independent, since they are defined at $\mu =M_\pi$ (as indicated by the bar). For the precise definition of these constants and their scale dependence we refer the reader to [56, 58].

First of all, lattice calculations can be used to test if chiral symmetry is indeed broken as SU$(N_\mathrm{f})_L \times$SU$(N_\mathrm{f})_R \rightarrow$SU$(N_\mathrm{f})_{L+R}$ by measuring non-zero chiral condensates and by verifying the validity of the GMOR relation $M_\pi ^2\propto m$ close to the chiral limit. If the chiral extrapolation of quantities calculated on the lattice is made with the help of $\chi$PT, apart from determining the observable at the physical value of the quark masses one also obtains the relevant LECs. This is a very important by-product for two reasons:

1. All LECs up to order $p^4$ (with the exception of $B$ and $B_0$, since only the product of these times the quark masses can be estimated from phenomenology) have either been determined by comparison to experiment or estimated theoretically. A lattice determination of the better known ones thus provides a test of the $\chi$PT approach.
2. The less well-known LECs are those which describe the quark-mass dependence of observables—these cannot be determined from experiment, and therefore the lattice provides unique quantitative information. This information is essential for improving phenomenological $\chi$PT predictions in which these LECs play a role.

We stress that this program is based on the non-obvious assumption that $\chi$PT is valid in the region of masses used in the lattice simulations under consideration.

The fact that, at large volume, the finite-size effects, which occur if a system undergoes spontaneous symmetry breakdown, are controlled by the Nambu–Goldstone modes, was first noted in solid-state physics, in connection with magnetic systems [187, 188]. As pointed out in [189] in the context of QCD, the thermal properties of such systems can be studied in a systematic and model-independent manner by means of the corresponding effective field theory, provided the temperature is low enough. While finite volumes are not of physical interest in particle physics, lattice simulations are necessarily carried out in a finite box. As shown in [190–192], the ensuing finite-size effects can also be studied on the basis of the effective theory—$\chi$PT in the case of QCD—provided the simulation is close enough to the continuum limit, the volume is sufficiently large and the explicit breaking of chiral symmetry generated by the quark masses is sufficiently small. Indeed, $\chi$PT represents also a useful tool for the analysis of the finite-size effects in lattice simulations.

In the following two subsections we summarise the lattice results for the SU(2) and SU(3) LECs, respectively. In either case we first discuss the $O(p^2)$ constants and then proceed to their $O(p^4)$ counterparts. The $O(p^2)$ LECs are determined from the chiral extrapolation of masses and decay constants or, alternatively, from a finite-size study of correlators in the $\epsilon$-regime. At order $p^4$ some LECs affect two-point functions while other appear only in three- or four-point functions; the latter need to be determined from form factors or scattering amplitudes. The $\chi$PT analysis of the (non-lattice) phenomenological quantities is nowadays^15^ based on $O(p^6)$ formulae. At this level the number of LECs explodes and we will not discuss any of these. We will, however, discuss how comparing different orders and different expansions (in particular $x$ versus $\xi$-expansion; see below) can help to assess the theoretical uncertainties of the LECs determined on the lattice.

### SU(2) low-energy constants 

#### Quark-mass dependence of pseudoscalar masses and decay constants

The expansions^16^ of $M_\pi ^2$ and $F_\pi$ in powers of the quark mass are known to next-to-next-to-leading order in the SU(2) chiral effective theory. In the isospin limit, $m_{u}=m_{d}=m$, the explicit expressions may be written in the form [193]

48 $$\begin{aligned} M_\pi ^2&= M^2 \left\{ 1-\frac{1}{2}x\ln \frac{\Lambda _3^2}{M^2} +\frac{17}{8}x^2 \left( \ln \frac{\Lambda _M^2}{M^2} \right) ^2 \right. \nonumber \\&\left. +x^2 k_M +O(x^3) \right\} ,\nonumber \\ F_\pi&= F \left\{ 1+x\ln \frac{\Lambda _4^2}{M^2} -\frac{5}{4}x^2 \left( \ln \frac{\Lambda _F^2}{M^2} \right) ^2 \right. \nonumber \\&\left. +x^2k_F +O(x^3) \right\} . \end{aligned}$$

Here the expansion parameter is given by

49 $$\begin{aligned} x=\frac{M^2}{(4\pi F)^2},\quad M^2=2Bm=\frac{2\Sigma m}{F^2}, \end{aligned}$$

but there is another option as discussed below. The scales $\Lambda _3,\Lambda _4$ are related to the effective coupling constants $\bar{\ell }_3,\bar{\ell }_4$ of the chiral Lagrangian at running scale $M_\pi \equiv M_\pi ^\mathrm{phys}$ by

50 $$\begin{aligned} \bar{\ell }_n=\ln \frac{\Lambda _n^2}{M_\pi ^2},\quad n=1,\ldots ,7. \end{aligned}$$

Note that in Eq. (48) the logarithms are evaluated at $M^2$, not at $M_\pi ^2$. The coupling constants $k_M,k_F$ in Eq. (48) are mass-independent. The scales of the squared logarithms can be expressed in terms of the $O(p^4)$ coupling constants as

51 $$\begin{aligned} \ln \frac{\Lambda _M^2}{M^2}&= \frac{1}{51}\left( 28\ln \frac{\Lambda _1^2}{M^2} +32\ln \frac{\Lambda _2^2}{M^2} -9 \ln \frac{\Lambda _3^2}{M^2}+49\right) ,\nonumber \\ \ln \frac{\Lambda _F^2}{M^2}&= \frac{1}{30} \left( 14\ln \frac{\Lambda _1^2}{M^2} +16\ln \frac{\Lambda _2^2}{M^2}+6 \ln \frac{\Lambda _3^2}{M^2} \right. \nonumber \\&\left. - 6 \ln \frac{\Lambda _4^2}{M^2}+23 \right) . \end{aligned}$$

Hence by analysing the quark-mass dependence of $M_\pi ^2$ and $F_\pi$ with Eq. (48), possibly truncated at NLO, one can determine^17^ the $O(p^2)$ LECs $B$ and $F$, as well as the $O(p^4)$ LECs $\bar{\ell }_3$ and $\bar{\ell }_4$. The quark condensate in the chiral limit is given by $\Sigma =F^2B$. With precise enough data at several small enough pion masses, one could in principle also determine $\Lambda _M$, $\Lambda _F$ and $k_M$, $k_F$. To date this is not yet possible. The results for the LO and NLO constants will be presented in Sect. 5.1.6.

Alternatively, one can invert Eq. (48) and express $M^2$ and $F$ as an expansion in

52 $$\begin{aligned} \xi \equiv \frac{M_\pi ^2}{16 \pi ^2 F_\pi ^2}, \end{aligned}$$

and the corresponding expressions then take the form

53 $$\begin{aligned}&M^2= M_\pi ^2\nonumber \\&\quad \times \left\{ 1\!+\!\frac{1}{2}\,\xi \,\ln \frac{\Lambda _3^2}{M_\pi ^2}-\frac{5}{8}\,\xi ^2 \left( \!\ln \frac{\Omega _M^2}{M_\pi ^2}\!\right) ^2\!+\! \xi ^2 c_{\scriptscriptstyle M}+O(\xi ^3)\right\} ,\nonumber \\&F= F_\pi \nonumber \\&\quad \times \left\{ 1-\xi \,\ln \frac{\Lambda _4^2}{M_\pi ^2}-\frac{1}{4}\,\xi ^2\left( \!\ln \frac{\Omega _F^2}{M_\pi ^2}\!\right) ^2 +\xi ^2 c_{\scriptscriptstyle F}+O(\xi ^3)\right\} .\nonumber \\ \end{aligned}$$

The scales of the quadratic logarithms are determined by $\Lambda _1,\ldots ,\Lambda _4$ through

54 $$\begin{aligned}&\ln \frac{\Omega _M^2}{M_\pi ^2}=\frac{1}{15}\nonumber \\&\quad \times \left( 28\,\ln \frac{\Lambda _1^2}{M_\pi ^2}\!+\!32\,\ln \frac{\Lambda _2^2}{M_\pi ^2}- 33\,\ln \frac{\Lambda _3^2}{M_\pi ^2}\!-\!12\,\ln \frac{\Lambda _4^2}{M_\pi ^2}+52\right) ,\nonumber \\&\quad \ln \frac{\Omega _F^2}{M_\pi ^2}=\frac{1}{3}\,\left( -7\,\ln \frac{\Lambda _1^2}{M_\pi ^2}-8\,\ln \frac{\Lambda _2^2}{M_\pi ^2}\!+\! 18\,\ln \frac{\Lambda _4^2}{M_\pi ^2}\!-\! \frac{29}{2}\right) .\nonumber \\ \end{aligned}$$

#### Two-point correlation functions in the epsilon-regime

The finite-size effects encountered in lattice calculations can be used to determine some of the LECs of QCD. In order to illustrate this point, we focus on the two lightest quarks, take the isospin limit $m_{u}=m_{d}=m$ and consider a box of size $L_{s}$ in the three space directions and size $L_{t}$ in the time direction. If $m$ is sent to zero at fixed box size, chiral symmetry is restored. The behaviour of the various observables in the symmetry-restoration region is controlled by the parameter $\mu \equiv m\,\Sigma \,V$, where $V=L_{s}^3L_{t}$ is the four-dimensional volume of the box. Up to a sign and a factor of two, the parameter $\mu$ represents the minimum of the classical action that belongs to the leading-order effective Lagrangian of QCD.

For $\mu \gg 1$, the system behaves qualitatively as if the box was infinitely large. In that region, the $p$-expansion, which counts $1/L_{s}$, $1/L_{t}$ and $M$ as quantities of the same order, is adequate. In view of $\mu =\frac{1}{2}F^2 M^2V$, this region includes configurations with $ML\gtrsim \! 1$, where the finite-size effects due to pion loop diagrams are suppressed by the factor $e^{-ML}$.

If $\mu$ is comparable to or smaller than 1, however, the chiral perturbation series must be reordered. The $\epsilon$-expansion achieves this by counting $1/L_{s}, 1/L_{t}$ as quantities of $O(\epsilon )$, while the quark mass $m$ is booked as a term of $O(\epsilon ^4)$. This ensures that the symmetry-restoration parameter $\mu$ represents a term of order $O(\epsilon ^0)$, so that the manner in which chiral symmetry is restored can be worked out.

As an example, we consider the correlator of the axial charge carried by the two lightest quarks, $q(x)\!=\!\{u(x),d(x)\}$. The axial current and the pseudoscalar density are given by

55 $$\begin{aligned} A_\mu ^i(x)= \bar{q}(x) \frac{1}{2}\tau ^i\,\gamma _\mu \gamma _5\,q(x),\ P^i(x) = \bar{q}(x) \frac{1}{2} \tau ^i\,{i} \gamma _5\,q(x),\nonumber \\ \end{aligned}$$

where $\tau ^1, \tau ^2,\tau ^3$, are the Pauli matrices in flavour space. In Euclidean space, the correlators of the axial charge and of the space integral over the pseudoscalar density are given by

56 $$\begin{aligned} \delta ^{ik}C_{AA}(t) = L_{s}^3\int \hbox {d}^3 \vec {x}\;{\langle }A_4^i(\vec {x},t) A_4^k(0)\rangle , \nonumber \\ \delta ^{ik}C_{PP}(t) = L_{s}^3\int \hbox {d}^3 \vec {x}\;{\langle }P^i(\vec {x},t) P^k(0)\rangle . \end{aligned}$$

$\chi$PT yields explicit finite-size scaling formulae for these quantities [192, 194, 195]. In the $\epsilon$-regime, the expansion starts with

57 $$\begin{aligned} C_{AA}(t) = \frac{F^2L_{s}^3}{L_{t}}\left[ a_A+ \frac{L_{t}}{F^2L_{s}^3}\,b_A\,h_1 \left( \frac{t}{L_{t}} \right) +O(\epsilon ^4)\right] , \nonumber \\ C_{PP}(t) = \Sigma ^2L_{s}^6\left[ a_P+\frac{L_{t}}{F^2L_{s}^3}\,b_P\,h_1 \left( \frac{t}{L_{t}} \right) +O(\epsilon ^4)\right] ,\nonumber \\ \end{aligned}$$

where the coefficients $a_A$, $b_A$, $a_P$, $b_P$ stand for quantities of $O(\epsilon ^0)$. They can be expressed in terms of the variables $L_{s}$, $L_{t}$ and $m$ and involve only the two leading low-energy constants $F$ and $\Sigma$. In fact, at leading order only the combination $\mu =m\,\Sigma \,L_{s}^3 L_{t}$ matters, the correlators are $t$-independent and the dependence on $\mu$ is fully determined by the structure of the groups involved in the SSB pattern. In the case of SU(2) $\times$ SU(2) $\rightarrow$ SU(2), relevant for QCD in the symmetry-restoration region with two light quarks, the coefficients can be expressed in terms of Bessel functions. The $t$-dependence of the correlators starts showing up at $O(\epsilon ^2)$, in the form of a parabola, viz. $h_1(\tau )=\frac{1}{2}[(\tau -\frac{1}{2})^2-\frac{1}{12}]$. Explicit expressions for $a_A$, $b_A$, $a_P$, $b_P$ can be found in [192, 194, 195], where some of the correlation functions are worked out to NNLO. By matching the finite-size scaling of correlators computed on the lattice with these predictions one can extract $F$ and $\Sigma$. A way to deal with the numerical challenges genuine to the $\epsilon$-regime has been described [196].

The fact that the representation of the correlators to NLO is not “contaminated” by higher-order unknown LECs, makes the $\epsilon$-regime potentially convenient for a clean extraction of the LO couplings. The determination of these LECs is then affected by different systematic uncertainties with respect to the standard case; simulations in this regime yield complementary information which can serve as a valuable cross-check to get a comprehensive picture of the low-energy properties of QCD.

The effective theory can also be used to study the distribution of the topological charge in QCD [197] and the various quantities of interest may be defined for a fixed value of this charge. The expectation values and correlation functions then not only depend on the symmetry-restoration parameter $\mu$, but also on the topological charge $\nu$. The dependence on these two variables can explicitly be calculated. It turns out that the two-point correlation functions considered above retain the form (57), but the coefficients $a_A$, $b_A$, $a_P$, $b_P$ now depend on the topological charge as well as on the symmetry restoration parameter (see [198–200] for explicit expressions).

A specific issue with $\epsilon$-regime calculations is the scale setting. Ideally one would perform a $p$-regime study with the same bare parameters to measure a hadronic scale (e.g. the proton mass). In the literature, sometimes a gluonic scale (e.g. $r_0$) is used to avoid such expenses. Obviously the issues inherent in scale setting are aggravated if the $\epsilon$-regime simulation is restricted to a fixed sector of topological charge.

It is important to stress that in the $\epsilon$-expansion higher-order finite-volume corrections might be significant, and the physical box size (in fm) should still be large in order to keep these contributions under control. The criteria for the chiral extrapolation and finite-volume effects are obviously different from the $p$-regime. For these reasons we have to adjust the colour coding defined in Sect. 2.1 (see Sect. 5.1.6 for more details).

Recently, the effective theory has been extended to the “mixed regime” where some quarks are in the $p$-regime and some in the $\epsilon$-regime [201, 202]. In [203] a technique is proposed to smoothly connect the $p$- and $\epsilon$-regimes. In [204] the issue is reconsidered with a counting rule which is essentially the same as in the $p$-regime. In this new scheme, the theory remains IR finite even in the chiral limit, while the chiral-logarithmic effects are kept present.

#### Energy levels of the QCD Hamiltonian in a box and $\delta$-regime

At low temperature, the properties of the partition function are governed by the lowest eigenvalues of the Hamiltonian. In the case of QCD, the lowest levels are due to the Nambu–Goldstone bosons and can be worked out with $\chi$PT [205]. In the chiral limit the level pattern follows the one of a quantum-mechanical rotator, i.e. $E_\ell =\ell (\ell +1)/(2\,\Theta )$ with $\ell = 0, 1,2,\ldots$. For a cubic spatial box and to leading order in the expansion in inverse powers of the box size $L_{s}$, the moment of inertia is fixed by the value of the pion decay constant in the chiral limit, i.e. $\Theta =F^2L_{s}^3$.

In order to analyse the dependence of the levels on the quark masses and on the parameters that specify the size of the box, a reordering of the chiral series is required, the so-called $\delta$-expansion; the region where the properties of the system are controlled by this expansion is referred to as the $\delta$-regime. Evaluating the chiral perturbation series in this regime, one finds that the expansion of the partition function goes in even inverse powers of $FL_{s}$, that the rotator formula for the energy levels holds up to NNLO and the expression for the moment of inertia is now also known up to and including terms of order $(FL_{s})^{-4}$ [206–208]. Since the level spectrum is governed by the value of the pion decay constant in the chiral limit, an evaluation of this spectrum on the lattice can be used to measure $F$. More generally, the evaluation of various observables in the $\delta$-regime offers an alternative method for a determination of some of the low-energy constants occurring in the effective Lagrangian. At present, however, the numerical results obtained in this way [209, 210] are not yet competitive with those found in the $p$- or $\epsilon$-regimes.

#### Other methods for the extraction of the low-energy constants

An observable that can be used to extract the LECs is the topological susceptibility

58 $$\begin{aligned} \chi _{t}=\int \hbox {d}^4\!x\; {\langle }\omega (x) \omega (0)\rangle , \end{aligned}$$

where $\omega (x)$ is the topological charge density,

59 $$\begin{aligned} \omega (x)=\frac{1}{32\pi ^2} \epsilon ^{\mu \nu \rho \sigma }\mathrm{Tr}\left[ F_{\mu \nu }(x)F_{\rho \sigma }(x)\right] . \end{aligned}$$

At infinite volume, the expansion of $\chi _{t}$ in powers of the quark masses starts with [211]

60 $$\begin{aligned} \chi _{t}=\overline{m}\,\Sigma \,\{1\!+\!O(m)\}, \quad \overline{m}\equiv \left( \frac{1}{m_{u}}\!+\!\frac{1}{m_{d}}+\frac{1}{m_{s}}+\cdots \right) ^{-1}. \end{aligned}$$

The condensate $\Sigma$ can thus be extracted from the properties of the topological susceptibility close to the chiral limit. The behaviour at finite volume, in particular in the region where the symmetry is restored, is discussed in [195]. The dependence on the vacuum angle $\theta$ and the projection on sectors of fixed $\nu$ have been studied in [197]. For a discussion of the finite-size effects at NLO, including the dependence on $\theta$, we refer to [200, 212].

The role that the topological susceptibility plays in attempts to determine whether there is a large paramagnetic suppression when going from the $N_\mathrm{f}=2$ to the $N_\mathrm{f}=2+1$ theory has been highlighted in Ref. [213]. The potential usefulness of higher moments of the topological charge distribution to determine LECs has been investigated in [214].

Another method for computing the quark condensate has been proposed in [215], where it is shown that starting from the Banks–Casher relation [216] one may extract the condensate from suitable (renormalisable) spectral observables, for instance the number of Dirac operator modes in a given interval. For those spectral observables higher-order corrections can be systematically computed in terms of the chiral effective theory. A recent paper based on this strategy is ETM 13 [217]. As an aside let us remark that corrections to the Banks–Casher relation that come from a finite quark mass, a finite four-dimensional volume and (with Wilson-type fermions) a finite lattice spacing can be parameterised in a properly extended version of the chiral framework [218].

An alternative strategy is based on the fact that at LO in the $\epsilon$-expansion the partition function in a given topological sector $\nu$ is equivalent to the one of a chiral Random Matrix Theory (RMT) [219–222]. In RMT it is possible to extract the probability distributions of individual eigenvalues [223–225] in terms of two dimensionless variables $\zeta =\lambda \Sigma V$ and $\mu =m\Sigma V$, where $\lambda$ represents the eigenvalue of the massless Dirac operator and $m$ is the sea quark mass. More recently this approach has been extended to the Hermitian (Wilson) Dirac operator [226] which is easier to study in numerical simulations. Hence, if it is possible to match the QCD low-lying spectrum of the Dirac operator to the RMT predictions, then one may extract^18^ the chiral condensate $\Sigma$. One issue with this method is that for the distributions of individual eigenvalues higher-order corrections are still not known in the effective theory, and this may introduce systematic effects which are hard^19^ to control. Another open question is that, while it is clear how the spectral density is renormalised [230], this is not the case for the individual eigenvalues, and one relies on assumptions. There have been many lattice studies [231–235] which investigate the matching of the low-lying Dirac spectrum with RMT. In this review the results of the LECs obtained in this way^20^ are not included.

#### Pion form factors

The scalar and vector form factors of the pion are defined by the matrix elements

61 $$\begin{aligned}&{\langle }\pi ^i(p_2) |\, \bar{q}\, q \, | \pi ^j(p_1) {\rangle } = \delta ^{ij} F_S^\pi (t) ,\\&{\langle }\pi ^i(p_2) | \,\bar{q}\, \frac{1}{2} \tau ^k \gamma ^\mu q\,| \pi ^j(p_1) {\rangle } = \hbox {i} \,\epsilon ^{ikj} (p_1^\mu + p_2^\mu ) F_V^\pi (t) ,\nonumber \end{aligned}$$

where the operators contain only the lightest two quark flavours, i.e. $\tau ^1$, $\tau ^2$, $\tau ^3$ are the Pauli matrices, and $t\equiv (p_1-p_2)^2$ denotes the momentum transfer.

The vector form factor has been measured by several experiments for timelike as well as for spacelike values of $t$. The scalar form factor is not directly measurable, but it can be evaluated theoretically from data on the $\pi \pi$ and $\pi K$ phase shifts [236] by means of analyticity and unitarity, i.e. in a model-independent way. Lattice calculations can be compared with data or model-independent theoretical evaluations at any given value of $t$. At present, however, most lattice studies concentrate on the region close to $t=0$ and on the evaluation of the slope and curvature which are defined as

62 $$\begin{aligned} F^\pi _V(t)&= 1+{\frac{1}{6}}{\langle }r^2 \rangle ^\pi _V t + c_V t^2+\cdots \;,\\ F^\pi _S(t)&= F^\pi _S(0) \left[ 1+ {\frac{1}{6}}{\langle }r^2 \rangle ^\pi _S t + c_S\, t^2+ \cdots \right] . \nonumber \end{aligned}$$

The slopes are related to the mean-square vector and scalar radii which are the quantities on which most experiments and lattice calculations concentrate.

In chiral perturbation theory, the form factors are known at NNLO [237]. The corresponding formulae are available in fully analytical form and are compact enough that they can be used for the chiral extrapolation of the data (as done, for example in [238, 239]). The expressions for the scalar and vector radii and for the $c_{S,V}$ coefficients at two-loop level read

63 $$\begin{aligned}&{\langle }r^2 \rangle ^\pi _S = \frac{1}{(4\pi F_\pi )^2}\nonumber \\&\times \left\{ 6 \ln \frac{\Lambda _4^2}{M_\pi ^2}-\frac{13}{2} \!-\!\frac{29}{3}\,\xi \left( \ln \frac{\Omega _{r_S}^2}{M_\pi ^2}\right) ^2+ 6 \xi \, k_{r_S}+O(\xi ^2)\right\} ,\nonumber \\&{\langle }r^2 \rangle ^\pi _V = \frac{1}{(4\pi F_\pi )^2}\nonumber \\&\times \left\{ \ln \frac{\Lambda _6^2}{M_\pi ^2}-1 +2\,\xi \left( \ln \frac{\Omega _{r_V}^2}{M_\pi ^2}\right) ^2+6 \xi \,k_{r_V}+O(\xi ^2)\right\} ,\nonumber \\&c_S =\frac{1}{(4\pi F_\pi M_\pi )^2} \left\{ \frac{19}{120} + \xi \left[ \frac{43}{36} \left( \! \ln \frac{\Omega _{c_S}^2}{M_\pi ^2} \!\right) ^2 + k_{c_S} \right] \right\} ,\nonumber \\&c_V =\frac{1}{(4\pi F_\pi M_\pi )^2} \left\{ \frac{1}{60}+\xi \left[ \frac{1}{72} \left( \ln \frac{\Omega _{c_V}^2}{M_\pi ^2} \right) ^2 + k_{c_V} \right] \right\} ,\nonumber \\ \end{aligned}$$

where

64 $$\begin{aligned} \ln \frac{\Omega _{r_S}^2}{M_\pi ^2}&= \frac{1}{29}\,\left( 31\,\ln \frac{\Lambda _1^2}{M_\pi ^2}+34\,\ln \frac{\Lambda _2^2}{M_\pi ^2}-36\,\ln \frac{\Lambda _4^2}{M_\pi ^2}+\frac{145}{24}\right) ,\nonumber \\ \ln \frac{\Omega _{r_V}^2}{M_\pi ^2}&= \frac{1}{2}\,\left( \ln \frac{\Lambda _1^2}{M_\pi ^2}-\ln \frac{\Lambda _2^2}{M_\pi ^2}+\ln \frac{\Lambda _4^2}{M_\pi ^2}+\ln \frac{\Lambda _6^2}{M_\pi ^2}-\frac{31}{12}\right) ,\nonumber \\ \ln \frac{\Omega _{c_S}^2}{M_\pi ^2}\!&= \!\frac{43}{63}\,\left( 11\,\ln \frac{\Lambda _1^2}{M_\pi ^2}\!+\!14\,\ln \frac{\Lambda _2^2}{M_\pi ^2}\!+\!18\,\ln \frac{\Lambda _4^2}{M_\pi ^2}\!-\!\frac{6041}{120}\right) ,\nonumber \\ \ln \frac{\Omega _{c_V}^2}{M_\pi ^2}&= \frac{1}{72}\,\left( 2\ln \frac{\Lambda _1^2}{M_\pi ^2}-2\ln \frac{\Lambda _2^2}{M_\pi ^2}-\ln \frac{\Lambda _6^2}{M_\pi ^2}-\frac{26}{30}\right) ,\end{aligned}$$

and $k_{r_S},k_{r_V}$ and $k_{c_S},k_{c_V}$ are independent of the quark masses. Their expression in terms of the $\ell _i$ and of the $O(p^6)$ constants $c_M,c_F$ is known but will not be reproduced here.

The difference between the quark-line connected and the full (i.e. containing the connected and the disconnected piece) scalar pion form factor has been investigated by means of Chiral Perturbation Theory in [240]. It is expected that the technique used can be applied to a large class of observables relevant in QCD-phenomenology.

As a point of practical interest let us remark that there are no finite-volume correction formulae for the mean-square radii ${\langle }r^2{\rangle }_{V,S}$ and the curvatures $c_{V,S}$. The lattice data for $F_{V,S}(t)$ need to be corrected, point by point in $t$, for finite-volume effects. In fact, if a given $t$ is realised through several inequivalent $p_1-p_2$ combinations, the level of agreement after the correction has been applied is indicative of how well higher-order effects are under control.

#### Lattice determinations

**Fig. 8:**
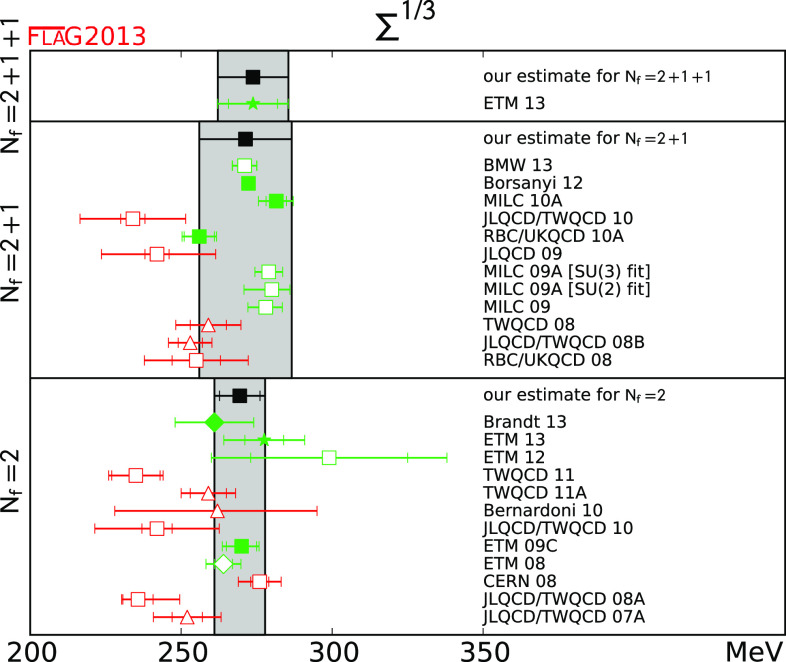
Quark condensate $\Sigma \equiv |\langle \bar{u}u\rangle |_{m_{u},m_{d}\rightarrow 0}$ ($\overline{\mathrm{MS}}$-scheme, scale $\mu =2$ GeV). *Squares* and *left triangles* indicate determinations from correlators in the $p$- and $\epsilon$-regimes, respectively. *Up triangles* refer to extractions from the topological susceptibility, *diamonds* to determinations from the pion form factor, and *star symbols* refer to the spectral density method. The *black squares* and *grey bands* indicate our estimates. The meaning of the colours is explained in Sect. 2

**Fig. 9:**
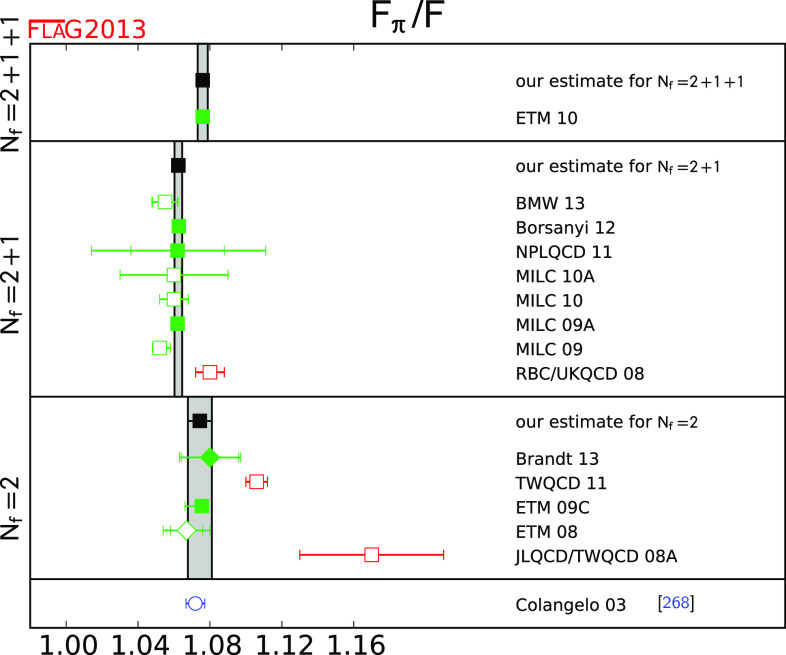
Comparison of the results for the ratio of the physical pion decay constant $F_\pi$ and the leading-order SU(2) low-energy constant $F$. The meaning of the symbols is the same as in Fig. 8

**Fig. 10:**
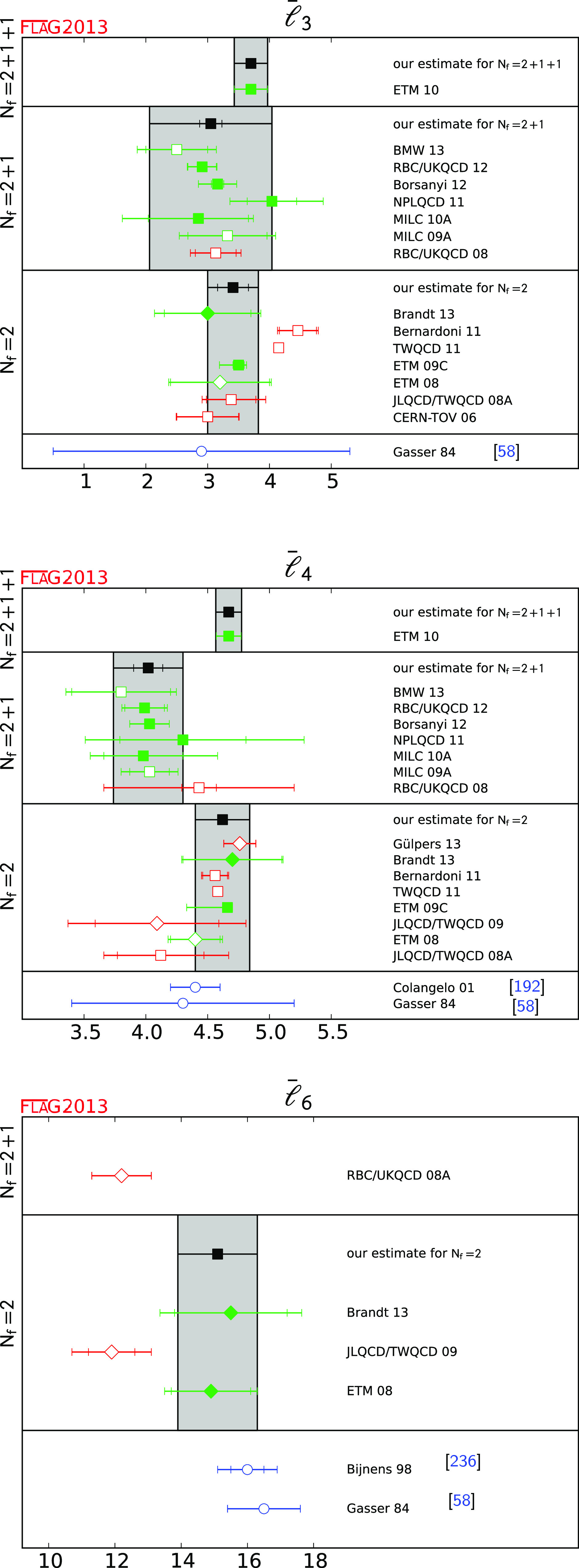
Effective coupling constants $\bar{\ell }_3$, $\bar{\ell }_4$ and $\bar{\ell }_6$. *Squares* indicate determinations from correlators in the $p$-regime, *diamonds* refer to determinations from the pion form factor

In this section we summarise the lattice results for the SU(2) couplings in a set of Tables 13, 14, 15, 16 and Figs. 8, 9, 10). The tables present our usual colour coding which summarises the main aspects related to the treatment of the systematic errors of the various calculations.

**Table 13:** Quark condensate $\Sigma \equiv |\langle \bar{u}u\rangle |_{m_{u},m_{d}\rightarrow 0}$: colour code and numerical values in MeV (compare Fig. 8)

Collaboration	Ref.	$N_\mathrm{f}$	Publication status	Chiral extrapolation	Continuum extrapolation	Finite volume	Renormalisation	$\Sigma ^{1/3}$
ETM 13	[217]	$2+1+1$	A					274 (08) (08)
BMW 13	[254]	$2+1$	P					271 (4) (1)
Borsanyi 12	[249]	$2+1$	A					272.3 (1.2) (1.4)
MILC 10A	[75]	$2+1$	C					281.5 (3.4)$$\left( {\begin{array}{c}+2.0\\ -5.9\end{array}}\right) $$ (4.0)
JLQCD/TWQCD 10	[252]	$2+1$	A					234 (4) (17)
RBC/UKQCD 10A	[78]	$2+1$	A					256 (5) (2) (2)
JLQCD 09	[251]	$2+1$	A					242 (4)$$\left( {\begin{array}{c}+19\\ -18\end{array}}\right) $$
MILC 09A	[37]	$2+1$	C					279 (1) (2) (4)
MILC 09A	[37]	$2+1$	C					280 (2)$$\left( {\begin{array}{c}+4\\ -8\end{array}}\right) $$ (4)
MILC 09	[15]	$2+1$	A					278 (1)$$\left( {\begin{array}{c}+2\\ -3\end{array}}\right) $$ (5)
TWQCD 08	[255]	$2+1$	A					259 (6) (9)
JLQCD/TWQCD 08B	[256]	$2+1$	C					253 (4) (6)
PACS-CS 08	[19]	$2+1$	A					312 (10)
PACS-CS 08	[19]	$2+1$	A					309 (7)
RBC/UKQCD 08	[79]	$2+1$	A					255 (8) (8) (13)
Brandt 13	[257]	2	A					261 (13) (1)
ETM 13	[217]	2	A					277 (06) (12)
ETM 12	[258]	2	A					299 (26) (29)
Bernardoni 11	[259]	2	C					306 (11)
TWQCD 11	[185]	2	A					235 (8) (4)
TWQCD 11A	[260]	2	A					259 (6) (7)
Bernardoni 10	[261]	2	A					262$$\left( {\begin{array}{c}+33\\ -34\end{array}}\right) \left( {\begin{array}{c}+4\\ -5\end{array}}\right) $$
JLQCD/TWQCD 10	[252]	2	A					242 (5) (20)
ETM 09C	[241]	2	A					270 (5)$$\left( {\begin{array}{c}+3\\ -4\end{array}}\right) $$
ETM 08	[238]	2	A					264 (3) (5)
CERN 08	[215]	2	A					276 (3) (4) (5)
JLQCD/TWQCD 08A	[67]	2	A					235.7 (5.0) (2.0)$$\left( {\begin{array}{c}+12.7\\ -0.0\end{array}}\right) $$
JLQCD/TWQCD 07A	[262]	2	A					252 (5) (10)
ETM 09B	[263]	2	C					239.6 (4.8)
Hasenfratz 08	[264]	2	A					248 (6)
JLQCD/TWQCD 07	[265]	2	A					239.8 (4.0)

**Table 14:** Results for the leading-order SU(2) low-energy constant $F$ (in MeV) and for the ratio $F_\pi /F$. Numbers in slanted fonts have been calculated by us (see text for details). Horizontal lines establish the same grouping as in Table 13

Collaboration	Ref.	$N_\mathrm{f}$	Publication status	Chiral extrapolation	Continuum extrapolation	Finite volume	Renormalisation	$F$	$F_\pi /F$
ETM 11	[266]	$2+1+1$	C					85.60 (4)	*1.077 (2)*
ETM 10$^{\dag }$	[98]	$2+1+1$	A					85.66 (6) (13)	1.076 (2) (2)
BMW 13	[254]	$2+1$	P					88.0 (1.3) (0.3)	1.055 (7) (2)
Borsanyi 12	[249]	$2+1$	A					86.78 (05) (25)	1.0627 (06) (27)
NPLQCD 11	[267]	$2+1$	A						1.062 (26)$$\left( {\begin{array}{c}+42\\ -40\end{array}}\right) $$
MILC 10A	[75]	$2+1$	C					87.5 (1.0)$$\left( {\begin{array}{c}+0.7\\ -2.6\end{array}}\right) $$	*1.06 (3)*
MILC 10	[159]	$2+1$	C					87.0 (4) (5)	*1.060 (8)*
MILC 09A	[37]	$2+1$	C					86.8 (2) (4)	1.062 (1) (3)
MILC 09	[15]	$2+1$	A						1.052 (2)$$\left( {\begin{array}{c}+6\\ -3\end{array}}\right) $$
PACS-CS 08	[19]	$2+1$	A					89.4 (3.3)	1.060 (7)
RBC/UKQCD 08	[79]	$2+1$	A					81.2 (2.9) (5.7)	1.080 (8)
Brandt 13	[257]	2	A					84 (8) (2)	1.080 (16) (6)
QCDSF 13	[268]	2	P					86 (1)	*1.07 (1)*
Bernardoni 11	[259]	2	C					79 (4)	*1.17 (5)*
TWQCD 11	[185]	2	A					83.39 (35) (38)	*1.106 (6)*
ETM 09C	[241]	2	A						1.0755 (6)$$\left( {\begin{array}{c}+08\\ -94\end{array}}\right) $$
ETM 08	[238]	2	A					86.6 (7) (7)	1.067 (9) (9)
JLQCD/TWQCD 08A	[67]	2	A					79.0 (2.5) (0.7)$$\left( {\begin{array}{c}+4.2\\ -0.0\end{array}}\right) $$	*1.17 (4)*
ETM 09B^§^	[263]	2	C					90.2 (4.8)	*1.02 (5)*
Hasenfratz 08	[264]	2	A					90 (4)	*1.02 (4)*
JLQCD/TWQCD 07	[265]	2	A					87.3 (5.6)	*1.06 (6)*
Colangelo 03	[269]							86.2 (5)	1.0719 (52)

$\dag$ The values of $M_{\pi ^+} L$ correspond to a green tag in the FV-column, while those of $M_{\pi ^0} L$ imply a red one; since both masses play a role in finite-volume effects, we opt for open green

^§^ Result for $r_0 F$ converted into a value for $F$ via $r_0=0.49\,\,{\mathrm {fm}}$ (despite ETM quoting smaller values of $r_0$)

**Table 15:** Results for the SU(2) NLO couplings $\bar{\ell }_3$ and $\bar{\ell }_4$. The MILC 10 results are obtained by converting the SU(3) LECs, while the MILC 10A results are obtained with a direct SU(2) fit. For comparison, the last two lines show results from phenomenological analyses

Collaboration	Ref.	$N_\mathrm{f}$	Publication status	Chiral extrapolation	Continuum extrapolation	Finite volume	$\bar{\ell }_3$	$\bar{\ell }_4$
ETM 11	[266]	$2+1+1$	C				3.53 (5)	4.73 (2)
ETM 10	[98]	$2+1+1$	A				3.70 (7) (26)	4.67 (3) (10)
BMW 13	[254]	$2+1$	P				2.5 (5) (4)	3.8 (4) (2)
RBC/UKQCD 12	[25]	$2+1$	A				2.91 (23) (07)	3.99 (16) (09)
Borsanyi 12	[249]	$2+1$	A				3.16 (10) (29)	4.03 (03) (16)
NPLQCD 11	[267]	$2+1$	A				4.04 (40) $$\left( {\begin{array}{c}+73\\ -55\end{array}}\right) $$	4.30 (51) $$\left( {\begin{array}{c}+84\\ -60\end{array}}\right) $$
MILC 10A	[75]	$2+1$	C				2.85 (81) $$\left( {\begin{array}{c}+37\\ -92\end{array}}\right) $$	3.98 (32) $$\left( {\begin{array}{c}+51\\ -28\end{array}}\right) $$
MILC 10	[159]	$2+1$	C				3.18 (50) (89)	4.29 (21) (82)
RBC/UKQCD 10A	[78]	$2+1$	A				2.57 (18)	3.83 (9)
MILC 09A	[37]	$2+1$	C				3.32 (64) (45)	4.03 (16) (17)
MILC 09A	[37]	$2+1$	C				3.0 (6) $$\left( {\begin{array}{c}+9\\ -6\end{array}}\right) $$	3.9 (2) (3)
PACS-CS 08	[19]	$2+1$	A				3.47 (11)	4.21 (11)
PACS-CS 08	[19]	$2+1$	A				3.14 (23)	4.04 (19)
RBC/UKQCD 08	[79]	$2+1$	A				3.13 (33) (24)	4.43 (14) (77)
Gülpers 13	[270]	2	P					4.76 (13) (–)
Brandt 13	[257]	2	A				3.0 (7) (5)	4.7 (4) (1)
QCDSF 13	[268]	2	P					4.2 (1)
Bernardoni 11	[259]	2	C				4.46 (30) (14)	4.56 (10) (4)
TWQCD 11	[185]	2	A				4.149 (35) (14)	4.582 (17) (20)
ETM 09C	[241]	2	A				3.50 (9) $$\left( {\begin{array}{c}+09\\ -30\end{array}}\right) $$	4.66 (4) $$\left( {\begin{array}{c}+04\\ -33\end{array}}\right) $$
JLQCD/TWQCD 09	[271]	2	A					4.09 (50) (52)
ETM 08	[238]	2	A				3.2 (8) (2)	4.4 (2) (1)
JLQCD/TWQCD 08A	[67]	2	A				3.38 (40) (24) $$\left( {\begin{array}{c}+31\\ -00\end{array}}\right) $$	4.12 (35) (30) $$\left( {\begin{array}{c}+31\\ -00\end{array}}\right) $$
CERN-TOV 06	[272]	2	A				3.0 (5) (1)	
Colangelo 01	[193]							4.4 (2)
Gasser 84	[58]						2.9 (2.4)	4.3 (9)

**Table 16:** *Top panel: vector form factor of the pion.* Lattice results for the charge radius ${\langle }r^2{\rangle }_V^\pi$ (in ${\mathrm {fm}}^2$), the curvature $c_V$ (in ${\mathrm {GeV}}^{-4}$) and the effective coupling constant $\bar{\ell }_6$ are compared with the experimental value obtained by NA7 and some phenomenological estimates. *Bottom panel: scalar form factor of the pion.* Lattice results for the scalar radius ${\langle } r^2 {\rangle }_S^\pi$ (in ${\mathrm {fm}}^2$) and the combination $\bar{\ell }_1-\bar{\ell }_2$ are compared with a dispersive calculation of these quantities [193]

Collaboration	Ref.	$N_\mathrm{f}$	Publication status	Chiral extrapolation	Continuum extrapolation	Finite volume	${\langle }r^2{\rangle }_V^\pi$	$c_V$	$\bar{\ell }_6$
RBC/UKQCD 08A	[253]	$2+1$	A				0.418 (31)		12.2 (9)
LHP 04	[275]	$2+1$	A				0.310 (46)		
Brandt 13	[257]	2	A				0.481 (33) (13)		15.5 (1.7) (1.3)
JLQCD/TWQCD 09	[271]	2	A				0.409 (23) (37)	3.22 (17) (36)	11.9 (0.7) (1.0)
ETM 08	[238]	2	A				0.456 (30) (24)	3.37 (31) (27)	14.9 (1.2) (0.7)
QCDSF/UKQCD 06A	[276]	2	A				0.441 (19) (56) (29)		
Bijnens 98	[237]						0.437 (16)	3.85 (60)	16.0 (0.5) (0.7)
NA7 86	[277]						0.439 (8)		
Gasser 84	[58]								16.5 (1.1)
Collaboration	Ref.	$N_\mathrm{f}$	Publication status	Chiral extrapolation	Continuum extrapolation	Finite volume	${\langle }r^2{\rangle }_S^\pi$	$\bar{\ell }_1-\bar{\ell }_2$	
Gülpers 13	[270]	2	P				0.637 (23) (–)		
JLQCD/TWQCD 09	[271]	2	A				0.617 (79) (66)	$-$2.9 (0.9) (1.3)	
Colangelo 01	[193]						0.61 (4)	$-$4.7 (6)

A delicate issue in the lattice determination of chiral LECs (in particular at NLO) which cannot be reflected by our colour coding is a reliable assessment of the theoretical error that comes from the chiral expansion. We add a few remarks on this point:

1. Using *both* the $x$ and the $\xi$ expansion is a good way to test how the ambiguity of the chiral expansion (at a given order) affects the numerical values of the LECs that are determined from a particular set of data. For instance, to determine $\bar{\ell }_4$ (or $\Lambda _4$) from lattice data for $F_\pi$ as a function of the quark mass, one may compare the fits based on the parameterisation $F_\pi =F\{1+x\ln (\Lambda _4^2/M^2)\}$ [see Eq. (48)] with those obtained from $F_\pi =F/\{1-\xi \ln (\Lambda _4^2/M_\pi ^2)\}$ [see Eq. (53)]. The difference between the two results provides an estimate of the uncertainty due to the truncation of the chiral series. Which central value one chooses is in principle arbitrary, but we find it advisable to use the one obtained with the $\xi$ expansion,^21^ in particular because it makes the comparison with phenomenological determinations (where it is standard practice to use the $\xi$ expansion) more meaningful.
2. Alternatively one could try to estimate the influence of higher chiral orders by reshuffling irrelevant higher-order terms. For instance, in the example mentioned above one might use $F_\pi =F/\{1-x\ln (\Lambda _4^2/M^2)\}$ as a different functional form at NLO. Another way to establish such an estimate is through introducing by hand “analytical” higher-order terms (e.g. “analytical NNLO” as done, in the past, by MILC [15]). In principle it would be preferable to include all NNLO terms or none, such that the structure of the chiral expansion is preserved at any order (this is what ETM [241] and JLQCD/TWQCD [67] have done for SU(2) $\chi$PT and MILC for SU(3) $\chi$PT [37]). There are different opinions in the field as to whether it is advisable to include terms to which the data are not sensitive. In case one is willing to include external (typically: non-lattice) information, the use of priors is a theoretically well-founded option (e.g. priors for NNLO LECs if one is interested in LECs at LO/NLO).
3. Another issue concerns the $s$-quark mass dependence of the LECs $\bar{\ell }_i$ or $\Lambda _i$ of the SU(2) framework. As far as variations of $m_{s}$ around $m_{s}^\mathrm{phys}$ are concerned (say for $0<m_{s}<1.5m_{s}^\mathrm{phys}$ at best) the issue can be studied in SU(3) ChPT, and this has been done in a series of papers [56, 242, 243]. However, the effect of sending $m_{s}$ to infinity, as is the case in $N_\mathrm{f}=2$ lattice studies of SU(2) LECs, cannot be addressed in this way. A unique way to analyse this difference is to compare the numerical values of LECs determined in $N_\mathrm{f}=2$ lattice simulations to those determined in $N_\mathrm{f}=2+1$ lattice simulations (see e.g. [244] for a discussion).
4. Last but not least let us recall that the determination of the LECs is affected by discretisation effects, and it is important that these are removed by means of a continuum extrapolation. In this step invoking an extended version of the chiral Lagrangian [245–247] may be useful^22^ in case one aims for a global fit of lattice data involving several $M_\pi$ and $a$ values and several chiral observables.

In the tables and figures we summarise the results of various lattice collaborations for the SU(2) LECs at LO ($F$ or $F/F_\pi$, $B$ or $\Sigma$) and at NLO ($\bar{\ell }_1-\bar{\ell }_2$, $\bar{\ell }_3$, $\bar{\ell }_4$, $\bar{\ell }_5$, $\bar{\ell }_6$). Throughout we group the results into those which stem from $N_\mathrm{f}=2+1+1$ calculations, those which come from $N_\mathrm{f}=2+1$ calculations and those which stem from $N_\mathrm{f}=2$ calculations (since, as mentioned above, the LECs are logically distinct even if the current precision of the data is not sufficient to resolve the differences). Furthermore, we make a distinction whether the results are obtained from simulations in the $p$-regime or whether alternative methods ($\epsilon$-regime, spectral quantities, topological susceptibility, etc.) have been used (this should not affect the result). For comparison we add, in each case, a few phenomenological determinations with high standing.

A generic comment applies to the issue of the scale setting. In the past none of the lattice studies with $N_\mathrm{f}\ge 2$ involved simulations in the $p$-regime at the physical value of $m_{ud}$. Accordingly, the setting of the scale $a^{-1}$ via an experimentally measurable quantity did necessarily involve a chiral extrapolation, and as a result of this dimensionful quantities used to be particularly sensitive to this extrapolation uncertainty, while in dimensionless ratios such as $F_\pi /F$, $F/F_0$, $B/B_0$, $\Sigma /\Sigma _0$ this particular problem is much reduced (and often finite lattice-to-continuum renormalisation factors drop out). Now, there is a new generation of lattice studies [20, 22, 23, 140, 249, 250] which does involve simulations at physical pion masses. In such studies even the uncertainty that the scale setting has on dimensionful quantities is much mitigated.

It is worth repeating here that the standard colour-coding scheme of our tables is necessarily schematic and cannot do justice to every calculation. In particular there is some difficulty in coming up with a fair adjustment of the rating criteria to finite-volume regimes of QCD. For instance, in the $\epsilon$-regime^23^ we re-express the “chiral-extrapolation” criterion in terms of $\sqrt{2m_\mathrm{min}\Sigma }/F$, with the same threshold values (in MeV) between the three categories as in the $p$-regime. Also the “infinite-volume” assessment is adapted to the $\epsilon$-regime, since the $M_\pi L$ criterion does not make sense here; we assign a green star if at least two volumes with $L>2.5\,{\mathrm {fm}}$ are included, an open symbol if at least one volume with $L>2\,{\mathrm {fm}}$ is invoked and a red square if all boxes are smaller than $2\,{\mathrm {fm}}$. Similarly, in the calculation of form factors and charge radii the tables do not reflect whether an interpolation to the desired $q^2$ has been performed or whether the relevant $q^2$ has been engineered by means of “partially twisted boundary conditions” [253]. In spite of these limitations we feel that these tables give an adequate overview of the qualities of the various calculations.

We begin with a discussion of the lattice results for the SU(2) LEC $\Sigma$. We present the results in Table 13 and Fig. 8. We add that results which include only a statistical error are listed in the table but omitted from the plot. Regarding the $N_\mathrm{f}=2$ computations there are five entries without a red tag (ETM 08, ETM 09C, ETM 12, ETM 13, Brandt 13). We form the average based on ETM 09C, ETM 13 (here we deviate from our “superseded” rule, since the latter work has a much bigger error) and Brandt 13. Regarding the $N_\mathrm{f}=2+1$ computations there are three published papers (RBC/UKQCD 10A, MILC 10A and Borsanyi 12) which make it into the $N_\mathrm{f}=2+1$ average and a preprint (BMW 13) which will be included in a future update. We also remark that among the three works included RBC/UKQCD 10A is inconsistent with the other two (MILC 10A and Borsanyi 12). For the time being we inflate the error of our $N_\mathrm{f}=2+1$ average such that it includes all three central values it is based on. This yields

65 $$\begin{aligned} \Sigma \big |_{N_\mathrm{f}=2}=269(08)\,{\mathrm {MeV}},\quad \Sigma \big |_{N_\mathrm{f}=2+1}=271(15)\,{\mathrm {MeV}},\nonumber \\ \end{aligned}$$

where the errors include both statistical and systematic uncertainties. In accordance with our guidelines we plead with the reader to cite [217, 241, 257] (for $N_\mathrm{f}=2$) or [75, 78, 249] (for $N_\mathrm{f}=2+1$) when using these numbers. Finally, for $N_\mathrm{f}=2+1+1$ there is only one calculation, and we recommend to use the result of [217] as given in Table 13. Another look at Fig. 8 confirms that these values are well consistent with each other.

The next quantity considered is $F$, i.e. the pion decay constant in the SU(2) chiral limit ($m_{ud}\rightarrow 0$ at fixed physical $m_{s}$) in the Bernese normalisation. As argued on previous occasions we tend to give preference to $F_\pi /F$ (here the numerator is meant to refer to the physical-pion-mass point) wherever it is available, since often some of the systematic uncertainties are mitigated. We collect the results in Table 14 and Fig. 9. In those cases where the collaboration provides only $F$, the ratio is computed on the basis of the phenomenological value of $F_\pi$, and the corresponding entries in Table 14 are in slanted fonts. Among the $N_\mathrm{f}=2$ determinations only three (ETM 08, ETM 09C and Brandt 13) are without red tags. Since the first two are by the same collaboration, only the latter two enter the average. Among the $N_\mathrm{f}=2+1$ determinations three values (MILC 09A as an obvious update of MILC 09, NPLQCD 11 and Borsanyi 12) make it into the average. Finally, there is a single $N_\mathrm{f}=2+1+1$ determination (ETM 10) which forms the current best estimate in this category.

Given this input our averaging procedure yields

66 $$\begin{aligned} \frac{F_\pi }{F}\big |_{N_\mathrm{f}=2}=1.0744(67),\quad \frac{F_\pi }{F}\big |_{N_\mathrm{f}=2+1}=1.0624(21), \end{aligned}$$

where the errors include both statistical and systematic uncertainties. We plead with the reader to cite [241, 257] (for $N_\mathrm{f}=2$) or [37, 249, 267] (for $N_\mathrm{f}=2+1$) when using these numbers. Finally, for $N_\mathrm{f}=2+1+1$ we recommend to use the result of [98]; see Table 14 for the numerical value. From these numbers (or from a look at Fig. 9) it is obvious that the $N_\mathrm{f}=2+1$ and $N_\mathrm{f}=2+1+1$ results are not quite consistent. From a theoretical viewpoint this is rather surprising, since the only difference (the presence of absence of a dynamical charm quark) is expected to have a rather insignificant effect on this ratio (which, in addition, would be monotonic in $N_\mathrm{f}$, contrary to what is seen in Fig. 9). In our view this indicates that—in spite of the conservative attitude taken in this report—the theoretical uncertainties in at least one of the two cases is likely underestimated. We hope that a future release of the FLAG report can clarify the issue.

We move on to a discussion of the lattice results for the NLO LECs $\bar{\ell }_3$ and $\bar{\ell }_4$. We remind the reader that on the lattice the former LEC is obtained as a result of the tiny deviation from linearity seen in $M_\pi ^2$ versus $Bm_{ud}$, whereas the latter LEC is extracted from the curvature in $F_\pi$ versus $Bm_{ud}$. The available determinations are presented in Table 15 and Fig. 10. Among the $N_\mathrm{f}=2$ determinations ETM 08, ETM 09C and Brandt 13 are published and without red tags, and our rules imply that the latter two determinations enter our average. The colour coding of the $N_\mathrm{f}=2+1$ results looks very promising; there is a significant number of lattice determinations without any red tag. At first sight it seems that RBC/UKQCD 10A, MILC 10A, NPLQCD 11, Borsanyi 12 and RBC/UKQCD 12 make it into the average. Unfortunately, $\bar{\ell }_3$ and $\bar{\ell }_4$ of RBC/UKQCD 10A have no systematic error; therefore we exclude this work from the $N_\mathrm{f}=2+1$ average. Among the $N_\mathrm{f}=2+1+1$ determinations only ETM 10 qualifies for an average.

Given this input our averaging procedure yields

67 $$\begin{aligned} \bar{\ell }_3\big |_{N_\mathrm{f}=2}=3.41(41),\quad \bar{\ell }_3\big |_{N_\mathrm{f}=2+1}=3.05(99), \end{aligned}$$

68 $$\begin{aligned} \bar{\ell }_4\big |_{N_\mathrm{f}=2}=4.62(22),\quad \bar{\ell }_4\big |_{N_\mathrm{f}=2+1}=4.02(28), \end{aligned}$$

where the errors include both statistical and systematic uncertainties. Again we plead with the reader to cite [241, 257] (for $N_\mathrm{f}=2$) or [25, 75, 249, 267] (for $N_\mathrm{f}=2+1$) when using these numbers. For $N_\mathrm{f}=2+1+1$ we stay with the recommendation to use the results of [98], see Table 15 for the numerical values.

Let us add two remarks. On the input side our procedure^24^ symmetrises the asymmetric error of ETM 09C with a slight adjustment of the central value. On the output side the error of the $\bar{\ell }_3$ average for $N_\mathrm{f}=2$ and of the $\bar{\ell }_3,\bar{\ell }_4$ averages for $N_\mathrm{f}=2+1$, according to the FLAG procedure, got inflated by hand to cover all central values. From these numbers (or from a look at Fig. 10) it is clear that the lattice results for $\bar{\ell }_3$ do not show any obvious $N_\mathrm{f}$-dependence—thanks, chiefly, to our conservative error treatment strategy. On the other hand, in the case of $\bar{\ell }_4$ even our practice of inflating the error of the $N_\mathrm{f}=2+1$ average did not manage to avoid some mild inconsistency between the $N_\mathrm{f}=2+1$ average on one side and either the $N_\mathrm{f}=2$ or the $N_\mathrm{f}=2+1+1$ average on the other side. Again, the dependence of the average on the number of active flavours is not monotonic, and this raises a decent amount of suspicion that some of the systematic errors might still be underestimated.

More specifically, it seems that again the $N_\mathrm{f}=2+1+1$ value by ETM shows some tension relative to the average $N_\mathrm{f}=2+1$ value quoted above, in close analogy to what happened for $F$ or $F_\pi /F$; see the discussion around (66). Since both $F$ and $\bar{\ell }_4$ are determined from the quark-mass dependence of the pseudoscalar decay constant, perhaps the formulae in Refs. [273, 274] for dealing with cutoff and finite-volume effects with twisted-mass data might prove useful in future analysis.

From a more phenomenological viewpoint there is a notable difference between $\bar{\ell }_3$ and $\bar{\ell }_4$ in Fig. 10. For $\bar{\ell }_4$ the precision of the phenomenological determination achieved in Colangelo 01 [193] represents a significant improvement compared to Gasser 84 [58]. Picking any $N_\mathrm{f}$, the lattice average of $\bar{\ell }_4$ is consistent with both of the phenomenological values and comes with an error which is roughly comparable to the uncertainty of the result in Colangelo 01 [193]. By contrast, for $\bar{\ell }_3$ the error of the lattice determination is significantly smaller than the error of the estimate given in Gasser 84 [58]. In other words, here the lattice really provides some added value.

We finish with a discussion of the lattice results for $\bar{\ell }_6$ and $\bar{\ell }_1-\bar{\ell }_2$. The LEC $\bar{\ell }_6$ determines the leading contribution in the chiral expansion of the pion charge radius—see (63). Hence from a lattice study of the vector form factor of the pion with several $M_\pi$ one may extract the radius ${\langle }r^2{\rangle }_V^\pi$, the curvature $c_V$ (both at the physical pion-mass point) and the LEC $\bar{\ell }_6$ in one go. Similarly, the leading contribution in the chiral expansion of the scalar radius of the pion determines $\bar{\ell }_4$—see (63). This LEC is also present in the pion-mass dependence of $F_\pi$, as we have seen. The difference $\bar{\ell }_1-\bar{\ell }_2$, finally, may be obtained from the momentum dependence of the vector and scalar pion form factors, based on the two-loop formulae of [237]. The top part of Table 16 collects the results obtained from the vector form factor of the pion (charge radius, curvature and $\bar{\ell }_6$). Regarding this low-energy constant two $N_\mathrm{f}=2$ calculations are published works without a red tag; we thus arrive at the estimate

69 $$\begin{aligned} \bar{\ell }_6\big |_{N_\mathrm{f}=2}=15.1(1.2) \end{aligned}$$

which is represented as a grey band in the last panel of Fig. 10. Here we plead with the reader to cite [238, 257] when using this number.

The experimental information concerning the charge radius is excellent and the curvature is also known very accurately, based on $e^+e^-$ data and dispersion theory. The vector form factor calculations thus present an excellent testing ground for the lattice methodology. The table shows that most of the available lattice results pass the test. There is, however, one worrisome point. For $\bar{\ell }_6$ the agreement seems less convincing than for the charge radius, even though the two quantities are closely related. So far we have no explanation, but we urge the groups to pay special attention to this point. Similarly, the bottom part of Table 16 collects the results obtained for the scalar form factor of the pion and the combination $\bar{\ell }_1-\bar{\ell }_2$ that is extracted from it.

Perhaps the most important physics result of this section is that the lattice simulations confirm the approximate validity of the Gell-Mann–Oakes–Renner formula and show that the square of the pion mass indeed grows in proportion to $m_{ud}$. The formula represents the leading term of the chiral perturbation series and necessarily receives corrections from higher orders. At first non-leading order, the correction is determined by the effective coupling constant $\bar{\ell }_3$. The results collected in Table 15 and in the top panel of Fig. 10 show that $\bar{\ell }_3$ is now known quite well. They corroborate the conclusion drawn already in Ref. [278]: the lattice confirms the estimate of $\bar{\ell }_3$ derived in [58]. In the graph of $M_\pi ^2$ versus $m_{ud}$, the values found on the lattice for $\bar{\ell }_3$ correspond to remarkably little curvature: the Gell-Mann–Oakes–Renner formula represents a reasonable first approximation out to values of $m_{ud}$ that exceed the physical value by an order of magnitude.

As emphasised by Stern and collaborators [279–281], the analysis in the framework of $\chi$PT is coherent only if (i) the leading term in the chiral expansion of $M_\pi ^2$ dominates over the remainder and (ii) the ratio $m_{s}/m_{ud}$ is close to the value 25.6 that follows from Weinberg’s leading-order formulae. In order to investigate the possibility that one or both of these conditions might fail, the authors proposed a more general framework, referred to as “Generalised $\chi$PT”, which includes $\chi$PT as a special case. The results found on the lattice demonstrate that QCD does satisfy both of the above conditions—in the context of QCD, the proposed generalisation of the effective theory does not appear to be needed. There is a modified version, however, referred to as “Resummed $\chi$PT” [282], which is motivated by the possibility that the Zweig-rule violating couplings $L_4$ and $L_6$ might be larger than expected. The available lattice data do not support this possibility, but they do not rule it out either (see Sect. 5.2.4 for details).

### SU(3) low-energy constants 

#### Quark-mass dependence of pseudoscalar masses and decay constants

In the isospin limit, the relevant SU(3) formulae take the form [56]

70 $$\begin{aligned}&M_\pi ^2 \mathop {=}\limits ^{\mathrm{NLO}}2B_0m_{ud} \left\{ 1+\mu _\pi -\frac{1}{3}\mu _\eta +\frac{B_0}{F_0^2} \right. \nonumber \\&\quad \times \left. \left[ 16m_{ud}(2L_8-L_5)+16(m_{s}+2m_{ud})(2L_6-L_4)\right] \right\} \; \,\nonumber \\&M_{K}^2 \mathop {=}\limits ^{\mathrm{NLO}}B_0(m_{s}+m_{ud}) \left\{ 1+\frac{2}{3}\mu _\eta +\frac{B_0}{F_0^2}\right. \nonumber \\&\quad \times \left. \left[ 8(m_{s}+m_{ud})(2L_8\!-\!L_5)+16(m_{s}+2m_{ud}) (2L_6\!-\!L_4)\right] \right\} \;\,\quad \nonumber \\&F_\pi \mathop {=}\limits ^{\mathrm{NLO}}F_0 \left\{ 1\!-\!2\mu _\pi \!-\!\mu _K\!+\!\frac{B_0}{F_0^2}\left[ 8m_{ud}L_5\!+\!8(m_{s}\!+\!2m_{ud})L_4\right] \right\} \;\, \\&F_K\mathop {=}\limits ^{\mathrm{NLO}}F_0 \left\{ 1-\frac{3}{4}\mu _\pi -\frac{3}{2}\mu _K-\frac{3}{4}\mu _\eta +\frac{B_0}{F_0^2}\right. \nonumber \\&\quad \times \left. \left[ 4(m_{s}+m_{ud})L_5+8(m_{s}+2m_{ud})L_4\right] \right\} \;\,\nonumber \end{aligned}$$

where $m_{ud}$ is the common up and down quark mass (which may be different from the one in the real world), and $B_0=\Sigma _0/F_0^2$, $F_0$ denote the condensate parameter and the pseudoscalar decay constant in the SU(3) chiral limit, respectively. In addition, we use the notation

71 $$\begin{aligned} \mu _P=\frac{M_P^2}{32\pi ^2F_0^2} \ln \!\left( \frac{M_P^2}{\mu ^2}\right) . \end{aligned}$$

At the order of the chiral expansion used in these formulae, the quantities $\mu _\pi$, $\mu _K$, $\mu _\eta$ can equally well be evaluated with the leading-order expressions for the masses,

72 $$\begin{aligned}&M_\pi ^2\mathop {=}\limits ^{\mathrm{LO}}2B_0\,m_{ud},\quad M_K^2\mathop {=}\limits ^{\mathrm{LO}}B_0(m_{s} + m_{ud}),\nonumber \\&\quad M_\eta ^2\mathop {=}\limits ^{\mathrm{LO}}{\frac{2}{3}}B_0(2m_{s} + m_{ud}). \end{aligned}$$

Throughout, $L_i$ denotes the renormalised low-energy constant/coupling (LEC) at scale $\mu$, and we adopt the convention which is standard in phenomenology, $\mu =770\,{\mathrm {MeV}}$. The normalisation used for the decay constants is specified in footnote 16.

#### Charge radius

The SU(3) formula for the slope of the pion vector form factor reads [152]

73 $$\begin{aligned} {\langle }r^2{\rangle }_V^\pi&\mathop {=}\limits ^{\mathrm{LO}} -\frac{1}{32\pi ^2F_0^2} \left\{ 3+2\ln \left( \frac{M_\pi ^2}{\mu ^2}\right) +\ln \left( \frac{M_K^2}{\mu ^2}\right) \right\} \nonumber \\&+\,\frac{12L_9}{F_0^2}, \end{aligned}$$

while the expression ${\langle }r^2\rangle _S^{\mathrm {oct}}$ for the octet part of the scalar radius does not contain any NLO low-energy constant at the one-loop order [152] (cf. 5.1.5 for the situation in SU(2)).

#### Partially quenched formulae

The term “partially quenched QCD” is used in two ways. For heavy quarks ($c,b$ and sometimes $s$) it usually means that these flavours are included in the valence sector, but not into the functional determinant. For the light quarks ($u,d$ and sometimes $s$) it means that they are present in both the valence and the sea sector of the theory, but with different masses (e.g. a series of valence quark masses is evaluated on an ensemble with a fixed sea quark mass).

The program of extending the standard (unitary) SU(3) theory to the (second version of) “partially quenched QCD” has been completed at the two-loop (NNLO) level for masses and decay constants [283]. These formulae tend to be complicated, with the consequence that a state-of-the-art analysis with $O(2000)$ bootstrap samples on $O(20)$ ensembles with $O(5)$ masses each [and hence $O(200'000)$ different fits] will require significant computational resources for the global fits. For an up-to-date summary of recent developments in Chiral Perturbation Theory relevant to lattice QCD we refer to [284].

The theoretical underpinning of how “partial quenching” is to be treated in the (properly extended) chiral framework is given in [285]. Specifically for partially quenched QCD with staggered quarks it is shown that a transfer matrix can be constructed which is not Hermitian but bounded, and can thus be used to construct correlation functions in the usual way.

#### Lattice determinations

To date, there are three comprehensive SU(3) papers with results based on lattice QCD with $N_\mathrm{f}= 2 + 1$ dynamical flavours [15, 19, 79], and one more with results based on $N_\mathrm{f}= 2 + 1 + 1$ dynamical flavours [156]. It is an open issue whether the data collected at $m_{s} \simeq m_{s}^\mathrm{phys}$ allow for an unambiguous determination of SU(3) low-energy constants (cf. the discussion in [79]). To make definite statements one needs data at considerably smaller $m_{s}$, and so far only MILC has some [15]. We are aware of a few papers with a result on one SU(3) low-energy constant each [78, 166, 253, 286] which we list for completeness. Some particulars of the computations are listed in Table 17.

**Table 17:** Lattice results for the low-energy constants $F_0$, $B_0$ and $\Sigma _0\equiv F_0^2B_0$, which specify the effective SU(3) Lagrangian at leading order (MeV units). The ratios $F/F_0$, $B/B_0$, $\Sigma /\Sigma _0$, which compare these with their SU(2) counterparts, indicate the strength of the Zweig-rule violations in these quantities (in the large-$N_{c}$ limit, they tend to unity). Numbers in slanted fonts are calculated by us, from the information given in the quoted references

	Ref.	$N_\mathrm{f}$	Publication status	Chiral extrapolation	Continuum extrapolation	Finite volume	Renormalisation	$F_0$	$F/F_0$	$B/B_0$
JLQCD/TWQCD 10	[252]	3	A					71 (3) (8)		
MILC 10	[159]	$2+1$	C					80.3 (2.5) (5.4)		
MILC 09A	[37]	$2+1$	C					78.3 (1.4) (2.9)	*1.104 (3) (41)*	*1.21 (4)* $$\left( {\begin{array}{c}+5\\ -6\end{array}}\right) $$
MILC 09	[15]	$2+1$	A						1.15 (5) $$\left( {\begin{array}{c}+13\\ -03\end{array}}\right) $$	*1.15 (16)* $$\left( {\begin{array}{c}+39\\ -13\end{array}}\right) $$
PACS-CS 08	[19]	$2+1$	A					83.8 (6.4)	1.078 (44)	1.089 (15)
RBC/UKQCD 08	[79]	$2+1$	A					66.1 (5.2)	1.229 (59)	1.03 (05)

	Ref.	$N_\mathrm{f}$	Publication status	Chiral extrapolation	Continuum extrapolation	Finite volume	Renormalisation	$\Sigma _0^{1/3}$	$\Sigma /\Sigma _0$	
JLQCD/TWQCD 10	[252]	3	A					214 (6) (24)	*1.31 (13) (52)*	
MILC 09A	[37]	$2+1$	C					245 (5) (4) (4)	*1.48 (9) (8) (10)*	
MILC 09	[15]	$2+1$	A					242 (9) $$\left( {\begin{array}{c}+05\\ -17\end{array}}\right) $$ (4)	1.52 (17) $$\left( {\begin{array}{c}+38\\ -15\end{array}}\right) $$	
PACS-CS 08	[19]	$2+1$	A					290 (15)	1.245 (10)	
RBC/UKQCD 08	[79]	$2+1$	A						1.55 (21)	

Results for the SU(3) low-energy constants of leading order are found in Table 17 and analogous results for some of the effective coupling constants that enter the chiral SU(3) Lagrangian at NLO are collected in Table 18. From PACS-CS [19] only those results are quoted which have been *corrected* for finite-size effects (misleadingly labelled “w/FSE” in their tables). For staggered data our colour-coding rule states that $M_\pi$ is to be understood as $M_\pi ^\mathrm{RMS}$. The rating of [15, 159] is based on the information regarding the RMS masses given in [37].

**Table 18:** Low-energy constants that enter the effective SU(3) Lagrangian at NLO (running scale $\mu =770\,{\mathrm {MeV}}$—the values in [15, 37, 56, 156, 159] are evolved accordingly). The MILC 10 entry for $L_6$ is obtained from their results for $2L_6 - L_4$ and $L_4$ (and similarly for other entries in slanted fonts). The JLQCD 08A result [which is for $\ell _5(770\,{\mathrm {MeV}})$ despite the paper saying $L_{10}(770\,{\mathrm {MeV}})$] has been converted to $L_{10}$ with the standard one-loop formula, assuming that the difference between $\bar{\ell }_5(m_{s} = m_{s}^\mathrm{phys})$ [needed in the formula] and $\bar{\ell }_5(m_{s} = \infty )$ [computed by JLQCD] can be ignored

	Ref.	$N_\mathrm{f}$	Publication status	Chiral extrapolation	Continuum extrapolation	Finite volume	$10^3L_4$	$10^3L_6$	$10^3 (2L_6-L_4)$
HPQCD 13A	[156]	$2+1+1$	A				0.09 (34)	0.16 (20)	0.22 (17)
JLQCD/TWQCD 10A	[252]	3	A					0.03 (7) (17)	
MILC 10	[159]	$2+1$	C				$-$0.08 (22) $$\left( {\begin{array}{c}+57\\ -33\end{array}}\right) $$	$-$ *0.02 (16)* $$\left( {\begin{array}{c}+33\\ -21\end{array}}\right) $$	0.03 (24) $$\left( {\begin{array}{c}+32\\ -27\end{array}}\right) $$
MILC 09A	[37]	$2+1$	C				0.04 (13) (4)	0.07 (10) (3)	0.10 (12) (2)
MILC 09	[15]	$2+1$	A				0.1 (3) $$\left( {\begin{array}{c}+3\\ -1\end{array}}\right) $$	0.2 (2)$$\left( {\begin{array}{c}+2\\ -1\end{array}}\right) $$	0.3 (1) $$\left( {\begin{array}{c}+2\\ -3\end{array}}\right) $$
PACS-CS 08	[19]	$2+1$	A				$-$0.06 (10) (–)	*0.02 (5) (–)*	0.10 (2) (–)
RBC/UKQCD 08	[79]	$2+1$	A				0.14 (8) (–)	0.07 (6) (–)	0.00 (4) (–)
Bijnens 11	[284]						0.75 (75)	0.29 (85)	$-$ *0.17 (1.86)*
Gasser 85	[56]						$-$0.3 (5)	$-$0.2 (3)	$-$ *0.1 (8)*

	Ref.	$N_\mathrm{f}$	Publication status	Chiral extrapolation	Continuum extrapolation	Finite volume	$10^3L_5$	$10^3L_8$	$10^3 (2L_8 - L_5)$
HPQCD 13A	[156]	$2+1+1$	A				1.19 (25)	0.55 (15)	$-$0.10 (20)
MILC 10	[159]	$2+1$	C				0.98 (16) $$\left( {\begin{array}{c}+28\\ -41\end{array}}\right) $$	*0.42 (10)* $$\left( {\begin{array}{c}+27\\ -23\end{array}}\right) $$	$-$0.15 (11) $$\left( {\begin{array}{c}+45\\ -19\end{array}}\right) $$
MILC 09A	[37]	$2+1$	C				0.84 (12) (36)	0.36 (5) (7)	$-$0.12 (8) (21)
MILC 09	[15]	$2+1$	A				1.4 (2) $$\left( {\begin{array}{c}+2\\ -1\end{array}}\right) $$	0.8 (1) (1)	0.3 (1) (1)
PACS-CS 08	[19]	$2+1$	A				1.45 (7) (–)	*0.62 (4) (–)*	$-$0.21 (3) (–)
RBC/UKQCD 08	[79]	$2+1$	A				0.87 (10) (–)	0.56 (4) (–)	0.24 (4) (–)
Bijnens 11	[284]						0.58 (13)	0.18 (18)	$-$ *0.22 (38)*
Gasser 85	[56]						1.4 (5)	0.9 (3)	*0.4 (8)*

	Ref.	$N_\mathrm{f}$	Publication status	Chiral extrapolation	Continuum extrapolation	Finite volume	$10^3L_5$	$10^3L_9$	$10^3L_{10}$
RBC/UKQCD 09	[287]	$2+1$	A						$-$5.7 (11) (07)
RBC/UKQCD 08A	[253]	$2+1$	A					3.08 (23) (51)	
NPLQCD 06	[166]	$2+1$	A				1.42 (2) $$\left( {\begin{array}{c}+18\\ -54\end{array}}\right) $$		
JLQCD 08A	[286]	2	A						$-$5.2 (2) $$\left( {\begin{array}{c}+5\\ -3\end{array}}\right) $$
Bijnens 11	[284]						0.58 (13)		
Bijnens 02	[288]							5.93 (43)	
Davier 98	[289]								$-$5.13 (19)
Gasser 85	[56]						1.4 (5)	6.9 (7)	$-$5.5 (7)

**Fig. 11:**
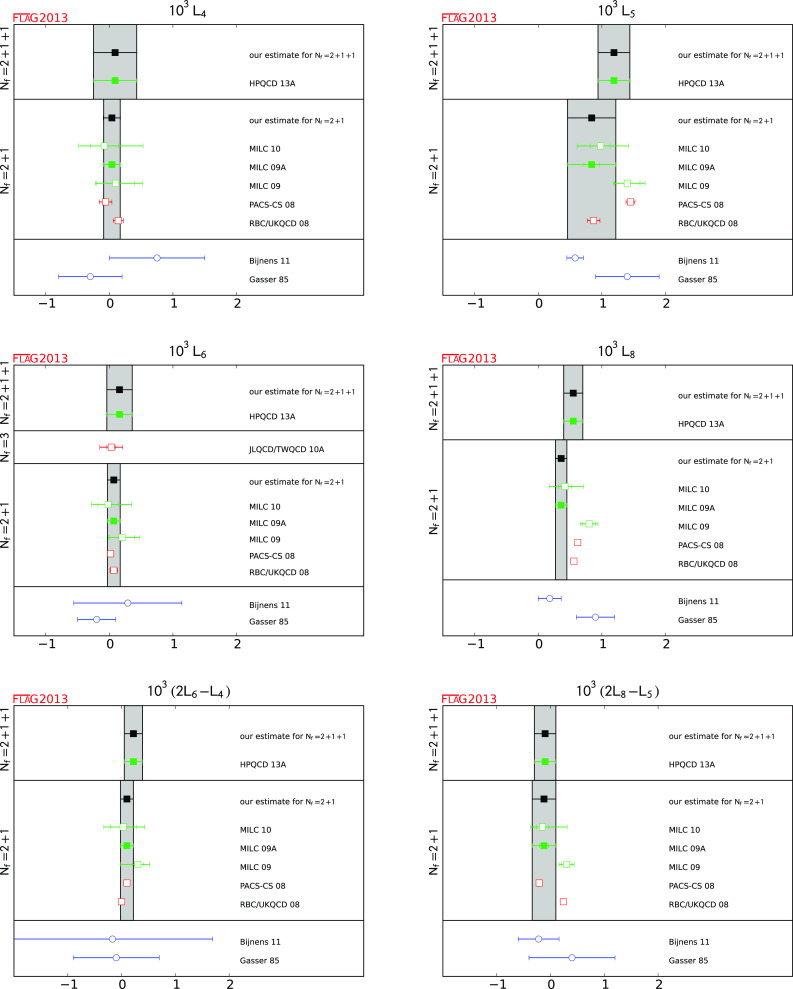
Low-energy constants that enter the effective SU(3) Lagrangian at NLO. The *grey bands* and *black dots* labelled as “our estimate” coincide with the results of MILC 09A [37] for $N_\mathrm{f}=2+1$ and HPQCD 13A [156] for $N_\mathrm{f}=2+1+1$, respectively

A graphical summary of the lattice results for the coupling constants $L_4$, $L_5$, $L_6$ and $L_8$, which determine the masses and the decay constants of the pions and kaons at NLO of the chiral SU(3) expansion, is displayed in Fig. 11, along with the two phenomenological determinations quoted in the above tables. The overall consistency seems fairly convincing. In spite of this apparent consistency, there is a point which needs to be clarified as soon as possible. Some collaborations (RBC/UKQCD and PACS-CS) find that they are having difficulties in fitting their partially quenched data to the respective formulae for pion masses above $\simeq$400 MeV. Evidently, this indicates that the data are stretching the regime of validity of these formulae. To date it is, however, not clear which subset of the data causes the troubles, whether it is the unitary part extending to too large values of the quark masses or whether it is due to $m^\mathrm{val}/m^\mathrm{sea}$ differing too much from one. In fact, little is known, in the framework of partially quenched $\chi$PT, about the *shape* of the region of applicability in the $m^\mathrm{val}$ versus $m^\mathrm{sea}$ plane for fixed $N_\mathrm{f}$. This point has also been emphasised in [244].

To date only the computations MILC 09A [37] (as an obvious update of MILC 09) and HPQCD 13A [156] are free of red tags. Since they use different $N_\mathrm{f}$ (in the former case $N_\mathrm{f}=2+1$, in the latter case $N_\mathrm{f}=2+1+1$) we stay away from averaging them. Hence the situation remains unsatisfactory in the sense that for each $N_\mathrm{f}$ only a single determination of high standing is available. Accordingly, we stay with the recommendation to use the results of MILC 09A [37] and HPQCD 13A [156] for $N_\mathrm{f}=2+1$ and $N_\mathrm{f}=2+1+1$, respectively, as given in Table 18. These numbers are shown as grey bands in Fig. 11.

In the large-$N_{c}$ limit, the Zweig rule becomes exact, but the quarks have $N_{c}=3$. The work done on the lattice is ideally suited to disprove or confirm the approximate validity of this rule for QCD. Two of the coupling constants entering the effective SU(3) Lagrangian at NLO disappear when $N_{c}$ is sent to infinity: $L_4$ and $L_6$. The upper part of Table 18 and the left panels of Fig. 11 show that the lattice results for these are quite coherent. At the scale $\mu =M_\rho$, $L_4$ and $L_6$ are consistent with zero, indicating that these constants do approximately obey the Zweig rule. As mentioned above, the ratios $F/F_0$, $B/B_0$ and $\Sigma /\Sigma _0$ also test the validity of this rule. Their expansion in powers of $m_{s}$ starts with unity and the contributions of first order in $m_{s}$ are determined by the constants $L_4$ and $L_6$, but they also contain terms of higher order. Apart from measuring the Zweig-rule violations, an accurate determination of these ratios will thus also allow us to determine the range of $m_{s}$ where the first few terms of the expansion represent an adequate approximation. Unfortunately, at present, the uncertainties in the lattice data on these ratios are too large to draw conclusions, both concerning the relative size of the subsequent terms in the chiral perturbation series and concerning the magnitude of the Zweig-rule violations. The data seem to confirm the *paramagnetic inequalities* [281], which require $F/F_0>1$ and $\Sigma /\Sigma _0>1$, and it appears that the ratio $B/B_0$ is also larger than unity, but the numerical results need to be improved before further conclusions can be drawn.

In principle, the matching formulae in [56] can be used to calculate^25^ the SU(2) couplings $\bar{l}_i$ from the SU(3) couplings $L_j$. This procedure, however, yields less accurate results than a direct determination within SU(2), as it relies on the expansion in powers of $m_{s}$, where the omitted higher-order contributions generate comparatively large uncertainties. We plead with every collaboration performing $N_\mathrm{f}=2+1$ simulations to *directly* analyse their data in the SU(2) framework. In practice, lattice simulations are performed at values of $m_{s}$ close to the physical value and the results are then corrected for the difference of $m_{s}$ from its physical value. If simulations with more than one value of $m_{s}$ have been performed, this can be done by interpolation. Alternatively one can use the technique of *reweighting* (for a review see e.g. [290]) to shift $m_{s}$ to its physical value.

## Kaon $B$-parameter $B_K$

### Indirect CP-violation and $\epsilon _{K}$

The mixing of neutral pseudoscalar mesons plays an important role in the understanding of the physics of CP-violation. In this section we will only focus on $K^0 - \bar{K}^0$ oscillations, which probe the physics of indirect CP-violation. We collect here the basic formulae; for extended reviews on the subject see, among others, Refs. [291–293]. Indirect CP-violation arises in $K_L \rightarrow \pi \pi$ transitions through the decay of the $\hbox {CP}=+1$ component of $K_L$ into two pions (which are also in a $\hbox {CP}=+1$ state). Its measure is defined as

74 $$\begin{aligned} \epsilon _{K} = \dfrac{\mathcal{A} [ K_L \rightarrow (\pi \pi )_{I=0}]}{\mathcal{A} [ K_S \rightarrow (\pi \pi )_{I=0}]}, \end{aligned}$$

with the final state having total isospin zero. The parameter $\epsilon _{K}$ may also be expressed in terms of $K^0 - \bar{K}^0$ oscillations. In particular, to lowest order in the electroweak theory, the contribution to these oscillations arises from so-called box diagrams, in which two $W$-bosons and two “up-type” quarks (i.e. up, charm, top) are exchanged between the constituent down and strange quarks of the $K$-mesons. The loop integration of the box diagrams can be performed exactly. In the limit of vanishing external momenta and external quark masses, the result can be identified with an effective four-fermion interaction, expressed in terms of the “effective Hamiltonian”

75 $$\begin{aligned} \mathcal{H}_\mathrm{eff}^{\Delta S = 2} = \frac{G_\mathrm{{F}}^2 M_{{W}}^2}{16\pi ^2} \mathcal{F}^0 Q^{\Delta S=2} + \hbox {h.c.}. \end{aligned}$$

In this expression, $G_\mathrm{{F}}$ is the Fermi coupling, $M_{{W}}$ the $W$-boson mass, and

76 $$\begin{aligned} Q^{\Delta S=2}&= \left[ \bar{s}\gamma _\mu (1-\gamma _5)d\right] \left[ \bar{s}\gamma _\mu (1-\gamma _5)d\right] \nonumber \\&\equiv O_\mathrm{VV+AA}-O_\mathrm{VA+AV} \end{aligned}$$

is a dimension-six, four-fermion operator. The function $\mathcal{F}^0$ is given by

77 $$\begin{aligned} \mathcal{F}^0 = \lambda _{c}^2 S_0(x_{c}) + \lambda _{t}^2 S_0(x_{t}) + 2 \lambda _{c} \lambda _{t} S_0(x_{c},x_{t}), \end{aligned}$$

where $\lambda _{a} = V^*_{as} V_{ad}$, and $a=c,t$ denotes a flavour index. The quantities $S_0(x_{c}),\,S_0(x_{t})$ and $S_0(x_{c},x_{t})$ with $x_{c}=m_{c}^2/M_{{W}}^2$, $x_{t}=m_{t}^2/M_{{W}}^2$ are the Inami–Lim functions [294], which express the basic electroweak loop contributions without QCD corrections. The contribution of the up quark, which is taken to be massless in this approach, has been taken into account by imposing the unitarity constraint $\lambda _{u} + \lambda _{c} + \lambda _{t} = 0$. For future reference we note that the dominant contribution comes from the term $\lambda _{t}^2 S_0(x_{t})$. This factor is proportional to $|V_{cb}|^4$ if one enforces the unitarity of the CKM matrix. The dependence on a high power of $V_{cb}$ is important from a phenomenological point of view, because it implies that uncertainties in $V_{cb}$ are magnified when considering $\epsilon _K$.

When strong interactions are included, $\Delta {S}=2$ transitions can no longer be discussed at the quark level. Instead, the effective Hamiltonian must be considered between mesonic initial and final states. Since the strong coupling constant is large at typical hadronic scales, the resulting weak matrix element cannot be calculated in perturbation theory. The operator product expansion (OPE) does, however, factorise long- and short-distance effects. For energy scales below the charm threshold, the $K^0-\bar{K}^0$ transition amplitude of the effective Hamiltonian can be expressed as

78 $$\begin{aligned}&{\langle }\bar{K}^0 \vert \mathcal{H}_\mathrm{eff}^{\Delta S = 2} \vert K^0{\rangle } = \frac{G_\mathrm{{F}}^2 M_{{W}}^2}{16 \pi ^2} \left[ \lambda _{c}^2 S_0(x_{c}) \eta _1 \, + \, \lambda _{t}^2 S_0(x_{t}) \eta _2\right. \nonumber \\&\qquad \left. + \, 2 \lambda _{c} \lambda _{t} S_0(x_{c},x_{t}) \eta _3\right] \nonumber \\&\qquad \times \left( \frac{\bar{g}(\mu )^2}{4\pi }\right) ^{-\gamma _0/(2\beta _0)} \exp \left\{ \int _0^{\bar{g}(\mu )} \, \hbox {d}g \, \left( \frac{\gamma (g)}{\beta (g)} + \frac{\gamma _0}{\beta _0g} \right) \right\} \nonumber \\&\qquad \times {\langle }\bar{K}^0 \vert Q^{\Delta S=2}_{R} (\mu ) \vert K^0 {\rangle } + \mathrm{h.c.}, \end{aligned}$$

where $$\bar{g}(\mu )$$ and $$Q^{\Delta S=2}_{R}(\mu )$$ are the renormalised gauge coupling and four-fermion operator in some renormalisation scheme. The factors $$\eta _1, \eta _2$$ and $$\eta _3$$ depend on the renormalised coupling $$\bar{g}$$, evaluated at the various flavour thresholds $$m_{t}, m_{b}, m_{c}$$ and $$ M_{{W}}$$, as required by the OPE and RG-running procedure that separates high- and low-energy contributions. Explicit expressions can be found in [292] and references therein, except that $$\eta _1$$ and $$\eta _3$$ have been recently calculated to NNLO in Refs. [295] and [296], respectively. We follow the same conventions for the RG-equations as in Ref. [292]. Thus the Callan–Symanzik function and the anomalous dimension $$\gamma (\bar{g})$$ of $$Q^{\Delta S=2}$$ are defined by

79 $$\begin{aligned} \dfrac{\hbox {d} \bar{g}}{\hbox {d} \ln \mu } = \beta (\bar{g}),\qquad \dfrac{\hbox {d} Q^{\Delta S=2}_{R}}{\hbox {d} \ln \mu } = -\gamma (\bar{g})\,Q^{\Delta S=2}_{R}, \end{aligned}$$

with perturbative expansions

80 $$\begin{aligned} \beta (g)&= -\beta _0 \dfrac{g^3}{(4\pi )^2} - \beta _1\dfrac{g^5}{(4\pi )^4} - \cdots \\ \gamma (g)&= \gamma _0 \dfrac{g^2}{(4\pi )^2} + \gamma _1\dfrac{g^4}{(4\pi )^4} + \cdots .\nonumber \end{aligned}$$

We stress that $\beta _0, \beta _1$ and $\gamma _0$ are universal, i.e. scheme-independent. $K^0-\bar{K}^0$ mixing is usually considered in the naive dimensional regularisation (NDR) scheme of ${\overline{\mathrm{MS}}}$, and below we specify the perturbative coefficient $\gamma _1$ in that scheme:

81 $$\begin{aligned}&\beta _0 = \left\{ \frac{11}{3}N_{c}-\frac{2}{3}N_\mathrm{f}\right\} , \nonumber \\&\beta _1 =\left\{ \frac{34}{3}N_{c}^2-N_\mathrm{f}\left( \frac{13}{3}N_{c}-\frac{1}{N_{c}}\right) \right\} , \nonumber \\&\gamma _0 = \frac{6(N_{c}-1)}{N_{c}}, \nonumber \\&\gamma _1 = \frac{N_{c}-1}{2N_{c}} \left\{ -21 + \frac{57}{N_{c}} -\frac{19}{3}N_{c} + \frac{4}{3}N_\mathrm{f}\right\} . \end{aligned}$$

Note that for QCD the above expressions must be evaluated for $N_{c}=3$ colours, while $N_\mathrm{f}$ denotes the number of active quark flavours. As already stated, Eq. (78) is valid at scales below the charm threshold, after all heavier flavours have been integrated out, i.e. $N_\mathrm{f}= 3$.

In Eq. (78), the terms proportional to $\eta _1,\,\eta _2$ and $\eta _3$, multiplied by the contributions containing $\bar{g}(\mu )^2$, correspond to the Wilson coefficient of the OPE, computed in perturbation theory. Its dependence on the renormalisation scheme and scale $\mu$ is cancelled by that of the weak matrix element ${\langle }\bar{K}^0 \vert Q^{\Delta S=2}_{R} (\mu ) \vert K^0 \rangle$. The latter corresponds to the long-distance effects of the effective Hamiltonian and must be computed non-perturbatively. For historical, as well as technical reasons, it is convenient to express it in terms of the $B$-parameter $B_{{K}}$, defined as

82 $$\begin{aligned} B_{{K}}(\mu )= \frac{{\left\langle \bar{K}^0\left| Q^{\Delta S=2}_{R}(\mu )\right| K^0\right\rangle } }{ {\frac{8}{3}f_{K}^2m_{K}^2}} \, \, . \end{aligned}$$

The four-quark operator $Q^{\Delta S=2}(\mu )$ is renormalised at scale $\mu$ in some regularisation scheme, for instance, NDR-${\overline{\mathrm{MS}}}$. Assuming that $B_{{K}}(\mu )$ and the anomalous dimension $\gamma (g)$ are both known in that scheme, the renormalisation group invariant (RGI) $B$-parameter $\hat{B}_{K}$ is related to $B_{{K}}(\mu )$ by the exact formula

83 $$\begin{aligned} \hat{B}_{{K}}&= \left( \frac{\bar{g}(\mu )^2}{4\pi }\right) ^{-\gamma _0/(2\beta _0)}\nonumber \\&\times \exp \left\{ \int _0^{\bar{g}(\mu )} \, \hbox {d}g \left( \frac{\gamma (g)}{\beta (g)} + \frac{\gamma _0}{\beta _0g} \right) \right\} \, B_{{K}}(\mu ). \end{aligned}$$

At NLO in perturbation theory the above reduces to

84 $$\begin{aligned} \hat{B}_{{K}}&= \left( \frac{\bar{g}(\mu )^2}{4\pi }\right) ^{-\gamma _0/(2\beta _0)} \nonumber \\&\times \left\{ 1+\dfrac{\bar{g}(\mu )^2}{(4\pi )^2}\left[ \frac{\beta _1\gamma _0-\beta _0\gamma _1}{2\beta _0^2} \right] \right\} \, B_{{K}}(\mu ). \end{aligned}$$

To this order, this is the scale-independent product of all $\mu$-dependent quantities in Eq. (78).

Lattice QCD calculations provide results for $B_K(\mu )$. These results, however, are usually obtained in intermediate schemes other than the continuum ${\overline{\mathrm{MS}}}$ scheme used to calculate the Wilson coefficients appearing in Eq. (78). Examples of intermediate schemes are the RI/MOM scheme [297] (also dubbed the “Rome–Southampton method”) and the Schrödinger functional (SF) scheme [87], which both allow for a non-perturbative renormalisation of the four-fermion operator, using an auxiliary lattice simulation. In this way $B_K(\mu )$ can be calculated with percent-level accuracy, as described below.

In order to make contact with phenomenology, however, and in particular to use the results presented above, one must convert from the intermediate scheme to the ${\overline{\mathrm{MS}}}$ scheme or to the RGI quantity $\hat{B}_{K}$. This conversion relies on one- or two-loop perturbative matching calculations, the truncation errors in which are, for many recent calculations, the dominant source of error in $\hat{B}_{{K}}$ [25, 77, 298–300]. While this scheme-conversion error is not, strictly speaking, an error of the lattice calculation itself, it must be included in results for the quantities of phenomenological interest, namely $B_K({\overline{\mathrm{MS}}},2\,\mathrm{GeV})$ and $\hat{B}_{K}$. We note that this error can be minimised by matching between the intermediate scheme and ${\overline{\mathrm{MS}}}$ at as large a scale $\mu$ as possible (so that the coupling constant which determines the rate of convergence is minimised). Recent calculations have pushed the matching $\mu$ up to the range 3–3.5 GeV. This is possible because of the use of non-perturbative RG running determined on the lattice [25, 301]. The Schrödinger functional offers the possibility to run non-perturbatively to scales $\mu \sim M_{{W}}$ where the truncation error can be safely neglected. However, so far this has been applied only for two flavours of Wilson quarks [302].

Perturbative truncation errors in Eq. (78) also affect the Wilson coefficients $\eta _1$, $\eta _2$ and $\eta _3$. It turns out that the largest uncertainty comes from that in $\eta _1$ [295]. Although it is now calculated at NNLO, the series shows poor convergence. The net effect is that the uncertainty in $\eta _1$ is larger than that in present lattice calculations of $B_K$.

The “master formula” for $\epsilon _{K}$, which connects the experimentally observable quantity $\epsilon _{K}$ to the matrix element of $\mathcal{H}_\mathrm{eff}^{\Delta S = 2}$, is [293, 303–305]

85 $$\begin{aligned} \epsilon _{K}&= \exp (i \phi _\epsilon ) \,\, \sin (\phi _\epsilon ) \,\, \left[ \frac{\hbox {Im} [ {\langle }\bar{K}^0 \vert \mathcal{H}_\mathrm{eff}^{\Delta S = 2} \vert K^0 {\rangle }]}{\Delta m_K }\right. \nonumber \\&+\left. \rho \frac{\hbox {Im}(A_0)}{\hbox {Re}(A_0)}\right] , \end{aligned}$$

for $\lambda _{u}$ real and positive; the phase of $\epsilon _{K}$ is given by

86 $$\begin{aligned} \phi _\epsilon = \arctan \frac{\Delta m_{K}}{\Delta \Gamma _{K}/2}. \end{aligned}$$

The quantities $\Delta m_K\equiv m_{K_L}-m_{K_S}$ and $\Delta \Gamma _K\equiv \Gamma _{K_S}-\Gamma _{K_L}$ are the mass- and decay width-differences between long- and short-lived neutral Kaons, while $A_0$ is the amplitude of the Kaon decay into a two-pion state with isospin zero. The experimentally measured values of the above quantities are [74]:

87 $$\begin{aligned} \vert \epsilon _{K} \vert&= 2.228(11) \times 10^{-3}, \nonumber \\ \phi _\epsilon&= 43.52(5)^\circ , \nonumber \\ \Delta m_{K}&= 3.4839(59) \times 10^{-12}\, \mathrm{MeV}, \nonumber \\ \Delta \Gamma _{K}&= 7.3382(33) \times 10^{-12} \,\mathrm{MeV}. \end{aligned}$$

The second term in the square brackets of Eq. (85), has been discussed and estimated, e.g., in Refs. [305, 306]. It can best be thought of as $\xi + (\rho -1)\xi$, with $\xi =\mathrm{Im}(A_0)/{Re}(A_0)$. The $\xi$ term is the contribution of direct CP violation to $\epsilon _K$. Using the estimate of $\xi$ from Ref. [306] (obtained from the experimental value of $\epsilon '/\epsilon$) this gives a $\sim -6.0(1.5)\,\%$ correction.^26^

The $(\rho -1)\xi$ term arises from long-distance contributions to the imaginary part of $K^0 -\bar{K}^0$ mixing [305] [contributions which are neglected in Eq. (78)]. Using the estimate $\rho =0.6\pm 0.3$ [305], this gives a contribution of about +2 % with large errors. Overall these corrections combine to give a $(4 \pm 2)\,\%$ reduction in the prediction for $\epsilon _K$. Although this is a small correction, we note that its contribution to the error of $\epsilon _K$ is larger than that arising from the value of $B_{K}$ reported below.

### Lattice computation of $B_{{K}}$

Lattice calculations of $B_{{K}}$ are affected by the same systematic effects discussed in previous sections. However, the issue of renormalisation merits special attention. The reason is that the multiplicative renormalisability of the relevant operator $Q^{\Delta S=2}$ is lost once the regularised QCD action ceases to be invariant under chiral transformations. For Wilson fermions, $Q^{\Delta S=2}$ mixes with four additional dimension-six operators, which belong to different representations of the chiral group, with mixing coefficients that are finite functions of the gauge coupling. This complicated renormalisation pattern was identified as the main source of systematic error in earlier, mostly quenched calculations of $B_{{K}}$ with Wilson quarks. It can be bypassed via the implementation of specifically designed methods, which are either based on Ward identities [309] or on a modification of the Wilson quark action, known as twisted-mass QCD [310, 311].

An advantage of staggered fermions is the presence of a remnant $U(1)$ chiral symmetry. However, at non-vanishing lattice spacing, the symmetry among the extra unphysical degrees of freedom (tastes) is broken. As a result, mixing with other dimension-six operators cannot be avoided in the staggered formulation, which complicates the determination of the $B$-parameter. The effects of the broken taste symmetry are usually treated via an effective field theory, such as staggered Chiral Perturbation Theory (S$\chi$PT).

Fermionic lattice actions based on the Ginsparg–Wilson relation [312] are invariant under the chiral group, and hence four-quark operators such as $Q^{\Delta S=2}$ renormalise multiplicatively. However, depending on the particular formulation of Ginsparg–Wilson fermions, residual chiral symmetry breaking effects may be present in actual calculations. For instance, in the case of domain-wall fermions, the finiteness of the extra fifth dimension implies that the decoupling of modes with different chirality is not exact, which produces a residual non-zero quark mass in the chiral limit. Whether or not a significant mixing with dimension-six operators is induced as well must be investigated on a case-by-case basis.

**Fig. 12:**
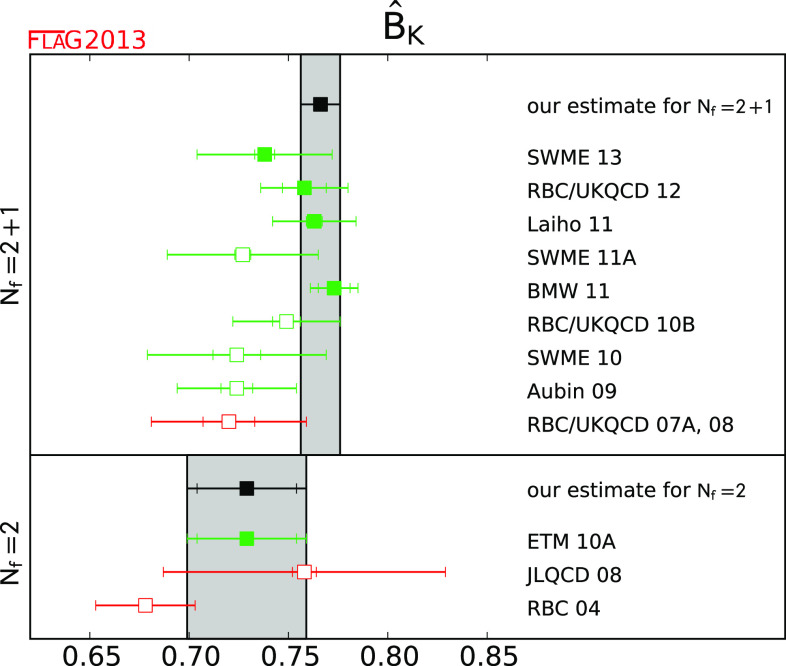
Lattice results for the renormalisation group invariant $B$-parameter (compare Table 19). The *black squares* and *grey bands* indicate our global averages (88) and (90). Our $N_\mathrm{f}=2$ estimate coincides with the ETM 10A result. The significance of the colours is explained in Sect. 2

In this section we focus on recent results for $B_{{K}}$, obtained for $N_\mathrm{f}=2$ and $2+1$ flavours of dynamical quarks. A compilation of results is shown in Table 19 and Fig. 12. An overview of the quality of systematic error studies is represented by the colour coded entries in Table 19. In Appendix B.4 we gather the simulation details and results from different collaborations, the values of the most relevant lattice parameters, and comparative tables on the various estimates of systematic errors.

**Table 19:** Results for the Kaon $B$-parameter together with a summary of systematic errors. If information about non-perturbative running is available, this is indicated in the column “running”, with details given at the bottom of the table

Collaboration	Ref.	$N_\mathrm{f}$	Publication status	Continuum extrapolation	Chiral extrapolation	Finite volume	Renormalisation	Running	$B_{{K}} (\overline{\mathrm{MS}},2\,\mathrm{GeV})$	$\hat{B}_{{K}}$
SWME 13	[316]	$2+1$	C				$^\ddagger$	–	0.539 (3) (25)	0.738 (5) (34)
RBC/UKQCD 12	[25]	$2+1$	A					$\,a$	0.554 (8) (14)$^1$	0.758 (11) (19)
Laiho 11	[77]	$2+1$	C					–	0.5572 (28) (150)	0.7628 (38) (205)$^2$
SWME 11A	[300]	$2+1$	A				$^\ddagger$	–	0.531 (3) (27)	0.727 (4) (38)
BMW 11	[301]	$2+1$	A					$\,b$	0.5644 (59) (58)	0.7727 (81) (84)
RBC/UKQCD 10B	[315]	$2+1$	A					$\,c$	0.549 (5) (26)	0.749 (7) (26)
SWME 10	[317]	$2+1$	A					–	0.529 (9) (32)	0.724 (12) (43)
Aubin 09	[298]	$2+1$	A					–	0.527 (6) (21)	0.724 (8) (29)
RBC/UKQCD 07A, 08	[79, 318]	$2+1$	A					–	0.524 (10) (28)	0.720 (13) (37)
HPQCD/UKQCD 06	[319]	$2+1$	A		$^*$			–	0.618 (18) (135)	0.83 (18)
ETM 10A	[314]	2	A					$\,d$	0.533 (18) (12)$^1$	0.729 (25) (17)
JLQCD 08	[320]	2	A					–	0.537 (4) (40)	0.758 (6) (71)
RBC 04	[313]	2	A			$^\dagger$		–	0.495 (18)	0.678 (25)$^2$
UKQCD 04	[321]	2	A			$^\dagger$		–	0.49 (13)	0.68 (18)

$^\ddagger$ The renormalisation is performed using perturbation theory at one loop, with a conservative estimate of the uncertainty

$^*$ This result has been obtained with only two “light” sea quark masses

$^\dagger$ These results have been obtained at $(M_\pi L)_\mathrm{min} > 4$ in a lattice box with a spatial extension $L < 2$ fm

$^a$ $B_K$ is renormalised non-perturbatively at a scale of 1.4 GeV in two RI/SMOM schemes for $N_\mathrm{f}= 3$ and then run to 3 GeV using a non-perturbatively determined step-scaling function. Conversion to ${\overline{\mathrm{MS}}}$ is at one-loop order at 3 GeV

$$^b$$ $$ B_K$$ is renormalised and run non-perturbatively to a scale of $$3.4\,\mathrm{GeV}$$ in the RI/MOM scheme. Non-perturbative and NLO perturbative running agrees down to scales of $$1.8\,\mathrm{GeV}$$ within statistical uncertainties of about 2 %

$^c$ $B_K$ is renormalised non-perturbatively at a scale of 2 GeV in two RI/SMOM schemes for $N_\mathrm{f}= 3$ and then run to 3 GeV using a non-perturbatively determined step-scaling function. Conversion to ${\overline{\mathrm{MS}}}$ is at one-loop order at 3 GeV. $d\,B_K$ is renormalised non-perturbatively at scales $1/a \sim 2 \div 3\,\mathrm{GeV}$ in the $N_\mathrm{f}= 2$ RI/MOM scheme. In this scheme, non-perturbative and NLO perturbative running are shown to agree from 4 GeV down 2 GeV to better than 3 % [71, 314]

$^1$ $B_{K}({\overline{\mathrm{MS}}}, 2\,\mathrm{GeV})$ is obtained from the estimate for $\hat{B}_{{K}}$ using the conversion factor 1.369

$^2$ $\hat{B}_{{K}}$ is obtained from the estimate for $B_{K}({\overline{\mathrm{MS}}}, 2\,\mathrm{GeV})$ using the conversion factor 1.369

Some of the groups whose results are listed in Table 19 do not quote results for both $B_{{K}}(\overline{\mathrm{MS}},2\,\mathrm{GeV})$—which we denote by the shorthand $B_{{K}}$ from now on—and $\hat{B}_{{K}}$. This concerns Refs. [313, 314] for $N_\mathrm{f}=2$ and [25, 77] for $2+1$ flavours. In these cases we perform the conversion ourselves by evaluating the proportionality factor in Eq. (84) at $\mu =2\,\mathrm{GeV}$, using the following procedure: For $N_\mathrm{f}=2+1$ we use the value $\alpha _\mathrm{s}(M_{{Z}})=0.1184$ from the PDG [74] and run it across the quark thresholds at $m_{b}=4.19$ GeV and $m_{c}=1.27$ GeV, and then run up in the three-flavour theory to $\mu =2\,\mathrm{GeV}$. All running is done using the four-loop RG $\beta$-function. The resulting value of $\alpha _\mathrm{s}(2\,\mathrm{GeV})$ is then used to evaluate $\hat{B}_{{K}}/B_{{K}}$ in one-loop perturbation theory, which gives $\hat{B}_{{K}}/B_{{K}}=1.369$ in the three-flavour theory.

In two-flavour QCD one can insert the updated non-perturbative estimate for the $\Lambda$-parameter by the ALPHA Collaboration [59], i.e. $\Lambda ^{(2)}=310(20)$ MeV, into the NLO expressions for $\alpha _\mathrm{s}$. The resulting value of the perturbative conversion factor $\hat{B}_K/B_K$ for $N_\mathrm{f}=2$ is then equal to 1.386. However, since the running coupling in the ${\overline{\mathrm{MS}}}$ scheme enters at several stages in the entire matching and running procedure, it is difficult to use this estimate of $\alpha _\mathrm{s}$ consistently without a partial reanalysis of the data in Refs. [313, 314]. We have therefore chosen to apply the conversion factor of 1.369 not only to results obtained for $N_\mathrm{f}=2+1$ flavours but also to the two-flavour theory (in cases where only one of $\hat{B_K}$ and $B_K$ are quoted). This is a change from the convention used in the previous edition of the FLAG review [1]. We note that the difference between 1.386 and 1.369 will produce an ambiguity of the order of 1 %, which is well below the overall uncertainties in Refs. [313, 314]. We have indicated explicitly in Table 19 in which way the conversion factor 1.369 has been applied to the results of Refs. [25, 77, 313, 314].

Note that in this section the colour code for chiral extrapolations is interpreted differently. We recall that the criteria are:

Chiral extrapolation:

$$M_{\pi ,{\mathrm {min}}}< 200$$ MeV
200 MeV $$\le M_{\pi ,{\mathrm {min}}} \le $$ 400 MeV
$$M_{\pi ,{\mathrm {min}}}> 400$$ MeV

Many calculations of $B_K$ employ partially quenched $\chi$PT, and in this case it is the mass of the valence pion which enters in chiral logarithms and leads to the most significant dependence on quark masses. Therefore, whenever a specific calculation employs partially quenched pions, the above colour code is applied with respect to the minimum valence pion mass.^27^

As before, it is assumed that the chiral extrapolation is done with at least a three-point analysis—otherwise this will be explicitly mentioned in a footnote. In case of non-degeneracies among the different pion states $M_{\pi ,{\mathrm {min}}}$ stands for a root-mean-square (RMS) pion mass.

Since the first publication of the FLAG review [1] several new or updated results for the Kaon $B$-parameter have been reported for $N_\mathrm{f}=2+1$, i.e. BMW 11 [301], SWME 11A [300], SWME 13 [316], Laiho 11 [77], and RBC/UKQCD 12 [25]. No new results for two-flavour QCD have appeared recently. There is a first, preliminary calculation with $N_\mathrm{f}=2+1+1$ [322] from the ETM collaboration. We do not include this result in the following discussion, however, because the interpretation of $B_{K}$ with active charm involves several subtleties that have yet to be addressed.^28^

We briefly discuss the main features of the most recent calculations below.

The BMW Collaboration has produced a new result for $B_{{K}}$ [301], using their ensembles of HEX-smeared, tree-level $O(a)$ improved Wilson fermions [23]. To this end the four finest lattice spacings, with $a$ ranging from 0.054 to 0.093 fm, are employed. Simulations are performed close to the physical pion mass, or even below that value (for the two largest lattice spacings). The smearing of the link variables results in a significant suppression of the effects of chiral symmetry breaking, since the coefficients multiplying the dimension-six operators of different chirality are found to be very small, in some cases even compatible with zero. The quoted value for $\hat{B}_{{K}}$ is obtained from a combined chiral and continuum extrapolation. In order to investigate the systematics associated with the chiral behaviour, several different cuts on the maximum pion mass are performed. Another important ingredient in BMW 11 [301] is the non-perturbative determination of the continuum step-scaling function for scales varying between 1.8 and 3.5 GeV. In this way, the perturbative matching to the RGI $B$-parameter can be performed at $\mu =3.5$ GeV, a value where perturbation theory at NLO is found to yield a good description of the scale dependence.

The SWME 11, 11A, 13 results [299, 300, 316] are obtained using a mixed action: HYP-smeared valence staggered quarks on the Asqtad improved, rooted staggered MILC ensembles. Compared to the previous edition of the FLAG review [1], one major update is the addition of a fourth, finer, lattice spacing. This allows for a more extensive analysis of the continuum extrapolation, leading to more reliable estimates of the associated error (which is the second-largest error at 1.1 %). A second major update, implemented only in SWME 13, is the addition of several ensembles with a range of sea-quark masses allowing a simultaneous extrapolation in $a^2$ and the sea-quark masses. A third change in SWME 13 is the use of larger volumes. Other updates include the use of correlated fits in the chiral extrapolation, the inclusion of finite-volume corrections in the chiral fits, and a significant reduction in statistical errors due to the use of an order of magnitude more sources on each lattice. The dominant error remains that from the use of one-loop perturbative matching between lattice and ${\overline{\mathrm{MS}}}$ schemes. This error is estimated conservatively assuming a missing two-loop matching term of size $1\times \alpha (1/a)^2$, i.e. with no factors of $1/(4\pi )$ included. The other methods for estimating this error described earlier in this review lead to smaller estimates [323]. This procedure is, in this review, deemed conservative enough to merit inclusion in the global average described below. The resulting matching error is 4.4 %.

The Laiho 11 result [77] uses a mixed action, with HYP-smeared domain-wall valence quarks on the Asqtad MILC ensembles. Compared to the earlier result obtained by this collaboration (Aubin 09 [298]), the main improvement consists in the implementation of an RI/MOM scheme based on non-exceptional momenta in the non-perturbative renormalisation of $B_{{K}}$, as well as the addition of a third lattice spacing. The largest error is still the matching factor between the lattice and ${\overline{\mathrm{MS}}}$ schemes. This error is 2.4 % out of a total quoted error of 2.8 %. The present calculation uses five additional ensembles over that of the previous edition of the FLAG review [1], leading to a reduction of the chiral/continuum-extrapolation error and to the statistical error.

The RBC/UKQCD Collaboration employ domain-wall fermions to determine $B_{{K}}$. The main feature of their latest update, Ref. [25], is the addition of two ensembles with unitary pion masses as low as 171 MeV and a minimum partially quenched pion mass of 143 MeV. In order to keep the numerical effort of simulating near-physical pion masses at a manageable level, the new ensembles are generated at a larger lattice spacing. Moreover, in order to control the larger residual chiral symmetry-breaking effects which are incurred on coarser lattices, a modified fermion action, the Dislocation Suppressing Determinant Ratio (DSDR) [324–327], is used in the simulations. As in their earlier publication [315], RBC/UKQCD employ non-perturbative renormalisation factors computed for a variety of RI/MOM schemes with non-exceptional momenta. Owing to the addition of ensembles with larger lattice spacing, the matching between lattice regularisation and the intermediate RI/MOM schemes is performed at the lower scale of 1.4 GeV. When combined with the non-perturbative determinations of the continuum step-scaling functions, the perturbative conversion to the ${\overline{\mathrm{MS}}}$ or RGI schemes can be done at $\mu =3\,\mathrm{GeV}$. The use of near-physical valence pion masses at a spatial volume of $L\approx 4.6$ fm implies a rather small value of $M_{\pi ,\mathrm{min}}L\approx 3.3$. However, the entire set of results collected in Refs. [25, 315] comprises several volumes with $L>2.7$ fm. The combined analysis of all data should allow for a reliable determination of $B_{{K}}$ with controlled finite-volume effects. It is noted in Ref. [25] that the inclusion of the lighter pion masses essentially halves the uncertainty in $B_{{K}}$ due to the chiral/continuum extrapolation. The largest systematic uncertainty remains the perturbative truncation error of 2.1 %. As regards the effects of residual chiral symmetry breaking induced by the finite extent of the fifth dimension in the domain-wall fermion formulation, it is noted in Ref. [328] that the mixing of $Q^{\Delta {S}=2}$ with operators of opposite chirality is negligibly small.

Summarizing the new developments, one must note that the biggest improvements since the previous edition of the FLAG review [1] concern the chiral extrapolation and the issue of renormalisation. Ensembles at near-physical pion masses have significantly reduced the uncertainty associated with chiral fits, while non-perturbative running is about to become routine. One must realise that, despite this improvement, perturbative matching is still applied only at moderately large scales. Most collaborations therefore identify the largest uncertainty to arise from neglecting higher orders in the perturbative relation to the RGI or ${\overline{\mathrm{MS}}}$ schemes.

We now describe our procedure for obtaining global averages. The rules of Sect. 2.1 stipulate that results which are free of red tags and are published in a refereed journal may enter an average. Papers which at the time of writing are still unpublished but are obvious updates of earlier published results can also be taken into account.

In the previous edition of the FLAG review [1] the results by SWME were excluded from the average, since the renormalisation factors were estimated in one-loop perturbation theory only. However, in this review such calculations are included as long as the estimate of the matching error is sufficiently conservative. Thus the result of SWME 13 [316] (which is an update of the earlier published calculations of Refs. [299, 300]) now qualifies for inclusion, despite the fact that non-perturbative information on the renormalisation factors is not available. Reference [77], Laiho 11 has appeared only as conference proceedings, but since it extends the study of Ref. [298] it will be included in our average.

Thus, for $N_\mathrm{f}=2+1$ our global average is based on the results of BMW 11 [301], SWME 13 [316], Laiho 11 [77] and RBC/UKQCD 12 [25]. Our procedure is as follows: in a first step statistical and systematic errors of each individual result for the RGI $B$-parameter, $\hat{B}_{{K}}$, are combined in quadrature. Next, a weighted average is computed from the set of results. For the final error estimate we take correlations between different collaborations into account. To this end we note that we consider the statistical and finite-volume errors of SWME 13 and Laiho 11 to be correlated, since both groups use the Asqtad ensembles generated by the MILC Collaboration. Laiho 11 and RBC/UKQCD 12A both use domain-wall quarks in the valence sector and also employ similar procedures for the non-perturbative determination of matching factors. Hence, we treat the quoted renormalisation and matching uncertainties by the two groups as correlated. After constructing the global covariance matrix according to Schmelling [16], we arrive at

88 $$\begin{aligned} N_\mathrm{f}=2+1: \quad \hat{B}_{{K}} = 0.7661(99), \end{aligned}$$

with a reduced $\chi ^2$-value of 0.387. The error is dominated by systematic uncertainties.^29^

By applying the NLO conversion factor $\hat{B}_{{K}}/B_{{K}}^{\overline{\mathrm{MS}}} (2\,\mathrm{GeV})=1.369$, this translates into

89 $$\begin{aligned} N_\mathrm{f}=2+1: \quad B_{{K}}^{\overline{\mathrm{MS}}}(2\,\mathrm{GeV}) = 0.5596(72). \end{aligned}$$

Thus, the accuracy of the current global estimate stands at an impressive 1.3 %, which represents a significant improvement over the 2.7 % uncertainty quoted in the previous edition of the FLAG review ($\hat{B}_{K}=0.738(20)$). The two results are, however, completely consistent.

Passing over to describing the results computed for $N_\mathrm{f}=2$ flavours, we note that the situation is unchanged since the publication of the previous edition of the FLAG review [1]. In particular, the result of ETM 10A [314] is the only one which allows for an extensive investigation of systematic uncertainties. In fact, it is the only published $N_\mathrm{f}=2$ calculation involving data computed at three values of the lattice spacing. Being the only result without red tags, it can therefore be identified with the currently best global estimate for two-flavour QCD, i.e.

90 $$\begin{aligned} N_\mathrm{f}&= 2: \quad \hat{B}_{{K}} = 0.729(25)(17),\nonumber \\&B_{{K}}^{\overline{\mathrm{MS}}}(2\,\mathrm{GeV}) = 0.533(18)(12). \end{aligned}$$

The result in the ${\overline{\mathrm{MS}}}$ scheme has been obtained by applying the same conversion factor of 1.369 as in the three-flavour theory.

The grey bands in Fig. 12 represent the global estimates for $N_\mathrm{f}=2$ and $N_\mathrm{f}=2+1$. It appears that $B_{{K}}$ may be slightly smaller in two-flavour QCD, but in view of the relatively large uncertainty of the $N_\mathrm{f}=2$ result, the difference is hardly significant.

## $D$-meson decay constants and form factors

Leptonic and semileptonic decays of charmed $D$- and $D_{s}$-mesons occur via charged $W$-boson exchange, and they are sensitive probes of $c \rightarrow d$ and $c \rightarrow s$ quark flavour-changing transitions. Given experimental measurements of the branching fractions combined with sufficiently precise theoretical calculations of the hadronic matrix elements, they enable the determination of the CKM matrix elements $|V_{cd}|$ and $|V_{cs}|$ (within the Standard Model) and a precise test of the unitarity of the second row of the CKM matrix. Here we summarise the status of lattice-QCD calculations of the charmed leptonic decay constants and semileptonic form factors. Significant progress has been made in computing $f_{D_{(s)}}$ and the $D\rightarrow \pi (K) \ell \nu$ form factors in the last few years, largely due to the introduction of highly improved lattice-fermion actions that enable the simulation of $c$-quarks with the same action as for the $u$, $d$ and $s$-quarks.

The charm-quark methods discussed in this review have been validated in a number of ways. Because several groups use the same action for charm and bottom quarks, tests of charm-quark methods are also relevant for the $B$-physics results discussed in Sect. 8, and they are therefore summarised in the introduction of that section. Finally, we note that we limit our review to results based on modern simulations with reasonably light pion masses (below approximately 500 MeV). This excludes results obtained from the earliest unquenched simulations, which typically had two flavours in the sea, and which were limited to heavier pion masses because of the constraints imposed by the computational resources and methods available at that time.

Following our review of lattice-QCD calculations of $D_{(s)}$-meson leptonic decay constants and semileptonic form factors, we then interpret our results within the context of the Standard Model. We combine our best-determined values of the hadronic matrix elements with the most recent experimentally measured branching fractions to obtain $|V_{cd(s)}|$ and test the unitarity of the second row of the CKM matrix.

### Leptonic decay constants $f_D$ and $f_{D_{s}}$

In the Standard Model the decay constant $f_{D_{(s)}}$ of a pseudoscalar $D$- or $D_{s}$-meson is related to the branching ratio for leptonic decays mediated by a $W$ boson through the formula

91 $$\begin{aligned} {\mathcal {B}}(D_{(s)} \rightarrow \ell \nu _\ell )&= {{G_F^2|V_{cq}|^2 \tau _{D_{(s)}}}\over {8 \pi }} f_{D_{(s)}}^2 m_\ell ^2 m_{D_{(s)}}\nonumber \\&\times \left( 1-{{m_\ell ^2}\over {m_{D_{(s)}}^2}}\right) ^2, \end{aligned}$$

where $V_{cd}$ ($V_{cs}$) is the appropriate CKM matrix element for a $D$ ($D_{s}$) meson. The branching fractions have been experimentally measured by CLEO, Belle and Babar with a precision around 5–6 % for the $D_{s}$-meson; the uncertainties are twice as large for the Cabibbo suppressed $D$-meson decay modes [74]. When combined with lattice results for the decay constants, they allow for determinations of $|V_{cs}|$ and $|V_{cd}|$.

**Fig. 13:**
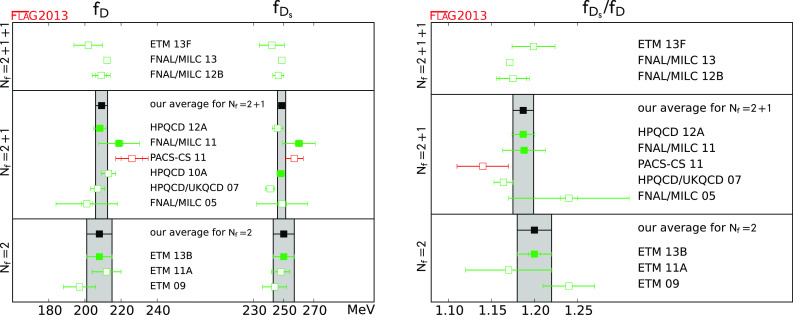
Decay constants of the $D$- and $D_{s}$-mesons [values in Table 20 and Eqs. (93), (94)]. The significance of the colours is explained in Sect. 2. The *black squares* and *grey bands* indicate our averages. Errors in FNAL/MILC 13 are smaller than the symbols

In lattice-QCD calculations the decay constants $f_{D_{(s)}}$ are extracted from Euclidean matrix elements of the axial current

92 $$\begin{aligned} {\langle }0| A^{\mu }_{cq} | D_{q}(p) {\rangle }= f_{D_{q}}\;p_{D_{q}}^\mu , \end{aligned}$$

with $q=d,s$ and $A^{\mu }_{cq} =\bar{c}\gamma _\mu \gamma _5 q$. Results for $N_\mathrm{f}=2,\; 2+1$ and $2+1+1$ dynamical flavours are summarised in Table 20 and Fig. 13.

**Table 20:** Decay constants of the $D$- and $D_{s}$-mesons (in MeV) and their ratio

Collaboration	Ref.	$N_\mathrm{f}$	Publication status	Continuum extrapolation	Chiral extrapolation	Finite volume	Renormalisation/ matching	Heavy quark treatment	$f_D$	$f_{D_{s}}$	$f_{D_{s}}/f_D$
ETM 13F	[155]	$2+1+1$	C						202 (8)	242 (8)	1.199 (25)
FNAL/MILC 13$^\nabla$	[329]	$2+1+1$	C						212.3 (0.3) (1.0)	248.7 (0.2) (1.0)	1.1714 (10) (25)
FNAL/MILC 12B	[330]	$2+1+1$	C						209.2 (3.0) (3.6)	246.4 (0.5) (3.6)	1.175 (16) (11)
HPQCD 12A	[331]	$2+1$	A						208.3 (1.0) (3.3)	246.0 (0.7) (3.5)	1.187 (4) (12)
FNAL/MILC 11	[332]	$2+1$	A						218.9 (11.3)	260.1 (10.8)	1.188 (25)
PACS-CS 11	[333]	$2+1$	A						226 (6) (1) (5)	257 (2) (1) (5)	1.14 (3)
HPQCD 10A	[94]	$2+1$	A						213 (4)$^{*}$	248.0 (2.5)	
HPQCD/UKQCD 07	[165]	$2+1$	A						207 (4)	241 (3)	1.164 (11)
FNAL/MILC 05	[334]	$2+1$	A						201 (3) (17)	249 (3) (16)	1.24 (1) (7)
ETM 13B$^\square$	[335]	2	P						208 (7)	250 (7)	1.20 (2)
ETM 11A	[336]	2	A						212 (8)	248 (6)	1.17 (5)
ETM 09	[169]	2	A						197 (9)	244 (8)	1.24 (3)

$^{\nabla }$ Update of FNAL/MILC 12B

$^{*}$ This result is obtained by using the central value for $f_{D_{s}}/f_D$ from HPQCD/UKQCD 07 and increasing the error to account for the effects from the change in the physical value of $r_1$

$^{\square }$ Update of ETM 11A and ETM 09

The ETM collaboration has published results for $D$- and $D_{s}$-meson decay constants with two dynamical flavours, using the twisted-mass fermionic action at maximal twist with the tree-level improved Symanzik gauge action. In this setup the decay constants can be extracted from an absolutely normalised current and they are automatically ${\mathcal {O}}(a)$ improved. In ETM 09 three lattice spacings between $0.1$ and $0.07$ fm are considered with pion masses down to 270 MeV. Heavy meson $\chi$PT formulae plus terms linear in $a^2$ have been used for the continuum/chiral extrapolations, which have been performed in two different ways in order to estimate systematic effects. In the first approach $f_{D_{s}}\sqrt{m_{D_{s}}}$ and $\frac{f_{D_{s}}\sqrt{m_{D_{s}}}}{ f_{D}\sqrt{m_{D}}}$ are fitted, whereas in the second case the ratios $\frac{f_{D_{s}}\sqrt{m_{D_{s}}}}{f_K}$ and $\frac{f_{D_{s}}\sqrt{m_{D_{s}}}}{f_K} \times \frac{f_\pi }{f_{D}\sqrt{m_{D}}}$ are analysed. As expected, the pion-mass dependence of $f_{D_{s}}\sqrt{m_{D_{s}}}$ turns out to be very mild. In addition the double ratio $\frac{f_{D_{s}}\sqrt{m_{D_{s}}}}{f_K} \times \frac{f_\pi }{f_{D}\sqrt{m_{D}}}$ shows little dependence on the pion mass as well as on the lattice spacing. Cutoff effects on the contrary are rather large on the decay constants, with the difference between the physical-mass result at the finest lattice spacing and in the continuum being approximately 5 %. ETM 11A contains an update of the results in ETM 09 obtained by enlarging the statistics on some of the ensembles and by including a finer lattice resolution with $a\approx 0.054$ fm, which implies a reduction of cutoff effects by a factor two. Moreover, in ETM 11A the continuum extrapolations are performed after interpolating the results at different lattice spacings to fixed values of the heavy-quark mass. In the case of the SU(3)-breaking ratio $f_{D_{s}}/f_D$, the uncertainty associated with the chiral extrapolation is estimated by comparing fits either following heavy meson $\chi$PT or assuming a simple linear dependence on the light-quark mass. These results have been further updated in ETM 13B [335] by using optimised smearing interpolating fields in order to suppress excited states contributions and by changing the chiral extrapolation. The ensembles used are the same as in ETM 11A. Values at the physical point are obtained by first extrapolating $f_{D_{s}} \sqrt{m_{D_{s}}}$ linearly in $m_{l}^2$ and in $a^2$ and then by extrapolating the double ratio $(f_{D_{s}}/f_D)/(f_K/f_\pi )$ using HM$\chi$PT. The value of $f_K/f_\pi$ is taken from the $N_\mathrm{f}=2+1$ average in [1], in order to avoid correlations with estimates obtained by the ETM collaboration.

As results from just one collaboration exist in the literature, the $N_\mathrm{f}=2$ averages are simply given by the values in ETM 13B, which read

93 $$\begin{aligned}&N_\mathrm{f}\!=\!2: \quad f_D\!=\!(208\pm 7) \;\mathrm{MeV}, \quad \! f_{D_{s}} \!=\! (250\pm 7)\;\mathrm{MeV}, \quad \!\nonumber \\&\quad {{f_{D_{s}}}\over {f_D}}=1.20\pm 0.02. \end{aligned}$$

The ALPHA Collaboration presented preliminary results on $f_{D_{(s)}}$ with two dynamical flavours at the Lattice 2013 Conference [337]. The proceedings, however, appeared after the deadline for consideration in this review and therefore are not discussed here.

Several collaborations have produced results with $N_\mathrm{f}=2+1$ dynamical flavours. The most precise determinations come from a sequence of publications by HPQCD/UKQCD [94, 165, 331]. In all cases configurations generated by MILC with Asqtad rooted staggered quarks in the sea and a one-loop tadpole-improved Symanzik gauge action have been analysed (see [15] and references therein). The main differences are in the ensembles utilised and in the absolute scale setting. The relative scale is always set through $r_1$ derived from the static quark–antiquark potential.

In HPQCD/UKQCD 07 [165] three lattice spacings, $a\approx 0.15,\; 0.12$ and $0.09$ fm, with RMS pion masses between 542 and 329 MeV, have been considered. This gives rather large values for the charm-quark mass in lattice units, $0.43 <am_{c}< 0.85$, and indeed lattice artefacts are estimated to be the second largest systematic uncertainty in the computation. The main systematic error is resulting from the absolute scale setting, which had previously been performed through the $\Upsilon$ spectrum, using NRQCD for the $b$ quark. The estimate reads $r_1=0.321(5)$ fm.

In 2010, HPQCD obtained a more precise determination of $r_1=0.3133(23)$, based on several different physical inputs (including $f_\pi$, $f_K$ and the $\Upsilon$ spectrum) and improved continuum limit extrapolations. It is worth noting that the new $r_1$ is about 1.5$\sigma$ lower than the older value. The publications HPQCD 10A [94] and HPCQD 12A [331] update the computations of $f_{D_{s}}$ and $f_D$, respectively, using the new scale determination. These results enter our final averages. The change in the scale requires a retuning of the bare quark masses and a change in the conversion of dimensionless quantities, measured in units of $r_1$, to physical ones, measured in MeV.

In HPQCD 10A, $f_{D_{s}}$ is calculated on ensembles with $a\approx 0.06$ and $0,045$ fm and with RMS pion masses ranging between 542 and 258 MeV. The chiral and continuum extrapolations have been performed simultaneously by employing polynomials quadratic in the sea-quark mass $\delta _{q} = \frac{m_{q,\mathrm{sea}} -m_{q,\mathrm{phys}}}{m_{q,\mathrm{phys}}}$, with $q = s, l$, and through the eighth power of the charm-quark mass, including cross terms of the form $\delta _{q} (am_{c})^n$. The valence strange- and charm-quark masses are fixed to their physical values obtained from matching to the $\eta _{s}$ and $\eta _{c}$ masses. The fits are robust against variations, such as the exclusion of ensembles with the coarsest and finest lattice spacings, or a change in the functional form such that terms up to $(am_{c})^4$ only are kept. The largest source of uncertainty in HPQCD 10A still comes from the value of $r_1$ and it amounts to 0.6 %. The published error includes a 0.1 % contribution coming from an estimate of electromagnetic effects obtained using a potential model.

The process of switching to the improved determination of $r_1$ is finally completed in HPQCD 12A [331], where new values of $f_D$ and the ratio $f_{D_{s}}/f_D$ are reported. The statistics is enlarged at the $a \approx 0.12$ fm and $a \approx 0.09$ fm lattices and for the latter a more chiral point, with light-quark masses halved with respect to HPQCD/UKQCD 07, is added. The three-point function for $D \rightarrow \pi$ at zero recoil momentum (calculated for a different project) is used to perform simultaneous fits to two- and three-point functions. This turns out to be beneficial in reducing the statistical errors on the hadron masses and decay-constant matrix elements. Chiral and continuum extrapolations are carried out at the same time adopting partially quenched heavy meson $\chi$PT augmented by $(am_{c})^2$ and $(am_{c})^4$ terms. Given the rather large values of $am_{c}$ between 0.4 and 0.6, the continuum extrapolation gives the largest systematical uncertainty, amounting to roughly 1 % out of the total 1.7 and 1.1 % total errors on $f_D$ and on $f_{D_{s}}/f_D$, respectively. Finally, the HPQCD collaboration also calculates the ratio $f_+^{D \rightarrow \pi }(0)/f_D$ using the result for the semileptonic form factor from [338] and find good agreement with the experimental ratio which is independent of $|V_{cd}|$. Summarizing the computations by HPQCD: concerning $f_D$, HPQCD 12A supersedes HPQCD/UKQCD 07 and HPQCD 10A because of the more chiral points considered but does not supersede HPQCD 10A for $f_{D_{s}}$ as finer resolutions are included in the latter, which contains the collaboration’s most precise result for the $D_{s}$-meson decay constant.

The PACS-CS Collaboration published in 2011 a computation of the $D$ and $D_{s}$ decay constants with $2+1$ flavours of non-perturbatively ${\mathcal {O}}(a)$ improved Wilson fermions and the Iwasaki gauge action [333]. For the charm quark the Tsukuba heavy quark action is used. The parameters in the action and the renormalisation constants of the charm-light and charm-strange axial currents are computed in a mixed setup, partly non-perturbatively (typically the massless contribution) and partly relying on one-loop perturbation theory; see Appendix A for details. This leaves residual cutoff and matching effects of ${\mathcal {O}}(\alpha ^2_{s} a\Lambda _\mathrm{QCD},\, (a\Lambda )^2,\, \alpha _\mathrm{s}^2)$ in the computation, which, in addition is carried out at one value of the lattice spacing only ($a\approx 0.09$ fm). Quark masses are quite low, yielding $m_\pi =152(6)$ MeV and the ensemble is reweighted to the physical point using the technique in [20]. However, measurements are performed on only one set of configurations with $L/a=32$, such that $m_\pi L$ is around 2.2. For this reason, and for the limitation to a single lattice spacing, the PACS-CS 11 results do not enter our averages.

The Fermilab Lattice and MILC collaborations have presented several computations of $D_{(s)}$-meson decay constants with $2+1$ flavours of dynamical quarks [332, 334]. Their first published results are in Ref. [334] (FNAL/MILC 05), which were later updated and superseded in Ref. [332] (FNAL/MILC 11). The MILC Asqtad ensembles, as for the HPQCD results, have been used in both cases. For the charm quark the Fermilab action is adopted, with mostly non-perturbative (mNPR) renormalisation of the axial currents (see Appendix A for details). In FNAL/MILC 05 three lattice spacings with $a\approx 0.18,\; 0.12$ and $0.09$ fm, according to the original estimate $r_1=0.321(5)$ fm, have been considered. RMS pion masses are slightly larger than 400 MeV. Chiral and continuum extrapolations are performed at the same time by using the $\chi$PT expressions at NLO for staggered quarks. Discretisation effects and the chiral fits are the largest sources of systematic errors in $f_D$ and in $f_{D_{s}}$, each effect being responsible for a systematic between 4 and 6 %. Cutoff effects are significantly smaller in the ratio $f_{D_{s}}/f_D$, whose systematic uncertainty (around 5 %) is dominated by the chiral extrapolation.

These uncertainties are reduced in FNAL/MILC 11. The same setup concerning lattice actions and renormalisation is used as in FNAL/MILC 05 but lighter pion masses (down to 320 MeV for the RMS values) are included in the analysis and the extremely coarse $0.18$ fm ensembles are replaced by finer $0.15$ fm ones. The scale is set through $r_1=0.3120(22)$ fm, as obtained from an average of previous MILC and HPQCD determinations. One-loop rooted staggered partially quenched $\chi$PT plus leading order in the heavy-quark expansion formulae are used for the chiral and continuum extrapolations. The expressions parameterise also the effects of hyperfine and flavour splittings. Discretisation effects are estimated using a combination of heavy-quark and Symanzik effective theories to be around 3 % for $f_{D_{(s)}}$ and negligible for the ratio. At this level of accuracy the truncation errors in the small correction factor inherent in the mNPR method are not negligible anymore; the authors conservatively estimate the two-loop and higher-order perturbative truncation errors to the full size of the known one-loop term, i.e. roughly 1 % for the decay constants.

As shown in Table 20 the $N_\mathrm{f}=2+1$ computations which fulfill our quality criteria and can enter the averages are HPCQD 12A and FNAL/MILC 11 for $f_D$ and the SU(3) breaking ratio $f_{D_{s}}/f_D$, and HPQCD 10A and FNAL/MILC 11 for $f_{D_{s}}$. Because FNAL/MILC and HPQCD use a largely overlapping set of configurations, we treat the statistical errors as 100 % correlated and finally quote

94 $$\begin{aligned}&N_\mathrm{f}=2+1: \quad \!\!\! f_D=(209.2\pm 3.3) \;\mathrm{MeV}, \quad \!\!\!\nonumber \\&f_{D_{s}}=(248.6\pm 2.7) \;\mathrm{MeV}, \quad \!\!\! {{f_{D_{s}}}\over {f_D}}=1.187\pm 0.012. \end{aligned}$$

The first computation of $f_D$ and $f_{D_{s}}$ with $N_\mathrm{f}=2+1+1$ sea quarks is presented in Ref. [330] (FNAL/MILC 12B), published as a proceeding contribution to the Lattice 2012 Conference. The calculation is performed on configurations generated by the MILC Collaboration using HISQ sea quarks and a one-loop tadpole-improved Symanzik gauge action [250]. Light, strange and charm valence quarks are also in the HISQ regularisation. Four lattice resolutions in the range $a\approx 0.15$–0.06 fm are considered. RMS pion masses vary between 306 and 144 MeV and include ensembles at each lattice spacing with Goldstone pions at the physical point. The dominant systematic uncertainties are due to the scale setting (through $f_\pi$) and the continuum extrapolation, and they are both estimated to be at the percent level. The results have been updated in FNAL/MILC 13 [329]. New measurements at the finest lattice spacing have been included in the analysis and the statistics have been significantly increased in each ensemble. In addition, heavy-meson, rooted, all-staggered chiral perturbation theory (HMrAS$\chi$PT), as introduced in Ref. [339] to treat both the light and charm quarks as staggered, has been used at NLO in performing chiral and continuum extrapolations. The configurations used in these computations have been generated using both the RHMC and the RHMD algorithms. The latter is an inexact algorithm, where the accept/reject step at the end of the molecular-dynamics trajectory is skipped. In Ref. [250] results for the plaquette, the bare fermion condensates and a few meson masses, using both algorithms, are compared and found to agree within statistical uncertainties.

The ETM collaboration has also reported results with $2+1+1$ dynamical flavours at the Lattice 2013 Conference [155]. The configurations have been generated using the Iwasaki action in the gauge and the Wilson twisted-mass action for sea quarks. The charm and strange valence quarks are discretised as Osterwalder–Seiler fermions [340]. Three different lattice spacings in the range 0.09–0.06 fm have been analysed with pion masses as low as 210 MeV in lattices of linear spatial extent of about 2.5–3 fm. As in the $N_\mathrm{f}=2$ computation in ETM 13B, the chiral and continuum extrapolations are performed first for $f_{D_{s}}$, including terms linear and quadratic in $m_{l}$ and one term linear in $a^2$ in the parameterisation, and then for the double ratio $(f_{D_{s}}/f_D)/(f_K/f_\pi )$ using continuum HM$\chi$PT. The main systematic uncertainties are due to the continuum and chiral extrapolation for $f_{D_{s}}$ and to the error on $f_K/f_\pi$, which is also provided in these proceedings and discussed in Sect. 4 of this review, for $f_D$.

As a final remark, since the accuracy of the lattice determinations of the $D$-meson decay constant is rapidly improving, it will become important in the future, especially when comparing to experimental numbers, to distinguish between $f_{D^+}$ and the average of $f_{D^+}$ and $f_{D^0}$. The current status is summarised as follows: FNAL/MILC results concern $f_{D^+}$, whereas HPQCD, PACS-CS and ETMC numbers correspond to the average of the decay constants for $D^+$ and $D^0$.

### Semileptonic form factors for $D\rightarrow \pi \ell \nu$ and $D\rightarrow K \ell \nu$

The form factors for semileptonic $D\rightarrow \pi \ell \nu$ and $D\rightarrow K \ell \nu$ decay, when combined with experimental measurements of the decay widths, enable determinations of the CKM matrix elements $|V_{cd}|$ and $|V_{cs}|$ via

95 $$\begin{aligned}&\frac{\hbox {d}\Gamma (D\rightarrow P\ell \nu )}{\hbox {d}q^2} = \frac{G_F^2 |V_{cx}|^2}{24 \pi ^3} \,\frac{(q^2-m_\ell ^2)^2\sqrt{E_P^2-m_P^2}}{q^4m_{D}^2} \,\nonumber \\&\quad \times \left[ \left( 1+\frac{m_\ell ^2}{2q^2}\right) m_{D}^2(E_P^2-m_P^2)|f_+(q^2)|^2\right. \nonumber \\&\quad \left. + \frac{3m_\ell ^2}{8q^2}(m_{D}^2-m_P^2)^2|f_0(q^2)|^2 \right] , \end{aligned}$$

where $x = d, s$ is the daughter light quark, $P= \pi , K$ is the daughter light pseudoscalar meson, and $q = (p_D - p_P)$ is the momentum of the outgoing lepton pair. The vector and scalar form factors $f_+(q^2)$ and $f_0(q^2)$ parameterise the hadronic matrix element of the heavy-to-light quark flavour-changing vector current $V_\mu = i \overline{x} \gamma _\mu c$:

96 $$\begin{aligned} {\langle }P| V_\mu | D {\rangle }&= f_+(q^2) \left( {p_D}_\mu + {p_P}_\mu - \frac{m_D^2 - m_P^2}{q^2}\,q_\mu \right) \nonumber \\&+ f_0(q^2) \frac{m_D^2 - m_P^2}{q^2}\,q_\mu , \end{aligned}$$

and satisfy the kinematic constraint $f_+(0) = f_0(0)$ at zero momentum-transfer. Because the contribution to the decay width from the scalar form factor is proportional to $m_\ell ^2$, it can be neglected for $\ell = e, \mu$, and Eq. (95) simplifies to

97 $$\begin{aligned} \frac{\hbox {d}\Gamma \!\left( D \rightarrow P \ell \nu \right) }{\hbox {d} q^2} = \frac{G_F^2}{24 \pi ^3} |\vec {p}_{P}|^3 {|V_{cx}|^2 |f_+^{DP} (q^2)|^2}. \end{aligned}$$

In practice, most lattice-QCD calculations of $D\rightarrow \pi \ell \nu$ and $D\rightarrow K \ell \nu$ focus on providing the value of the vector form factor at a single value of the momentum transfer, $f_+(q^2=0)$, which is sufficient to obtain $|V_{cd}|$ and $|V_{cs}|$. Because the decay rate cannot be measured directly at zero momentum transfer, comparison of these lattice-QCD results with experiment requires a slight extrapolation of the experimental measurement. Some lattice-QCD calculations also provide determinations of the $D\rightarrow \pi \ell \nu$ and $D\rightarrow K \ell \nu$ form factors over the full kinematic range $0 < q^2 < q^2_\mathrm{max} = (m_D - m_P)^2$, thereby allowing a comparison of the shapes of the lattice simulation and experimental data. This non-trivial test in the $D$ system provides a strong check of lattice-QCD methods that are also used in the $B$-meson system.

Lattice-QCD calculations of the $D\rightarrow \pi \ell \nu$ and $D\rightarrow K \ell \nu$ form factors typically use the same light-quark and charm-quark actions as those of the leptonic decay constants $f_D$ and $f_{D_{s}}$. Therefore many of the same issues arise, e.g. chiral extrapolation of the light-quark mass(es) to the physical point and discretisation errors from the charm quark, and matching the lattice weak operator to the continuum, as discussed in the previous section. Two strategies have been adopted to eliminate the need to renormalise the heavy–light vector current in recent calculations of $D\rightarrow \pi \ell \nu$ and $D\rightarrow K \ell \nu$, both of which can be applied to simulations in which the same relativistic action is used for the light $(u,d,s)$ and charm quarks. The first method was proposed by Bećirević and Haas in Ref. [341], and introduces double-ratios of lattice three-point correlation functions in which the vector current renormalisation cancels. Discretisation errors in the double ratio are of ${\mathcal O}((am_{h})^2)$ provided that the vector-current matrix elements are ${\mathcal O}(a)$ improved. The vector and scalar form factors $f_+(q^2)$ and $f_0(q^2)$ are obtained by taking suitable linear combinations of these double ratios. The second method was introduced by the HPQCD Collaboration in Ref. [342]. In this case, the quantity $(m_{c} - m_{x} ) {\langle }P | S | D \rangle$, where $m_{x}$ and $m_{c}$ are the bare lattice quark masses and $S = \bar{x}c$ is the lattice scalar current, does not get renormalised. The desired form factor at zero momentum transfer can be obtained by (i) using a Ward identity to relate the matrix element of the vector current to that of the scalar current, and (ii) taking advantage of the kinematic identity at zero momentum transfer $f_+(0) = f_0(0)$, such that $f_+(q^2=0) = (m_{c} - m_{x} ) {\langle }P | S | D {\rangle }/ (m^2_D - m^2_P)$.

Additional complications enter for semileptonic decay matrix elements due to the non-zero momentum of the outgoing pion or kaon. Both statistical errors and discretisation errors increase at larger momenta, so results for the lattice form factors are most precise at $q^2_\mathrm{max}$. However, because lattice calculations are performed in a finite spatial volume, the pion or kaon three-momentum can only take discrete values in units of $2\pi /L$ when periodic boundary conditions are used. For typical box sizes in recent lattice $D$- and $B$-meson form-factor calculations, $L \sim 2.5$–3 fm; thus the smallest non-zero momentum in most of these analyses ranges from $p_P \equiv |\vec {p}_P| \sim 400$–$500$ MeV. The largest momentum in lattice heavy–light form-factor calculations is typically restricted to $p_P \le 4\pi /L$ For $D \rightarrow \pi \ell \nu$ and $D \rightarrow K \ell \nu$, $q^2=0$ corresponds to $p_\pi \sim 940$ MeV and $p_K \sim 1$ GeV, respectively, and the full recoil-momentum region is within the range of accessible lattice momenta.^30^ Therefore the interpolation to $q^2=0$ is relatively insensitive to the fit function used to parameterise the momentum dependence, and the associated systematic uncertainty in $f_+(0)$ is small. In contrast, determinations of the form-factor shape can depend strongly on the parameterisation of the momentum dependence, and the systematic uncertainty due to the choice of model function is often difficult to quantify. This is becoming relevant for $D \rightarrow \pi \ell \nu$ and $D \rightarrow K \ell \nu$ decays as collaborations are beginning to present results for $f_+(q^2)$ and $f_0(q^2)$ over the full kinematic range. The parameterisation of the form-factor shape is even more important for semileptonic $B$ decays, for which the momentum range needed to connect to experiment is often far from $q^2_\mathrm{max}$.

A class of functions based on general field-theory properties, known as $z$-expansions, has been introduced to allow model-independent parameterisations of the $q^2$ dependence of semileptonic form factors over the entire kinematic range (see, e.g., Refs. [349, 350]). The use of such functions is now standard for the analysis of $B \rightarrow \pi \ell \nu$ transitions and the determination of $|V_{ub}|$ [126, 351–353]; we therefore discuss approaches for parameterising the $q^2$ dependence of semileptonic form factors, including $z$-expansions, in Sect. 8.3. Here we briefly summarise the aspects most relevant to calculations of $D \rightarrow \pi \ell \nu$ and $D \rightarrow K \ell \nu$. In general, all semileptonic form factors can be expressed as a series expansion in powers of $z^n$ times an overall multiplicative function that accounts for any sub-threshold poles and branch cuts, where the new variable $z$ is a non-linear function of $q^2$. The series coefficients $a_n$ depend upon the physical process (as well as the choice of the prefactors), and can only be determined empirically by fits to lattice or experimental data. Unitarity establishes strict upper bounds on the size of the $a_n$’s, while guidance from heavy-quark power counting provides even tighter constraints. Recently the HPQCD Collaboration introduced a variation on this approach, which they refer to as a “modified $z$-expansion,” that they use to simultaneously extrapolate their lattice simulation data to the physical light-quark masses and the continuum limit, and to interpolate/extrapolate their lattice data in $q^2$. They do so by allowing the coefficients $a_n$ to depend on the light-quark masses, squared lattice spacing, and, in some cases the charm-quark mass and pion or kaon energy. Because the modified $z$-expansion is not derived from an underlying effective field theory, there are several potential concerns with this approach that have yet to be studied in the literature. The most significant is that there is no theoretical derivation relating the coefficients of the modified $z$-expansion to those of the physical coefficients measured in experiment; it therefore introduces an unquantified model dependence in the form-factor shape. Further, if Bayesian methods are used to constrain the parameters of the modified $z$-expansion, there is no a priori way to obtain priors for their natural size. The “modified” $z$-expansion is now being utilised by collaborations other than HPQCD and for quantities other than $D \rightarrow \pi \ell \nu$ and $D \rightarrow K \ell \nu$ [354, 355]. We advise treating results that utilise the “modified” $z$-expansion to obtain form-factor shapes and CKM matrix elements with caution, however, since the systematics of this approach warrant further study.

#### Results for $f_+(0)$

We now review the status of lattice calculations of the $D \rightarrow \pi \ell \nu$ and $D \rightarrow K \ell \nu$ form factors at $q^2=0$. As in the first version of this review, although we also describe ongoing calculations of the form-factor shapes, we do not rate these calculations.

The most advanced $N_\mathrm{f} = 2$ lattice-QCD calculation of the $D \rightarrow \pi \ell \nu$ and $D \rightarrow K \ell \nu$ form factors is by the ETM Collaboration [345]. This still preliminary work uses the twisted-mass Wilson action for both the light and charm quarks, with three lattice spacings down to $a \approx 0.068$ fm and (charged) pion masses down to $m_\pi \approx 270$ MeV. The calculation employs the ratio method of Ref. [341] to avoid the need to renormalise the vector current, and extrapolates to the physical light-quark masses using SU(2) heavy–light meson $\chi$PT formulated for twisted-mass fermions. ETM simulate with non-periodic boundary conditions for the valence quarks to access arbitrary momentum values over the full physical $q^2$ range, and interpolate to $q^2=0$ using the Bećirević–Kaidalov ansatz [356]. The statistical errors in $f_+^{D\pi }(0)$ and $f_+^{DK}(0)$ are 9 and 7 %, respectively, and lead to rather large systematic uncertainties in the fits to the light-quark mass and energy dependence (7 and 5 %, respectively). Another significant source of uncertainty is from discretisation errors (5 and 3 %, respectively). On the finest lattice spacing used in this analysis $am_{c} \sim 0.17$, so ${\mathcal O}((am_{c})^2)$ cutoff errors are expected to be about 5 %. This can be reduced by including the existing $N_\mathrm{f} = 2$ twisted-mass ensembles with $a \approx 0.051$ fm discussed in Ref. [241]. Work is in progress by the ETM Collaboration to compute $f_+^{D\pi }(0)$ and $f_+^{DK}(0)$ using the same methods on the $N_\mathrm{f} = 2+1+1$ twisted-mass Wilson lattices [98]. This calculation will include dynamical charm-quark effects and use three lattice spacings down to $a\approx 0.06$ fm.

The first published $N_\mathrm{f} = 2+1$ lattice-QCD calculation of the $D \rightarrow \pi \ell \nu$ and $D \rightarrow K \ell \nu$ form factors is by the Fermilab Lattice, MILC, and HPQCD Collaborations [357]. (Because only two of the authors of this work are in HPQCD, and to distinguish it from other more recent works on the same topic by HPQCD, we hereafter refer to this work as “FNAL/MILC.”) This work uses Asqtad-improved staggered sea quarks and light ($u,d,s$) valence quarks and the Fermilab action for the charm quarks, with a single lattice spacing of $a \approx 0.12$ fm. At this lattice spacing, the staggered taste splittings are still fairly large, and the minimum RMS pion mass is $\approx 510$ MeV. This calculation renormalises the vector current using a mostly non-perturbative approach, such that the perturbative truncation error is expected to be negligible compared to other systematics. The Fermilab Lattice and MILC Collaborations present results for the $D \rightarrow \pi \ell \nu$ and $D \rightarrow K \ell \nu$ semileptonic form factors over the full kinematic range, rather than just at zero momentum transfer. In fact, the publication of this result predated the precise measurements of the $D\rightarrow K \ell \nu$ decay width by the FOCUS [358] and Belle experiments [359], and predicted the shape of $f_+^{DK}(q^2)$ quite accurately. This bolsters confidence in calculations of the $B$-meson semileptonic decay form factors using the same methodology. Work is in progress [360] to reduce both the statistical and systematic errors in $f_+^{D\pi }(q^2)$ and $f_+^{DK}(q^2)$ through increasing the number of configurations analysed, simulating with lighter pions, and adding lattice spacings as fine as $a \approx 0.045$ fm. In parallel, the Fermilab Lattice and MILC collaborations are initiating a new calculation of $D \rightarrow \pi \ell \nu$ and $D \rightarrow K \ell \nu$ using the HISQ action for all valence and sea quarks [361]; this calculation will focus on obtaining the form factors at zero momentum transfer using the scalar form-factor method [342] to avoid the need for current renormalisation and (partially) twisted boundary conditions [344, 362] to simulate directly at $q^2=0$.

The most precise published calculations of the $D \rightarrow \pi \ell \nu$ [338] and $D \rightarrow K \ell \nu$ [342] form factors are by the HPQCD Collaboration. These analyses also use the $N_\mathrm{f} = 2+1$ Asqtad-improved staggered MILC configurations at two lattice spacings $a \approx 0.09$ and 0.12 fm, but they use the HISQ action for the valence $u,d,s$ and $c$ quarks. In these mixed-action calculations, the HISQ valence light-quark masses are tuned so that the ratio $m_{l}/m_{s}$ is approximately the same as for the sea quarks; the minimum RMS sea-pion mass is $\approx 390$ MeV. They calculate the form factors at zero momentum transfer by relating them to the matrix element of the scalar current, which is not renormalised. They use the “modified $z$-expansion” to simultaneously extrapolate to the physical light-quark masses and continuum and interpolate to $q^2 = 0$, and they allow the coefficients of the series expansion to vary with the light- and charm-quark masses. The form of the light-quark dependence is inspired by $\chi$PT, and includes logarithms of the form $m_\pi ^2 \mathrm{log} (m_\pi ^2)$ as well as polynomials in the valence-, sea-, and charm-quark masses. Polynomials in $E_{\pi (K)}$ are also included to parameterise momentum-dependent discretisation errors. The coefficients of each term are constrained using Gaussian priors with widths inspired by $\chi$PT power counting for the light-quark mass terms and by HISQ power-counting for the others. The number of terms is increased until the result for $f_+(0)$ stabilises, such that the quoted fit error for $f_+(0)$ includes both statistical uncertainties and those due to most systematics. The largest uncertainties in these calculations are from statistics and charm-quark discretisation errors.

The HPQCD Collaboration is now extending their work on $D$-meson semileptonic form factors to determining their shape over the full kinematic range [346], and recently obtained results for the $D \rightarrow K \ell \nu$ form factors $f+(q^2)$ and $f_0(q^2)$ [347]. This analysis uses a subset of the ensembles included in their earlier work, with two sea-quark masses at $a \approx 0.12$ fm and one sea-quark mass at $a \approx 0.09$ fm, but with approximately three times more statistics on the coarser ensembles and ten times more statistics on the finer ensemble. As above, the scalar current is not renormalised. The spatial vector-current renormalisation factor is obtained by requiring that $f_+(0)^{H\rightarrow H} = 1$ for $H = D, D_{s}, \eta _{s}$ and $\eta _{c}$. The renormalisation factors for the flavour-diagonal currents agree for different momenta as well as for charm-charm and strange-strange external mesons within a few percent, and they are then used to renormalise the flavour-changing charm-strange and charm-light currents. The charm-strange temporal vector current is normalised by matching to the scalar current $f_0(q^2_\mathrm{max})$. Also as above, they simultaneously extrapolate to the physical light-quark masses and continuum and interpolate/extrapolate in $q^2$ using the modified $z$-expansion. In this case, however, they only allow for light-quark mass and lattice-spacing dependence in the series coefficients, but not for charm-quark mass or kaon energy dependence, and constrain the parameters with Bayesian priors. It is not clear, however, that only three sea-quark ensembles at two lattice spacings are sufficient to resolve the quark-mass and lattice spacing dependence, even within the context of constrained fitting. The quoted error in the zero-recoil form factor $f_+(0) = 0.745(11)$ is significantly smaller than in their 2010 work, but we are unable to understand the sources of this improvement with the limited information provided in Ref. [347]. The preprint does not provide an error budget, nor any information on how the systematic uncertainties are estimated. Thus we cannot rate this calculation, and do not include it in the summary table and plot.

Table 21 summarises the existing $N_\mathrm{f} =2$ and $N_\mathrm{f} = 2+1$ calculations of the $D \rightarrow \pi \ell \nu$ and $D \rightarrow K \ell \nu$ semileptonic form factors. The quality of the systematic error studies is indicated by the symbols. Additional tables in Appendix B.5.2 provide further details on the simulation parameters and comparisons of the error estimates. Recall that only calculations without red tags that are published in a refereed journal are included in the FLAG average. Of the calculations described above, only those of HPQCD 10B,11 satisfy all of the quality criteria. Therefore our average of the $D \rightarrow \pi \ell \nu$ and $D \rightarrow K \ell \nu$ semileptonic form factors from $N_\mathrm{f} = 2+1$ lattice QCD is

98 $$\begin{aligned} N_\mathrm{f}\!=\!2+1: \quad f_+^{D\pi }(0)\! =\! 0.666(29), \quad f_+^{DK}(0) \!= \!0.747(19). \end{aligned}$$

**Fig. 14:**
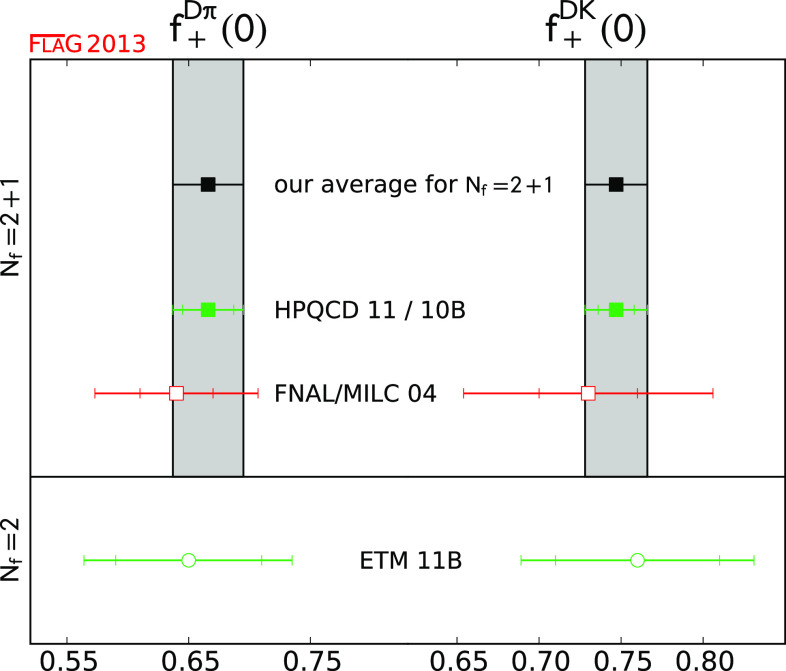
$D\rightarrow \pi \ell \nu$ and $D\rightarrow K\ell \nu$ semileptonic form factors at zero momentum transfer. The HPQCD result for $f_+^{D\pi }(0)$ is from HPQCD 11, the one for $f_+^{DK}(0)$ represents HPQCD 10B (see Table 21)

Figure 14 plots the existing $N_\mathrm{f} =2$ and $N_\mathrm{f} = 2+1$ results for $f_+^{D\pi }(0)$ and $f_+^{DK}(0)$; the grey bands show our average of these quantities. Section 7.3 discusses the implications of these results for determinations of the CKM matrix elements $|V_{cd}|$ and $|V_{cs}|$ and tests of unitarity of the second row of the CKM matrix.

**Table 21:** $D \rightarrow \pi \ell \nu$ and $D\rightarrow K\ell \nu$ semileptonic form factors at zero momentum transfer

Collaboration	Ref.	$N_\mathrm{f}$	Publication status	Continuum extrapolation	Chiral extrapolation	Finite volume	Renormalisation	Heavy-quark treatment	$f_+^{D\pi } (0)$	$f_+^{DK} (0)$
HPQCD 11	[338]	$2+1$	A						0.666 (29)	
HPQCD 10B	[342]	$2+1$	A							0.747 (19)
FNAL/MILC 04	[357]	$2+1$	A						0.64 (3) (6)	0.73 (3) (7)
ETM 11B	[345]	2	C						0.65 (6) (6)	0.76 (5) (5)

### Determinations of $|V_{cd}|$ and $|V_{cs}|$ and test of second-row CKM unitarity

We now interpret the lattice-QCD results for the $D_{(s)}$-meson decay constants and semileptonic form factors as determinations of the CKM matrix elements $|V_{cd}|$ and $|V_{cs}|$ in the Standard Model.

For the leptonic decays, we use the latest experimental averages from Rosner and Stone for the Particle Data Group [114] (where electromagnetic corrections of $\sim$1 % have been removed):

99 $$\begin{aligned}&f_D |V_{cd}| = 46.40(1.98)~\mathrm{MeV},\nonumber \\&f_{D_{s}} |V_{cs}| = 253.1(5.3)~\hbox {MeV}. \end{aligned}$$

We combine these with the average values of $f_D$ and $f_{D_{s}}$ from the individual $N_\mathrm{f} = 2$ and $N_\mathrm{f} = 2+1$ lattice-QCD calculations that satisfy the FLAG criteria, and summarise the results for the CKM matrix elements $|V_{cd}|$ and $|V_{cs}|$ in Table 22. For our preferred values we use the averaged $N_\mathrm{f}=2$ and $N_\mathrm{f} = 2+1$ results for $f_D$ and $f_{D_{s}}$ in Eqs. (93) and (94). We obtain

100 $$\begin{aligned}&|V_{cd}| = 0.2218(35)(95), \quad |V_{cs}| = 1.018 (11)(21),\nonumber \\&\quad (\hbox {leptonic~decays}, N_\mathrm{f}=2+1) \, \end{aligned}$$

101 $$\begin{aligned}&|V_{cd}| = 0.2231(95)(75), \quad |V_{cs}| = 1.012(21)(28),\nonumber \\&\quad (\hbox {leptonic~decays}, N_\mathrm{f}=2) \end{aligned}$$

where the errors shown are from the lattice calculation and experiment (plus non-lattice theory), respectively. For the $N_\mathrm{f} = 2+1$ determinations, the uncertainties from the lattice-QCD calculations of the decay constants are two to three times smaller than the experimental uncertainties in the branching fractions; the lattice central values and errors are dominated by those of the HPQCD calculations. Although the $N_\mathrm{f}=2$ and $N_\mathrm{f} = 2+1$ results for $|V_{cs}|$ are slightly larger than one, they are both consistent with unity within errors.

**Table 22:** Determinations of $|V_{cd}|$ (upper panel) and $|V_{cs}|$ (lower panel) obtained from lattice calculations of $D$-meson leptonic decay constants and semileptonic form factors. The errors shown are from the lattice calculation and experiment (plus non-lattice theory), respectively

Collaboration	Ref.	$N_\mathrm{f}$	From	$|V_{cd}|$ or $|V_{cs}|$
HPQCD 12A	[331]	$2+1$	$f_{D}$	0.2228 (36) (95)
FNAL/MILC 11	[332]	$2+1$	$f_{D}$	0.2120 (109) (91)
HPQCD 11	[338]	$2+1$	$D \rightarrow \pi \ell \nu$	0.2192 (95) (45)
ETM 13B	[335]	2	$f_{D}$	0.2231 (95) (75)
HPQCD 10A	[94]	$2+1$	$f_{D_{s}}$	1.021 (10) (21)
FNAL/MILC 11	[332]	$2+1$	$f_{D_{s}}$	0.9731 (404) (202)
HPQCD 10B	[342]	$2+1$	$D \rightarrow K \ell \nu$	0.9746 (248) (67)
ETM 13B	[335]	2	$f_{D_{s}}$	1.012 (21) (28)

For the semileptonic decays, we use the latest experimental averages from the Heavy Flavour Averaging Group [126]:^31^

102 $$\begin{aligned} f_+^{D\pi }(0) |V_{cd}| = 0.146(3), \quad f_+^{DK}(0) |V_{cs}| = 0.728(5). \end{aligned}$$

For each of $f_+^{D\pi }(0)$ and $f_+^{DK}(0)$, there is only a single $N_\mathrm{f} = 2+1$ lattice-QCD calculation that satisfies the FLAG criteria. Using these results, which are given in Eq. (98), we obtain our preferred values for $|V_{cd}|$ and $|V_{cs}|$:

103 $$\begin{aligned}&|V_{cd}| = 0.2192(95)(45), \quad |V_{cs}| = 0.9746(248)(67), \nonumber \\&\quad (\mathrm{semileptonic~decays}, N_\mathrm{f}=2+1) \end{aligned}$$

where the errors shown are from the lattice calculation and experiment (plus non-lattice theory), respectively.

**Fig. 15:**
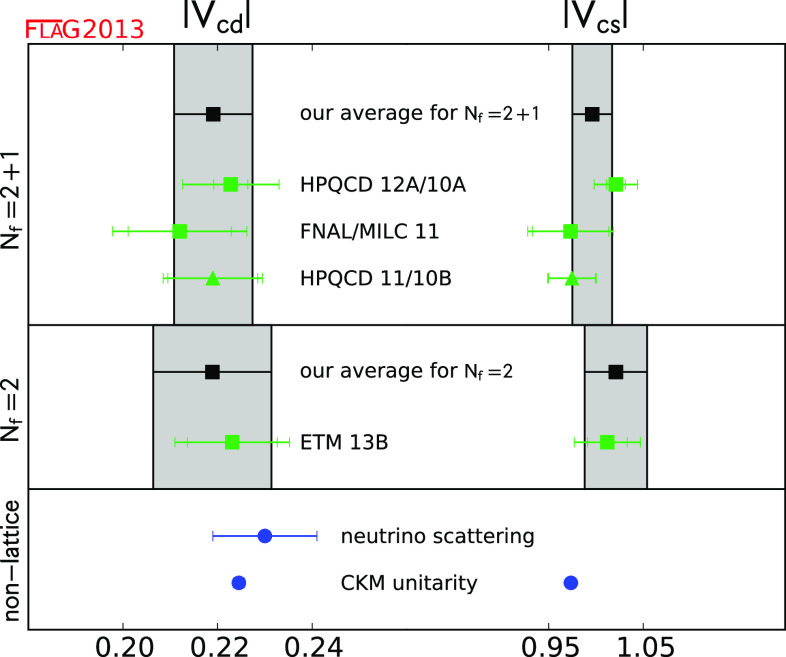
Comparison of determinations of $|V_{cd}|$ and $|V_{cs}|$ obtained from lattice methods with non-lattice determinations and the Standard Model prediction based on CKM unitarity. When two references are listed on a single row, the first corresponds to the lattice input for $|V_{cd}|$ and the second to that for $|V_{cs}|$. The results denoted by *squares* are from leptonic decays, while those denoted by *triangles* are from semileptonic decays

Table 23 summarises the results for $|V_{cd}|$ and $|V_{cs}|$ from leptonic and semileptonic decays, and compares them to determinations from neutrino scattering (for $|V_{cd}|$ only) and CKM unitarity. These results are also plotted in Fig. 15. The determinations of $|V_{cd}|$ all agree within uncertainties, but the errors in the direct determinations from leptonic and semileptonic decays are approximately ten times larger than the indirect determination from CKM unitarity. The determination of $|V_{cs}|$ from $N_\mathrm{f} = 2+1$ lattice-QCD calculations of leptonic decays is noticeably larger than that from both semileptonic decays and CKM unitarity. The disagreement between $|V_{cs}|$ from leptonic and semileptonic decays is slight (only 1.2$\sigma$ assuming no correlations), but the disagreement between $|V_{cs}|$ from leptonic decays and CKM unitarity is larger at 1.9$\sigma$. This tension with CKM unitarity is driven primarily by the HPQCD calculation of $f_{D_{s}}$ in Ref. [94], but we note that the ETM $N_\mathrm{f}=2$ calculation of $f_{D_{s}}$ in Ref. [335] leads to the same high central value of $|V_{cs}|$, just with larger uncertainties. Further, the recent preliminary lattice-QCD calculation of $f_{D_{s}}$ using $N_\mathrm{f} = 2+1+1$ configurations with dynamical HISQ quarks by Fermilab/MILC [329] agrees with the HPQCD result and quotes smaller uncertainties due to the inclusion of data at the physical light-quark mass, so it will be interesting to see how this tension evolves with improved experimental measurements and more independent lattice-QCD results with competitive errors.

**Table 23:** Comparison of determinations of $|V_{cd}|$ and $|V_{cs}|$ obtained from lattice methods with non-lattice determinations and the Standard Model prediction assuming CKM unitarity

	From	Ref.	$|V_{cd}|$	$|V_{cs}|$
$N_\mathrm{f} = 2+1$	$f_D$ and $f_{D_{s}}$		0.2218 (101)	1.018 (24)
$N_\mathrm{f} = 2$	$f_D$ and $f_{D_{s}}$		0.2231 (121)	1.012 (35)
$N_\mathrm{f} = 2+1$	$D \rightarrow \pi \ell \nu$ and $D\rightarrow K \ell \nu$		0.2192 (105)	0.9746 (257)
PDG	Neutrino scattering	[74]	0.230 (11)	
Rosner 12 (*for the* PDG)	CKM unitarity	[114]	0.2245 (12)	0.97345 (22)

The $N_\mathrm{f}=2+1$ averages for $|V_{cd}|$ and $|V_{cs}|$ in Fig. 15 are obtained by averaging the results in Table 22 including correlations. We assume that the statistical errors are 100 % correlated between all of the calculations because they use the MILC Asqtad gauge configurations. We also assume that the heavy-quark discretisation errors are 100 % correlated between the HPQCD calculations of leptonic and semileptonic decays because they use the same charm-quark action, and that the scale-setting uncertainties are 100 % correlated between the HPQCD results as well. Finally, we include the 100 % correlation between the experimental inputs for the two extractions of $|V_{cd(s)}|$ from leptonic decays. We obtain

104 $$\begin{aligned}&|V_{cd}| = 0.2191(83), \quad |V_{cs}| = 0.996(21), \nonumber \\&\quad (\mathrm{our\ average}, N_\mathrm{f}=2+1) \end{aligned}$$

where the errors include both theoretical and experimental uncertainties, and the error on $|V_{cs}|$ has been increased by $\sqrt{\chi ^2/\hbox {dof}}=1.03$.

Using the determinations of $|V_{cd}|$ and $|V_{cs}|$ in Eq. (104), we can test the unitarity of the second row of the CKM matrix. We obtain

105 $$\begin{aligned} |V_{cd}|^2 + |V_{cs}|^2 + |V_{cb}|^2 - 1 = 0.04(6) \end{aligned}$$

which agrees with the Standard Model at the percent level. Given the current level of precision, this result does not depend on the value used for $|V_{cb}|$, which is of ${\mathcal {O}}(10^{-2})$ [see Eq. (162)].

## $B$-meson decay constants, mixing parameters and form factors

Leptonic and semileptonic decays of bottom $B$- and $B_{s}$-mesons probe the quark-flavour-changing transitions $b \rightarrow u$ and $b \rightarrow c$. Tree-level semileptonic $B$ decays with light charged leptons ($\ell = e,\mu$) in the final state, such as $B \rightarrow \pi \ell \nu$ and $B \rightarrow D^{(*)}\ell \nu$, enable determinations of the CKM matrix elements $|V_{ub}|$ and $|V_{cb}|$ within the Standard Model. Semileptonic $B$ decays that occur via loops in the Standard Model, such as $B\rightarrow K^{(*)} \ell ^+ \ell ^-$, provide sensitive probes of physics beyond-the-Standard Model because contributions from new heavy particles in the loops may be comparable to the Standard Model “background.” Further, because $B$-mesons are sufficiently massive, they can decay to final states involving $\tau$-leptons. Tree-level decays such as $B\rightarrow \tau \nu$ and $B\rightarrow D^{(*)}\tau \nu$ are promising new-physics search channels because they can receive significant contributions from charged-Higgs bosons.

Mixing of neutral $B^0_{d}$- and $B^0_{s}$-mesons occurs in the Standard Model via one-loop box diagrams containing up-type quarks ($u,c,t$) and charged $W$ bosons. Because the Standard Model contributions are proportional to the CKM factors $|V_{u(c,t)q}V_{u(c,t)b}^*|^2$ (where $q=d,s$) and the quark masses $m_{u(c,t)}^2$, neutral $B$-meson mixing is dominated by intermediate top quarks. Thus experimental measurements of the neutral $B^0_{d(s)}$-meson oscillation frequencies, $\Delta M_{d(s)}$ combined with sufficiently precise theoretical calculations of the hadronic mixing matrix elements (often presented as dimensionless “bag” parameters), enable the determination of the CKM matrix elements $|V_{td}|$ and $|V_{ts}|$ within the Standard Model. Conversely, neutral $B$-meson mixing places stringent constraints on the scale of generic new heavy particles that can enter the loops in beyond-the-Standard Model scenarios. Finally, neutral meson mixing is also sensitive to the phase of the CKM matrix $(\rho , \eta )$. Thus the ratio of oscillation frequencies $\Delta M_{d} / \Delta M_{s}$ places a tight constraint on the apex of the CKM unitarity triangle that is complementary to those from other observables.

Lattice-QCD calculations of $b$ quarks have an added complication not present for charm and light quarks: at the lattice spacings that are currently used in numerical simulations, the $b$ quark mass is of order one in lattice units. Therefore a direct treatment of $b$ quarks with the fermion actions commonly used for light quarks will result in large cutoff effects, and all current lattice-QCD calculations of $b$ quark quantities make use of effective field theory at some stage. The two most widely used general approaches for lattice $b$ quarks are (i) direct application of effective field theory treatments such as HQET or NRQCD, which allow for a systematic expansion in $1/m_{b}$; or (ii) the interpretation of a relativistic quark action in a manner suitable for heavy quarks using an extended Symanzik improvement program to suppress cutoff errors. This introduces new systematic uncertainties that are not present in light-quark calculations, either from truncation of the effective theory, or from more complicated lattice-spacing dependence. Further, because with these approaches the light and bottom quarks are simulated with different fermion actions, it is in general not possible to construct absolutely normalised bottom-light currents; this leads to systematic uncertainties due to matching the lattice operators to the continuum that can be significant. A third approach is to use an improved light-quark action to calculate the quantity of interest over a range of heavy-quark masses with $am_{h} < 1$, and then to use heavy-quark effective theory and/or knowledge of the static limit to extrapolate or interpolate to the physical $b$-quark mass. Such methods can avoid some of the aforementioned complications, but they require simulations at very small lattice spacings in order to keep discretisation errors under control. Appendix A.1.3 reviews the methods used to treat $b$ quarks on the lattice in more detail.

Here we summarise the status of lattice-QCD calculations of the bottom leptonic decay constants, neutral meson mixing parameters, and semileptonic form factors. We limit our review to results based on modern simulations with reasonably light pion masses (below approximately 500 MeV). This excludes results obtained from the earliest unquenched simulations, which typically had two flavours in the sea, and which were limited to heavier pion masses because of the constraints imposed by the computational resources and methods available at that time. Fewer collaborations have presented results for these quantities than for the light-quark sector ($u$, $d$, $s$), and the calculations tend to be on coarser lattice spacings with heavier pions. Therefore, for some quantities, there is only a single lattice calculation that satisfies the criteria to be included in our average. Several collaborations, however, are currently pursuing the needed matrix-element calculations with different lattice $b$-quark actions, finer lattice spacings, and lighter pions, so we expect the appearance of many new results with controlled errors in the next year or two.

We also note that the heavy-quark methods discussed in this review have been validated in a number of ways. Because several groups use the same action for charm and bottom quarks, tests of such methods with charm quarks are relevant for $B$ physics results, and they are therefore included in the following discussion. Calculations of hadron masses with one or more heavy (charm or bottom) valence quark provide phenomenological tests of the heavy-quark action. Such calculations have been performed with NRQCD, HQET, Fermilab, RHQ, Tsukuba, HISQ, Overlap, twisted-mass Wilson, and other $\mathcal{O}(a)$ improved Wilson heavy quarks for the hyperfine splittings in the $D_{(s)}$- and $B_{(s)}$-meson systems [94, 333, 363–374], and for the low-lying charmonium [333, 367, 368, 371, 375–379], bottomonium [380–386], and $B_{c}$ [364, 369, 387–389] systems. All of them are in good agreement with experimental measurements. Hyperfine splittings are sensitive to higher-order terms in the heavy-quark action and therefore provide particularly good tests of such terms. The comparison of lattice-QCD calculations of hadronic matrix elements for leptonic and radiative decays in charmonium [377, 389] with experimental measurements provides CKM-free tests of heavy-HISQ currents. The comparison of lattice-QCD calculations of the shape of the semileptonic form factors for $D \rightarrow \pi (K) \ell \nu$ [357] with experimental measurements provides CKM independent tests of charm-quark currents with the Fermilab action. In two of the above mentioned tests, the lattice-QCD calculations were predictions, in one case predating the experimental discovery of the $B_{c}$ mass, and in the other predating experimental measurements of the shape of the semileptonic $D$-meson form factors with comparable precision. Truncation errors in HQET have been studied by comparing simulations of the effective field theory with corresponding quenched simulations using a non-perturbatively improved Wilson action with heavy quark masses in the charm-mass region in large volumes [390] and up to the $b$-quark mass in small volumes [391]. Moreover, the consistency between independent determinations of the bottom [73, 336, 365, 370, 392–394] and charm [60, 72, 73, 85, 333, 395, 396] quark masses using NRQCD, HQET, Tsukuba, HISQ, twisted-mass Wilson, and other $\mathcal{O}(a)$ improved Wilson heavy quarks, as well as their agreement with non-lattice determinations [74] further validate lattice heavy-quark methods.

Following our review of lattice-QCD calculations of $B_{(s)}$-meson leptonic decay constants, neutral meson mixing parameters, and semileptonic form factors, we then interpret our results within the context of the Standard Model. We combine our best-determined values of the hadronic matrix elements with the most recent experimentally measured branching fractions to obtain $|V_{(u)cb}|$ and compare these results to those obtained from inclusive semileptonic $B$ decays.

### Leptonic decay constants $f_B$ and $f_{B_{s}}$

The $B$- and $B_{s}$-meson decay constants are relevant for decays of charged $B$-mesons to a lepton–neutrino pair via the charged current interaction, as well as for rare leptonic decays of neutral $B_{d(s)}$-mesons to a charged-lepton pair via a flavour-changing neutral-current (FCNC) interaction.

In the Standard Model the decay rate for $B^+ \rightarrow \ell ^+ \nu _{\ell }$ is given by a formula identical to the one for $D$ decays in Eq. (91) but with $D_{(s)}$ replaced by $B$ and the relevant CKM matrix element $V_{cq}$ replaced by $V_{ub}$:

106 $$\begin{aligned} \Gamma ( B \rightarrow \ell \nu _{\ell } ) = \frac{ m_B}{8 \pi } G_F^2 f_B^2 |V_{ub}|^2 m_{\ell }^2 \left( 1-\frac{ m_{\ell }^2}{m_B^2} \right) ^2. \end{aligned}$$

The only charged-current $B$-meson decay that has been observed so far is $B \rightarrow \tau \nu _{\tau }$, which has been measured by the Belle and Babar collaborations with a combined precision of 20 % [74]. This measurement can therefore be used to determine $|V_{ub}|$ when combined with lattice-QCD predictions of the corresponding decay constant.

The decay of a neutral $B_{d(s)}$-meson to a charged lepton pair is loop-suppressed in the Standard Model. The corresponding expression for the branching fraction has the form

107 $$\begin{aligned} B ( B_{q} \rightarrow \ell ^+ \ell ^-)&= \tau _{B_{q}} \frac{G_F^2}{\pi } \, Y \, \left( \frac{\alpha }{4 \pi \sin ^2 \Theta _W} \right) ^2 \nonumber \\&\times m_{B_{q}} f_{B_{q}}^2 |V_{tb}^*V_{tq}|^2 m_{\ell }^2 \sqrt{1\!-\! 4 \frac{m_{\ell }^2}{m_B^2} }, \end{aligned}$$

where the light quark $q=s$ or $d$, and the loop function $Y$ includes NLO QCD and electroweak corrections [397]. Evidence for $B_{s} \rightarrow \mu ^+ \mu ^-$ decay was recently seen at LHCb at the $3.5 \sigma$ level, with a branching fraction of $BR(B_{s} \rightarrow \mu ^+ \mu ^-) = (3.2^{+1.5}_{-1.2}) \,10^{-9}$ [398].

The decay constants $f_{B_{q}}$ (with $q=u,d,s$) parameterise the matrix elements of the corresponding axial-vector currents, $A^{\mu }_{bq} = \bar{b}\gamma ^{\mu }\gamma ^5q$, analogously to the definition of $f_{D_{q}}$ in Sect. 7.1:

108 $$\begin{aligned} {\langle }0| A^{\mu } | B_{q}(p) {\rangle }= p_B^{\mu } f_{B_{q}}. \end{aligned}$$

For heavy–light mesons, it is convenient to define and analyse the quantity

109 $$\begin{aligned} \Phi _{B_{q}} \equiv f_{B_{q}} \sqrt{m_{B_{q}}}, \end{aligned}$$

which approaches a constant (up to logarithmic corrections) in the $m_B \rightarrow \infty$ limit. In the following discussion we denote lattice data for $\Phi$($f$) obtained at a heavy quark mass $m_{h}$ and light valence-quark mass $m_{\ell }$ as $\Phi _{h\ell }$($f_{hl}$), to differentiate them from the corresponding quantities at the physical $b$ and light-quark masses.

The SU(3)-breaking ratio $f_{B_{s}}/f_B$ is an interesting quantity to study with lattice QCD, since most systematic errors partially cancel in this ratio, including discretisation errors, heavy-quark mass tuning effects, and renormalisation errors, among others. The SU(3)-breaking ratio is, however, sensitive to the chiral extrapolation. So one can, in principle, combine a lattice-QCD calculation of the SU(3)-breaking ratio that includes a careful study of the chiral extrapolation, with a different lattice-QCD calculation of $f_{B_{s}}$ (which is relatively insensitive to chiral extrapolation errors) that includes a careful study of all other systematic errors to obtain a more precise result for $f_B$ than would be possible from either lattice-QCD calculation alone. Indeed, this strategy is used by both the ETM and HPQCD collaborations, as described below.

**Fig. 16:**
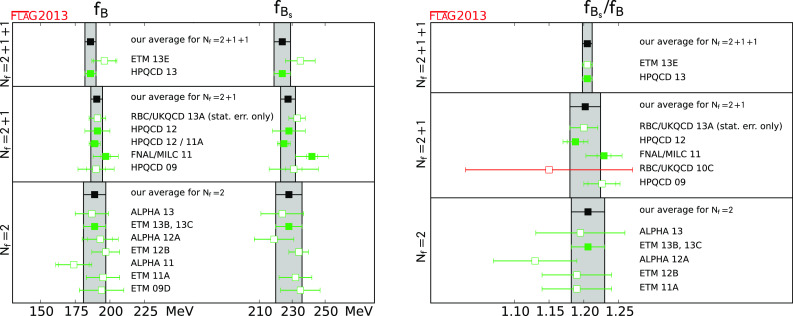
Decay constants of the $B$- and $B_{s}$-mesons. The values are taken from Table 24 (the $f_B$ entry for FNAL/MILC 11 represents $f_{B^+}$). The significance of the colours is explained in Sect. 2. The *black squares* and *grey bands* indicate our averages in Eqs. (110), (111) and (112)

A number of different heavy-quark formulations are being used to obtain results for $B_{q}$-meson decay constants from numerical simulations with $N_\mathrm{f}=2$, $N_\mathrm{f}=2+1$ and $N_\mathrm{f}=2+1+1$ sea quarks. They are summarised in Tables 24 and 25 and in Fig. 16. Additional details as regards the underlying simulations and systematic error estimates are given in Appendix B.6.1.

**Table 24:** Decay constants of the $B$, $B^+$, $B^0$ and $B_{s}$ mesons (in MeV). Here $f_B$ stands for the mean value of $f_{B^+}$ and $f_{B^0}$, extrapolated (or interpolated) in the mass of the light valence-quark to the physical value of $m_{ud}$

Collaboration	Ref.	$N_\mathrm{f}$	Publication status	Continuum extrapolation	Chiral extrapolation	Finite volume	Renormalisation/ matching	Heavy quark treatment	$f_{B^+}$	$f_{B^0}$	$f_{B}$	$f_{B_{s}}$
ETM 13E	[399]	$2+1+1$	C						–	–	196 (9)	235 (9)
HPQCD 13	[400]	$2+1+1$	A						184 (4)	188 (4)	186 (4)	224 (5)
RBC/UKQCD 13A	[401]	$2+1$	C						–	–	191 (6)$_\mathrm{stat}^\diamond$	233 (5)$_\mathrm{stat}^\diamond$
HPQCD 12	[402]	$2+1$	A						–	–	191 (9)	228 (10)
HPQCD 12	[402]	$2+1$	A						–	–	189 (4)$^\triangle$	–
HPQCD 11A	[366]	$2+1$	A						–	–	–	225 (4)$^\nabla$
FNAL/MILC 11	[332]	$2+1$	A						197 (9)	–	–	242 (10)
HPQCD 09	[403]	$2+1$	A						–	–	190 (13)$^\bullet$	231 (15)$^\bullet$
ALPHA 13	[404]	2	C						–	–	187 (12) (2)	224 (13)
ETM 13B, 13C	[335, 405]	2	P$^\dagger$						–	–	189 (8)	228 (8)
ALPHA 12A	[370]	2	C						–	–	193 (9) (4)	219 (12)
ETM 12B	[393]	2	C						–	–	197 (10)	234 (6)
ALPHA 11	[365]	2	C						–	–	174 (11) (2)	–
ETM 11A	[336]	2	A						–	–	195 (12)	232 (10)
ETM 09D	[392]	2	A						–	–	194 (16)	235 (12)

$^\diamond$ Statistical errors only

$^\triangle$ Obtained by combining $f_{B_{s}}$ from HPQCD 11A with $f_{B_{s}}/f_B$ calculated in this work

$^\nabla$ This result uses one ensemble per lattice spacing with light to strange sea-quark mass ratio $m_{\ell }/m_{s} \approx 0.2$

$^\bullet$ This result uses an old determination of $r_1=0.321(5)$ fm from Ref. [380] that has since been superseded

$^\dagger$ Update of ETM 11A and 12B

**Table 25:** Ratios of decay constants of the $B$ and $B_{s}$ mesons (for details see Table 24)

Collaboration	Ref.	$N_\mathrm{f}$	Publication status	Continuum extrapolation	Chiral extrapolation	Finite volume	Renormalisation/ matching	Heavy quark treatment	$f_{B_{s}}/f_{B^+}$	$f_{B_{s}}/f_{B^0}$	$f_{B_{s}}/f_{B}$
ETM 13E	[399]	$2+1+1$	C						–	–	1.201 (25)
HPQCD 13	[400]	$2+1+1$	A						1.217 (8)	1.194 (7)	1.205 (7)
RBC/UKQCD 13A	[401]	$2+1$	C						–	–	1.20 (2)$_\mathrm{stat}^\diamond$
HPQCD 12	[402]	$2+1$	A						–	–	1.188 (18)
FNAL/MILC 11	[332]	$2+1$	A						1.229 (26)	–	–
RBC/UKQCD 10C	[406]	$2+1$	A						–	–	1.15 (12)
HPQCD 09	[403]	$2+1$	A						–	–	1.226 (26)
ALPHA 13	[404]	2	C						–	–	1.195 (61) (20)
ETM 13B, 13C	[335, 405]	2	P$^\dagger$						–	–	1.206 (24)
ALPHA 12A	[370]	2	C						–	–	1.13 (6)
ETM 12B	[393]	2	C						–	–	1.19 (5)
ETM 11A	[336]	2	A						–	–	1.19 (5)

$^\diamond$ Statistical errors only

$^\dagger$ Update of ETM 11A and 12B

The ETM collaboration has presented a series of calculations of the $B$-meson decay constants based on simulations with $N_\mathrm{f}=2$ sea quarks [335, 336, 392, 393, 405]. Three lattice spacings in the range $a \approx 0.067$–0.098 fm are used in ETM 09D [392]. In ETM 11A, ETM 12B, and ETM 13B, 13C [335, 336, 393, 405] additional ensembles at $a\approx 0.054$ fm are included. The valence and sea quarks are simulated with two different versions of the twisted-mass Wilson fermion action. In ETM 09D and ETM 11A the heavy-quark masses are in the charm region and above while keeping $am_{h} \lesssim 0.5$. ETM 12B includes slightly heavier masses than ETM 09D and ETM 11A, while ETM 13B, 13C includes masses as heavy as $am_{h} \sim 0.85$ at the largest two lattice spacings. In ETM 11A two methods are used to obtain $f_{B_{(s)}}$ from their heavy Wilson data: the ratio and the interpolation methods. In the interpolation method they supplement their heavy Wilson data with a static limit calculation. In the ratio method (see Appendix A.1.3) they construct ratios (called $z_{(s)}$) from a combination of the decay constants $f_{h\ell (s)}$ and the heavy-quark pole masses that are equal to unity in the static limit. Ratios of pole-to-${\overline{\mathrm{MS}}}$ massconversion factors are included at NLO in continuum perturbation theory. ETM 09D, ETM 12B and ETM 13B, 13C use only the ratio method. Finally, ETM analyses the SU(3)-breaking ratio $\Phi _{hs}/\Phi _{h\ell }$ (or the ratio of ratios, $z_{s}/z$) and combines it with $\Phi _{hs}$ or ($z_{s}$) to obtain $f_B$, instead of directly extracting it from their $\Phi _{h\ell }$ (or $z$) data. In ETM 11A, ETM 12B, and ETM 13B, 13C the data are interpolated to a fixed set of reference masses on all ensembles, and subsequently extrapolated to the continuum and to the physical light-quark masses in a combined fit. The static limit calculation for the interpolation method in ETM 11A is done at two intermediate lattice spacings, $a \approx 0.085, 0.067$ fm. The results from the interpolation method have larger (statistical and systematic) errors than those from the ratio method, since statistical and systematic errors tend to cancel in the ratios. The observed discretisation effects (as measured by the percentage difference between the lattice data at the smallest lattice spacing and the continuum extrapolated results) are smaller than what would be expected from power-counting estimates. Over the range of heavy quark masses used in their simulations ETM finds discretisation errors $\lesssim$3 % for $\Phi _{hs}$ and $\lesssim$1.5 % for the ratio $z_{s}$. As a result, the dominant error on $f_{B_{s}}$ is the statistical (combined with the chiral and continuum extrapolation and heavy quark interpolation) uncertainty, whereas the dominant error on the SU(3) breaking ratio is due to the chiral extrapolation.

The ALPHA collaboration calculates the $B$- and $B_{s}$-meson decay constants at the physical $b$-quark mass using non-perturbative lattice HQET through $\mathcal{O}(1/m_{h})$ on ensembles with $N_\mathrm{f}=2$ non-perturbatively $\mathcal{O}(a)$ improved Wilson quarks at three lattice spacings in the range $a \approx 0.048$–0.075 fm. The parameters of the HQET action and the static-current renormalisation are determined non-perturbatively in a separate matching calculation using small physical volumes ($L\simeq 0.4$ fm) with Schrödinger functional boundary conditions together with a recursive finite-size scaling procedure to obtain the non-perturbative parameters at the large physical volumes used in the simulations. In ALPHA 11 [365] ensembles with pion masses in the range $m_{\pi } \approx 440$–270 MeV are used. ALPHA 12A [370] and ALPHA 13 [404] include an ensemble at a lighter sea-quark mass corresponding to $m_{\pi } \approx 190$ MeV. ALPHA 11 presents results for $f_B$ only, while ALPHA 12A also presents a preliminary result for $f_{B_{s}}$, and ALPHA 13 presents the collaboration’s final results for $f_B$, $f_{B_{s}}$ and $f_{B_{s}}/f_B$. The combined statistical and extrapolation errors are of order 5–6 % in these calculations, and are larger than the chiral fit uncertainty. Truncation errors which are ${\mathcal O}(\Lambda _\mathrm{QCD}/m_{h})^2$ are not included in this error budget. Simple power-counting would suggest that they are $\approx$1–4 %. However, the results from both the ETM collaboration discussed above and the HPQCD collaboration (from their heavy HISQ analysis) discussed below, as well as results obtained by ALPHA in the quenched approximation [390] indicate that ${\mathcal O}(\Lambda _\mathrm{QCD}/m_{h})^2$ effects are probably quite small for heavy–light decay constants at the physical $b$-quark mass.

In summary, for the $N_\mathrm{f}=2$ case, only ETM’s results qualify for averaging, since ALPHA’s results have appeared in conference proceedings only so far. Since ETM 13B, 13C updates the published ETM 11A results, we use it for our average:

110 $$\begin{aligned}&N_\mathrm{f}=2: f_B = (189 \pm 8) \,{\mathrm {MeV}}, f_{B_{s}} = (228 \pm 8) \,{\mathrm {MeV}},\nonumber \\&\quad f_{B_{s}}/f_B = 1.206 \pm 0.024. \end{aligned}$$

For the $N_\mathrm{f}=2+1$ case there are currently four published papers describing lattice-QCD calculations of $f_{B_{(s)}}$ performed by two different groups: FNAL/MILC and HPQCD. The HPQCD collaboration has published several calculations of the $B$-meson decay constants with NRQCD $b$ quarks [402, 403]. In Ref. [403] (HPQCD 09) they use Asqtad light valence quarks, and include ensembles at two lattice spacings $a\approx 0.12, 0.09$ fm and sea quarks with minimum RMS sea-pion masses $m_{\pi , \mathrm{RMS}} \approx 400$ MeV equal to the light sea-quark masses. In Ref. [402] HISQ light valence quarks are employed instead. This analysis uses the same Asqtad ensembles as in HPQCD 09 but includes an additional ensemble at $a \approx 0.09$ fm at a lighter sea-quark mass, so that the minimum RMS sea-pion mass is approximately $320$ MeV. The HISQ light valence masses are matched to the Asqtad sea-quark masses via the ratio $m_{\ell }/m_{s}$. The dominant systematic error in both calculations is due to using one-loop mean-field improved lattice perturbation theory for the current renormalisation. In both calculations, HPQCD performs a combined chiral and continuum extrapolation of the data, in the first case using NLO (full QCD) heavy-meson rooted staggered $\chi$PT (HMrS$\chi$PT) and in the latter case using NLO continuum partially quenched HM$\chi$PT, supplemented in both cases by NNLO analytic and generic discretisation terms. HPQCD finds a significant reduction in discretisation errors in their calculation with HISQ light valence quarks, as compared to their calculation with Asqtad valence quarks. Indeed, in HPQCD 12 the continuum-extrapolated results overlap within errors with the data at finite lattice spacing.

Another calculation of the $B_{s}$-meson decay constant is presented by the HPQCD collaboration in Ref. [366], this time using the HISQ action for the strange and heavy valence quarks, i.e. the heavy HISQ method. This analysis includes Asqtad ensembles over a large range of lattice spacings, $a\approx 0.15$–0.045 fm and heavy-quark masses in the range $am_{h} \approx 0.2$–0.85. Only one sea-quark ensemble per lattice spacing is included in this analysis, all with a sea-quark to strange-quark mass ratio of $m_{\ell }/m_{s} \approx 0.2$, yielding a minimum RMS sea-pion mass of approximately $330$ MeV. The sea-quark mass dependence is assumed to be negligible, which is based on the analysis of $f_{D_{s}}$ in Ref. [94]. HPQCD uses an HQET-type expansion in $1/m_H$ (where $m_H$ is the mass of an $h$-flavoured meson) with coefficients that are polynomials in $am_{h}$, $a\Lambda$ and $am_{s}$ to perform a combined fit to all their data, including terms up to $1/m_H^3$, $(am_{h})^6$, $(a\Lambda )^6$ and $(am_{s})^6$. The continuum-extrapolated fit curve is then used to obtain the decay constant at the physical $B_{s}$-meson mass, which requires another small extrapolation. As can be seen in Figure 1 of Ref. [366], discretisation errors (as measured by the percentage difference between the lattice data and the continuum fit curve) are smaller for a given value of $am_{h}$ when $m_H$ is larger. This somewhat counterintuitive result for an action that formally contains discretisation errors of $\mathcal{O}(am_{h})^2$ is likely due to coefficients in the form of powers of $v/c$ that suppress these errors. After statistical (and extrapolation) errors, the largest sources of uncertainty in this analysis are discretisation and heavy-quark extrapolation errors. They are estimated by varying the fit Ansatz and by excluding data at the largest and smallest lattice spacings as well as data at the largest values of $am_{h}$.

The Fermilab Lattice and MILC collaborations present a lattice-QCD calculation of the $D$- and $B$-meson decay constants in Ref. [332], which uses the Fermilab method for the heavy ($b$ and $c$) valence quarks together with Asqtad light and strange valence quarks on a subset of the MILC Asqtad $N_\mathrm{f}=2+1$ ensembles. The current renormalisations are calculated using a mostly non-perturbative renormalisation (mNPR) method. Their estimate of the perturbative errors for the small perturbative correction factors calculated at one loop in mean-field improved lattice perturbation theory are comparable to the size of actual one-loop corrections. The simulations include lattice spacings in the range $a \approx 0.15$–0.09 fm and a minimum RMS pion mass of approximately $320$ MeV. In this calculation lattice data at 9–12 valence light-quark masses are generated for each sea-quark ensemble. The chiral- and continuum-extrapolated results are obtained from combined chiral and continuum fits. The chiral fit function uses NLO partially quenched HMrS$\chi$PT including $1/m_{h}$ terms and supplemented by NNLO analytic terms. Also included are light-quark as well as heavy-quark discretisation terms. The dominant uncertainties after statistical errors are due to heavy-quark discretisation effects, heavy-quark mass tuning, and correlator fit errors. A calculation of the $B$- and $D$-meson decay constants using Fermilab heavy quarks on the full set of Asqtad ensembles is still in progress [407].

The RBC/UKQCD collaboration has presented a result for the SU(3) breaking ratio in Ref. [406] using a static-limit action on $N_\mathrm{f}=2+1$ domain wall ensembles at a single lattice spacing $a \approx 0.11$ fm with a minimum pion mass of approximately $430$ MeV. They use both HYP and APE smearing for the static action and one-loop mean-field improved lattice perturbation theory to renormalise and improve the static-limit current. Their static-limit action and current do not, however, include $1/m_{h}$ effects. Reference [406] includes an estimate of this effect via power counting as $\mathcal{O}((m_{s}-m_{d})/m_{b})$ in the error budget. The statistical errors in this work are significantly larger ($\sim$5–8 %), as are the chiral-extrapolation errors ($\sim$7 %), due to the rather large pion masses used in this work. With data at only one lattice spacing, discretisation errors cannot be estimated from the data. A power counting estimate of this error of 3 % is included in the systematic error budget. An update of this work was presented at the Lattice 2013 conference [408], where the new analysis includes ensembles at two lattice spacings and with smaller pion masses, as well as calculations of the decay constants themselves. However, Ref. [408] did not appear until after the closing deadline and is therefore not included in this review. The RBC/UKQCD collaboration has also presented preliminary calculations of the $B$-meson decay constants using the RHQ action (another relativistic heavy-quark action) [401, 409] on $N_\mathrm{f}=2+1$ domain-wall ensembles at two lattice spacings, $a \approx 0.086, 0.11$ fm with sea-pion masses in the range $m_{\pi } \approx 420$–290 MeV. The parameters of the RHQ action are tuned non-perturbatively, and the axial-vector current is renormalised using the mNPR method. Results are quoted with statistical errors only [401] after a combined chiral-continuum extrapolation using SU(2) HM$\chi$PT and a term linear in $a^2$. A complete systematic error analysis is still in progress.

In summary, for the $N_\mathrm{f}=2+1$ case there currently are four different results for the $B$- and $B_{s}$-meson decay constants and three different results for the SU(3)-breaking ratio that satisfy the quality criteria (see Tables 24, 25). However, they all use overlapping subsets of MILC Asqtad ensembles. We therefore treat the statistical errors between the results as 100 % correlated. Furthermore, one of the results for $f_B$ in HPQCD 12 [402] is obtained by combining HPQCD 12’s result for the ratio $f_{B_{s}}/f_B$ using NRQCD $b$ quarks with HPQCD 11A’s result for $f_{B_{s}}$. However, no itemised error budget is given for the so-combined $f_B$ result. In order to include sensible correlations between the two HPQCD results for $f_B$, we construct an itemised error budget for the combined $f_B$ from the individual itemised error budgets, by adding the itemised errors in quadrature. This is conservative, because the resulting total uncertainty on the combined $f_B$ is slightly larger than the quoted uncertainty in Ref. [402], 4.3 MeV compared to 4 MeV. We then treat the chiral-extrapolation errors, the light-quark discretisation errors, the scale-setting errors, and renormalisation errors as 100 % correlated between the two $f_B$ results in HPQCD 12. Finally, the HPQCD 09 result was obtained using a value for the scale $r_1$ that has since been superseded. We drop this result from the average, since it is effectively updated by HPQCD 12. We find

111 $$\begin{aligned}&N_\mathrm{f}=2+1: f_B = (190.5 \pm 4.2) \,{\mathrm {MeV}},\nonumber \\&f_{B_{s}} = (227.7 \pm 4.5) \,{\mathrm {MeV}}, f_{B_{s}}/f_B \!=\! 1.202 \pm 0.022. \end{aligned}$$

The uncertainties on the averages for $f_{B_{s}}$ and for the SU(3) breaking ratio $f_{B_{s}}/f_B$ include PDG rescaling factors of $1.1$ and $1.3$, respectively.

Finally, the first published results for $$B$$-meson decay constants with $$N_\mathrm{f}=2+1+1$$ sea quarks are presented by the HPQCD collaboration [400] (HPQCD 13) using the MILC HISQ ensembles at three lattice spacings, $$a\approx 0.15, 0.12, 0.09$$ fm, where at each lattice spacing one ensemble with Goldstone pions at the physical value is included. HPQCD 13 uses NRQCD $$b$$ quarks and HISQ light valence quarks. The combined chiral interpolation and continuum extrapolation is performed using NLO (full QCD) HM$$\chi $$PT, supplemented by generic discretisation terms of $$O(a^2,a^4)$$. HPQCD also performs a continuum extrapolation of the data at the physical point only, with results that are in good agreement with the extrapolated results obtained from the full data set. The dominant systematic error in this calculation is due to using one-loop mean-field improved lattice perturbation theory for the current renormalisation. In HPQCD 13 it is estimated at 1.4 %, almost a factor of 3 smaller than in HPQCD 12, after reorganizing the perturbative series similar to the mNPR method, and using the fact that the heavy–heavy NRQCD temporal vector current is absolutely normalised and that the light–light HISQ vector current has a small one-loop correction. The next largest uncertainties are due to heavy-quark truncation effects and statistics and scale setting. In this work the scale is set using the $$\Upsilon (2S$$–$$1S)$$ splitting calculated in Ref. [383] without using $$r_1$$ to set the relative scale between ensembles at different lattice spacings, as was done in previous HPQCD work.

Most recently, the ETM collaboration presented their new results for $B$-meson decay constants on their $N_\mathrm{f}=2+1+1$ ensembles in ETM 13E [399]. This work uses the same methods as ETM’s $N_\mathrm{f}=2\,B$-meson decay-constant analyses. In particular, different versions of twisted-mass Wilson actions are used for sea and valence quarks. The decay constants are calculated with the ratio method using heavy-quark masses in the charm region and above while keeping $am_{h} \lesssim 0.8$. ETM 13E includes ensembles with lattice spacings in the range $a \approx 0.062$–0.089 fm and with sea-pion masses in the range $m_{\pi } \approx 211$–443 MeV which are used for combined chiral–continuum extrapolations. As before, the ratio data for $z_{s}$ show small discretisation effects. Somewhat larger discretisation effects are observed, however, for the decay-constant data at the charm-quark mass, since the smallest lattice spacing for the $N_\mathrm{f}=2+1+1$ ensembles is larger than for $N_\mathrm{f}=2$.

In summary, for the $N_\mathrm{f}=2+1+1$ case, the only published results are from HPQCD 13, which therefore form our average:

112 $$\begin{aligned} N_\mathrm{f}&= 2+1+1: f_B = (186 \pm 4) \,{\mathrm {MeV}},\nonumber \\ f_{B_{s}}&= (224 \pm 5) \,{\mathrm {MeV}}, f_{B_{s}}/f_B = 1.205 \pm 0.007. \end{aligned}$$

A comparison of all $N_\mathrm{f}=2$, $N_\mathrm{f}=2+1$ and $N_\mathrm{f}=2+1+1$ lattice-QCD results for $f_B$, $f_{B_{s}}$ and their ratio is shown in Fig. 16. The averages presented in Eqs. (110), (111) and (112) are represented by the grey bands in the figures.

A final comment concerns which light valence-quark mass is used for the chiral extrapolations (or interpolations) to the physical point. First, we note that all the results discussed in this review use simulations with degenerate up and down sea-quark masses. However, since the observed sea-quark mass dependence is much smaller than the valence-quark mass dependence, the dominant contribution to differences between $B^+$- and $B^0$-meson quantities is due to the light valence quarks. Almost all the results quoted in this review are obtained from chiral extrapolations to the average of the up- and down-quark masses, and therefore correspond to the average of the $B^0$- and $B^+$-meson decay constants. The exceptions are FNAL/MILC 11 and HPQCD 13 which both quote results for the $B^+$ meson decay constant from chiral extrapolations (interpolations) of the light valence-quark to the physical up-quark mass. HPQCD 13 also quotes results for the $B^0$-meson decay constant from chiral interpolations to the physical down-quark mass as well as results for the average of the $B^+$- and $B^0$-mesons. The $N_\mathrm{f} = 2+1$ and $N_\mathrm{f} = 2$ averages presented in Eqs. (110), (111) and (112) are for the average of the $B^+$- and $B^0$-meson decay constant, $f_B$, and the corresponding ratio, $f_{B_{s}}/f_B$. Given the errors quoted in the results that enter our averages, we currently include the FNAL/MILC 11 results for the $B^+$-meson in Eq. (111). As the precision with which $B$-meson decay constants are obtained continues to improve, especially given the availability of physical-mass ensembles, future reviews will need to distinguish between these cases. Indeed HPQCD 13 finds a 2 % difference between the $B^+$ and $B^0$ decay constants, which is the same size as the total uncertainty in this calculation. We strongly recommend that future lattice-QCD calculations of $B$-meson decay constants quote results for the $B^+$- and $B^0$-mesons separately.

### Neutral $B$-meson mixing matrix elements

Neutral $B$-meson mixing is induced in the Standard Model through one-loop box diagrams to lowest order in the electroweak theory, similar to those for neutral kaon mixing. The effective Hamiltonian is given by

113 $$\begin{aligned} \mathcal{H}_\mathrm{eff}^{\Delta B = 2, \mathrm{SM}} = \frac{G_F^2 M_\mathrm{{W}}^2}{16\pi ^2} \left( \mathcal{F}^0_{d} \mathcal{Q}^d_1 + \mathcal{F}^0_{s} \mathcal{Q}^s_1\right) + \hbox {h.c.}, \end{aligned}$$

with

114 $$\begin{aligned} \mathcal{Q}^q_1 = \left[ \bar{b}\gamma _\mu (1-\gamma _5)q\right] \left[ \bar{b}\gamma _\mu (1-\gamma _5)q\right] , \end{aligned}$$

where $q\!=\!d$ or $s$. The short-distance function $\mathcal{F}^0_{q}$ in Eq. (113) is much simpler compared to the kaon mixing case due to the hierarchy in the CKM matrix elements. Here, only one term is relevant,

115 $$\begin{aligned} \mathcal{F}^0_{q} = \lambda _{tq}^2 S_0(x_{t}) \end{aligned}$$

where

116 $$\begin{aligned} \lambda _{tq} = V^*_{tq}V_{tb}, \end{aligned}$$

and where $S_0(x_{t})$ is an Inami–Lim function with $x_{t}=m_{t}^2/M_W^2$, which describes the basic electroweak loop contributions without QCD [294]. The transition amplitude for $B_{q}^0$ with $q=d$ or $s$ can be written as

117 $$\begin{aligned}&{\langle }\bar{B}^0_{q} \vert \mathcal{H}_\mathrm{eff}^{\Delta B = 2} \vert B^0_{q}{\rangle } = \frac{G_F^2 M_\mathrm{{W}}^2}{16 \pi ^2} \left[ \lambda _{tq}^2 S_0(x_{t}) \eta _{2B}\right] \nonumber \\&\quad \times \left( \frac{\bar{g}(\mu )^2}{4\pi }\right) ^{-\gamma _0/(2\beta _0)}\exp \left\{ \int _0^{\bar{g}(\mu )} \, \hbox {d}g \, \left( \frac{\gamma (g)}{\beta (g)} + \frac{\gamma _0}{\beta _0g} \right) \right\} \nonumber \\&\quad \times {\langle }\bar{B}^0_{q} \vert Q^q_{R} (\mu ) \vert B^0_{q}{\rangle } + \mathrm{h.c.}, \end{aligned}$$

where $Q^q_{R} (\mu )$ is the renormalised four-fermion operator (usually in the NDR scheme of ${\overline{\mathrm{MS}}}$). The running coupling ($\bar{g}$), the $\beta$-function ($\beta (g)$), and the anomalous dimension of the four-quark operator ($\gamma (g)$) are defined in Eqs. (79) and (80). The product of $\mu$-dependent terms on the second line of Eq. (117) is, of course, $\mu$ independent (up to truncation errors if perturbation theory is used). The explicit expression for the short-distance QCD correction factor $\eta _{2B}$ (calculated to NLO) can be found in Ref. [292].

For historical reasons the $B$-meson mixing matrix elements are often parameterised in terms of bag parameters defined as

118 $$\begin{aligned} B_{B_{q}}(\mu )= \frac{{\left\langle \bar{B}^0_{q}\left| Q^q_{R}(\mu )\right| B^0_{q}\right\rangle } }{ {\frac{8}{3}f_{B_{q}}^2m_{\mathrm {B}}^2}}. \end{aligned}$$

The RGI $B$ parameter $\hat{B}$ is defined, as in the case of the kaon, and expressed to two-loop order as

119 $$\begin{aligned}&\hat{B}_{B_{q}} =\left( \frac{\bar{g}(\mu )^2}{4\pi }\right) ^{- \gamma _0/(2\beta _0)}\,\nonumber \\&\quad \times \left\{ 1+\dfrac{\bar{g}(\mu )^2}{(4\pi )^2}\left[ \frac{\beta _1\gamma _0-\beta _0\gamma _1}{2\beta _0^2}\right] \right\} \,B_{B_{q}}(\mu ), \end{aligned}$$

with $\beta _0$, $\beta _1$, $\gamma _0$ and $\gamma _1$ defined in Eq. (81).

Non-zero transition amplitudes result in a mass difference between the $CP$ eigenstates of the neutral $B$-meson system. Writing the mass difference for a $B_{q}^0$-meson as $\Delta m_{q}$, its Standard Model prediction is

120 $$\begin{aligned} \Delta m_{q} = \frac{G^2_Fm^2_W m_{B_{q}}}{6\pi ^2} \, |\lambda _{tq}|^2 S_0(x_{t}) \eta _{2B} f_{B_{q}}^2 \hat{B}_{B_{q}}. \end{aligned}$$

Experimentally the mass difference is measured as oscillation frequency of the $CP$ eigenstates. The frequencies are measured precisely with an error of less than a percent. Many different experiments have measured $\Delta m_{d}$, but the current average [74] is dominated by measurements from the $B$-factory experiments Belle and Babar, and from the LHC experiment LHC$b$. For $\Delta m_{s}$ the experimental average is based on results from the Tevatron experiment CDF and from the LHC experiment LHC$b$ [74]. With these experimental results and lattice-QCD calculations of $f_{B_{q}}^2\hat{B}_{B_{q}}$ at hand, $\lambda _{tq}$ can be determined. In lattice-QCD calculations the flavour SU(3)-breaking ratio

121 $$\begin{aligned} \xi ^2 = \frac{f_{B_{s}}^2B_{B_{s}}}{f_{B_{d}}^2B_{B_{d}}} \end{aligned}$$

can be obtained more precisely than the individual $B_{q}$-mixing matrix elements because statistical and systematic errors cancel in part. With this ratio $|V_{td}/V_{ts}|$ can be determined, which can be used to constrain the apex of the CKM triangle.

Neutral $B$-meson mixing, being loop-induced in the Standard Model is also a sensitive probe of new physics. The most general $\Delta B=2$ effective Hamiltonian that describes contributions to $B$-meson mixing in the Standard Model and beyond is given in terms of five local four-fermion operators:

122 $$\begin{aligned} \mathcal{H}_\mathrm{eff}^{\Delta B = 2} = \sum _{i=1}^5 \mathcal{C}_i \mathcal{Q}_i , \end{aligned}$$

where $\mathcal{Q}_1$ is defined in Eq. (114) and where

123 $$\begin{aligned} \mathcal{Q}^q_2&= \left[ \bar{b}(1-\gamma _5)q\right] \left[ \bar{b}(1-\gamma _5)q\right] ,\nonumber \\ \mathcal{Q}^q_3&= \left[ \bar{b}^{\alpha }(1-\gamma _5)q^{\beta }\right] \left[ \bar{b}^{\beta }(1-\gamma _5)q^{\alpha }\right] ,\nonumber \\ \mathcal{Q}^q_4&= \left[ \bar{b}(1-\gamma _5)q\right] \left[ \bar{b}(1+\gamma _5)q\right] ,\nonumber \\ \mathcal{Q}^q_5&= \left[ \bar{b}^{\alpha }(1-\gamma _5)q^{\beta }\right] \left[ \bar{b}^{\beta }(1+\gamma _5)q^{\alpha }\right] , \end{aligned}$$

with the superscripts $\alpha ,\beta$ denoting colour indices, which are shown only when they are contracted across the two bilinears. The short-distance Wilson coefficients $\mathcal{C}_i$ depend on the underlying theory and can be calculated perturbatively. In the Standard Model only matrix elements of $\mathcal{Q}^q_1$ contribute to $\Delta m_{q}$, and combinations of matrix elements of $\mathcal{Q}^q_1$, $\mathcal{Q}^q_2$ and $\mathcal{Q}^q_3$ contribute to the width difference $\Delta \Gamma _{q}$ [410, 411]. Matrix elements of $\mathcal{Q}^q_4$ and $\mathcal{Q}^q_5$ are needed for calculating the contributions to $B_{q}$-meson mixing from beyond the Standard Model theories.

**Fig. 17:**
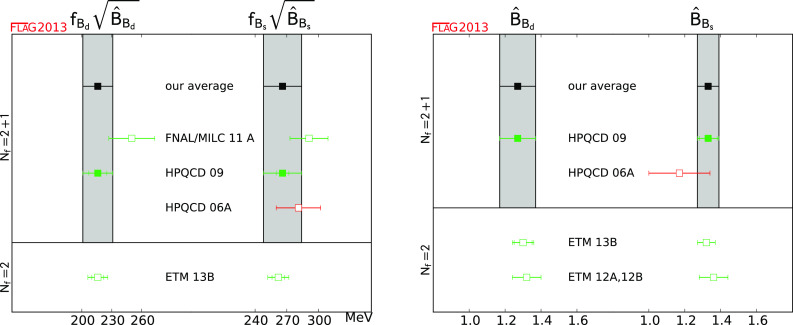
Neutral $B$- and $B_{s}$-meson mixing matrix elements and bag parameters [values in Table 26 and Eqs. (124), (125)]

**Fig. 18:**
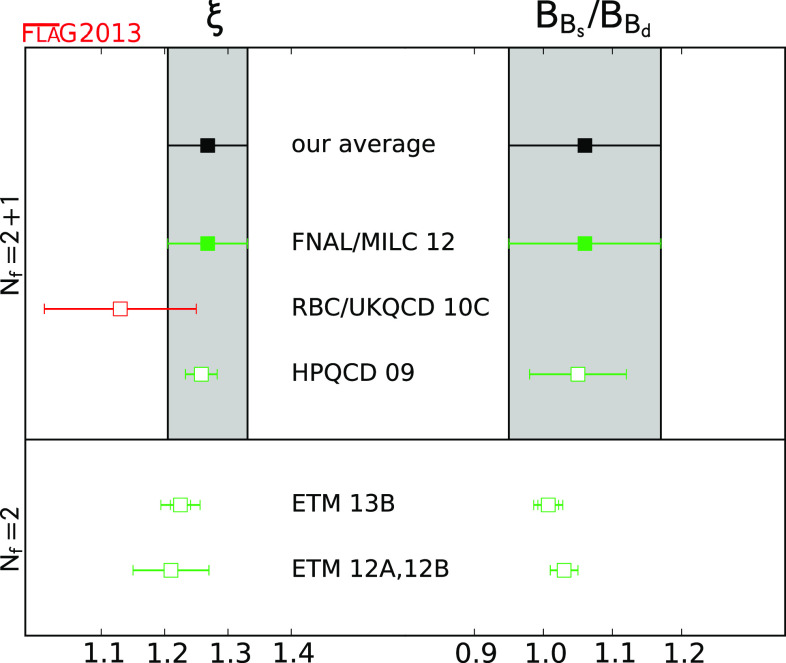
The SU(3)-breaking quantities $\xi$ and $B_{B_{s}}/B_{B_{d}}$ [values in Table 27 and Eq. (126)]

In this section we report on results from lattice-QCD calculations for the neutral $B$-meson mixing parameters $\hat{B}_{B_{d}}$, $\hat{B}_{B_{s}}$, $f_{B_{d}}\sqrt{\hat{B}_{B_{d}}}$, $f_{B_{s}}\sqrt{\hat{B}_{B_{s}}}$ and the SU(3)-breaking ratios $B_{B_{s}}/B_{B_{d}}$ and $\xi$ defined in Eqs. (118), (119), and (121). The results are summarised in Tables 26 and 27 and in Figs. 17 and 18. Additional details as regards the underlying simulations and systematic error estimates are given in Appendix B.6.2. Some collaborations do not provide the RGI quantities $\hat{B}_{B_{q}}$ but quote instead $B_B(\mu )^{\overline{\mathrm{MS}},\mathrm{NDR}}$. In such cases we convert the results to the RGI quantities quoted in Table 26 using Eq. (119). More details on the conversion factors are provided below in the descriptions of the individual results. One group also reports results for $B$-meson matrix elements of the other operators $\mathcal{Q}_{2\hbox {--}5}$ in Ref. [412], which is a conference proceedings.

**Table 26:** Neutral $B$- and $B_{s}$-meson mixing matrix elements (in MeV) and bag parameters

Collaboration	Ref.	$N_\mathrm{f}$	Publication status	Continuum Extrapolation	Chiral extrapolation	Finite volume	Renormalisation/ matching	Heavy quark treatment	$f_{B_{d}}\sqrt{\hat{B}_{B_{d}}}$	$f_{B_{d}}\sqrt{\hat{B}_{B_{s}}}$	$\hat{B}_{B_{d}}$	$\hat{B}_{B_{s}}$
FNAL/MILC 11A	[412]	$2+1$	C						250 (23)$^\dagger$	291 (18)$^\dagger$	–	–
HPQCD 09	[403]	$2+1$	A		$^\nabla$				216 (15)$^*$	266 (18)$^*$	1.27 (10)$^*$	1.33 (6)$^*$
HPQCD 06A	[413]	$2+1$	A						–	281 (21)	–	1.17 (17)
ETM 13B	[335]	2	P						216 (6) (8)	262 (6) (8)	1.30 (5) (3)	1.32 (5) (2)
ETM 12A, 12B	[393, 414]	2	C						–	–	1.32 (8)$^\diamond$	1.36 (8)$^\diamond$

$^\dagger$ Reported $f_B^2B$ at $\mu =m_{b}$ is converted to RGI by multiplying the two-loop factor 1.517

$^\nabla$ Wrong-spin contributions are not included in the rS$\chi$PT fits

$^*$ This result uses an old determination of $r_1=0.321(5)$ fm from Ref. [380] that has since been superseded

$^\diamond$ Reported $B$ at $\mu =m_{b}=4.35$ GeV is converted to RGI by multiplying the two-loop factor 1.521

**Table 27:** Results for SU(3)-breaking ratios of neutral $B_{d}$- and $B_{s}$-meson mixing matrix elements and bag parameters

Collaboration	Ref.	$N_\mathrm{f}$	Publication status	Continuum extrapolation	Chiral extrapolation	Finite volume	Renormalisation/ matching	Heavy quark treatment	$\xi$	$B_{B_{s}}/B_{B_{d}}$
FNAL/MILC 12	[415]	$2+1$	A						1.268 (63)	1.06 (11)
RBC/UKQCD 10C	[406]	$2+1$	A						1.13 (12)	–
HPQCD 09	[403]	$2+1$	A		$^\nabla$				1.258 (33)	1.05 (7)
ETM 13B	[335]	2	P						1.225 (16) (14) (22)	1.007 (15) (14)
ETM 12A, 12B	[393, 414]	2	C						1.21 (6)	1.03 (2)

$^\nabla$ Wrong-spin contributions are not included in the rS$\chi$PT fits

The ETM collaboration has presented their first results for $B$-mixing quantities with $N_\mathrm{f}=2$ sea quarks in Refs. [393, 414] (ETM 12A, 12B) using ensembles at three lattice spacings in the range $a \approx 0.065$–0.098 fm with a minimum pion mass of $270$ MeV. Additional ensembles at $a \approx 0.054 \,\mathrm{fm}$ are included in ETM 13B [335]. The valence and sea quarks are simulated with two different versions of the twisted-mass Wilson fermion action. The heavy-quark masses are in the charm region and above while keeping $am_{h} \lesssim 0.6$ for ETM 12A and 12B. Larger masses up to $am_{h} \lesssim 0.85$ are used for ETM 13B. In this series of calculations the ratio method first developed for $B$-meson decay constants (see Appendix A.1.3 and Sect. 8.1) is extended to $B$-meson mixing quantities. ETM again constructs ratios of $B$-mixing matrix elements (now called $\omega _{d(s)}$) that are equal to unity in the static limit, including also an analogous ratio for $\xi$. The renormalisation of the four-quark operator is calculated non-perturbatively in the RI’/MOM scheme. As an intermediate step for the interpolation to the physical $b$-quark mass, these ratios include perturbative matching factors to match the four-quark operator from QCD to HQET; these include tree-level and leading-log contributions in ETM 12A and 12B, and additionally next-to-leading-log contributions in ETM 13B. Similar to their decay-constant analysis, ETM analyses the SU(3) breaking ratio of ratios, $\omega _{s}/\omega _{\ell }$, and combines it with $\omega _{s}$ to obtain $B_{B_{d}}$. The data are interpolated to a fixed set of heavy-quark reference masses on all ensembles, and subsequently extrapolated to the continuum and to the physical light-quark masses in a combined fit. The interpolation to the physical $b$-quark mass is linear or quadratic in the inverse of the heavy-quark mass. While ETM 13B reports RGI bag parameters, ETM 12A and 12B report only $B_B(m_{b})^{\overline{\mathrm{MS}},\mathrm{NDR}}$ at $m_{b}=4.35$ GeV. Taking $\alpha _\mathrm{s}(M_Z)=0.1184$ [97], we apply an RGI conversion factor of $\hat{B}_B/B_B(m_{b})^{\overline{\mathrm{MS}},\mathrm{NDR}}=1.521$ to obtain the $\hat{B}_B$ values quoted in Table 26. The observed discretisation effects (as measured by the percentage difference between the lattice data at the smallest lattice spacing and the continuum-extrapolated results) are $\lesssim$1 % over the range of heavy-quark masses used in their simulations. As a result, the dominant error on the bag parameters and on the ratio of bag parameters is the combined statistical uncertainty, whereas the dominant error on the SU(3)-breaking ratio $\xi$ is due to the chiral extrapolation. Because these studies appear either in conference proceedings or preprint only, the results do not enter our averages.

For the $N_\mathrm{f}=2+1$ case there are three collaborations that have presented results for $B-\bar{B}$ mixing matrix elements: HPQCD, RBC/UKQCD, and FNAL/MILC. The first published results are by the HPQCD collaboration [403, 413] and use NRQCD $b$ quarks and Asqtad light valence quarks on $N_\mathrm{f}=2+1$ MILC Asqtad ensembles. In HPQCD 06A [413] results are presented for $B_{s}$-mixing quantities only, using one lattice spacing and two light sea-quark masses with a minimum RMS pion mass of 510 MeV. The observed sea-quark mass dependence is much smaller than the rather large statistical errors. This calculation uses one-loop mean-field improved lattice perturbation theory for the operator renormalisation. Discretisation errors cannot be estimated from the data with only one lattice spacing, but they are estimated using power counting arguments to be smaller than the dominant statistical and renormalisation errors. With only one lattice spacing and given the rather large minimum RMS pion mass, this result does not enter our averages. These shortcomings are removed in HPQCD 09 [403] with two lattice spacings, ($a \approx 0.09, 0.12$ fm) and four or two sea-quark masses per lattice spacing with a minimum RMS pion mass of about 400 MeV. The calculation is also extended to include both $B_{d}$ and $B_{s}$ mixing quantities and thus also the SU(3)-breaking ratios. A combined chiral and continuum extrapolation of the data is performed, using NLO HMrS$\chi$PT, supplemented by NNLO analytic and generic discretisation terms of $\mathcal{O}(\alpha _\mathrm{s} a^2, a^4)$. The dominant systematic error is due to using one-loop mean-field improved lattice perturbation theory for the operator renormalisation and matching, the same as in HPQCD 06. It is estimated as 4 and 2.5 %, respectively, consistent with power counting. The statistical, chiral, and continuum-extrapolation uncertainties are also prominent sources of uncertainty, followed by heavy-quark truncation and scale-setting errors. The dominant error on $\xi$ is due to statistics and chiral extrapolation. Finally, we note that this work uses an old determination of $r_1=0.321(5)$ fm from Ref. [380] to set the scale, which has since been superseded, and that differs from the new value by about two standard deviations. Dimensionless quantities are, of course, affected by a change in $r_1$ only through the inputs, which are a subdominant source of uncertainty. The scale uncertainty itself is also subdominant in the error budget, and this change therefore does not affect HPQCD 09’s results for $f_{B_{q}}\sqrt{B_{B_{q}}}$ outside of the total error.

The RBC/UKQCD collaboration has presented a result for the SU(3) breaking ratio $\xi$ in Ref. [406] using a static-limit action on $N_\mathrm{f}=2+1$ domain-wall ensembles at a single lattice spacing $a \approx 0.11$ fm with a minimum pion mass of approximately 430 MeV. They use both HYP and APE smearing for the static-limit action and one-loop mean-field improved lattice perturbation theory to renormalise the static-limit four-quark operators. Effects of $\mathcal{O}(1/m_{h})$ are not included in the static-limit action and operators, but Ref. [406] includes an estimate of this effect via power counting as $\mathcal{O}((m_{s}-m_{d})/m_{b})$ in the error budget. The statistical errors in this work are significant ($\sim$5–6 %), as are the chiral-extrapolation errors ($\sim$7 %, estimated from the difference between fits using NLO SU(2) HM$\chi$PT and a linear fit function), due to the rather large pion masses used in this in this work. With data at only one lattice spacing, discretisation errors cannot be estimated from the data, but a power counting estimate of this error of 4 % is included in the systematic error budget. With only one lattice spacing this result does not enter our averages. The RBC/UKQCD collaboration reported at Lattice 2013 [408] that they are extending this study, using HYP and HYP2 smearings for the static-limit action, smaller pion masses, larger volumes and two lattice spacings. The conference proceedings [408], however, did not appear until after the closing deadline and is therefore not included in this review.

Another calculation of the SU(3)-breaking ratio $\xi$ is presented by the Fermilab Lattice and MILC collaborations in Ref. [415] (FNAL/MILC 12). The calculation uses the Fermilab method for the $b$ quarks together with Asqtad light and strange valence quarks on a subset of the MILC Asqtad $N_\mathrm{f}=2+1$ ensembles, including lattice spacings in the range $a \approx 0.09$–0.12 fm and a minimum RMS pion mass of approximately 320 MeV. This analysis includes partially quenched lattice data at six valence light-quark masses for each sea-quark ensemble. The operator renormalisations are calculated using one-loop mean-field improved lattice perturbation theory, which does not result in a significant source of uncertainty for the SU(3)-breaking ratios. The combined chiral and continuum extrapolations use a chiral fit function based on NLO partially quenched HMrS$\chi$PT supplemented by NNLO analytic terms. Also included are light-quark discretisation terms of $\mathcal{O}(\alpha _\mathrm{s}^2 a^2, a^4)$. The combined statistical, light-quark discretisation, and chiral-extrapolation error dominates the error budget together with an uncertainty that is described as the error due to the omission of “wrong-spin contributions” (see below). First results for the $B$ mixing matrix elements from an ongoing FNAL/MILC calculation of all $B$-meson mixing quantities on the full set of Asqtad ensembles are presented in [412], including the matrix elements of all five operators that contribute to $B$-meson mixing in the Standard Model and beyond. The dominant uncertainties on the matrix elements are due to the combined statistical, chiral extrapolation, and light-quark discretisation error and due to the one-loop matching. FNAL/MILC 11A reports results for $f_{B_{q}}\sqrt{B_{B_{q}}}$ evaluated at $\mu =m_{b}$ in the $\overline{\mathrm{MS}}$ NDR scheme. Taking $\alpha _\mathrm{s}(M_Z)=0.1184$ [97] and $m_{b} = 4.19$ GeV [74], we apply an RGI conversion factor of $\hat{B}_B/B_B(m_{b})^{\overline{\mathrm{MS}},\mathrm{NDR}}=1.517$ to obtain the values for the RGI quantities listed in Table 26. Reference [412] presents a complete error budget, but since the paper is a conference proceedings, its results are not included in our averages.

For the $N_\mathrm{f}=2$ case there are no published results, so we do not quote an average for this case. For $N_\mathrm{f}=2+1$ only the results of HPQCD 09 and FNAL/MILC 12 enter our averages. First, we must consider the issue of the so-called “wrong-spin contributions,” described in Ref. [415] and explained in detail in Ref. [416]. With staggered light quarks, interactions between different unphysical species (“tastes”) of quarks induce mixing between the operator ${\mathcal Q}_1^q$ in Eq. (114) and the operators ${\mathcal Q}_2^q$ and ${\mathcal Q}_3^q$ in Eq. (123) at ${\mathcal O}(a^2)$. These additional contributions to the matrix element $f_{B_{q}} \sqrt{B_{B_{q}}}$ are discretisation errors that vanish in the continuum limit. The contributions of ${\mathcal Q}_1^q$–${\mathcal Q}_5^q$ have been derived at next-to-leading order in HMrS$\chi$PT [416]. The result is that, in the chiral expansion of the matrix elements of ${\mathcal Q}_1^q$, the matrix elements of ${\mathcal Q}_{2,3}^q$ appear with ${\mathcal O}(a^2)$ coefficients that depend upon the light-quark masses. These contributions can be accounted for in the chiral-continuum extrapolation by fitting the numerical results for the matrix elements of the three operators simultaneously. Further, if the matrix elements of all five basis operators in Eqs. (114) and (123) are computed on the lattice, then no additional low-energy constants are required to describe the wrong-spin contributions effects in the chiral–continuum extrapolation. In principle, instead of using HMrS$\chi$PT as described above, it is possible to account for the wrong-spin terms via the inclusion of generic mass-dependent terms such as $O(a^2 m^2_{\pi })$ in the combined chiral–continuum extrapolation, provided that the lattice spacing and light-quark masses are small enough.

Both HPQCD 09 and FNAL/MILC 11A use chiral fit functions based on NLO HMrS$\chi$PT. Since, however, these works predate Refs. [415, 416], the wrong-spin terms are not included in their chiral extrapolations. The calculation in FNAL/MILC 12 also does not include the matrix elements of all three operators, so here the effect of the wrong-spin contributions is treated as a systematic error, which is estimated using the lattice data described in Ref. [412]. As discussed above, the estimated uncertainty of 3 % for $\xi$ is a dominant contribution to the error budget in Ref. [415]. Because, however, HPQCD 09 does not include the wrong-spin contributions in its chiral extrapolations, we must consider how they affect the results. First, the chiral fit functions used in HPQCD 09 and in FNAL/MILC 12 are very similar with similar (though not identical) choices for prior widths. The main difference is that the generic light-quark discretisation term of $\mathcal{O}(\alpha _\mathrm{s} a^2)$ included in HPQCD 09 is a little less constrained than the $\mathcal{O}(\alpha _\mathrm{s}^2 a^2)$ term included in FNAL/MILC 12. It is therefore possible that the chiral extrapolation in HPQCD 09 accounts for the wrong-spin contributions via the generic discretisation terms. Furthermore, for $f_{B_{q}}\sqrt{B_{B_{q}}}$ the chiral-extrapolation error, while not insignificant, is not a dominant source of error in the HPQCD calculation. For $\xi$, however, the chiral-extrapolation error is a dominant source of uncertainty, and the FNAL/MILC 12 analysis indicates that the omission of the wrong-spin contributions from HMrS$\chi$PT fits may also be a significant source of error. We therefore make the conservative choice of excluding HPQCD 09’s result for $\xi$ from our average, but keeping HPQCD 09’s results for $f_{B_{q}}\sqrt{B_{B_{q}}}$ and $B_{B_{q}}$ in our averages. As a result, we now have only one calculation that enters our averages for each quantity. Our averages are ($N_\mathrm{f}=2+1$):

124 $$\begin{aligned}&f_{B_{d}}\sqrt{\hat{B}_{B_{d}}} = 216(15)\,\mathrm{MeV},\quad f_{B_{s}}\sqrt{\hat{B}_{B_{s}}} = 266(18)\,\mathrm{MeV}\nonumber \\ \end{aligned}$$

125 $$\begin{aligned}&\hat{B}_{B_{d}} = 1.27(10), \quad \hat{B}_{B_{s}} = 1.33(6), \end{aligned}$$

126 $$\begin{aligned}&\xi = 1.268(63), \quad B_{B_{s}}/B_{B_{d}} = 1.06(11). \end{aligned}$$

Finally, we note that the above results are all correlated with each other: the numbers in (124) and (125) are from HPQCD 09 [403], while those in (126) are from FNAL/MILC 12 [415]—the same Asqtad MILC ensembles are used in these simulations. The results are also correlated with the averages obtained in Sect. 8.1 and shown in Eq. (111), because the calculations of $B$-meson decay constants and mixing quantities are performed on the same (or on similar) sets of ensembles, and results obtained by a given collaboration use the same actions and setups. These correlations must be considered when using our averages as inputs to UT fits. In the future, as more independent calculations enter the averages, correlations between the lattice-QCD inputs to the UT fit will become less significant.

### Semileptonic form factors for $B$ decays to light flavours

The Standard Model differential rate for the decay $B_{(s)}\rightarrow P\ell \nu$ involving a quark-level $b\rightarrow u$ transition is given, at leading order in the weak interaction, by a formula identical to the one for $D$ decays in Eq. (95) but with $D \rightarrow B_{(s)}$ and the relevant CKM matrix element $|V_{cq}| \rightarrow |V_{ub}|$:

127 $$\begin{aligned}&\frac{d\Gamma (B_{(s)}\rightarrow P\ell \nu )}{dq^2} = \frac{G_F^2 |V_{ub}|^2}{24 \pi ^3} \,\frac{(q^2-m_\ell ^2)^2\sqrt{E_P^2-m_P^2}}{q^4m_{B_{(s)}}^2}\nonumber \\&\quad \times \left[ \left( 1+\frac{m_\ell ^2}{2q^2}\right) m_{B_{(s)}}^2(E_P^2-m_P^2)|f_+(q^2)|^2\right. \nonumber \\&\quad \left. +\,\frac{3m_\ell ^2}{8q^2}(m_{B_{(s)}}^2-m_P^2)^2|f_0(q^2)|^2 \right] . \end{aligned}$$

Again, for $\ell =e,\mu$ the contribution from the scalar form factor $f_0$ can be neglected, and one has a similar expression to Eq. (97), which in principle allows for a direct extraction of $|V_{ub}|$ by matching theoretical predictions to experimental data. However, while for $D$ (or $K$) decays the entire physical range $0 \le q^2 \le q^2_\mathrm{max}$ can be covered with moderate momenta accessible to lattice simulations, in $B \rightarrow \pi \ell \nu$ decays one has $q^2_\mathrm{max} \sim 26~\mathrm{GeV}^2$ and only part of the full kinematic range is reachable. As a consequence, obtaining $|V_{ub}|$ from $B\rightarrow \pi \ell \nu$ is more complicated then obtaining $|V_{cd(s)}|$ from semileptonic $D$-meson decays. The standard procedure involves the matching of theoretical predictions and experimental data for the integrated decay rate over a limited $q^2$ range,

128 $$\begin{aligned} \Delta \zeta = \frac{1}{|V_{ub}|^2} \int _{q^2_{1}}^{q^2_{2}} \left( \frac{\hbox {d} \Gamma }{\hbox {d} q^2} \right) \,\hbox {d}q^2. \end{aligned}$$

This requires knowledge of the relevant form factor(s) within the integration interval. In practice, lattice computations are restricted to small values of the momentum transfer (see Sect. 7.2) where statistical and momentum-dependent discretisation errors can be controlled,^32^ which in existing calculations roughly cover the upper third of the kinematically allowed $q^2$ range. Experimental results normally cover the whole interval, but they are more precise in the low-$q^2$ region. Therefore, both experimental and lattice data for the $q^2$ dependence have to be parameterised by fitting data to a specific ansatz, either separately or jointly (with the relative normalisation $|V_{ub}|^2$ as a free parameter). A good control of the systematic uncertainty induced by the choice of parameterisation is hence crucial to obtain a precise determination of $|V_{ub}|$.

#### Parameterisations of heavy-to-light semileptonic form factors

All form factors are analytic functions of $q^2$ outside physical poles and inelastic threshold branch points; in the case of $B\rightarrow \pi \ell \nu$, the only pole expected below the $B\pi$ production region, starting at $q^2 = t_+ = (m_B+m_\pi )^2$, is the $B^*$. A simple ansatz for the $q^2$ dependence of the $B\rightarrow \pi \ell \nu$ semileptonic form factors that incorporates vector-meson dominance is the Bećirević–Kaidalov (BK) parameterisation [356]:

129 $$\begin{aligned} f_+(q^2)&= \frac{f(0)}{(1-q^2/m_{B^*}^2)(1-\alpha q^2/m_{B^*}^2)}, \nonumber \\ f_0(q^2)&= \frac{f(0)}{1-\frac{1}{\beta }\,q^2/m_{B^*}^2}. \end{aligned}$$

Because the BK ansatz has few free parameters, it has been used extensively to parameterise the shape of experimental branching-fraction measurements and theoretical form-factor calculations. A variant of this parameterisation proposed by Ball and Zwicky (BZ) adds extra pole factors to the expressions in Eq. (129) in order to mimic the effect of multiparticle states [418]. Another variant (RH) has been proposed by Hill in [419]. Although all of these parameterisations capture some known properties of form factors, they do not manifestly satisfy others. For example, perturbative QCD scaling constrains the high-$q^2$ behaviour to be $f_+(q^2)\sim 1/q^2$ up to logarithmic corrections [420–422], and angular momentum conservation constrains the asymptotic behaviour near thresholds—e.g. $\mathrm{Im}\,f_+(q^2) \sim (q^2-t_+)^{3/2}$ (see e.g. [350]). Further, they do not allow for an easy quantification of systematic uncertainties.

A more systematic approach that improves upon the use of simple models for the $q^2$ behaviour exploits the positivity and analyticity properties of two-point functions of vector currents to obtain optimal parameterisations of form factors [349, 422–426]. Any form factor $f$ can be shown to admit a series expansion of the form

130 $$\begin{aligned} f(q^2) = \frac{1}{B(q^2)\phi (q^2,t_0)}\,\sum _{n=0}^\infty a_n(t_0)\,z(q^2,t_0)^n, \end{aligned}$$

where the squared momentum transfer is replaced by the variable

131 $$\begin{aligned} z(q^2,t_0) = \frac{\sqrt{t_+-q^2}-\sqrt{t_+-t_0}}{\sqrt{t_+-q^2}+\sqrt{t_+-t_0}}. \end{aligned}$$

This is a conformal transformation, depending on an arbitrary real parameter $t_0<t_+$, that maps the $q^2$ plane cut for $q^2 \ge t_+$ onto the disk $|z(q^2,t_0)|<1$ in the $z$ complex plane. The function $B(q^2)$ is called the *Blaschke factor*, and contains poles and cuts below $t_+$—for instance, in the case of $B\rightarrow \pi$ decays

132 $$\begin{aligned} B(q^2)=\frac{z(q^2,t_0)-z(m_{B^*}^2,t_0)}{1-z(q^2,t_0)z(m_{B^*}^2,t_0)}=z(q^2,m_{B^*}^2). \end{aligned}$$

Finally, the quantity $\phi (q^2,t_0)$, called the *outer function*, is an analytic function that does not introduce further poles or branch cuts. The crucial property of this series expansion is that the sum of the squares of the coefficients

133 $$\begin{aligned} \sum _{n=0}^\infty a_n^2 = \frac{1}{2\pi i}\oint \frac{\hbox {d}z}{z}\,|B(z)\phi (z)f(z)|^2, \end{aligned}$$

is a finite quantity. Therefore, by using this parameterisation an absolute bound to the uncertainty induced by truncating the series can be obtained. The criteria involved in the optimal choice of $\phi$ then aim at obtaining a bound that is useful in practice, while (ideally) preserving the correct behaviour of the form factor at high $q^2$ and around thresholds.

The simplest form of the bound would correspond to $\sum _{n=0}^\infty a_n^2=1$. *Imposing* this bound yields the following “standard” choice for the outer function

134 $$\begin{aligned} \phi (q^2,t_0)&= \sqrt{\frac{1}{32\pi \chi _{1^-}(0)}}\, \left( \sqrt{t_+-q^2}+\sqrt{t_+-t_0}\right) \nonumber \\&\times \left( \sqrt{t_+-q^2}+\sqrt{t_+-t_-}\right) ^{3/2}\nonumber \\&\times \left( \sqrt{t_+-q^2}+\sqrt{t_+}\right) ^{-5}\,\frac{t_+-q^2}{(t_+-t_0)^{1/4}}, \end{aligned}$$

where $\chi _{1^-}(0)$ is the derivative of the transverse component of the polarisation function (i.e. the Fourier transform of the vector two-point function) $\Pi _{\mu \nu }(q)$ at Euclidian momentum $Q^2=-q^2=0$. It is computed perturbatively, using operator product expansion techniques, by relating the $B\rightarrow \pi \ell \nu$ decay amplitude to $\ell \nu \rightarrow B\pi$ inelastic scattering via crossing symmetry and reproducing the correct value of the inclusive rate $\ell \nu \rightarrow X_{b}$. We will refer to the series parameterisation with the outer function in Eq. (134) as Boyd, Grinstein, and Lebed (BGL). The perturbative and OPE truncations imply that the bound is not strict, and one should take it as

135 $$\begin{aligned} \sum _{n=0}^N a_n^2 \lesssim 1, \end{aligned}$$

where this holds for any choice of $N$. Since the values of $|z|$ in the kinematical region of interest are well below 1 for judicious choices of $t_0$, this provides a very stringent bound on systematic uncertainties related to truncation for $N\ge 2$. On the other hand, the outer function in Eq. (134) is somewhat unwieldy and, more relevantly, spoils the correct large $q^2$ behaviour and induces an unphysical singularity at the $B\pi$ threshold.

A simpler choice of outer function has been proposed by Bourrely, Caprini and Lellouch (BCL) in [350], which leads to a parameterisation of the form

136 $$\begin{aligned} f_+(q^2)=\frac{1}{1-q^2/m_{B^*}^2}\,\sum _{n=0}^N a_n(t_0) z(q^2,t_0)^n. \end{aligned}$$

This satisfies all the basic properties of the form factor, at the price of changing the expression for the bound to

137 $$\begin{aligned} \sum _{j,k=0}^N B_{jk}(t_0)a_j(t_0)a_k(t_0) \le 1. \end{aligned}$$

The constants $B_{jk}$ can be computed and shown to be $|B_{jk}|\lesssim \mathcal {O}(10^{-2})$ for judicious choices of $t_0$; therefore, one again finds that truncating at $N\ge 2$ provides sufficiently stringent bounds for the current level of experimental and theoretical precision. It is actually possible to optimise the properties of the expansion by taking

138 $$\begin{aligned} t_0 = t_\mathrm{opt} = (m_B-m_\pi )(\sqrt{m_B}-\sqrt{m_\pi })^2, \end{aligned}$$

which for physical values of the masses results in the semileptonic domain being mapped onto the symmetric interval $|z| \lesssim 0.279$ (where this range differs slightly for the $B^{\pm }$ and $B^0$ decay channels), minimizing the maximum truncation error. If one also imposes the requirement that the asymptotic behaviour $\mathrm{Im}\,f_+(q^2) \sim (q^2-t_+)^{3/2}$ near threshold is satisfied, then the highest-order coefficient is further constrained as

139 $$\begin{aligned} a_N=-\,\frac{(-1)^N}{N}\,\sum _{n=0}^{N-1}(-1)^n\,n\,a_n. \end{aligned}$$

Substituting the above constraint on $a_N$ into Eq. (136) leads to the constrained BCL parameterisation

140 $$\begin{aligned} f_+(q^2)=\frac{1}{1-q^2/m_{B^*}^2}\,\sum _{n=0}^{N-1} a_n\left[ z^n-(-1)^{n-N}\,\frac{n}{N}\,z^N\right] ,\nonumber \\ \end{aligned}$$

which is the standard implementation of the BCL parameterisation used in the literature.

Parameterisations of the BGL and BCL kind (to which we will refer collectively as “$z$-parameterisations”) have already been adopted by the Babar and Belle collaborations to report their results, and also by the Heavy Flavour Averaging Group (HFAG). Some lattice collaborations, such as FNAL/MILC and ALPHA, have already started to report their results for form factors in this way. The emerging trend is to use the BCL parameterisation as a standard way of presenting results for the $q^2$ dependence of semileptonic form factors. Our policy will be to quote results for $z$-parameterisations when the latter are provided in the paper (including the covariance matrix of the fits); when this is not the case, but the published form factors include the full correlation matrix for values at different $q^2$, we will perform our own fit to the constrained BCL ansatz in Eq. (140); otherwise no fit will be quoted.

#### Form factors for $B\rightarrow \pi \ell \nu$ and $B_{s}\rightarrow K\ell \nu$

The semileptonic decay processes $B\rightarrow \pi \ell \nu$ and $B_{s}\rightarrow K\ell \nu$ enable determinations of the CKM matrix element $|V_{ub}|$ within the Standard Model via Eq. (127). Results for the $B\rightarrow \pi \ell \nu$ form factors have been published by the HPQCD [427] and FNAL/MILC [351] Collaborations, in both cases for $N_\mathrm{f}=2+1$ dynamical quark flavours. Work is also under way by ALPHA [428, 429] (on $N_\mathrm{f}=2$ non-perturbatively $\mathcal {O}(a)$ improved Wilson configurations), FNAL/MILC [430, 431] (updating the published analysis), HPQCD [432, 433] (with HISQ valence light quarks), and the RBC/UKQCD Collaborations [434, 435] (with $N_\mathrm{f}=2+1$ DWF). These calculations, however, are so far described only in conference proceedings which do not provide quotable results, so they will not be discussed in this report. No unquenched computation of $B_{s}\rightarrow K\ell \nu$ form factors is currently available. Preliminary results by the HPQCD Collaboration are reported in [432, 433], while work in progress by the FNAL/MILC Collaboration is discussed in [430, 436].

Both the HPQCD and the FNAL/MILC computations of the $B\rightarrow \pi \ell \nu$ amplitudes use ensembles of gauge configurations with $N_\mathrm{f}=2+1$ flavours of rooted staggered quarks produced by the MILC Collaboration at two different values of the lattice spacing ($a\sim 0.12,~0.09$ fm). The relative scale is fixed in both cases through $r_1/a$, while the absolute scale is set through the $\Upsilon \,2S$–$1S$ splitting for HPQCD and $f_\pi$ (with uncertainty estimated from the same $\Upsilon$ splitting) for FNAL/MILC. The spatial extent of the lattices is $L\simeq 2.4$ fm, save for the lightest mass point ($a\sim 0.09$ fm) for which $L\simeq 2.9$ fm. The lightest RMS pion mass is around 400 MeV. Lattice-discretisation effects are estimated within HMrS$\chi$PT in the FNAL/MILC computation, while HPQCD quotes the results at $a\sim 0.12$ fm as central values and uses the $a\sim 0.09$ fm results to quote an uncertainty.

The main difference between the computations lies in the treatment of heavy quarks. HPQCD uses the NRQCD formalism, with a one-loop matching of the relevant currents to the ones in the relativistic theory. FNAL/MILC employs the clover action with the Fermilab interpretation, with a mostly non-perturbative renormalisation of the relevant currents, within which light–light and heavy–heavy currents are renormalised non-perturbatively and one-loop perturbation theory is used for the relative normalisation. (See Table 28; full details as regards the computations are provided in tables in Appendix B.6.3.)

**Table 28:** Results for the $B \rightarrow \pi \ell \nu$ semileptonic form factor. The quantity $\Delta \zeta$ is defined in Eq. (128); the quoted values correspond to $q_1=4$ GeV, $q_2=q_{max}$, and they are given in $\hbox {ps}^{-1}$. The “cov. matrix” entry indicates whether or not the correlations, either between the lattice form-factor data at different values of $q^2$, or between the coefficients of a $z$-parameterisation, are provided. This information is needed to use the lattice results in a combined fit to obtain $|V_{ub}|$

Collaboration	Ref.	$N_\mathrm{f}$	Publication status	Continuum extrapolation	Chiral extrapolation	Finite volume	Renormalisation	Heavy-quark treatment	$\Delta \zeta ^{B\pi }$	$z$-parameterisation		Cov. matrix
										Type	$\{ a_0, a_1, a_2\}$	
FNAL/MILC 08A	[351]	$2+1$	A						$\quad 2.21^{+0.47}_{-0.42} {}^\dagger$	BGL$^\ddagger$	$\{0.0216(27)$, $-0.038(19)$, $-0.113(27)\}$	Yes^§^
HPQCD 06	[427]	$2+1$	A						$\quad 2.07(41)(39)$	–	–	No

$^\dagger$ Value based on the calculation of Ref. [351] (private communication with the FNAL/MILC collaboration)

$^\ddagger$ Result of BGL fit to FNAL/MILC data in Ref. [351] using $\chi _{1^-}(0) = 6.88919 \times 10^{-4}$ and given in [437]

^§^ Covariance matrix $C_{ij} = \mathrm{cov}(a_i,a_j)$ given in Table IV of Ref. [437]

Chiral extrapolations are an important source of systematic uncertainty, since the pion masses at which the computations are carried out are relatively heavy. In order to control deviations from the expected $\chi$PT behaviour, FNAL/MILC supplements SU(3) HMrS$\chi$PT formulae with higher-order powers in $E_\pi$ to extend the form factor parameterisation up to $E_\pi \sim 1$ GeV. Chiral-extrapolation effects do indeed make the largest contribution to their systematic error budget. HPQCD performs chiral extrapolations using HMrS$\chi$PT formulae, and estimates systematic uncertainties by comparing the result with the ones from fits to a linear behaviour in the light-quark mass, continuum HM$\chi$PT, and partially quenched HMrS$\chi$PT formulae (including also data with different sea and valence light quark masses). This is again the dominant contribution to the error budget of the computation, along with the matching of the heavy–light current.

HPQCD provides results for both $f_+(q^2)$ and $f_0(q^2)$. In this case, the parameterisation of the $q^2$ dependence of form factors is somewhat intertwined with chiral extrapolations: a set of fiducial values $\{E_\pi ^{(n)}\}$ is fixed for each value of the light-quark mass, and $f_{+,0}$ are interpolated to each of the $E_\pi ^{(n)}$; chiral extrapolations are then performed at fixed $E_\pi$. The interpolation is performed using a BZ ansatz. The $q^2$ dependence of the resulting form factors in the chiral limit is then described by means of a BZ ansatz, which is cross-checked against BK, RH, and BGL parameterisations. FNAL/MILC presents results for $f_+(q^2)$ only, and provides as its preferred description a three-parameter fit to the BGL form in a companion paper [437]; this result is quoted in Table 28. HPQCD, on the other hand, does not provide the correlation matrix for the values of $f_{+}(q^2)$ in the chiral limit, and therefore no independent fit to a $z$-parameterisation is possible.

**Fig. 19:**
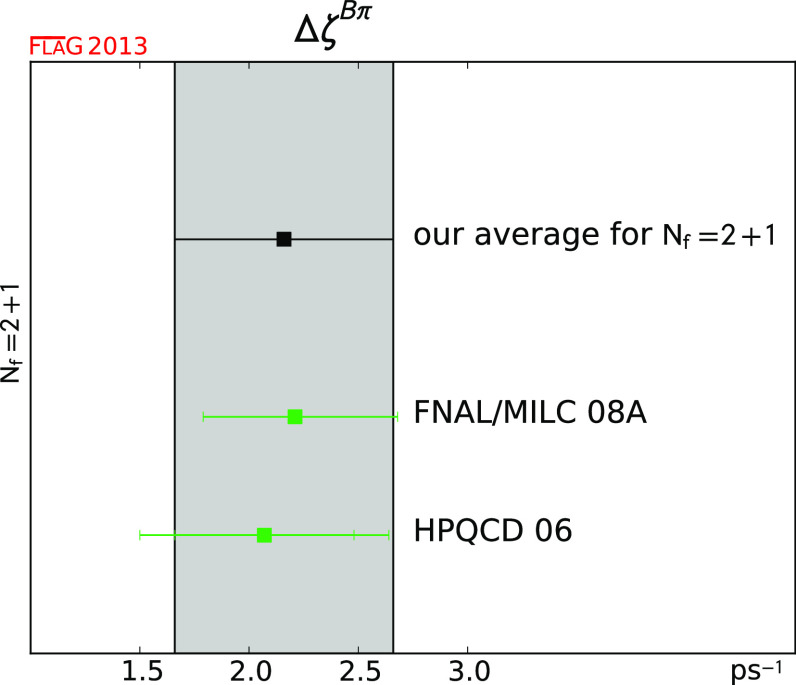
Integrated width of the decay $B\rightarrow \pi \ell \nu$ divided by $|V_{ub}|^2$ [values in Table 28 and Eq. (141)]

Results for the integrated decay rate $\Delta \zeta ^{B\pi }$, which is defined in Eq. (128) and depends on the chosen interval of integration, are available in both cases (see Table 28 and Fig. 19). We quote the average ($q_1=4\, \hbox {GeV}$, $q_2=q_\mathrm{max}$):

141 $$\begin{aligned} N_\mathrm{f}=2+1: \qquad \Delta \zeta ^{B\pi } = 2.16(50)~\mathrm{ps}^{-1}, \end{aligned}$$

where we have conservatively assumed that the calculations are 100 % correlated because neither FNAL/MILC nor HPQCD provide itemised error budgets for $\Delta \zeta ^{B\pi }$.^33^

The results for $f_+(q^2)$ in HPQCD 06 and FNAL/MILC 08A can also be combined into a single fit to our preferred BCL $z$-parameterisation, Eq. (140). While FNAL/MILC 08A provides the full correlation matrix between $f_+(q^2)$ values, this information is not available for HPQCD data; we thus perform a simultaneous fit including all the $f_+(q^2)$ values from FNAL/MILC 08A and only one point from HPQCD 06. The value of $f_+$ from HPQCD 06 that we choose to include in the fit is the one at the lowest quoted momentum transfer for which no extrapolation in the energy of the final-state pion is involved in the computation, $q^2_\mathrm{min}=17.35~\mathrm{GeV}^2$. Since in FNAL/MILC 08A $q^2_\mathrm{min}=18.4~\mathrm{GeV}^2$, this extends the covered kinematical range, and, together with the smaller relative error of the HPQCD datum, results in the latter having a significant weight in the fit. The HPQCD and FNAL/MILC computations are correlated by the use of an overlapping set of gauge-field ensembles for the evaluation of observables. We therefore treat the combined statistical plus chiral-extrapolation errors as 100 % correlated between the two calculations in the fit. We treat the other systematic uncertainties as uncorrelated because they are mostly associated with the choice of $b$-quark action, which is different in the two calculations.

**Fig. 20:**
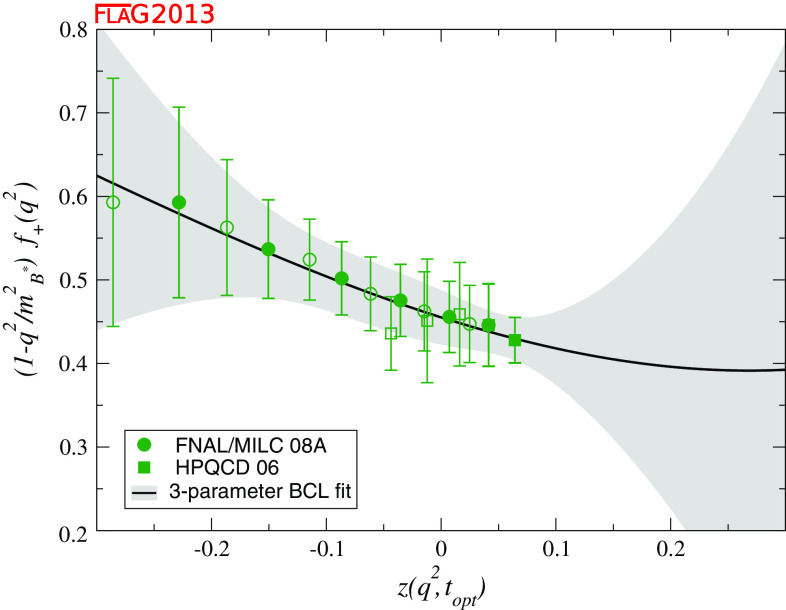
The form factors $(1 - q^2/m_{B^*}^2) f_+(q^2)$ versus $z$. The *filled symbols* denote data points included in the fit, while the *open symbols* show points that are not included in the fit (either because of unknown correlations or strong correlations). The *grey band* displays our preferred three-parameter BCL fit to the plotted data with errors

We fit the two sets of lattice data for $f_+(q^2)$ together to the BCL parameterisation in Eq. (140) and assess the systematic uncertainty due to truncating the series expansion by considering fits to different orders in $z$. Figure 20 plots the FNAL/MILC and HPQCD data points for $(1-q^2/m_{B^*}^2) f_+(q^2)$ versus $z$; the data are highly linear, and only a simple two-parameter fit is needed for a good $\chi ^2/\hbox {dof}$. (Note that a fit to the constrained BCL form in Eq. (140) with two free parameters corresponds to a polynomial through ${\mathcal O}(z^2)$, etc.) Further, we cannot constrain the coefficients of the $z$-expansion beyond this order, as evidenced by the error on the coefficient $a_2$ being significantly greater than 100 % for a three-parameter fit. Because the FNAL/MILC synthetic data points are all from the output of the same chiral–continuum extrapolation, they are strongly correlated, so inverting the full $12\times 12$ correlation matrix is problematic. We address these correlations in the FNAL/MILC data in several ways and make sure that the outcome of the fit is stable: we thin the data set to either six (every other) or four (every third) points, and imposing singular value decomposition (SVD) cuts of various severities in the construction of the pseudoinverse. The results (central values and errors) for the fit parameters are all very consistent irrespective of the treatment of correlations.

We quote as our preferred result the outcome of the three-parameter ${\mathcal O}(z^3)$ BCL fit using a thinned FNAL/MILC data set that includes every second data point starting at $q^2 = 18.4~\mathrm{GeV}^2$ in addition to the HPQCD point at $q^2=17.35~\mathrm{GeV}^2$:

142 $$\begin{aligned}&N_\mathrm{f}=2+1: \quad a_0 = 0.453(33),\quad a_1 = -0.43(33),\nonumber \\&a_2 = 0.9(3.9);\nonumber \\&\hbox {cov}(a_i,a_j)=\left( \begin{array}{l@{\quad }l@{\quad }l} 1.00 &{} -0.55 &{} -0.63 \\ -0.55 &{} 1.00 &{} 0.59 \\ -0.63 &{} 0.59 &{} 1.00 \end{array}\right) , \end{aligned}$$

where the above uncertainties encompass both the lattice errors and the systematic error due to truncating the series in $z$. This can be used as the averaged FLAG result for the lattice-computed form factor $f_+(q^2)$. The coefficient $a_3$ can be obtained from the values for $a_0$–$a_2$ using Eq. (139). We emphasise that future lattice-QCD calculations of semileptonic form factors should publish their full statistical and systematic correlation matrices to enable others to use the data fully.

#### Form factors for rare and radiative $B$ semileptonic decays to light flavours

Lattice-QCD input is also available for some exclusive semileptonic decay channels involving neutral-current $b\rightarrow s$ transitions at the quark level. Being forbidden at tree level in the SM, these processes allow for stringent tests of potential new physics; simple examples are $B\rightarrow K^*\gamma$ and $B\rightarrow K^{(*)}\ell ^+\ell ^-$, where the $B$-meson (and therefore the kaon) can be either neutral or charged.

The corresponding SM effective weak Hamiltonian is considerably more complicated than the one for the tree-level processes discussed above: after neglecting top quark effects, as many as ten dimension-six operators formed by the product of two hadronic currents or one hadronic and one leptonic current appear.^34^ Three of the latter, coming from penguin and box diagrams, dominate at short distances, and within a reasonable approximation one can keep these contributions only. Long-distance hadronic physics is then again encoded in matrix elements of current operators (vector, tensor, and axial-vector) between one-hadron states, which in turn can be parameterised in terms of a number of form factors (see [439] for a complete description). In addition, the lattice computation of the relevant form factors in channels with a vector meson in the final state faces extra challenges on top of those already present when the decay product is a Goldstone boson: the state is unstable and the extraction of the relevant matrix element from correlation functions is significantly more complicated; and $\chi$PT cannot be used as a guide to extrapolate results at unphysically heavy pion masses to the chiral limit. As a result, the current lattice methods and simulations that allow for control over systematic errors for kaon and pion final states leave uncontrolled systematic errors in calculations of weak decay form factors into unstable vector-meson final states, such as the $K^*$- or $\rho$-mesons.

Several collaborations are calculating form factors for $B \rightarrow K^{(*)}$ transitions in the Standard Model and beyond on the MILC $N_\mathrm{f}=2+1$ rooted Aqstad staggered gauge configurations. Two new results have appeared since the initial April closing date for this review. We summarise their content briefly here, but a full discussion of the calculations, including their rating, is postponed to the next major update of the FLAG review. The HPQCD Collaboration has published in Ref. [440] a determination of the three form factors for $B\rightarrow K\ell ^+\ell ^-$ with NRQCD $b$ quarks and HISQ valence light quarks. In this work, they parameterise the form factors over the full kinematic range using a model-independent $z$-expansion as in Sect. 8.3.1, and provide the series coefficients and covariance matrix. HPQCD also published a companion paper [441] in which they calculate the Standard Model predictions for the differential branching fractions and other observables and compare to experiment. Horgan et al. have obtained the seven form factors governing $B \rightarrow K^* \ell ^+ \ell ^-$ (as well as those for $B_{s} \rightarrow \phi \, \ell ^+ \ell ^-$) in Ref. [354] using NRQCD $b$ quarks and Asqtad staggered light quarks. In this work, they use a “modified” $z$-expansion to simultaneously extrapolate to the physical light-quark masses and continuum and extrapolate in $q^2$ to the full kinematic range. As discussed in Sect. 7.2, the “modified” $z$-expansion is not based on an underlying effective theory, and the associated uncertainties have yet to be fully studied. Horgan et al. use their form-factor results to calculate the differential branching fractions and angular distributions and discuss the implications for phenomenology in a companion paper [442]. Finally, the FNAL/MILC Collaboration has reported preliminary results for the three $B \rightarrow K\ell ^+\ell ^-$ form factors using Fermilab bottom quarks and Astqad light quarks in Refs. [430, 436].

### Semileptonic form factors for $B \rightarrow D \ell \nu$, $B \rightarrow D^* \ell \nu$ and $B \rightarrow D \tau \nu$

The semileptonic processes $B \rightarrow D \ell \nu$ and $B \rightarrow D^* \ell \nu$ ($\ell = e, \mu$) have been studied extensively by experimentalists and theorists over the years. They allow for the determination of the CKM matrix element $|V_{cb}|$, an extremely important parameter of the Standard Model. $|V_{cb}|$ appears in many quantities that serve as inputs into CKM Unitarity Triangle analyses and reducing its uncertainties is of paramount importance. For example, when $\epsilon _K$, the measure of indirect $CP$-violation in the neutral kaon system, is written in terms of the parameters $\rho$ and $\eta$ that specify the apex of the unitarity triangle, a factor of $|V_{cb}|^4$ multiplies the dominant term. As a result, the errors coming from $|V_{cb}|$ (and not those from $B_K$) are now the dominant uncertainty in the Standard Model (SM) prediction for this quantity. Decay rates for $B \rightarrow D^{(*)} \ell \nu$ processes can be parameterised as

143 $$\begin{aligned} \frac{\hbox {d}\Gamma _{B^-\rightarrow D^{0} \ell ^-\bar{\nu }}}{\hbox {d}w}&= \frac{G^2_\mu m^3_{D}}{48\pi ^3}(m_B+m_{D})^2(w^2-1)^{3/2}\nonumber \\&\times \,|\eta _{\mathrm {EW}}|^2|V_{cb}|^2 |\mathcal {G}(w)|^2,\end{aligned}$$

144 $$\begin{aligned} \frac{\hbox {d}\Gamma _{B^-\rightarrow D^{0*}\ell ^-\bar{\nu }}}{\hbox {d}w}&= \frac{G^2_\mu m^3_{D^*}}{4\pi ^3}(m_B-m_{D^*})^2(w^2-1)^{1/2}\nonumber \\&\times \,|\eta _{\mathrm {EW}}|^2|V_{cb}|^2\chi (w)|\mathcal {F}(w)|^2 , \end{aligned}$$

where $w \equiv v_B \cdot v_{D^{(*)}}$, $v_P=p_P/m_P$ are the four-velocities of the mesons, and $\eta _{\mathrm {EW}}=1.0066$ is the one-loop electroweak correction [443]. The function $\chi (w)$ in Eq. (144) depends upon the recoil $w$ and the meson masses, and reduces to unity at zero recoil [438]. These formulae do not include terms that are proportional to the lepton mass squared which can be neglected for $\ell = e, \mu$.

Most unquenched lattice calculations for $B \rightarrow D^* \ell \nu$ and $B \rightarrow D l \nu$ decays to date focus on the form factors at zero recoil [444, 445] $\mathcal{F}^{B \rightarrow D^*}(1)$ and $\mathcal{G}^{B \rightarrow D}(1)$. These can then be combined with experimental input to extract $|V_{cb}|$. The main reasons for concentrating on the zero recoil point are that (i) the decay rate then depends on a single form factor, and (ii) for $B \rightarrow D^*\ell \nu$, there are no $\mathcal{O}(\Lambda _\mathrm{QCD}/m_Q)$ contributions due to Luke’s theorem. Further, the zero recoil form factor can be computed via a double ratio in which most of the current renormalisation cancels and heavy-quark discretisation errors are suppressed by an additional power of $\Lambda _\mathrm{QCD}/m_Q$.

Some recent work on $B \rightarrow D^{(*)}\ell \nu$ transitions has started to explore the dependence of the relevant form factors on the momentum transfer, but these results are not yet published. The methodology for this is similar to the one employed in $B\rightarrow \pi \ell \nu$ transitions; we refer the reader to Sect. 8.3 for a detailed discussion. Also recently, first results have appeared for $B_{s} \rightarrow D_{s}\ell \nu$ amplitudes, again including information about the momentum-transfer dependence; this will allow for an independent determination of $|V_{cb}|$ as soon as experimental data are available for these transitions.

#### $B_{(s)} \rightarrow D_{(s)}$ decays

Until recently, the only unquenched lattice result for the $B \rightarrow D \ell \nu$ form factor $\mathcal{G}^{B \rightarrow D}(1)$ at zero recoil had appeared in a 2004 conference proceeding by FNAL/MILC [446]. This calculation employs MILC $N_\mathrm{f} = 2 +1$ configurations at a single lattice spacing, again with Fermilab bottom and charm quarks and Asqtad staggered light quarks. Three values of the light-quark mass are used and results extrapolated linearly to the chiral limit. The preliminary result is $\mathcal{G}^{B \rightarrow D}(1) = 1.074(18)(16)$.

The FNAL/MILC study of $B \rightarrow D \ell \nu$ transitions is now being greatly updated by considering several lattice spacings and quark masses, as well as transitions outside the zero recoil limit. Preliminary results have been published in conference proceedings [447], following the strategy previously outlined in [448]. This work employs ensembles at four values of the lattice spacing ranging between approximately 0.045 and 0.12 fm, and four values of the light-quark mass corresponding to pions with RMS masses ranging between 330 and 470 MeV.

The quantities directly studied are the form factors $h_\pm$ defined by

145 $$\begin{aligned} \frac{{\langle }D(p_{D})| i\bar{c} \gamma _\mu b| B(p_B)\rangle }{\sqrt{m_D m_B}}&= h_+(w)(v_B+v_D)_\mu \,\nonumber \\&+ h_-(w)(v_B-v_D)_\mu , \end{aligned}$$

which are related to the standard vector and scalar form factors by

146 $$\begin{aligned}&f_+(q^2) = \frac{1}{2\sqrt{r}}\,\left[ (1+r)h_+(w)-(1-r)h_-(w)\right] , \nonumber \\&\quad f_0(q^2) = \sqrt{r}\left[ \frac{1+w}{1+r}\,h_+(w)\,+\,\frac{1-w}{1-r}\,h_-(w)\right] , \end{aligned}$$

with $r=m_D/m_B$. (Recall that $q^2=(p_B-p_D)^2=m_B^2+m_D^2-2wm_Bm_D$.) The hadronic form factor relevant for experiment, $\mathcal {G}(w)$, is then obtained from the relation $\mathcal {G}(w)=4rf_+(q^2)/(1+r)$. The form factors are obtained from double ratios of three-point functions in which the flavour-conserving current renormalisation factors cancel. The remaining matching factor $\rho _{V^\mu _{cb}}$ is estimated with one-loop lattice perturbation theory.

In order to obtain $h_\pm (w)$ the results are fitted to an ansatz that contains the light-quark mass and lattice spacing dependence predicted by next-to-leading order rSHMChPT, and the leading dependence on $m_{c}$ predicted by the heavy quark expansion ($1/m_{c}^2$ for $h_+$ and $1/m_{c}$ for $h_-$). The $w$-dependence, which allows for an interpolation in $w$, is given by analytic terms up to $(1-w)^2$, as well as a contribution from the log proportional to $g^2_{D^*D\pi }$. The total systematic error is 2.1 % for $h_+$ and 22 % for $h_-$ (note that $h_-$ is of $\mathcal{O}(1-w)$ in the recoil parameter, while $h_+$ is of $\mathcal{O}(1)$), where the error budget is dominated by the heavy-quark discretisation (estimated from HQET) in the case of $h_+$, and by the perturbative current matching factor for $h_-$.

Synthetic data points at three values of $w$ that cover the simulated range are generated for $h_\pm (w)$, from which the form factors $f_{+,0}$ are reconstructed and their $q^2$-dependence fitted to a $z$-parameterisation of the BGL form [349], cf. Sect. 8.3. The values of the series coefficients and their correlations are not given in the conference proceedings, but are left for a forthcoming full publication. From the fit result one can extract, in particular, the value of the relevant hadronic form factor at zero recoil

147 $$\begin{aligned} \mathcal{G}^{B \rightarrow D}(1) = 1.081(25). \end{aligned}$$

Another recent work [449] provides the first study of $B_{s} \rightarrow D_{s}\ell \nu$ transitions with $N_\mathrm{f}=2$ flavours of dynamical quarks, using the publicly available ETMC configurations obtained with the twisted-mass QCD action at maximal twist. Four values of the lattice spacing, ranging between 0.054 and 0.098 fm, are considered, with physical box lengths ranging between 1.7 and 2.7 fm. At two values of the lattice spacing two different physical volumes are available. Charged-pion masses range between $\approx$270 MeV and $\approx$490 MeV, with two or three masses available per lattice spacing and volume, save for the $a \approx 0.054~\mathrm{fm}$ point at which only one light mass is available for each of the two volumes. The strange and heavy valence quarks are also treated with maximally twisted-mass QCD.

The quantities of interest are again the form factors $h_\pm$ defined above. In order to control discretisation effects from the heavy quarks, a strategy similar to the one employed by the ETM Collaboration in their studies of $B$-meson decay constants (cf. Sect. 8.1) is employed: the value of $\mathcal{G}(w)$ is computed at a fixed value of $m_{c}$ and several values of a heavier quark mass $m_{h}^{(k)}=\lambda ^k m_{c}$, where $\lambda$ is a fixed scaling parameter, and step-scaling functions are built as

148 $$\begin{aligned} \Sigma _k(w) = \frac{\mathcal{G}(w,\lambda ^{k+1} m_{c},m_{c},a^2)}{\mathcal{G}(w,\lambda ^k m_{c},m_{c},a^2)}. \end{aligned}$$

Each ratio is extrapolated to the continuum limit, $\sigma _k(w)=\lim \nolimits _{a \rightarrow 0}\Sigma _k(w)$. One then exploits the fact that the $m_{h} \rightarrow \infty$ limit of the step-scaling is fixed—in particular, it is easy to find from the heavy-quark expansion that $\lim _{m_{h}\rightarrow \infty }\sigma (1)=1$. In this way, the physical result at the $b$-quark mass can be reached by interpolating $\sigma (w)$ between the charm region (where the computation can be carried out with controlled systematics) and the known static limit value.

In practice, the values of $m_{c}$ and $m_{s}$ are fixed at each value of the lattice spacing such that the experimental kaon and $D_{s}$ masses are reached at the physical point, as determined in [60]. For the scaling parameter $\lambda =1.176$ is chosen, and eight step-scaling steps are performed, reaching $m_{h}/m_{c}=1.176^9\simeq 4.30$, approximately corresponding to the ratio of the physical $b$ and $c$ masses in the $\overline{\mathrm{MS}}$ scheme at 2 GeV. All observables are obtained from ratios that do not require (re)normalisation. The ansatz for the continuum and chiral extrapolation of $\Sigma _k$ contains a constant and linear terms in $m_\mathrm{sea}$ and $a^2$. Twisted boundary conditions in space are used for valence-quark fields for better momentum resolution. Applying this strategy the form factors are finally obtained at four reference values of $w$ between 1.004 and 1.062, and, after a slight extrapolation to $w=1$, the result is quoted

149 $$\begin{aligned} \mathcal{G}^{B_{s} \rightarrow D_{s}}(1) = 1.052(46). \end{aligned}$$

The authors also provide values for the form factor relevant for the meson states with light valence quarks, obtained from a similar analysis to the one described above for the $B_{s}\rightarrow D_{s}$ case. Values are quoted from fits with and without a linear $m_\mathrm{sea}/m_{s}$ term in the chiral extrapolation. The result in the former case, which safely covers systematic uncertainties, is

150 $$\begin{aligned} \mathcal{G}^{B \rightarrow D}(1)=1.033(95). \end{aligned}$$

Given the identical strategy, and the small sensitivity of the ratios used in their method to the light valence- and sea-quark masses, we assign this result the same ratings in Table 29 as those for their calculation of $\mathcal{G}^{B_{s} \rightarrow D_{s}}(1)$. Currently the precision of this calculation is not competitive with that of FNAL/MILC 13A, but this is due largely to the small number of configurations analysed by Atoui et al. The viability of their method has been clearly demonstrated, however, which leaves significant room for improvement on the errors of both the $B \rightarrow D$ and $B_{s} \rightarrow D_{s}$ form factors with this approach by including either additional two-flavour data or analysing more recent ensembles with $N_\mathrm{f}>2$.

**Table 29:** Lattice results for the $B \rightarrow D^* \ell \nu$, $B\rightarrow D\ell \nu$ and $B_{s} \rightarrow D_{s} \ell \nu$ semileptonic form factors and $R(D)$

Collaboration	Ref.	$N_\mathrm{f}$	Publication status	Continuum extrapolation	Chiral extrapolation	Finite volume	Renormalisation	Heavy-quark treatment	Form factor	
FNAL/MILC 13B	[447]	$2+1$	C$^\triangledown$						${\mathcal F}^{B\rightarrow D^*} (1)$	0.906 (4) (12)
FNAL/MILC 10	[444]	$2+1$	C^§^						${\mathcal F}^{B\rightarrow D^*} (1)$	0.9017 (51) (87) (83) (89) (30) (33)$^\ddagger$
FNAL/MILC 08	[445]	$2+1$	A						${\mathcal F}^{B\rightarrow D^*} (1)$	0.921 (13) (8) (8) (14) (6) (3) (4)
FNAL/MILC 13B	[447]	$2+1$	C						${\mathcal G}^{B\rightarrow D} (1)$	1.081 (25)
FNAL/MILC 04A	[446]	$2+1$	C			$^*$	$^\dagger$		${\mathcal G}^{B\rightarrow D} (1)$	1.074 (18) (16)
FNAL/MILC 12A	[453]	$2+1$	A						$R (D)$	0.316 (12) (7)
Atoui 13	[449]	2	P				–		${\mathcal G}^{B\rightarrow D} (1)$	1.033 (95)
Atoui 13	[449]	2	P				–		${\mathcal G}^{B_{s}\rightarrow D_{s}} (1)$	1.052 (46)

$^\triangledown$ Update of FNAL/MILC 08 for Lattice 2013

^§^ Update of FNAL/MILC 08 for CKM 2010

$^\ddagger$ Value of ${\mathcal F}(1)$ presented in Ref. [444] includes 0.7 % correction $\eta _{EW}$. This correction is unrelated to the lattice calculation and has been removed here

$^*$ No explicit estimate of FV error, but it is expected to be small

$^\dagger$ No explicit estimate of perturbative truncation error in vector-current renormalisation factor, but it is expected to be small because of mostly non-perturbative approach

Finally, Atoui et al. also study the scalar and tensor form factors, as well as the momentum-transfer dependence of $f_{+,0}$. The value of the ratio $f_0(q^2)/f_+(q^2)$ is provided at a reference value of $q^2$ as a proxy for the slope of $\mathcal{G}(w)$ around the zero-recoil limit.

#### $B \rightarrow D^*$ decays

The most precise computation of the zero-recoil form factors needed for the determination of $|V_{cb}|$ from exclusive $B$ semileptonic decays comes from the $B \rightarrow D^* \ell \nu$ form factor at zero recoil, $\mathcal{F}^{B \rightarrow D^*}(1)$, calculated by the Fermilab Lattice and MILC Collaborations [444, 445]. This work uses the MILC $N_\mathrm{f} = 2 + 1$ ensembles. The bottom and charm quarks are simulated using the clover action with the Fermilab interpretation and light quarks are treated via the Asqtad staggered fermion action. At zero recoil $\mathcal{F}^{B \rightarrow D^*}(1)$ reduces to a single form factor, $h_{A_1}(1)$, coming from the axial-vector current

151 $$\begin{aligned} {\langle }D^*(v,\epsilon ^\prime )| \mathcal{A}_\mu | \overline{B}(v) {\rangle }= i \sqrt{2m_B 2 m_{D^*}} \; {\epsilon ^\prime _\mu }^*h_{A_1}(1), \end{aligned}$$

where $\epsilon ^\prime$ is the polarisation of the $D^*$. Reference  [445] introduces a new ratio of three-point correlators which directly gives $|h_{A_1}(1)|$:

152 $$\begin{aligned} \mathcal{R}_{A_1} \!=\! \frac{{\langle }D^*|\bar{c} \gamma _j \gamma _5 b | \overline{B} {\rangle }\; {\langle }\overline{B}| \bar{b} \gamma _j \gamma _5 c | D^* \rangle }{{\langle }D^*|\bar{c} \gamma _4 c | D^* {\rangle }\; {\langle }\overline{B}| \bar{b} \gamma _4 b | \overline{B} \rangle } \!=\! |h_{A_1}(1)|^2. \end{aligned}$$

In Ref. [445] simulation data are obtained on MILC ensembles with three lattice spacings, $a \approx 0.15$, 0.12, and 0.09 fm, for two, four or three different light-quark masses respectively. Results are then extrapolated to the physical, continuum/chiral, limit employing staggered $\chi$PT.

The $D^*$-meson is not a stable particle in QCD and decays predominantly into a $D$ plus a pion. Nevertheless, heavy–light meson $\chi$PT can be applied to extrapolate lattice simulation results for the $B\rightarrow D^*\ell \nu$ form factor to the physical light-quark mass. The $D^*$ width is quite narrow, 0.096 MeV for the $D^{*\pm }(2010)$ and less than 2.1 MeV for the $D^{*0}(2007)$, making this system much more stable and long lived than the $\rho$ or the $K^*$ systems. The fact that the $D^* - D$ mass difference is close to the pion mass leads to the well-known “cusp” in $\mathcal{R}_{A_1}$ just above the physical pion mass [450–452]. This cusp makes the chiral extrapolation sensitive to values used in the $\chi$PT formulae for the $D^*D \pi$ coupling $g_{D^*D\pi }$. The error budget in Ref. [445] includes a separate error of 0.9 % coming from the uncertainty in $g_{D^*D \pi }$ in addition to general chiral-extrapolation errors in order to take this sensitivity into account.

The final value presented in [445], $\mathcal{F}^{B \rightarrow D^*}(1) \!=\! h_{A_1}(1) \!=\! 0.921(13)(20)$, where the first error is statistical, and the second the sum of systematic errors added in quadrature, has a total error of 2.6 %. This result is updated in Ref. [444] after increasing statistics and adding data from $a \approx 0.06$ fm lattices, and even further in Ref. [447] adding data from an $a \approx 0.045$ fm ensemble. The latest value is

153 $$\begin{aligned} \mathcal{F}^{B \rightarrow D^*}(1) = 0.906(4)_\mathrm{stat}(12)_\mathrm{sys}, \end{aligned}$$

with the total error reduced to 1.4 %. The largest systematic uncertainty comes from discretisation errors followed by effects of higher-order corrections in the chiral perturbation theory ansatz.

#### $B \rightarrow D^{(*)} \tau \nu$ decays

Another interesting semileptonic process is $B \rightarrow D^{(*)} \tau \nu$. Here the mass of the outgoing charged lepton cannot be neglected in the decay rate formula, so that both vector and scalar form factors come into play. Recently Babar announced their first observations of the semileptonic decays of $B$-mesons into third generation leptons at a rate in slight excess over SM expectations. Since the lepton mass is now large enough for the branching fraction $\mathcal{B}(B \rightarrow D \tau \nu )$ to be sensitive to the scalar form factor $f_0(q^2)$, this could be a hint for some New Physics scalar exchange contribution. Accurate SM predictions for the ratio

154 $$\begin{aligned} R(D^{(*)}) \!=\! \mathcal{B}(B \!\rightarrow \! D^{(*)} \tau \nu ) / \mathcal{B}(B \!\rightarrow \! D^{(*)} \ell \nu )\quad \hbox {with}\;\ell \!=\!e,\mu \nonumber \\ \end{aligned}$$

have therefore become important and timely. FNAL/MILC has published the first unquenched lattice determination of $R(D)$ [453]. They use a subset of the MILC ensembles from the ongoing $B \rightarrow D\ell \nu$ semileptonic project [448], namely two light-quark masses each on $a \approx 0.12$ and $0.09$ fm lattices, and find,

155 $$\begin{aligned} R(D) = 0.316(12)(7). \end{aligned}$$

This SM prediction is about $\sim$1.7$\sigma$ lower than the Babar measurement.

#### Ratios of $B$ and $B_{s}$ semileptonic decay form factors

**Fig. 21:**
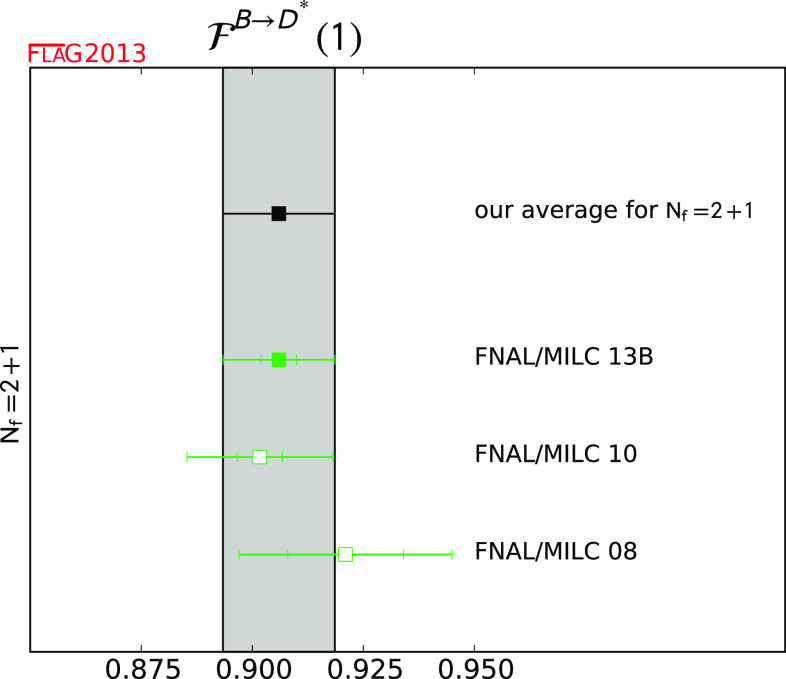
$B \rightarrow D^* \ell \nu$ semileptonic form factor at zero recoil [values in Table 29 and Eq. (157)]

In addition to $B \rightarrow D\ell \nu$ semileptonic decays there is also interest in $B_{s} \rightarrow D_{s} \ell \nu$ semileptonic decays. In particular, $[B_{s} \rightarrow D_{s} \ell \nu ] / [B \rightarrow D \ell \nu ]$ semileptonic form factor ratios can be used to obtain ratios of $B_{q}$-meson ($q = d,s$) fragmentation fractions, $f_{s} / f_{d}$. This latter ratio enters into LHCb’s analysis of $B_{s} \rightarrow \mu ^+ \mu ^-$ decays. There is now one unquenched calculation by FNAL/MILC of ratios of the scalar form factors $f_0^{(q)}(q^2)$ [454]:

156 $$\begin{aligned}&f_0^{(s)}(M_\pi ^2) / f_0^{(d)}(M_K^2) = 1.046(44)(15), \nonumber \\&\quad f_0^{(s)}(M_\pi ^2) / f_0^{(d)}(M_\pi ^2) = 1.054(47)(17), \end{aligned}$$

where the first error is statistical and the second systematic. These results lead to fragmentation fraction ratios $f_{s}/f_{d}$ that are consistent with LHCb’s measurements via other methods (Fig. 21).

#### Summary

In Table 29 we summarise the existing results for the $B \rightarrow D^* \ell \nu$, $B \rightarrow D \ell \nu$, and $B_{s} \rightarrow D _{s}\ell \nu$ form factors at zero recoil, $\mathcal{F}^{B \rightarrow D^*}(1)$, $\mathcal{G}^{B \rightarrow D}(1)$, and $\mathcal{G}^{B_{s} \rightarrow D_{s}}(1)$, as well as for the ratio $R(D) = \mathcal{B}(B \rightarrow D \tau \nu ) / \mathcal{B}(B \rightarrow D l \nu )$. Further details of the lattice calculations are provided in Appendix B.6.4. Selecting those results that are published in refereed journals (or are straightforward updates thereof) and have no red tags, our averages for $\mathcal{F}^{B \rightarrow D^*}(1)$ and $R(D)$ are

157 $$\begin{aligned} N_\mathrm{f}&= 2 + 1 : \quad \mathcal{F}^{B \rightarrow D^*} = 0.906(4)(12),\nonumber \\&R(D) = 0.316(12)(7). \end{aligned}$$

### Determination of $|V_{ub}|$

We now use the lattice-determined Standard Model transition amplitudes for leptonic (Sect. 8.1) and semileptonic (Sect. 8.3) $B$-meson decays to obtain exclusive determinations of the CKM matrix element $|V_{ub}|$. The relevant formulae are Eqs. (106) and (127). Among leptonic channels the only input comes from $B\rightarrow \tau \nu _\tau$, since the rates for decays to $e$ and $\mu$ have not yet been measured. In the semileptonic case we only consider $B\rightarrow \pi \ell \nu _\ell$ transitions (experimentally measured for $\ell =e,\mu$), since no theoretical prediction for hadronic effects in other $b\rightarrow u$ transitions is currently available that satisfies FLAG requirements for controlled systematics.

The branching fraction for the decay $B\rightarrow \tau \nu _\tau$ has been measured by the Belle and Babar collaborations with both semileptonic [455, 456] and hadronic tagging [457, 458] methods. The uncertainties in these measurements are still dominated by statistical errors, and none of them individually are of 5$\sigma$ significance. When combined, however, they cross the threshold needed to establish discovery of this mode. Until recently, the various largely independent measurements have agreed well within errors. Earlier this year, however, the Belle collaboration published the single-most precise measurement of $B\rightarrow \tau \nu$ using the hadronic tagging method with an improved efficiency and the full data set [458], and obtained a result which is more than 2$\sigma$ below the previous average [74, 126]. The errors on the analogous measurement from Babar [457] are not competitive due to the smaller available data set, and the Babar result has not yet been published.

Both Belle and Babar quote averages of the hadronic and the semileptonic tagging modes that we can use to obtain $|V_{ub}|$. In the case of Belle, the average $BR(B^+ \rightarrow \tau ^+ \nu _\tau )=(0.96 \pm 0.26)\times 10^{-4}$ [458] includes slight correlations between systematics with the two tagging methods, but it does not include a rescaling factor due to the fact that the hadronic and semileptonic measurements are inconsistent at the $\sim$1.5$\sigma$ level. The Babar average $BR(B^+ \rightarrow \tau ^+ \nu _\tau )=(1.79 \pm 0.48)\times 10^{-4}$ [457] neglects correlations. By combining these values with the mean $B^+$-meson lifetime $\tau _{B^+}=1.641(8)~\mathrm{ps}$ quoted by the PDG, and our averages $f_B = (189\pm 8)~\mathrm{MeV}~(N_\mathrm{f}=2)$, $f_B= 190.5 \pm 4.2~\mathrm{MeV}~(N_\mathrm{f}=2+1)$ and $f_B= 186 \pm 4~\mathrm{MeV}~(N_\mathrm{f}=2+1+1)$ for the $B$-meson decay constants, we obtain

158 $$\begin{aligned} \begin{aligned}&\!\hbox {Belle}~B\rightarrow \tau \nu _\tau : \quad |V_{ub}| = 3.90(53)(17) \times 10^{-3},\\&\quad N_\mathrm{f}=2,\\&\!\hbox {Belle}~B\rightarrow \tau \nu _\tau : \quad |V_{ub}| = 3.87(52)(9) \times 10^{-3},\\&\quad N_\mathrm{f}=2+1,\\&\!\hbox {Belle}~B\rightarrow \tau \nu _\tau : \quad |V_{ub}| = 3.96(54)(9) \times 10^{-3},\\&\quad N_\mathrm{f}=2+1+1\,;\\&\!\hbox {Babar}~B\rightarrow \tau \nu _\tau : \quad |V_{ub}| = 5.32(71)(23) \times 10^{-3},\\&\quad N_\mathrm{f}=2,\\&\!\hbox {Babar}~B\rightarrow \tau \nu _\tau : \quad |V_{ub}| = 5.28(71)(12) \times 10^{-3},\\&\quad N_\mathrm{f}=2+1,\\&\!\hbox {Babar}~B\rightarrow \tau \nu _\tau : \quad |V_{ub}| = 5.41(73)(12) \times 10^{-3},\\&\quad N_\mathrm{f}=2+1+1, \end{aligned} \end{aligned}$$

where the first error comes from experiment and the second comes from the uncertainty in $f_B$. We can also average all four results for $BR(B^+ \rightarrow \tau ^+ \nu _\tau )$ from Belle and Babar. The measurements using hadronic and semileptonic tagging are statistically independent; further, because the measurements are dominated by statistical errors, the correlations between systematic errors in the two approaches can be reasonably neglected. We obtain $BR(B^+ \rightarrow \tau ^+ \nu _\tau ) = (1.12 \pm 0.28) \times 10^{-4}$, where we have applied a $\sqrt{(\chi ^2/\hbox {dof})} \sim 1.3$ rescaling factor because the Belle hadronic tagging measurement differs significantly from the other three. Using this value for the branching fraction, and again combining with the $N_\mathrm{f}=2$, $N_\mathrm{f}=2+1$ and $N_\mathrm{f}=2+1+1$ lattice-QCD averages for $f_B$ from Eqs. (110)–(112), our preferred determinations of $|V_{ub}|$ from leptonic $B\rightarrow \tau \nu$ decay are

159 $$\begin{aligned} \hbox {Belle + Babar}~B\rightarrow \tau \nu _\tau :&\quad |V_{ub}| = 4.21(53)(18) \times 10^{-3},\nonumber \\&\quad N_\mathrm{f}=2,\nonumber \\ \hbox {Belle + Babar}~B\rightarrow \tau \nu _\tau :&\quad |V_{ub}| = 4.18(52)(9) \times 10^{-3},\nonumber \\&\quad N_\mathrm{f}=2+1,\nonumber \\ \hbox {Belle + Babar}~B\rightarrow \tau \nu _\tau :&\quad |V_{ub}| = 4.28(53)(9) \times 10^{-3},\nonumber \\&\quad N_\mathrm{f}=2+1+1. \end{aligned}$$

In semileptonic decays, the experimental value of $|V_{ub}|f_+(q^2)$ can be extracted from the measured branching fractions of $B^0\rightarrow \pi ^-\ell ^+\nu$ decays by applying Eq. (127); $|V_{ub}|$ can then be determined by performing fits to the constrained BCL $z$-parameterisation of the form factor $f_+(q^2)$ given in Eq. (140). This can be done in two ways: one option is to perform separate fits to lattice (cf. Sect. 8.3) and experimental results, and extract the value of $|V_{ub}|$ from the ratio of the respective $a_0$ coefficients; a second option is to perform a simultaneous fit to lattice and experimental data, leaving their relative normalisation $|V_{ub}|$ as a free parameter. We adopt the second strategy because it more optimally combines the lattice and experimental information and minimises the uncertainty in $|V_{ub}|$. As experimental input we take the latest untagged 12-bin Babar data [353] and 13-bin Belle data [352], and we assume no correlation between experimental and lattice data. As in the fit to lattice data only in Sect. 8.3, we assume that the statistics plus chiral-extrapolation errors are 100 % correlated between the FNAL/MILC 08A and HPQCD 06 data, and we reduce the correlations in the FNAL/MILC data by keeping only every second data point.

**Fig. 22:**
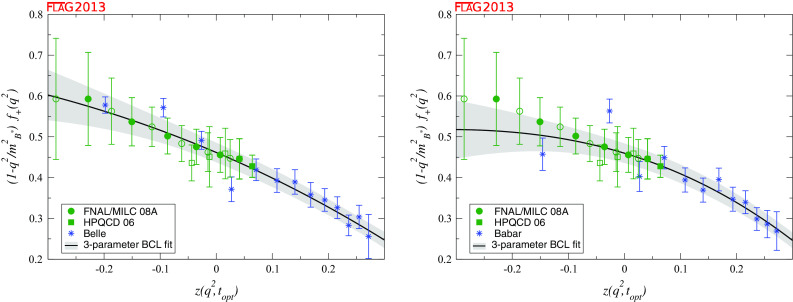
Lattice and experimental data for $(1 - q^2/m_{B^*}^2) f_+(q^2)$ versus $z$. The *filled green symbols* denote lattice-QCD points included in the fit, while the *open green symbols* show those that are not included in the fit (either because of unknown correlations or strong correlations). The *blue stars* show the experimental data divided by the value of $|V_{ub}|$ obtained from the fit. The *grey band* in the *left* (*right*) plots shows the preferred three-parameter BCL fit to the lattice-QCD and Belle (Babar) data with errors

Figure 22 shows both the lattice and experimental data for $(1 - q^2/m_{B^*}^2) f_+(q^2)$ versus $z$. For illustration, the experimental data are divided by the value of $|V_{ub}|$ obtained from the preferred fit. Both the lattice-QCD and experimental data are linear and display no visible signs of curvature; further, the slopes of the lattice and experimental data sets appear consistent. A simple three-parameter constrained BCL fit (i.e. through ${\mathcal O}(z^2)$ plus $|V_{ub}|$) is sufficient to describe the combined data sets with a good $\chi ^2/\hbox {dof}$, however, the addition of the experimental points enables a better determination of higher-order terms in the $z$-expansion than from the lattice-only fit. In order to address the potential systematic uncertainty due to truncating the series in $z$, we continue to add terms to the fit until the result for $|V_{ub}|$ stabilises, i.e. the central value settles and the errors stop increasing. We find that this happens at ${\mathcal O}(z^3)$, and we take the value of $|V_{ub}|$ from this combined fit of the lattice-QCD and experimental data as our preferred result:

160 $$\begin{aligned} \begin{aligned} \hbox {global lattice + Babar:}&\quad |V_{ub}| = 3.37(21) \times 10^{-3},\\&\quad N_\mathrm{f}=2+1,\\ \hbox {global lattice + Belle:}&\quad |V_{ub}| = 3.47(22) \times 10^{-3},\\&\quad N_\mathrm{f}=2+1. \end{aligned} \end{aligned}$$

We do not quote a result for a combined lattice + Babar + Belle fit, since we are unable to properly take into account possible correlations between experimental results. Again, we emphasise the importance of publishing statistical and systematic correlation matrices in future lattice-QCD work on semileptonic form factors, so that the lattice results can be fully used to obtain CKM matrix elements and for other phenomenological applications.

**Fig. 23:**
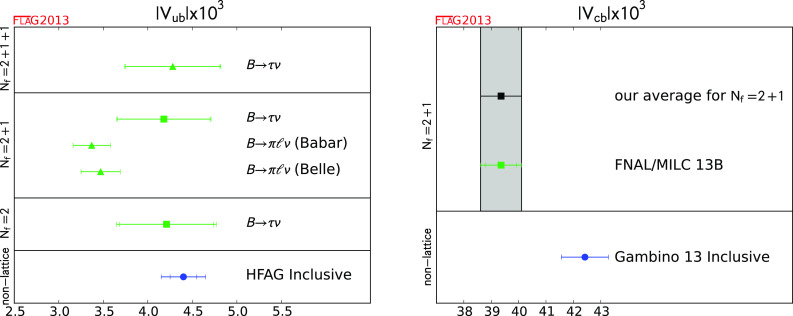
Comparison of the results for $|V_{ub}|$ and $|V_{cb}|$ obtained from lattice methods with non-lattice determinations based on inclusive semileptonic $B$ decays. In the *left plot*, the results denoted by *squares* are from leptonic decays, while those denoted by *triangles* are from semileptonic decays. The *grey band* indicates our $N_\mathrm{f}=2+1$ average

Our results for $|V_{ub}|$ are summarised in Table 30 and Fig. 23, where we also show the inclusive determinations from HFAG for comparison. The spread of values for $|V_{ub}|$ does not yield a clear picture. We observe the well-known $\sim$3$\sigma$ tension between determinations of $|V_{ub}|$ from exclusive and inclusive semileptonic decays. The determination of $|V_{ub}|$ from leptonic $B\rightarrow \tau \nu$ decay lies in between the inclusive and exclusive determinations, but the experimental errors in $\hbox {BR}(B\rightarrow \tau \nu )$ are so large that it agrees with both within $\sim$1.5$\sigma$. If, however, we consider separately the different experimental measurements of $\mathrm{BR}(B\rightarrow \tau \nu )$, the Belle measurement from hadronic tagging leads to a value of $|V_{ub}|$ that agrees well with the one from exclusive $B\rightarrow \pi \ell \nu$ decay, while the remaining Belle and Babar measurements lead to values of $|V_{ub}|$ that are larger than both the latter and inclusive determinations. The exclusive determination of $|V_{ub}|$ will improve in the next few years with better lattice-QCD calculations of the $B\rightarrow \pi \ell \nu$ form factor, while the improvement in $|V_{ub}|$ from $B\rightarrow \tau \nu$ decays will have to wait longer for the Belle II experiment, which aims to begin running in 2016, to collect a larger data set than is currently available.

**Table 30:** Comparison of exclusive determinations of $|V_{ub}|$ (upper panel) and inclusive determinations (lower panel). For $B\rightarrow \tau \nu$, the two uncertainties shown come from experiment (plus non-lattice theory) and from the lattice calculation, respectively. Each inclusive determination corresponds to a different theoretical treatment of the same experimental partial branching fractions compiled by the Heavy Flavour Averaging Group [465]; the errors shown are experimental and theoretical, respectively

	From	$|V_{ub}|\times 10^3$
Our result for $N_\mathrm{f} = 2$	$B\rightarrow \tau \nu$	4.21 (53) (18)
Our result for $N_\mathrm{f} = 2+1$	$B\rightarrow \tau \nu$	4.18 (52) (9)
Our result for $N_\mathrm{f} = 2+1+1$	$B\rightarrow \tau \nu$	4.28 (53) (9)
Our result for $N_\mathrm{f} = 2+1$	$B\rightarrow \pi \ell \nu$ (Babar)	3.37 (21)
Our result for $N_\mathrm{f} = 2+1$	$B\rightarrow \pi \ell \nu$ (Belle)	3.47 (22)
Bauer 01 [459]	$B\rightarrow X_{u} \ell \nu$	4.62 (20) (29)
Lange 05 [460]	$B\rightarrow X_{u} \ell \nu$	4.40 (15) $(^{+19}_{-21})$
Andersen 05 [461], Gardi 08 [462]	$B\rightarrow X_{u} \ell \nu$	$4.45\ (15)(^{+15}_{-16})$
Gambino 07 [463]	$B\rightarrow X_{u} \ell \nu$	4.39 (15) $(^{+12}_{-14})$
Aglietti 07 [464]	$B\rightarrow X_{u} \ell \nu$	4.03 (13) $(^{+18}_{-12})$
HFAG inclusive average [126]	$B\rightarrow X_{u} \ell \nu$	4.40 (15) (20)

### Determination of $|V_{cb}|$

We now interpret the lattice-QCD results for the $B\rightarrow D^{(*)}\ell \nu$ form factors as determinations of the CKM matrix element $|V_{cb}|$ in the Standard Model.

For the experimental branching fractions at zero recoil, we use the latest experimental averages from the Heavy Flavour Averaging Group [126]:^35^

161 $$\begin{aligned}&{\mathcal F}^{B\rightarrow D^*}(1) \eta _\mathrm{EW} |V_{cb}| = 35.90(45),\nonumber \\&\quad {\mathcal G}^{B\rightarrow D}(1) \eta _{EW} |V_{cb}| = 42.64(1.53). \end{aligned}$$

For ${\mathcal F}^{B\rightarrow D^*}(1)$, there is only a single $N_\mathrm{f} = 2+1$ lattice-QCD calculation that satisfies the FLAG criteria, while there is currently no such calculation of ${\mathcal G}^{B \rightarrow D}(1)$. Using the result given in Eq. (157), we obtain our preferred value for $|V_{cb}|$:

162 $$\begin{aligned} B \!\rightarrow \! D^* \ell \nu : |V_{cb}| \!=\! 39.36(56)(50) \!\times \! 10^{-3}, \quad N_\mathrm{f}\!=\!2\!+\!1\nonumber \\ \end{aligned}$$

where the errors shown are from the lattice calculation and experiment (plus non-lattice theory), respectively. Table 31 compares the determination of $|V_{cb}|$ from exclusive $B\rightarrow D^* \ell \nu$ decays to that from inclusive $B \rightarrow X_{c} \ell \nu$ decays, where $X_{c}$ denotes all possible charmed hadronic final states. The results, also shown in Fig. 23, differ by approximately 2.7$\sigma$. The exclusive determination of $|V_{cb}|$ will improve significantly over the next year or two with new lattice-QCD calculations of the $B \rightarrow D^{(*)} \ell \nu$ form factors at non-zero recoil.

**Table 31:** Determinations of $|V_{cb}|$ obtained from semileptonic $B$ decay. The errors shown in the first row indicate those from lattice and experimental (plus non-lattice theory) uncertainties, respectively, while the error shown in the second row is the total (experimental plus theoretical) uncertainty

	Ref.	From	$|V_{cb}| \times 10^3$
Our average for $N_\mathrm{f} = 2+1$	[444]	$B\rightarrow D^* \ell \nu$	39.36 (56) (50)
Inclusive (Gambino 13)	[466]	$B\rightarrow X_{c} \ell \nu$	42.42 (86)

## The strong coupling $\alpha _\mathrm{s}$

### Introduction

The strong coupling $\bar{g}(\mu )$ defined at scale $\mu$, plays a key rôle in the understanding of QCD and in its application for collider physics. For example, the parametric uncertainty from $\alpha _\mathrm{s}$ is one of the dominant sources of uncertainty in the Standard Model prediction for the $H \rightarrow b\bar{b}$ partial width, and the largest source of uncertainty for $H \rightarrow gg$. Thus higher precision determinations $\alpha _\mathrm{s}$ are needed to maximise the potential of experimental measurements at the LHC, and for high-precision Higgs studies at future colliders [467–469]. The value of $\alpha _\mathrm{s}$ also yields one of the essential boundary conditions for completions of the standard model at high energies.

In order to determine the running coupling at scale $\mu$

163 $$\begin{aligned} \alpha _\mathrm{s}(\mu ) = { \bar{g}^2(\mu ) \over 4\pi }, \end{aligned}$$

we should first “measure” a short-distance quantity ${\mathcal {O}}$ at scale $\mu$ either experimentally or by lattice calculations and then match it with a perturbative expansion in terms of a running coupling, conventionally taken as $\alpha _{\overline{\mathrm{MS}}}(\mu )$,

164 $$\begin{aligned} {\mathcal {O}}(\mu ) = c_1 \alpha _{\overline{\mathrm{MS}}}(\mu ) + c_2 \alpha _{\overline{\mathrm{MS}}}(\mu )^2 + \cdots . \end{aligned}$$

The essential difference between continuum determinations of $\alpha _\mathrm{s}$ and lattice determinations is the origin of the values of ${\mathcal {O}}$ in Eq. (164).

The basis of continuum determinations are experimentally measurable cross sections from which ${\mathcal {O}}$ is defined. These cross sections have to be sufficiently inclusive and at sufficiently high scales such that perturbation theory can be applied. Often hadronisation corrections have to be used to connect the observed hadronic cross sections to the perturbative ones. Experimental data at high $\mu$, where perturbation theory is progressively more precise, usually have increasing experimental errors, and it is not easy to find processes which allow one to follow the $\mu$ dependence of a single ${\mathcal {O}}(\mu )$ over a range where $\alpha _\mathrm{s}(\mu )$ changes significantly and precision is maintained.

In contrast, in lattice gauge theory, one can design ${\mathcal {O}}(\mu )$ as Euclidean short-distance quantities which are not directly related to experimental observables. This allows us to follow the $\mu$ dependence until the perturbative regime is reached and non-perturbative “corrections” are negligible. The only experimental input for lattice computations of $\alpha _\mathrm{s}$ is the hadron spectrum which fixes the overall energy scale of the theory and the quark masses. Therefore experimental errors are completely negligible and issues such as hadronisation do not occur. We can construct many short-distance quantities that are easy to calculate non-perturbatively in lattice simulations with small statistical uncertainties. We can also simulate at parameter values that do not exist in nature (for example with unphysical quark masses between bottom and charm) to help control systematic uncertainties. These features mean that very precise results for $\alpha _\mathrm{s}$ can be achieved with lattice gauge-theory computations. Further, as in the continuum, the many different methods available to determine $\alpha _\mathrm{s}$ in lattice calculations with different associated systematic uncertainties enable valuable cross-checks. Practical limitations are discussed in the next section, but a simple one is worth mentioning here. Experimental results (and therefore the continuum determinations) of course have all quarks present, while in lattice gauge theories only the light ones are included and one then is forced to use the matching at thresholds, as discussed in the following subsection.

It is important to keep in mind that the dominant source of uncertainty in most present day lattice-QCD calculations of $\alpha _\mathrm{s}$ is from the truncation of either continuum or lattice perturbation theory. Perturbative truncation errors are of a different nature than most other lattice (or experimental) systematics, in that they often cannot be estimated from studying the data themselves. Further, the size of higher-order coefficients in the perturbative series can sometimes turn out to be larger than naive expectations based on power-counting from the behaviour of lower-order terms. Therefore for the purposes of this review we choose to be cautious in the range presented in Sect. 9.9 for $\alpha ^{(5)}_{\overline{\mathrm{MS}}}(M_Z)$ from lattice calculations.

The various phenomenological approaches to determining the running coupling, $\alpha ^{(5)}_{\overline{\mathrm{MS}}}(M_Z)$ are summarised by the Particle Data Group [74]. The PDG review lists four categories of phenomenological results used to obtain the running coupling using hadronic $\tau$ decays, hadronic final states of $e^+e^-$ annihilation, deep inelastic lepton–nucleon scattering and electroweak precision data. Excluding lattice results, the PDG quotes a weighted average of

165 $$\begin{aligned} \alpha ^{(5)}_{\overline{\mathrm{MS}}}(M_Z) = 0.1183(12). \end{aligned}$$

For a general overview of the status of the various phenomenological and lattice approaches see e.g. [470]. We note that perturbative truncation errors are also the dominant source of uncertainty in several of the phenomenological determinations of $\alpha _\mathrm{s}$. In particular, the extraction of $\alpha _\mathrm{s}$ from $\tau$ data, which is the most precise and has the largest impact on the non-lattice average in Eq. (165) is especially sensitive to the treatment of higher-order perturbative terms. This is important to keep in mind when comparing our chosen range for $\alpha ^{(5)}_{\overline{\mathrm{MS}}}(M_Z)$ from lattice determinations in Eq. (205) with the non-lattice average from the PDG.

#### Scheme and scale dependence of $\alpha _\mathrm{s}$ and $\Lambda _\mathrm{QCD}$

Despite the fact that the notion of the QCD coupling is initially a perturbative concept, the associated $\Lambda$-parameter is non-perturbatively defined

166 $$\begin{aligned} \Lambda&\equiv \mu \,(b_0\bar{g}^2(\mu ))^{-b_1/(2b_0^2)} e^{-1/(2b_0\bar{g}^2(\mu ))} \nonumber \\&\times \exp \left[ -\int _0^{\bar{g}(\mu )}\,\hbox {d}x \left( {1\over \beta (x)} + {1 \over b_0x^3} - {b_1 \over b_0^2x} \right) \right] , \end{aligned}$$

where $\beta$ is the full renormalisation group function in the scheme which defines $\bar{g}$ and $b_0$ and $b_1$ are the first two scheme-independent coefficients of the perturbative expansion $\beta (x) \sim -b_0 x^3 -b_1 x^5 + \cdots$. Thus the $\Lambda$-parameter is renormalisation scheme dependent but in an exactly computable way, and lattice gauge theory is an ideal method to relate it to the low-energy properties of QCD.

The change in the coupling from one scheme, $S$, to another (taken here to be the $\overline{\mathrm{MS}}$ scheme) is perturbative,

167 $$\begin{aligned} g_{\overline{\mathrm{MS}}}^2(\mu ) = g_{S}^2(\mu ) (1 + c^{(1)}_g g_{S}^2(\mu ) + \cdots ), \end{aligned}$$

where $c^{(i)}_g$ are the finite renormalisation coefficients. The scale $\mu$ must be taken high enough for the error in keeping only the first few terms in the expansion to be small. The conversion to the $\Lambda$-parameter in the $\overline{\mathrm{MS}}$ scheme is given by

168 $$\begin{aligned} \Lambda _{\overline{\mathrm{MS}}}= \Lambda _{S} \exp \left[ c_g^{(1)}/(2b_0)\right] . \end{aligned}$$

By convention $\alpha _{\overline{\mathrm{MS}}}$ is usually quoted at a scale $\mu =M_Z$ where the appropriate effective coupling is the one in the five-flavour theory: $\alpha ^{(5)}_{\overline{\mathrm{MS}}}(M_Z)$. In order to obtain it from a lower-flavour result, one connects effective theories with different number of flavour as discussed by Bernreuther and Wetzel [471]. For example one considers the ${\overline{\mathrm{MS}}}$ scheme, matches the three-flavour theory to the four-flavour theory at a scale given by the charm quark mass, runs with the four-loop beta-function of the four-flavour theory to a scale given by the $b$-quark mass and there matches to the five-flavour theory, after which one runs up to $\mu =M_Z$. For the matching relation at a given quark threshold we use the mass $m_\star$ which satisfies $m_\star = \overline{m}_{\overline{\mathrm{MS}}}(m_\star )$, where $\overline{m}$ is the running mass (analogous to the running coupling). Then

169 $$\begin{aligned} \bar{g}^2_{N_\mathrm{f}-1}(m_\star )&= \bar{g}^2_{N_\mathrm{f}}(m_\star )\times [1+t_2\,\bar{g}^4_{N_\mathrm{f}}(m_\star )\nonumber \\&+t_3\,\bar{g}^6_{N_\mathrm{f}}(m_\star )+ \cdots ] \end{aligned}$$

with [472]

170 $$\begin{aligned} t_2&= {1 \over (4\pi ^2)^2} {11\over 72}\end{aligned}$$

171 $$\begin{aligned} t_3&= {1 \over (4\pi ^2)^3} \left[ - {82043\over 27648}\zeta _3 +{564731\over 124416}-{2633\over 31104}(N_\mathrm{f}-1)\right] \,\nonumber \\ \end{aligned}$$

(where $\zeta _3$ is the Riemann zeta-function) provides the matching at the thresholds in the ${\overline{\mathrm{MS}}}$-scheme. While $t_2$, $t_3$ are numerically small coefficients, the charm threshold scale is also relatively low and so there could be some non-perturbative uncertainties in the matching procedure, which are difficult to estimate. Obviously there is no perturbative matching formula across the strange “threshold”; here matching is entirely non-perturbative. Model-dependent extrapolations of $\bar{g}^2_{N_\mathrm{f}}$ from $N_\mathrm{f}=0,2$ to $N_\mathrm{f}=3$ were done in the early days of lattice gauge theory. We will include these in our listings of results but not in our estimates, since such extrapolations are based on untestable assumptions.

#### Overview of the review of $\alpha _\mathrm{s}$

We begin by explaining lattice-specific difficulties in Sect. 9.2 and the FLAG quality criteria designed to assess whether the associated systematic uncertainties can be controlled and estimated in a reasonable manner. We then discuss, in Sects. 9.3–9.8, the various lattice approaches. For completeness, we present results from calculations with $N_\mathrm{f} = 0, 2, 3$ and 4 flavours. Finally, in Sect. 9.9, we present averages together with our best estimates for $\alpha _{\overline{\mathrm{MS}}}^{(5)}$. These are determined from three- and four-flavour QCD simulations. The earlier $N_\mathrm{f} = 0, 2$ works obtained results for $N_\mathrm{f} = 3$ by extrapolation in $N_\mathrm{f}$. Because this is not a theoretically controlled procedure, we do not include these results in our averages. For the $\Lambda$ parameter, we also give results for other number of flavours, including $N_\mathrm{f}=0$. Even though the latter numbers should not be used for phenomenology, they represent valuable non-perturbative information concerning field theories with variable numbers of quarks.

### Discussion of criteria for computations entering the averages

As in the PDG review, we only use calculations of $\alpha _\mathrm{s}$ published in peer-reviewed journals, and that use NNLO or higher-order perturbative expansions, to obtain our final range in Sect. 9.9. We also, however, introduce further quality criteria designed to assess the ability to control important systematics which we describe here. Some of these criteria, e.g. that for the continuum extrapolation, are associated with lattice-specific systematics and have no continuum analogue. Other criteria, e.g. that for the renormalisation scale, could in principle be applied to non-lattice determinations but are not considered in the PDG average. Expecting that lattice calculations will continue to improve significantly in the near future, our goal in reviewing the state of the art here is to be conservative and avoid prematurely choosing an overly small range.

In lattice calculations, we generally take ${\mathcal {O}}$ to be some combination of physical amplitudes or Euclidean correlation functions which are free from UV and IR divergences and have a well-defined continuum limit. Examples include the force between static quarks and $2$-point functions of quark bilinear currents.

In comparison to values of observables ${\mathcal {O}}$ determined experimentally, those from lattice calculations require two more steps. The first step concerns setting the scale $\mu$ in GeV, where one needs to use some experimentally measurable low-energy scale as input. Ideally one employs a hadron mass. Alternatively convenient intermediate scales such as $\sqrt{t_0}$, $w_0$, $r_0$, $r_1$, [65, 183, 184, 473] can be used if their relation to an experimental dimensionful observable is established. The low-energy scale needs to be computed at the same bare parameters where ${\mathcal {O}}$ is determined, at least as long as one does not use the step-scaling method (see below). This induces a practical difficulty given present computing resources. In the determination of the low-energy reference scale the volume needs to be large enough to avoid finite-size effects. On the other hand, in order for the perturbative expansion of Eq. (164) to be reliable, one has to reach sufficiently high values of $\mu$, i.e. short enough distances. To avoid uncontrollable discretisation effects the lattice spacing $a$ has to be accordingly small. This means

172 $$\begin{aligned} L \gg \hbox {hadron size}\sim \Lambda _\mathrm{QCD}^{-1}\quad \hbox {and} \quad 1/a \gg \mu , \end{aligned}$$

(where $L$ is the box size) and therefore

173 $$\begin{aligned} L/a \ggg \mu /\Lambda _\mathrm{QCD}. \end{aligned}$$

The currently available computer power, however, limits $L/a$, typically to $L/a = 20-64$. Unless one accepts compromises in controlling discretisation errors or finite-size effects, this means one needs to set the scale $\mu$ according to

174 $$\begin{aligned} \mu \lll L/a \times \Lambda _\mathrm{QCD}&\sim \! 5-20\, \hbox {GeV}. \end{aligned}$$

Therefore, $\mu$ can be $1-3\, \hbox {GeV}$ at most. This raises the concern whether the asymptotic perturbative expansion truncated at one loop, two loop, or three loop in Eq. (164) is sufficiently accurate. There is a finite-size scaling method, usually called step-scaling method, which solves this problem by identifying $\mu =1/L$ in the definition of ${\mathcal {O}}(\mu )$; see Sect. 9.3.

For the second step after setting the scale $\mu$ in physical units ($\hbox {GeV}$), one should compute ${\mathcal {O}}$ on the lattice, ${\mathcal {O}}_\mathrm{lat}(a,\mu )$ for several lattice spacings and take the continuum limit to obtain the left hand side of Eq. (164) as

175 $$\begin{aligned} {\mathcal {O}}(\mu ) \equiv \lim _{a\rightarrow 0} {\mathcal {O}}_\mathrm{lat}(a,\mu )\hbox { with }\mu \hbox { fixed}. \end{aligned}$$

This is necessary to remove the discretisation error.

Here it is assumed that the quantity ${\mathcal {O}}$ has a continuum limit, which is regularisation-independent up to discretisation errors. The method discussed in Sect. 9.6, which is based on the perturbative expansion of a lattice-regulated, divergent short-distance quantity $W_\mathrm{lat}(a)$ differs in this respect and must be treated separately.

In summary, a controlled determination of $\alpha _\mathrm{s}$ needs to satisfy the following:

1. The determination of $\alpha _\mathrm{s}$ is based on a comparison of a short-distance quantity ${\mathcal {O}}$ at scale $\mu$ with a well–defined continuum limit without UV and IR divergences to a perturbative expansion formula in Eq. (164).
2. The scale $\mu$ is large enough so that the perturbative expansion in Eq. (164) is precise, i.e. it has good *asymptotic* convergence.
3. If ${\mathcal {O}}$ is defined by physical quantities in infinite volume, one needs to satisfy Eq. (173).

Non-universal quantities need a separate discussion; see Sect. 9.6.

Conditions 2. and 3. give approximate lower and upper bounds for $\mu$, respectively. It is important to see whether there is a window to satisfy 2. and 3. at the same time. If it exists, it remains to examine whether a particular lattice calculation is done inside the window or not.

Obviously, an important issue for the reliability of a calculation is whether the scale $\mu$ that can be reached lies in a regime where perturbation theory can be applied with confidence. However, the value of $\mu$ does not provide an unambiguous criterion. For instance, the Schrödinger Functional, or SF-coupling (Sect. 9.3) is conventionally identified with $\mu =1/L$, but one could also choose $\mu =2/L$. Instead of $\mu$ we therefore define an effective $\alpha _\mathrm{eff}$. For schemes such as SF (see Sect. 9.3) or $qq$ (see Sect. 9.4.1) this is directly the coupling constant of the scheme. For other schemes such as the vacuum polarisation we use the perturbative expansion Eq. (164) for the observable ${\mathcal {O}}$ to define

176 $$\begin{aligned} \alpha _\mathrm{eff} = {\mathcal {O}}/c_1. \end{aligned}$$

If there is an $\alpha _\mathrm{s}$-independent term it should first be subtracted. Note that this is nothing but defining an effective, regularisation-independent coupling, a physical renormalisation scheme.

Let us now comment further on the use of the perturbative series. Since it is only an asymptotic expansion, the remainder $R_n({\mathcal {O}})={\mathcal {O}}-\sum _{i\le n}c_i \alpha _\mathrm{s}^i$ of a truncated perturbative expression ${\mathcal {O}}\sim \sum _{i\le n}c_i \alpha _\mathrm{s}^i$ cannot just be estimated as a perturbative error $k\,\alpha _\mathrm{s}^{n+1}$. The error is non-perturbative. Often one speaks of “non-perturbative contributions”, but non-perturbative and perturbative contributions cannot be strictly separated due to the asymptotic nature of the series (see e.g. [474]).

Still, we do have some general ideas concerning the size of non-perturbative effects. The known ones such as instantons or renormalons decay for large $\mu$ like inverse powers of $\mu$ and are thus roughly of the form

177 $$\begin{aligned} \exp (-\gamma /\alpha _\mathrm{s}), \end{aligned}$$

with some positive constant $\gamma$. Thus we have, loosely speaking,

178 $$\begin{aligned} {\mathcal {O}}&= c_1 \alpha _\mathrm{s} + c_2 \alpha _\mathrm{s}^2 + \dots + c_n\alpha _\mathrm{s}^n+ {\mathrm {O}}(\alpha _\mathrm{s}^{n+1})\nonumber \\&+ {\mathrm {O}}(\exp (-\gamma /\alpha _\mathrm{s})). \end{aligned}$$

For small $\alpha _\mathrm{s}$, the $\exp (-\gamma /\alpha _\mathrm{s})$ is negligible. Similarly the perturbative estimate for the magnitude of relative errors in Eq. (178) is small; as an illustration for $n=3$ and $\alpha _\mathrm{s} = 0.2$ the relative error is $\sim$0.8 % (assuming coefficients $|c_n /c_1 | \sim 1$).

For larger values of $\alpha _\mathrm{s}$ non-perturbative effects can become significant in Eq. (178). An instructive example comes from the values obtained from $\tau$ decays, for which $\alpha _\mathrm{s}\approx 0.3$. Here, different applications of perturbation theory (fixed order, FOPT, and contour improved, CIPT) each look reasonably asymptotically convergent but the difference does not seem to decrease much with the order (see, e.g., the contribution of Pich in [470]). In addition non-perturbative terms in the spectral function may be non-negligible even after the integration up to $m_\tau$ (Golterman in [470]). All of this is because $\alpha _\mathrm{s}$ is not really small.

Since the size of the non-perturbative effects is very hard to estimate one should try to avoid such regions of the coupling. In a fully controlled computation one would like to verify the perturbative behaviour by changing $\alpha _\mathrm{s}$ over a significant range instead of estimating the errors as $\sim \alpha _\mathrm{s}^{n+1}$. Some computations try to take non-perturbative power ‘corrections’ to the perturbative series into account by including such terms in a fit to the $\mu$ dependence. We note that this is a delicate procedure, both because the separation of non-perturbative and perturbative is theoretically not well defined and because in practice a term like, e.g., $\alpha _\mathrm{s}(\mu )^3$ is hard to distinguish from a $1/\mu ^2$ term when the $\mu$-range is restricted and statistical and systematic errors are present. We consider it safer to restrict the fit range to the region where the power corrections are negligible compared to the estimated perturbative error.

The above considerations lead us to the following special quality criteria for the determination of $\alpha _\mathrm{s}$.

$$\bullet $$ Renormalisation scale

all points relevant in the analysis have $\alpha _\mathrm{eff} < 0.2$
all points have $\alpha _\mathrm{eff} < 0.4$ and at least one $\alpha _\mathrm{eff} \le 0.25$
otherwise

$$\bullet $$ Perturbative behaviour

verified over a range of a factor $2$ in $\alpha _\mathrm{eff}$ (without power corrections)
agreement with perturbation theory over a range of a factor 1.5 in $\alpha _\mathrm{eff}$ (possibly fitting with power corrections)
otherwise

$$\bullet $$ Continuum extrapolation

At a reference point of $\alpha _\mathrm{eff} = 0.3$ (or less) we require

three lattice spacings with $\mu a < 1/2$ and full $O(a)$ improvement, or three lattice spacings with $\mu a \le 1/4$ and two-loop $O(a)$ improvement, or $\mu a \le 1/8$ and one-loop $O(a)$ improvement
three lattice spacings with $\mu a < 1.5$ reaching down to $\mu a =1$ and full $O(a)$ improvement, or three lattice spacings with $\mu a \le 1/4$ and one-loop $O(a)$ improvement
otherwise

We here assume that the two-loop relation between the used coupling and $\alpha _{\overline{\mathrm{MS}}}$ is always known such that the three-loop beta-function is known in the scheme considered. Therefore we have no separate criterion for the order of perturbation theory. Similarly we assume that quark mass effects of light quarks (including strange) are negligible in the effective coupling itself where large, perturbative, $\mu$ is considered.

We also need to specify what is meant by $\mu$. For SF we mean $\mu =1/L$, for $qq$ it is $\mu =2/r$, for schemes with observables in momentum space we take the magnitude of the momentum. Finally, for moments of heavy quark currents with quark masses $m_{h}$ we use $\mu =2m_{h}$. We note again that the above criteria cannot be applied when regularisation-dependent quantities $W_\mathrm{lat}(a)$ are used instead of ${\mathcal {O}}(\mu )$. These cases are specifically discussed in Sect. 9.6.

The usual criterion for the chiral extrapolation and the control over finite-volume effects is missing here for the following reason. These criteria would apply only to the setting of the scale. Usually this has been determined in preceding papers of the collaboration determining the coupling constant (or indeed by another collaboration). However, the determination of the scale does not need to be very precise, since using the lowest-order $\beta$-function shows that a 3 % error in the scale determination corresponds to a $\sim$0.5 % error in $\alpha _\mathrm{s}(M_Z)$. So as long as systematic errors from chiral-extrapolation and finite-volume effects are below 3 % we do not need to be concerned about those. This covers practically all cases. When, exceptionally, it matters we include the precision of the scale setting in our discussion.

A popular scale choice is the intermediate $r_0$ scale, although one should also bear in mind that its determination from physical observables has also to be taken into account. The phenomenological value of $r_0$ was originally determined as $r_0 \approx 0.49$ fm through potential models describing quarkonia [65]. Recent determinations from 2-flavour QCD are $r_0 = 0.420(14) - 0.450(14)$ fm by the ETM collaboration [169, 241], using as input $f_\pi$ and $f_K$ and carrying out various continuum extrapolations. On the other hand, the ALPHA collaboration [59] determined $r_0 = 0.503(10)$ fm with input from $f_K$, and the QCDSF Collaboration [475] cites $0.501(10)(11)\,\hbox {fm}$ from the mass of the nucleon (no continuum limit). Recent determinations from three-flavour QCD are consistent with $r_1 = 0.313(3)\,\hbox {fm}$ and $r_0 = 0.472(5)\,\hbox {fm}$ [159, 186, 476]. Due to the uncertainty in these estimates, and as many results are based directly on $r_0$ to set the scale, we shall often give both the dimensionless number $r_0 \Lambda _{\overline{\mathrm{MS}}}$, as well as $\Lambda _{\overline{\mathrm{MS}}}$. In case $r_1 \Lambda _{\overline{\mathrm{MS}}}$ is given in the publications, we use $r_0 /r_1 = 1.508$ [476] to convert, neglecting the error on this ratio.

The attentive reader will have noticed that bounds such as $\mu a < 1.5$ and $\alpha _\mathrm{eff}<0.25$ which we require for  are not very stringent. There is a considerable difference between  and . We have chosen the above bounds since not too many computations would satisfy more stringent ones at present. Nevertheless, we believe that the  criteria already give reasonable bases for estimates of systematic errors. In the future, we expect that we will be able to tighten our criteria for inclusion in the average, and that many more computations will reach the present  rating in one or more categories.

In principle one should also account for electroweak radiative corrections. However, both in the determination of $\alpha _\mathrm{s}$ at intermediate scales $\mu$ and in the running to high scales, we expect electroweak effects to be much smaller than the presently reached precision. Such effects are therefore not further discussed.

### $\alpha _\mathrm{s}$ from the Schrödinger functional

#### General considerations

The method of step-scaling functions avoids the scale problem, Eq. (172). It is in principle independent of the particular boundary conditions used and was first developed with periodic boundary conditions in a two-dimensional model [477]. However, at present all applications in QCD use Schrödinger functional boundary conditions [87, 478]. An important reason is that these boundary conditions avoid zero modes for the quark fields and quartic modes [479] in the perturbative expansion in the gauge fields. Furthermore the corresponding renormalisation scheme is well studied in perturbation theory [480–482] with the three-loop $\beta$-function and two-loop cutoff effects (for the standard Wilson regularisation) known.

Let us first briefly review the step-scaling strategy. The essential idea is to split the determination of the running coupling at large $\mu$ and of a hadronic scale into two lattice calculations and connect them by ‘step scaling’. In the former part, we determine the running coupling constant in a finite-volume scheme, in practice a ‘Schrödinger Functional (SF) scheme’ in which the renormalisation scale is set by the inverse lattice size $\mu = 1/L$. In this calculation, one takes a high renormalisation scale while keeping the lattice spacing sufficiently small as

179 $$\begin{aligned} \mu \equiv 1/L \sim 10\,\ldots \, 100\,\hbox {GeV}, \quad a/L \ll 1. \end{aligned}$$

In the latter part, one chooses a certain $\bar{g}^2_\mathrm{max}=\bar{g}^2(1/L_\mathrm{max})$, typically such that $L_\mathrm{max}$ is around 0.5 $\,{\mathrm {fm}}$. With a common discretisation, one then determines $L_\mathrm{max}/a$ and (in a large volume $L \ge 2$–$3\,\hbox {fm}$) a hadronic scale such as a hadron mass, $\sqrt{t_0}/a$ or $r_0/a$ at the same bare parameters. In this way one gets numbers for $L_\mathrm{max}/r_0$ and by changing the lattice spacing $a$ carries out a continuum limit extrapolation of that ratio.

In order to connect $\bar{g}^2(1/L_\mathrm{max})$ to $\bar{g}^2(\mu )$ at high $\mu$, one determines the change of the coupling in the continuum limit when the scale changes from $L$ to $L/2$, starting from $L=L_\mathrm{max}$ and arriving at $\mu = 2^k /L_\mathrm{max}$. This part of the strategy is called step scaling. Combining these results yields $\bar{g}^2(\mu )$ at $\mu = 2^k {r_0 \over L_\mathrm{max}} r_0^{-1}$, where $r_0$ stands for the particular chosen hadronic scale.

In order to have a perturbatively well-defined scheme, the SF scheme uses Dirichlet boundary condition at time $t = 0$ and $t = T$. These break translation invariance and permit ${O}(a)$ counter terms at the boundary through quantum corrections. Therefore, the leading discretisation error is ${O}(a)$. In practice, improving the lattice action is achieved by adding one-loop or two-loop perturbative counter terms at the boundaries whose coefficients are denoted as $c_{t},{\tilde{c}}_{t}$. A better precision in this step yields a better control over discretisation errors, which is important, as can be seen, e.g., in [483, 484]. The finite $c^{(i)}_g$, Eq. (167), are known for $i=1,2$ [481, 482].

#### Discussion of computations

In Table 32 we give results from various determinations of the $\Lambda$-parameter. For a clear assessment of the $N_\mathrm{f}$ dependence, the last column also shows results that refer to a common hadronic scale, $r_0$. As discussed above, the renormalisation scale can be chosen large enough such that $\alpha _\mathrm{s} < 0.2$ and the perturbative behaviour can be verified. Consequently only  is present for these criteria. With dynamical fermions, results for the step-scaling functions are always available for at least $a/L = \mu a =1/4,1/6, 1/8$. All calculations have a non-perturbatively ${\mathrm {O}}(a)$ improved action in the bulk. For the discussed boundary ${\mathrm {O}}(a)$ terms this is not so. In most recent calculations two-loop ${\mathrm {O}}(a)$ improvement is employed together with at least three lattice spacings.^36^ This means a  for the continuum extrapolation. In the other cases only one-loop $c_{t}$ was available and we arrive at . We note that the discretisation errors in the step-scaling functions are usually found to be very small, at the percent level or below. However, the overall desired precision is very high as well, and the results in CP-PACS 04 [483] show that discretisation errors at the below percent level cannot be taken for granted. In particular with staggered fermions (unimproved except for boundary terms) few percent effects are seen in Perez 10 [486].

**Table 32:** Results for the $\Lambda$-parameter from computations using step scaling of the SF-coupling. Entries without values for $\Lambda$ computed the running and established perturbative behaviour at large $\mu$

Collaboration	Ref.	$N_\mathrm{f}$	Publication status	Renormalisation scale	Perturbative behaviour	Continuum extrapolation	Scale	$\Lambda _{\overline{\mathrm{MS}}}[\,{\mathrm {MeV}}]$	$r_0\Lambda _{\overline{\mathrm{MS}}}$
ALPHA 10A	[485]	4	A				Only running of $\alpha _\mathrm{s}$ in Fig. 4		
Perez 10	[486]	4	P				Only step-scaling function in Fig. 4		
PACS-CS 09A	[487]	$2+1$	A				$m_\rho$	$371\,(13)(8)(^{+0}_{-27}) ^{\#}$	$0.888\,(30)(18)(^{+0}_{-65})^\dagger$
			A				$m_\rho$	$345\,(59) ^{\#\#}$	$0.824\,(141) ^\dagger$
ALPHA 12$^*$	[59]	2	A				$f_{K}$	$310\,(20)$	$0.789\,(52)$
ALPHA 04	[488]	2	A				$r_0$ ^§^	$245\,(16)(16)$ ^§^	$0.62\,(2)(2)$ ^§^
ALPHA 01A	[489]	2	A				Only running of $\alpha _\mathrm{s}$ in Fig. 5		
CP-PACS 04^&^	[483]	0	A				Only tables of $g^2_\mathrm{SF}$		
ALPHA 98$^{\dagger \dagger }$	[490]	0	A				$r_0=0.5\,{\mathrm {fm}}$	$238(19)$	0.602 (48)
Lüscher 93	[480]	0	A				$r_0=0.5\,{\mathrm {fm}}$	233 (23)	0.590 (60)^§§^

$^{\#}$ Result with a constant (in $a$) continuum extrapolation of the combination $L_{\mathrm{max}}m_\rho$

${}^\dagger$ In conversion to $r_0\Lambda _{\overline{\mathrm{MS}}}$, $r_0$ is taken to be $0.472\,\hbox {fm}$

${}^{\#\#}$ Result with a linear continuum extrapolation in $a$ of the combination $L_\mathrm{max}m_\rho$

$${}^{*}$$ Supersedes ALPHA 04

^§^ The $N_\mathrm{f}=2$ results were based on values for $r_0/a$ which have later been found to be too small by [59]. The effect will be of the order of 10–15 %, presumably an increase in $\Lambda r_0$. We have taken this into account by a  in the renormalisation scale

^&^ This investigation was a precursor for PACS-CS 09A and confirmed two step-scaling functions as well as the scale setting of ALPHA 98

$^{\dagger \dagger }$ Uses data of Lüscher 93 and therefore supersedes it

^§§^ Converted from $$\alpha _{{\overline{\mathrm{MS}}}}(37r_0^{-1})=0.1108(25)$$

In the work by PACS-CS 09A [487], the continuum extrapolation in the scale setting is performed using a constant function in $a$ and with a linear function. Potentially the former leaves a considerable residual discretisation error. We here use, as discussed with the collaboration, the continuum extrapolation linear in $a$, as given in the second line of PACS-CS 09A results in Table 32.

A single computation, PACS-CS 09A [487], quotes also $\alpha _{\overline{\mathrm{MS}}}(M_Z)$. We take the linear continuum extrapolation as discussed above:

180 $$\begin{aligned} \alpha _{\overline{\mathrm{MS}}}^{(5)}(M_Z)=0.118(3), \end{aligned}$$

where the conversion from a three-flavour result to five-flavours was done perturbatively (see Sect. 9.2). Other results do not have a sufficient number of quark flavours (ALPHA 10A [485], Perez 10 [486]) or do not yet contain the conversion of the scale to physical units. Thus no value for $\alpha _{\overline{\mathrm{MS}}}^{(5)}(M_Z)$ is quoted.

More results for $\alpha _{\overline{\mathrm{MS}}}^{(5)}(M_Z)$ using step-scaling functions can be expected soon. Their precision is likely to be much better than what we were able to report on here. A major reason is the use of the gradient flow [183] in the definitions of finite-volume schemes [491, 492].

### $\alpha _\mathrm{s}$ from the potential at short distances

#### General considerations

The basic method was introduced in [493] and developed in [494]. The force or potential between an infinitely massive quark and antiquark pair defines an effective coupling constant via

181 $$\begin{aligned} F(r) = {\hbox {d} V(r) \over \hbox {d}r}= C_F {\alpha _\mathrm{qq}(r) \over r^2}. \end{aligned}$$

The coupling can be evaluated non-perturbatively from the potential through a numerical differentiation; see below. In perturbation theory one also defines couplings in different schemes $\alpha _{\bar{V}}$, $\alpha _V$ via

182 $$\begin{aligned} V(r) = - C_F {\alpha _{\bar{V}}(r) \over r},\quad \hbox {or} \quad \tilde{V}(Q) = - C_F {\alpha _V(Q) \over Q^2}, \end{aligned}$$

where one fixes the unphysical constant in the potential by $\lim _{r\rightarrow \infty }V(r)=0$ and $\tilde{V}(Q)$ is the Fourier transform of $V(r)$. Non-perturbatively, the subtraction of a constant in the potential introduces an additional renormalisation constant, the value of $V(r_\mathrm{ref})$ at some distance $r_\mathrm{ref}$. Perturbatively, it entails a renormalon ambiguity. In perturbation theory, these definitions are all simply related to each other, and their perturbative expansions are known including the $\alpha _\mathrm{s}^4$ and $\alpha _\mathrm{s}^4 \log \alpha _\mathrm{s}$ terms [495–502].

The potential $V(r)$ is determined from ratios of Wilson loops, $W(r,t)$, which behave as

183 $$\begin{aligned} {\langle }W(r, t) {\rangle }= |c_0|^2 e^{-V(r)t} + \sum _{n\not = 0} |c_n|^2 e^{-V_n(r)t}, \end{aligned}$$

where $t$ is taken as the temporal extension of the loop, $r$ is the spatial one and $V_n$ are excited-state potentials. To improve the overlap with the ground state, and to suppress the effects of excited states, $t$ is taken large. Also various additional techniques are used, such as a variational basis of operators (spatial paths) to help in projecting out the ground state. Furthermore some lattice-discretisation effects can be reduced by averaging over Wilson loops related by rotational symmetry in the continuum.

In order to reduce discretisation errors it is of advantage to define the numerical derivative giving the force as

184 $$\begin{aligned} F(r_{I}) = { V(r) - V(r-a) \over a }, \end{aligned}$$

where $r_{I}$ is chosen so that at tree level the force is the continuum force. $F(r_{I})$ is then a ‘tree-level improved’ quantity and similarly the tree-level improved potential can be defined [503].

Finally, as was noted in Sect. 9.2, a determination of the force can also be used to determine the $r_0$ scale, by defining it from the static force by

185 $$\begin{aligned} r_0^2 F(r_0) = {1.65}. \end{aligned}$$

#### Discussion of computations

In Table 33, we list results of determinations of $r_0\Lambda _{{\overline{\mathrm{MS}}}}$ (together with $\Lambda _{{\overline{\mathrm{MS}}}}$ using the scale determination of the authors).

**Table 33:** Short-distance potential results

Collaboration	Ref.	$N_\mathrm{f}$	Publication status	Renormalisation scale	Perturbative behaviour	Continuum extrapolation	Scale	$\Lambda _{\overline{\mathrm{MS}}}\,(\,{\mathrm {MeV}})$	$r_0\Lambda _{\overline{\mathrm{MS}}}$
Bazavov 12	[504]	$$2+1$$	A	$$^\dagger $$		$$^\#$$	$$r_0 = 0.468\,\hbox {fm}$$	$$295\, (30)^\star $$	$$0.70\, (7)^{**}$$
ETM 11C	[505]	2	A				$r_0 = 0.42\,\hbox {fm}$	315 (30)^§^	$0.658\,(55)$
Brambilla 10	[506]	0	A			$^{\dagger \dagger }$			$0.637\, (^{+32}_{-30}) ^{\dagger \dagger +}$
UKQCD 92	[494]	0	A		$^{++}$		$\sqrt{\sigma }=0.44\,\,{\mathrm {GeV}}$	256 (20)	0.686 (54)
Bali 92	[507]	0	A		$^{++}$		$\sqrt{\sigma }=0.44\,\,{\mathrm {GeV}}$	247 (10)	0.661 (27)

$^\dagger$ Since values of $\alpha _{\mathrm {eff}}$ within our designated range are used, we assign a  despite values of $\alpha _\mathrm{eff}$ up to $\alpha _{\mathrm {eff}}=0.5$ being used

$^\#$ Since values of $2a/r$ within our designated range are used, we assign a  although only values of $2a/r\ge 1.14$ are used at $\alpha _{\mathrm {eff}}=0.3$

$^\star$ Using results from [476]

$$^{\star \star }$$ $$\alpha ^{(3)}_{\overline{\mathrm{MS}}}(1.5\,\hbox {GeV}) = 0.326(19)$$, $$\alpha ^{(5)}_{\overline{\mathrm{MS}}}(M_Z) = 0.1156(^{+21}_{-22})$$

^§^ Both potential and $r_0/a$ are determined on a small ($L=3.2r_0$) lattice

${}^{\dagger \dagger }$ Uses lattice results of [484], some of which have very small lattice spacings where according to more recent investigations a bias due to the freezing of topology may be present

$^+$ Only $r_0\Lambda _{\overline{\mathrm{MS}}}$ is given

$^{++}$ We give a  because only a NLO formula is used and the error bars are very large; our criterion does not apply well to these very early calculations

The first determinations in the three-colour Yang Mills theory are by UKQCD 92 [494] and Bali 92 [507], who used $\alpha _\mathrm{qq}$ as explained above, but not in the tree-level improved form. Rather a phenomenologically determined lattice artefact correction was subtracted from the lattice potentials. The comparison with perturbation theory was on a more qualitative level on the basis of a two-loop formula and a continuum extrapolation could not be performed as yet. A much more precise computation of $\alpha _\mathrm{qq}$ with continuum extrapolation was performed in [484, 503]. Satisfactory agreement with perturbation theory was found [503] but the stability of the perturbative prediction was not considered sufficient to be able to extract a $\Lambda$-parameter.

In Brambilla 10 [506] the same quenched lattice results of [503] were used and a fit was performed to the continuum potential, instead of the force, using three-loop perturbation theory with the $\alpha _\mathrm{s}^4 \ln \alpha _\mathrm{s}$ term. Close agreement with perturbation theory was found when a renormalon subtraction was performed. Note that the renormalon subtraction introduces a second scale into the perturbative formula which is absent when the force is considered.

For the quenched calculation very small lattice spacings were available. For both ETM 11C [505] and Bazavov 12 [504] using dynamical fermions such small lattice spacings are not yet realised. They use the tree-level improved potential as described above. We note that the value of $\Lambda _{\overline{\mathrm{MS}}}$ in physical units by ETM 11C [505] is based on a value of $r_0=0.42$ fm. This is at least 10 % smaller than the large majority of other values of $r_0$. Also the value of $r_0/a$ on the finest lattice in that computation comes from a rather small lattice with $L\approx 3.2r_0 \approx 2.4/m_\pi$.

One of the main issues for all these computations is whether the perturbative running of the coupling constant has been reached. While for quenched or $N_\mathrm{f}=0$ fermions this seems to be the case at the smallest distances, for dynamical fermions at present there is no consensus. While both Brambilla 10 [506] and Bazavov 12 [504] find good agreement with perturbation theory after the renormalon is subtracted, Ref. [508] uses the force, where no renormalon contributes, and finds that far shorter distances are needed than are presently accessible for dynamical fermion simulations in order to match to perturbation theory. Further work is needed to clarify this point.

### $\alpha _\mathrm{s}$ from the vacuum polarisation at short distances

#### General considerations

The vacuum polarisation function for the flavour non-singlet currents $J^a_\mu$ ($a=1,2,3$) in the momentum representation is parameterised as

186 $$\begin{aligned} {\langle }J^a_\mu J^b_\nu \rangle&= \delta ^{ab} [(\delta _{\mu \nu }Q^2 - Q_\mu Q_\nu ) \Pi ^{(1)}(Q)\nonumber \\&- Q_\mu Q_\nu \Pi ^{(0)}(Q)], \end{aligned}$$

where $Q_\mu$ is a space like momentum and $J_\mu \equiv V_\mu$ for a vector current and $J_\mu \equiv A_\mu$ for an axial-vector current. Defining $\Pi _J(Q)\equiv \Pi _J^{(0)}(Q)+\Pi _J^{(1)}(Q)$, the operator product expansion (OPE) of the vacuum polarisation function $\Pi _{V+A}(Q)=\Pi _V(Q)+\Pi _A(Q)$ is given by

187 $$\begin{aligned}&\Pi _{V+A}|_\mathrm{OPE}(Q^2,\alpha _\mathrm{s})\nonumber \\&\quad = c + C_1(Q^2) + C_{m}^{V+A}(Q^2)\frac{\bar{m}^2(Q)}{Q^2}\nonumber \\&\qquad + \sum _{q=u,d,s}C_{\bar{q}q}^{V+A}(Q^2)\frac{{\langle }m_Q\bar{q}q \rangle }{Q^4}\nonumber \\&\qquad + C_{GG}(Q^2)\frac{{\langle }\alpha _\mathrm{s} GG\rangle }{Q^4}+{O}(Q^{-6}), \end{aligned}$$

for large $Q^2$. $C_X^{V+A}(Q^2)=\sum _{i\ge 0}(C_X^{V+A})^{(i)}\alpha ^i(Q^2)$ are the perturbative coefficient functions for the operators $X$ ($X=1$, $\bar{q}q$, $GG$). Here $C_1$ is known up to four- loop order in a continuum renormalisation scheme such as the $\overline{\mathrm{MS}}$ scheme [509, 510]. Non-perturbatively, there are terms in $C_X$ which do not have a series expansion in $\alpha _\mathrm{s}$. For an example for the unit operator see [511]. The term $c$ is $Q$–independent and divergent in the limit of infinite ultraviolet cutoff. However the Adler function defined as

188 $$\begin{aligned} D(Q^2) \equiv - Q^2 { \hbox {d}\Pi (Q^2) \over \hbox {d}Q^2}, \end{aligned}$$

is a scheme independent finite quantity. Therefore one can determine the running coupling constant in the $\overline{\mathrm{MS}}$ scheme from the vacuum polarisation function computed by a lattice QCD simulation.

In more detail, the lattice data of the vacuum polarisation are fitted with the perturbative formula (187) with fit parameter $\Lambda _{\overline{\mathrm{MS}}}$ parameterising the running coupling $\alpha _{\overline{\mathrm{MS}}}(Q^2)$.

While there is no problem in discussing the OPE at the non-perturbative level, the ‘condensates’ such as ${{\langle }\alpha _\mathrm{s} GG\rangle }$ are ambiguous, since they mix with lower-dimensional operators including the unity operator. Therefore one should work in the high $Q^2$ regime where power corrections are negligible within the given accuracy. Thus setting the renormalisation scale as $\mu \equiv \sqrt{Q^2}$, one should seek, as always, the window $\Lambda _\mathrm{QCD} \ll \mu \ll a^{-1}$.

#### Discussion of computations

Results using this method are, to date, only available using overlap fermions. These are collected in Table 34 for $N_\mathrm{f}=2$, JLQCD/TWQCD 08C [513] and for $N_\mathrm{f} = 2+1$, JLQCD 10 [512]. At present, only one lattice spacing $a \approx 0.11\,\hbox {fm}$ has been simulated.

**Table 34:** Vacuum polarisation results

Collaboration	Ref.	$N_\mathrm{f}$	Publication status	Renormalisation scale	Perturbative behaviour	Continuum extrapolation	Scale	$\Lambda _{\overline{\mathrm{MS}}}\,(\,{\mathrm {MeV}})$	$r_0\Lambda _{\overline{\mathrm{MS}}}$
JLQCD 10	[512]	$2+1$	A				$r_0 = 0.472\,\hbox {fm}$	$247\, (5) ^\dagger$	$0.591\, (12)$
JLQCD/TWQCD 08C	[513]	2	A				$r_0 = 0.49\,\hbox {fm}$	$234\, (9) (^{+16}_{-0})$	$0.581\, (22) (^{+40}_{-0})$

$^\dagger$ The paper cites $\alpha _{\overline{\mathrm{MS}}}^{(5)}(M_Z)=0.1181(3)(^{+14}_{-12})$. As a result of an inconsistency found in this estimate by the FLAG working group, the number will be revised by JLQCD

The fit to Eq. (187) is done with the four-loop relation between the running coupling and $\Lambda _{{\overline{\mathrm{MS}}}}$. It is found that without introducing condensate contributions, the momentum scale where the perturbative formula gives good agreement with the lattice results is very narrow, $aQ \simeq 0.8$–1.0. When condensate contributions are included the perturbative formula gives good agreement with the lattice results for the extended range $aQ \simeq 0.6$–1.0. Since there is only a single lattice spacing there is a  for the continuum limit. The renormalisation scale $\mu$ is in the range of $Q=1.6$–2 GeV. Choosing $\alpha _\mathrm{eff}=\alpha _{\overline{\mathrm{MS}}}(Q)$, we find that $\alpha _\mathrm{eff}=0.25-0.30$ for $N_\mathrm{f}=2$ and $\alpha _\mathrm{eff}=0.29-0.33$ for $N_\mathrm{f}=2+1$. Thus we give a  and  for $N_\mathrm{f}=2$ and $N_\mathrm{f}=2+1$ respectively for the renormalisation scale and a  for the perturbative behaviour.

### $\alpha _\mathrm{s}$ from observables at the lattice spacing scale

#### General considerations

The general method is to evaluate a short-distance quantity ${\mathcal {O}}$ at the scale of the lattice spacing $\sim$1/$a$ and then determine its relationship to $\alpha _{\overline{\mathrm{MS}}}$ via a power series expansion.

This is epitomised by the strategy of the HPQCD Collaboration [514, 515], discussed here for illustration, which computes and then fits to a variety of short-distance quantities, $Y$,

189 $$\begin{aligned} Y = \sum _{n=1}^{n_\mathrm{max}} c_n \alpha _{\mathrm {V'}}^n(q^*). \end{aligned}$$

$Y$ is taken as the logarithm of small Wilson loops (including some non-planar ones), Creutz ratios, ‘tadpole-improved’ Wilson loops and the tadpole-improved or ‘boosted’ bare coupling ($O(20)$ quantities in total). $c_n$ are perturbative coefficients (each depending on the choice of $Y$) known to $n = 3$ with additional coefficients up to $n_\mathrm{max}$ being numerically fitted. $\alpha _{\mathrm {V'}}$ is the running coupling constant related to $\alpha _{\mathrm {V}}$ from the static quark potential (see Sect. 9.4.1).^37^

The coupling constant is fixed at a scale $q^* = d/a$. This is chosen as the mean value of $\ln q$ with the one gluon loop as measure, [516, 517]. (Thus a different result for $d$ is found for every short-distance quantity.) A rough estimate yields $d \approx \pi$, and in general the renormalisation scale is always found to lie in this region.

For example for the Wilson loop $W_{mn} \equiv {\langle }W(ma,na) \rangle$ we have

190 $$\begin{aligned} \ln \left( \frac{W_{mn}}{u_0^{2(m+n)}}\right)&= c_1 \alpha _{\mathrm {V'}}(q^*) + c_2 \alpha _{\mathrm {V'}}^2(q^*)\nonumber \\&+ c_3 \alpha _{\mathrm {V'}}^3(q^*)+ \cdots , \end{aligned}$$

for the tadpole-improved version, where $c_1$, $c_2, \ldots$ are the appropriate perturbative coefficients and $u_0 = W_{11}^{1/4}$. Substituting the non-perturbative simulation value in the left hand side, we can determine $\alpha _{\mathrm {V'}}(q^*)$, at the scale $q^*$. Note that one finds empirically that perturbation theory for these tadpole-improved quantities have smaller $c_n$ coefficients and so the series has a faster apparent convergence.

Using the $\beta$ function in the $V'$-scheme, results can be run to a reference value, chosen as $\alpha _0 \equiv \alpha _{\mathrm {V'}}(q_0)$, $q_0 = 7.5\,\hbox {GeV}$. This is then converted perturbatively to the continuum ${\overline{\mathrm{MS}}}$ scheme

191 $$\begin{aligned} \alpha _{\overline{\mathrm{MS}}}(q_0)= \alpha _0 + d_1 \alpha _0^2 + d_2 \alpha _0^3 + \cdots , \end{aligned}$$

where $d_1, d_2$ are known one and two loop coefficients.

Other collaborations have focussed more on the bare ‘boosted’ coupling constant and directly determined its relationship to $\alpha _{\overline{\mathrm{MS}}}$. Specifically, the boosted coupling is defined by

192 $$\begin{aligned} \alpha _{\mathrm {P}}(1/a) = {1\over 4\pi } {g_0^2 \over u_0^4}, \end{aligned}$$

again determined at a scale $\sim$1/$a$. As discussed previously since the plaquette expectation value in the boosted coupling contains the tadpole diagram contributions to all orders, which are dominant contributions in perturbation theory, there is an expectation that the perturbation theory using the boosted coupling has smaller perturbative coefficients [516], and hence smaller perturbative errors.

#### Continuum limit

Lattice results always come along with discretisation errors, which one needs to remove by a continuum extrapolation. As mentioned previously, in this respect the present method differs in principle from those in which $\alpha _\mathrm{s}$ is determined from physical observables. In the general case, the numerical results of the lattice simulations at a value of $\mu$ fixed in physical units can be extrapolated to the continuum limit, and the result can be analysed as to whether it shows perturbative running as a function of $\mu$ in the continuum. For observables at the cutoff-scale ($q^*=d/a$), discretisation effects cannot easily be separated out from perturbation theory, as the scale for the coupling comes from the lattice spacing. Therefore the restriction $a\mu \ll 1$ (the ‘continuum-extrapolation’ criterion) is not applicable here. Discretisation errors of order $a^2$ are, however, present. Since $a\sim \exp (-1/(2b_0 g_0^2)) \sim \exp (-1/(8\pi b_0 \alpha (q^*))$, these errors now appear as power corrections to the perturbative running, and have to be taken into account in the study of the perturbative behaviour, which is to be verified by changing $a$. One thus always should fit with power corrections in this method.

In order to keep a symmetry with the ‘continuum-extrapolation’ criterion for physical observables and to remember that discretisation errors are, of course, relevant, we replace it here by one for the lattice spacings used:

$$\bullet $$ Lattice spacings

three or more lattice spacings, at least two points below $a = 0.1$ fm
two lattice spacings, at least one point below $a = 0.1$ fm
otherwise

#### Discussion of computations

Note that due to $\mu \sim 1/a$ being relatively large the results easily have a  or  in the rating on renormalisation scale.

The work of El-Khadra 92 [524] employs a one-loop formula to relate $\alpha ^{(0)}_{\overline{\mathrm{MS}}}(\pi /a)$ to the boosted coupling for three lattice spacings $a^{-1} = 1.15$, 1.78, 2.43 GeV. (The lattice spacing is determined from the charmonium $1S$–$1P$ splitting.) They obtain $\Lambda ^{(0)}_{\overline{\mathrm{MS}}}=234\,\hbox {MeV}$, corresponding to $\alpha _\mathrm{eff} = \alpha ^{(0)}_{\overline{\mathrm{MS}}}(\pi /a) \approx 0.15$–$0.2$. The work of Aoki 94 [523] calculates $\alpha ^{(2)}_V$ and $\alpha ^{(2)}_{\overline{\mathrm{MS}}}$ for a single lattice spacing $a^{-1}\sim 2\,\hbox {GeV}$ again determined from charmonium $1S$–$1P$ splitting in two-flavour QCD. Using one-loop perturbation theory with boosted coupling, they obtain $\alpha ^{(2)}_V=0.169$ and $\alpha ^{(2)}_{\overline{\mathrm{MS}}}=0.142$. Davies 94 [522] gives a determination of $\alpha _{\mathrm {V}}$ from the expansion

193 $$\begin{aligned} -\ln W_{11}&\equiv \frac{4\pi }{3}\alpha _{\mathrm {V}}^{(N_\mathrm{f})}(3.41/a)\nonumber \\&\times [1 - (1.185+0.070N_\mathrm{f})\alpha _{\mathrm {V}}^{(N_\mathrm{f})} ], \end{aligned}$$

neglecting higher-order terms. They compute the $\Upsilon$ spectrum in $N_\mathrm{f}=0$, 2 QCD for single lattice spacings at $a^{-1} = 2.57$, $2.47\,\hbox {GeV}$ and obtain $\alpha _{\mathrm {V}}(3.41/a)\simeq 0.1$5, 0.18 respectively. Extrapolating the inverse coupling linearly in $N_\mathrm{f}$, a value of $\alpha _{\mathrm {V}}^{(3)}(8.3\,\hbox {GeV}) = 0.196(3)$ is obtained. SESAM 99 [520] follows a similar strategy, again for a single lattice spacing. They linearly extrapolated results for $1/\alpha _{\mathrm {V}}^{(0)}$, $1/\alpha _{\mathrm {V}}^{(2)}$ at a fixed scale of $9\,\hbox {GeV}$ to give $\alpha _{\mathrm {V}}^{(3)}$, which is then perturbatively converted to $\alpha _{\overline{\mathrm{MS}}}^{(3)}$. This finally gave $\alpha _{\overline{\mathrm{MS}}}^{(5)}(M_Z) = 0.1118(17)$. Wingate 95 [521] also follow this method. With the scale determined from the charmonium $1S$–$1P$ splitting for single lattice spacings in $N_\mathrm{f} = 0$, $2$ giving $a^{-1}\simeq 1.80\,\hbox {GeV}$ for $N_\mathrm{f}=0$ and $a^{-1}\simeq 1.66\,\hbox {GeV}$ for $N_\mathrm{f}=2$ they obtain $\alpha _{\mathrm {V}}^{(0)}(3.41/a)\simeq 0.15$ and $\alpha _{\mathrm {V}}^{(2)}\simeq 0.18$ respectively. Extrapolating the coupling linearly in $N_\mathrm{f}$, they obtain $\alpha _{\mathrm {V}}^{(3)}(6.48\,\hbox {GeV})=0.194(17)$.

The QCDSF/UKQCD Collaborations, QCDSF/UKQCD [519, 525–527], use the two-loop relation (re-written here in terms of $\alpha$)

194 $$\begin{aligned} {1 \over \alpha _{\overline{\mathrm{MS}}}(\mu )}&= {1 \over \alpha _{\mathrm {P}}(1/a)} + 4\pi (2b_0\ln a\mu - t_1^P)\nonumber \\&+ (4\pi )^2(2b_1\ln a\mu - t_2^P)\alpha _{\mathrm {P}}(1/a), \end{aligned}$$

where $t_1^P$ and $t_2^P$ are known. (A two-loop relation corresponds to a three-loop lattice beta function.) This was used to directly compute $\alpha _\mathrm{\overline{\mathrm{MS}}}$, and the scale was chosen so that the $O(\alpha ^0_P)$ term vanishes, i.e.

195 $$\begin{aligned} \mu ^* = {1 \over a} \exp {[t_1^P/(2b_0)] } \approx \left\{ \begin{array}{c@{\quad }c} 2.63/a &{} N_\mathrm{f} = 0 \\ 1.4/a &{} N_\mathrm{f} = 2 \\ \end{array} \right. \end{aligned}$$

The method is to first compute $$\alpha _{\mathrm {P}}(1/a)$$ and from this using Eq. (194) to find $$\alpha _{\overline{\mathrm{MS}}}(\mu ^*)$$. The RG equation, Eq. (166), then determines $$\mu ^*/\Lambda _{\overline{\mathrm{MS}}}$$ and hence using Eq. (195) leads to the result for $$r_0\Lambda _{\overline{\mathrm{MS}}}$$. This avoids giving the scale in $$\hbox {MeV}$$ until the end. In the $$N_\mathrm{f}=0$$ case $$7$$ lattice spacings were used [484], giving a range $$\mu ^*/\Lambda _{\overline{\mathrm{MS}}} \approx 24$$–72 (or $$a^{-1} \approx 2$$–$$7\,\hbox {GeV}$$) and $$\alpha _\mathrm{eff} = \alpha _{\overline{\mathrm{MS}}}(\mu ^*) \approx 0.14$$–$$ 0.11$$. Neglecting higher-order perturbative terms (see discussion after Eq. (196) below) in Eq. (194) this is sufficient to allow a continuum extrapolation of $$r_0\Lambda _{\overline{\mathrm{MS}}}$$. A similar computation for $$N_\mathrm{f} = 2$$ by QCDSF/UKQCD 05 [519] gave $$\mu ^*/\Lambda _{\overline{\mathrm{MS}}} \approx 12$$–$$17$$ (or roughly $$a^{-1} \approx 2$$–$$3\,\hbox {GeV}$$) and $$\alpha _\mathrm{eff} = \alpha _{\overline{\mathrm{MS}}}(\mu ^*) \approx 0.20$$–$$ 0.18$$.

The $N_\mathrm{f}=2$ results of QCDSF/UKQCD 05 are affected by an uncertainty which was not known at the time of publication: It has been realised that the values of $r_0/a$ of [519] were significantly too low [59]. As this effect is expected to depend on $a$, it influences the perturbative behaviour leading us to assign a  for that criterion.

The work of HPQCD 05A [514] (which supersedes the original work [528]) uses three lattice spacings $a^{-1} \approx 1.2$, 1.6, $2.3\,\hbox {GeV}$ for $2+1$ flavour QCD. Typically the renormalisation scale $q \approx \pi /a \approx 3.50 - 7.10\,\hbox {GeV}$, corresponding to $\alpha _\mathrm{eff} \equiv \alpha _{V'} \approx 0.22$–0.28.

In the later update HPQCD 08A [515] twelve data sets (with six lattice spacings) are now used reaching up to $a^{-1} \approx 4.4\,\hbox {GeV}$ corresponding to $\alpha _\mathrm{eff} \approx 0.18$. The values used for the scale $r_1$ were further updated in HPQCD 10 [73]. Maltman 08 [518] uses most of the same lattice ensembles as HPQCD 08A [515] but considers a much smaller set of quantities (3 versus 22) that are less sensitive to condensates. They also use different strategies for evaluating the condensates and for the perturbative expansion, and a slightly different value for the scale $r_1$. The central values of the final results from Maltman 08 and HPQCD 08A differ by 0.0009 (which would be decreased to 0.0007 taking into account a reduction of 0.0002 in the value of the $r_1$ scale used by Maltman 08).

As mentioned before, the perturbative coefficients are computed through three-loop order [529], while the higher-order perturbative coefficients $c_n$ with $n_\mathrm{max} \ge n > 3$ (with $n_\mathrm{max} = 10$) are numerically fitted using the lattice simulation data for the lattice spacings with the help of Bayesian methods. It turns out that corrections in Eq. (190) are of order $|c_i/c_1|\alpha ^i=$ 20 %, 5–15 % and 3–10 % for $i=1,2,3$, respectively. The inclusion of a fourth-order term is necessary to obtain a good fit to the data, and leads to a shift of the result by 1–2 sigma. For all but one of the 22 quantities, central values of $\approx$2–4 were found, with errors from the fits of $\approx$2.

For many of the quantities, the fitted central values of the ratios $|c_4/c_1|$ appear to be larger than corresponding lower-order ratios (which would be worrying for the application of perturbation theory), but the coefficients $|c_5/c_1|$ are essentially undetermined by the data and the errors on $|c_4/c_1|$ are sufficiently large that it is premature to decide this issue.

Perturbative truncation errors are the largest source of uncertainty in HPQCD 08A/10A, and a significant contribution in Maltman; both estimate this error to be about 0.3–0.4 %. Maltman uses the changes observed from fitting to data at the three finest versus fitting to data at all lattice spacings, while HPQCD uses the (correlated) errors in their fitted coefficients $c_4$ and $c_5$. As discussed in the introduction and conclusions, however, perturbative truncation errors are notoriously difficult to estimate. In the concluding section (Sect. 9.9), we therefore also consider a more conservative power-counting estimate of the perturbative error, taking the estimated size of the $c_4$ term as the uncertainty. With $\alpha _1=\alpha _{\overline{\mathrm{MS}}}^{(3)}(5\,\,{\mathrm {GeV}})$ and $\alpha _2=\alpha _{\overline{\mathrm{MS}}}^{(5)}(M_Z)$ we have

196 $$\begin{aligned} \Delta \alpha _1 \!=\! \left| {c_4 \over c_1}\right| \alpha _1^4,\quad \Delta \alpha _2 \!=\! \left| {c_4 \over c_1}\right| \alpha _1^2 \alpha _2^2,\quad {\Delta \Lambda \over \Lambda } \!=\! {1\over 8\pi b_0 \alpha _1} { \Delta \alpha _1 \over \alpha _1}. \nonumber \\ \end{aligned}$$

In order to obtain a numerical value we need $|c_4/c_1|$. It has been estimated as part of the fit by HPQCD. Since the fit results are $|c_4/c_1| = 4\pm 2$ for the (log of the) plaquette and unimproved Wilson loops, the estimated four-loop correction from Eq. (196) is of order 2–6 %.

As perturbative coefficients are fit parameters, it is important to have isolated the perturbative piece of the short-distance quantity, or to show that non-perturbative effects are small. Checks were made expanding the short-distance quantity in a Taylor expansion in the quark mass and adding ‘gluon condensate’-like terms. This did not change the fits perceptibly. With the $\alpha _\mathrm{eff}$ values given above we assign a  for the renormalisation scale. According to our criterion the perturbative behaviour is verified. However, one should keep mind that it was necessary to include fitted higher-order coefficients in order to describe the data. The fact that these fitted coefficients are not well-determined by the data makes the test less stringent. Table 35 summarises the results.

**Table 35:** Wilson loop results

Collaboration	Ref.	$N_\mathrm{f}$	Publication status	Renormalisation scale	Perturbative behaviour	Lattice spacings	Scale	$\Lambda _{\overline{\mathrm{MS}}}\,(\,{\mathrm {MeV}})$	$r_0\Lambda _{\overline{\mathrm{MS}}}$
HPQCD 10$^a$ ^§^	[73]	$2+1$	A				$r_1 = 0.3133(23)\, \hbox {fm}$	340 (9)	0.812 (22)
HPQCD 08A$^a$	[515]	$2+1$	A				$r_1 = 0.321(5)\,\hbox {fm}^{\dagger \dagger }$	338 (12)$^\star$	0.809 (29)
Maltman 08$^a$	[518]	$2+1$	A				$r_1 = 0.318\, \hbox {fm}$	352 (17)$^\dagger$	0.841 (40)
HPQCD 05A$^a$	[514]	$2+1$	A				$r_{1}^{\dagger \dagger }$	319 (17)$^{\star \star }$	0.763 (42)
QCDSF/UKQCD 05	[519]	2	A				$r_0 = 0.467\,(33)\,\hbox {fm}$	261 (17) (26)	0.617 (40) (21)$^b$
SESAM 99$^c$	[520]	2	A				$c\bar{c}$ ($1S-1P$)		
Wingate 95$^d$	[521]	2	A				$c\bar{c}$ ($1S-1P$)		
Davies 94$^e$	[522]	2	A				$\Upsilon$		
Aoki 94$^f$	[523]	2	A				$c\bar{c}$ ($1S-1P$)		
QCDSF/UKQCD 05	[519]	0	A				$r_0 = 0.467\,(33)\,\hbox {fm}$	259 (1) (20)	0.614 (2) (5)$^b$
SESAM 99$^c$	[520]	0	A				$c\bar{c}$ ($1S-1P$)		
Wingate 95$^d$	[521]	0	A				$c\bar{c}$ ($1S-1P$)		
Davies 94$^e$	[522]	0	A				$\Upsilon$		
El-Khadra 92$^g$	[524]	0	A				$c\bar{c}$ ($1S-1P$)	234 (10)	0.593 (25)$^h$

$$^\dagger $$ $$\alpha ^{(5)}_{\overline{\mathrm{MS}}}(M_Z)=0.1192(11)$$

$$^\star \,\alpha _V^{(3)}(7.5\,\hbox {GeV})=0.2120(28)$$, $$\alpha ^{(5)}_{\overline{\mathrm{MS}}}(M_Z)=0.1183(8)$$, supersedes HPQCD 05

^§^ $\alpha _{\overline{\mathrm{MS}}}^{(3)}(5\ \hbox {GeV})=0.2034(21)$, $\alpha ^{(5)}_{\overline{\mathrm{MS}}}(M_Z)=0.1184(6)$, only update of intermediate scale and $c$, $b$ quark masses, supersedes HPQCD 08A and Maltman 08

$^{\dagger \dagger }$ Scale is originally determined from $\Upsilon$ mass splitting. $r_1$ is used as an intermediate scale. In conversion to $r_0\Lambda _{\overline{\mathrm{MS}}}$, $r_0$ is taken to be $0.472\,\hbox {fm}$

$$^{\star \star }$$ $$\alpha _V^{(3)}(7.5\,\hbox {GeV})=0.2082(40)$$, $$\alpha ^{(5)}_{\overline{\mathrm{MS}}}(M_Z)=0.1170(12)$$

$^a$ The numbers for $\Lambda$ have been converted from the values for $\alpha _\mathrm{s}^{(5)}(M_Z)$

$^b$ This supersedes [525–527]. $\alpha ^{(5)}_{\overline{\mathrm{MS}}}(M_Z)=0.112(1)(2)$. The $N_\mathrm{f}=2$ results were based on values for $r_0 /a$ which have later been found to be too small [59]. The effect will be of the order of 10–15 %, presumably an increase in $\Lambda r_0$

$$^c$$ $$\alpha ^{(5)}_{\overline{\mathrm{MS}}}(M_Z)=0.1118(17)$$

$$^d$$ $$\alpha _V^{(3)}(6.48\,\hbox {GeV})=0.194(7)$$ extrapolated from $$N_\mathrm{f}=0,2$$. $$\alpha ^{(5)}_{\overline{\mathrm{MS}}}(M_Z)=0.107(5)$$

$$^e$$ $$\alpha _P^{(3)}(8.2\,\hbox {GeV})=0.1959(34)$$ extrapolated from $$N_\mathrm{f}=0,2$$. $$\alpha ^{(5)}_{\overline{\mathrm{MS}}}(M_Z)=0.115(2)$$

$$^f$$ Estimated $$\alpha ^{(5)}_{\overline{\mathrm{MS}}}(M_Z)=0.108(5)(4)$$

$^g$ This early computation violates our requirement that scheme conversions are done at the two-loop level

$^h$ Used $r_0=0.5$ fm to convert to $r_0 \Lambda _{{\overline{\mathrm{MS}}}}$. $\Lambda _{\overline{\mathrm{MS}}}^{(4)}=160(^{+47}_{-37})\hbox {MeV}$, $\alpha ^{(4)}_{\overline{\mathrm{MS}}}(5\hbox {GeV})=0.174(12)$. We converted this number to give $\alpha ^{(5)}_{\overline{\mathrm{MS}}}(M_Z)=0.106(4)$

### $\alpha _\mathrm{s}$ from current two-point functions

#### General considerations

The method has been introduced in [85] and updated in [73]; see also [530]. The basic observable is constructed from a current $J(x) = i m_{h}\overline{\psi }_{h}(x)\gamma _5\psi _{h'}(x)$ of two mass-degenerate heavy valence quarks, $h$, $h^\prime$. The pre-factor $m_{h}$ denotes the bare mass of the quark. With a residual chiral symmetry, $J(x)$ is a renormalisation group invariant local field, i.e. it requires no renormalisation. Staggered fermions and twisted mass fermions have such a residual chiral symmetry. The (Euclidean) time-slice correlation function

197 $$\begin{aligned} G(x_0) = a^3 \sum _{\vec {x}} {\langle }J^\dagger (x) J(0) {\rangle }, \end{aligned}$$

($J^\dagger (x) = im_{h}\overline{\psi }_{h'}(x)\gamma _5\psi _{h}(x)$) has a $\sim x_0^{-3}$ singularity at short distances and moments

198 $$\begin{aligned} G_n = a \sum _{t=-(T/2-a)}^{T/2-a} t^n \,G(t), \end{aligned}$$

are finite for $n \ge 4$. Here $T$ is the time extent of the lattice. The moments are dominated by contributions at $t$ of order $1/m_{h}$. For large mass $m_{h}$ these are short distances and the moments become increasingly perturbative for decreasing $n$. Denoting the lowest-order perturbation theory moments by $G_n^{(0)}$, one defines the normalised moments

199 $$\begin{aligned} R_n = \left\{ \begin{array}{cc} G_4/G_4^{(0)} &{} \quad \hbox {for }n=4, \\ {m_\mathrm{p} G_n^{1/(n-4)} \over 2m_{h} (G_n^{(0)})^{1/(n-4)} } &{} \quad \hbox {for }n \ge 6, \\ \end{array} \right. \end{aligned}$$

of even order $n$. The mass, $m_\mathrm{p}$, of the pseudoscalar flavoured $h h'$ state is used to make $G_n$ dimensionless, while in the denominator the bare quark mass is used for this purpose. In the continuum limit the normalised moments can be parameterised in terms of functions

200 $$\begin{aligned} R_n \equiv \left\{ \begin{array}{cc} r_4(\alpha _\mathrm{s}(\mu ), \mu /\bar{m}_{h}(\mu )) &{} \quad \hbox {for }n=4, \\ z\cdot r_n(\alpha _\mathrm{s}(\mu ), \mu /\bar{m}_{h}(\mu )) &{} \quad \hbox {for }n \ge 6, \\ \end{array} \right. \end{aligned}$$

where

201 $$\begin{aligned} z = { m_\mathrm{p} \over 2\bar{m}_{h}(\mu ) }, \end{aligned}$$

with $\bar{m}_{h}(\mu )$ being the renormalised quark mass. The prefactor $z$ parameterises the heavy quark mass and the quantities $r_n$ have a perturbative expansion

202 $$\begin{aligned} r_n = 1 + r_{n,1}\alpha _\mathrm{s} + r_{n,2}\alpha _\mathrm{s}^2 + r_{n,3}\alpha _\mathrm{s}^3 + \cdots , \end{aligned}$$

where the written terms $r_{n,i}(\mu /\bar{m}_{h}(\mu ))$, $i \le 3$ are known for low $n$ from [509, 510, 531–533]. In practice, the expansion is used in the $\overline{\mathrm{MS}}$ scheme. Matching non-perturbative lattice results for the moments to the perturbative expansion, one can determine an approximation to $\alpha _{\overline{\mathrm{MS}}}(\mu )$ as well as $\bar{m}_{h}(\mu )$. With the lattice spacing (scale) determined from some extra physical input, this calibrates $\mu$.

A difficulty with this approach is that large masses are needed to enter the perturbative domain. Lattice artefacts can then be sizeable and have a complicated form. The ratios in Eq. (199) use the tree-level lattice results in the usual way for normalisation. This results in unity as the leading term in Eq. (202), suppressing some of the kinematical lattice artefacts. We note that in contrast to e.g. the definition of $\alpha _\mathrm{qq}$, here the cutoff effects are of order $a^n\alpha _\mathrm{s}$, while there the tree-level term defines $\alpha _\mathrm{s}$ and therefore the cutoff effects after tree-level improvement are of order $a^n\alpha _\mathrm{s}^2$.

Furthermore finite-size effects (FSE) due to the omission of $|t| > T /2$ in Eq. (198) grow with $n$ as $(m_\mathrm{p}T/2)^n \exp {(-m_\mathrm{p} T/2)}$. In practice, however, since the (lower) moments are short-distance dominated, the FSE are expected to be irrelevant at the present level of precision. In the definitions above, the mass of an artificial non-singlet pseudoscalar meson has been used, since this is done in the simulations. In the determinations of the quark masses, this mass is approximated by the mass of the $\eta$ (or $\eta _{b}$) in Nature. The difference, due to quark-line disconnected diagrams is usually assumed to be small. For the determination of $\alpha _\mathrm{s}$, this approximation is actually irrelevant, since one can consider the moments at arbitrary (valence) quark masses.

Moments of correlation functions of the quark’s electromagnetic current can also be obtained from experimental data for $e^+e^-$ annihilation [534, 535]. This enables a non-lattice determination of $\alpha _\mathrm{s}$ using a similar analysis method. In particular, the same continuum perturbation theory enters both the lattice and the phenomenological determinations.

#### Discussion of computations

The method has been applied in HPQCD 08B [85] and in HPQCD 10 [73], based on the MILC ensembles with $2 + 1$ flavours of ASQTAD staggered quarks and HISQ valence quarks. The scale was set using $r_1 = 0.321(5)\,\hbox {fm}$ in HPQCD 08B and the updated value $r_1 = 0.3133(23)\,\hbox {fm}$ in HPQCD 10. The effective range of couplings used is here given for $n = 4$, which is the moment most dominated by short (perturbative) distances and important in the determination of $\alpha _\mathrm{s}$. The range is similar for other ratios. With $r_{4,1} = 0.7427$ and $R_4 = 1.281(5)$ determined in the continuum limit at the charm mass in [85], we have $\alpha _\mathrm{eff} = 0.38$ at the charm quark mass, which is the mass value where HPQCD 08B carries out the analysis. In HPQCD 10 a set of masses is used, with $R_4 \in [1.090, 1.293]$ which corresponds to $\alpha _\mathrm{eff} \in [0.121, 0.395]$.

**Fig. 24:**
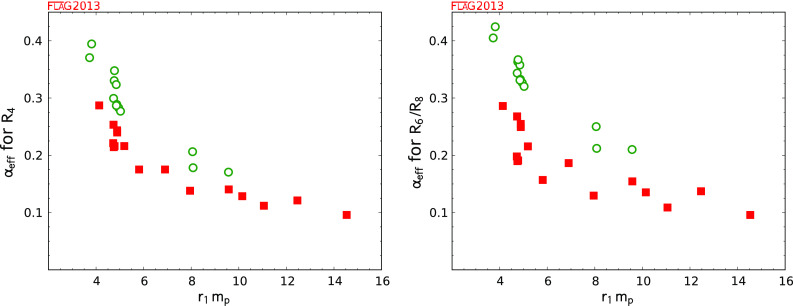
$\alpha _\mathrm{eff}$ for $R_4$ (*left*) and $R_6/R_8$ (*right*) versus $r_1m_\mathrm{p}$. *Symbols* correspond to our continuum limit criterion, namely  for data with $1\le a\mu \le 1.5$ and  for $a\mu >1.5$, while  is not present

The available data of HPQCD 10 are summarised in Fig. 24 where we plot $\alpha _\mathrm{eff}$ against $m_\mathrm{p} r_1$. For the continuum limit criterion, we choose the scale $\mu = 2\bar{m}_{h} \approx m_\mathrm{p}/1.1$, where we have taken $\bar{m}_{h}$ in the ${\overline{\mathrm{MS}}}$ scheme at scale $\bar{m}_{h}$ and the numerical value $1.1$ was determined in HPQCD 10B.

The data in Fig. 24 are grouped according to the range of $a\mu$ that they cover. The vertical spread of the results for $\alpha _\mathrm{eff}$ at fixed $r_1m_\mathrm{p}$ in the figure measures the discretisation errors seen for large masses: in the continuum we would expect all the points to lie on one universal curve. The plots illustrate the selection applied by our quality criterion for the continuum limit with our choices for $\mu$. Figure 24 gives reason for concern, since it shows that the discretisation errors that need to be removed in the continuum extrapolation are not small.

With our choices for $\mu$, the continuum limit criterion is satisfied for 3 lattice spacings when $\alpha _\mathrm{eff} \le 0.3$ and $n=4$. Larger $n$ moments are more influenced by non-perturbative effects. For the $n$ values considered, adding a gluon condensate term, which largely accounts for these effects, only changed error bars slightly. We note that HPQCD in their papers perform a global fit to all data using a joint expansion in powers of $\alpha _\mathrm{s}^n$, $(\Lambda /(m_\mathrm{p}/2))^j$ to parameterise the heavy-quark mass dependence, and $(am_\mathrm{p}/2)^{2i}$ to parameterise the lattice-spacing dependence. To obtain a good fit, they must exclude data with $am_\mathrm{p} > 1.95$ and include lattice-spacing terms $a^{2i}$ with $i$ greater than $10$. Because these fits include many more fit parameters than data points, HPQCD uses their expectations for the sizes of coefficients as Bayesian priors. The fits include data with masses as large as $am_\mathrm{p}/2 \sim 0.86$, so there is only minimal suppression of the many high-order contributions for the heavier masses. It is not clear, however, how sensitive the final results are to the larger $am_\mathrm{p}/2$ values in the data. The continuum limit of the fit is in agreement with a perturbative scale dependence (a five-loop running $\alpha _{\overline{\mathrm{MS}}}$ with a fitted five-loop coefficient in the beta-function is used). Indeed, Fig. 2 of Ref. [73] suggests that HPQCD’s fit describes the data well.

In Table 36 we list the current two point function results. Thus far, only one group has used this approach, which models complicated and potentially large cutoff effects together with a perturbative coefficient. We therefore are waiting to see confirmation by other collaborations of the small systematic errors obtained (cf. discussion in Sect. 9.9.2). We do however include the values of $\alpha _{\overline{\mathrm{MS}}}(M_Z)$ and $\Lambda _{\overline{\mathrm{MS}}}$ of HPQCD 10 in our final range.

**Table 36:** Current two point function results

Collaboration	Ref.	$N_\mathrm{f}$	Publication status	Renormalisation scale	Perturbative behaviour	Continuum extrapolation	Scale	$\Lambda _{\overline{\mathrm{MS}}}\,(\,{\mathrm {MeV}})$	$r_0\Lambda _{\overline{\mathrm{MS}}}$
HPQCD 10	[73]	$2+1$	A				$r_1 = 0.3133\,(23)\, \hbox {fm}^\dagger$	338 (10)$^\star$	0.809 (25)
HPQCD 08B	[85]	$2+1$	A				$r_1 = 0.321\,(5)\,\hbox {fm}^\dagger$	325 (18)$^+$	0.777 (42)

$^\dagger$ Scale is determined from $\Upsilon$ mass splitting

$$^\star $$ $$\alpha ^{(3)}_{\overline{\mathrm{MS}}}(5\,\hbox {GeV}) = 0.2034(21)$$, $$\alpha ^{(5)}_{\overline{\mathrm{MS}}}(M_Z) = 0.1183(7)$$

$$^+$$ $$\alpha ^{(4)}_{\overline{\mathrm{MS}}}(3\,\hbox {GeV}) = 0.251(6)$$, $$\alpha ^{(5)}_{\overline{\mathrm{MS}}}(M_Z) = 0.1174(12)$$

### $\alpha _\mathrm{s}$ from QCD vertices

#### General considerations

The most intuitive and in principle direct way to determine the coupling constant in QCD is to compute the appropriate three or four point gluon vertices or alternatively the quark–quark–gluon vertex or ghost–ghost–gluon vertex (i.e. $q\overline{q}A$ or $c\overline{c}A$ vertex respectively). A suitable combination of renormalisation constants then leads to the relation between the bare (lattice) and renormalised coupling constant. This procedure requires the implementation of a non-perturbative renormalisation condition and the fixing of the gauge. For the study of non-perturbative gauge fixing and the associated Gribov ambiguity, we refer to [536–538] and references therein.

In practice the Landau gauge is used and the renormalisation constants are defined by requiring that the vertex is equal to the tree-level value at a certain momentum configuration. The resulting renormalisation schemes are called ‘MOM’ scheme (symmetric momentum configuration) or ‘$\widetilde{\mathrm{MOM}}$’ (one momentum vanishes), which are then converted perturbatively to the $\overline{\mathrm{MS}}$ scheme.

A pioneering work to determine the three gluon vertex in the $N_\mathrm{f} = 0$ theory is Alles 96 [539] (which was followed by [540] for two flavour QCD); a more recent $N_\mathrm{f} = 0$ computation was [541] in which the three gluon vertex as well as the ghost–ghost–gluon vertex was considered. (This requires in general a computation of the propagator of the Faddeev–Popov ghost on the lattice.) The latter paper concluded that the resulting $\Lambda _{\overline{\mathrm{MS}}}$ depended strongly on the scheme used, the order of perturbation theory used in the matching and also on non-perturbative corrections, [542].

Subsequently in [543, 544] a specific $\widetilde{\mathrm{MOM}}$ scheme with zero ghost momentum for the ghost–ghost–gluon vertex was used. In this scheme, dubbed the ‘MM’ (Minimal MOM) or ‘Taylor’ (T) scheme, the vertex is not renormalised, and so the renormalised coupling reduces to

203 $$\begin{aligned} \alpha _\mathrm{T}(\mu ) = D^\mathrm{ghost}_\mathrm{lat}(\mu , a) D^\mathrm{gluon}_\mathrm{lat}(\mu , a)^2 \, {g_0^2(a) \over 4\pi }, \end{aligned}$$

where $D^\mathrm{ghost}_\mathrm{lat}$ and $D^\mathrm{gluon}_\mathrm{lat}$ are the (bare lattice) dressed ghost and gluon ‘form factors’ of these propagator functions in the Landau gauge,

204 $$\begin{aligned} D^{ab}(p)&= - \delta ^{ab}\, {D^\mathrm{ghost}(p) \over p^2},\nonumber \\ D_{\mu \nu }^{ab}(p)&= \delta ^{ab} \left( \delta _{\mu \nu } - {p_\mu p_\nu \over p^2} \right) \,{D^\mathrm{gluon}(p) \over p^2 }, \end{aligned}$$

and we have written the formula in the continuum with $D^\mathrm{ghost/gluon}(p)=D^\mathrm{ghost/gluon}_\mathrm{lat}(p, 0)$. Thus there is now no need to compute the ghost–ghost–gluon vertex, just the ghost and gluon propagators.

#### Discussion of computations

For the calculations considered here, to match to perturbative scaling, it was first necessary to reduce lattice artefacts by an $H(4)$ extrapolation procedure (addressing $O(4)$ rotational invariance), e.g. ETM 10F [550] or lattice perturbation theory, e.g. Sternbeck 12 [548]. To match to perturbation theory, collaborations vary in their approach. In ETM 10F [550] it was necessary to include the operator $A^2$ in the OPE of the ghost and gluon propagators, while in Sternbeck 12 [548] very large momenta are used and $a^2p^2$ and $a^4p^4$ terms are included in their fit to the momentum dependence. A further later refinement was the introduction of non-perturbative OPE power corrections in ETM 11D [547] and ETM 12C [546]. Although the expected leading power correction, $1/q^4$, was tried, ETM finds good agreement with their data only when they fit with the next-to-leading order term, $1/q^6$. The update ETM 13D [545] investigates this point in more detail, using better data with reduced statistical errors. They find that after again including the $1/q^6$ term they can describe their data over a large momentum range from about 1.75–7 GeV.

In all calculations except for Sternbeck 10 [549], Sternbeck 12 [548], the matching with the perturbative formula is performed including power corrections in the form of condensates, in particular ${\langle }A^2 \rangle$.

Three lattice spacings are present in almost all calculations with $N_\mathrm{f}=0$, $2$, but the scales $ap$ are rather large. This mostly results in a  on the continuum extrapolation. (Sternbeck 10 [549], Boucaud 01B [540] for $N_\mathrm{f}=2$. Ilgenfritz 10 [551], Boucaud 08 [544], Boucaud 05 [541], Becirevic 99B [556], Becirevic 99A [557], Boucaud 98B [558], Boucaud 98A [559], Alles 96 [539] for $N_\mathrm{f}=0$).

A  is reached in the $N_\mathrm{f}=0$ computations Boucaud 00A [555], 00B [554], 01A [553], Soto 01 [552] due to a rather small lattice spacing, but this is done on a lattice of a small physical size. The $N_\mathrm{f}=2+1+1$ calculation, fitting with condensates, is carried out for two lattice spacings and with $ap>1.5$, giving  for the continuum extrapolation as well. In ETM 10F [550] we have $0.25 < \alpha _\mathrm{eff} < 0.4$, while in ETM 11D, ETM 12C (and ETM 13) we find $0.24 < \alpha _\mathrm{eff} < 0.38$ which gives a green circle in these cases for the renormalisation scale. In ETM 10F the values of $ap$ violate our criterion for a continuum limit only slightly, and we give a .

In Sternbeck 10 [549], the coupling ranges over $0.07 \le \alpha _\mathrm{eff} \le 0.32$ for $N_\mathrm{f}=0$ and $0.19 \le \alpha _\mathrm{eff} \le 0.38$ for $N_\mathrm{f}=2$ giving  and  for the renormalisation scale, respectively. The fit with the perturbative formula is carried out without condensates, giving a satisfactory description of the data. In Boucaud 01A [553], depending on $a$, a large range of $\alpha _\mathrm{eff}$ is used which goes down to $0.2$ giving a  for the renormalisation scale and perturbative behaviour, and several lattice spacings are used leading to  in the continuum extrapolation. The $N_\mathrm{f}=2$ computation Boucaud 01B [553], fails the continuum limit criterion because both $a\mu$ is too large and an unimproved Wilson fermion action is used. Finally in the conference proceedings Sternbeck 12 [548], the $N_\mathrm{f}=0,2,3$ coupling $\alpha _\mathrm{T}$ is studied. Subtracting one-loop lattice artefacts and subsequently fitting with $a^2p^2$ and $a^4p^4$ additional lattice artefacts, agreement with the perturbative running is found for large momenta ($r_0^2p^2 > 600$) without the need for power corrections. In these comparisons, the values of $r_0\Lambda _{\overline{\mathrm{MS}}}$ from other collaborations are used. As no numbers are given, we have not introduced ratings for this study.

In Table 37 we summarise the results. Presently there are no $N_\mathrm{f} \ge 3$ calculations of $\alpha _\mathrm{s}$ from QCD vertices that satisfy the FLAG criteria to be included in the range.

**Table 37:** Results for the gluon–ghost vertex

Collaboration	Ref.	$N_\mathrm{f}$	Publication status	Renormalisation scale	Perturbative behaviour	Continuum extrapolation	Scale	$\Lambda _{\overline{\mathrm{MS}}}\,(\,{\mathrm {MeV}})$	$r_0\Lambda _{\overline{\mathrm{MS}}}$
ETM 13D	[545]	$2+1+1$	A				$f_\pi$	314 (7) (14) (10)^§^	$0.752\,(18)\,(34)\,(81)^\dagger$
ETM 12C	[546]	$2+1+1$	A				$f_\pi$	324 (17)^§^	$0.775\,(41)^\dagger$
ETM 11D	[547]	$2+1+1$	A				$f_\pi$	$316\,(13)\,(8)\,(^{+0}_{-9})^\star$	$0.756\,(31)\,(19)\,(^{+0}_{-22})^\dagger$
Sternbeck 12	[548]	$2+1$	C				Only running of $\alpha _\mathrm{s}$ in Fig. 4		
Sternbeck 12	[548]	2	C				Agreement with $r_0\Lambda _{\overline{\mathrm{MS}}}$ value of [59]		
Sternbeck 10	[549]	2	C						$0.60\,(3)\,(2)^\#$
ETM 10F	[550]	2	A				$f_\pi$	$330\,(23)\,(22)(^{+0}_{-33})$	$0.72\,(5)^+$
Boucaud 01B	[540]	2	A				$K^{*}-K$	$264\,(27)^{\star \star }$	0.669 (69)
Sternbeck 12	[548]	0	C				Agreement with $r_0\Lambda _{\overline{\mathrm{MS}}}$ value of [506]		
Sternbeck 10	[549]	0	C						$0.62\,(1)^\#$
Ilgenfritz 10	[551]	0	A				Only running of $\alpha _\mathrm{s}$ in Fig. 13		
Boucaud 08	[544]	0	A				$\sqrt{\sigma } = 445\,\hbox {MeV}$	$224\,(3)\,(^{+8}_{-5})$	$0.59\,(1)\,(^{+2}_{-1})$
Boucaud 05	[541]	0	A				$\sqrt{\sigma } = 445\,\hbox {MeV}$	320 (32)	0.85 (9)
Soto 01	[552]	0	A				$\sqrt{\sigma } = 445\,\hbox {MeV}$	260 (18)	0.69 (5)
Boucaud 01A	[553]	0	A				$\sqrt{\sigma } = 445\,\hbox {MeV}$	233 (28) MeV	0.62 (7)
Boucaud 00B	[554]	0	A					Only running of $\alpha _\mathrm{s}$	
Boucaud 00A	[555]	0	A				$\sqrt{\sigma } = 445\,\hbox {MeV}$	$237\,(3)\,(^{+~0}_{-10})$	$0.63\,(1)\,(^{+0}_{-3})$
Becirevic 99B	[556]	0	A				$\sqrt{\sigma } = 445\,\hbox {MeV}$	$319\,(14)\,(^{+10}_{-20})$	$0.84\,(4)\,(^{+3}_{-5})$
Becirevic 99A	[557]	0	A				$\sqrt{\sigma } = 445\,\hbox {MeV}$	$\lesssim 353\,(2)\,(^{+25}_{-15})$	$\lesssim 0.93 \,(^{+7}_{-4})$
Boucaud 98B	[558]	0	A				$\sqrt{\sigma } = 445\,\hbox {MeV}$	295 (5) (15)	0.78 (4)
Boucaud 98A	[559]	0	A				$\sqrt{\sigma } = 445\,\hbox {MeV}$	300 (5)	0.79 (1)
Alles 96	[539]	0	A				$\sqrt{\sigma } = 440\,\hbox {MeV}^{++}$	340 (50)	0.91 (13)

$^\dagger$ We use the $2+1$ value $r_0=0.472$ fm

^§^ $$\alpha _{\overline{\mathrm{MS}}}^{(5)}(M_Z)=0.1200(14)$$

$^\star$ First error is statistical; second is due to the lattice spacing and third is due to the chiral extrapolation. $\alpha _{\overline{\mathrm{MS}}}^{(5)}(M_Z)=0.1198(9)(5)(^{+0}_{-5})$

$^\#$ Only $r_0\Lambda _{\overline{\mathrm{MS}}}$ is given

$^+$ The determination of $r_0$ from the $f_\pi$ scale is found in [241]

$$^{\star \star }$$ $$\alpha _{\overline{\mathrm{MS}}}^{(5)}(M_Z)=0.113(3)(4)$$

$^{++}$ The scale is taken from the string tension computation of [507]

### Summary

#### The present situation

**Fig. 25:**
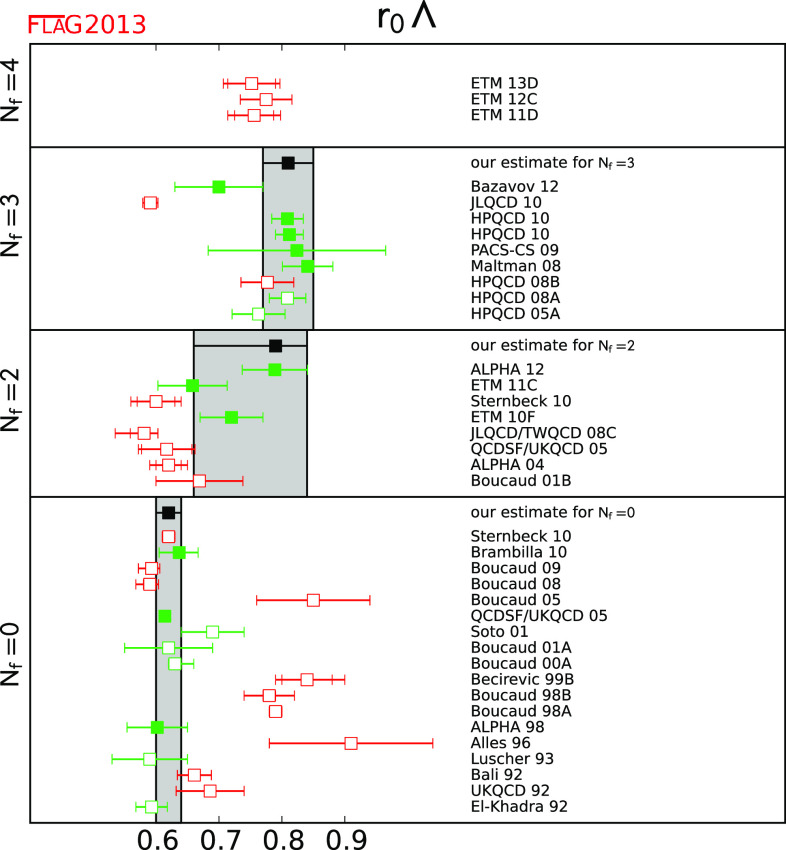
$r_0\Lambda _{\overline{\mathrm{MS}}}$ estimates for $N_\mathrm{f} = 0$, $2$, $3$, $4$ flavours. *Full green squares* are used in our final ranges, *open green squares* also indicate that there are no red squares in the colour coding but the computations were superseded by later more complete ones, while *red open squares* mean that there is at least one *red square* in the colour coding

We first summarise the status of lattice-QCD calculations of the QCD scale $\Lambda _{\overline{\mathrm{MS}}}$. Figure 25 shows all results for $r_0\Lambda _{\overline{\mathrm{MS}}}$ discussed in the previous sections.

Many of the numbers are the ones given directly in the papers. However, when only $\Lambda _{\overline{\mathrm{MS}}}$ in physical units (MeV) is available, we have converted them by multiplying with the value of $r_0$ in physical units. The notation used is full green squares for results used in our final average, while an open green square indicates that there are no red squares in the previous colour coding but the computation does not enter the ranges because either it has been superseded by an update or it is not published. Red open squares mean that there is at least one red square in the colour coding.

For $N_\mathrm{f}=0$ there is relatively little spread in the more recent numbers, even in those which do not satisfy our quality criteria. Clearly one could improve the statistical and many systematic errors considerably nowadays, but the emphasis is on the theory with quarks.

When two flavours of quarks are included, the numbers extracted by the various groups show a considerable spread, as in particular older computations did not yet control the systematics sufficiently. This illustrates the difficulty of the problem and emphasises the need for strict quality criteria. The agreement among the more modern calculations with three or more flavours, however, is quite good.

**Fig. 26:**
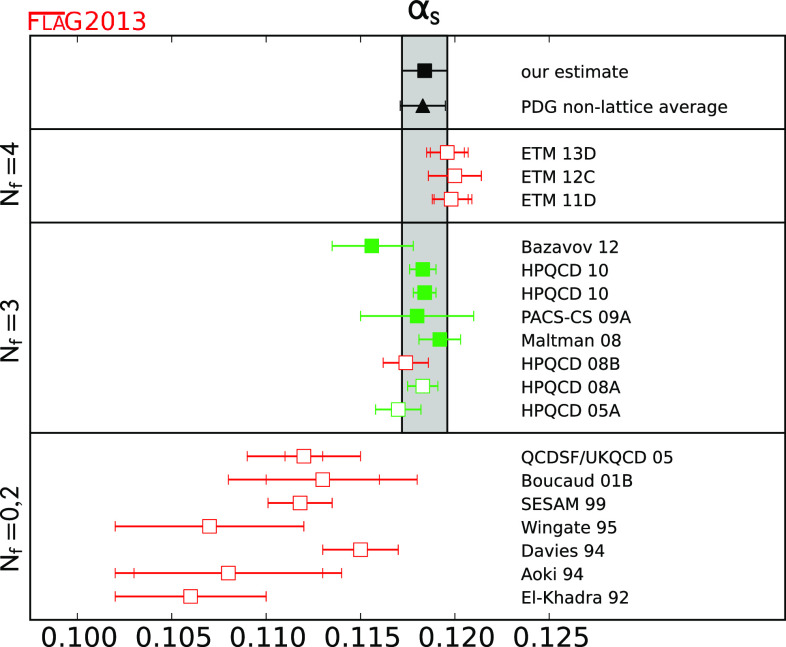
$\alpha _{\overline{\mathrm{MS}}}^{(5)}(M_Z)$, the coupling constant in the $\overline{\mathrm{MS}}$ scheme at the $Z$ mass. The results labelled $N_\mathrm{f}=0,2$ use estimates for $N_\mathrm{f}=3$ obtained by first extrapolating in $N_\mathrm{f}$ from $N_\mathrm{f}=0,2$ results. Since this is not a theoretically justified procedure, these are not included in our final estimate and are thus given a *red symbol*. However, they are shown to indicate the progress made since these early calculations. The PDG entry indicates the outcome of their analysis excluding lattice results (see Sect. 9.9.4)

We now turn to the status of the essential result for phenomenology, $\alpha _{\overline{\mathrm{MS}}}^{(5)}(M_Z)$. In Table 38 and Fig. 26 we show all the results for $\alpha _{\overline{\mathrm{MS}}}^{(5)}(M_Z)$ (i.e. $\alpha _{\overline{\mathrm{MS}}}$ at the $M_Z$ mass) obtained from $N_\mathrm{f}=2+1$ and $N_\mathrm{f} = 2+1+1$ simulations. For comparison, we also include results from $N_\mathrm{f} = 0,2$ simulations, which are not relevant for phenomenology. For the $N_\mathrm{f} \ge 3$ simulations, the conversion from $N_\mathrm{f}= 3$ to $N_\mathrm{f}= 5$ is made by matching the coupling constant at the charm and bottom quark thresholds and using the scale as determined or used by the authors. For $N_\mathrm{f} = 0$, $2$ the results for $\alpha _{\overline{\mathrm{MS}}}$ in the summary table come from evaluations of $\alpha _{\overline{\mathrm{MS}}}$ at a low scale and are extrapolated in $N_\mathrm{f}$ to $N_\mathrm{f}= 3$.

**Table 38:** Results for $\alpha _{\overline{\mathrm{MS}}}(M_\mathrm {Z})$. $N_\mathrm{f} = 3$ results are matched at the charm and bottom thresholds and scaled to $M_Z$ to obtain the $N_\mathrm{f} =5$ result. The arrows in the $N_\mathrm{f}$ column indicates which $N_\mathrm{f}$ ($N_\mathrm{f} = 0$, $2$ or a combination of both) were used to first extrapolate to $N_\mathrm{f} = 3$ or estimate the $N_\mathrm{f} = 3$ value through a model/assumption. The exact procedures used vary and are given in the various papers

Collaboration	Ref.	$N_\mathrm{f}$	Publication status	Renormalisation scale	Perturbative behaviour	Continuum extrapolation	$\alpha _{\overline{\mathrm{MS}}}(M_\mathrm {Z})$	Method	Table
ETM 13D	[545]	$2+1+1$	A				0.1196 (4) (8) (16)	Gluon–ghost vertex	37
ETM 12C	[546]	$2+1+1$	A				0.1200 (14)	Gluon–ghost vertex	37
ETM 11D	[547]	$2+1+1$	A				$0.1198\,(9)\,(5)\,(^{+0}_{-5})$	Gluon–ghost vertex	37
Bazavov 12	[504]	$2+1$	A				$0.1156\,(^{+21}_{-22})$	$Q$–$\bar{Q}$ potential	33
HPQCD 10	[73]	$2+1$	A				0.1183 (7)	Current two points	36
HPQCD 10	[73]	$2+1$	A				0.1184 (6)	Wilson loops	35
PACS-CS 09A	[487]	$2+1$	A				$0.118\,(3)^\#$	Schrödinger functional	32
Maltman 08	[518]	$2+1$	A				$0.1192\,(11)$	Wilson loops	35
HPQCD 08B	[85]	$2+1$	A				0.1174 (12)	Current two points	36
HPQCD 08A	[515]	$2+1$	A				0.1183 (8)	Wilson loops	35
HPQCD 05A	[514]	$2+1$	A				0.1170 (12)	Wilson loops	35
QCDSF/UKQCD 05	[519]	$0,2 \rightarrow 3$	A				0.112 (1) (2)	Wilson loops	35
Boucaud 01B	[540]	$2\rightarrow 3$	A				0.113 (3) (4)	Gluon–ghost vertex	37
SESAM 99	[520]	$0,2\rightarrow 3$	A				0.1118 (17)	Wilson loops	35
Wingate 95	[521]	$0,2\rightarrow 3$	A				0.107 (5)	Wilson loops	35
Davies 94	[522]	$0,2\rightarrow 3$	A				0.115 (2)	Wilson loops	35
Aoki 94	[523]	$2\rightarrow 3$	A				0.108 (5) (4)	Wilson loops	35
El-Khadra 92	[524]	$0\rightarrow 3$	A				0.106 (4)	Wilson loops	35

$^\#$ Result with a linear continuum extrapolation in $a$

As can be seen from the tables and figures, at present there are several computations satisfying the quality criteria to be included in the FLAG average. We note that none of those calculations of $\alpha _{\overline{\mathrm{MS}}}^{(5)}(M_Z)$ satisfy all of our more stringent criteria: a  for the renormalisation scale, perturbative behaviour and continuum extrapolation. The results, however, are obtained from four different methods that have different associated systematics, and agree well within the stated uncertainties.

#### Our range for $\alpha _{\overline{\mathrm{MS}}}^{(5)}$

We now explain the determination of our range. We only include those results without a red tag and that are published in a refereed journal. We also do not include any numbers which were obtained by extrapolating from theories with less than three flavours. There is no real basis for such extrapolations; rather they use ad hoc assumptions on the low-energy behaviour of the theories. One also notices from the published results that the estimated numbers are quite significantly below those with at least $2+1$ flavours.

A general issue with most recent lattice calculations of $\alpha _{\overline{\mathrm{MS}}}$ is that they are dominated by perturbative truncation errors, which are difficult to estimate. This concern also applies to many non-lattice determinations. Further, all results except for those of Sects. 9.3, 9.6 are based on extractions of $\alpha _{\overline{\mathrm{MS}}}$ that are largely influenced by data with $\alpha _\mathrm{eff}\ge 0.3$. At smaller $\alpha$ the momentum scale $\mu$ quickly is at or above $a^{-1}$. We have included computations using $a\mu$ up to $1.5$ and $\alpha _\mathrm{eff}$ up to 0.4, but one would ideally like to be significantly below that. Accordingly we wish at this stage to estimate the error ranges in a conservative manner, and not simply perform weighted averages of the individual errors estimated by each group.

Many of the methods have thus far only been applied by a single collaboration, and with simulation parameters that could still be improved. We therefore think that the following aspects of the individual calculations are important to keep in mind, and look forward to additional clarification and/or corroboration in the future.

$\bullet \,$ The potential computations Brambilla 10 [506], ETM 11C [505] and Bazavov 12 [504] give evidence that they have reached distances where perturbation theory can be used. However, in addition to $\Lambda _\mathrm{QCD}$, a scale is introduced into the perturbative prediction by the process of subtracting the renormalon contribution. The extractions of $\Lambda$ are dominated by data with $\alpha _\mathrm{eff}\ge 0.3$. In contrast, Ref. [508], which studies the force instead of the potential and therefore does not need a renormalon subtraction, finds that significantly smaller lattice spacings would be needed in order for perturbation theory to be reliable. Further study is needed to clarify the situation.

$\bullet$ In the determination of $\alpha _s$ from observables at the lattice spacing scale, there is an interplay of higher-order perturbative terms and lattice artefacts. In HPQCD 05A [514], HPQCD 08A [515] and Maltman 08 [518] both lattice artefacts (which are power corrections in this approach) and higher-order perturbative terms are fitted. We note that, Maltman 08 [518] and HPQCD 08A [515] analyse largely the same data set but use different versions of the perturbative expansion and treatments of non-perturbative terms. After adjusting for the slightly different lattice scales used, the values of $\alpha _{\overline{\mathrm{MS}}}(M_Z)$ differ by 0.0004–0.0008 for the three quantities considered. In fact the largest of these differences ($0.0008$) comes from a tadpole-improved loop, which is expected to be best behaved perturbatively.

$\bullet$ Another computation with very small errors is HPQCD 10 [73], where correlation functions of heavy quarks are used to construct short-distance quantities. Due to the large quark masses needed to reach the region of small coupling, considerable discretisation errors are present; see Fig. 24. These are treated by fits to the perturbative running (a five-loop running $\alpha _{\overline{\mathrm{MS}}}$ with a fitted five-loop coefficient in the beta-function is used) with high-order terms in a double expansion in $a^2\Lambda ^2$ and $a^2 m_{h}^2$ supplemented by priors which limit the size of the coefficients. The priors play an especially important role in these fits given the much larger number of fit parameters than data points. We note, however, that the size of the coefficients does not prevent high-order terms from contributing significantly, since the data include values of $am_\mathrm{p}/2$ that are rather close to 1. It is not clear how sensitive the final results are to these large values of $am_\mathrm{p}/2$.

As previously discussed $\alpha _{\overline{\mathrm{MS}}}^{(5)}(M_Z)$ is summarised in Table 38 and Fig. 26. Early computations estimated the effect of the strange quark by extrapolations from $N_\mathrm{f}=0$ and $N_\mathrm{f}=2$. They are included in the table and figure but do not enter the final range. Indeed with our present knowledge we see that such estimates were rather rough ones, but also other systematic errors such as a lack of control of discretisation errors presumably play a rôle in the differences seen with today’s results. A number of calculations that include the effect of the strange quark make up our final estimate. These are Bazavov 12, HPQCD 10A/10B, PACS-CS 09A, Maltman 08 while HPQCD 08A/05A have been superseded by more complete calculations. We obtain the central value for our range,

205 $$\begin{aligned} \alpha _{\overline{\mathrm{MS}}}^{(5)}(M_Z) = 0.1184(12), \end{aligned}$$

from the weighted average of the five results. Of the results that enter our range, those from Wilson loops (HPQCD 10A and Maltman 08) and current two-point correlators (HPQCD 10B) presently have the smallest quoted errors. In both cases the uncertainties are dominated by perturbative truncation errors. Such errors are difficult to estimate, and there is a considerable spread in opinion both in the lattice and continuum phenomenology communities regarding how they should be estimated. We therefore choose to be conservative, and take a larger range for $\alpha _{\overline{\mathrm{MS}}}^{(5)}(M_Z)$ than one would obtain from the weighted average, or even from the most precise individual calculation. We make a conservative estimate of the perturbative uncertainty in the calculation of $\alpha _\mathrm{s}$ from small Wilson loops, and take that estimate as the error range of the current weighted average of all lattice results. One approach for making such an estimate would be to take the largest of the differences between the calculations of Maltman 08 [518] and HPQCD 08A [515], 0.0008, which comes from the quantity computed by both groups that is expected to be best behaved perturbatively. This is somewhat larger than some of the estimates in the individual papers. An even more conservative estimate increases this error further to make it commensurate with a power-counting estimate of the truncation errors in the Wilson loop analyses. Taking the coefficient $|c_4/c_1|\approx 2$ in Eq. (196) yields the estimate $\Delta \alpha _2 = 0.0012$ for $\alpha ^{(5)}_{\overline{\mathrm{MS}}}(M_Z)$. This is what we adopt as our final range.

The range for $\alpha _{\overline{\mathrm{MS}}}^{(5)}(M_Z)$ presented here is based on results with rather different systematics (apart from the matching across the charm threshold). We therefore believe that the true value is quite likely to lie within this range.

We would like to emphasise once more that all computations which enter this range rely on a perturbative inclusion of the charm and beauty quarks. While perturbation theory for the matching of $\bar{g}^2_{N_\mathrm{f}}$ and $\bar{g}^2_{N_\mathrm{f}-1}$ looks very well behaved even at the mass of the charm, this scale is rather low and we have no reliable information about the precision of perturbation theory. However, it seems unlikely that the associated uncertainty is comparable with the present errors. With future improved precision, this will become a relevant issue. Note that this uncertainty is also present in some of the phenomenological determinations, in particular from $\tau$ decays.

#### Ranges for $[r_0 \Lambda ]^{(N_\mathrm{f})}$ and $\Lambda _{{\overline{\mathrm{MS}}}}$

In the present situation, we give ranges for $[r_0 \Lambda ]^{(N_\mathrm{f})}$ and $\Lambda _{{\overline{\mathrm{MS}}}}$, discussing their determination case by case. We include results with $N_\mathrm{f}<3$ because it is interesting to see the $N_\mathrm{f}$-dependence of the connection of low- and high-energy QCD. This aids our understanding of the field theory and helps in finding possible ways to tackle it beyond the lattice approach. It is also of interest in providing an impression on the size of the vacuum polarisation effects of quarks, in particular with an eye on the still difficult-to-treat heavier charm and beauty quarks. Even if this information is rather qualitative, it may be valuable, given that it is of a completely non-perturbative nature.

We emphasise that results for $[r_0 \Lambda ]^{(0)}$ and $[r_0 \Lambda ]^{(2)}$ are *not*meant to be used in phenomenology.

For $N_\mathrm{f}=2+1+1$, we presently do not quote a range. Our best estimate is given by using the $N_\mathrm{f}=2+1$ result and converting it to $N_\mathrm{f}=2+1+1$ perturbatively at the charm quark-mass threshold.

For $N_\mathrm{f}=2+1$, we take as a central value the weighted average of Bazavov 12, HPQCD 10A, 10B, PACS-CS 09A and Maltman 08. For the error we take our own conservative estimate of the perturbative uncertainty remaining in the determinations from small Wilson loops, HPQCD 10A and Maltman 08. From an estimate of $|c_4/c_1|\approx 2$ we obtain (Eq. (196) in Sect. 9.6) $\Delta \Lambda /\Lambda = 0.05$. An independent estimate of the uncertainty due to the fit to the $a$-dependence in the analysis of moments of heavy quark correlators is much more difficult to make; as discussed above, and in the absence of confirmation by other groups, we are not yet ready to use the result of HPQCD 10 to reduce our conservative estimate of the errors from other approaches. Noting that the statistical error is negligible, we thus assign the just mentioned 5 % error to the overall range,

206 $$\begin{aligned} {[r_0 \Lambda _{{\overline{\mathrm{MS}}}}]^{(3)}} = 0.81(4). \end{aligned}$$

It is in good agreement with all $2+1$ results without red tags. In physical units, using $r_0=0.472$ fm, this means

207 $$\begin{aligned} \Lambda _{{\overline{\mathrm{MS}}}}^{(3)} = 339(17)\,\hbox {MeV}. \end{aligned}$$

For $N_\mathrm{f}=2$, at present there is one computation with a  rating for all criteria, ALPHA 12. We adopt it as our central value and enlarge the error to cover the central values of the other two results with filled green boxes. This results in an asymmetric error. Our present range is

208 $$\begin{aligned} {[r_0 \Lambda _{{\overline{\mathrm{MS}}}}]^{(2)}} = 0.79(^{+~5}_{-13}), \quad \end{aligned}$$

and in physical units, using $r_0=0.472$ fm,

209 $$\begin{aligned} \Lambda _{{\overline{\mathrm{MS}}}}^{(2)} = 330(^{+21}_{-54})\, \hbox {MeV}. \quad \end{aligned}$$

A weighted average of the three eligible numbers would yield $[r_0 \Lambda _{{\overline{\mathrm{MS}}}}]^{(2)} = 0.725(30)$, not covering the best result and in particular leading to a smaller error than we feel is justified, given the issues discussed above. Thus we believe that our estimate is a conservative choice; the lower value of ETM 11C [505] leads to the large downwards error. We hope that future work will improve the situation.

For $N_\mathrm{f}=0$, ALPHA 98 has a  in the continuum limit since the ${O}(a)$ improvement at the boundary was carried out only to one-loop order. On the other hand, QCDSF/UKQCD 05 receives a  for the perturbative behaviour since a power law correction was fitted to the results, and additionally we note again that it is not obvious that higher-order perturbative terms are negligible; an estimate as for HPQCD 10A (with $|c_4/c_1| \approx 2$) would be $\Delta [r_0 \Lambda _{{\overline{\mathrm{MS}}}}]^{(0)} = 0.018$. A third result which enters our average is Brambilla 10 but we exclude the older estimates shown in the graph. They have a limited control of the systematic errors due to power law corrections and discretisation errors.^38^ Taking a weighted average of the three numbers, we obtain $[r_0 \Lambda _{{\overline{\mathrm{MS}}}}]^{(0)} = 0.615(5)$, dominated by the QCDSF/UKQCD 05 result. Since we are not yet convinced that such a small uncertainty has been reached, we prefer to presently take a range which encompasses all three central values and whose uncertainty comes close to our estimate of the perturbative error:

210 $$\begin{aligned} {[r_0 \Lambda _{{\overline{\mathrm{MS}}}}]^{(0)}} = 0.62(2). \quad \end{aligned}$$

Converting to physical units, using $r_0=0.472$ fm,

211 $$\begin{aligned} \Lambda _{{\overline{\mathrm{MS}}}}^{(0)} = 260(7)\,\hbox {MeV}. \quad \end{aligned}$$

While the conversion of the $\Lambda$-parameter to physical units is quite unambiguous for $N_\mathrm{f}=2+1$, our choice of $r_0=0.472$ fm also for smaller numbers of flavour amounts to a convention, in particular for $N_\mathrm{f}=0$. Indeed, in the Tables 32, 33, 34, 35, 36, and 37 somewhat different numbers in MeV are found.

How sure are we about our ranges for $[r_0 \Lambda _{{\overline{\mathrm{MS}}}}]$? In one case we have a result, Eq. (208) which easily passes our criteria, in another one (Eq. (210)) we have three compatible results which are close to that quality and agree. For $N_\mathrm{f}=2+1$ the range (Eq. (206)) takes account of results with rather different systematics (apart from the matching across the charm threshold). We therefore find it difficult to imagine that the ranges could be violated by much.

#### Conclusions

With the present results our range for the strong coupling is (repeating Eq. (205))

$$\begin{aligned} \alpha _{\overline{\mathrm{MS}}}^{(5)}(M_Z) = 0.1184(12). \end{aligned}$$

As can be seen from Fig. 26, when surveying the green data points, the individual lattice results agree within their quoted errors. Further those points are based on different methods for determining $\alpha _\mathrm{s}$, each with its own difficulties and limitations. Thus the overall consistency of the lattice $\alpha _\mathrm{s}$ results engenders confidence in our range.

While our range for $\alpha _{\overline{\mathrm{MS}}}(M_Z)$ in Eq. (205) has about the same central value as the PDG average of lattice results, $\alpha _{\overline{\mathrm{MS}}}(M_Z) = 0.1185(5)$, our error estimate is more conservative, derived from an estimate of perturbative uncertainties. In contrast, in the PDG review all published lattice results are taken with their errors at face value and a $\chi$-squared weighted average is chosen because the results are largely independent and compatible within errors. We note that there is a diversity of opinion over the size of our range for $\alpha _{\overline{\mathrm{MS}}}(M_Z)$ in Eq. (205) within FLAG. Some members are sufficiently convinced by the overall consistency of the results from various groups within their quoted errors, as well as by the internal tests performed by individual groups, to take the quoted errors at face value. Others prefer the more conservative error estimate cited above, which aims to account for the difficulty associated with estimating perturbative truncation errors, the largest source of uncertainty in most of the calculations that enter the range. Given this diversity of opinion, we think it is appropriate to choose the more conservative estimate for our quoted range.

It is also interesting to compare our result, Eq. (205), with the value quoted by the PDG for the average over all other (non-lattice) sources, $\alpha _\mathrm{s}=0.1183(12)$. In the 2013 review, for all subclasses of $\alpha _\mathrm{s}$ determinations except for the lattice results, the results disagree beyond those expected from the quoted errors, presumably because of the challenges of evaluating systematic uncertainties. Thus the quoted range for each subclass is increased to encompass the central values of all individual determinations. This leads to subclass averages with errors that are larger than the smallest error of individual determinations by factors between two and four.

Our range for the lattice determination of $\alpha _{\overline{\mathrm{MS}}}(M_Z)$ in Eq. (205) is in excellent agreement with the PDG non-lattice average: the work done on the lattice provides an entirely independent determination, which already reaches the same precision even with our conservative estimate of the perturbative error.

We finish by commenting on perspectives for the future. In the next few years we anticipate that a growing number of lattice calculations of $\alpha _\mathrm{s}$ from different quantities and by different collaborations will enable increasingly precise determinations, coupled with stringent cross-checks. The determination of $\alpha _\mathrm{s}$ from observables at the lattice spacing scale will improve due to a further reduction of the lattice spacing. This reduces $\alpha _\mathrm{eff}$ and thus the dominating error in $\alpha _{\overline{\mathrm{MS}}}$. Schrödinger functional methods for $N_\mathrm{f}=2+1$ will certainly reach the precision of the present $N_\mathrm{f}=2$ results soon, as this just requires an application of the presently known techniques. Furthermore, we may expect a significant reduction of errors due to new definitions of running couplings [491, 492] using the Yang Mills gradient flow [183]. Factors of two and more in precision are certainly possible. At this point it will then also be necessary to include the charm quark in the computations such that the perturbative matching of $N_\mathrm{f}=2+1$ and $2+1+1$ theories at the charm quark threshold is avoided. $N_\mathrm{f}=2+1+1$ simulations are presently being carried out.

## Appendix A: Glossary

### A.1 Lattice actions

In this appendix we give brief descriptions of the lattice actions used in the simulations and summarise their main features.

#### A.1.1 Gauge actions

The simplest and most widely used discretisation of the Yang–Mills part of the QCD action is the Wilson plaquette action [560]:

212 $$\begin{aligned} S_{G} = \beta \sum _{x} \sum _{\mu <\nu }\left( 1-\frac{1}{3}{Re\,\mathrm{Tr}\,}\,W_{\mu \nu }^{1\times 1}(x)\right) , \end{aligned}$$

where $\beta \equiv 6/g_0^2$ (with $g_0$ the bare gauge coupling) and the plaquette $W_{\mu \nu }^{1\times 1}(x)$ is the product of link variables around an elementary square of the lattice, i.e.

213 $$\begin{aligned} W_{\mu \nu }^{1\times 1}(x) \equiv U_\mu (x)U_\nu (x+a\hat{\mu })U_\mu (x+a\hat{\nu })^{-1} U_\nu (x)^{-1}.\nonumber \\ \end{aligned}$$

This expression reproduces the Euclidean Yang–Mills action in the continuum up to corrections of order $a^2$. There is a general formalism, known as the “Symanzik improvement programme” [9, 10], which is designed to cancel the leading lattice artefacts, such that observables have an accelerated rate of convergence to the continuum limit. The improvement programme is implemented by adding higher-dimensional operators, whose coefficients must be tuned appropriately in order to cancel the leading lattice artefacts. The effectiveness of this procedure depends largely on the method with which the coefficients are determined. The most widely applied methods (in ascending order of effectiveness) include perturbation theory, tadpole-improved (partially resummed) perturbation theory, renormalisation group methods, and the non-perturbative evaluation of improvement conditions.

In the case of Yang–Mills theory, the simplest version of an improved lattice action is obtained by adding rectangular $1\times 2$ loops to the plaquette action, i.e.

214 $$\begin{aligned} S_{G}^\mathrm{imp}&= \beta \sum _{x}\left\{ c_0\sum _{\mu <\nu }\left( 1-\frac{1}{3}{Re\,\mathrm{Tr}\,}\,W_{\mu \nu }^{1\times 1}(x)\right) \right. \nonumber \\&\left. +c_1\sum _{\mu ,\nu } \left( 1-\frac{1}{3}{Re\,\mathrm{Tr}\,}\,W_{\mu \nu }^{1\times 2}(x)\right) \right\} , \end{aligned}$$

where the coefficients $c_0, c_1$ satisfy the normalisation condition $c_0+8c_1=1$. The *Symanzik-improved* [561], *Iwasaki* [562], and *DBW2* [563, 564] actions are all defined through Eq. (214) via particular choices for $c_0, c_1$. Details are listed in Table 39 together with the abbreviations used in the summary tables.

**Table 39:** Summary of lattice gauge actions. The leading lattice artefacts are $O(a^2)$ or better for all discretisations

Abbrev.	$c_1$	Description
Wilson	0	Wilson plaquette action
tlSym	$-1/12$	Tree-level Symanzik-improved gauge action
tadSym	Variable	Tadpole Symanzik-improved gauge action
Iwasaki	$-0.331$	Renormalisation group improved (“Iwasaki”) action
DBW2	$-1.4088$	Renormalisation group improved (“DBW2”) action

#### A.1.2 Light-quark actions

If one attempts to discretise the quark action, one is faced with the fermion doubling problem: the naive lattice transcription produces a 16-fold degeneracy of the fermion spectrum.

*Wilson fermions*

Wilson’s solution to the fermion doubling problem is based on adding a dimension-5 (irrelevant) operator to the lattice action. The Wilson-Dirac operator for the massless case reads [560, 565]

215 $$\begin{aligned} D_{w} = \frac{1}{2}\gamma _\mu (\nabla _\mu +\nabla _\mu ^*)+a\nabla _\mu ^*\nabla _\mu , \end{aligned}$$

where $\nabla _\mu ,\,\nabla _\mu ^*$ denote the covariant forward and backward lattice derivatives, respectively. The addition of the Wilson term $a\nabla _\mu ^*\nabla _\mu$, results in fermion doublers acquiring a mass proportional to the inverse lattice spacing; close to the continuum limit these extra degrees of freedom are removed from the low-energy spectrum. However, the Wilson term also results in an explicit breaking of chiral symmetry even at zero bare quark mass. Consequently, it also generates divergences proportional to the UV cutoff (inverse lattice spacing), besides the usual logarithmic ones. Therefore the chiral limit of the regularised theory is not defined simply by the vanishing of the bare quark mass but must be appropriately tuned. As a consequence quark mass renormalisation requires a power subtraction on top of the standard multiplicative logarithmic renormalisation. The breaking of chiral symmetry also implies that the non-renormalisation theorem has to be applied with care [566, 567], resulting in a normalisation factor for the axial current which is a regular function of the bare coupling. On the other hand, vector symmetry is unaffected by the Wilson term and thus a lattice (point split) vector current is conserved and obeys the usual non-renormalisation theorem with a trivial (unity) normalisation factor. Thus, compared to lattice fermion actions which preserve chiral symmetry, or a subgroup of it, the Wilson regularisation typically results in more complicated renormalisation patterns.

Furthermore, the leading-order lattice artefacts are of order $a$. With the help of the Symanzik improvement programme, the leading artefacts can be cancelled in the action by adding the so-called “Clover” or Sheikholeslami–Wohlert (SW) term [568]. The resulting expression in the massless case reads

216 $$\begin{aligned} D_\mathrm{sw} = D_{w}+\frac{ia}{4}\,c_{\mathrm{sw}}\sigma _{\mu \nu }\widehat{F}_{\mu \nu }, \end{aligned}$$

where $\sigma _{\mu \nu }=\frac{i}{2}[\gamma _\mu ,\gamma _\nu ]$, and $\widehat{F}_{\mu \nu }$ is a lattice transcription of the gluon field strength tensor $F_{\mu \nu }$. The coefficient $c_{\mathrm{sw}}$ can be determined perturbatively at tree level ($c_{\mathrm{sw}}= 1$; tree-level improvement or tlSW for short), via a mean-field approach [516] (mean-field improvement or mfSW) or via a non-perturbative approach [569] (non-perturbatively improved or npSW). Hadron masses, computed using $D_\mathrm{sw}$, with the coefficient $c_{\mathrm{sw}}$ determined non-perturbatively, will approach the continuum limit with a rate proportional to $a^2$; with tlSW for $c_{\mathrm{sw}}$ the rate is proportional to $g_0^2 a$.

Other observables require additional improvement coefficients [568]. A common example consists in the computation of the matrix element ${\langle }\alpha \vert Q \vert \beta \rangle$ of a composite field $Q$ of dimension-$d$ with external states $\vert \alpha \rangle$ and $\vert \beta \rangle$. In the simplest cases, the above bare matrix element diverges logarithmically and a single renormalisation parameter $Z_Q$ is adequate to render it finite. It then approaches the continuum limit with a rate proportional to the lattice spacing $a$, even when the lattice action contains the Clover term. In order to reduce discretisation errors to ${\mathcal {O}}(a^2)$, the lattice definition of the composite operator $Q$ must be modified (or “improved”), by the addition of all dimension-$(d+1)$ operators with the same lattice symmetries as $Q$. Each of these terms is accompanied by a coefficient which must be tuned in a way analogous to that of $c_{\mathrm{sw}}$. Once these coefficients are determined non-perturbatively, the renormalised matrix element of the improved operator, computed with a npSW action, converges to the continuum limit with a rate proportional to $a^2$. A tlSW improvement of these coefficients and $c_{\mathrm{sw}}$ will result in a rate proportional to $g_0^2 a$.

It is important to stress that the improvement procedure does not affect the chiral properties of Wilson fermions; chiral symmetry remains broken.

Finally, we mention “twisted-mass QCD” as a method which was originally designed to address another problem of Wilson’s discretisation: the Wilson-Dirac operator is not protected against the occurrence of unphysical zero modes, which manifest themselves as “exceptional” configurations. They occur with a certain frequency in numerical simulations with Wilson quarks and can lead to strong statistical fluctuations. The problem can be cured by introducing a so-called “chirally twisted” mass term. The most common formulation applies to a flavour doublet $\bar{\psi }= ( u \quad d)$ of mass degenerate quarks, with the fermionic part of the QCD action in the continuum assuming the form [310]

217 $$\begin{aligned} S_{F}^\mathrm{tm;cont} \!=\! \int \!\hbox {d}^4{x}\, \bar{\psi }(x)(\gamma _\mu D_\mu + m + i\mu _{q}\gamma _5\tau ^3)\psi (x). \end{aligned}$$

Here, $\mu _{q}$ is the twisted-mass parameter, and $\tau ^3$ is a Pauli matrix in flavour space. The standard action in the continuum can be recovered via a global chiral field rotation. The physical quark mass is obtained as a function of the two mass parameters $m$ and $\mu _{q}$. The corresponding lattice regularisation of twisted-mass QCD (tmWil) for $N_\mathrm{f}=2$ flavours is defined through the fermion matrix

218 $$\begin{aligned} D_{w}+m_0+i\mu _{q}\gamma _5\tau ^3. \end{aligned}$$

Although this formulation breaks physical parity and flavour symmetries, resulting in non-degenerate neutral and charged pions, is has a number of advantages over standard Wilson fermions. Firstly, the presence of the twisted-mass parameter $\mu _{q}$ protects the discretised theory against unphysical zero modes. A second attractive feature of twisted-mass lattice QCD is the fact that, once the bare mass parameter $m_0$ is tuned to its “critical value” (corresponding to massless pions in the standard Wilson formulation), the leading lattice artefacts are of order $a^2$ without the need to add the Sheikholeslami–Wohlert term in the action, or other improving coefficients [570]. A third important advantage is that, although the problem of explicit chiral symmetry breaking remains, quantities computed with twisted fermions with a suitable tuning of the mass parameter $\mu _{q}$, are subject to renormalisation patterns which are simpler than the ones with standard Wilson fermions. Well-known examples are the pseudoscalar decay constant and $B_{K}$.

*Staggered fermions*

An alternative procedure to deal with the doubling problem is based on so-called “staggered” or Kogut–Susskind fermions [571–574]. Here the degeneracy is only lifted partially, from 16 down to 4. It has become customary to refer to these residual doublers as “tastes” in order to distinguish them from physical flavours. Taste-changing interactions can occur via the exchange of gluons with one or more components of momentum near the cutoff $\pi /a$. This leads to the breaking of the SU(4) vector symmetry among tastes, thereby generating order $a^2$ lattice artefacts.

The residual doubling of staggered quarks (four tastes per flavour) is removed by taking a fractional power of the fermion determinant [575]—the “fourth-root procedure,” or, sometimes, the “fourth-root trick.” This procedure would be unproblematic if the action had full SU(4) taste symmetry, which would give a Dirac operator that was block-diagonal in taste space. However, the breaking of taste symmetry at non-zero lattice spacing leads to a variety of problems. In fact, the fourth root of the determinant is not equivalent to the determinant of any local lattice Dirac operator [576]. This in turn leads to violations of unitarity on the lattice [577–580].

According to standard renormalisation group lore, the taste violations, which are associated with lattice operators of dimension greater than four, might be expected go away in the continuum limit, resulting in the restoration of locality and unitarity. However, there is a problem with applying the standard lore to this non-standard situation: the usual renormalisation group reasoning assumes that the lattice action is local. Nevertheless, Shamir [581, 582] shows that one may apply the renormalisation group to a “nearby” local theory, and thereby gives a strong argument that the desired local, unitary theory of QCD is reproduced by the rooted staggered lattice theory in the continuum limit.

A version of chiral perturbation that includes the lattice artefacts due to taste violations and rooting (“rooted staggered chiral perturbation theory”) can also be worked out [583–585] and shown to correctly describe the unitarity-violating lattice artefacts in the pion sector [578, 586]. This provides additional evidence that the desired continuum limit can be obtained. Further, it gives a practical method for removing the lattice artefacts from simulation results. Versions of rooted staggered chiral perturbation theory exist for heavy–light mesons with staggered light quarks but non-staggered heavy quarks [587], heavy–light mesons with staggered light and heavy quarks [339, 588], staggered baryons [589], and mixed actions with a staggered sea [317, 590], as well as the pion-only version referenced above.

There is also considerable numerical evidence that the rooting procedure works as desired. This includes investigations in the Schwinger model [591–593], studies of the eigenvalues of the Dirac operator in QCD [594–597], and evidence for taste restoration in the pion spectrum as $a\rightarrow 0$ [15, 36].

Issues with the rooting procedure have led Creutz [598–604] to argue that the continuum limit of the rooted staggered theory cannot be QCD. These objections have, however, been answered in Refs. [12–14, 597, 605–608]. In particular, a claim that the continuum ’t Hooft vertex [12–14, 597, 605–608] could not be properly reproduced by the rooted theory has been refuted [597, 606].

Overall, despite the lack of rigorous proof of the correctness of the rooting procedure, we think the evidence is strong enough to consider staggered QCD simulations on a par with simulations using other actions. See the following reviews for further evidence and discussion: [11–15].

*Improved staggered fermions*

An improvement program can be used to suppress taste-changing interactions, leading to “improved staggered fermions,” with the so-called “Asqtad” [611, “HISQ” [612, “Stout-smeared” [613], and “HYP” [614] actions as the most common versions. All these actions smear the gauge links in order to reduce the coupling of high-momentum gluons to the quarks, with the main goal of decreasing taste-violating interactions. In the Asqtad case, this is accomplished by replacing the gluon links in the derivatives by averages over 1-, 3-, 5-, and 7-link paths. The other actions reduce taste changing even further by smearing more. In addition to the smearing, the Asqtad and HISQ actions include a three-hop term in the action (the “Naik term” [615]) to remove order $a^2$ errors in the dispersion relation, as well as a “Lepage term” [616] to cancel other order $a^2$ artefacts introduced by the smearing. In both the Asqtad and HISQ actions, the leading taste violations are of order $\alpha _S^2 a^2$, and “generic” lattices artefacts (those associated with discretisation errors other than taste violations) are of order $\alpha _S a^2$. The overall coefficients of these errors are, however, significantly smaller with HISQ than with Asqtad. With the Stout-smeared and HYP actions, the errors are formally larger (order $\alpha _S a^2$ for taste violations and order $a^2$ for generic lattices artefacts). Nevertheless, the smearing seems to be very efficient, and the actual size of errors at accessible lattice spacings appears to be at least as small as with HISQ.

Although logically distinct from the light-quark improvement program for these actions, it is customary with the HISQ action to include an additional correction designed to reduce discretisation errors for heavy quarks (in practice, usually charm quarks) [612]. The Naik term is adjusted to remove leading $(am_{c})^4$ and $\alpha _S(am_{c})^2$ errors, where $m_{c}$ is the charm quark mass and “leading” in this context means leading in powers of the heavy-quark velocity $v$ ($v/c\sim 1/3$ for $D_{s}$). With these improvements, the claim is that one can use the staggered action for charm quarks, although it must be emphasised that it is not obvious a priori how large a value of $am_{c}$ may be tolerated for a given desired accuracy, and this must be studied in the simulations.

*Ginsparg–Wilson fermions*

Fermionic lattice actions, which do not suffer from the doubling problem whilst preserving chiral symmetry go under the name of “Ginsparg–Wilson fermions”. In the continuum the massless Dirac operator ($D$) anticommutes with $\gamma _5$. At non-zero lattice spacing a chiral symmetry can be realised if this condition is relaxed to [617–619]

219 $$\begin{aligned} \left\{ D,\gamma _5\right\} = aD\gamma _5 D, \end{aligned}$$

which is now known as the Ginsparg–Wilson relation [312]. The Nielsen–Ninomiya theorem [620], which states that any lattice formulation for which $D$ anticommutes with $\gamma _5$ necessarily has doubler fermions, is circumvented since $\{D,\gamma _5\}\ne 0$.

A lattice Dirac operator which satisfies Eq. (219) can be constructed in several ways. The so-called “overlap” or Neuberger–Dirac operator [621] acts in four space-time dimensions and is, in its simplest form, defined by

220 $$\begin{aligned}&D_{N} = \frac{1}{\overline{a}} \left( 1-\epsilon (A)\right) ,\quad {\mathrm {where}}\quad \epsilon (A)\equiv A (A^\dagger A)^{-1/2},\nonumber \\&\quad A=1+s-aD_{w},\quad \overline{a}=\frac{a}{1+s}, \end{aligned}$$

$D_{w}$ is the massless Wilson-Dirac operator and $|s|<1$ is a tunable parameter. The overlap operator $D_{N}$ removes all doublers from the spectrum, and can readily be shown to satisfy the Ginsparg–Wilson relation. The occurrence of the sign function $\epsilon (A)$ in $D_{N}$ renders the application of $D_{N}$ in a computer program potentially very costly, since it must be implemented using, for instance, a polynomial approximation.

The most widely used approach to satisfying the Ginsparg–Wilson relation Eq. (219) in large-scale numerical simulations is provided by *Domain-Wall Fermions* (DWF) [622–624] and we therefore describe this in some more detail. Following early exploratory studies [625]. this approach has been developed into a practical formulation of lattice QCD with good chiral and flavour symmetries leading to results which contribute significantly to this review. In this formulation, the fermion fields $\psi (x,s)$ depend on a discrete fifth coordinate $s=1,\ldots ,N$ as well as the physical four-dimensional space-time coordinates $x_\mu ,\,\mu =1\dots 4$ (the gluon fields do not depend on $s$). The lattice on which the simulations are performed, is therefore a five-dimensional one of size $L^3\times T\times N$, where $L,\,T$ and $N$ represent the number of points in the spatial, temporal and fifth dimensions, respectively. The remarkable feature of DWF is that for each flavour there exists a physical light mode corresponding to the field $q(x)$:

221 $$\begin{aligned} q(x)&= \frac{1+\gamma ^5}{2}\psi (x,1)+\frac{1-\gamma ^5}{2}\psi (x,N)\end{aligned}$$

222 $$\begin{aligned} \bar{q}(x)&= \overline{\psi }(x,N)\frac{1+\gamma ^5}{2} +\overline{\psi }(x,1)\frac{1-\gamma ^5}{2}. \end{aligned}$$

The left and right-handed modes of the physical field are located on opposite boundaries in the five-dimensional space which, for $N\rightarrow \infty$, allows for independent transformations of the left and right components of the quark fields, that is, for chiral transformations. Unlike Wilson fermions, where for each flavour the quark mass parameter in the action is fine-tuned requiring a subtraction of contributions of $O(1/a)$ where $a$ is the lattice spacing, with DWF no such subtraction is necessary for the physical modes, whereas the unphysical modes have masses of $O(1/a)$ and decouple.

In actual simulations $N$ is finite and there are small violations of chiral symmetry which must be accounted for. The theoretical framework for the study of the residual breaking of chiral symmetry has been a subject of intensive investigation (for a review and references to the original literature see e.g. [626]). The breaking requires one or more *crossings* of the fifth dimension to couple the left and right-handed modes; the more crossings that are required the smaller the effect. For many physical quantities the leading effects of chiral symmetry breaking due to finite $N$ are parameterised by a *residual* mass, $m_{\mathrm {res}}$. For example, the PCAC relation (for degenerate quarks of mass $m$) $\partial _\mu A_\mu (x) = 2m P(x)$, where $A_\mu$ and $P$ represent the axial current and pseudoscalar density, respectively, is satisfied with $m=m^{\mathrm {DWF}}+m_\mathrm{res}$, where $m^{\mathrm {DWF}}$ is the bare mass in the DWF action. The mixing of operators which transform under different representations of chiral symmetry is found to be negligibly small in current simulations. The important thing to note is that the chiral symmetry-breaking effects are small and that there are techniques to mitigate their consequences.

The main price which has to be paid for the good chiral symmetry is that the simulations are performed in five dimensions, requiring approximately a factor of $N$ in computing resources and resulting in practice in ensembles at fewer values of the lattice spacing and quark masses than is possible with other formulations. The current generation of DWF simulations is being performed at physical quark masses so that ensembles with good chiral and flavour symmetries are being generated and analysed [25]. For a discussion of the equivalence of DWF and overlap fermions see [627, 628].

A third example of an operator which satisfies the Ginsparg–Wilson relation is the so-called fixed-point action [629–631]. This construction proceeds via a renormalisation group approach. A related formalism are the so-called “chirally improved” fermions [632].

*Smearing*

A simple modification which can help improve the action as well as the computational performance is the use of smeared gauge fields in the covariant derivatives of the fermionic action. Any smearing procedure is acceptable as long as it consists of only adding irrelevant (local) operators. Moreover, it can be combined with any discretisation of the quark action. The “Asqtad” staggered quark action mentioned above [611] is an example which makes use of so-called “Asqtad” smeared (or “fat”) links. Another example is the use of n-HYP-smeared [614, 633], stout-smeared [634, 635] or HEX (hypercubic stout) smeared [636] gauge links in the tree-level clover improved discretisation of the quark action, denoted by “n-HYP tlSW”, “stout tlSW” and “HEX tlSW” in the following.

In Table 40 we summarise the most widely used discretisations of the quark action and their main properties together with the abbreviations used in the summary tables. Note that in order to maintain the leading lattice artefacts of the actions as given in the table in non-spectral observables (like operator matrix elements) the corresponding non-spectral operators need to be improved as well.

**Table 40:** The most widely used discretisations of the quark action and some of their properties. Note that in order to maintain the leading lattice artefacts of the action in non-spectral observables (like operator matrix elements) the corresponding non-spectral operators need to be improved as well

Abbrev.	Discretisation	Leading lattice artefacts	Chiral symmetry	Remarks
Wilson	Wilson	$O(a)$	Broken	
tmWil	Twisted-mass Wilson	$O(a^2)$ at maximal twist	Broken	Flavour symmetry breaking: $(M_\text {PS}^{0})^2-(M_\text {PS}^\pm )^2\sim O(a^2)$
tlSW	Sheikholeslami–Wohlert	$O(g^2 a)$	Broken	Tree-level impr., $c_{\mathrm{sw}}=1$
n-HYP tlSW	Sheikholeslami–Wohlert	$O(g^2 a)$	Broken	Tree-level impr., $c_{\mathrm{sw}}=1$, n-HYP-smeared gauge links
Stout tlSW	Sheikholeslami–Wohlert	$O(g^2 a)$	Broken	Tree-level impr., $c_{\mathrm{sw}}=1$, stout-smeared gauge links
HEX tlSW	Sheikholeslami–Wohlert	$O(g^2 a)$	Broken	Tree-level impr., $c_{\mathrm{sw}}=1$, HEX-smeared gauge links
mfSW	Sheikholeslami–Wohlert	$O(g^2 a)$	Broken	Mean-field impr.
npSW	Sheikholeslami–Wohlert	$O(a^2)$	Broken	Non-perturbatively impr.
KS	Staggered	$O(a^2)$	U(1) $\otimes$ U(1) subgr. unbroken	Rooting for $N_\mathrm{f}<4$
Asqtad	Staggered	$O(a^2)$	U(1) $\otimes$ U(1) subgr. unbroken	Asqtad smeared gauge links, rooting for $N_\mathrm{f}<4$
HISQ	Staggered	$O(a^2)$	U(1) $\otimes$ U(1) subgr. unbroken	HISQ-smeared gauge links, rooting for $N_\mathrm{f}<4$
DW	Domain Wall	Asymptotically $O(a^2)$	Remnant breaking exponentially suppr.	Exact chiral symmetry and $O(a)$ impr. only in the limit $L_{s}\rightarrow \infty$
Overlap	Neuberger	$O(a^2)$	Exact	

#### A.1.3 Heavy-quark actions

Charm and bottom quarks are often simulated with different lattice-quark actions than up, down, and strange quarks because their masses are large relative to typical lattice spacings in current simulations; for example, $a m_{c} \sim 0.4$ and $am_{b} \sim 1.3$ at $a=0.06$ fm. Therefore, for the actions described in the previous section, using a sufficiently small lattice spacing to control generic $(am_{h})^n$ discretisation errors is computationally costly, and in fact prohibitive at the physical $b$-quark mass.

One approach for lattice heavy quarks is direct application of effective theory. In this case the lattice heavy-quark action only correctly describes phenomena in a specific kinematic regime, such as Heavy-Quark Effective Theory (HQET) [637–639] or Non-relativistic QCD (NRQCD) [640, 641]. One can discretise the effective Lagrangian to obtain, for example, Lattice HQET [642] or Lattice NRQCD [643, 644], and then simulate the effective theory numerically. The coefficients of the operators in the lattice-HQET and lattice-NRQCD actions are free parameters that must be determined by matching to the underlying theory (QCD) through the chosen order in $1/m_{h}$ or $v_{h}^2$, where $m_{h}$ is the heavy-quark mass and $v_{h}$ is the heavy-quark velocity in the heavy–light meson rest frame.

Another approach is to interpret a relativistic quark action such as those described in the previous section in a manner suitable for heavy quarks. One can extend the standard Symanzik improvement program, which allows one to systematically remove lattice cutoff effects by adding higher-dimension operators to the action, by allowing the coefficients of the dimension 4 and higher operators to depend explicitly upon the heavy-quark mass. Different prescriptions for tuning the parameters correspond to different implementations: those in common use are often called the Fermilab action [645], the relativistic heavy-quark action (RHQ) [646], and the Tsukuba formulation [647]. In the Fermilab approach, HQET is used to match the lattice theory to continuum QCD at the desired order in $1/m_{h}$.

More generally, effective theory can be used to estimate the size of cutoff errors from the various lattice heavy-quark actions. The power counting for the sizes of operators with heavy quarks depends on the typical momenta of the heavy quarks in the system. Bound-state dynamics differ considerably between heavy–heavy and heavy–light systems. In heavy–light systems, the heavy quark provides an approximately static source for the attractive binding force, like the proton in a hydrogen atom. The typical heavy-quark momentum in the bound-state rest frame is $|\vec {p}_{h}| \sim \Lambda _\mathrm{QCD}$, and heavy–light operators scale as powers of $(\Lambda _\mathrm{QCD}/m_{h})^n$. This is often called “HQET power-counting”, although it applies to heavy–light operators in HQET, NRQCD, and even relativistic heavy-quark actions described below. Heavy–heavy systems are similar to positronium or the deuteron, with the typical heavy-quark momentum $|\vec {p}_{h}| \sim \alpha _S m_{h}$. Therefore motion of the heavy quarks in the bound state rest frame cannot be neglected. Heavy–heavy operators have complicated power counting rules in terms of $v_{h}^2$ [644]; this is often called “NRQCD power counting.”

Alternatively, one can simulate bottom or charm quarks with the same action as up, down, and strange quarks provided that (1) the action is sufficiently improved, and (2) the lattice spacing is sufficiently fine. These qualitative criteria do not specify precisely how large a numerical value of $am_{h}$ can be allowed while obtaining a given precision for physical quantities; this must be established empirically in numerical simulations. At present, both the HISQ and twisted-mass Wilson actions discussed previously are being used to simulate charm quarks. Simulations with HISQ quarks have employed heavier quark masses than those with twisted-mass Wilson quarks because the action is more highly improved, but neither action can be used to simulate at the physical $am_{b}$ for current lattice spacings. Therefore calculations of heavy–light decay constants with these actions still rely on effective theory to reach the $b$-quark mass: the ETM Collaboration interpolates between twisted-mass Wilson data generated near $am_{c}$ and the static point [336], while the HPQCD Collaboration extrapolates HISQ data generated below $am_{b}$ up to the physical point using an HQET-inspired series expansion in $(1/m_{h})^n$ [366].

*Heavy-quark effective theory*

HQET was introduced by Eichten and Hill in Ref. [638]. It provides the correct asymptotic description of QCD correlation functions in the static limit $m_{h}/|\vec {p}_{h}| \rightarrow \infty$. Subleading effects are described by higher-dimensional operators whose coupling constants are formally of ${\mathcal O}((1/m_{h})^n)$. The HQET expansion works well for heavy–light systems in which the heavy-quark momentum is small compared to the mass.

The HQET Lagrangian density at the leading (static) order in the rest frame of the heavy quark is given by

223 $$\begin{aligned} {\mathcal L}^\mathrm{stat}(x) = \overline{\psi }_{h}(x) \,D_0\, \psi _{h}(x), \end{aligned}$$

with

224 $$\begin{aligned} P_+ \psi _{h} = \psi _{h}, \quad \quad \overline{\psi }_{h} P_+=\overline{\psi }_{h}, \quad \quad P_+={{1+\gamma _0}\over {2}}. \end{aligned}$$

A bare quark mass $m_\mathrm{bare}^\mathrm{stat}$ has to be added to the energy levels $E^\mathrm{stat}$ computed with this Lagrangian to obtain the physical ones. For example, the mass of the $B$-meson in the static approximation is given by

225 $$\begin{aligned} m_{B} = E^\mathrm{stat} + m_\mathrm{bare}^\mathrm{stat}. \end{aligned}$$

At tree level $m_\mathrm{bare}^\mathrm{stat}$ is simply the (static approximation of the) $b$-quark mass, but in the quantised lattice formulation it has to further compensate a divergence linear in the inverse lattice spacing. Weak composite fields are also rewritten in terms of the static fields, e.g.

226 $$\begin{aligned} A_0(x)^\mathrm{stat}=Z_{A}^\mathrm{stat} \left( \overline{\psi }(x) \gamma _0\gamma _5\psi _{h}(x)\right) , \end{aligned}$$

where the renormalisation factor of the axial current in the static theory $Z_{A}^\mathrm{stat}$ is scale-dependent. Recent lattice-QCD calculations using static $b$ quarks and dynamical light quarks [336, 406] perform the operator matching at one loop in mean-field improved lattice perturbation theory [648, 649]. Therefore the heavy-quark discretisation, truncation, and matching errors in these results are of ${\mathcal O}(a^2 \Lambda _\mathrm{QCD}^2)$, ${\mathcal O} (\Lambda _\mathrm{QCD}/m_{h})$ and ${\mathcal O}(\alpha _\mathrm{s}^2, \alpha _\mathrm{s}^2 a \Lambda _\mathrm{QCD})$.

In order to reduce heavy-quark truncation errors in $B$-meson masses and matrix elements to the few-percent level, state-of-the-art lattice-HQET computations now include corrections of ${\mathcal O}(1/m_{h})$. Adding the $1/m_{h}$ terms, the HQET Lagrangian reads

227 $$\begin{aligned}&{\mathcal L}^\mathrm{HQET}(x) \!=\! {\mathcal L}^\mathrm{stat}(x) \!-\!\omega _{\mathrm {kin}}{\mathcal {O}}_{\mathrm {kin}}(x)\!-\! \omega _{\mathrm {spin}}{\mathcal {O}}_{\mathrm {spin}}(x), \end{aligned}$$

228 $$\begin{aligned}&{\mathcal {O}}_{\mathrm {kin}}(x) \!=\! \overline{\psi }_{h}(x)\mathbf{D}^2\psi _{h}(x),\quad {\mathcal {O}}_{\mathrm {spin}}(x) \!=\! \overline{\psi }_{h}(x){\varvec{\sigma }} \cdot \mathbf{B}\psi _{h}(x).\nonumber \\ \end{aligned}$$

At this order, two other parameters appear in the Lagrangian, $\omega _{\mathrm {kin}}$ and $\omega _{\mathrm {spin}}$. The normalisation is such that the tree-level values of the coefficients are $\omega _{\mathrm {kin}}=\omega _{\mathrm {spin}}=1/(2m_{h})$. Similarly the operators are formally expanded in inverse powers of the heavy-quark mass. The time component of the axial current, relevant for the computation of mesonic decay constants is given by

229 $$\begin{aligned}&A_0^\mathrm{HQET}(x) = Z_{A}^\mathrm{HQET}\left( A_0^\mathrm{stat}(x) +\sum _{i=1}^2 c_{A}^{(i)} A_0^{(i)}(x)\right) , \end{aligned}$$

230 $$\begin{aligned}&A_0^{(1)}(x)=\overline{\psi }\frac{1}{2}\gamma _5 \gamma _k (\nabla _k-\overleftarrow{\nabla }_k)\psi _{h}(x), \quad k=1,2,3\end{aligned}$$

231 $$\begin{aligned}&A_0^{(2)} = -\partial _kA_k^\mathrm{stat}(x), \quad A_k^\mathrm{stat}=\overline{\psi }(x) \gamma _k\gamma _5\psi _{h}(x), \end{aligned}$$

and depends on two additional parameters $c_{A}^{(1)}$ and $c_{A}^{(2)}$.

A framework for non-perturbative HQET on the lattice has been introduced in [642, 650]. As pointed out in Refs. [651, 652], since $\alpha _\mathrm{s}(m_{h})$ decreases logarithmically with $m_{h}$, whereas corrections in the effective theory are power-like in $\Lambda /m_{h}$, it is possible that the leading errors in a calculation will be due to the perturbative matching of the action and the currents at a given order $(\Lambda /m_{h})^l$ rather than to the missing ${\mathcal O}((\Lambda /m_{h})^{l+1})$ terms. Thus, in order to keep matching errors below the uncertainty due to truncating the HQET expansion, the matching is performed non-perturbatively beyond leading order in $1/m_{h}$. The asymptotic convergence of HQET in the limit $m_{h} \rightarrow \infty$ indeed holds only in that case.

The higher-dimensional interaction terms in the effective Lagrangian are treated as space-time volume insertions into static correlation functions. For correlators of some multi-local fields ${\mathcal {O}}$ and up to the $1/m_{h}$ corrections to the operator, this means

232 $$\begin{aligned} {\langle }{\mathcal {O}} {\rangle }&= {\langle }{\mathcal {O}} \rangle _\mathrm{stat} +\omega _{\mathrm {kin}}a^4 \sum _x {\langle }{\mathcal {OO}}_\mathrm{kin}(x) \rangle _\mathrm{stat}\nonumber \\&+\, \omega _{\mathrm {spin}}a^4 \sum _x {\langle }{\mathcal {OO}}_\mathrm{spin}(x) \rangle _\mathrm{stat}, \end{aligned}$$

where ${\langle }{\mathcal {O}} \rangle _\mathrm{stat}$ denotes the static expectation value with ${\mathcal {L}}^\mathrm{stat}(x) +{\mathcal {L}}^\mathrm{light}(x)$. Non-perturbative renormalisation of these correlators guarantees the existence of a well-defined continuum limit to any order in $1/m_{h}$. The parameters of the effective action and operators are then determined by matching a suitable number of observables calculated in HQET (to a given order in $1/m_{h}$) and in QCD in a small volume (typically with $L\simeq 0.5$ fm), where the full relativistic dynamics of the $b$-quark can be simulated and the parameters can be computed with good accuracy. In [650, 653] the Schrödinger Functional (SF) setup has been adopted to define a set of quantities, given by the small volume equivalent of decay constants, pseudoscalar–vector splittings, effective masses and ratio of correlation functions for different kinematics, which can be used to implement the matching conditions. The kinematical conditions are usually modified by changing the periodicity in space of the fermions, i.e. by directly exploiting a finite-volume effect. The new scale $L$, which is introduced in this way, is chosen such that higher orders in $1/m_{h}L$ and in $\Lambda _\mathrm{QCD}/m_{h}$ are of about the same size. At the end of the matching step the parameters are known at lattice spacings which are of the order of $0.01$ fm, significantly smaller than the resolutions used for large volume, phenomenological, applications. For this reason a set of SF-step scaling functions is introduced in the effective theory to evolve the parameters to larger lattice spacings. The whole procedure yields the non-perturbative parameters with an accuracy which allows to compute phenomenological quantities with a precision of a few percent (see [370, 390] for the case of the $B_{(s)}$ decay constants). Such an accuracy cannot be achieved by performing the non-perturbative matching in large volume against experimental measurements, which in addition would reduce the predictivity of the theory. For the lattice-HQET action matched non-perturbatively through ${\mathcal O}(1/m_{h})$, discretisation and truncation errors are of ${\mathcal O}(a \Lambda ^2_\mathrm{QCD}/m_{h}, a^2 \Lambda ^2_\mathrm{QCD})$ and ${\mathcal {O}}((\Lambda _\mathrm{QCD}/m_{h} )^2)$.

The noise-to-signal ratio of static-light correlation functions grows exponentially in Euclidean time, $\propto e^{\mu x_0}$. The rate $\mu$ is non-universal but diverges as $1/a$ as one approaches the continuum limit. By changing the discretisation of the covariant derivative in the static action one may achieve an exponential reduction of the noise-to-signal ratio. Such a strategy led to the introduction of the $S^\mathrm{stat}_\mathrm{HYP1,2}$ actions [654], where the thin links in $D_0$ are replaced by HYP-smeared links [614]. These actions are now used in all lattice applications of HQET.

*Non-relativistic QCD*

Non-relativistic QCD (NRQCD) [643, 644] is an effective theory that can be matched to full QCD order by order in the heavy-quark velocity $v_{h}^2$ (for heavy–heavy systems) or in $\Lambda _\mathrm{QCD}/m_{h}$ (for heavy–light systems) and in powers of $\alpha _\mathrm{s}$. Relativistic corrections appear as higher-dimensional operators in the Hamiltonian.

As an effective field theory, NRQCD is only useful with an ultraviolet cutoff of order $m_{h}$ or less. On the lattice this means that it can be used only for $am_{h}>1$, which means that $O(a^n)$ errors cannot be removed by taking $a\rightarrow 0$ at fixed $m_{h}$. Instead heavy-quark discretisation errors are systematically removed by adding additional operators to the lattice Hamiltonian. Thus, while strictly speaking no continuum limit exists at fixed $m_{h}$, continuum physics can be obtained at finite lattice spacing to arbitrarily high precision provided enough terms are included, and provided that the coefficients of these terms are calculated with sufficient accuracy. Residual discretisation errors can be parameterised as corrections to the coefficients in the non-relativistic expansion, as shown in Eq. (235). Typically they are of the form $(a|\vec {p}_{h}|)^n$ multiplied by a function of $am_{h}$ that is smooth over the limited range of heavy-quark masses (with $am_{h} > 1$) used in simulations, and can therefore can be represented by a low-order polynomial in $am_{h}$ by Taylor’s theorem (see Ref. [364] for further discussion). Power-counting estimates of these effects can be compared to the observed lattice spacing dependence in simulations. Provided that these effects are small, such comparisons can be used to estimate and correct the residual discretisation effects.

An important feature of the NRQCD approach is that the same action can be applied to both heavy–heavy and heavy–light systems. This allows, for instance, the bare $b$-quark mass to be fixed via experimental input from $\Upsilon$ so that simulations carried out in the $B$ or $B_{s}$ systems have no adjustable parameters left. Precision calculations of the $B_{s}$-meson mass (or of the mass splitting $M_{B_{s}} - M_\Upsilon /2$) can then be used to test the reliability of the method before turning to quantities one is trying to predict, such as decay constants $f_B$ and $f_{B_{s}}$, semileptonic form factors or neutral $B$ mixing parameters.

Given the same lattice-NRQCD heavy-quark action, simulation results will not be as accurate for charm quarks as for bottom ($1/m_{b} < 1/m_{c}$, and $v_{b} < v_{c}$ in heavy–heavy systems). For charm, however, a more serious concern is the restriction that $am_{h}$ must be greater than one. This limits lattice-NRQCD simulations at the physical $am_{c}$ to relatively coarse lattice spacings for which light-quark and gluon discretisation errors could be large. Thus recent lattice-NRQCD simulations have focussed on bottom quarks because $am_{b} > 1$ in the range of typical lattice spacings between $\approx$ 0.06 and 0.15 fm.

In most simulations with NRQCD $b$-quarks during the past decade one has worked with an NRQCD action that includes tree-level relativistic corrections through ${\mathcal {O}}(v_{h}^4)$ and discretisation corrections through ${\mathcal {O}}(a^2)$,

233 $$\begin{aligned}&S_\mathrm{NRQCD}\! =\! a^4 \sum _x \left\{ {\Psi }^\dagger _{t} \Psi _{t} \!-\!{\Psi }^\dagger _{t}\left( \!1 \!-\!\frac{a \delta H}{2}\!\right) _{t}\left( \!1\!-\! \frac{aH_0}{2n}\!\right) ^{n}_{t} \nonumber \right. \\&\quad \times \left. U^\dagger _{t}(t\!-\!a)\left( \!1 \!-\! \frac{aH_0}{2n}\!\right) ^{n}_{t-a}\left( \!1 \!-\! \frac{a\delta H}{2}\!\right) _{t-a} \Psi _{t-a}\right\} , \end{aligned}$$

where the subscripts “$t$” and “$t\!-\!a$” denote that the heavy-quark, gauge, $\mathbf {E}$ and $\mathbf {B}$-fields are on time slices $t$ or $t\!-\!a$, respectively. $H_0$ is the non-relativistic kinetic energy operator,

234 $$\begin{aligned} H_0 = - {\Delta ^{(2)}\over 2m_{h}}, \end{aligned}$$

and $\delta H$ includes relativistic and finite-lattice-spacing corrections,

235 $$\begin{aligned} \delta H&= - c_1\,\frac{(\Delta ^{(2)})^2}{8m_{h}^3} + c_2\,\frac{i g}{8m_{h}^2}\left( \mathbf{\nabla }\cdot \tilde{\mathbf{E}}- \tilde{\mathbf{E}}\cdot \mathbf{\nabla }\right) \nonumber \\&- c_3\,\frac{g}{8m_{h}^2} \varvec{{\sigma }}\cdot (\tilde{\mathbf{\nabla }}\times \tilde{\mathbf{E}}- \tilde{\mathbf{E}}\times \tilde{\mathbf{\nabla }})\nonumber \\&- c_4\,\frac{g}{2m_{h}}\,\varvec{{\sigma }}\cdot \tilde{\mathbf{B}}+ c_5\,\frac{a^2{\Delta ^{(4)}}}{24m_{h}} - c_6\,\frac{a(\Delta ^{(2)})^2}{16nm_{h}^2}. \end{aligned}$$

$m_{h}$ is the bare heavy-quark mass, $\Delta ^{(2)}$ the lattice Laplacian, $\mathbf{\nabla }$ the symmetric lattice derivative and ${\Delta ^{(4)}}$ the lattice discretisation of the continuum $\sum _i D^4_i$. $\tilde{\mathbf{\nabla }}$ is the improved symmetric lattice derivative and the $\tilde{\mathbf{E}}$ and $\tilde{\mathbf{B}}$ fields have been improved beyond the usual clover leaf construction. The stability parameter $n$ is discussed in [644]. In most cases the $c_i$’s have been set equal to their tree-level values $c_i = 1$. With this implementation of the NRQCD action, errors in heavy–light meson masses and splittings are of ${\mathcal {O}}(\alpha _S \Lambda _\mathrm{QCD}/m_{h} )$, ${\mathcal {O}}(\alpha _S (\Lambda _\mathrm{QCD}/m_{h})^2 )$, ${\mathcal {O}}((\Lambda _\mathrm{QCD}/m_{h} )^3)$ and ${\mathcal {O}}(\alpha _\mathrm{s} a^2 \Lambda _\mathrm{QCD}^2)$, with coefficients that are functions of $am_{h}$. One-loop corrections to many of the coefficients in Eq. (235) have now been calculated, and they are starting to be included in simulations [383, 655, 656].

Most of the operator matchings involving heavy–light currents or four-fermion operators with NRQCD $b$-quarks and AsqTad or HISQ light quarks have been carried out at one-loop order in lattice perturbation theory. In calculations published to date of electroweak matrix elements, heavy–light currents with massless light quarks have been matched through ${\mathcal {O}}(\alpha _\mathrm{s}, \Lambda _\mathrm{QCD}/m_{h}, \alpha _\mathrm{s}/(a m_{h}), \alpha _\mathrm{s} \Lambda _\mathrm{QCD}/m_{h})$ and four-fermion operators through ${\mathcal {O}}(\alpha _\mathrm{s}, \Lambda _\mathrm{QCD}/m_{h}, \alpha _\mathrm{s}/(a m_{h}))$. NRQCD/HISQ currents with massive HISQ quarks are also of interest, e.g. for the bottom-charm currents in $B \rightarrow D^{(*)}, l \nu$ semileptonic decays and the relevant matching calculations have been performed at one-loop order in Ref. [657]. Taking all the above into account, the most significant systematic error in electroweak matrix elements published to date with NRQCD $b$-quarks is the ${\mathcal {O}}(\alpha _\mathrm{s}^2)$ perturbative matching uncertainty. Work is therefore under way to use current–current correlator methods combined with very high-order continuum perturbation theory to do current matchings non-perturbatively [658].

*Relativistic heavy quarks*

An approach for relativistic heavy-quark lattice formulations was first introduced by El-Khadra et al. in Ref. [645]. Here they showed that, for a general lattice action with massive quarks and non-Abelian gauge fields, discretisation errors can be factorised into the form $f(m_{h} a)(a|\vec {p}_{h}|)^n$, and that the function $f(m_{h} a)$ is bounded to be of ${\mathcal O}(1)$ or less for all values of the quark mass $m_{h}$. Therefore cutoff effects are of ${\mathcal O}(a \Lambda _\mathrm{QCD})^n$ and ${\mathcal O}((a|\vec {p}_{h}|)^n)$, even for $am_{h} \gtrsim 1$, and they can be controlled using a Symanzik-like procedure. As in the standard Symanzik improvement program, cutoff effects are systematically removed by introducing higher-dimension operators to the lattice action and suitably tuning their coefficients. In the relativistic heavy-quark approach, however, the operator coefficients are allowed to depend explicitly on the quark mass. By including lattice operators through dimension $n$ and adjusting their coefficients $c_{n,i}(m_{h} a)$ correctly, one enforces that matrix elements in the lattice theory are equal to the analogous matrix elements in continuum QCD through $(a|\vec {p}_{h}|)^n$, such that residual heavy-quark discretisation errors are of ${\mathcal O}(a|\vec {p}_{h}|)^{n+1}$.

The relativistic heavy-quark approach can be used to compute the matrix elements of states containing heavy quarks for which the heavy-quark spatial momentum $|\vec {p}_{h}|$ is small compared to the lattice spacing. Thus it is suitable to describe bottom and charm quarks in both heavy–light and heavy–heavy systems. Calculations of bottomonium and charmonium spectra serve as non-trivial tests of the method and its accuracy.

At fixed lattice spacing, relativistic heavy-quark formulations recover the massless limit when $(am_{h}) \ll 1$, recover the static limit when $(am_{h}) \gg 1$, and smoothy interpolate between the two; thus they can be used for any value of the quark mass, and, in particular, for both charm and bottom. Discretisation errors for relativistic heavy-quark formulations are generically of the form $\alpha _\mathrm{s}^k f(am_{h})(a |\vec {p}_{h}|)^n$, where $k$ reflects the order of the perturbative matching for operators of ${\mathcal O}((a |\vec {p}_{h}|)^n)$. For each $n$, such errors are removed completely if the operator matching is non-perturbative. When $(am_{h}) \sim 1$, this gives rise to non-trivial lattice-spacing dependence in physical quantities, and it is prudent to compare estimates based on power-counting with a direct study of scaling behaviour using a range of lattice spacings. At fixed quark mass, relativistic heavy-quark actions possess a smooth continuum limit without power divergences. Of course, as $m_{h} \rightarrow \infty$ at fixed lattice spacing, the power divergences of the static limit are recovered (see, e.g. Ref. [659]).

The relativistic heavy-quark formulations in use all begin with the anisotropic Sheikholeslami–Wohlert (“clover”) action [660]:

236 $$\begin{aligned} S_\mathrm{lat}&= a^4 \sum _{x,x'} \bar{\psi }(x') \left( m_0 + \gamma _0 D_0 + \zeta \vec {\gamma } \cdot \vec {D} - \frac{a}{2} (D^0)^2\right. \nonumber \\&\left. - \frac{a}{2} \zeta (\vec {D})^2+ \sum _{\mu ,\nu } \frac{ia}{4} c_\mathrm{SW} \sigma _{\mu \nu } F_{\mu \nu } \right) _{x' x} \psi (x), \end{aligned}$$

where $D_\mu$ is the lattice covariant derivative and $F_{\mu \nu }$ is the lattice field-strength tensor. Here we show the form of the action given in Ref. [646]. The introduction of a space-time anisotropy, parameterised by $\zeta$ in Eq. (236), is convenient for heavy-quark systems because the characteristic heavy-quark four-momenta do not respect space-time axis exchange ($\vec {p}_{h} < m_{h}$ in the bound-state rest frame). Further, the Sheikoleslami–Wohlert action respects the continuum heavy-quark spin and flavour symmetries, so HQET can be used to interpret and estimate lattice-discretisation effects [659, 661, 662]. We discuss three different prescriptions for tuning the parameters of the action in common use below. In particular, we focus on aspects of the action and operator improvement and matching relevant for evaluating the quality of the calculations discussed in the main text.

The meson energy-momentum dispersion relation plays an important role in relativistic heavy-quark formulations:

237 $$\begin{aligned} E(\vec {p}) = M_1 + \frac{\vec {p}^2}{2M_2} + {\mathcal O}(\vec {p}^4), \end{aligned}$$

where $M_1$ and $M_2$ are known as the rest and kinetic masses, respectively. Because the lattice breaks Lorentz invariance, there are corrections proportional to powers of the momentum. Further, the lattice rest masses and kinetic masses are not equal ($M_1 \ne M_2$), and only become equal in the continuum limit.

The Fermilab interpretation [645] is suitable for calculations of mass splittings and matrix elements of systems with heavy quarks. The Fermilab action is based on the hopping-parameter form of the Wilson action, in which $\kappa _{h}$ parameterises the heavy-quark mass. In practice, $\kappa _{h}$ is tuned such that the kinetic meson mass equals the experimentally measured heavy-strange meson mass ($m_{B_{s}}$ for bottom and $m_{D_{s}}$ for charm). In principle, one could also tune the anisotropy parameter such that $M_1 = M_2$. This is not necessary, however, to obtain mass splittings and matrix elements, which are not affected by $M_1$ [661]. Therefore in the Fermilab action the anisotropy parameter is set equal to unity. The clover coefficient in the Fermilab action is fixed to the value $c_\mathrm{SW} = 1/u_0^3$ from mean-field improved lattice perturbation theory [516]. With this prescription, discretisation effects are of ${\mathcal O}(\alpha _\mathrm{s}a|\vec {p}_{h}|, (a|\vec {p}_{h}|)^2)$. Calculations of electroweak matrix elements also require improving the lattice current and four-fermion operators to the same order, and matching them to the continuum. Calculations with the Fermilab action remove tree-level ${\mathcal O}(a)$ errors in electroweak operators by rotating the heavy-quark field used in the matrix element and setting the rotation coefficient to its tadpole-improved tree-level value (see e.g. Eqs. (7.8) and (7.10) of Ref. [645]). Finally, electroweak operators are typically renormalised using a mostly non-perturbative approach in which the flavour-conserving light–light and heavy–heavy current renormalisation factors $Z_V^{ll}$ and $Z_V^{hh}$ are computed non-perturbatively [663]. The flavour-conserving factors account for most of the heavy–light current renormalisation. The remaining correction is expected to be close to unity due to the cancellation of most of the radiative corrections including tadpole graphs [659]; therefore it can be reliably computed at one loop in mean-field improved lattice perturbation theory with truncation errors at the percent to few-percent level.

The relativistic heavy-quark (RHQ) formulation developed by Li et al. builds upon the Fermilab approach, but it tunes all the parameters of the action in Eq. (236) non-perturbatively [646]. In practice, the three parameters $\{m_0a, c_\mathrm{SW}, \zeta \}$ are fixed to reproduce the experimentally measured $B_{s}$-meson mass and hyperfine splitting ($m_{B_{s}^*}-m_{B_{s}}$), and to make the kinetic and rest masses of the lattice $B_{s}$-meson equal [385]. This is done by computing the heavy-strange meson mass, hyperfine splitting, and ratio $M_1/M_2$ for several sets of bare parameters $\{m_0a, c_\mathrm{SW}, \zeta \}$ and interpolating linearly to the physical $B_{s}$ point. By fixing the $B_{s}$-meson hyperfine splitting, one loses a potential experimental prediction with respect to the Fermilab formulation. However, by requiring that $M_1 = M_2$, one gains the ability to use the meson rest masses, which are generally more precise than the kinetic masses, in the RHQ approach. The non-perturbative parameter-tuning procedure eliminates ${\mathcal O}(a)$ errors from the RHQ action, such that discretisation errors are of ${\mathcal O}((a|\vec {p}_{h}|)^2)$. Calculations of $B$-meson decay constants and semileptonic form factors with the RHQ action are in progress [409, 434], as is the corresponding one-loop mean-field improved lattice perturbation theory [664]. For these works, cutoff effects in the electroweak vector and axial-vector currents will be removed through ${\mathcal O}(\alpha _\mathrm{s} a)$, such that the remaining discretisation errors are of ${\mathcal O}(\alpha _\mathrm{s}^2a|\vec {p}_{h}|, (a|\vec {p}_{h}|)^2)$. Matching the lattice operators to the continuum will be done following the mostly non-perturbative approach described above.

The Tsukuba heavy-quark action is also based on the Sheikholeslami–Wohlert action in Eq. (236), but it allows for further anisotropies and hence has additional parameters: specifically the clover coefficients in the spatial $(c_B)$ and temporal $(c_E)$ directions differ, as do the anisotropy coefficients of the $\vec {D}$ and $\vec {D}^2$ operators [647]. In practice, the contribution to the clover coefficient in the massless limit is computed non-perturbatively [665], while the mass-dependent contributions, which differ for $c_B$ and $c_E$, are calculated at one loop in mean-field improved lattice perturbation theory [666]. The hopping parameter is fixed non-perturbatively to reproduce the experimentally measured spin-averaged $1S$ charmonium mass [333]. One of the anisotropy parameters ($r_{t}$ in Ref. [333]) is also set to its one-loop perturbative value, while the other ($\nu$ in Ref. [333]) is fixed non-perturbatively to obtain the continuum dispersion relation for the spin-averaged charmonium $1S$ states (such that $M_1 = M_2$). For the renormalisation and improvement coefficients of weak current operators, the contributions in the chiral limit are obtained non-perturbatively [21, 667], while the mass-dependent contributions are estimated using one-loop lattice perturbation theory [668]. With these choices, lattice cutoff effects from the action and operators are of ${\mathcal O}(\alpha _\mathrm{s}^2 a|\vec {p}|, (a|\vec {p}_{h}|)^2)$.

*Light-quark actions combined with HQET*

The heavy-quark formulations discussed in the previous sections use effective field theory to avoid the occurrence of discretisation errors of the form $(am_{h})^n$. In this section we describe methods that use improved actions that were originally designed for light-quark systems for $B$ physics calculations. Such actions unavoidably contain discretisation errors that grow as a power of the heavy-quark mass. In order to use them for heavy-quark physics, they must be improved to at least ${\mathcal {O}}(am_{h})^2$. However, since $am_{b} > 1$ at the smallest lattice spacings available in current simulations, these methods also require input from HQET to guide the simulation results to the physical $b$-quark mass.

The ETM collaboration has developed two methods, the “ratio method” [392] and the “interpolation method” [669, 670]. They use these methods together with simulations with twisted-mass Wilson fermions, which have discretisation errors of $O(am_{h})^2$. In the interpolation method $\Phi _{hs}$ and $\Phi _{h\ell }$ (or $\Phi _{hs}/\Phi _{h\ell }$) are calculated for a range of heavy-quark masses in the charm region and above, while roughly keeping $am_{h} \lesssim 0.5$. The relativistic results are combined with a separate calculation of the decay constants in the static limit, and then interpolated to the physical $b$ quark mass. In ETM’s implementation of this method, the heavy Wilson decay constants are matched to HQET using NLO in continuum perturbation theory. The static limit result is renormalised using one-loop mean-field improved lattice perturbation theory, while for the relativistic data PCAC is used to calculate absolutely normalised matrix elements. Both, the relativistic and static limit data are then run to the common reference scale $\mu _{b} = 4.5 \,{\mathrm {GeV}}$ at NLO in continuum perturbation theory. In the ratio method, one constructs physical quantities $P(m_{h})$ from the relativistic data that have a well-defined static limit ($P(m_{h}) \rightarrow$ const. for $m_{h} \rightarrow \infty$) and evaluates them at the heavy-quark masses used in the simulations. Ratios of these quantities are then formed at a fixed ratio of heavy quark masses, $z = P(m_{h}) / P(m_{h}/\lambda )$ (where $1 < \lambda \lesssim 1.3$), which ensures that $z$ is equal to unity in the static limit. Hence, a separate static limit calculation is not needed with this method. In ETM’s implementation of the ratio method for the $B$-meson decay constant, $P(m_{h})$ is constructed from the decay constants and the heavy-quark pole mass as $P(m_{h}) = f_{h\ell }(m_{h}) \cdot (m^\mathrm{pole}_{h})^{1/2}$. The corresponding $z$-ratio therefore also includes ratios of perturbative matching factors for the pole mass to ${\overline{\mathrm{MS}}}$ conversion. For the interpolation to the physical $b$-quark mass, ratios of perturbative matching factors converting the data from QCD to HQET are also included. The QCD-to-HQET matching factors improve the approach to the static limit by removing the leading-logarithmic corrections. In ETM’s implementation of this method (ETM 11 and 12) both conversion factors are evaluated at NLO in continuum perturbation theory. The ratios are then simply fit to a polynomial in $1/m_{h}$ and interpolated to the physical $b$-quark mass. The ratios constructed from $f_{h\ell }$ ($f_{hs}$) are called $z$ ($z_{s}$). In order to obtain the $B$-meson decay constants, the ratios are combined with relativistic decay-constant data evaluated at the smallest reference mass.

The HPQCD collaboration has introduced a method in Ref. [366] which we shall refer to as the “heavy HISQ” method. The first key ingredient is the use of the HISQ action for the heavy and light valence quarks, which has leading discretisation errors of ${\mathcal {O}} (\alpha _\mathrm{s} (v/c) (am_{h})^2, (v/c)^2 (am_{h})^4)$. With the same action for the heavy and light valence quarks it is possible to use PCAC to avoid renormalisation uncertainties. Another key ingredient is the availability of gauge ensembles over a large range of lattice spacings, in this case in the form of the library of $N_\mathrm{f} = 2+1$ asqtad ensembles made public by the MILC collaboration which includes lattice spacings as small as $a \approx 0.045$ fm. Since the HISQ action is so highly improved and with lattice spacings as small as 0.045 fm, HPQCD is able to use a large range of heavy-quark masses, from below the charm region to almost up to the physical $b$ quark mass with $am_{h} \lesssim 0.85$. They then fit their data in a combined continuum and HQET fit (i.e. using a fit function that is motivated by HQET) to a polynomial in $1/m_H$ (the heavy pseudo scalar meson mass of a meson containing a heavy ($h$) quark).

In Table 41 we list the discretisations of the quark action most widely used for heavy $c$ and $b$ quarks together with the abbreviations used in the summary tables. We also summarise the main properties of these actions and the leading lattice-discretisation errors for calculations of heavy–light meson matrix quantities with them. Note that in order to maintain the leading lattice artefacts of the actions as given in the table in non-spectral observables (like operator matrix elements) the corresponding non-spectral operators need to be improved as well.

**Table 41:** Discretisations of the quark action most widely used for heavy $c$ and $b$ quarks and some of their properties

Abbrev.	Discretisation	Leading lattice artefacts and truncation errors for heavy–light mesons	Remarks
tmWil	Twisted-mass Wilson	${\mathcal {O}}((am_{h})^{2})$	PCAC relation for axial-vector current
HISQ	Staggered	${\mathcal {O}}(\alpha _{S} (am_{h})^{2} (v/c), (am_{h})^{4} (v/c)^{2})$	PCAC relation for axial-vector current; Ward identity for vector current
Static	Static effective action	${\mathcal {O}}( a^{2} \Lambda _{{\mathrm{QCD}}}^{2}, \Lambda _{{\mathrm{QCD}}}/m_{h}, \alpha _\mathrm{s}^{2}, \alpha _\mathrm{s}^{2} a \Lambda _{\mathrm{QCD}})$	Implementations use APE, HYP1, and HYP2 smearing
HQET	Heavy-Quark Effective Theory	${\mathcal {O}}( a \Lambda ^{2}_{\mathrm{QCD}}/m_{h}, a^{2} \Lambda ^{2}_{\mathrm{QCD}}, (\Lambda _{\mathrm{QCD}}/m_{h})^{2})$	Non-perturbative matching through ${\mathcal {O}}(1/m_{h})$
NRQCD	Non-relativistic QCD	$\mathcal{O}(\alpha _{S} \Lambda _{\mathrm{QCD}}/m_{h}, \alpha _{S} (\Lambda _{\mathrm{QCD}}/m_{h})^{2} ,(\Lambda _{\mathrm{QCD}}/m_{h} )^{3}, \alpha _\mathrm{s} a^{2} \Lambda _{\mathrm{QCD}}^{2})$	Tree-level relativistic corrections through $\mathcal{O}(v_{h}^{4})$ and discretisation corrections through $\mathcal{O}(a^{2})$
Fermilab	Sheikholeslami–Wohlert	${\mathcal {O}}(\alpha _{sa}\Lambda _{\mathrm{QCD}}, (a\Lambda _{\mathrm{QCD}})^{2})$	Hopping parameter tuned non-perturbatively; clover coefficient computed at tree level in mean-field improved lattice perturbation theory
Tsukuba	Sheikholeslami–Wohlert	${\mathcal {O}}( \alpha _\mathrm{s}^{2} a\Lambda _{\mathrm{QCD}}, (a\Lambda _{\mathrm{QCD}})^{2})$	NP clover coefficient at $ma=0$ plus mass-dependent corrections calculated at one loop in lattice perturbation theory; $\nu$ calculated NP from dispersion relation; $r_{s}$ calculated at one loop in lattice perturbation theory

### A.2 Setting the scale

In simulations of lattice QCD quantities such as hadron masses and decay constants are obtained in “lattice units” i.e. as dimensionless numbers. In order to convert them into physical units they must be expressed in terms of some experimentally known, dimensionful reference quantity $Q$. This procedure is called “setting the scale”. It amounts to computing the non-perturbative relation between the bare gauge coupling $g_0$ (which is an input parameter in any lattice simulation) and the lattice spacing $a$ expressed in physical units. To this end one chooses a value for $g_0$ and computes the value of the reference quantity in a simulation: This yields the dimensionless combination, $(aQ)|_{g_0}$, at the chosen value of $g_0$. The calibration of the lattice spacing is then achieved via

238 $$\begin{aligned} a^{-1}\,(\mathrm{MeV}) = \frac{Q|_\mathrm{{exp}}\,(\mathrm{MeV})}{(aQ)|_{g_0}}, \end{aligned}$$

where $Q|_\mathrm{{exp}}$ denotes the experimentally known value of the reference quantity. Common choices for $Q$ are the mass of the nucleon, the $\Omega$ baryon or the decay constants of the pion and the kaon. Vector mesons, such as the $\rho$ or $K^*$-meson, are unstable and therefore their masses are not very well suited for setting the scale, despite the fact that they have been used over many years for that purpose.

Another widely used quantity to set the scale is the hadronic radius $r_0$, which can be determined from the force between static quarks via the relation [65]

239 $$\begin{aligned} F(r_0)r_0^2 = 1.65. \end{aligned}$$

If the force is derived from potential models describing heavy quarkonia, the above relation determines the value of $r_0$ as $r_0\approx 0.5$ fm. A variant of this procedure is obtained [473] by using the definition $F(r_1)r_1^2=1.00$, which yields $r_1\approx 0.32$ fm. It is important to realise that both $r_0$ and $r_1$ are not directly accessible in experiment, so that their values derived from phenomenological potentials are necessarily model-dependent. Inspite of the inherent ambiguity whenever hadronic radii are used to calibrate the lattice spacing, they are very useful quantities for performing scaling tests and continuum extrapolations of lattice data. Furthermore, they can be easily computed with good statistical accuracy in lattice simulations.

### A.3 Matching and running

The lattice formulation of QCD amounts to introducing a particular regularisation scheme. Thus, in order to be useful for phenomenology, hadronic matrix elements computed in lattice simulations must be related to some continuum reference scheme, such as the ${\overline{\mathrm{MS}}}$-scheme of dimensional regularisation. The matching to the continuum scheme usually involves running to some reference scale using the renormalisation group.

In principle, the matching factors which relate lattice matrix elements to the ${\overline{\mathrm{MS}}}$-scheme can be computed in perturbation theory formulated in terms of the bare coupling. It has been known for a long time, though, that the perturbative expansion is not under good control. Several techniques have been developed which allow for a non-perturbative matching between lattice regularisation and continuum schemes, and they are briefly introduced here.

*Regularisation-independent momentum subtraction*

In the *Regularisation-independent momentum subtraction* (“RI/MOM” or “RI”) scheme [297] a non-perturbative renormalisation condition is formulated in terms of Green functions involving quark states in a fixed gauge (usually Landau gauge) at non-zero virtuality. In this way one relates operators in lattice regularisation non-perturbatively to the RI scheme. In a second step one matches the operator in the RI scheme to its counterpart in the ${\overline{\mathrm{MS}}}$-scheme. The advantage of this procedure is that the latter relation involves perturbation theory formulated in the continuum theory. The uncontrolled use of lattice perturbation theory can thus be avoided. A technical complication is associated with the accessible momentum scales (i.e. virtualities), which must be large enough (typically several $\mathrm{GeV}$) in order for the perturbative relation to ${\overline{\mathrm{MS}}}$ to be reliable. The momentum scales in simulations must stay well below the cutoff scale (i.e. $2\pi$ over the lattice spacing), since otherwise large lattice artefacts are incurred. Thus, the applicability of the RI scheme traditionally relies on the existence of a “window” of momentum scales, which satisfy

240 $$\begin{aligned} \Lambda _\mathrm{QCD} \lesssim p \lesssim 2\pi a^{-1}. \end{aligned}$$

However, solutions for mitigating this limitation, which involve continuum limit, non-perturbative running to higher scales in the RI/MOM scheme, have recently been proposed and implemented [22, 23, 315, 671].

*Schrödinger functional*

Another example of a non-perturbative matching procedure is provided by the Schrödinger functional (SF) scheme [87]. It is based on the formulation of QCD in a finite volume. If all quark masses are set to zero the box length remains the only scale in the theory, such that observables like the coupling constant run with the box size $L$. The great advantage is that the RG running of scale-dependent quantities can be computed non-perturbatively using recursive finite-size scaling techniques. It is thus possible to run non-perturbatively up to scales of, say, $100\,\mathrm{GeV}$, where one is sure that the perturbative relation between the SF and ${\overline{\mathrm{MS}}}$-schemes is controlled.

*Perturbation theory*

The third matching procedure is based on perturbation theory in which higher order are effectively resummed [516]. Although this procedure is easier to implement, it is hard to estimate the uncertainty associated with it.

*Mostly non-perturbative renormalisation*

Some calculations of heavy–light and heavy–heavy matrix elements adopt a mostly non-perturbative matching approach. Let us consider a weak decay process mediated by a current with quark flavours $h$ and $q$, where $h$ is the initial heavy quark (either bottom or charm) and $q$ can be a light ($\ell = u,d$), strange, or charm quark. The matrix elements of lattice current $J_{hq}$ are matched to the corresponding continuum matrix elements with continuum current $\mathcal{J}_{hq}$ by calculating the renormalisation factor $Z_{J_{hq}}$. The mostly non-perturbative renormalisation method takes advantage of rewriting the current renormalisation factor as the following product:

241 $$\begin{aligned} Z_{J_{hq}} = \rho _{J_{hq}} \sqrt{Z_{V^4_{hh}}Z_{V^4_{qq}}} \end{aligned}$$

The flavour-conserving renormalisation factors $Z_{V^4_{hh}}$ and $Z_{V^4_{qq}}$ can be obtained non-perturbatively from standard heavy–light and light–light meson charge normalisation conditions. $Z_{V^4_{hh}}$ and $Z_{V^4_{qq}}$ account for the bulk of the renormalisation. The remaining correction $\rho _{J_{hq}}$ is expected to be close to unity because most of the radiative corrections, including self-energy corrections and contributions from tadpole graphs, cancel in the ratio [659, 662]. The one-loop coefficients of $\rho _{J_{hq}}$ have been calculated for heavy–light and heavy–heavy currents for Fermilab heavy and both (improved) Wilson light [659, 662] and asqtad light [672] quarks. In all cases the one-loop coefficients are found to be very small, yielding sub-percent to few percent level corrections.

In Table 42 we list the abbreviations used in the compilation of results together with a short description.

**Table 42:** The most widely used matching and running techniques

Abbrev.	Description
RI	Regularisation-independent momentum subtraction scheme
SF	Schrödinger functional scheme
PT1$\ell$	Matching/running computed in perturbation theory at one loop
PT2$\ell$	Matching/running computed in perturbation theory at two loops
mNPR	Mostly non-perturbative renormalisation

### A.4 Chiral extrapolation

As mentioned in the introduction, Symanzik’s framework can be combined with Chiral Perturbation Theory. The well-known terms occurring in the chiral effective Lagrangian are then supplemented by contributions proportional to powers of the lattice spacing $a$. The additional terms are constrained by the symmetries of the lattice action and therefore depend on the specific choice of the discretisation. The resulting effective theory can be used to analyse the $a$-dependence of the various quantities of interest—provided the quark masses and the momenta considered are in the range where the truncated chiral perturbation series yields an adequate approximation. Understanding the dependence on the lattice spacing is of central importance for a controlled extrapolation to the continuum limit.

For staggered fermions, this program has first been carried out for a single staggered flavour (a single staggered field) [583] at $O(a^2)$. In the following, this effective theory is denoted by S$\chi$PT. It was later generalised to an arbitrary number of flavours [584, 673], and to next-to-leading order [585]. The corresponding theory is commonly called Rooted Staggered chiral perturbation theory and is denoted by RS$\chi$PT.

For Wilson fermions, the effective theory has been developed in [245, 246, 674] and is called W$\chi$PT, while the theory for Wilson twisted-mass fermions [273, 675, 676] is termed tmW$\chi$PT.

Another important approach is to consider theories in which the valence and sea quark masses are chosen to be different. These theories are called *partially quenched*. The acronym for the corresponding chiral effective theory is PQ$\chi$PT [677–680].

Finally, one can also consider theories where the fermion discretisations used for the sea and the valence quarks are different. The effective chiral theories for these “mixed-action” theories are referred to as MA$\chi$PT [247, 590, 681–685].

### A.5 Summary of simulated lattice actions

In the following tables we summarise the gauge and quark actions used in the various calculations with $N_\mathrm{f}=2, 2+1$ and $2+1+1$ quark flavours. The calculations with $N_\mathrm{f}=0$ quark flavours mentioned in Sect. 9 all used the Wilson gauge action and are not listed. Abbreviations are explained in Sects. A.1.1, A.1.2 and A.1.3, and summarised in Tables 39, 40, 41 and 42 (Tables 43, 44, 45).

**Table 43:** Summary of simulated lattice actions with $N_\mathrm{f}=2$ quark flavours

Collab.	Ref.	$N_\mathrm{f}$	Gauge action	Quark action
ALPHA 01A, 04, 05, 12	[59, 64, 488, 489]	2	Wilson	npSW
Aoki 94	[523]	2	Wilson	KS
Bernardoni 10	[261]	2	Wilson	npSW${}^\dagger$
Bernardoni 11	[259]	2	Wilson	npSW
Brandt 13	[257]	2	Wilson	npSW
Boucaud 01B	[540]	2	Wilson	Wilson
CERN-TOV 06	[272]	2	Wilson	Wilson/npSW
CERN 08	[215]	2	Wilson	npSW
CP-PACS 01	[63]	2	Iwasaki	mfSW
Davies 94	[522]	2	Wilson	KS
Dürr 11	[61]	2	Wilson	npSW
ETM 07, 07A, 08, 09, 09A-D, 10B, 10D, 10F, 11C, 12, 13	[60, 62, 145, 146, 169, 217, 238, 241, 258, 263, 392, 505, 550, 686]	2	tlSym	tmWil
ETM 10A	[314]	2	tlSym	tmWil${}^*$
Gülpers 13	[270]	2	Wilson	npSW
Hasenfratz 08	[264]	2	tadSym	n-HYP tlSW
JLQCD 08	[320]	2	Iwasaki	Overlap
JLQCD 02, 05	[70, 149]	2	Wilson	npSW
JLQCD/TWQCD 07, 08A, 10	[67, 252, 265]	2	Iwasaki	Overlap
QCDSF 07	[147]	2	Wilson	npSW
QCDSF/UKQCD 04, 06, 06A, 07	[66, 68, 170, 276]	2	Wilson	npSW
RBC 04, 06, 07	[34, 148, 313]	2	DBW2	DW
UKQCD/UKQCD 07	[144]	2	Wilson	npSW
RM123 11, 13	[45, 105]	2	tlSym	tmWil
Sesam 99	[520]	2	Wilson	Wilson
Sternbeck 10, 12	[548, 549]	2	Wilson	npSW
SPQcdR 05	[69]	2	Wilson	Wilson
TWQCD 11, 11A	[185, 260]	2	Wilson	Optimal DW
UKQCD 04	[144, 321]	2	Wilson	npSW
Wingate 95	[521]	2	Wilson	KS

${}^\dagger$ The calculation uses overlap fermions in the valence quark sector

${}^*$ The calculation uses Osterwalder–Seiler fermions [340] in the valence quark sector

**Table 44:** Summary of simulated lattice actions with $N_\mathrm{f}=2+1$ or $N_\mathrm{f}=2+1+1$ quark flavours

Collab.	Ref.	$N_\mathrm{f}$	Gauge action	Quark action
ALPHA 10A	[485]	$4$	Wilson	npSW
Aubin 08, 09	[163, 298]	$2+1$	tadSym	Asqtad${}^\dagger$
Bazavov 12	[504]	$2+1+1$	tlSym	HISQ
Blum 10	[32]	$2+1$	Iwasaki	DW
BMW 10A-C, 11, 13	[22, 23, 43, 254, 301]	$2+1$	tlSym	2-level HEX tlSW
BMW 10	[161]	$2+1$	tlSym	6-level stout tlSW
CP-PACS/JLQCD 07	[80]	$2+1$	Iwasaki	npSW
ETM 10, 10E, 11, 11D, 12C, 13, 13D	[98, 158, 217, 266, 545–547]	$2+1+1$	Iwasaki	tmWil
FNAL/MILC 12	[415]	$2+1$	tadSym	Asqtad
FNAL/MILC 12B, 13	[329, 330]	$2+1+1$	tadSym	HISQ
HPQCD 05, 05A, 08A, 13A	[81, 156, 514, 515]	$2+1$	tadSym	Asqtad
HPQCD 10	[73]	$2+1$	tadSym	Asqtad${}^*$
HPQCD/UKQCD 06	[319]	$2+1$	tadSym	Asqtad
HPQCD/UKQCD 07	[165]	$2+1$	tadSym	Asqtad${}^*$
HPQCD/MILC/UKQCD 04	[82]	$2+1$	tadSym	Asqtad
JLQCD 09, 10	[251, 512]	$2+1$	Iwasaki	Overlap
JLQCD 11, 12	[141, 142]	$2+1$	Iwasaki (fixed topology)	Overlap
JLQCD/TWQCD 08B, 09A	[162, 256]	$2+1$	Iwasaki	Overlap
JLQCD/TWQCD 10	[252]	$2+1, 3$	Iwasaki	Overlap
Laiho 11	[77]	$2+1$	tadSym	Asqtad${}^\dagger$
LHP 04	[275]	$2+1$	tadSym	Asqtad$^\dagger$
Maltman 08	[518]	$2+1$	tadSym	Asqtad
MILC 04, 07, 09, 09A, 10, 10A	[15, 36, 75, 82, 159, 687]	$2+1$	tadSym	Asqtad
NPLQCD 06	[166]	$2+1$	tadSym	Asqtad$^\dagger$
PACS-CS 08, 08A, 09, 09A, 10, 12	[19–21, 164, 487]	$2+1$	Iwasaki	npSW
Perez 10	[486]	$4$	Wilson	npSW
RBC/UKQCD 07, 08, 08A, 10, 10A-B, 11, 12, 13	[25, 78, 79, 139, 143, 253, 315, 318, 688]	$2+1$	Iwasaki, Iwasaki+DSDR	DW
Sternbeck 12	[548]	$2+1$	tlSym	npSW
SWME 10, 11, 11A, 13	[299, 300, 316, 317]	$2+1$	tadSym	Asqtad${}^+$
TWQCD 08	[255]	$2+1$	Iwasaki	DW

${}^\dagger$ The calculation uses domain-wall fermions in the valence quark sector

${}^*$ The calculation uses HISQ staggered fermions in the valence quark sector

${}^\dagger$ The calculation uses domain-wall fermions in the valence quark sector

${}^+$ The calculation uses HYP-smeared improved staggered fermions in the valence quark sector

**Table 45:** Summary of lattice simulations with $b$ and $c$ valence quarks

Collab.	Ref.	$N_\mathrm{f}$	Gauge action	Quark actions		
				Sea	Light valence	Heavy
ALPHA 11, 12A, 13	[365, 370, 404]	2	Plaquette	npSW	npSW	HQET
Atoui 13	[449]	2	tlSym	tmWil	tmWil	tmWil
ETM 09, 09D, 11B, 12A, 12B, 13B, 13C	[169, 335, 345, 392, 393, 405, 414]	2	tlSym	tmWil	tmWil	tmWil
ETM 11A	[336]	2	tlSym	tmWil	tmWil	tmWil, static
ETM 13E, 13F	[155, 399]	$2+1+1$	Iwasaki	tmWil	tmWil	tmWil
FNAL/MILC 04, 04A, 05, 08, 08A, 10, 11, 11A, 12, 13B	[332, 334, 351, 357, 412, 415, 444–447]	$2+1$	tadSym	Asqtad	Asqtad	Fermilab
FNAL/MILC 12B, 13	[329, 330]	$2+1+1$	tadSym	HISQ	HISQ	HISQ
HPQCD 06, 06A, 08B, 09	[85, 403, 413, 427]	$2+1$	tadSym	Asqtad	Asqtad	NRQCD
HPQCD 12	[402]	$2+1$	tadSym	Asqtad	HISQ	NRQCD
HPQCD/UKQCD 07, HPQCD 10A, 10B, 11, 11A, 12A, 13C	[94, 165, 331, 338, 342, 348, 366]	$2+1$	tadSym	Asqtad	HISQ	HISQ
HPQCD 13	[400]	$2+1+1$	tadSym	HISQ	HISQ	NRQCD
RBC/UKQCD 10C	[406]	$2+1$	Iwasaki	DWF	DWF	Static
RBC/UKQCD 13A	[401]	$2+1$	Iwasaki	DWF	DWF	RHQ
PACS-CS 11	[333]	$2+1$	Iwasaki	npSW	npSW	Tsukuba

## Appendix B: Notes

### B.1 Notes to Sect. 3 on quark masses

See Tables 46, 47, 48, 49, 50, 51, 52, and 53.

**Table 46:** Continuum extrapolations/estimation of lattice artefacts in determinations of $m_{ud}$, $m_{s}$ and, in some cases $m_{u}$ and $m_{d}$, with $N_\mathrm{f}=2+1$ quark flavours

Collab.	Ref.	$N_\mathrm{f}$	$a$ (fm)	Description
RBC/UKQCD 12	[25]	$2+1$	0.144, 0.113, 0.085	Scale set through $M_\Omega$. Coarsest lattice uses Iwasaki+DSDR gauge action
PACS-CS 12	[76]	$1+1+1$	0.09	Reweighting of PACS-CS 08 $N_\mathrm{f}=2+1$ QCD configurations with e.m. and $m_{u}\ne m_{d}$
Laiho 11	[77]	$2+1$	0.15, 0.09, 0.06	MILC staggered ensembles [75], scale set using $r_1$ determined by HPQCD with $\Upsilon$ splittings, pseudoscalar decay constants, through $r_1$ [186]
PACS-CS 10	[21]	$2+1$	0.09	cf. PACS-CS 08
MILC 10A	[75]	$2+1$		cf. MILC 09, 09A
BMW 10A, 10B	[22, 23]	$2+1$	0.116, 0.093, 0.077, 0.065, 0.054	Scale setting via $M_\pi , M_K, M_\Omega$
RBC/UKQCD 10A	[78]	$2+1$	0.114, 0.087	Scale set through $M_\Omega$
Blum 10	[32]	$2+1$	0.11	Relies on RBC/UKQCD 08 scale setting
PACS-CS 09	[20]	$2+1$	0.09	Scale setting via $M_\Omega$
HPQCD 09A, 10	[72, 73]	$2+1$		
MILC 09A, 09	[15, 37]	$2+1$	0.045, 0.06, 0.09	Scale set through $r_1$ and $\Upsilon$ and continuum extrapolation based on RS$\chi$PT
PACS-CS 08	[19]	$2+1$	0.09	Scale set through $M_\Omega$. Non-perturbatively $O(a)$-improved
RBC/UKQCD 08	[79]	$2+1$	0.11	Scale set through $M_\Omega$. Automatic $O(a)$-improvement due to approximate chiral symmetry. $(\Lambda _\mathrm{QCD}a)^2\approx 4\,\%$ systematic error due to lattice artefacts added
CP-PACS/JLQCD 07	[80]	$2+1$	0.07, 0.10, 0.12	Scale set through $M_K$ or $M_\phi$. Non-perturbatively $O(a)$-improved
HPQCD 05	[81]	$2+1$	0.09, 0.12	Scale set through the $\Upsilon -\Upsilon '$ mass difference
HPQCD/MILC/UKQCD 04, MILC 04	[36, 82]	$2+1$	0.09, 0.12	Scale set through $r_1$ and $\Upsilon$ and continuum extrapolation based on RS$\chi$PT

**Table 47:** Continuum extrapolations/estimation of lattice artefacts in determinations of $m_{ud}$, $m_{s}$ and, in some cases $m_{u}$ and $m_{d}$, with $N_\mathrm{f}=2$ quark flavours

Collab.	Ref.	$N_\mathrm{f}$	$a$ (fm)	Description
RM123 13	[45]	2	0.098, 0.085, 0.067, 0.054	cf. ETM 10B
ALPHA 12	[59]	2	0.076, 0.066, 0.049	Scale set through $F_K$
RM123 11	[105]	2	0.098, 0.085, 0.067, 0.054	cf. ETM 10B
Dürr 11	[61]	2	0.076, 0.072, 0.060	Scale for light quark masses set through $m_{c}$
ETM 10B	[60]	2	0.098, 0.085, 0.067, 0.054	Scale set through $F_\pi$
JLQCD/TWQCD 08A	[67]	2	0.12	Scale set through $r_0$
RBC 07	[34]	2	0.12	Scale set through $M_\rho$
ETM 07	[62]	2	0.09	Scale set through $F_\pi$
QCDSF/UKQCD 06	[68]	2	0.065–0.09	Scale set through $r_0$
SPQcdR 05	[69]	2	0.06, 0.08	Scale set through $M_{K^*}$
ALPHA 05	[64]	2	0.07–0.12	Scale set through $r_0$
QCDSF/UKQCD 04	[66]	2	0.07–0.12	Scale set through $r_0$
JLQCD 02	[70]	2	0.09	Scale set through $M_\rho$
CP-PACS 01	[63]	2	0.11, 0.16, 0.22	Scale set through $M_\rho$

**Table 48:** Chiral extrapolation/minimum pion mass in determinations of $m_{ud}$, $m_{s}$ and, in some cases $m_{u}$ and $m_{d}$, with $N_\mathrm{f}=2+1$ quark flavours

Collab.	Ref.	$N_\mathrm{f}$	$M_{\pi ,\text {min}}$ (MeV)	Description
RBC/UKQCD 12	[25]	$2+1$	170	Combined fit to Iwasaki and Iwasaki+DSDR gauge action ensembles
PACS-CS 12	[76]	$1+1+1$		cf. PACS-CS 08
Laiho 11	[77]	$2+1$	210 (val.) 280 (sea-RMS)	NLO SU(3), mixed-action $\chi$PT [590], with N$^2$LO-N$^4$LO analytic terms
PACS-CS 10	[21]	$2+1$		cf. PACS-CS 08
MILC 10A	[75]	$2+1$		NLO SU(2) S$\chi$PT. Cf. also MILC 09A,09
BMW 10A, 10B	[22, 23]	$2+1$	135	Interpolation to the physical point
RBC/UKQCD 10A	[78]	$2+1$	290	
Blum 10	[32, 79]	$2+1$	242 (valence), 330 (sea)	Extrapolation done on the basis of PQ$\chi$PT formulae with virtual photons
PACS-CS 09	[20]	$2+1$	135	Physical point reached by reweighting technique, no chiral extrapolation needed
HPQCD 09A, 10	[72, 73]	$2+1$		
MILC 09A, 09	[15, 37]	$2+1$	177, 240	NLO SU(3) RS$\chi$PT, continuum $\chi$PT at NNLO and NNNLO and NNNNLO analytic terms. The lightest Nambu–Goldstone mass is 177 MeV (09A) and 224 MeV (09) (at $a=$ 0.09 fm) and the lightest RMS mass is 258MeV (at $a=0.06$ fm)
PACS-CS 08	[19]	$2+1$	156	NLO SU(2) $\chi$PT and SU(3) (Wilson)$\chi$PT
RBC/UKQCD 08	[79]	$2+1$	242 (valence), 330 (sea)	SU(3) PQ$\chi$PT and heavy kaon NLO SU(2) PQ$\chi$PT fits
CP-PACS/JLQCD 07	[80]	$2+1$	620	NLO Wilson $\chi$PT fits to meson masses
HPQCD 05	[81]	$2+1$	240	PQ RS$\chi$PT fits
HPQCD/MILC/UKQCD 04, MILC 04	[36, 82]	$2+1$	240	PQ RS$\chi$PT fits

**Table 49:** Chiral extrapolation/minimum pion mass in determinations of $m_{ud}$, $m_{s}$ and, in some cases $m_{u}$ and $m_{d}$, with $N_\mathrm{f}=2$ quark flavours

Collab.	Ref.	$N_\mathrm{f}$	$M_{\pi ,\text {min}}$ (MeV)	Description
RM123 13	[45]	2	270	Fits based on NLO $\chi$PT and Symanzik expansion up to $O(a^2)$. $O(\alpha )$ e.m. effects included
ALPHA 12	[59]	2	270	NLO SU(2) and SU(3) $\chi$PT and $O(a^2)$ on LO LEC
RM123 11	[105]	2	270	Fits based on NLO $\chi$PT and Symanzik expansion up to $O(a^2)$
Dürr 11	[61]	2	285	$m_{c}/m_{s}$ determined by quadratic or cubic extrapolation in $M_\pi$
ETM 10B	[60]	2	270	Fits based on NLO $\chi$PT and Symanzik expansion up to $O(a^2)$
JLQCD/TWQCD 08A	[67]	2	290	NLO $\chi$PT fits
RBC 07	[34]	2	440	NLO fit including $O(\alpha )$ effects
ETM 07	[62]	2	300	Polynomial and PQ$\chi$PT fits
QCDSF/UKQCD 06	[68]	2	520 (valence), 620 (sea)	NLO (PQ)$\chi$PT fits
SPQcdR 05	[69]	2	600	Polynomial fit
ALPHA 05	[64]	2	560	LO $\chi$PT fit
QCDSF/UKQCD 04	[66]	2	520 (valence), 620 (sea)	NLO (PQ)$\chi$PT fits
JLQCD 02	[70]	2	560	Polynomial and $\chi$PT fits
CP-PACS 01	[63]	2	430	Polynomial fits

**Table 50:** Finite-volume effects in determinations of $m_{ud}$, $m_{s}$ and, in some cases $m_{u}$ and $m_{d}$, with $N_\mathrm{f}=2+1$ quark flavours

Collab.	Ref.	$N_\mathrm{f}$	$L$ (fm)	$M_{\pi ,\text {min}} L$	Description
RBC/UKQCD 12	[25]	$2+1$	2.7, 4.6	$\gtrsim 4.0$	Uses FV chiral perturbation theory to estimate the error
PACS-CS 12	[76]	$1+1+1$			cf. PACS-CS 08
Laiho 11	[77]	$2+1$	2.5, 2.9, 3.0, 3.6, 3.8, 4.8	4.1 (val.) 4.1 (sea)	Data corrected using NLO SU(3) $\chi$PT finite-V formulae
PACS-CS 10	[21]	$2+1$			cf. PACS-CS 08
MILC 10A	[75]	$2+1$			cf. MILC 09A,09
BMW 10A, 10B	[22, 23]	$2+1$	$\gtrsim 5.0$	$\gtrsim 4.0$	FS corrections below 5 per mil on the largest lattices
RBC/UKQCD 10A	[78]	$2+1$	2.7	$\gtrsim 4.0$	
Blum 10	[32]	$2+1$	1.8, 2.7	–	Simulations done with quenched photons; large finite-volume effects analytically corrected for, but they are not related to $M_\pi L$
PACS-CS 09	[20]	$2+1$	2.9	2.0	Only one volume
HPQCD 09A, 10	[72, 73]	$2+1$			
MILC 09A, 09	[15, 37]	$2+1$	2.5, 2.9, 3.4, 3.6, 3.8, 5.8	4.1, 3.8	
PACS-CS 08	[19]	$2+1$	2.9	2.3	Correction for FSE from $\chi$PT using [689]
RBC/UKQCD 08	[79]	$2+1$	1.8, 2.7	4.6	Various volumes for comparison and correction for FSE from $\chi$PT [189, 190, 689]
CP-PACS/JLQCD 07	[80]	$2+1$	2.0	6.0	Estimate based on the comparison to a $L=1.6$ fm volume assuming power-like dependence on $L$
HPQCD 05	[81]	$2+1$	2.4, 2.9	3.5	
HPQCD/MILC/UKQCD 04, MILC 04	[36, 82]	$2+1$	2.4, 2.9	3.5	NLO S$\chi$PT

**Table 51:** Finite-volume effects in determinations of $m_{ud}$, $m_{s}$ and, in some cases $m_{u}$ and $m_{d}$, with $N_\mathrm{f}=2$ quark flavours

Collab.	Ref.	$N_\mathrm{f}$	$L$ (fm)	$M_{\pi ,\text {min}} L$	Description
RM123 13	[45]	2	$\gtrsim 2.0$	3.5	One volume $L=1.7$ fm at $m_\pi =495, a=0.054$ fm
ALPHA 12	[59]	2	2.1–3.2	4.2	Roughly 2 distinct volumes; no analysis of FV effects
RM123 11	[105]	2	$\gtrsim 2.0$	3.5	One volume $L=1.7$ fm at $m_\pi =495, a=0.054$ fm
Dürr 11	[61]	2	1.22–2.30	2.8	Number of volumes in determination of $m_{c}/m_{s}$, but all but one have $L<2\,\,{\mathrm {fm}}$
ETM 10B	[60]	2	$\gtrsim 2.0$	3.5	One volume $L=1.7$ fm at $m_\pi =495, a=0.054$ fm
JLQCD/TWQCD 08A	[67]	2	1.9	2.8	Corrections for FSE based on NLO $\chi$PT
RBC 07	[34]	2	1.9	4.3	Estimate of FSE based on a model
ETM 07	[62]	2	2.1	3.2	NLO PQ$\chi$PT
QCDSF/UKQCD 06	[68]	2	1.4–1.9	4.7	
SPQcdR 05	[69]	2	1.0–1.5	4.3	Comparison between 1.0 and 1.5 fm
ALPHA 05	[64]	2	2.6	7.4	
QCDSF/UKQCD 04	[66]	2	1.7–2.0	4.7	
JLQCD 02	[70]	2	1.8	5.1	Numerical study with three volumes
CP-PACS 01	[63]	2	2.0–2.6	5.7

**Table 52:** Renormalisation in determinations of $m_{ud}$, $m_{s}$ and, in some cases $m_{u}$ and $m_{d}$, with $N_\mathrm{f}=2+1$ quark flavours

Collab.	Ref.	$N_\mathrm{f}$	Description
RBC/UKQCD 12	[25]	$2+1$	Non-perturbative renormalisation (RI/SMOM)
PACS-CS 12	[76]	$1+1+1$	cf. PACS-CS 10
Laiho 11	[77]	$2+1$	$Z_A$ from AWI and $Z_A/Z_S-1$ from one-loop, tadpole-improved, perturbation theory
PACS-CS 10	[21]	$2+1$	Non-perturbative renormalisation and running; Schrödinger functional method
MILC 10A	[75]	$2+1$	cf. MILC 09A,09
BMW 10A, 10B	[22, 23]	$2+1$	Non-perturbative renormalisation (tree-level improved RI-MOM), non-perturbative running
RBC/UKQCD 10A	[78]	$2+1$	Non-perturbative renormalisation (RI/SMOM)
Blum 10	[32]	$2+1$	Relies on non-perturbative renormalisation factors calculated by RBC/UKQCD 08; no QED renormalisation
PACS-CS 09	[20]	$2+1$	Non-perturbative renormalisation; Schrödinger functional method
HPQCD 09A, 10	[72, 73]	$2+1$	Lattice calculation of $m_{s}/m_{c}$: $m_{s}$ derived from a perturbative determination of $m_{c}$
MILC 09A, 09	[15, 37]	$2+1$	Two-loop perturbative renormalisation
PACS-CS 08	[19]	$2+1$	One-loop perturbative renormalisation
RBC/UKQCD 08	[79]	$2+1$	Non-perturbative renormalisation, three-loop perturbative matching
CP-PACS/JLQCD 07	[80]	$2+1$	One-loop perturbative renormalisation, tadpole improved
HPQCD 05	[81]	$2+1$	Two-loop perturbative renormalisation
HPQCD/MILC/UKQCD 04, MILC 04	[36, 82]	$2+1$	One-loop perturbative renormalisation

**Table 53:** Renormalisation in determinations of $m_{ud}$, $m_{s}$ and, in some cases $m_{u}$ and $m_{d}$, with $N_\mathrm{f}=2$ quark flavours

Collab.	Ref.	$N_\mathrm{f}$	Description
RM123 13	[45]	2	Non-perturbative renormalisation
ALPHA 12	[59]	2	Non-perturbative renormalisation
RM123 11	[105]	2	Non-perturbative renormalisation
Dürr 11	[61]	2	Lattice calculation of $m_{s}/m_{c}$: $m_{s}$ derived from a perturbative determination of $m_{c}$
ETM 10B	[60]	2	Non-perturbative renormalisation
JLQCD/TWQCD 08A	[67]	2	Non-perturbative renormalisation
RBC 07	[34]	2	Non-perturbative renormalisation
ETM 07	[62]	2	Non-perturbative renormalisation
QCDSF/UKQCD 06	[68]	2	Non-perturbative renormalisation
SPQcdR 05	[69]	2	Non-perturbative renormalisation
ALPHA 05	[64]	2	Non-perturbative renormalisation
QCDSF/UKQCD 04	[66]	2	Non-perturbative renormalisation
JLQCD 02	[70]	2	One-loop perturbative renormalisation
CP-PACS 01	[63]	2	One-loop perturbative renormalisation

### B.2 Notes to Sect. 4 on $|V_{ud}|$ and $|V_{us}|$

See Tables 54, 55, 56, 57, 58, 59, 60, 61, and 62.

**Table 54:** Continuum extrapolations/estimation of lattice artefacts in determinations of $f_+(0)$

Collab.	Ref.	$N_\mathrm{f}$	$a$ (fm)	Description
FNAL/MILC 13C	[138]	$2+1+1$	0.09, 0.12, 0.15	Relative scale through $r_1$, physical scale from $f_\pi$ calculated by MILC 09A at $N_\mathrm{f} = 2 + 1$
FNAL/MILC 12	[140]	$2+1$	0.09, 0.12	Relative scale $r_1$, physical scale determined from a mixture of $f_\pi$, $f_K$, radial excitation of $\Upsilon$ and $m_{D_{s}}-\frac{1}{2} m_{ {\eta }_{c}}$
RBC/UKQCD 13	[139]	$2+1$	0.09, 0.11, 0.14	Scale set through $\Omega$ mass
JLQCD 12	[141]	$2+1$	0.112	Scale set through $\Omega$ mass
JLQCD 11	[142]	$2+1$	0.112	Scale set through $\Omega$ mass
RBC/UKQCD 07,10	[143, 144]	$2+1$	0.114 (2)	Scale fixed through $\Omega$ baryon mass. Add $(\Lambda _\mathrm{QCD}a)^2\approx 4$ % systematic error for lattice artefacts. Fifth dimension with extension $L_{s}=16$, therefore small residual chiral symmetry breaking and approximate $O(a)$-improvement
ETM 10D	[145]	2	0.05, 0.07, 0.09, 0.10	Scale set through $F_\pi$. Automatic $O(a)$ impr., flavour symmetry breaking: $(M_{PS}^{0})^2 - (M_{PS}^\pm )^2\sim O(a^2)$
ETM 09A	[146]	2	0.07, 0.09, 0.10	Scale set through $F_\pi$. Automatic $O(a)$ impr., flavour symmetry breaking: $(M_{PS}^{0})^2 - (M_{PS}^\pm )^2\sim O(a^2)$. Three lattice spacings only for pion mass 470 MeV
QCDSF 07	[147]	2	0.075	Scale set with $r_0$. Non-perturbatively $O(a)$-improved Wilson fermions, not clear whether currents improved
RBC 06	[148]	2	0.12	Scale set through $M_\rho$. Automatic $O(a)$-improvement due to approximate chiral symmetry of the action
JLQCD 05	[149]	2	0.0887	Scale set through $M_\rho$. Non-perturbatively $O(a)$-improved Wilson fermions

**Table 55:** Chiral extrapolation/minimum pion mass in determinations of $f_+(0)$. The subscripts RMS and $\pi ,5$ in the case of staggered fermions indicate the root-mean-square mass and the Nambu–Goldstone boson mass, respectively. In the case of twisted-mass fermions $\pi ^0$ and $\pi ^\pm$ indicate the neutral- and charged-pion mass where applicable

Collab.	Ref.	$N_\mathrm{f}$	$M_{\pi ,\text {min}}$ (MeV)	Description
FNAL/MILC 13C	[138]	$2+1+1$	$173_\mathrm{RMS}(128_{\pi ,5})$	NLO SU(3) PQ staggered $\chi$PT with continuum $\chi$PT at NNLO. Lightest Nambu–Goldstone mass is 128 MeV and lightest RMS mass is 173 MeV for the same gauge ensemble with $a \simeq 0.09$ fm
FNAL/MILC 12	[140]	$2+1$	$378_\mathrm{RMS}(263_{\pi ,5})$	NLO SU(3) PQ staggered $\chi$PT with either phenomenological NNLO ansatz or NNLO $\chi$PT. Lightest Nambu–Goldstone mass is 263 MeV with $a=0.12$ fm and lightest RMS mass is 378 MeV with $a=0.09$ fm
RBC/UKQCD 13	[139]	$2+1$	170	NLO SU(3) $\chi$PT with phenomenological ansatz for higher orders
JLQCD 12	[141]	$2+1$	290	NLO SU(3) $\chi$PT with phenomenological ansatz for higher orders
JLQCD 11	[142]	$2+1$	290	NLO SU(3) $\chi$PT with phenomenological ansatz for higher orders
RBC/UKQCD 07,10	[143, 144]	$2+1$	330	NLO SU(3) $\chi$PT with phenomenological ansatz for higher orders
ETM 10D	[145]	2	$210_{\pi ^0}(260_{\pi ^\pm })$	NLO heavy kaon SU(2) $\chi$PT and NLO SU(3) $\chi$PT and phenomenological ansatz for higher orders. Average of $f_+(0)$ fit and joint $f_+(0)$–$f_K/f_\pi$ fit
ETM 09A	[146]	2	$210_{\pi ^0}(260_{\pi ^\pm })$	NLO heavy kaon SU(2) $\chi$PT and NLO SU(3) $\chi$PT and phenomenological ansatz for higher orders
QCDSF 07	[147]	2	591	Only one value for the pion mass
RBC 06	[148]	2	490	NLO SU(3) $\chi$PT and phenomenological ansatz for higher orders
JLQCD 05	[149]	2	550	NLO SU(3) $\chi$PT and phenomenological ansatz for higher orders

**Table 56:** Finite-volume effects in determinations of $f_+(0)$. The subscripts RMS and $\pi ,5$ in the case of staggered fermions indicate the root-mean-square mass and the Nambu–Goldstone boson mass, respectively. In the case of twisted-mass fermions $\pi ^0$ and $\pi ^\pm$ indicate the neutral and charged-pion mass where applicable

Collab.	Ref.	$N_\mathrm{f}$	$L$ (fm)	$M_{\pi ,\text {min}}L$	Description
FNAL/MILC 13C	[138]	$2+1+1$	2.9–5.8	$4.9_\mathrm{RMS}(3.6_{\pi ,5})$	The values correspond to $M_{\pi ,\mathrm RMS}=173$ MeV and $M_{\pi ,5}=128$ MeV, respectively
FNAL/MILC 12	[140]	$2+1$	2.4–3.4	$6.2_\mathrm{RMS}(3.8_{\pi ,5})$	The values correspond to $M_{\pi ,\mathrm RMS}=378$ MeV and $M_{\pi ,5}=263$ MeV, respectively
RBC/UKQCD 13	[139]	$2+1$	2.7, 4.6	3.9	
JLQCD 12	[141]	$2+1$	1.8, 2.7	4.1	
JLQCD 11	[142]	$2+1$	1.8, 2.7	4.1	
RBC/UKQCD 07,10	[143, 144]	$2+1$	1.8, 2.7	4.7	Two volumes for all but the lightest pion mass
ETM 10D	[145]	2	2.1–2.8	$3.0_{\pi ^0}(3.7_{\pi ^\pm })$	
ETM 09A	[146]	2	2.1, 2.8	$3.0_{\pi ^0}(3.7_{\pi ^\pm })$	Two volumes at $M_\pi =300$ MeV and $\chi$PT-motivated estimate of the error due to FSE
QCDSF 07	[147]	2	1.9	5.4	
RBC 06	[148]	2	1.9	4.7	
JLQCD 05	[149]	2	1.8	4.9

**Table 57:** Continuum extrapolations/estimation of lattice artefacts in determinations of $f_K/f\pi$ for $N_\mathrm{f}=2+1$ and $N_\mathrm{f}=2+1+1$ simulations

Collab.	Ref.	$N_\mathrm{f}$	$a$ (fm)	Description
HPQCD 13A	[156]	$2+1+1$	0.09, 0.12, 0.15	Relative scale through Wilson flow and absolute scale through $f_\pi$
MILC 13A	[157]	$2+1+1$	0.06, 0.09, 0.12, 0.15	Absolute scale though $f_\pi$
ETM 13F	[155]	$2+1+1$	0.062, 0.082, 0.089	Scale set through $f_\pi$. Automatic $Ow(a)$ improvement, flavour symmetry breaking: $(M_{PS}^{0})^2-(M_{PS}^\pm )^2\sim O(a^2)$. Discretisation and volume effects due to the $\pi ^0 - \pi ^\pm$ mass splitting are taken into account through $\chi$PT for twisted-mass fermions
ETM 10E	[158]	$2+1+1$	0.061, 0.078	Scale set through $f_\pi /m_\pi$. Two lattice spacings but $a$-dependence ignored in all fits. Finer lattice spacing from [266]
MILC 11	[24]	$2+1+1$	0.12, 0.09	Relative scale through $f_\mathrm{PS}/m_\mathrm{PS}=\hbox {fixed}$, absolute scale though $f_\pi$
RBC/UKQCD 12	[25]	$$2+1 $$	0.09, 0.11, 0.14	Scale set through $$m_\Omega $$
LAIHO 11	[77]	$2+1$	0.125, 0.09, 0.06	Scale set through $r_1$ and $\Upsilon$ and continuum extrapolation based on MA$\chi$PT
JLQCD/TWQCD 10	[160]	$2+1$	0.112	Scale set through $M_\Omega$
RBC/UKQCD 10A	[78]	$2+1$	0.114, 0.087	Scale set through $M_\Omega$
MILC 10	[159]	$2+1$	0.09, 0.06, 0.045	three lattice spacings, continuum extrapolation by means of RS$\chi$PT
BMW 10	[161]	$2+1$	0.07, 0.08, 0.12	Scale set through $M_{\Omega ,\Xi }$. Perturbative $O(a)$-improvement
JLQCD/TWQCD 09A	[67]	$2+1$	0.1184 (3) (21)	Scale set through $F_\pi$. Automatic $O(a)$-improvement due to chiral symmetry of action
PACS-CS 09	[20]	$2+1$	0.0900 (4)	Scale set through $M_\Omega$
MILC 09A	[37]	$2+1$	0.045, 0.06, 0.09	Scale set through $r_1$ and $\Upsilon$ and continuum extrapolation based on RS$\chi$PT
MILC 09	[15]	$2+1$	0.045, 0.06, 0.09, 0.12	Scale set through $r_1$ and $\Upsilon$ and continuum extrapolation based on RS$\chi$PT
Aubin 08	[163]	$2+1$	0.09, 0.12	Scale set through $r_1$ and $\Upsilon$ and continuum extrapolation based on MA$\chi$PT
PACS-CS 08, 08A	[19, 164]	$2+1$	0.0907 (13)	Scale set through $M_\Omega$. Non-perturbatively $O(a)$-improved
HPQCD/UKQCD 07	[165]	$2+1$	0.09, 0.12, 0.15	Scale set through $r_1$ and $\Upsilon$ and continuum extrapolation on continuum-$\chi$PT-motivated ansatz. Taste breaking of sea quarks ignored
RBC/UKQCD 08	[79]	$2+1$	0.114 (2)	Scale set through $M_\Omega$. Automatic $O(a)$-improvement due to approximate chiral symmetry. $(\Lambda _\mathrm{QCD}a)^2\approx 4\,\%$ systematic error due to lattice artefacts added
NPLQCD 06	[166]	$2+1$	0.125	Scale set through $r_0$ and $F_\pi$. Taste breaking of sea quarks ignored

**Table 58:** Continuum extrapolations/estimation of lattice artefacts in determinations of $f_K/f\pi$ for $N_\mathrm{f}=2$ simulations

Collab.	Ref.	$N_\mathrm{f}$	$a$ (fm)	Description
ALPHA 13	[167]	2	0.05, 0.065, 0.075	Scale set through $F_\pi$. $O(a)$-improved Wilson action
BGR 11	[168]	2	0.135	Scale set through $r_0 = 0.48$ fm. Chirally improved Dirac operator
ETM 10D	[145]	2	0.05, 0.07, 0.09, 0.10	Scale set through $F_\pi$. Automatic $O(a)$ impr., flavour symmetry breaking: $(M_{PS}^{0})^2-(M_{PS}^\pm )^2\sim O(a^2)$
ETM 09	[169]	2	0.07, 0.09, 0.10	Scale set through $F_\pi$. Automatic $O(a)$ impr., flavour symmetry breaking: $(M_{PS}^{0})^2-(M_{PS}^\pm )^2\sim O(a^2)$
QCDSF/UKQCD 07	[170]	2	0.06, 0.07	Scale set through $F_\pi$. Non-perturbative $O(a)$-improvement

**Table 59:** Chiral extrapolation/minimum pion mass in determinations of $f_K / f\pi$ for $N_\mathrm{f}=2+1+1$ simulations. The subscripts RMS and $\pi ,5$ in the case of staggered fermions indicate the root-mean-square mass and the Nambu–Goldstone boson mass. In the case of twisted-mass fermions $\pi ^0$ and $\pi ^\pm$ indicate the neutral- and charged-pion mass and, where applicable, “val” and “sea” indicate valence- and sea-pion masses

Collab.	Ref.	$N_\mathrm{f}$	$M_{\pi ,\text {min}}$ (MeV)	Description
HPQCD 13A	[156]	$2+1+1$	$173_\mathrm{RMS}(128_{\pi ,5})$	NLO $\chi$PT supplemented by model for NNLO. Both the lightest RMS and the lightest Nambu–Goldstone mass are from the $a=0.09$ fm ensemble
MILC 13A	[157]	$2+1+1$	$143_\mathrm{RMS}(128_{\pi ,5})$	Linear interpolation to physical point. The lightest RMS mass is from the $a=0.06$ fm ensemble and the lightest Nambu–Goldstone mass is from the $a=0.09$ fm ensemble
ETM 13F	[155]	$2+1+1$	$155_{\pi ^0}(220_{\pi ^\pm })$	Chiral extrapolation performed through SU(2) $\chi$PT or polynomial fit
ETM 10E	[158]	$2+1+1$	$215_{\pi ^0}(265_{\pi ^\pm })$	
MILC 11	[24]	$2+1+1$	$173_\mathrm{RMS}(128_{\pi ,5})$	Quoted result from polynomial interpolation to the physical point. The lightest RMS mass is from the $a=0.06$ fm ensemble and the lightest Nambu–Goldstone mass is from the $a=0.09$ fm ensemble

**Table 60:** Chiral extrapolation/minimum pion mass in determinations of $f_K/f\pi$ for $N_\mathrm{f}=2+1$ simulations. The subscripts RMS and $\pi ,5$ in the case of staggered fermions indicate the root-mean-square mass and the Nambu–Goldstone boson mass. In the case of twisted-mass fermions $\pi ^0$ and $\pi ^\pm$ indicate the neutral and charged pion mass and where applicable, “val” and “sea” indicate valence- and sea-pion masses

Collab.	Ref.	$N_\mathrm{f}$	$M_{\pi ,\text {min}}$ (MeV)	Description
RBC/UKQCD 12	[25]	$2+1$	171$_\mathrm{sea}$, 143$_\mathrm{val}$	NLO PQ SU(2) $\chi$PT as well as analytic ansätze
LAIHO 11	[77]	$2+1$	250$_\mathrm{RMS}(220_{\pi ,5})$	NLO MA$\chi$PT
JLQCD/TWQCD 10	[160]	$2+1$	290	NNLO $\chi$PT
RBC/UKQCD 10A	[78]	$2+1$	290	Results are based on heavy kaon NLO SU(2) PQ$\chi$PT
MILC 10	[159]	$2+1$	$258_\mathrm{RMS}(177_{\pi ,5})$	Lightest Nambu–Goldstone mass is 177 MeV (at 0.09 fm) and lightest RMS mass is 258 MeV (at 0.06 fm). NLO rS$\chi$PT and NNLO $\chi$PT
BMW 10	[161]	$2+1$	190	Comparison of various fit-ansätze: SU(3) $\chi$PT, heavy kaon SU(2) $\chi$PT, polynomial
JLQCD/TWQCD 09A	[67]	$2+1$	290	NNLO SU(3) $\chi$PT
PACS-CS 09	[20]	$2+1$	156	NNLO $\chi$PT
MILC 09A	[37]	$2+1$	$258_\mathrm{RMS}(177_{\pi ,5})$	NLO SU(3) RS$\chi$PT, continuum $\chi$PT at NNLO and up to NNNNLO analytic terms. Heavy kaon SU(2) RS$\chi$PT with NNLO continuum chiral logs on a subset of the lattices. The lightest Nambu–Goldstone mass is 177 MeV (at $a=0.09$ fm) and the lightest RMS mass is 258 MeV (at $a=0.06$ fm)
MILC 09	[15]	$2+1$	$258_\mathrm{RMS}(224_{\pi ,5})$	NLO SU(3) RS$\chi$PT with continuum $\chi$PT NNLO and NNNLO analytic terms added. According to [37] the lightest sea Nambu–Goldstone mass is 224 MeV and the lightest RMS mass is 258 MeV (at $a=0.06$ fm)
Aubin 08	[163]	$2+1$	$329_\mathrm{RMS}(246_{\pi ,5})$	NLO MA$\chi$PT. According to [37] the lightest sea Nambu–Goldstone mass is 246 MeV (at $a=0.09$ fm) and the lightest RMS mass is 329 MeV (at $a=0.09$ fm)
PACS-CS 08, 08A	[19, 164]	$2+1$	156	NLO SU(2) $\chi$PT and SU(3) (Wilson)$\chi$PT
HPCD/UKQCD 07	[165]	$2+1$	$375_\mathrm{RMS}(263_{\pi ,5})$	NLO SU(3) chiral perturbation theory with NNLO and NNNLO analytic terms. The lightest RMS mass is from the $a=0.09$ fm ensemble and the lightest Nambu–Goldstone mass is from the $a=0.12$ fm ensemble
RBC/UKQCD 08	[79]	$2+1$	$330_\mathrm{sea}$, $242_\mathrm{val}$	While SU(3) PQ$\chi$PT fits were studied, final results are based on heavy kaon NLO SU(2) PQ$\chi$PT
NPLQCD 06	[166]	$2+1$	300	NLO SU(3) $\chi$PT and some NNLO terms. The sea RMS mass for the employed lattices is heavier

**Table 61:** Chiral extrapolation/minimum pion mass in determinations of $f_K/f\pi$ for $N_\mathrm{f}=2$ simulations. The subscripts RMS and $\pi ,5$ in the case of staggered fermions indicate the root-mean-square mass and the Nambu–Goldstone boson mass. In the case of twisted-mass fermions $\pi ^0$ and $\pi ^\pm$ indicate the neutral- and charged-pion mass and where applicable, “val” and “sea” indicate valence- and sea-pion masses

Collab.	Ref.	$N_\mathrm{f}$	$M_{\pi ,\text {min}}$ (MeV)	Description
ALPHA 13	[167]	2	190	NLO SU(3) $\chi$PT and phenomenological ansatz for higher orders
BGR 11	[168]	2	250	NLO SU(2) $\chi$PT. Strange quark mass fixed by reproducing the $\Omega$ mass
ETM 10D	[145]	2	$210_{\pi ^0}(260_{\pi ^\pm })$	NLO SU(3) $\chi$PT and phenomenological ansatz for higher orders. Joint $f_+(0)$–$f_K/f_\pi$ fit
ETM 09	[169]	2	$210_{\pi ^0}(260_{pi^\pm })$	NLO heavy-meson SU(2) $\chi$PT and NLO SU(3) $\chi$PT
QCDSF/UKQCD 07	[170]	2	300	Linear extrapolation of lattice data

**Table 62:** Finite-volume effects in determinations of $f_K/f\pi$. The subscripts RMS and $\pi ,5$ in the case of staggered fermions indicate the root-mean-square mass and the Nambu–Goldstone boson mass. In the case of twisted-mass fermions $\pi ^0$ and $\pi ^\pm$ indicate the neutral- and charged-pion mass and where applicable, “val” and “sea” indicate valence- and sea-pion masses

Collab.	Ref.	$N_\mathrm{f}$	$L$ (fm)	$M_{\pi ,\mathrm{min}}L$	Description
HPQCD 13A	[156]	$2+1+1$	2.5–5.8	$4.9_\mathrm{RMS}(3.7_{\pi ,5})$	
MILC 13A	[157]	$2+1+1$	2.8–5.8	$3.9_\mathrm{RMS}(3.7_{\pi ,5})$	
ETM 13F	[155]	$2+1+1$	2.0–3.0	$1.7_{\pi ^0}(3.3_{\pi ^\pm })$	FSE for the pion is corrected through resummed NNLO $\chi$PT for twisted-mass fermions, which takes into account the effects due to the $\pi ^0$–$\pi ^\pm$ mass splitting
ETM 10E	[158]	$2+1+1$	1.9–2.9	$3.1_{\pi ^0}(3.9_{\pi ^\pm })$	Simulation parameters from [266, 690]
MILC 11	[24]	$2+1+1$	5.6, 5.7	$4.9_\mathrm{RMS}(3.7_{\pi ,5})$	
RBC/UKQCD 12	[25]	$2+1$	2.7, 4.6	$3.3$	For partially quenched $M_\pi =143$ MeV, $M_\pi L=3.3$ and for unitary $M_\pi =171$ MeV, $M_\pi L=4.0$
LAIHO 11	[77]	$2+1$	2.5–4.0	$4.9_\mathrm{RMS}(4.3_{\pi ,5})$	
JLQCD/TWQCD 10	[160]	$2+1$	1.8, 2.7	4.0	
RBC/UKQCD 10A	[78]	$2+1$	2.7	$4.0$	$M_\pi L=4.0$ for lightest sea quark mass and $M_\pi L=3.1$ for lightest partially quenched quark mass
MILC 10	[159]	$2+1$	2.5–3.8	$7.0_\mathrm{RMS}(4.0_{\pi ,5})$	$L\!\ge \!2.9\,\,{\mathrm {fm}}$ for the lighter masses
BMW 10	[161]	$2+1$	2.0–5.3	4.0	Various volumes for comparison and correction for FSE from $\chi$PT using [689]
JLQCD/TWQCD 09A	[67]	$2+1$	1.9	2.8	Estimate of FSE using $\chi$PT [689, 691]
PACS-CS 09	[20]	$2+1$	2.9	2.28	After reweighting to the physical point $M_{\pi ,\mathrm min}L=1.97$
MILC 09A	[37]	$2+1$	2.5–5.8	$7.0_\mathrm{RMS}(4.1_{\pi ,5})$	
MILC 09	[15]	$2+1$	2.4–5.8	$7.0_\mathrm{RMS}(4.8_{\pi ,5})$	Various volumes for comparison and correction for FSEs from (RS)$\chi$PT [689]
Aubin 08	[163]	$2+1$	2.4–3.6	4.0	Correction for FSE from MA$\chi$PT
PACS-CS 08, 08A	[19, 164]	$2+1$	2.9	2.3	Correction for FSE from $\chi$PT using [689]
HPCD/UKQCD 07	[165]	$2+1$	2.4–2.9	$4.1_\mathrm{RMS}(3.8_{\pi ,5})$	Correction for FSE from $\chi$PT using [689]
RBC/UKQCD 08	[79]	$2+1$	1.8, 2.7	$4.6_\mathrm{sea}$, $3.4_\mathrm{val}$	Various volumes for comparison and correction for FSE from $\chi$PT [189, 190, 689]
NPLQCD 06	[166]	$2+1$	2.5	3.8	Correction for FSE from S$\chi$PT [584, 673]
ALPHA 13	[167]	2	2.1, 2.4, 3.1	$4.0$	
BGR 11	[168]	2	2.1, 2.2	$2.7$	
ETM 10D	[145]	2	2.1–2.8	$3.0_{\pi ^0}(3.7_{\pi ^\pm })$	
ETM 09	[169]	2	2.0–2.7	$3.0_{\pi ^0}(3.7_{\pi ^\pm })$	Correction for FSE from $\chi$PT [189, 190, 689]
QCDSF/UKQCD 07	[170]	2	1.4,..., 2.6	4.2	Correction for FSE from $\chi$PT

### B.3 Notes to Sect. 5 on Low-Energy Constants

See Tables 63, 64, 65, 66, 67, 68, and 69.

**Table 63:** Continuum extrapolations/estimation of lattice artefacts in $N_\mathrm{f}=2+1+1$ and $2+1$ determinations of the low-energy constants

Collab.	Ref.	$N_\mathrm{f}$	$a$ (fm)	Description
HPQCD 13A	[156]	$2+1+1$	0.09–0.15	Configurations are shared with MILC
ETM 13	[217]	$2+1+1$	0.0607–0.0863	Configurations are shared with ETM 11
ETM 11	[266]	$2+1+1$	0.0607–0.0863	Three lattice spacings fixed through $F_\pi /M_\pi$
ETM 10	[98]	$2+1+1$	0.078, 0.086	Two lattice spacings fixed through $F_\pi /M_\pi$
BMW 13	[254]	$2+1$	0.054–0.093	Scale set through Omega baryon mass
RBC/UKQCD 12	[25]	$2+1$	0.086, 0.114 and 0.144 for $M_\pi ^\mathrm{min}$	Scale set through Omega baryon mass
Borsanyi 12	[249]	$2+1$	0.097–0.284	Scale fixed through $F_\pi /M_\pi$
NPLQCD 11	[267]	$2+1$	0.09, 0.125	Configurations are shared with MILC 09 [15]
MILC 10, 10A	[75, 159]	$2+1$	0.045–0.09	Three lattice spacings, continuum extrapolation by means of RS$\chi$PT
JLQCD/TWQCD 10	[252]	$2+1$, 3	0.11	One lattice spacing, scale fixed through $m_\Omega$
RBC/UKQCD 9, 10A	[78, 287]	$2+1$	0.1106 (27), 0.0888 (12)	Two lattice spacings. Data combined in global chiral-continuum fits
JLQCD 09	[251]	$2+1$	0.1075 (7)	Scale fixed through $r_0$
MILC 09, 09A	[15, 37]	$2+1$	0.045–0.18	Total of six lattice spacings, continuum extrapolation by means of RS$\chi$PT
TWQCD 08	[255]	$2+1$	0.122 (3)	Scale fixed through $m_\rho$, $r_0$
JLQCD/TWQCD 08B	[256]	$2+1$	0.1075 (7)	Scale fixed through $r_0$
PACS-CS 08	[19]	$2+1$	0.0907	One lattice spacing
RBC/UKQCD 08	[79]	$2+1$	0.114	One lattice spacing, attempt to estimate cutoff effects via formal argument
RBC/UKQCD 08A	[253]	$2+1$	0.114	Only one lattice spacing, attempt to estimate size of cutoff effects via formal argument
NPLQCD 06	[166]	$2+1$	0.125	One lattice spacing, continuum $\chi$PT used
LHP 04	[275]	$2+1$	$\simeq 0.12$	Only one lattice spacing, mixed discretisation approach

**Table 64:** Continuum extrapolations/estimation of lattice artefacts in $N_\mathrm{f}=2$ determinations of the low-energy constants

Collab.	Ref.	$N_\mathrm{f}$	$a$ (fm)	Description
Gülpers 13	[270]	2	0.063	Scale fixed through omega Baryon mass
Brandt 13	[257]	2	0.05–0.08	Configurations are shared with CLS
QCDSF 13	[268]	2	0.06–0.076	Scale fixed through $r_0 = 0.50(1)$ fm
ETM 13	[217]	2	0.05–0.1	Configurations are shared with ETM 09C
ETM 12	[258]	2	0.05–0.1	Configurations are shared with ETM 09C
Bernardoni 11	[259]	2	0.0649 (10)	Configurations are shared with CLS
TWQCD 11	[185]	2	0.1034 (1) (2)	Scale fixed through $r_0$
TWQCD 11A	[260]	2	0.1032 (2)	Scale fixed through $r_0$
Bernardoni 10	[261]	2	0.0784 (10)	Scale fixed through $M_K$. Non-perturbative $O(a)$ improvement. No estimate of systematic error
JLQCD/TWQCD 09, 10	[252]	2	0.11	One lattice spacing fixed through $r_0$
ETM 09B	[263]	2	0.063, 0.073	Automatic $O(a)$ impr. $r_0=0.49$ fm used
ETM 09C	[241]	2	0.051–0.1	Automatic $O(a)$ impr. Scale fixed through $F_\pi$. Four lattice spacings, continuum extrapolation
ETM 08	[238]	2	0.07–0.09	Automatic $O(a)$ impr. Two lattice spacings. Scale fixed through $F_\pi$
JLQCD/TWQCD 08A	[67]	2	0.1184 (3) (21)	Automatic $O(a)$ impr., exact chiral symmetry. Scale fixed through $r_0$
JLQCD 08A	[286]			
CERN 08	[215]	2	0.0784 (10)	Scale fixed through $M_K$. Non-perturbative $O(a)$ improvement
Hasenfratz 08	[264]	2	0.1153 (5)	Tree-level $$ O(a)$$ improvement. Scale fixed through $$r_0$$. Estimate of lattice artefacts via W$$\chi $$PT [692]
JLQCD/TWQCD 07	[265]	2	0.1111 (24)	Automatic $O(a)$ impr., exact chiral symmetry. Scale fixed through $r_0$
JLQCD/TWQCD 07A	[262]	2	$\simeq$ 0.12	Automatic $O(a)$ impr., exact chiral symmetry. Scale fixed through $r_0$
CERN-TOV 06	[272]	2	0.0717 (15), 0.0521 (7), 0.0784 (10)	Scale fixed through $M_K$. The lattice with $a=0.0784\,(10)$ is obtained with non-perturbative $O(a)$ improvement
QCDSF/UKQCD 06A	[276]	2	0.07–0.115	5 lattice spacings. Non-perturbative $O(a)$ improvement. Scale fixed through $r_0$

**Table 65:** Chiral extrapolation/minimum pion mass in $N_\mathrm{f}=2+1+1$ and $2+1$ determinations of the low-energy constants

Collab.	Ref.	$N_\mathrm{f}$	$M_{\pi ,\text {min}}$ (MeV)	Description
HPQCD 13A	[156]	$2+1+1$	128	NLO chiral fit
ETM 13	[217]	$2+1+1$	270	Linear fit in the quark mass
ETM 11	[266]	$2+1+1$	270	NLO SU(2) chiral fit
ETM 10	[98]	$2+1+1$	$270$	SU(2) NLO and NNLO fits
BMW 13	[254]	$2+1$	120	NLO and NNLO SU(2) fits tested with x and $\xi$ expansion
RBC/UKQCD 12	[25]	$2+1$	293 plus run at 171, 246	NLO SU(2) ChPT incl. finite-V and some discr. effects
Borsanyi 12	[249]	$2+1$	135	NNLO SU(2) chiral fit
NPLQCD 11	[267]	$2+1$	235	NNLO SU(2) mixed action $\chi$PT
MILC 10, 10A	[75, 159]	$2+1$		Cf. MILC 09A
JLQCD/TWQCD 09, 10	[252]	$2+1,3$	100($\epsilon$-reg.), 290($p$-reg.)	$N_\mathrm{f}=2+1$ runs both in $\epsilon$- and $p$-regime; $N_\mathrm{f}=3$ runs only in $p$-regime. NLO $\chi$PT fit of the spectral density interpolating the two regimes
RBC/UKQCD 9, 10A	[78, 287]	$2+1$	290–420	Valence pions mass is 225–420 MeV. NLO SU(2) $\chi$PT fit
MILC 09, 09A	[15, 37]	$2+1$	$258$	Lightest Nambu–Goldstone mass is 224 MeV and lightest RMS mass is 258 MeV (at 0.06 fm)
TWQCD 08	[255]	$2+1$	$m_{ud}=m_{s}/4$, $m_{s}\sim$ phys	Quark condensate extracted from topological susceptibility, LO chiral fit
JLQCD/TWQCD 08B	[256]	$2+1$	$m_{ud}\ge m_{s}/6$, $m_{s}\sim$ phys	Quark condensate extracted from topological susceptibility, LO chiral fit
PACS-CS 08	[19]	$2+1$	$156$	To date, lightest published quark mass reached in a direct simulation
RBC/UKQCD 08	[79]	$$ 2+1$$	$$330$$	Lightest valence pion mass is $$242\,{\mathrm {MeV}}$$
RBC/UKQCD 08A	[253]	$2+1$	330	Pion electromagnetic form factor computed at one pion mass
NPLQCD 06	[166]	$2+1$	$460$	Value refers to lightest RMS mass at $a=0.125$ fm as quoted in [37]
LHP 04	[275]	$2+1$	$318$	Pion form factor extracted from vector-meson dominance fit

**Table 66:** Chiral extrapolation/minimum pion mass in $N_\mathrm{f}=2$ determinations of the low-energy constants

Collab.	Ref.	$N_\mathrm{f}$	$M_{\pi ,\text {min}}$ (MeV)	Description
Gülpers 13	[270]	2	280	NLO ChPT fit
Brandt 13	[257]	2	280	Configurations are shared with CLS
QCDSF 13	[268]	2	130	Fit with ChPT + analytic
ETM 13	[217]	2	260	Configurations are shared with ETM 09C
ETM 12	[258]	2	260	Configurations are shared with ETM 09C
Bernardoni 11	[259]	2	312	Overlap valence + $O(a)$ improved Wilson sea, mixed regime $\chi$PT
TWQCD 11	[185]	2	230	NLO SU(2) $\chi$PT fit
TWQCD 11A	[260]	2	220	NLO $\chi$PT (infinite $V$) for topological susceptibility $\chi _\mathrm{top}$
Bernardoni 10	[261]	2	297, 377, 426	NLO SU(2) fit of $\chi _\mathrm{top}$
JLQCD/TWQCD 10	[252]	2	$\sqrt{2m_\mathrm{min}\Sigma }/F=120$ ($\epsilon$-reg.), 290 ($p$-reg.)	Data both in the $p$ and $\epsilon$-regime. NLO chiral fit of the spectral density interpolating the two regimes
JLQCD/TWQCD 09	[271]	2	290	LECs extracted from NNLO chiral fit of vector and scalar radii ${\langle }r^2\rangle ^\pi _{V,S}$
ETM 09B	[263]	2	$\sqrt{2m_\mathrm{min}\Sigma }/F=85$	NLO SU(2) $\epsilon$-regime fit
ETM 09C	[241]	2	$280$	NNLO SU(2) fit
ETM 08	[238]	2	$260$	From pion form factor using NNLO $\chi$PT and exp. value of ${\langle }r^2\rangle ^\pi _{S}$
JLQCD/TWQCD 08A	[67]	2	290	NNLO SU(2) fit
JLQCD 08A	[286]			
CERN 08	[215]	2	$m_{q,\text {min}}=13$ MeV	NLO SU(2) fit for the mode number
Hasenfratz 08	[264]	2	$\sqrt{2m_\mathrm{min}\Sigma }/F=220$	NLO SU(2) $\epsilon$-regime fit
JLQCD/TWQCD 07	[265]	2	$\sqrt{2m_\mathrm{min}\Sigma }/F=120$	NLO SU(2) $\epsilon$-regime fit
JLQCD/TWQCD 07A	[262]	2	$m_{ud}= m_{s}/6-m_{s}$	Quark condensate from topological susceptibility, LO chiral fit
CERN-TOV 06	[272]	2	403, 381, 377	NLO SU(2) fit
QCDSF/UKQCD 06A	[276]	2	400	Several fit functions to extrapolate the pion form factor

**Table 67:** Finite-volume effects in $N_\mathrm{f}=2+1+1$ and $2+1$ determinations of the low-energy constants

Collab.	Ref.	$N_\mathrm{f}$	$L$ (fm)	$M_{\pi ,\text {min}}L$	Description
HPQCD 13A	[156]	$2+1+1$	4.8–5.5	3.3	Three volumes are compared
ETM 13	[217]	$2+1+1$	1.9–2.8	3.0	Four volumes compared
ETM 11	[266]	$2+1+1$	1.9–2.8	3.0	See [98]
ETM 10	[98]	$2+1+1$	1.9–2.8	3.0	FSE estimate using [689]. $M_{\pi ^+} L\gtrsim 4$, but $M_{\pi ^0} L \sim 2$
BMW 13	[254]	$2+1$	2.1	3.0	Three volumes are compared
RBC/UKQCD 12	[25]	$2+1$	2.7–4.6	$>$4	FSE seem to be very small
Borsanyi 12	[249]	$2+1$	3.9	3.3	Expected to be less than 1 %
NPLQCD 11	[267]	$2+1$	2.5–3.5	3.6	Expected to be less than 1 %
MILC 10, 10A	[75, 159]	$2+1$	2.52	4.11	$L \ge 2.9\,\,{\mathrm {fm}}$ for lighter masses
JLQCD/TWQCD 09, 10	[252]	$2+1$, 3	1.9, 2.7		Two volumes are compared for a fixed quark mass
RBC/UKQCD 9, 10A	[78, 287]	$2+1$	2.7	$\simeq$4	FSE estimated using $\chi$PT
MILC 09, 09A	[15, 37]	$2+1$	2.4/2.9	3.5/4.11	$L \ge 2.9\,\,{\mathrm {fm}}$ for lighter masses
TWQCD 08	[255]	$2+1$	1.95	–	No estimate of FSE
JLQCD/TWQCD 08B	[256]	$2+1$	1.72	–	Fixing topological charge (to $\nu =0$) gives FSE [693]
PACS-CS 08	[19]	$2+1$	2.9	2.3	FSE is the main concern of the authors
RBC/UKQCD 08	[79]	$2+1$	2.74	4.6	FSE by means of $\chi$PT
RBC/UKQCD 08A	[253]	$2+1$	2.74	4.6	FSE estimated to be $<$1 % using $\chi$PT
NPLQCD 06	[166]	$2+1$	2.5	3.7	Value refers to lightest valence pion mass
LHP 04	[275]	$2+1$	$\simeq$2.4	3.97	Value refers to domain-wall valence pion mass

**Table 68:** Finite-volume effects in $N_\mathrm{f}=2$ determinations of the low-energy constants

Collab.	Ref.	$N_\mathrm{f}$	$L$ (fm)	$M_{\pi ,\text {min}}L$	Description
Gülpers 13	[270]	2	4–6	4.3	Configs. shared with CLS
Brandt 13	[257]	2	$\sim 5$	4	Configs. shared with CLS
QCDSF 13	[268]	2	1.8–2.4	2.7	NLO ChPT is used for FSE
ETM 13	[217]	2	2.0–2.5	3–4	Configs. shared with ETM 09C
ETM 12	[258]	2	2.0–2.5	3–4	Configs. shared with ETM 09C
Bernardoni 11	[259]	2	1.56	2.5	Mixed regime $\chi$PT for FSE used
TWQCD 11	[185]	2	1.65	1.92	SU(2) $\chi$PT is used for FSE
TWQCD 11A	[260]	2	1.65	1.8	No estimate of FSE
Bernardoni 10	[261]	2	1.88	2.8	FSE included in the NLO chiral fit
JLQCD/TWQCD 10	[252]	2	1.8–1.9		FSE estimated from different topological sectors
JLQCD/TWQCD 09	[271]	2	1.89	2.9	FSE by NLO $\chi$PT, Additional FSE for fixing topology [693]
ETM 09B	[263]	2	1.3, 1.5	$\epsilon$-regime	Topology: not fixed. 2 volumes
ETM 09C	[241]	2	2.0–2.5	3.2–4.4	Several volumes. Finite-volume effects estimated through [689]
ETM 08	[238]	2	2.1, 2.8	3.4, 3.7	Only data with $M_\pi L\gtrsim 4$ are considered
JLQCD/TWQCD 08A	[67]	2	1.89	2.9	FSE estimates through [689]. Additional FSE for fixing topology [693]
JLQCD 08A	[286]				
CERN 08	[215]	2	1.88, 2.51	–	Two volumes compared
Hasenfratz 08	[264]	2	1.84, 2.77	$\epsilon$-regime	Topology: not fixed, 2 volumes
JLQCD/TWQCD 07	[265]	2	1.78	$\epsilon$-regime	Topology: fixed to $\nu =0$
JLQCD/TWQCD 07A	[262]	2	1.92	–	Topology fixed to $\nu =0$ [693]
CERN-TOV 06	[272]	2	1.72, 1.67, 1.88	3.5, 3.2, 3.6	No estimate for FSE
QCDSF/UKQCD 06A	[276]	2	1.4–2.0	3.8	NLO $\chi$PT estimate for FSE [694]

**Table 69:** Renormalisation in determinations of the low-energy constants

Collab.	Ref.	$N_\mathrm{f}$	Description
HPQCD 13A	[156]	$2+1+1$	–
ETM 13	[217]	$2+1+1$	Non-perturbative
ETM 11	[266]	$2+1+1$	Non-perturbative
ETM 10	[98]	$2+1+1$	Non-perturbative
BMW 13	[254]	$2+1$	Non-perturbative
RBC/UKQCD 12	[25]	$2+1$	Non-perturbative (RI/SMOM)
Borsanyi 12	[249]	$2+1$	Indirectly non-perturbative through [22] for $\Sigma$; no renormalisation needed for $F$, since only $F_\pi /F$ computed and scale set through $F_\pi$
NPLQCD 11	[267]	$2+1$	Not needed (no result for $\Sigma$)
JLQCD/TWQCD 10	[252]	$2+1$, 3	Non-perturbative
MILC 10, 10A	[75, 159]	$2+1$	2 loop
RBC/UKQCD 10A	[78]	$2+1$	Non-perturbative
JLQCD 09	[251]	$2+1$	Non-perturbative
MILC 09, 09A	[15, 37]	$2+1$	2 loop
TWQCD 08	[255]	$2+1$	Non-perturbative
JLQCD/TWQCD 08B	[256]	$2+1$	Non-perturbative
PACS-CS 08	[19]	$2+1$	1 loop
RBC/UKQCD 08, 08A	[79, 253]	$2+1$	Non-perturbative
NPLQCD 06	[166]	$2+1$	–
LHP 04	[275]	$2+1$	–
All collaborations		2	Non-perturbative

### B.4 Notes to Sect. 6 on Kaon $B$-parameter $B_K$

In the following, we summarise the characteristics (lattice actions, pion masses, lattice spacings, etc.) of the recent $N_\mathrm{f}= 2+1$ and $N_\mathrm{f}= 2$ runs. We also provide brief descriptions of how systematic errors are estimated by the various authors (Tables 70, 71, 72, 73).

**Table 70:** Continuum extrapolations/estimation of lattice artefacts in determinations of $B_K$

Collab.	Ref.	$N_\mathrm{f}$	$a$ (fm)	Description
SWME 13	[316]	$2+1$	0.12, 0.09, 0.06, 0.045	Continuum extrapolation with the coarsest lattice spacing omitted; residual combined discretisation and sea-quark extrapolationg error of 1.1 % from difference between linear fit in $a^2$, $m_\mathrm{sea}$ and a constrained nine-parameter extrapolation
RBC/UKQCD 12	[25]	$2+1$	0.146, 0.114, 0.087	Coarsest lattice spacing uses different action. Combined continuum and chiral fits
Laiho 11	[77]	$2+1$	0.12, 0.09, 0.06	Combined continuum and chiral extrapolation based on SU(3) mixed-action partially quenched $\chi$PT
SWME 11, 11A	[299, 300]	$2+1$	0.12, 0.09, 0.06, 0.045	Continuum extrapolation with the coarsest lattice spacing omitted; residual discretisation error of 1.9 % from difference between fit to a constant and a constrained five-parameter extrapolation
BMW 11	[301]	$2+1$	0.093, 0.077, 0.065, 0.054	Combined continuum and chiral extrapolation; discretisation error of 0.1 % from comparison of $O({\alpha _\mathrm{s}}a)$ and $O(a^2)$ extrapolations
RBC/UKQCD 10B	[315]	$2+1$	0.114, 0.087	Two lattice spacings. Combined chiral and continuum fits
SWME 10	[317]	$2+1$	0.12, 0.09, 0.06	Continuum extrapolation of results obtained at four lattice spacings; residual discretisation error of 0.21 % from difference to result at smallest lattice spacing
Aubin 09	[298]	$2+1$	0.12, 0.09	Two lattice spacings; quote 0.3 % discretisation error, estimated from various $a^2$-terms in fit function
RBC/UKQCD 07A, 08	[79, 318]	$2+1$	0.114(2)	Single lattice spacing; quote 4 % discretisation error, estimated from the difference between computed and experimental values of $f_\pi$
HPQCD/UKQCD 06	[319]	$2+1$	0.12	Single lattice spacing; 3 % discretisation error quoted without providing details
ETM 10A	[314]	2	0.1, 0.09, 0.07	Three lattice spacings; 1.2 % error quoted
JLQCD 08	[320]	2	0.118 (1)	Single lattice spacing; no error quoted
RBC 04	[313]	2	0.117 (4)	Single lattice spacing; no error quoted
UKQCD 04	[321]	2	0.10	Single lattice spacing; no error quoted

**Table 71:** Chiral extrapolation/minimum pion mass in determinations of $B_K$

Collab.	Ref.	$N_\mathrm{f}$	$M_{\pi ,\mathrm min}\,(\mathrm{MeV})$	Description
SWME 13	[316]	$2+1$	442/445, 299/273, 237/256, 222/334	Valence/sea RMS $M_{\pi ,\mathrm min}$ entries correspond to the four lattice spacings. Chiral extrapolations based on SU(2) staggered $\chi$PT at NNLO (with some coefficients fixed by Bayesian priors), and also including one analytic NNNLO term. Residual error of 0.33 % error from doubling the widths of Bayesian priors
RBC/UKQCD 12	[25]	$2+1$	140/170, 240/330, 220/290	Valence/sea $M_{\pi ,\mathrm min}$ entries correspond to the three lattice spacings. Combined chiral & continuum extrapolation, using $M_\pi <350\;$MeV
Laiho 11	[77]	$2+1$	210/280	$M_{\pi ,\mathrm min}$ entries correspond to the smallest valence/sea quark masses. Chiral & continuum fits based on NLO mixed action $\chi$PT, including a subset of NNLO terms. Systematic error estimated from spread arising from variations in the fit function
SWME 11, 11A	[299, 300]	$2+1$	442/445, 299/325, 237/340, 222/334	Valence/sea RMS $M_{\pi ,\mathrm min}$ entries correspond to the four lattice spacings. Chiral extrapolations based on SU(2) staggered $\chi$PT at NNLO (with some coefficients fixed by Bayesian priors), and also including one analytic NNNLO term. Residual error of 0.33 % error from doubling the widths of Bayesian priors
BMW 11	[301]	$2+1$	219, 182, 120, 131	$M_{\pi ,\mathrm min}$ entries correspond to the four lattice spacings used in the final result. Combined fit to the chiral and continuum behaviour. Systematics investigated by applying cuts to the maximum pion mass used in fits. Uncertainty of 0.1 % assigned to chiral fit
RBC/UKQCD 10B	[315]	$2+1$	240/330, 220/290	Valence/sea $M_{\pi ,\mathrm min}$ entries correspond to the two lattice spacings. Combined chiral and continuum extrapolations
SWME 10	[317]	$2+1$	442/445, 299/325, 237/340	Valence/sea $M_{\pi ,\mathrm min}$ entries correspond to the three lattice spacings. Chiral extrapolations based on SU(2) staggered $\chi$PT at NLO, including some analytic NNLO terms. SU(3) staggered $\chi$PT as cross-check. Combined 1.1 % error from various different variations in the fit procedure
Aubin 09	[298]	$2+1$	240/370	$M_{\pi ,\mathrm min}$ entries correspond to the smallest valence/sea quark masses. Chiral and continuum fits based on NLO mixed-action $\chi$PT at NLO, including a subset of NNLO terms. Systematic error estimated from spread arising from variations in the fit function
RBC/UKQCD 07A, 08	[79, 318]	$2+1$	330	Fits based on SU(2) PQ$\chi$PT at NLO. Effect of neglecting higher orders estimated at 6 % via difference between fits based on LO and NLO expressions
HPQCD/UKQCD 06	[319]	$2+1$	360	3 % uncertainty from chiral extrapolation quoted, without giving further details
ETM 10A	[314]	2	400, 270, 300	Each $M_{\pi ,\mathrm min}$ entry corresponds to a different lattice spacing. Simultaneous chiral & continuum extrapolations, based on $\chi$PT at NLO, are carried out. Systematic error from several sources, including lattice calibration, quark mass calibration, chiral and continuum extrapolation etc., estimated at 3.1 %
JLQCD 08	[320]	2	290	Fits based on NLO PQ$\chi$PT. Range of validity investigated. Fit error included in statistical uncertainty
RBC 04	[313]	2	490	Fits based on NLO PQ$\chi$PT. Fit error included in statistical uncertainty
UKQCD 04	[321]	2	780	Fits to continuum chiral behaviour at fixed sea quark mass. Separate extrapolation in sea quark mass. Fit error included in overall uncertainty

**Table 72:** Finite-volume effects in determinations of $B_K$. If partially quenched fits are used, the quoted $M_{\pi ,\mathrm min}L$ is for lightest valence (RMS) pion

Collab.	Ref.	$N_\mathrm{f}$	$L$ (fm)	${M_{\pi ,\mathrm{min}}}L$	Description
SWME 13	[316]	$2+1$	2.4–3.3, 2.4–5.5, 2.8–3.8, 2.8	$\gtrsim$3.2	$L$ entries correspond to the four lattice spacings, with several volumes in most cases. Finite-volume effects estimated using NLO $\chi$PT
RBC/UKQCD 12	[25]	$2+1$	4.6, 2.7, 2.8	$\gtrsim$3.2	$L$ entries correspond to the three lattice spacings. Finite-volume effects estimated using NLO $\chi$PT
Laiho 11	[77]	$2+1$	2.4, 3.4, 3.8	$\gtrsim$3.5	$L$ entries correspond to the three lattice spacings. Finite-volume effects estimated using NLO $\chi$PT
SWME 11, 11A	[299, 300]	$2+1$	2.4/3.3, 2.4, 2.8, 2.8	$\gtrsim$3.2	$L$ entries correspond to the four lattice spacings, with two volumes at the coarsest lattice. Finite-volume effects estimated using NLO $\chi$PT
BMW 11	[301]	$2+1$	6.0, 4.9, 4.2, 3.5	$\gtrsim$3.8, 3.0	$L$ entries correspond to the four lattice spacings, and they are the largest of several volumes at each $a$. ${M_{\pi ,\mathrm min}}L\approx 3.0$ for the ensemble at $a\approx 0.08\,\,{\mathrm {fm}}$. Finite-volume effects estimated in $\chi$PT and by combined fit to multiple volumes
RBC/UKQCD 10B	[315]	$2+1$	2.7, 2.8	$\gtrsim 3.1$	$L$ entries correspond to the three lattice spacings. Finite-volume effects estimated using NLO $\chi$PT
SWME 10	[317]	$2+1$	2.4/3.3, 2.4,2.8	$\gtrsim 3.4$	$L$ entries correspond to the three lattice spacings, with two volumes for the coarsest spacing. Finite-volume error of 0.9 % estimated from difference obtained these two volumes
Aubin 09	[298]	$2+1$	2.4, 3.4	3.5	$L$ entries correspond to the two lattice spacings. Keep $m_{\pi }L\;\gtrsim \;3.5$; no comparison of results from different volumes; 0.6 % error estimated from mixed-action $\chi$PT correction
RBC/UKQCD 07A, 08	[79, 318]	$2+1$	1.83/2.74	4.60	Each $L$ entry corresponds to a different volume at the same lattice spacing; 1 % error from difference in results on two volumes
HPQCD/UKQCD 06	[319]	$2+1$	2.46	4.49	Single volume; no error quoted
ETM 10A	[314]	2	2.1, 2.2/2.9, 2.2	5, 3.3/4.3, 3.3	Each $L$ entry corresponds to a different lattice spacing, with two volumes at the intermediate lattice spacing. Results from these two volumes at $M_{\pi } \sim 300$ MeV are compatible
JLQCD 08	[320]	2	1.89	2.75	Single volume; data points with $m_\mathrm{val} < m_\mathrm{sea}$ excluded; 5 % error quoted as upper bound of PQ$\chi$PT estimate of the effect
RBC 04	[313]	2	1.87	4.64	Single volume; no error quoted
UKQCD 04	[321]	2	1.6	6.51	Single volume; no error quoted

**Table 73:** Running and matching in determinations of $B_K$

SWME 13	[316]	$2+1$	PT1$\ell$	PT1$\ell$	Uncertainty from neglecting higher orders estimated at 4.4 % by identifying the unknown two-loop coefficient with result at the smallest lattice spacing
RBC/UKQCD 12	[25]	$2+1$	RI	PT1$\ell$	Two different RI-SMOM schemes used to estimate 2 % systematic error in conversion to ${\overline{\mathrm{MS}}}$
Laiho 11	[77]	$2+1$	RI	PT1$\ell$	Total uncertainty in matching & running of 3 %. Perturbative truncation error in the conversion to ${\overline{\mathrm{MS}}}$, RGI schemes is dominant uncertainty
SWME 11, 11A	[299, 300]	$2+1$	PT1$\ell$	PT1$\ell$	Uncertainty from neglecting higher orders estimated at 4.4 % by identifying the unknown two-loop coefficient with result at the smallest lattice spacing
BMW 11	[301]	$2+1$	RI	PT1$\ell$	Uncertainty of 0.05 % in the determination of the renormalisation factor included. 1 % error estimated due to truncation of perturbative matching to ${\overline{\mathrm{MS}}}$ and RGI schemes at NLO
RBC/UKQCD 10B	[315]	$2+1$	RI	PT1$\ell$	Variety of different RI-MOM schemes including non-exceptional momenta. Residual uncertainty of 2 % uncertainty in running & matching
SWME 10	[317]	$2+1$	PT1$\ell$	PT1$\ell$	Uncertainty from neglecting higher orders estimated at 5.5 % by identifying the unknown two-loop coefficient with result at the smallest lattice spacing
Aubin 09	[298]	$2+1$	RI	PT1$\ell$	Total uncertainty in matching and running of 3.3 %, estimated from a number of sources, including chiral-extrapolation fit ansatz for n.p. determination, strange sea quark mass dependence, residual chiral symmetry breaking, perturbative matching and running
RBC/UKQCD 07A, 08	[79, 318]	$2+1$	RI	PT1$\ell$	Uncertainty from n.p. determination of ren. factor included in statistical error; 2 % systematic error from perturbative matching to ${\overline{\mathrm{MS}}}$ estimated via size of correction itself
HPQCD/UKQCD 06	[319]	$2+1$	PT1$\ell$	PT1$\ell$	Uncertainty due to neglecting two-loop order in perturbative matching and running estimated by multiplying result by $\alpha ^2$
ETM 10A	[314]	2	RI	PT1$\ell$	Uncertainty from RI renormalisation estimated at 2.5 %
JLQCD 08	[320]	2	RI	PT1$\ell$	Uncertainty from n.p. determination of ren. factor included in statistical error; 2.3 % systematic error from perturbative matching to ${\overline{\mathrm{MS}}}$ estimated via size of correction itself
RBC 04	[313]	2	RI	PT1$\ell$	Uncertainty from n.p. determination of ren. factor included
UKQCD 04	[321]	2	PT1$\ell$	PT1$\ell$	No error quoted

### B.5 Notes to Sect. 7 on $D$-meson decay constants and form factors

In the following, we summarise the characteristics (lattice actions, pion masses, lattice spacings, etc.) of the recent $N_\mathrm{f}= 2+1$ and $N_\mathrm{f}= 2$ runs. We also provide brief descriptions of how systematic errors are estimated by the various authors. We focus on calculations with either preliminary or published quantitative results.

#### B.5.1 $D_{(s)}$-meson decay constants

See Tables 74, 75, 76, 77, 78, 79, 80.

**Table 74:** Lattice spacings and description of actions used in $N_\mathrm{f}=2+1+1$ determinations of the $D$- and $D_{s}$-meson decay constants

Collab.	Ref.	$N_\mathrm{f}$	$a$ (fm)	Continuum extrapolation	Scale setting
ETM 13F	[155]	$2+1+1$	0.09, 0.08, 0.06	Chiral and continuum extrapolations performed simultaneously by adding an $O(a^2)$ term to the chiral fit	Relative scale set through $M_{c's'}$, the mass of a fictitious meson made of valence quarks of mass $r_0m_{s'}\!=0.22$ and $r_0m_{c'}=2.4$. Absolute scale through $f_\pi$
FNAL/MILC 12B FNAL/MILC 13	[329, 330]	$2+1+1$	0.15, 0.12, 0.09, 0.06	Chiral and continuum extrapolations performed simultaneously. Central values produced using a fit function quadratic in $a^2$ and linear in the sea quark mass. In FNAL/MILC 13 terms of $O(a^4)$ are included	Absolute scale set through $f_\pi$; the uncertainty is propagated into the final error

**Table 75:** Lattice spacings and description of actions used in $N_\mathrm{f}=2+1$ determinations of the $D$- and $D_{s}$-meson decay constants

Collab.	Ref.	$N_\mathrm{f}$	$a$ (fm)	Continuum extrapolation	Scale setting
HPQCD 12A	[331]	$2+1$	0.12, 0.09	Chiral and continuum extrapolations performed simultaneously using PQHM$\chi$PT augmentd by $a$-dependent terms: $c_0(am_{c})^2 + c_1(am_{c})^4$	Relative scale set through $r_1$; absolute scale from $f_\pi$, $f_{K}$ and the $\Upsilon$ splitting. Uncertainties from both $r_1$ and $r_1/a$ propagated
FNAL/MILC 11	[332]	$2+1$	0.15, 0.12, 0.09	Chiral and continuum extrapolations performed simultaneously using one-loop HM$\chi$PT for rooted staggered quarks. Effects of hyperfine and flavour splittings are also included	Relative scale set through $r_1=0.3117(22)$. The error in $r_1$ comes from the spread of different absolute scale determinations using $f_\pi$, $f_{K}$ and the $\Upsilon$ splitting
PACS-CS 11	[333]	$2+1$	$0.09$	Cutoff effects from the heavy-quark action estimated by naive power counting to be at the percent level	Scale set through $m_\Omega$
HPQCD 10A	[94]	$2+1$	0.15, 0.12, 0.09, 0.06, 0.044	Chiral and continuum extrapolations performed simultaneously. Polynomials up to $am_{c}^8$ are kept (even powers only)	See the discussion for HPQCD 12A
HPQCD/UKQCD 07	[165]	$2+1$	0.15, 0.12, 0.09	Combined chiral and continuum extrapolations using HM$\chi$PT at NLO augmented by second and third-order polynomial terms in $m_{q}$ and terms up to $a^4$	Scale set through $r_1$ obtained from the $\Upsilon$ spectrum using the non-relativistic QCD action for $b$ quarks. Uncertainty propagated among the systematics
FNAL/MILC 05	[334]	$2+1$	0.175, 0.121, 0.086	Most light-quark cutoff effects are removed through NLO HM$\chi$PT for rooted staggered quarks. Continuum values are then obtained by averaging the $a \approx 0.12$ and $a \approx 0.09$ fm results	Scale set through $r_1$ obtained from the $\Upsilon$ spectrum using the non-relativistic QCD action for $b$ quarks

**Table 76:** Lattice spacings and description of actions used in $N_\mathrm{f}=2$ determinations of the $D$- and $D_{s}$-meson decay constants

Collab.	Ref.	$N_\mathrm{f}$	$a$ (fm)	Continuum extrapolation	Scale setting
ETM 09 ETM 11A ETM 13B	[169, 335, 336]	2	0.10, 0.085, 0.065, 0.054	NLO SU(2) HM$\chi$PT supplemented by terms linear in $a^2$ and in $m_D a^2$ is used in the combined chiral/continuum extrapolation	Scale set through $f_\pi$

**Table 77:** Chiral extrapolation/minimum pion mass in determinations of the $D$- and $D_{s}$-meson decay constants. For actions with multiple species of pions, masses quoted are the RMS pion masses. The different $M_{\pi ,\mathrm min}$ entries correspond to the different lattice spacings

Collab.	Ref.	$N_\mathrm{f}$	$M_{\pi ,\mathrm min}\,(\mathrm{MeV})$	Description
ETM 13F	[155]	$2+1+1$	245, 238, 211	$f_{D_{s}}$ is extrapolated using both a quadratic and a linear fit in $m_{l}$ plus $O(a^2)$ terms. Then the double ratio $(f_{D_{s}}/f_D)/(f_K/f_\pi )$ is fitted in continuum HM$\chi$PT, as no lattice spacing dependence is visible within statistical errors
FNAL/MILC 12B FNAL/MILC 13	[329, 330]	$2+1+1$	310, 245, 179, 145	Chiral and continuum extrapolations are performed simultaneously. Central values are produced using a fit function quadratic in $a^2$ and linear in the sea-quark mass. In FNAL/MILC 13 terms of $O(a^4)$ are included
HPQCD 12A	[331]	$2+1$	460, 329	Chiral and continuum extrapolations are performed simultaneously using PQHM$\chi$PT augmented by $a$-dependent terms: $c_0(am_{c})^2 + c_1(am_{c})^4$
FNAL/MILC 11	[332]	$2+1$	570, 440, 320	Chiral and continuum extrapolations are performed simultaneously using HM$\chi$PT for rooted staggered quarks. Effects of hyperfine and flavour splittings are also included
PACS-CS 11	[333]	$2+1$	152	Simulations are reweighted in the light- and strange-quark masses to the physical point
HPQCD 10A	[94]	$2+1$	542, 460, 329, 258, 334	Chiral and continuum extrapolations are performed simultaneously. Polynomials up to $(\frac{m_{q,\mathrm{sea}}-m_{q,\mathrm{phys}}}{m_{q,\mathrm{phys}}})^2$ for $q=s,l$ and up to $(am_{c})^8$ are kept
HPQCD/UKQCD 07	[165]	$2+1$	542, 460, 329	Combined chiral and continuum extrapolations using HM$\chi$PT at NLO augmented by second and third-order polynomial terms in $m_{q}$ and terms up to $a^4$
FNAL/MILC 05	[334]	$2+1$	$>$440, 440, 400	Chiral extrapolations are first performed at each lattice spacing using NLO HM$\chi$PT for rooted staggered quarks. Lattice artefacts are then extrapolated linearly in $a^2$
ETM 09 ETM 11A ETM 13B	[169, 335, 336]	2	410, 270, 310, 270	$M_{\pi ,\mathrm{min}}$ refers to the charged pions. NLO SU(2) HM$\chi$PT supplemented by terms linear in $a^2$ and in $m_D a^2$ is used in the combined chiral/continuum extrapolation. To estimate the systematic due to chiral extrapolation, once $f_{D_{s}}\sqrt{m_{D_{s}}}$ and $f_{D_{s}}\sqrt{m_{D_{s}}}/(f_{D}\sqrt{m_{D}})$ and once $f_{D_{s}}\sqrt{m_{D_{s}}}/f_K$ and $f_{D_{s}}\sqrt{m_{D_{s}}}/f_K \; \times \; f_\pi /(f_{D}\sqrt{m_{D}})$ are fitted. In ETM 13 the double ratio $(f_{D_{s}}/f_D)/(f_K/f_\pi )$ is fitted in HM$\chi$PT

**Table 78:** Finite-volume effects in determinations of the $D$- and $D_{s}$-meson decay constants. Each $L$-entry corresponds to a different lattice spacing, with multiple spatial volumes at some lattice spacings. For actions with multiple species of pions, the lightest masses are quoted

Collab.	Ref.	$N_\mathrm{f}$	$L$ (fm)	${M_{\pi ,\mathrm{min}}}L$	Description
ETM 13F	[155]	$$2+1+1$$	2.13/2.84, 1.96/2.61, 2.97	$$3.5, 3.2, 3.2 $$	The comparison of two different volumes at the two largest lattice spacings indicates that FV effects are below the statistical errors
FNAL/MILC 12B FNAL/MILC 13	[329, 330]	$2+1+1$	2.4/4.8, 2.88/5.76, 2.88/5.76, 2.88/5.76	$3.3, 3.9$, $3.7, 4$	FV errors estimated in $\chi$PT at NLO and, in FNAL/MILC 12B, by analysing otherwise identical ensembles with three different spatial sizes at $a=0.12$ fm and $m_{l}/m_{s}=0.1$
HPQCD 12A	[331]	$2+1$	2.4/2.8, 2.4/3.4	3.8, 4.2	FV errors estimated by comparing finite- and infinite-volume $\chi$PT
FNAL/MILC 11	[332]	$2+1$	2.4, 2.4/2.88, 2.52/3.6	3.9, 3.8, 4,2	FV errors estimated using finite-volume $\chi$PT
PACS-CS 11	[333]	$2+1$	$2.88$	2.2 (before reweighting)	No discussion of FSE
HPQCD 10A	[94]	$2+1$	2.4, 2.4/2.88/3.36, $2.52, 2.88$, 2.82	$3.9, 3.8$, $4.1, 4.5$, 4.6	FV errors estimated using finite- vs. infinite-volume $\chi$PT
HPQCD/UKQCD 07	[165]	$2+1$	2.4, 2.4/2.88, 2.52	3.9, 3.8, 4.1	FV errors estimated using finite- vs infinite-volume $\chi$PT
FNAL/MILC 05	[334]	$2+1$	2.8, 2.9, 2.5	$3.8, 3.8, 4.1$	FV errors estimated to be 1.5 % or less from $\chi$PT
ETM 09 ETM 11A ETM 13B	[169, 335, 336]	2	2.4, 2.0/2.7, 2.1, 2.6	5, 3.3, 3.3, 3.5	FV errors are found to be negligible by comparing results at $m_\pi L = 3.3$ and $m_\pi L=4.3$ for $m_\pi \simeq 310$ MeV

**Table 79:** Operator renormalisation in determinations of the $D$- and $D_{s}$-meson decay constants

Collab.	Ref.	$N_\mathrm{f}$	Ren.	Description
ETM 13F	[155]	$2+1+1$	–	The axial current is absolutely normalised
FNAL/MILC 12B	[329, 330]	$2+1+1$	–	The axial current is absolutely normalised
FNAL/MILC 13				
HPQCD 12A	[331]	$2+1$	–	The axial current is absolutely normalised
FNAL/MILC 11	[332]	$2+1$	mNPR	Two-loop and higher-order perturbative truncation errors estimated to be the full size of the one-loop term
PACS-CS 11	[333]	$2+1$	PT1$\ell$ + NP	Mass dependent part of the renormalisation constant of the axial current computed at one loop; the NP contribution is added in the chiral limit
HPQCD 10A	[94]	$2+1$	–	The axial current is absolutely normalised
HPQCD/UKQCD 07	[165]	$2+1$	–	The axial current is absolutely normalised
FNAL/MILC 05	[334]	$2+1$	mNPR	Errors due to higher-order corrections in the perturbative part are estimated to be $1.3\,\%$
ETM 09	[169, 335, 336]	2	–	The axial current is absolutely normalised
ETM 11A				
ETM 13B

**Table 80:** Heavy-quark treatment in determinations of the $D$- and $D_{s}$-meson decay constants

Collab.	Ref.	$N_\mathrm{f}$	Action	Description
ETM 13F	[155]	$2+1+1$	tmWil	$0.15 \lesssim am_{c} \lesssim 0.20$. $D(a_\mathrm{min}) \ge 2\,\%$ also when the relative scale is set through $M_{c's'}$
FNAL/MILC 12B FNAL/MILC 13	[329, 330]	$2+1+1$	HISQ (on HISQ)	$0.29<am_{c}<0.7$. Discretisation errors estimated using different fit ansätze to be $\approx 1.5\,\%$ for $f_{D_{(s)}}$
HPQCD 12A	[331]	$2+1$	HISQ	$0.41<am_{c}<0.62$. Heavy-quark discretisation errors estimated using different fit ansätze to be $\approx 1.2\,\%$
FNAL/MILC 11	[332]	$2+1$	Fermilab	Discretisation errors from charm quark estimated through a combination of Heavy Quark and Symanzik Effective Theories to be around 3 % for $f_{D_{(s)}}$ and negligible for the ratio
PACS-CS 11	[333]	$2+1$	Tsukuba	$am_{c}\approx 0.57$. Heavy-quark discretisation errors estimated to be at the percent level by power counting
HPQCD 10A	[94]	$2+1$	HISQ	$0.193< am_{c}<0.825$. Heavy-quark discretisation errors estimated by changing the fit-inputs to be $\approx 0.4\,\%$
HPQCD/UKQCD 07	[165]	$2+1$	HISQ	$0.43<am_{c}<0.85$. Heavy-quark discretisation errors estimated from the chiral/continuum fits to be $\approx$0.5 %. $\delta (a_\mathrm{min})$ slightly $>$1 for $f_{D_{s}}$
FNAL/MILC 05	[334]	$2+1$	Fermilab	Discretisation errors from charm quark estimated via heavy-quark power-counting at 4.2 % for $f_{D_{(s)}}$ and 0.5 % for the ratio
ETM 09	[169, 335, 336]	2	tmWil	$0.16<am_{c}<0.23$. $D(a_\mathrm{min})\approx 5\,\%$ in ETM 09
ETM 11A				
ETM 13B				

#### B.5.2 $D\rightarrow \pi \ell \nu$ and $D\rightarrow K\ell \nu$ form factors

See Tables 81, 82, 83, 84, 85.

**Table 81:** Continuum extrapolations/estimation of lattice artefacts in determinations of the $D\rightarrow \pi \ell \nu$ and $D\rightarrow K \ell \nu$ form factors

Collab.	Ref.	$N_\mathrm{f}$	$a$ (fm)	Continuum extrapolation	Scale setting
HPQCD 13C	[348]	$2+1$	0.09, 0.12	Modified $z$-expansion fit combining the continuum and chiral extrapolations and the momentum-transfer dependence	Relative scale $r_1/a$ set from the static quark potential. Absolute scale $r_1$ set from several quantities including $f_\pi$, $f_K$ and $\Upsilon \,2S$–$1S$ splitting cf. HPQCD 09B [186]
HPQCD 10B, 11	[338, 342]	$2+1$	0.09, 0.12	Modified $z$-expansion fit combining the continuum and chiral extrapolations and the momentum-transfer dependence. Leading discretisation errors from $(am_{c})^n$ charm-mass effects (see Table 85). Subleading $(aE)^n$ discretisation corrections estimated to be 1.0 % for both $D\rightarrow \pi$ and $D\rightarrow K$	Relative scale $r_1/a$ set from the static quark potential. Absolute scale $r_1$ set from several quantities including $f_\pi$, $f_K$ and $\Upsilon \,2S$–$1S$ splitting cf. HPQCD 09B [186]. Scale uncertainty estimated to be 0.7 % in $D\rightarrow \pi$ and 0.2 % in $D\rightarrow K$
FNAL/MILC 04	[357]	$2+1$	0.12	Discretisation effects from light-quark sector estimated to be 4 % by power counting. Discretisation effects from final-state pion and kaon energies estimated to be 5 %	Scale set through $\Upsilon \,2S$–$1S$ splitting cf. HPQCD 03 [695]. Error in $a^{-1}$ estimated to be 1.2 %, but scale error in dimensionless form factor negligible compared to other uncertainties
ETM 11B	[345]	2	0.068, 0.086, 0.102	Discretisation errors estimated to be 5 % for $D\rightarrow \pi$ and 3 % for $D\rightarrow K$ from comparison of results in the continuum limit to those at the finest lattice spacing	Scale set through $f_\pi$ cf. ETM 07A [686] and ETM 09C [241]

**Table 82:** Chiral extrapolation/minimum pion mass in determinations of the $D\rightarrow \pi \ell \nu$ and $D\rightarrow K \ell \nu$ form factors. For actions with multiple species of pions, masses quoted are the RMS pion masses. The different $M_{\pi ,\mathrm min}$ entries correspond to the different lattice spacings

Collab.	Ref.	$N_\mathrm{f}$	$M_{\pi ,\mathrm min}\,(\mathrm{MeV})$	Description
HPQCD 13C	[348]	$2+1$	390, 390	Modified $z$-expansion fit combining the continuum and chiral extrapolations and the momentum-transfer dependence
HPQCD 10B, 11	[338, 342]	$2+1$	390, 390	Modified $z$-expansion fit combining the continuum and chiral extrapolations and the momentum-transfer dependence. Contributions to error budget from light valence and sea-quark mass dependence estimated to be 2.0 % for $D \rightarrow \pi$ and 1.0 % for $D \rightarrow K$
FNAL/MILC 04	[357]	$2+1$	510	Fit to S$\chi$PT, combined with the Becirevic–Kaidalov ansatz for the momentum transfer dependence of form factors. Error estimated to be 3 % for $D \rightarrow \pi$ and 2 % for $D \rightarrow K$ by comparing fits with and without one extra analytic term
ETM 11B	[345]	2	270	SU(2) tmHM$\chi$PT plus Becirevic–Kaidalov ansatz for fits to the momentum-transfer dependence of form factors. Fit uncertainty estimated to be 7 % for $D \rightarrow \pi$ and 5 % for $D \rightarrow K$ by considering fits with and without NNLO corrections of order ${\mathcal {O}}(m_\pi ^4)$ and/or higher-order terms through $E^5$, and by excluding data with $E \gtrsim 1$ GeV

**Table 83:** Finite-volume effects in determinations of the $D\rightarrow \pi \ell \nu$ and $D\rightarrow K\ell \nu$ form factors. Each $L$-entry corresponds to a different lattice spacing, with multiple spatial volumes at some lattice spacings. For actions with multiple species of pions, the lightest pion masses are quoted

Collab.	Ref.	$N_\mathrm{f}$	$L$ (fm)	${M_{\pi ,\mathrm min}}L$	Description
HPQCD 13C	[348]	$2+1$	2.4, 2.4/2.9	$\gtrsim 3.8$	No explicit estimate of FV error, but it is expected to be small for simulation masses and volumes
HPQCD 10B, 11	[338, 342]	$2+1$	2.4, 2.4/2.9	$\gtrsim 3.8$	Finite-volume effects estimated to be 0.04 % for $D\rightarrow \pi$ and 0.01 % for $D\rightarrow K$ by comparing the “$m_\pi ^2 \mathrm{log} (m_\pi ^2)$” term in infinite and finite volume
FNAL/MILC 04	[357]	$2+1$	2.4/2.9	$\gtrsim 3.8$	No explicit estimate of FV error, but it is expected to be small for simulation masses and volumes
ETM 11B	[345]	2	2.2, 2.1/2.8, 2.4	$\gtrsim 3.7$	Finite-volume uncertainty estimated to be at most 2 % by considering fits with and without the lightest pion mass point at $m_\pi L \approx 3.7$

**Table 84:** Operator renormalisation in determinations of the $D\rightarrow \pi \ell \nu$ and $D\rightarrow K \ell \nu$ form factors

Collab.	Ref.	$N_\mathrm{f}$	Ren.	Description
HPQCD 13C	[348]	$2+1$	–	Scalar form factor extracted from absolutely normalised scalar current. Vector form factor extracted from both point-split spatial (normalised by requiring $Zf_+(0) = 1$ for flavourless currents and checking mass independence) and local temporal vector currents (normalised by matching to the scalar case using kinematic constraint at zero momentum transfer $f_+(0) = f_0(0)$)
HPQCD 10B, 11	[338, 342]	$2+1$	–	Form factor extracted from absolutely normalised scalar-current matrix element then using kinematic constraint at zero momentum-transfer $f_+(0) = f_0(0)$
FNAL/MILC 04	[357]	$2+1$	mNPR	Size of two-loop correction to current renormalisation factor assumed to be negligible
ETM 11B	[345]	2	–	Form factors extracted from double ratios insensitive to current normalisation

**Table 85:** Heavy quark treatment in determinations of the $D\rightarrow \pi \ell \nu$ and $D\rightarrow K \ell \nu$ form factors

Collab.	Ref.	$N_\mathrm{f}$	Action	Description
HPQCD 13C	[348]	$2+1$	HISQ	Bare charm-quark mass $am_{c} \sim 0.41$–0.63. No explicit estimate of $(am_{c})^n$ errors
HPQCD 10B, 11	[338, 342]	$2+1$	HISQ	Bare charm-quark mass $am_{c} \sim 0.41$–0.63. Errors of $(am_{c})^n$ estimated within modified $z$-expansion to be 1.4 % for $D\rightarrow K$ and 2.0 % for $D \rightarrow \pi$. Consistent with expected size of dominant one-loop cutoff effects on the finest lattice spacing, ${\mathcal O}(\alpha _S (am_{c})^2 (v/c) )\sim 1.6$ %
FNAL/MILC 04	[357]	$2+1$	Fermilab	Discretisation errors from charm quark estimated via heavy-quark power-counting to be 7 %
ETM 11B	[345]	2	tmWil	Bare charm-quark mass $am_{c} \sim 0.17$–0.30. Expected size of ${\mathcal O}((am_{c})^2)$ cutoff effects on the finest lattice spacing consistent with quoted 5 % continuum-extrapolation uncertainty

### B.6 Notes to Sect. 8 on $B$-meson decay constants, mixing parameters, and form factors

In the following, we summarise the characteristics (lattice actions, pion masses, lattice spacings, etc.) of the recent $N_\mathrm{f}= 2+1$ and $N_\mathrm{f}= 2$ runs. We also provide brief descriptions of how systematic errors are estimated by the various authors. We focus on calculations with either preliminary or published quantitative results.

#### B.6.1 $B_{(s)}$-meson decay constants

See Tables 86, 87, 88, 89, 90, 91, 92, and 93.

**Table 86:** Continuum extrapolations/estimation of lattice artefacts in determinations of the $B$- and $B_{s}$-meson decay constants for $N_\mathrm{f}=2$ simulations

Collab.	Ref.	$N_\mathrm{f}$	$a$ (fm)	Continuum extrapolation	Scale setting
ALPHA 13 ALPHA 12A ALPHA 11	[365, 370, 404]	2	0.075, 0.065, 0.048	Combined continuum and chiral extrapolation with linear in $a^2$ term. Continuum extrapolation errors estimated to be 5 MeV in ALPHA 11	Relative scale set from $r_0$. Absolute scale set from $f_K$. Scale-setting uncertainty included in combined statistical and extrapolation error
ETM 13B, 13C ETM 12B ETM 11A	[335, 336, 393, 405]	2	0.098, 0.085, 0.067, 0.054	Combined continuum and chiral extrapolation, with a term linear in $a^2$. ETM 12 and 13 include a heavier masses than ETM 11A. Discretisation error included in combined statistical and systematic error, estimated by dropping the data at the coarsest lattice spacing as $\sim$0.5–1 %	Scale set from $f_\pi$. Scale-setting uncertainty included in combined statistical and systematic error
ETM 09D	[392]	2	0.098, 0.085, 0.067	Combined continuum and chiral extrapolation with a term linear in $a^2$	Scale set from $f_\pi$. Scale-setting uncertainty included in combined statistical and systematic error

**Table 87:** Continuum extrapolations/estimation of lattice artefacts in determinations of the $B$- and $B_{s}$-meson decay constants for $N_\mathrm{f}=2+1+1$ and $N_\mathrm{f}=2+1$ simulations

Collab.	Ref.	$N_\mathrm{f}$	$a$ (fm)	Continuum extrapolation	Scale setting
ETM 13E	[399]	$2+1+1$	0.89, 0.82, 0.62	Combined continuum and chiral extrapolation, linear in $a^2$	Scale set from $f_\pi$. Scale-setting uncertainty included in combined statistical and systematic error
HPQCD 13	[400]	$2+1+1$	0.15, 0.12, 0.09	Combined continuum and chiral extrapolation. Continuum extrapolation errors estimated to be 0.7 %	Scale set from $\Upsilon$($2S$–$1S$) splitting; see Ref. [383]. Scale uncertainty included in statistical error
RBC/UKQCD 13A	[401]	$2+1$	0.11, 0.086	Combined continuum and chiral extrapolation with linear in $a^2$ term. No systematic error estimate	Scale set by the $\Omega$ baryon mass
HPQCD 12	[402]	$2+1$	0.12, 0.09	Combined continuum and chiral extrapolation. Continuum extrapolation errors estimated to be 0.9 %	Relative scale $r_1/a$ from the static quark potential. Absolute scale $r_1$ from $f_\pi$, $f_K$, and $\Upsilon$($2S$–$1S$) splitting. Scale uncertainty estimated to be 1.1 %
HPQCD 11A	[366]	$2+1$	0.15, 0.12, 0.09, 0.06, 0.045	$am_Q \approx 0.2$–0.85. Combined continuum and HQET fit. Continuum extrapolation error estimated by varying the fit ansatz and the included data points to be 0.63 %. Discretisation errors appear to decrease with increasing heavy-meson mass	Relative scale $r_1/a$ from the static quark potential. Absolute scale $r_1$ from $f_\pi$, $f_K$ and $\Upsilon$($2S$–$1S$) splitting. Scale uncertainty estimated to be 0.74 %
FNAL/MILC 11	[332]	$2+1$	0.15, 0.12, 0.09	Combined continuum and chiral extrapolation. Continuum-extrapolation errors estimated to be 1.3 %	Relative scale $r_1/a$ from the static quark potential. Absolute scale $r_1$ from $f_\pi$, $f_K$, and $\Upsilon$($2S$–$1S$) splitting. Scale uncertainty estimated to be 1 MeV
RBC/UKQCD 10C	[406]	$2+1$	0.11	One lattice spacing with discretisation errors estimated by power counting as 3 %	Scale set by the $\Omega$ baryon mass. Combined scale and mass tuning uncertainties on $f_{B_{s}}/f_B$ estimated as 1 %
HPQCD 09	[403]	$2+1$	0.12, 0.09	Combined continuum and chiral extrapolation. Continuum extrapolation errors estimated to be 3 %	Relative scale $r_1/a$ from the static quark potential. Absolute scale $r_1$ from the $\Upsilon$($2S$–$1S$) splitting. Scale uncertainty estimated to be 2.3 %

**Table 88:** Chiral extrapolation/minimum pion mass in determinations of the $B$- and $B_{s}$-meson decay constants for $N_\mathrm{f}=2$ simulations. For actions with multiple species of pions, masses quoted are the RMS pion masses. The different $M_{\pi ,\mathrm min}$ entries correspond to the different lattice spacings

Collab.	Ref.	$N_\mathrm{f}$	$M_{\pi ,\mathrm min}\,(\mathrm{MeV})$	Description
ETM 13B, 13C ETM 12B ETM 11A	[335, 336, 393, 405]	2	410, 275, 300, 270	$M_{\pi ,\mathrm{min}}$ refers to the charged pions. Linear and NLO (full QCD) HM$\chi$PT supplemented by an $a^2$ term is used. The chiral fit error is estimated from the difference between the NLO HM$\chi$PT and linear fits with half the difference used as estimate of the systematic error. For the static limit calculation in ETM 11A, $\Phi _{s}^\mathrm{stat}$ is extrapolated assuming a constant in light quark mass. The ratio $\Phi _{s}^\mathrm{stat}/\Phi _{\ell }^\mathrm{stat}$ is fit using three different chiral fit forms (NLO HM$\chi$PT, linear, and quadratic) to estimate the chiral fir error
ETM 09D	[392]	2	410, 275, 300	$M_{\pi ,\mathrm{min}}$ refers to the charged pions. Linear and NLO (full QCD) HM$\chi$PT is used. The final result given by the average of NLO HMChiPT and linear *Ansätze* $\pm$ half the difference)
ALPHA 13 ALPHA 12A	[370, 404]	2	270, 190, 270	LO and NLO HMChPT supplemented by a term linear in $a^2$ are used. The final result is an average between LO and NLO with half the difference used as estimate of the systematic error
ALPHA 11	[365]	2	331, 268, 267	Linear and NLO (full QCD) HMChPT supplemented by a term linear in $a^2$ are used. The final result is an average between linear and NLO fits with half the difference used as estimate of the systematic error

**Table 89:** Chiral extrapolation/minimum pion mass in determinations of the $B$- and $B_{s}$-meson decay constants for $N_\mathrm{f}=2+1+1$ and $N_\mathrm{f}=2+1$ simulations. For actions with multiple species of pions, masses quoted are the RMS pion masses. The different $M_{\pi ,\mathrm{min}}$ entries correspond to the different lattice spacings

Collab.	Ref.	$N_\mathrm{f}$	$M_{\pi ,\mathrm min}\,(\mathrm{MeV})$	Description
ETM 13E	[399]	$2+1+1$	245, 239, 211	$M_{\pi ,\mathrm{min}}$ refers to the charged pions. Linear and NLO (full QCD) HM$\chi$PT supplemented by an $a^2$ term is used for the SU(3)-breaking ratios.The chiral fit error is estimated from the difference between the NLO HM$\chi$PT and linear fits with half the difference used as estimate of the systematic error. The ratio $z_{s}$ is fit using linear light quark mass dependence supplemented by an $a^2$ term
HPQCD 13	[400]	$2+1+1$	310, 294, 173	Two or three pion masses at each lattice spacing, one each with a physical-mass GB pion. NLO (full QCD) HM$\chi$PT supplemented by generic $a^2$ and $a^4$ terms is used to interpolate to the physical pion mass
RBC/UKQCD 13A	[401]	$2+1$	329, 289	Three (two) light quark masses per lattice spacing. NLO SU(2) HM$\chi$PT is used. No systematic error estimate
HPQCD 12	[402]	$2+1$	390, 390	Two or three pion masses at each lattice spacing. NLO (full QCD) HM$\chi$PT supplemented by NNLO analytic terms and generic $a^2$ and $a^4$ terms is used. The systematic error is estimated by varying the fit Ansatz, in particular for the NNLO analytic terms and the $a^{2n}$ terms
HPQCD 11A	[366]	$2+1$	570, 450, 390, 330, 330	One light sea quark mass only at each lattice spacing. The sea-quark mass dependence is assumed to be negligible, based on the calculation of $f_{D_{s}}$ in Ref. [94], where the sea quark extrapolation error is estimated as 0.34 %
FNAL/MILC 11	[332]	$2+1$	570, 440, 320	Three to five sea-quark masses per lattice spacing, and 9–12 valence light quark masses per ensemble. NLO partially quenched HMrS$\chi$PT including $1/m$ terms and supplemented by NNLO analytic and $\alpha _\mathrm{s}^2a^2$ terms is used. The systematic error is estimated by varying the fit Ansatz, in particular the NNLO analytic terms and the chiral scale
RBC/UKQCD 10C	[406]	$2+1$	430	Three light quark masses at one lattice spacing. NLO SU(2) $\chi$PT is used. The systematic error is estimated from the difference between NLO $\chi$PT and linear fits as $\sim$7 %
HPQCD 09	[403]	$2+1$	440, 400	Four or two pion masses per lattice spacing. NLO (full QCD) HMrS$\chi$PT supplemented by NNLO analytic terms and $\alpha _\mathrm{s} a^2, a^4$ terms is used. The chiral fit error is estimated by varying the fit Ansatz, in particular, by adding or removing NNLO and discretisation terms

**Table 90:** Finite-volume effects in determinations of the $B$- and $B_{s}$-meson decay constants. Each $L$-entry corresponds to a different lattice spacing, with multiple spatial volumes at some lattice spacings

Collab.	Ref.	$N_\mathrm{f}$	$L$ (fm)	${M_{\pi ,\mathrm min}}L$	Description
ETM 13E	[399]	$2+1+1$	2.84/2.13, 2.61/1.96, 2.97	3.53, 3.16, 3.19	FV error estimated how?
HPQCD 13	[400]	$2+1+1$	2.4/3.5/4.7, 2.9/3.8/5.8, 2.8/5.6	3.30, 3.88, 3.66	The analysis uses finite-volume $\chi$PT
RBC/UKQCD 13A	[401]	$2+1$	2.6, 2.75	4.54, 4.05	No FV error estimate
HPQCD 12	[402]	$2+1$	2.4/2.9, 2.5/3.6	3.84, 4.21	FV error is taken from Ref. [165] for HPQCD’s $D$-meson analysis, where it was estimated using finite volume $\chi$PT
HPQCD 11A	[366]	$2+1$	2.4, 2.4, 2.5, 2.9, 2.9	3.93, 4.48, 4.14, 4.49, 4.54	FV error is assumed to negligible
FNAL/MILC 11	[332]	$2+1$	2.4, 2.4/2.9, 2.5/3.6	3.93, 3.78, 4.14	FV error is estimated using finite-volume $\chi$PT
RBC/UKQCD 10C	[406]	$2+1$	1.8	$3.9$	FV error estimated using finite-volume $\chi$PT to be 1 % for SU(3) breaking ratios
HPQCD 09	[403]	$2+1$	2.4/2.9, 2.5	3.78, 4.14	FV error is assumed to negligible
ETM 13B, 13C ETM 12B ETM 11A	[335, 405] [393] [336]	2	2.4, 2.0/2.7, 2.1, 1.7/2.6	5, 3.7, 3.3, 3.5	FV errors are found to be negligible by comparing results at $m_\pi L = 3.3$ and $m_\pi L=4.3$ for $m_\pi \simeq 310$ MeV
ALPHA 13 ALPHA 12A ALPHA 11	[404] [370] [365]	2	2.4, 2.1/4.2 [3.1], 2.3/3.1	5.2, 4.1, 4.2	No explicit estimate of FV errors, but it is expected to be much smaller than other uncertainties

**Table 91:** Description of the renormalisation/matching procedure adopted in the determinations of the $B$- and $B_{s}$-meson decay constants for $N_\mathrm{f}=2+1$ simulations

Collab.	Ref.	$N_\mathrm{f}$	Ren.	Description
ETM 13E	[399]	$2+1+1$	–, PT1$\ell$	The current used for the relativistic decay constants is absolutely normalised. The ratio is constructed from the relativistic decay constant data and the heavy-quark pole masses. Ratios of pole-to-${\overline{\mathrm{MS}}}$ mass conversion factors are included at NLO in continuum perturbation theory
HPQCD 13	[400]	$2+1+1$	PT1$\ell$	The NRQD effective current is matched through $O(1/m$) and renormalised using one-loop PT. Included are all terms though $O(\alpha _\mathrm{s})$, $O(\alpha _\mathrm{s} \, a)$, $O(\Lambda _\mathrm{QCD}/M)$, $O(\alpha _\mathrm{s}/a M)$, $O(\alpha _\mathrm{s} \, \Lambda _\mathrm{QCD}/M)$. The dominant error is due unknown $O(\alpha _\mathrm{s}^2)$ contributions to the current renormalisation. The perturbation theory used in this work is the same as in HPQCD 09 and 12, but it is rearranged to match the mNPR method. Using the fact that the heavy–heavy temporal vector current is normalised, and that the light–light HISQ vector current receives a small one-loop correction, the error is estimated as $\sim$1.4 %
RBC/UKQCD 13A	[401]	$2+1$	mNPR	No systematic error estimate
HPQCD 12/09	[402, 403]	$2+1$	PT1$\ell$	The NRQD effective current is matched through $O(1/m$) and renormalised using one-loop PT. Included are all terms though $O(\alpha _\mathrm{s})$, $O(\alpha _\mathrm{s} \, a)$, $O(\Lambda _\mathrm{QCD}/M)$, $O(\alpha _\mathrm{s}/a M)$, $O(\alpha _\mathrm{s} \, \Lambda _\mathrm{QCD}/M)$. The dominant error is due unknown $O(\alpha _\mathrm{s}^2)$ contributions to the current renormalisation. The authors take the perturbative error as $\sim 2 \rho _0 \, \alpha _\mathrm{s}^2$, where $\rho _0$ is the coefficient of the one-loop correction to the leading term, which yields an error of $\sim$4 %
HPQCD 11A	[366]	$2+1$	–	This work uses PCAC together with an absolutely normalised current
FNAL/MILC 11	[332]	$2+1$	mNPR	The authors’ estimate of the perturbative errors is comparable in size to the actual one-loop corrections
RBC/UKQCD 10C	[406]	$2+1$	PT1$\ell$	The static-light current is matched through $O(\alpha _\mathrm{s} a, \alpha _\mathrm{s})$ and renormalised using one-loop tadpole-improved PT. For massless light quarks, the renormalisation factors cancel in the ratio of decay constants

**Table 92:** Description of the renormalisation/matching procedure adopted in the determinations of the $B$- and $B_{s}$-meson decay constants for $N_\mathrm{f}=2$ simulations

Collab.	Ref.	$N_\mathrm{f}$	Ren.	Description
ALPHA 13	[404]	2	NPR	The authors use the Schr${\ddot{\mathrm{d}}}$ingier functional for the NP matching
ALPHA 12A	[370]			
ALPHA 11	[365]			
ETM 13B, 13C ETM 12B ETM 11A	[335, 405] [393] [336]	2	–, PT1$\ell$	The current used for the relativistic decay constants is absolutely normalised. **Interpolation method:** The static limit current renormalisation is calculated in one-loop mean-field improved perturbation theory, there half the correction is used to estimate the error. **Ratio method:** The ratio is constructed from the relativistic decay constant data and the heavy-quark pole masses. Ratios of pole-to-${\overline{\mathrm{MS}}}$ mass conversion factors are included at NLO in continuum perturbation theory

**Table 93:** Heavy quark treatment in determinations of the $B$- and $B_{s}$-meson decay constants

Collab.	Ref.	$N_\mathrm{f}$	Action	Description
ETM 13E	[399]	$2+1+1$	tmWil	The estimate of the discretisation effects is described in the continuum table. The relativistic data are matched to HQET using NLO continuum PT in an intermediate step, and converted back to QCD at the end. The error due to HQET matching (estimated by replacing the NLO expressions with LO) is a very small contribution to the systematic error due to the heavy quark mass dependence
HPQCD 13	[400]	$2+1+1$	NRQCD	HQ truncation effects estimated as in HPQCD 09 to be 1.0 %
RBC/UKQCD 13A	[401]	$2+1$	RHQ	No estimate of HQ discretisation errors
HPQCD 12	[402]	$2+1$	NRQCD	HQ truncation effects estimated as in HPQCD 09 to be 1.0 %
HPQCD 11A	[366]	$2+1$	HISQ	The analysis uses a combined continuum and $1/m$ extrapolation
FNAL/MILC 11	[332]	$2+1$	Fermilab	HQ discretisation effects are included in the combined chiral and continuum fits, and they are estimated by varying the fit Ansatz and excluding the data at the coarsest lattice spacing to be $\sim$2 %, consistent with simple power counting estimates but larger than the residual discretisation errors observed in the data
RBC/UKQCD 10C	[406]	$2+1$	Static	Truncation effects of ${\mathcal {O}}(1/{m_{h}})$ on the SU(3)-breaking ratios are estimated by power counting to be 2 %
HPQCD 09	[403]	$2+1$	NRQCD	The leading HQ truncation effects are of ${\mathcal {O}}({\alpha _\mathrm{s}} {\Lambda _\mathrm{QCD}}/{m_{h}})$ due to the tree-level coefficient of the ${\varvec{\sigma }}\cdot {\mathbf {B}}$ term. The error is estimated by calculating the ${B^{*}}$–$B$ hyperfine splitting and comparing with experiment as 1 %
ALPHA 13 ALPHA 12A	[404] [370]	2	HQET	NP improved through ${\mathcal {O}}(1/{m_{h}})$. Truncation errors of ${\mathcal {O}}({\Lambda _\mathrm{QCD}}/{{m_{h}})^{2}}$ are not included
ALPHA 11	[365]			
ETM 13B, 13C ETM 12B ETM 11A	[335, 405] [393] [336]	2	tmWil	The estimate of the discretisation effects is described in the continuum table. In both methods the relativistic data are matched to HQET using NLO continuum PT in an intermediate step, and converted back to QCD at the end. The error due to HQET matching (estimated by replacing the NLO expressions with LO) is a very small contribution to the systematic error due to the heavy quark mass dependence. The variation observed from adding heavier masses to their data and/or including $1/{m_{h}^{3}}$ terms is 0.4–1.3 %

#### B.6.2 $B_{(s)}$-meson mixing matrix elements

See Tables 94, 95, 96, 97, 98, 99.

**Table 94:** Continuum extrapolations/estimation of lattice artefacts in determinations of the neutral $B$-meson mixing matrix elements for $N_\mathrm{f}=2+1$ simulations

Collab.	Ref.	$N_\mathrm{f}$	$a$ (fm)	Continuum extrapolation	Scale setting
FNAL/MILC 12	[415]	$2+1$	0.12, 0.09	Combined continuum and chiral extrapolation with NLO rHMS$\chi$PT, NNLO analytic and generic $\mathcal{O}(\alpha _\mathrm{s}^2 a^2, a^4)$ terms. Combined statistical, chiral and light-quark discretisation error is estimated, by examining the variation with different fit Ansätze to be 3.7 % on $\xi$	Relative scale $r_1/a$ is set via static quark potential. Absolute scale $r_1=0.3117(22)$ fm is determined [332] through averaging the $f_\pi$ input and the estimate of HPQCD collaboration [186]. The scale uncertainty on $\xi$ is estimated as 0.2 %
FNAL/MILC 11A	[412]	$2+1$	0.12, 0.09, 0.06	Combined continuum and chiral extrapolation with NLO rHMS$\chi$PT, NNLO analytic and generic $\mathcal{O}(\alpha _\mathrm{s}^2 a^2, a^4)$ terms	See above. The error in $r_1$ yields a 3 % uncertainty on $f^2_B B_B$
RBC/UKQCD 10C	[406]	$2+1$	0.11	Only one lattice spacing is used. Discretisation error is estimated to be 4 % on $\xi$ by power counting	Scale is set using the $\Omega ^-$ mass as input [79]. The error on $\xi$ due to the combined scale and light quark mass uncertainties is estimated as 1 %
HPQCD 09	[403]	$2+1$	0.12, 0.09	Combined continuum and chiral extrapolation with NLO rHMS$\chi$PT and NNLO analytic terms. Light-quark discretisation error is estimated as 3, 2 and 0.3 % for $f_B\sqrt{B_B}$, $f_{B_{s}}\sqrt{B_{B_{s}}}$ and $\xi$, respectively	Relative scale $r_1/a$ is set via static quark potential. Absolute scale $r_1=0.321(5)$ fm is determined through $\Upsilon$ mass [380]. The error on $f_B\sqrt{B_B}$ due to the scale uncertainty is estimated as 2.3 %
HPQCD 06A	[413]	$2+1$	0.12	Only one lattice spacing is used. Light-quark discretisation error on $f_{B_{s}}^2B_{B_{s}}$ is estimated as 4 % by power counting	Scale is set using the $\Upsilon \,2S$–$1S$ splitting as input [380]. The error on $f^2_B B_B$ due to the scale uncertainty is estimated as 5 %

**Table 95:** Continuum extrapolations/estimation of lattice artefacts in determinations of the neutral $B$-meson mixing matrix elements for $N_\mathrm{f}=2$ simulations

Collab.	Ref.	$N_\mathrm{f}$	$a$ (fm)	Continuum extrapolation	Scale setting
ETM 13B	[335]	2	0.098, 0.085, 0.067, 0.054	Combined chiral and continuum extrapolation, with a term linear in $a^2$. Discretisation error is estimated by omitting the coarsest lattice as 0.5, 1.7, 1.3 and 1.0 % for $B_{B_{s}}$, $B_B$, $B_{B_{s}}/B_B$ and $\xi$, respectively. The heavy-quark masses vary in the range $0.13 \lesssim a m_{h} \lesssim 0.85$	See below
ETM 12A,12B	[393, 414]	2	0.098, 0.085, 0.067	Combined chiral and continuum extrapolation, with a term linear in $a^2$. Discretisation error included in combined statistical, chiral and continuum-extrapolation error and estimated as 4.5 %. The heavy-quark masses vary in the range $0.25 \lesssim a m_{h} \lesssim 0.6$	Relative scale $r_0/a$ set from the static quark potential. Absolute scale set from $f_\pi$. Scale-setting uncertainty included in combined statistical and systematic error

**Table 96:** Chiral extrapolation/minimum pion mass in determinations of the neutral $B$-meson mixing matrix elements. For actions with multiple species of pions, masses quoted are the RMS pion masses. The different $M_{\pi ,\mathrm min}$ entries correspond to the different lattice spacings

Collab.	Ref.	$N_\mathrm{f}$	$M_{\pi ,\mathrm{min}}\,(\mathrm{MeV})$	Description
FNAL/MILC 12	[415]	$2+1$	440, 320	Combined continuum and chiral extrapolation with NLO rHMS$\chi$PT and NNLO analytic terms. See the entry in Table 94. The omission of wrong-spin contributions [416] in the HMrS$\chi$PT is treated as a systematic error and estimated to be 3.2 % for $\xi$
FNAL/MILC 11A	[412]	$2+1$	440, 320, 250	Combined continuum and chiral extrapolation with NLO rHMS$\chi$PT and NNLO analytic terms
RBC/UKQCD 10C	[406]	$2+1$	430	Linear fit matched with SU(2) NLO HM$\chi$PT at the lightest $ud$ mass point is used as the preferred fit. Many different fit Ansätze are considered. The systematic error is estimated from the difference between the SU(2) HM$\chi$PT fit described above and a linear fit
HPQCD 09	[403]	$2+1$	440, 400	Combined continuum and chiral extrapolation with NLO rHMS$\chi$PT and NNLO analytic terms
HPQCD 06A	[413]	$2+1$	510	Two sea $ud$ quark masses $m_{ud}/m_{s}=0.25$ and $0.5$ are used to calculate the matrix element for $B_{s}$-meson at the predetermined value of the strange quark mass. No significant sea quark mass dependence is observed and the value at the lighter sea $ud$ mass is taken as the result
ETM 13B ETM 12A,12B	[335] [393, 414]	2	410, 275, 300, 270	$M_{\pi ,\mathrm{min}}$ refers to the charged pions, where 270 MeV on the finest lattice only included in ETM 13. Linear and NLO (full QCD) HM$\chi$PT supplemented by an $a^2$ term is used. The chiral fit error is estimated from the difference between the NLO HM$\chi$PT and linear fits

**Table 97:** Finite-volume effects in determinations of the neutral $B$-meson mixing matrix elements. Each $L$-entry corresponds to a different lattice spacing, with multiple spatial volumes at some lattice spacings. For actions with multiple species of pions, the lightest masses are quoted

Collab.	Ref.	$N_\mathrm{f}$	$L$ (fm)	${M_{\pi ,\mathrm min}}L$	Description
FNAL/MILC 12	[415]	$2+1$	2.4/2.9, 2.5	$\gtrsim$3.8	FV error is estimated to be less than 0.1 % for SU(3)-breaking ratios from FV HMrS$\chi$PT
FNAL/MILC 11A	[412]	$2+1$	2.4/2.9, 2.5/2.9/3.6, 3.8	$\gtrsim$3.8	FV error on $f_B\sqrt{B_B}$ is estimated to be less than 1 %, which is inferred from the study of the $B$-meson decay constant using FV HM$\chi$PT [332]
RBC/UKQCD 10C	[406]	$2+1$	1.8	$\gtrsim$3.9	FV error estimated through FV HM$\chi$PT as 1 % for SU(3) breaking ratios
HPQCD 09	[403]	$2+1$	2.4/2.9, 2.5	$\gtrsim$3.8	No explicit estimate of FV error, but it is expected to be much smaller than other uncertainties
HPQCD 06A	[413]	$2+1$	2.4	$\gtrsim$4.5	No explicit estimate of FV error, but it is expected to be much smaller than other uncertainties
ETM 13B ETM 12A,12B	[335] [393, 414]	2	2.4, 2.0/2.7, 2.1, 1.7/2.6	$\gtrsim$3.2	$L=1.7/2.6\, \mathrm{fm}$ only included in ETM 13. FV error is assumed to be negligible based on the study of $D$-meson decay constants in Ref. [169]

**Table 98:** Operator renormalisation in determinations of the neutral $B$-meson mixing matrix elements

Collab.	Ref.	$N_\mathrm{f}$	Ren.	Description
FNAL/MILC 12	[415]	$2+1$	PT$1l$	One-loop mean-field improved PT is used to renormalise the four-quark operators with heavy quarks rotated to eliminate tree-level $O(a)$ errors. The error from neglecting higher-order corrections is estimated to be 0.5 % on $\xi$
FNAL/MILC 11A	[412]	$2+1$	PT$1l$	One-loop mean-field improved PT is used to renormalise the four-quark operators with heavy quarks rotated to eliminate tree-level $O(a)$ errors. The error from neglected higher-order corrections is estimated to be 4 % on $f_B\sqrt{B_B}$
RBC/UKQCD 10C	[406]	$2+1$	PT$1l$	Static-light four-quark operators are renormalised with one-loop mean-field improved PT. The error due to neglected higher-order effects is estimated to be 2.2 % on $\xi$
HPQCD 09	[403]	$2+1$	PT$1l$	Four-quark operators in lattice NRQCD are matched to QCD through order $\alpha _\mathrm{s}$, $\Lambda _\mathrm{QCD}/M$ and $\alpha _\mathrm{s}/(aM)$ [696] using one-loop PT. The error due to neglected higher-order effects is estimated to be 4 % on $f_B\sqrt{B_B}$ and 0.7 % on $\xi$
HPQCD 06A	[413]	$2+1$	PT$1l$	Four-quark operators in lattice NRQCD are matched to full QCD through order $\alpha _\mathrm{s}$, $\Lambda _\mathrm{QCD}/M$ and $\alpha _\mathrm{s}/(aM)$ [696]. The error is estimated as $\sim 1\cdot \alpha _\mathrm{s}^2$ to be 9 % on $f_{B_{s}}^2 B_{B_{s}}$
ETM 13B, 12A, 12B	[335, 393, 414]	2	NPR	The bag parameters are non-perturbatively renormalised in the RI’-MOM scheme. They are calculated as functions of the (${\overline{\mathrm{MS}}}$) heavy-quark mass (renormalised non-perturbatively in RI/MOM)

**Table 99:** Heavy-quark treatment in determinations of the neutral $B$-meson mixing matrix elements

Collab.	Ref.	$N_\mathrm{f}$	Action	Description
FNAL/MILC 12	[415]	$2+1$	Fermilab	The heavy-quark discretisation error on $\xi$ is estimated to be 0.3 %. The error on $\xi$ due to the uncertainty in the $b$-quark mass is are estimated to be 0.4 %
FNAL/MILC 11A	[412]	$2+1$	Fermilab	The heavy-quark discretisation error on $f_B\sqrt{B_B}$ is estimated as 4 % using power-counting
RBC/UKQCD 10C	[406]	$2+1$	Static	Two different static quark actions with Ape and HYP smearings are used. The discretisation error on $\xi$ is estimated as $\sim$4 % and the error due to the missing $1/m_{b}$ corrections as $\sim$2 %, both using power-counting
HPQCD 09	[403]	$2+1$	NRQCD	Heavy-quark truncation errors due to relativistic corrections are estimated to be 2.5, 2.5 and 0.4 % for $f_B\sqrt{B_B}$, $f_{B_{s}}\sqrt{B_{B_{s}}}$ and $\xi$, respectively
HPQCD 06A	[413]	$2+1$	NRQCD	Heavy-quark truncation errors due to relativistic corrections are estimated to be 3 % for $f_{B_{s}}^2{B_{B_{s}}}$
ETM 13B ETM 12A,12B	[335] [393, 414]	2	tmWilson	The ratio method is used to perform an interpolation to the physical $b$ quark mass from the simulated heavy mass and the known static limit. In an intermediate step, the ratios include HQET matching factors calculated to tree level, leading-log, and next-to-leading-log (ETM 13 only) in continuum PT. The interpolation uses a polynomial up to quadratic in the inverse quark-mass. The systematic errors added together with those of the chiral fit are estimated as 1.3–1.6 % for bag parameters for ETM 13, while they are estimated from changing the interpolating polynomial as 2 % and from changing the order of HQET matching factors as 3 % for ETM 12A and 12B

#### B.6.3 $B\rightarrow \pi \ell \nu$ form factor

See Tables 100, 101, 102, 103, and 104.

**Table 100:** Continuum extrapolations/estimation of lattice artefacts in determinations of the $B\rightarrow \pi \ell \nu$ form factor

Collab.	Ref.	$N_\mathrm{f}$	$a$ (fm)	Continuum extrapolation	Scale setting
FNAL/MILC 08A	[351]	$2+1$	0.09, 0.12	Fit to rHMS$\chi$PT to remove light-quark discretisation errors. Residual heavy-quark discretisation errors estimated with power-counting to be 3.4 %	Relative scale $r_1/a$ set from the static quark potential. Absolute scale $r_1$ set through $f_\pi$ cf. MILC 07 [687]; error in scale taken to be difference from scale set through $\Upsilon \,2S$–$1S$ splitting cf. HPQCD 05B [380]. Scale uncertainty estimated at between 1 and 1.5 % in the range of $q^2$ explored
HPQCD 06	[427]	$2+1$	0.09, 0.12	Central values obtained from data at $a=0.12$ fm. Discretisation errors observed to be within the statistical error by comparison with data at $a=0.09$ fm	Relative scale $r_1/a$ set from the static quark potential. Absolute scale $r_1$ set through $\Upsilon \,2S$–$1S$ splitting cf. HPQCD 05B [380]

**Table 101:** Chiral extrapolation/minimum pion mass in determinations of the $B\rightarrow \pi \ell \nu$ form factor. For actions with multiple species of pions, masses quoted are the RMS pion masses. The different $M_{\pi ,\mathrm min}$ entries correspond to the different lattice spacings

Collab.	Ref.	$N_\mathrm{f}$	$M_{\pi ,\mathrm min}\,(\mathrm{MeV})$	Description
FNAL/MILC 08A	[351]	$2+1$	400, 440	Simultaneous chiral-continuum extrapolation and $q^2$ interpolation using SU(3) rHMS$\chi$PT. Systematic error estimated by adding higher-order analytic terms and varying the $B^*$–$B$–$\pi$ coupling
HPQCD 06	[427]	$2+1$	400, 440	First interpolate data at fixed quark mass to fiducial values of $E_\pi$ using the Becirevic–Kaidalov and Ball–Zwicky ansätze, then extrapolate data at fixed $E_\pi$ to physical quark masses using SU(3) rHMS$\chi$PT. Systematic error estimated by varying interpolation and extrapolation fit functions

**Table 102:** Finite-volume effects in determinations of the $B\rightarrow \pi \ell \nu$ form factor. Each $L$-entry corresponds to a different lattice spacing, with multiple spatial volumes at some lattice spacings. For actions with multiple species of pions, the lightest masses are quoted

Collab.	Ref.	$N_\mathrm{f}$	$L$ (fm)	${M_{\pi ,\mathrm min}}L$	Description
FNAL/MILC 08A	[351]	$2+1$	2.4, 2.4/2.9	$\gtrsim 3.8$	Estimate FV error to be 0.5 % using one-loop rHMS$\chi$PT
HPQCD 06	[427]	$2+1$	2.4/2.9	$\gtrsim 3.8$	No explicit estimate of FV error, but it is expected to be much smaller than other uncertainties

**Table 103:** Operator renormalisation in determinations of the $B\rightarrow \pi \ell \nu$ form factor

Collab.	Ref.	$N_\mathrm{f}$	Ren.	Description
FNAL/MILC 08A	[351]	$2+1$	mNPR	Perturbative truncation error estimated at 3 % with size of one-loop correction on finer ensemble
HPQCD 06	[427]	$2+1$	PT1$\ell$	Currents included through ${\mathcal O}(\alpha _S \Lambda _\mathrm{QCD}/M$, $\alpha _S/(aM)$, $\alpha _S\, a \Lambda _\mathrm{QCD})$. Perturbative truncation error estimated from power-counting

**Table 104:** Heavy quark treatment in determinations of the $B\rightarrow \pi \ell \nu$ form factor

Collab.	Ref.	$N_\mathrm{f}$	Action	Description
FNAL/MILC 08A	[351]	$2+1$	Fermilab	Discretisation errors in $f+(q^2)$ from heavy-quark action estimated to be 3.4 % by heavy-quark power-counting
HPQCD 06	[427]	$2+1$	NRQCD	Discretisation errors in $f_+(q^2)$ estimated to be ${\mathcal O} (\alpha _\mathrm{s} (a \Lambda _\mathrm{QCD})^2) \sim 3$ %. Relativistic errors estimated to be ${\mathcal O}((\Lambda _\mathrm{QCD}/M)^2) \sim 1\,\%$

#### B.6.4 $B\rightarrow D \ell \nu$ and $B \rightarrow D^* \ell \nu$ form factors and $R(D)$

See Tables 105, 106, 107, 108, 109, and 110.

**Table 105:** Continuum extrapolations/estimation of lattice artefacts in determinations of the $B\rightarrow D \ell \nu$, $B\rightarrow D^* \ell \nu$, $B_{s}\rightarrow D_{s} \ell \nu$ form factors and of $R(D)$

Collab.	Ref.	$N_\mathrm{f}$	$a$ (fm)	Continuum extrapolation	Scale setting
FNAL/MILC 13B $B \rightarrow D^*$	[447]	$2+1$	0.045, 0.06, 0.09, 0.12, 0.15	See FNAL/MILC 10	See below
FNAL/MILC 13B $B \rightarrow D$	[447]	$2+1$	0.045, 0.06, 0.09, 0.12	Continuum extrapolation using rHMS$\chi$PT to remove light-quark discretisation errors. Residual discretisation errors estimated from power-counting to be 2 % in $h_+$ (the dominant contributor to $f_+$) and 10 % in $h_-$	Relative scale $r_1/a$ set from the static quark potential. Absolute scale $r_1$ set through combination of $f_\pi$ from MILC 09B [697] and several quantities including $\Upsilon$ splittings from HPQCD [186], as described in [332]
FNAL/MILC 12A	[453]	$2+1$	0.09, 0.12	Continuum extrapolation using rHMS$\chi$PT to remove light-quark discretisation errors. Residual discretisation errors estimated to be very small for the ratio of branching fractions $R(D)$ at 0.2 %	See below
FNAL/MILC 10	[444]	$2+1$	0.06, 0.09, 0.12, 0.15	Continuum extrapolation using rHMS$\chi$PT to remove light-quark discretisation errors. Residual discretisation errors estimated to be 1.0 % from power-counting. Further, the data display no observable trend with lattice spacing	Relative scale $r_1/a$ set from the static quark potential. Absolute scale $r_1$ set through $f_\pi$ cf. MILC 09B [697]. Comparison with $r_1$ set via other quantities by HPQCD [186] shows negligible change
FNAL/MILC 08	[445]	$2+1$	0.09, 0.12, 0.15	Continuum extrapolation using rHMS$\chi$PT to remove light-quark discretisation errors. Residual discretisation errors estimated to be 1.5 % from power-counting and by comparison of data at 0.12 and 0.09 fm	Relative scale $r_1/a$ set from the static quark potential. Absolute scale $r_1$ set through $f_\pi$ cf. MILC 07 [687]. Comparison with scale set through $\Upsilon \,2S-1S$ shows negligible change

**Table 106:** Continuum extrapolations/estimation of lattice artefacts in determinations of the $B\rightarrow D \ell \nu$, $B\rightarrow D^* \ell \nu$, $B_{s}\rightarrow D_{s} \ell \nu$ form factors and of $R(D)$

Collab.	Ref.	$N_\mathrm{f}$	$a$ (fm)	Continuum extrapolation	Scale setting
FNAL/MILC 04A	[446]	$2+1$	0.12	Central value obtained from data at a single lattice spacing. Comparison with quenched simulations at different lattice spacings interpreted as indication of small discretisation effects	Relative scale $r_1/a$ set from the static quark potential. Absolute scale $r_1$ set through $\Upsilon \,2S$–$1S$ splitting cf. HPQCD 03 [695]
Atoui 13	[449]	2	0.054, 0.067, 0.085, 0.098	Combined continuum and chiral extrapolation, with linear terms in $a^2$ and $m_\mathrm{sea}$. No dependence on $a$ or $m_\mathrm{sea}$ observed within errors. Stability of results vs. fits with no $m_\mathrm{sea}$ dependence checked	Scale set through $F_\pi$

**Table 107:** Chiral extrapolation/minimum pion mass in determinations of the $B\rightarrow D \ell \nu$, $B\rightarrow D^* \ell \nu$, $B_{s}\rightarrow D_{s} \ell \nu$ form factors and of $R(D)$. For actions with multiple species of pions, masses quoted are the RMS pion masses. The different $M_{\pi ,\mathrm min}$ entries correspond to the different lattice spacings

Collab.	Ref.	$N_\mathrm{f}$	$M_{\pi ,\mathrm min}\,(\mathrm{MeV})$	Description
FNAL/MILC 13B $B \rightarrow D^{*}$	[447]	$2+1$	330, 260, 280, 470, 590	Simultaneous chiral-continuum extrapolation using SU(3) rHMS$\chi$PT. Systematic error estimated by adding higher-order analytic terms, varying the $D^*$–$D$–$\pi$ coupling, and comparison with continuum $\hbox {HM}\chi$PT
FNAL/MILC 13B $B \rightarrow D$	[447]	$2+1$	330, 260, 280, 470	Simultaneous chiral–continuum extrapolation using SU(3) rHMS$\chi$PT supplemented by terms analytic in $(w-1)$ to interpolate at non-zero recoil. Systematic error included in statistical errors via inclusion of NNLO analytic terms and $D^*$–$D$–$\pi$ coupling with Bayesian priors
FNAL/MILC 12A	[453]	$2+1$	400, 440	See below
FNAL/MILC 10	[444]	$2+1$	340, 320, 440, 570	See below
FNAL/MILC 08	[445]	$2+1$	320, 440, 570	Simultaneous chiral–continuum extrapolation using SU(3) rHMS$\chi$PT. Systematic errors estimated by adding higher-order analytic terms and varying the $D^*$–$D$–$\pi$ coupling
FNAL/MILC 04A	[446]	$2+1$	510	Linear extrapolation in the light-quark mass
Atoui 13	[449]	2	270, 300, 270, 410	Combined continuum and chiral extrapolation, with linear terms in $a^2$ and $m_\mathrm{sea}$. No dependence on $a$ or $m_\mathrm{sea}$ observed within errors. Stability of results vs. fits with no $m_\mathrm{sea}$ dependence checked

**Table 108:** Finite-volume effects in determinations of the $B\rightarrow D \ell \nu$, $B\rightarrow D^* \ell \nu$, $B_{s}\rightarrow D_{s} \ell \nu$ form factors and of $R(D)$. Each $L$-entry corresponds to a different lattice spacing, with multiple spatial volumes at some lattice spacings. For actions with multiple species of pions, the lightest pion masses are quoted

Collab.	Ref.	$N_\mathrm{f}$	$L$ (fm)	${M_{\pi ,\mathrm min}}L$	Description
FNAL/MILC 13B $B \rightarrow D^*$	[447]	$2+1$	2.9, 2.9/3.4/3.8, 2.4/2.7/3.4/5.5, 2.4/2.9, 2.4	$\gtrsim 3.8$	Estimate FV error to be negligible using one-loop rHMS$\chi$PT
FNAL/MILC 13B $B \rightarrow D$	[447]	$2+1$	2.9, 2.9/3.4/3.8, 2.4/2.7/3.4/5.5, 2.4/2.9	$\gtrsim 3.8$	FV error estimated to be negligible
FNAL/MILC 12A	[453]	$2+1$	2.5, 2.4	$\gtrsim 3.8$	FV error estimated to be negligible in [454]
FNAL/MILC 10	[444]	$2+1$	2.8, 2.4/3.4, 2.4/2.9, 2.4	$\gtrsim 3.8$	See below
FNAL/MILC 08	[445]	$2+1$	2.4/3.4, 2.4/2.9, 2.4	$\gtrsim 3.8$	Estimate FV error to be negligible using one-loop rHMS$\chi$PT
FNAL/MILC 04A	[446]	$2+1$	2.4/2.9	$\gtrsim 4.5$	No estimate of FV error quoted
Atoui 13	[449]	2	1.7/2.6, 2.1, 2.0/2.7, 2.4	$\gtrsim 3.6$	No volume dependence observed within errors

**Table 109:** Operator renormalisation in determinations of the $B\rightarrow D \ell \nu$, $B\rightarrow D^* \ell \nu$, $B_{s}\rightarrow D_{s} \ell \nu$ form factors and of $R(D)$

Collab.	Ref.	$N_\mathrm{f}$	Ren.	Description
FNAL/MILC 13B $B \rightarrow D^*$	[447]	$2+1$	mNPR	See error estimate for $h_+$ below
FNAL/MILC 13B $B \rightarrow D$	[447]	$2+1$	mNPR	See FNAL/MILC 08A. A 0.4 % perturbative truncation error for $h_+$ (the dominant contributor to $f_+$) is estimated from $\alpha _\mathrm{s}^2$ times the largest one-loop coefficient observed in the relevant mass range for all similar currents, while a conservative 20 % error is taken for $h_-$
FNAL/MILC 12A	[453]	$2+1$	mNPR	Only the relative matching of the spatial and temporal components of currents is relevant for the ratio $R(D)$. Uncertainty for this is estimated to be 0.4 %
FNAL/MILC 10	[444]	$2+1$	mNPR	See below. A 0.3 % perturbative truncation error is estimated
FNAL/MILC 08	[445]	$2+1$	mNPR	Majority of current renormalisation factor cancels in double ratio of lattice correlation functions. Remaining correction calculated with one-loop tadpole-improved lattice perturbation theory. 0.3 % perturbative truncation error estimated from size of one-loop correction on finest ensemble
FNAL/MILC 04A	[446]	$2+1$	mNPR	No explicit estimate of perturbative truncation error
Atoui 13	[449]	2	–	Observables obtained from ratios that do not require renormalisation. Checks performed by comparing with results coming from currents that are renormalised separately with non-perturbative $Z_{V}$

**Table 110:** Heavy quark treatment in determinations of the $B\rightarrow D \ell \nu$, $B\rightarrow D^* \ell \nu$, $B_{s}\rightarrow D_{s} \ell \nu$ form factors and of $R(D)$

Collab.	Ref.	$N_\mathrm{f}$	Action	Description
FNAL/MILC 13B $B \rightarrow D^*$	[447]	$2+1$	Fermilab	See FNAL/MILC 10
FNAL/MILC 13B $B \rightarrow D$	[447]	$2+1$	Fermilab	Heavy-quark discretisation errors estimated from power-counting to be 2 % in $h_+$ (the dominant contributor to $f_+$) and 10 % in $h_-$
FNAL/MILC 12A	[453]	$2+1$	Fermilab	Discretisation errors of form factors estimated via power counting which leads to negligible ($\sim$0.2 %) errors in the ratio $R(D)$
FNAL/MILC 10	[444]	$2+1$	Fermilab	Discretisation errors from heavy quark action estimated to be 1.1 % from power counting and a more detailed theory of cutoff effects
FNAL/MILC 08	[445]	$2+1$	Fermilab	Heavy-quark discretisation errors estimated to be 1.5 % from power counting and comparisons of data at different lattice spacings
FNAL/MILC 04A	[446]	$2+1$	Fermilab	No explicit estimate of heavy-quark discretisation errors
Atoui 13	[449]	2	tmWil	Results obtained from step-scaling in heavy quark mass via the ratio method. Separate continuum limit extrapolations with mild $a^2$ dependence carried out for each mass point separately. Result at physical value of $m_{b}$ obtained by interpolation between data region and known exact HQET limit

### B.7 Notes to Sect. 9 on the strong coupling $\alpha _\mathrm{s}$

#### B.7.1 Renormalisation scale and perturbative behaviour

See Tables 111, 112, 113, and 114.

**Table 111:** Renormalisation scale and perturbative behaviour of $\alpha _\mathrm{s}$ determinations for $N_\mathrm{f}=0$

Collab.	Ref.	$N_\mathrm{f}$	$\alpha _\mathrm{eff}$	Description
Sternbeck 12	[548]	0	0.11–0.18	$\alpha _{\mathrm {T}}(p)$ for $p = 5$–40 GeV. Fitted with four-loop formulae without power corrections ($\beta = 6.0$, 6.4, 6.7, 6.92)
Ilgenfritz 10	[551]	0	0.07–0.9	$\alpha _\mathrm{T}(p)$ for $p = 1$–240 GeV ($\beta = 5.8$, 6.0, 6.2, 6.4, 9.0)
Sternbeck 10	[549]	0	0.07–0.32	$\alpha _{\mathrm {T}}$ for $p = 2.5$–140 GeV, fitted with four-loop formula partially on very small lattices
Brambilla 10	[506]	0	0.22–0.47	$\alpha _{\mathrm {qq}}(1/r)$ for the range $r/r_0 = 0.15$–0.5. Fit of $V(r)$ with three-loop formula (NNNLO) with renormalon subtraction and resummation reproduces the static potential for $r/r_0 = 0.15$–0.45 well
Boucaud 08	[544]	0	0.18–0.35	$\alpha _{\mathrm {T}}(p)$ with $p = 3$–6 GeV. Fitted to four-loop perturbation formula with $1/p^2$ correction
Boucaud 05	[541]	0	0.22–0.55	$\Lambda _{\widetilde{\mathrm{MOM}}_{g,c}}$ using gluon and ghost propagators with $2 \le \mu \le 6$ GeV. Fitted to four-loop perturbation theory
QCDSF- UKQCD 05	[519]	0	0.10–0.15	$\alpha _{\overline{\mathrm{MS}}}(2.63/a)$ computed from the boosted coupling
CP-PACS 04	[483]	0	0.08–0.28	$\alpha _\mathrm{SF}(1/L)$ step-scaling functions at $\alpha _\mathrm{eff} = 0.08, 0.19$, study of continuum limit. Agreement of continuum limit with ALPHA 98
Boucaud 01A	[553]	0	0.18–0.45	$\alpha _\mathrm{MOM}$ with $p = 2.5$–10 GeV. Consistency check of three-loop perturbation formula with gluon condensate. ${\langle }A^2 \rangle$ from $\alpha _\mathrm{MOM}$ and gluon propagator are consistent
Soto 01	[552]	0	0.25–0.36, 0.3–0.36, 0.19–0.24	$$\alpha _{\widetilde{\mathrm{MOM}}}$$ for $$p = 3 $$–$$ 10$$ GeV. Fit with three-loop formula with gluon condensate. Three-loop formula without condensate does not fit the lattice data ($$\beta = 6.0$$, 6.2, 6.8)
Boucaud 00A	[555]	0	0.35–0.55, 0.25–0.45, 0.22–0.28, 0.18–0.22	$\alpha _{\widetilde{\mathrm{MOM}}}$ with $p = 2$–10 GeV. Fitted to three-loop perturbation theory with power correction. Four-loop coefficient is strongly correlated to the power correction coefficient ($\beta = 6.0$, 6.2, 6.4, 6.8)
Boucaud 00B	[554]	0	0.35–0.55, 0.25–0.45, 0.22–0.28, 0.18–0.22	$\alpha _{\mathrm{MOM}, \widetilde{\mathrm{MOM}}}$ with $2 \le \mu \le 10$ GeV. Consistency check of three-loop perturbation formula with gluon condensate. $\beta _2^\mathrm{MOM}=1.5\times \beta _2^{\widetilde{\mathrm{MOM}}}$ is needed ($\beta$ = 6.0, 6.2, 6.4, 6.8)
Becirevic 99A	[557]	0	0.25–0.4	$\alpha _{\widetilde{\mathrm{MOM}}}$ with $p = 2.5$–5.5 GeV
Becirevic 99B	[556]	0	0.18–0.25	$\alpha _{\widetilde{\mathrm{MOM}}}$ from a single lattice spacing with $p = 5.6$–9.5 GeV
SESAM 99	[520]	0	0.15	$\alpha _{V}(3.41/a)$ computed from the boosted coupling
ALPHA 98	[490]	0	0.07–0.28	$\alpha _\mathrm{SF}(1/L)$ step scaling, agreement with three-loop running for $\alpha _\mathrm{eff} < 0.15$
Boucaud 98A	[559]	0	0.35–0.5	$\alpha _{\mathrm {MOM}}$, with $2.1 \le \mu \le 3.9$ GeV. Fitted to three-loop perturbation theory without power correction
Boucaud 98B	[558]	0	0.27–0.50	$\alpha _{\widetilde{\mathrm{MOM}}}$ with $\mu = 2.2$–4.5 GeV
Alles 96	[539]	0	0.35–0.71	$\alpha _{\widetilde{\mathrm{MOM}}}(p)$ with $p=1.8$–3.0 GeV
Wingate 95	[521]	0	0.15	$\alpha _{V}(3.41/a)$ computed from the boosted coupling
Davies 94	[522]	0	0.15	$\alpha _{V}(3.41/a)$ computed from the boosted coupling
Lüscher 93	[480]	0	0.09–0.28	$\alpha _\mathrm{SF}(1/L)$ step scaling, agreement with three-loop running for $\alpha _\mathrm{eff} < 0.17$
UKQCD 92	[494]	0	0.17–0.40	$\alpha _{\mathrm {qq}}(1/r)$ for a single lattice spacing. Fit of $\alpha _{\mathrm {qq}}(1/r)$ to a NLO formula
Bali 92	[507]	0	0.15–0.35	$\alpha _{\mathrm {qq}}(1/r)$ for the lattice spacing used in the analysis. Box size $L\approx 1.05$ fm. Fit of $\alpha _{\mathrm {qq}}(1/r)$ to a NLO formula. $\Lambda _{\overline{\mathrm{MS}}}$ is found to depend on the fit range
El-Khadra 92	[524]	0	0.15, 0.13, 0.12	$\alpha _{\overline{\mathrm{MS}}}(\pi /a)$ from one-loop boosted perturbation theory

**Table 112:** Renormalisation scale and perturbative behaviour of $\alpha _\mathrm{s}$ determinations for $N_\mathrm{f}=2$

Collab.	Ref.	$N_\mathrm{f}$	$\alpha _\mathrm{eff}$	Description
ALPHA 12	[59]	2	See ALPHA 04	Determination of $\Lambda _{\overline{\mathrm{MS}}}/ f_K$ using ALPHA 04
Sternbeck 12	[548]	2	0.17–0.23	$\alpha _{\mathrm {T}}$ for $(r_0 p)^2=200$–2000. Fit with four-loop formula without condensate. Deviation at higher energy is observed
ETM 11C	[505]	2	0.26–0.96	$\alpha _\mathrm{qq}(1/r)$ as computed by us from $\Lambda _{\overline{\mathrm{MS}}}=315$ MeV
				Fit of $V(r)$ with three-loop formula (NNNLO) with renormalon subtraction and resummation reproduces the static potential for $r/r_0=0.2$–0.6 well. One fit range, using $r/a=2-4$ at the smallest lattice spacing corresponds to $\alpha _\mathrm{eff}=0.26$–0.40. In the ${\overline{\mathrm{MS}}}$ scheme one has $\alpha _{\overline{\mathrm{MS}}}(1/r)=0.24$–0.63 and for the restricted fit $\alpha _{\overline{\mathrm{MS}}}(1/r)=0.24$–0.36. Central values taken from $a=0.042$ fm lattice with $L=1.3$ fm and $m_\pi =350$ MeV
ETM 10F	[550]	2	0.24–0.45	$\alpha _T$ for momentum up to 2.6–5.6 GeV. Fitted with four-loop formula with gluon condensate
Sternbeck 10	[549]	2	0.19–0.38	$\alpha _\mathrm {T}$ for $1\le (ap)^2\le 10$. Fitted with four-loop formula
JLQCD 08	[513]	2	0.25–0.30	$\alpha _{\overline{\mathrm{MS}}}(Q)$ for $0.65<(aQ)^2<1.32$. Fit with the perturbative formula with power corrections
QCDSF-UKQCD 05	[519]	2	0.20–0.18	$\alpha _{\overline{\mathrm{MS}}}(1.4/a)$ computed from the boosted coupling
ALPHA 04	[488]	2	0.078–0.44	$\alpha _\mathrm{SF}(1/L)$ step scaling, agreement with three-loop running for $\alpha _\mathrm{s} < 0.2$
ALPHA 01	[489]	2	0.078–0.44	$\alpha _\mathrm{SF}(1/L)$ step scaling, agreement with three-loop running for $\alpha _\mathrm{s} < 0.2$
Boucaud 01B	[540]	2	0.25–0.5	$\alpha _{\widetilde{\mathrm{MOM}}}$ for momentum up to 7 GeV. Fitted with four-loop formula with and without power correction, leading to different results for $\Lambda _{\overline{\mathrm{MS}}}^{(2)}$. Extrapolation of $\alpha _\mathrm{s}(1.3\,\,{\mathrm {GeV}})$ in $N_\mathrm{f}$ from $N_\mathrm{f}=0, 2$ to $N_\mathrm{f}=3$ is made
SESAM 99	[520]	2	0.17	The boosted coupling $\alpha _P(3.41/a)$
Wingate 95	[521]	2	0.18	$\alpha _{V}(3.41/a)$ computed from the boosted coupling
Aoki 94	[523]	2	0.14	$\alpha _{\overline{\mathrm{MS}}}(\pi /a)$ computed from the boosted coupling
Davies 94	[522]	2	0.18	$\alpha _{V}(3.41/a)$ computed from the boosted coupling

**Table 113:** Renormalisation scale for $N_\mathrm{f}=3$

Collab.	Ref.	$N_\mathrm{f}$	$\alpha _\mathrm{eff}$	Description
Bazavov 12	[504]	$2+1$	0.23–0.57	$\alpha _\mathrm{qq}$ computed by us from $\Lambda _{\overline{\mathrm{MS}}}r_0=0.70$. Fit of $V(r)$ with three-loop formula (NNNLO) with renormalon subtraction and resummation reproduces the static potential for $r/r_0=0.135$–0.5 well
Sternbeck 12	[548]	$2+1$	0.19–0.25	$\alpha _\mathrm {T}$ for $(p r_0)^2=200-2000$. Comparison with four-loop formula
JLQCD 10	[512]	$2+1$	0.29–0.35	$\alpha _{\overline{\mathrm{MS}}}(Q)$ for $0.4<(aQ)^2<1.0$. Fit with the perturbative formula with power corrections
HPQCD 10	[73]	$2+1$		Update of $r_1$ and $r_1/a$ in HPQCD 08A
HPQCD 10	[73]	$2+1$	0.12–0.42	Combined range given for $\alpha _{\mathrm {eff}}$ from $R_4$ and $R_6/R_8$. Fit of $R_n, \;n=4\ldots 10$ to NNNLO of the ratios (meaning NNLO for $\alpha _\mathrm{s}$) including $(a m)^{2i}$ terms with $i\le 10$; coefficients constrained by priors
PACS-CS 09A	[487]	$2+1$	0.08–0.27	$\alpha _\mathrm{SF}(1/L)$ step scaling, agreement with three-loop running for $\alpha _\mathrm{s} \le 0.27$
HPQCD 08B	[85]	$2+1$	0.378	Fit to NNNLO of the ratios (meaning NNLO for $\alpha _\mathrm{s}$) at the charm mass including $(a m)^{2i}$ terms with $i\le 2\ldots 4$; coefficients constrained by priors
HPQCD 08A	[515]	$2+1$	0.15–0.4	$\alpha _{\mathrm {V}}(q^*)$ for a variety of short-distance quantities, using same method as in HPQCD 05A
Maltman 08	[518]	$2+1$		Reanalysis of HPQCD 05A for a restricted set of short-distance quantities with similar results
HPQCD 05A	[514]	$2+1$	0.2–0.4	$\alpha _{\mathrm {V}}(q^*)$ for a variety of short-distance quantities

**Table 114:** Renormalisation scale of $\alpha _\mathrm{s}$ determinations for $N_\mathrm{f}=4$

Collab.	Ref.	$N_\mathrm{f}$	$\alpha _\mathrm{eff}$	Description
ETM 13D	[545]	$2+1+1$	0.26–0.7	$\alpha _\mathrm {T}(p)$ $=$ for $p=1.6$–6.5 GeV. Update of [546] with improved power law determination
ETM 12C	[546]	$2+1+1$	0.24–0.38	$\alpha _\mathrm {T}(p)$ for $p=1.7$–6.8 GeV. Fit with four-loop formula with gluon condensate or higher power
ALPHA 10A	[485]	4	0.07–0.28	$\alpha _{\mathrm {SF}}(1/L)$. Comparison with two-, three-loop $\beta$ function
ETM 11D	[547]	$2+1+1$	0.24–0.4	$\alpha _\mathrm {T}(p)$ for $p=3.8-7.1$ GeV with H(4)-procedure. Fit with four-loop formula with gluon condensate
Perez 10	[486]	4	0.06–0.28	$\alpha _{\mathrm {SF}}(1/L)$. Comparison with one-, two-, three-loop $\beta$ function

#### B.7.2 Continuum limit

See Tables 115, 116, 117, and 118.

**Table 115:** Continuum limit for $\alpha _\mathrm{s}$ determinations with $N_\mathrm{f}=0$

Collab.	Ref.	$N_\mathrm{f}$	$a\,\mu$	Description
Sternbeck 12	[548]	0	Four lattice spacings $a\le 0.1$ fm	At $\alpha _\mathrm{s}$ = 0.18, $ap$ = 2.7, 1.5 for $\beta = 6.0, 6.4$
Brambilla 10	[506]	0	At least three lattice spacings with $0.2 \le 2a/r \le 1.1$	Extrapolation of potential differences $V(r)-V(0.51r_0)$ linear in $a^2$ performed in [484] with several lattice spacings
Ilgenfritz 10	[551]	0	$a$ = 0.136, 0.093, 0.068, 0.051 fm ($\beta =$ 5.8, 6.0, 6.2, 6.4), while no value of $a$ is given for $\beta = 9.0$	At $\alpha _\mathrm{s} = 0.3$, $ap =$ 2.0, 1.4, 1.0, 0.8 ($\beta$ = 5.8, 6.0, 6.2, 6.4). For $\beta = 9.0$ at $ap = 1.4$, $\alpha _\mathrm{s} = 0.082$
Sternbeck 10	[549]	0	Eight lattice spacings $a$ = 0.004–0.087 fm ($r_0 = 0.467$ fm)	$\sqrt{3} < ap < \sqrt{12}$
Boucaud 08	[544]	0	$a = 0.1, 0.07, 0.05$ fm	At $\alpha _\mathrm{s} = 0.3$ the data have $ap = 2.6, 1.9, 1.5$
QCDSF/UKQCD 05	[519]	0	Seven lattice spacings with $a = 0.10$–0.028 fm	$r_0/a$, together with $r_0$ = 0.467 fm
Boucaud 05	[541]	0	$$a = 0.1, 0.07, 0.05 $$ fm	At $$\alpha _\mathrm{s} \le 0.3\,ap = 1.9, 1.4, 1.0$$
CP-PACS 04	[483]	0	Four spacings, $a/L = 1/12 - 1/4$	Iwasaki and Lüscher Weisz tree-level improved bulk actions; boundary improvement at tree level, one loop and with two different choices of implementation
Soto 01	[552]	0	$a = 0.07, 0.05, 0.03$ fm	At $\alpha _\mathrm{s} \le 0.3$, the data have $ap = 1.4, 1.0, 0.6$
Boucaud 01A	[553]	0	$a = 0.1, 0.07, 0.05, 0.03$ fm	At $\alpha _\mathrm{s} \le 0.3\,ap = 1.9, 1.4, 1.0, 0.6$
Boucaud 00A	[555]	0	$a = 0.1, 0.07, 0.05, 0.03$ fm	At $\alpha _\mathrm{s} \le 0.3\,ap = 1.9, 1.4, 1.0, 0.6$
Boucaud 00B	[554]	0	$a = 0.1, 0.07, 0.05, 0.03$ fm	At $\alpha _\mathrm{s} \le 0.3\,ap = 1.9, 1.4, 1.0, 0.6$
SESAM 99	[520]	0	One lattice spacing with $$a = 0.086 $$ fm	$$\Upsilon $$ spectrum splitting
Becirevic 99A	[557]	0	$a = 0.07, 0.05$ fm	At $\alpha _\mathrm{s} \le 0.3\,ap = 1.4, 1.0$
Becirevic 99B	[556]	0	$a = 0.1, 0.07, 0.03$ fm	Only $a = 0.03$ fm used to extract $\alpha _\mathrm{s}$. At $\alpha _\mathrm{s} \le 0.3$, $ap = 0.6 - 1.5$
ALPHA 98	[490]	0	Four to six spacings, $a/L = 1/12 - 1/5$ in step-scaling functions (SSF)	One-loop O($a$) boundary improvement, linear extrapolation in $a/L$
				$a/L = 1/8 - 1/5$ for $\alpha _\mathrm{s}\le 0.11$ SSF,
				$a/L = 1/12 - 1/5$ for $0.12 \le \alpha _\mathrm{s}\le 0.20$ SSF,
				$L_\mathrm{max}/r_0$ from [698], where several lattice spacings were used
Boucaud 98A	[559]	0	$$a = 0.1, 0.07, 0.05 $$ fm	At $$\alpha _\mathrm{s} \le 0.3$$, $$ap = 1.9, 1.4, 1.0$$
Boucaud 98B	[558]	0	$a = 0.1, 0.07, 0.05$ fm	At $\alpha _\mathrm{s} \le 0.3$, $ap = 1.9, 1.4, 1.0$
Alles 96	[539]	0	$a \le 0.1$ fm	At $\alpha _\mathrm{s} = 0.35$, $ap = 1.5$
Wingate 95	[521]	0	One lattice spacing with $a = 0.11\,\hbox {fm}$	Charmonium $1S$–$1P$ splitting
Davies 94	[522]	0	One lattice spacing with $a = 0.077\,\hbox {fm}$	$\Upsilon$ spectrum splitting
Lüscher 93	[480]	0	Four or five lattice spacings, $a/L = 1/12 - 1/5$ in step-scaling functions	One-loop O($a$) boundary improvement, linear extrapolation in $a/L$
				$a/L = 1/8 - 1/5$ for $\alpha _\mathrm{s}\le 0.11$ SSF,
				$a/L = 1/10 - 1/5$ for $0.11 \le \alpha _\mathrm{s}\le 0.22$ SSF,
				$a/L = 1/12 - 1/6$ for $0.22 \le \alpha _\mathrm{s}\le 0.28$ SSF,
				$a/L = 1/8.5 - 1/4.5$ for continuum extrapolation of $L_\mathrm{max}/ \sqrt{K}$
UKQCD 92	[494]	0	One lattice spacing with	No continuum limit
			$$0.44 \le 2a/r \le 1.6$$	
Bali 92	[507]	0	One lattice spacing with	No continuum limit
			$$0.4 \le 2a/r \le 1.6 $$	
El-Khadra 92	[524]	0	Three lattice spacings with $a = 0.17,\ 0.11,\ 0.08\,\hbox {fm}$	Charmonium $1S$–$1P$ splitting

**Table 116:** Continuum limit for $\alpha _\mathrm{s}$ determinations with $N_\mathrm{f}=2$

Collab.	Ref.	$N_\mathrm{f}$	$a\,\mu$	Description
ALPHA 12	[59]	2	$a=0.049, 0.066, 0.076$ fm from $f_K$	Two-loop O($a$) boundary improvement, linear extrapolation of $L_{\mathrm {max}} f_\mathrm {K}$ in $a^2$
Sternbeck 12	[548]	2	$a=0.073,0.07,0.06$ fm	At $\alpha _\mathrm{s} = 0.23$, $ap = 2.1, 2.0, 1.7$
ETM 11C	[505]	2	$0.30 \le 2a/r \le 1.0$ $0.67 \le 2a/r \le 1.26$ when $\alpha _\mathrm{s} = 0.3$	Four lattice spacings; continuum limit studied with a particular range in $r$; central result from the smallest lattice spacing, $a = 0.042$ fm
ETM 10F	[550]	2	$a=0.05,0.07,0.08$ fm. Different lattice spacings are patched together	At $\alpha _\mathrm{s}=0.3$, $ap=1.6, 1.3, 1.1$
Sternbeck 10	[549]	2	$a=0.068,0.076,0.082$ fm	At $\alpha _\mathrm{s} \le 0.3$, $ap \ge 1.7$
JLQCD 08	[513]	2	$a=0.12$ fm from $r_0=0.49$ fm	Single lattice spacing, $0.64 < (aQ)^2 < 1.32$. At $\alpha _\mathrm{s} = 0.3$, $ap = 0.81$
QCDSF- UKQCD 05	[519]	2	Four lattice spacings with $a = 0.10$–$0.066\,\hbox {fm}$	$r_0$, together with $r_0 = 0.467\,\hbox {fm}$
ALPHA 04	[488]	2	$a/L=1/8,1/6,1/5,1/4$	One-loop (at weak coupling) and two-loop O($a$) boundary improvement, linear extrapolation of SSF in $(a/L)^2$
ALPHA 01A	[489]	2	$a/L = 1/6, 1/5, 1/4$	One-loop (at weak coupling) and two-loop O($a$) boundary improvement, weighted average of SSF with $a/L=1/5,1/6$
Boucaud 01B	[540]	2	$a = 0.05, 0.07, 0.09$ fm. Data at different lattice spacings are patched together	At $\alpha _\mathrm{s} = 0.3$, $ap = 1.6, 1.3, 0.9$; plain Wilson action with ${O}(a)$ errors
SESAM 99	[520]	2	One lattice spacing with $a = 0.079\,\hbox {fm}$	$\Upsilon$ spectrum splitting
Wingate 95	[521]	2	One lattice spacing with $a = 0.11\,\hbox {fm}$	Charmonium $1S$–$1P$ splitting
Aoki 94	[523]	2	One lattice spacing with $a = 0.10\,\hbox {fm}$	Charmonium $1P$–$1S$ splitting
Davies 94	[522]	2	One lattice spacing with $a = 0.08\,\hbox {fm}$	$\Upsilon$ spectrum splitting

**Table 117:** Continuum limit for $\alpha _\mathrm{s}$ determinations with $N_\mathrm{f}=3$

Collab.	Ref.	$N_\mathrm{f}$	$a\,\mu$	Description
Bazavov 12	[504]	$2+1$	$2a/r=0.6$–2.0	Seven lattice spacings; four lattice spacings with $1.14 \le 2a/r \le 1.5$ when $\alpha _\mathrm{s}(1/r) = 0.3$. $2a/r = 2$ when $\alpha _\mathrm{s}(1/r) = 0.23$ (on the finest lattice)
Sternbeck 12	[548]	$2+1$	$a=0.07$ fm	At $\alpha _\mathrm{s} = 0.23$, $ap = 2.1$
HPQCD 10	[73]	$2+1$	$a\mu =2 a\bar{m}_{h} = 0.61$–1.75	Five lattice spacings; three lattice spacings with $1.0 \le a\mu \le 1.5$ when $\alpha _{R_4}(\mu ) \le 0.3$; three lattice spacings with $1.0 \le a\mu \le 1.5$ when $\alpha _{R_6/R_8}(\mu ) \le 0.33$
JLQCD 10	[512]	$2+1$	$a = 0.11$ fm from $r_0 = 0.49$ fm	Single lattice spacing, $0.4 < (aQ)^2 < 1.0$ for the momentum fit range. At $\alpha _\mathrm{s} = 0.3$, $ap = 0.89$
HPQCD 10	[73]	$2+1$		Update of $r_1$ and $r_1/a$ in HPQCD 08A
PACS-CS 09A	[487]	$2+1$	$a/L=1/8,1/6,1/4$	Tree-level O($a$) boundary improvement, which has been seen to behave better than one loop in simulations [483]; weighted average of $a/L=1/8,1/6$ for step-scaling function which agrees with a linear extrapolation in $a/L$ of all data points of the SSF. Linear extrapolation in $a/L$ of $L_{\mathrm {max}}m_\rho$ with $a/L_{\mathrm {max}} = 1/8, 1/6, 1/4$
HPQCD 08B	[85]	$2+1$	$a\mu =2 a\bar{m}_{h}$ = 0.8, 1.2, 1.7, 2.1	Four lattice spacings with heavy quark mass approximately the charm mass, where $\alpha _{R_4}(\mu ) = 0.38$
HPQCD 08A	[515]	$2+1$	Six lattice spacings with $a = 0.18$–$0.045\,\hbox {fm}$	$r_1$ using $\Upsilon$ spectrum splitting
Maltman 08	[518]	$2+1$	Five lattice spacings with $a = 0.18$–$0.06\,\hbox {fm}$	Reanalysis of HPQCD 05A with additional lattice spacings $a = 0.06$, $0.15\,\hbox {fm}$
HPQCD 05A	[514]	$2+1$	Three lattice spacings with $a = 0.18$–$0.09\,\hbox {fm}$	$r_1$ using $\Upsilon$ spectrum splitting

**Table 118:** Continuum limit for $\alpha _\mathrm{s}$ determinations with $N_\mathrm{f}=4$

Collab.	Ref.	$N_\mathrm{f}$	$a\,\mu$	Description
ETM 13B	[545]	$2+1+1$	$a=0.060, 0.068$ fm from $f_\pi$	For $\alpha _\mathrm{s} \le 0.3$, $ap=1.5,1.7$. Update of [546]
ETM 12C	[546]	$2+1+1$	$a=0.061, 0.078$ from $f_\pi$	Global fit with $(ap)^2$ discretisation effects. For $\alpha _\mathrm{s} \le 0.3$, $ap = 1.5, 2.2$
ETM 11D	[547]	$2+1+1$	$a = 0.061, 0,078$ fm	For $\alpha _\mathrm{s} \le 0.3$, $ap = 1.5, 2.0$
ALPHA 10A	[485]	4	$a/L = 1/4, 1/6, 1/8$	Constant or global linear fit in $(a/L)^2$
Perez 10	[486]	4	$a/L=1/4, 1/6, 1/8$	Linear extrapolation in $(a/L)^2$. One-loop improvement at the boundary

## Footnotes

1 The original group had been set up in the framework of a European Network on Flavour Physics (Flavianet).

2 The WG on semileptonic $D$ and $B$ decays has currently four members, but only three of them belong to lattice collaborations.

3 J. Shigemitsu has withdrawn from FLAG, immediately after completion of the first version of the present paper (arXiv:1310.8555 [hep-lat]), of which she is a co-author. She is listed here in recognition of her full involvement in the review of B(s) and D semileptonic and radiative decays, as well as for her valuable contribution of the whole FLAG effort.

4 We refer the interested reader to a number of good reviews on the subject [11–15].

5 In the case of MILC, we are referring to the staggered root-mean-square average mass of the taste partners (see discussion in Sect. 2.1). The mass of the corresponding taste-Goldstone pion in these simulations is the physical value.

6 Throughout this review, the quark masses $m_{u}$, $m_{d}$ and $m_{s}$ refer to the ${\overline{\mathrm{MS}}}$ scheme at running scale $\mu =2\,\mathrm{GeV}$ and the numerical values are given in MeV units.

7 Since the decomposition of the sum ${\mathcal {L}}_{\mathrm{QCD}}+{\mathcal {L}}_{\mathrm{QED}}$ into two parts is not unique, specifying the QCD part requires a convention. In order to give results for the quark masses in the Standard Model at scale $\mu =2\,\hbox {GeV}$, on the basis of a calculation done within QCD, it is convenient to match the two theories at that scale. We use this convention throughout the present review. Note that a different convention is used in the analysis of the precision measurements carried out in low-energy pion physics (e.g. [26]). When comparing lattice results with experiment, it is important to fix the QCD parameters in accordance with the convention used in the analysis of the experimental data (for a more detailed discussion, see [27–30]).

8 Sometimes, e.g. in [32], the violation of Dashen’s theorem is given in terms of a different quantity, $\bar{\epsilon }\equiv (\Delta _{K^+}^\gamma -\Delta _{K^0}^\gamma )/(\Delta _{\pi ^+}^\gamma -\Delta _{\pi ^0}^\gamma )-1$. This parameter is related to $\epsilon$ used here through $\epsilon =(1-\epsilon _{m})\bar{\epsilon }$. Given the value of $\epsilon _{m}$ (see (9)), these two quantities differ by only 4 %.

9 The pion decay constant represents a QCD matrix element—in the full Standard Model, the one-pion state is not a meaningful notion: the correlation function of the charged axial current does not have a pole at $p^2=M_{\pi ^+}^2$, but a branch cut extending from $M_{\pi ^+}^2$ to $\infty$. The analytic properties of the correlation function and the problems encountered in the determination of $f_\pi$ are thoroughly discussed in [113]. The “experimental” value of $f_\pi$ depends on the convention used when splitting the sum $\mathcal{L}_{\mathrm{QCD}}+\mathcal{L}_{\mathrm{QED}}$ into two parts (compare Sect. 3.1). The lattice determinations of $f_\pi$ do not yet reach the accuracy where this is of significance, but at the precision claimed by the Particle Data Group [114], the numerical value does depend on the convention used [27–29, 113].

10 It is not a trivial matter to perform the data analysis at this precision. In particular, isospin-breaking effects need to be properly accounted for [116–120]. For a review of recent work on this issue, we refer to [121].

11 Fortran programs for the numerical evaluation of the form factor representation in [174] are available on request from Johan Bijnens.

12 Email exchange between HPQCD and FLAG.

13 Since the MILC result is obtained for a charged kaon, we remove the isospin-breaking effect according to the formula $f_K = f_{K^+} (1 - \delta _\mathrm{SU(2)}/2)$, valid at NLO in ChPT, with $\delta _{SU(2)}$ for MILC 10 computed using Eq. (37).

14 More precisely, they are independent of the two or three light quark masses which are explicitly considered in the respective framework. However, all low-energy constants depend on the masses of the remaining quarks $s,c,b,t$ or $c,b,t$ in the SU(2) and SU(3) framework, respectively.

15 Some of the $O(p^6)$ formulae presented below have been derived in an unpublished note by three of us (GC, SD and HL) and Jürg Gasser. We thank him for allowing us to publish them here.

16 Here and in the following, we stick to the notation used in the papers where the $\chi$PT formulae were established, i.e. we work with $F_\pi \equiv f_\pi /\sqrt{2}=92.2(1)\,{\mathrm {MeV}}$ and $F_K\equiv f_K/\sqrt{2}$. The occurrence of different normalisation conventions is not convenient, but avoiding it by reformulating the formulae in terms of $f_\pi$, $f_K$ is not a good way out. Since we are using different symbols, confusion cannot arise.

17 Notice that one could analyse the quark-mass dependence entirely in terms of the parameter $M^2$ defined in Eq. (49) and determine equally well all other LECs. Using the determination of the quark masses described in Sect. 3 one can then extract $B$ or $\Sigma$.

18 By introducing an imaginary isospin chemical potential, the framework can be extended such that the low-lying spectrum of the Dirac operator is also sensitive to the pseudoscalar decay constant $F$ at LO [227].

19 Higher-order systematic effects in the matching with RMT have been investigated in [228, 229].

20 The results for $\Sigma$ and $F$ lie in the same range as the determinations reported in Tables 13 and 14.

21 There are theoretical arguments suggesting that the $\xi$ expansion is preferable to the $x$ expansion, based on the observation that the coefficients in front of the squared logs in (48) are somewhat larger than in (53). This can be traced to the fact that a part of every formula in the $x$ expansion is concerned with locating the position of the pion pole (at the previous order) while in the $\xi$ expansion the knowledge of this position is built in exactly. Numerical evidence supporting this view is presented in [67].

22 This means that for any given lattice formulation one needs to determine additional low-energy constants, often denoted $W_i$. For certain formulations, e.g. the twisted-mass approach, first steps in this direction have already been taken [248].

23 Also in case of [251] and [252] the colour-coding criteria for the $\epsilon$-regime have been applied.

24 There are two naive procedures to symmetrise an asymmetric systematic error: (i) keep the central value untouched and enlarge the smaller error, (ii) shift the central value by half of the difference between the two original errors and enlarge/shrink both errors by the same amount. Our procedure (iii) is to average the results of (i) and (ii). In other words, a result $$c(s)\left( {\begin{array}{c}u\\ \ell \end{array}}\right) $$ with $\ell >u$ is changed into $c+(u-\ell )/4$ with statistical error $s$ and a symmetric systematic error $(u+3\ell )/4$. The case $\ell <u$ is handled accordingly.

25 For instance, for the MILC data this yields $\bar{l}_3=3.32(64)(45)$ and $\bar{l}_4=4.03(16)(17)$ [37].

26 A very recent lattice calculation of $\mathrm{Im}(A_2)$ by the RBC/UKQCD collaboration opens up the possibility of a more accurate determination of $\xi$ using the measured value of $\epsilon '$ [307, 308]. This lattice calculation uses only a single lattice spacing, so we do not quote the resulting value here, but note that it is consistent with that obtained in Ref. [306], with errors estimated to be significantly smaller.

27 This approach is supported by the results of the calculations using partial quenching (see in particular Refs. [77] and [315]), which find that the dependence on sea-quark masses is weaker than that on the valence-quark masses (which itself is very mild).

28 For example, the master formula Eq. (85) no longer holds as written because contributions containing two insertions of $\Delta S=1$ weak Hamiltonians connected by dynamical charm quarks no longer lead to a short-distance $\Delta S=2$ matrix element.

29 We can approximately quantify this as follows. A weighted average of BMW 11, Laiho 11 and RBC/UKQCD 12A using only statistical errors gives $\hat{B}_{K}=0.7640(33)$. Taking $0.0033$ as the total statistical error, a total systematic error of $0.0093$ is needed to obtain the combined total error of $0.0099$ quoted in the text. (We exclude the SWME 13 result from this calculation as it is only consistent with the other results when its relatively large systematic error is included.) We note that this estimate of the total systematic error is larger than the smallest individual systematic error ($0.0084$ from BMW 11).

30 This situation differs from that of calculations of the $K\rightarrow \pi \ell \nu$ form factor, where the physical pion recoil momenta are smaller than $2 \pi /L$. For $K\rightarrow \pi \ell \nu$ it is now standard to use non-periodic (“twisted”) boundary conditions [343, 344] to simulate directly at $q^2=0$; see Sect. 4.3. Some collaborations have also begun to use twisted boundary conditions for $D$ decays [345–348].

31 We note that HFAG currently averages results for neutral and charged $D$-meson decays without first removing the correction due to the Coulomb attraction between the charged final-state particles for the neutral $D$-meson decays.

32 The variance of hadron correlation functions at non-zero momentum is dominated at large Euclidean times by zero-momentum multiparticle states [417]; therefore the noise-to-signal grows more rapidly than for the vanishing momentum case.

33 These calculations are based on an overlapping set of gauge-field ensembles, so their statistical errors are highly correlated. They use different heavy-quark actions, renormalisation methods, and chiral-extrapolation fit functions, however, so we expect their systematic errors to be largely uncorrelated. Therefore we expect that assuming 100 % correlation will lead to a conservative (over)estimate for the errors in $\Delta \zeta ^{B\pi }$.

34 See e.g. [438] and references therein.

35 We note that HFAG currently averages results for neutral and charged $B$-meson decays without first removing the correction due to the Coulomb attraction between the charged final-state particles for the neutral $B$-meson decays.

36 With two-loop ${\mathrm {O}}(a)$ improvement we here mean $c_{t}$ including the $g_0^4$ term and $\tilde{c}_{t}$ with the $g_0^2$ term. For gluonic observables such as the running coupling this is sufficient for cutoff effects being suppressed to ${\mathrm {O}}(g^6 a)$.

37
$\alpha _{\mathrm {V'}}$ is defined by $\Lambda _{{V}'}=\Lambda _{V}$ and $b_i^{{\mathrm {V}}'}=b_i^{\mathrm {V}}$ for $i=0,1,2$ but $b_i=0$ for $i\ge 3$.

38 We have assigned a  for the continuum limit, in Boucaud 00A [555], 00B [554], 01A [553], Soto 01 [552] but these results are from lattices of a very small physical size with finite size effects that are not easily quantified.
